# iEzy-Drug: A Web Server for Identifying the Interaction between Enzymes and Drugs in Cellular Networking

**DOI:** 10.1155/2013/701317

**Published:** 2013-11-26

**Authors:** Jian-Liang Min, Xuan Xiao, Kuo-Chen Chou

**Affiliations:** ^1^Computer Department, Jing-De-Zhen Ceramic Institute, Jing-De-Zhen 333046, China; ^2^Information School, ZheJiang Textile & Fashion College, NingBo 315211, China; ^3^Gordon Life Science Institute, Belmont, MA 02478, USA; ^4^Center of Excellence in Genomic Medicine Research (CEGMR), King Abdulaziz University, Jeddah, Saudi Arabia

## Abstract

With the features of extremely high selectivity and efficiency in catalyzing almost all the chemical reactions in cells, enzymes play vitally important roles for the life of an organism and hence have become frequent targets for drug design. An essential step in developing drugs by targeting enzymes is to identify drug-enzyme interactions in cells. It is both time-consuming and costly to do this purely by means of experimental techniques alone. Although some computational methods were developed in this regard based on the knowledge of the three-dimensional structure of enzyme, unfortunately their usage is quite limited because three-dimensional structures of many enzymes are still unknown. Here, we reported a sequence-based predictor, called “iEzy-Drug,” in which each drug compound was formulated by a molecular fingerprint with 258 feature components, each enzyme by the Chou's pseudo amino acid composition generated via incorporating sequential evolution information and physicochemical features derived from its sequence, and the prediction engine was operated by the fuzzy *K*-nearest neighbor algorithm. The overall success rate achieved by iEzy-Drug via rigorous cross-validations was about 91%. Moreover, to maximize the convenience for the majority of experimental scientists, a user-friendly web server was established, by which users can easily obtain their desired results.

## 1. Introduction

**Figure 1:**
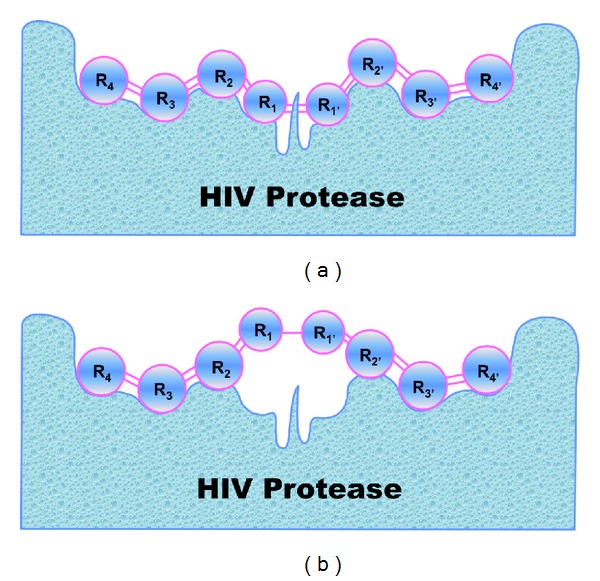
A schematic drawing to illustrate how to use Chou's distorted key theory to develop peptide drugs against HIV/AIDS. (a) shows a good fitting and binding of a peptide to the active site of HIV protease right before it is cleaved by the enzyme. (b) shows that the peptide has become a noncleavable one after its scissile bond is modified although it can still tightly bind to the active site. Such a modified peptide, or ‘‘distorted key”, will automatically become an inhibitor candidate against HIV protease.

Enzymes are biomacromolecules that catalyze almost all the chemical reactions essential for the life of a cell [1]. Most enzymes are proteins although some RNA molecules have been identified to possess the function of enzyme as well. As catalysts, enzymes possess two exceptional features: one is of high efficiency and the other of high selectivity. For instance, the second-order rate constant between some enzymes and their substrates [2] was surprisingly high [3], which could almost reach the upper limit of diffusion-controlled reaction rate according to the calculation and analysis by Chou and coworkers [4–6]. The high selectivity or specificity of enzymes was likened to the “lock-and-key” model, implying that an accurate fit is required between the active site of an enzyme and its substrate for the catalytic reaction to occur. Owing to the previous unique features, enzymes play a crucial role in controlling and regulating the order of chemical reactions in cells that is vitally important for their survival. It is also because of this that enzymes are excellent drug targets, and actually many drugs are enzyme inhibitors. For example, some peptide inhibitors against HIV/AIDS [7–10] and SARS (severe acute respiratory syndrome) [11–13] were based on the Chou's distorted key theory [14], as illustrated in Figure 1, where (a) shows a good fit for a cleavable octapeptide with the active site of HIV-protease and (b) shows that the peptide has become an ideal inhibitor or “distorted key” after its scissile bond is modified. For a brief introduction about the Chou's distorted key theory and its application for designing peptide drugs, see a Wikipedia article at http://en.wikipedia.org/wiki/Chou's_distorted_key_theory_for_peptide_drugs.

To develop enzyme-targeting drugs, an essential step is to identify drug-enzyme interaction in cellular networking [15]. The completion of the human genome project and the emergence of molecular medicine have provided excellent opportunity to discover unknown target enzymes for drugs. Many efforts were made in this regard by computationally analyzing drug-enzyme interactions. The most commonly used approaches are docking simulations (see, e.g., [16–19]) and protein cleavage site analysis (see, e.g., [8, 12, 13]) based on Chou's distorted key theory [14]. However, the latter approach is mainly used to find peptide drugs. Compared with the smaller organic compounds, although peptide drugs have the advantage of low toxicity to human body, they have the shortcoming of poor metabolic stability and low bioavailability due to their inability to readily crossing thru membrane barriers such as the intestinal and blood-brain barriers [20]. In contrast, the molecular docking is indeed a useful vehicle for investigating the interaction of an enzyme receptor with its organic inhibitor and revealing their binding mechanism as demonstrated by a series of studies [11, 19–23]. However, to conduct molecular docking, a necessary prerequisite is the availability of the 3D (three dimensional) structure of the targeted enzyme. Unfortunately, the 3D structures of many enzymes are still unknown. Although X-ray crystallography is a powerful tool in determining the 3D structures of enzymes, it is time-consuming and expensive. Particularly, not all enzymes can be successfully crystallized. For example, membrane enzymes are very difficult to crystallize and most of them will not dissolve in normal solvents. Therefore, so far very few membrane enzyme 3D structures have been determined. Although NMR is indeed a very powerful tool in determining the 3D structures of membrane proteins as indicated by a series of recent publications (see, e.g., [24–30]), it is time-consuming and costly. To acquire the structural information in a timely manner, one has to resort to various structural bioinformatics tools (see, e.g., [18, 31, 32]). Unfortunately the number of templates for developing high quality 3D structures by structural bioinformatics is very limited.

Therefore, it would save us a lot of time and money if we could identify the interactions between enzymes and drugs before carrying out any intense study in this regard. In view of this, the present study was initiated in an attempt to develop a computational method based on the sequence-derived features that can be used to predict the drug-enzyme interactions in cellular networking.

As summarized in a comprehensive review [33] and demonstrated by a series of recent publications [34–37], to successfully develop the desired method, we need to consider the following procedures: (i) construct or select a valid benchmark dataset to train and test the predictor; (ii) denote the drug-enzyme samples with an effective formulation that can truly reflect their intrinsic relation with the target to be predicted; (iii) introduce or develop a powerful algorithm (or engine) to operate the prediction; (iv) conduct a rigorous cross-validation to objectively evaluate its anticipated accuracy; (v) establish a user-friendly web-server for the predictor that is freely accessible to the public. Next, let us elaborate how to deal with these procedures one by one.

## 2. Materials and Methods

### 2.1. Benchmark Dataset

The data used in this study were collected from Kyoto Encyclopedia of Genes and Genomes (KEGG) [38] at http://www.kegg.jp/kegg/, which is a database resource for understanding high-level functions and utilities of the biological system, such as the cell, the organism, and the ecosystem, from molecular-level information, especially large-scale molecular datasets generated by genome sequencing and other high-throughput experimental technologies. For the current study, the benchmark dataset *𝕊* can be formulated as

(1) $$\begin{array}{c} 𝕊={𝕊}^{+}\cup {𝕊}^{-}, \end{array}$$

where *𝕊* ^+^ is the positive subset that consists of the interactive enzyme-drug pairs only, while *𝕊* ^−^ is the negative subset that contains of the noninteractive enzyme-drug pairs only, and the symbol ∪ represents the union in the set theory. Here, the “interactive” pair means the pair whose two counterparts are interacted with each other in the drug-target networks as defined in the KEGG database [38], while the “noninteractive” pair means that its two counter parts are not interacted with each other in the drug-target networks. The positive dataset *𝕊* ^+^ contains 2,719 enzyme-drug pairs derived from Yamanishi et al. [39]. The negative dataset *𝕊* ^−^ contains 5,438 noninteractive enzyme-drug pairs, which were derived according to the following procedures: (i) separating each of the pairs in *𝕊* ^+^ into single drug and enzyme; (ii) recoupling each of the single drugs with each of the single enzymes into pairs in a way that none of them occurred in *𝕊* ^+^; (iii) randomly picking the pairs, thus, formed until they reached the number two times as many as the pairs in *𝕊* ^+^. The 2,719 interactive enzyme-drug pairs and 5,438 noninteractive enzyme-drug pairs are given in Online Supporting Information S1 (see Supplementary Material available online at http://dx.doi.org/10.1155/2013/701317) All the detailed information for the compounds or drugs listed there can be found in the KEGG database via their codes.

### 2.2. Sample Representation

Since each of the samples in the current network system contains an enzyme (protein) and a drug, a combination of the following two approaches was adopted to represent the enzyme-drug pair samples.

#### 2.2.1. Drug

*(a) 2D Molecular Fingerprints*. Although the number of drugs is extremely large, most of them are small organic molecules and are composed of some fixed small structures [40]. The identification of small molecules or structures can be used to detect the drug-target interactions [41]. Molecular fingerprints are bit-string representations of molecular structure and properties [42]. It should be pointed out that there are many types of structural representations that have been suggested for the description of drug molecules, including physicochemical properties [43], chemical graphs [44], topological indices [45], 3D pharmacophore patterns, and molecular fields. In the current study, let us use the simple and generally adopted 2D molecular fingerprints to represent drug molecules, as described below.

First, for each of the drugs concerned, we can obtain a MOL file from the KEGG database [38] via its code that contains the detailed information of chemical structure. Second, we can convert the MOL file format into its 2D molecular fingerprint file format by using a chemical toolbox software called OpenBabel [46], which can be downloaded from the website at http://openbabel.org/. The current version of OpenBabel can generate four types of fingerprints: FP2, FP3, FP4, and MACCS. In the current study, we used the FP2 fingerprint format. It is a path-based fingerprint that identifies small molecule fragments based on all linear and ring substructures and maps them onto a bit-string using a hash function (somewhat similar to the daylight fingerprints [47, 48]). It is a length of 256-bit hexadecimal string obtained from the OpenBabel, and we can convert it to a 256-bit vector. Then, a molecular fingerprint can be formulated as a 256-D vector given by

(2) $$\begin{array}{c} \text{MF}={\left[\begin{array}{ccccc} A_{1} & \cdots & A_{j} & \cdots & A_{256} \end{array}\right]}^{T}, \end{array}$$

where *A*_*j*_ (*j* = 1,2,…, 256) is an integer between 0 and 15, and **T** is the matrix transpose operator.

In order to capture as much useful information from a molecular fingerprint as possible, we can also convert the above 256-bit hexadecimal string into a 1024-bit binary vector, which is a digital sequence only including 0 and 1, and consider two different digital signal characteristics for the digital sequence as follows.

*(b) Information Entropy.* Shannon proposed that any information is redundant, and redundant size is related with the occurrence probability or uncertainty of each symbol such as numbers, letters, or words among the information. The information entropy for a system with a probability distribution *P*(*x*_*i*_) for two classes information entropy [49] is defined as

(3) $$\begin{array}{c} H_{x}=-\sum_{x}P\left(x_{i}\right)\log_{2}P\left(x_{i}\right)\;\left(i=0,1\right), \end{array}$$

where *P*(*x*_*i*_) represents the occurrence probability of number *i* in the aforementioned 1024-bit binary vector and the information entropy *H*_*x*_ is a measure value of the information amount. For example, for the digital sequence 100100011010010, the value of the information entropy *H*_*x*_, thus, obtained is

(4) $$\begin{array}{c} P\left(x_{0}\right)=\frac{9}{15}=0.6,\\ P\left(x_{1}\right)=\frac{6}{15}=0.4,\\ H_{x}=-\left(0.6\times \text{lo}{\text{g}}_{2}0.6+0.4\times \text{lo}{\text{g}}_{2}0.4\right)=0.971. \end{array}$$

*(c) Complexity Factor*. The Lempel-Ziv (LZ) complexity [50] reflects the order that is retained in the sequence, and hence was adopted in this study. For each step only two operations were allowed in the process to get the LZ complexity: either copying the longest section from the part of a nonempty sequence or generating an additional symbol mark that ensures the uniqueness of per component *S*(*i*_*k*−1_ → *i*_*k*_). Its substring is expressed by

(5) $$\begin{array}{c} S\left(i\to j\right)=m_{i}m_{i+1}m_{i+2}\cdots m_{j}\;\left(1\leq i\leq j\leq L\right), \end{array}$$

where *m*_1_ represents the 1st digital value, *m*_2_ the 2nd value, and so forth. A nonempty digital sequence is synthesized according to the following formula:

(6) Syn(S)=S(1→i1)•S(i1+1→i2)•⋯•S(im−1+1→iL).

Suppose that *S* = *m*_1_ *m*_2_ *m*_3_ *m*_4_ *m*_5_ ⋯ *m*_*L*_ has been reconstructed by the subsymbol *m*_*r*_ which is viewed as the newly inserted symbol. The substring up to *m*_*r*_ will be denoted by *S*(1 → *r*)•, where the bold dot • indicates that *m*_*r*_ is a newly inserted symbol for checking whether the rest of the substring *S*(*r* + 1 → *L*) can be reconstructed by a simple process. At first suppose *S*(*q*) = *m*_*r*_ + 1, and see whether *S*(*q*) is the substring for the subsequence *S*(1 → *r*), which means deleting the last symbol from the substring *S*(1 → *r*)*S*(*q*). If the answer is “no”, we insert *S*(*q*) into the sequence followed by a dot •. Thus, it could not be obtained by the same operation. If the answer is “yes”, no new symbol is needed, and we can go on to proceed with *S*(*q*) = *m*_*r*+1_ *m*_*r*+2_ and repeat the same previous procedure. The LZ complexity is the number of dots (plus one if the string is not terminated by a dot). For example, for the sequence 100100011010010, syn(*P*) and the corresponding complexity factor CF are described as

(7) $$\begin{array}{c} \text{Syn}\left(S\right)=1•0•01•000•11•01001•0\\ \text{CF}=7. \end{array}$$

Thus, by adding the information entropy *H*_*x*_ (4) and complexity factor CF (7) into the molecular fingerprint MF (2), we obtained a total of (256 + 1 + 1) = 258 feature elements to represent a drug compound; that is, it can now be formulated as a 258-D vector given by

(8) $$\begin{array}{c} D={\left[\begin{array}{cccccc} A_{1} & A_{2} & \cdots & A_{256} & H_{x} & \text{CF} \end{array}\right]}^{T}, \end{array}$$

where *A*_*i*_ has the same meaning as in (2), while *H*_*x*_and CF are the information entropy and complexity factor, respectively, as described in the previous two sections.

#### 2.2.2. Enzyme

The sequences of the enzymes involved in this study are given in Online Supporting Information S2. Now the problem is how to effectively represent these enzyme sequences for the current study. Generally speaking, there are two kinds of approaches to formulate enzyme sequences: the sequential model and the nonsequential or discrete model [51]. The most typical sequential representation for an enzyme sample **E** with *L* residues is its entire amino acid sequence; that is,

(9) $$\begin{array}{c} E=R_{1}R_{2}R_{3}R_{4}R_{5}R_{6}R_{7}\cdots R_{L}, \end{array}$$

where *R*_1_ represents the 1st residue, *R*_2_ the 2nd residue, and so forth. An enzyme sample thus formulated can contain its most complete information. This is an obvious advantage of the sequential representation. To get the desired results, the sequence-similarity-search-based tools, such as BLAST [52, 53], are usually utilized to conduct the prediction. However, this kind of approach failed to work when the query enzyme did not have significant homology to enzyme of known characters. Thus, various nonsequential representation models were proposed. The simplest nonsequential model for an enzyme was based on its amino acid composition (AAC), as defined by

(10) $$\begin{array}{c} E={\left[\begin{array}{cccc} f_{1} & f_{2} & \cdots & f_{20} \end{array}\right]}^{T}, \end{array}$$

where *f*_*u*_ (*u* = 1,2,…, 20) are the normalized occurrence frequencies of the 20 native amino acids [54–56] in the enzyme **E**, and **T** has the same meaning as in (2) and (8). The AAC-discrete model was widely used for identifying various attributes of proteins (see, e.g., [57–61]). However, as can be seen from (10), all the sequence order effects were lost by using the AAC-discrete model. This is its main shortcoming. To avoid completely losing the sequence-order information, the pseudo amino acid composition [62, 63] or Chou's PseAAC [3] was proposed to replace the simple AAC model. Since the concept of PseAAC was proposed in 2001 [62], it has penetrated into almost all the fields of protein attribute predictions and computational proteomics, such as predicting supersecondary structure [64], predicting metalloproteinase family [65], predicting membrane protein types [66, 67], predicting protein structural class [68], discriminating outer membrane proteins [69], identifying antibacterial peptides [70], identifying allergenic proteins [71], identifying bacterial virulent proteins [72], predicting protein subcellular location [73, 74], identifying GPCRs and their types [75], identifying protein quaternary structural attributes [76], predicting protein submitochondria locations [77], identifying risk type of human papillomaviruses [78], identifying cyclin proteins [79], predicting GABA(A) receptor proteins [80], and predicting cysteine S-nitrosylation sites in proteins [81], among many others (see a long list of papers cited in the References section of [33]). Recently, the concept of PseAAC was further extended to represent the feature vectors of DNA and nucleotides [36, 82], as well as other biological samples (see, e.g., [83, 84]). Because it has been widely and increasingly used, recently two powerful soft-wares called “PseAAC-Builder” [85] and “propy” [86] were established for generating various special Chou's pseudo-amino acid compositions, in addition to the web-server PseAAC [87] built in 2008. According to a recent review [33], the general form of Chou's PseAAC for an enzyme sample can be formulated by

(11) $$\begin{array}{c} E={\left[\begin{array}{cccccc} \psi_{1} & \psi_{2} & \cdots & \psi_{u} & \cdots & \psi_{\Omega } \end{array}\right]}^{T}, \end{array}$$

where the subscript *Ω* is an integer, and its value as well as the components *ψ*_*u*_ (*u* = 1,2,…, *Ω*) will depend on how to extract the desired information from the amino acid sequence of **E** (cf. (10)). Next, let us describe how to extract useful information from the benchmark dataset *𝕊* and Online Supporting Information S2 to define the enzyme samples concerned via (11).

To incorporate as much useful information as possible from an enzyme sample, we are to approach this problem from three different angles, followed by incorporating the feature elements thus obtained into the general form of PseAAC of (11).

*(a) Amino Acid Composition*. The components of amino acid composition have been widely used to predict various protein attributes [57–61]. In this study, they were also included as the first 20 elements in the general Chou's PseAAC (cf. (11)); that is,

(12) $$\begin{array}{c} \psi_{u}=f_{u}\;\left(u=1,2,\ldots ,20\right), \end{array}$$

where *f*_*u*_ has the same meaning as in (10).

*(b) Dipeptide Composition*. Dipeptide composition has been used to predict the protein secondary structural contents [88, 89] as well as various protein attributes (see, e.g., [90–93]). The number of different dipeptides is 20 × 20 = 400. Suppose that the normalized occurrence frequencies of the 400 dipeptides in an enzyme sample are given by

(13) $$\begin{array}{c} f_{u}^{\left(2\right)}\;\left(u=1,2,\ldots ,400\right). \end{array}$$

Incorporating the above 400 dipeptide components into (11), we have

(14) $$\begin{array}{c} \psi_{u+20}=f_{u}^{\left(2\right)}\;\left(u=1,2,\ldots ,400\right). \end{array}$$

*(c) Sequential Evolution Information*. Biology is a natural science with a historic dimension. All biological species have developed starting out from a very limited number of ancestral species. Their evolution involves changes of single residues, insertions and deletions of several residues [94], gene doubling, and gene fusion. With these changes accumulated for a long period of time, many similarities between initial and resultant amino acid sequences are gradually eliminated, but the corresponding proteins may still share many common attributes [18], such as having basically the same biological function and residing at a same subcellular location. To extract the sequential evolution information and use it to define the components of (11), the PSSM (Position Specific Scoring Matrix) was used as described next.

According to Schäffer et al. [95], the sequence evolution information of enzyme **E** with *L* amino acid residues can be expressed by an *L* × 20 matrix, as given by

(15) $$\begin{array}{c} P_{\text{PSSM}}^{\left(0\right)}=\left[\begin{array}{cccc} E_{1\to 1}^{0} & E_{1\to 2}^{0} & \cdots & E_{1\to 20}^{0}\\ E_{2\to 1}^{0} & E_{2\to 2}^{0} & \cdots & E_{2\to 20}^{0}\\ \vdots & \vdots & \vdots & \vdots \\ E_{L\to 1}^{0} & E_{L\to 2}^{0} & \cdots & E_{L\to 20}^{0} \end{array}\right], \end{array}$$

where *E*_*i*→*j*_^0^ represents the original score of the *i*th amino acid residue (*i* = 1, 2,…, *L*) in the enzyme sequence changed to amino acid type *j* (*j* = 1, 2,…, 20) in the process of evolution. Here, the numerical codes 1, 2,…, 20 are used to represent the 20 native amino acid types denoted by A, C, D, E, F, G, H, I, K, L, M, N, P, Q, R, S, T, V, W, and Y. The *L* × 20 scores in (15) were generated by using PSI-BLAST [96] to search the UniProtKB/Swiss-Prot database (Release 2013-05) through three iterations with 0.001 as the *E* value cutoff for multiple sequence alignment against the sequence of the enzyme **E**. In order to make every element in (15) be scaled from their original score ranges into region of [0, 1], we performed a conversion through the standard sigmoid function to make it become

(16) $$\begin{array}{c} P_{\text{PSSM}}^{\left(1\right)}=\left[\begin{array}{cccc} E_{1\to 1}^{1} & E_{1\to 2}^{1} & \cdots & E_{1\to 20}^{1}\\ E_{2\to 1}^{1} & E_{2\to 2}^{1} & \cdots & E_{2\to 20}^{1}\\ \vdots & \vdots & \vdots & \vdots \\ E_{L\to 1}^{1} & E_{L\to 2}^{1} & \cdots & E_{L\to 20}^{1} \end{array}\right], \end{array}$$

where

(17) $$\begin{array}{c} E_{i\to j}^{1}=\frac{1}{1+e^{-E_{i\to j}^{0}}}\;\left(1\leq i\leq L,1\leq j\leq 20\right). \end{array}$$

Now, we extract the useful information from (16) to define the components of (11) via the following approach:

(18) $$\begin{array}{c} \psi_{u+420}=\ell_{u}\;\left(u=1,2,\ldots ,20\right), \end{array}$$

where

(19) $$\begin{array}{c} \ell_{j}=\frac{1}{L}\times \sum_{k=1}^{L}E_{k\to j}^{1}\;\left(j=1,2,\ldots ,20\right). \end{array}$$

*(d) Grey System Model Approach*. The grey system theory [97] is quite useful in dealing with complicated systems that lack sufficient information, or need to process uncertain information. According to the grey system theory, we can extract the following information from the *j*th column of (16); that is,

(20) $$\begin{array}{c} \left[\begin{array}{c} a_{1}^{j}\\ a_{2}^{j} \end{array}\right]={\left(B_{j}^{T}B_{j}\right)}^{-1}B_{j}^{T}U_{j}\;\left(j=1,2,\ldots ,20\right), \end{array}$$

where

(21) $$\begin{array}{c} B_{j}=\left[\begin{array}{ccc} -E_{2\to j}^{1} & -E_{1\to j}^{1}-0.5E_{2\to j}^{1} & 1\\ -E_{3\to j}^{1} & -\sum_{i=1}^{2}E_{i\to j}^{1}-0.5E_{3\to j}^{1} & 1\\ \vdots & \vdots & \vdots \\ -E_{L\to j}^{1} & -\sum_{i=1}^{L-1}E_{i\to j}^{1}-0.5E_{L\to j}^{1} & 1 \end{array}\right],\\ U_{j}=\left[\begin{array}{c} E_{2\to j}^{1}-E_{1\to j}^{1}\\ E_{3\to j}^{1}-E_{2\to j}^{1}\\ \vdots \\ E_{L\to j}^{1}-E_{L-1\to j}^{1} \end{array}\right]. \end{array}$$

Therefore, based on the grey system theory and (20), we can extract another 20 × 2 = 40 quantities from (16) to define the components of (11); that is,

(22) φj={w1a1j when  j  is  an  odd  numberw2a2jwhen  j  is  an  even  number 1≤j≤20,

where *a*_1_^*j*^ and *a*_2_^*j*^ are given by (20); *w*_1_ and *w*_2_ are weight factors, which were all set to 1 in the current study.

Substituting the elements in (12), (14), (18), and (22), we finally obtain a total of *Ω* = 20 + 400 + 20 + 40 = 480 components for the PseAAC of (11), where

(23) $$\begin{array}{c} \psi_{u}^{}=\{\begin{array}{cc} f_{u}^{} & \text{when}\;\;1\leq u\leq 20\\ f_{u}^{\left(2\right)} & \text{when}\;\;21\leq u\leq 420\\ \ell_{u}^{} & \text{when}\;\;421\leq u\leq 440\\ \varphi_{u} & \text{when}\;\;440\leq u\leq 480. \end{array} \end{array}$$

In other words, in this study (11) or Chou's PseAAC is a 480-D vector, whose 480 components are given by (23) derived from the amino acid composition, dipeptide composition, sequential evolution information, and grey system theory.

*(e) Representing Enzyme-Drug Pairs*. Now the pair between an enzyme molecule **E** and a drug compound **D** can be formulated by combing (8) and (11), as given by

(24) G=D⊕E=[A1⋯A256HxCFψ1⋯ψ480]T,

where **G** represents the enzyme-drug pair, ⊕ the orthogonal sum [51], and each of the (258 + 480) = 738 feature elements is given in (8) and (23).

For the convenience of the later formulation, let us use *x*_*i*_ (*i* = 1,2,…, 738) to represent the 738 components of (24); that is,

(25) $$\begin{array}{c} G={\left[\begin{array}{cccccc} x_{1} & x_{2} & \cdots & x_{i} & \cdots & x_{738} \end{array}\right]}^{T}. \end{array}$$

To optimize the prediction results, different weights were usually tested for each of the elements in (25). However, since it would consume a lot of computational time for a total of 738 weight factors, here let us adopt the normalization approach to deal with this problem as done in [98, 99]; that is, convert *x*_*i*_ in (25) to *y*_*i*_ according to the following equation:

(26) $$\begin{array}{c} y_{i}=\frac{2\text{ta}{\text{n}}^{-1}\left(x_{i}^{}\right)}{\pi }\;\left(i=1,2,\ldots ,738\right), \end{array}$$

where tan^−1^ means arctangent. By means of (26), every component in (25) will be converted into the range of [−1,1]; that is, we have −1 ≤ *y*_*i*_ ≤ 1. As demonstrated in [98, 99], the normalization approach via (26) was quite effective in enhancing the quality of prediction operated in a high dimension space. Therefore, in this study, we would not to take the procedure of optimizing the weight factors, significantly reducing the computational times.

### 2.3. Fuzzy *K*-Nearest Neighbour Algorithm

The *K*-NN (*K*-Nearest Neighbor) classifier is quite popular in pattern recognition community owing to its good performance and simple-to-use feature. According to the *K*-NN rule [100], named also as the “voting *K*-NN rule,” the query sample should be assigned to the subset represented by a majority of its *K* nearest neighbors, as illustrated in Figure 5 of [33].

Fuzzy *K*-NN classification method [101] is a special variation of the *K*-NN classification family. Instead of roughly assigning the label based on a voting from the *K* nearest neighbors, it attempts to estimate the membership values that indicate how much degree the query sample belongs to the classes concerned, Obviously, it is impossible for any characteristic description to contain complete information, which would make the classification ambiguous. In view of this, the fuzzy principle is very reasonable and particularly useful in dealing with complicated biological systems, such as identifying nuclear receptor subfamilies [102], characterizing the structure of fast-folding proteins [103], classifying G protein-coupled receptors [104], predicting protein quaternary structural attributes [105], predicting protein structural classes [106, 107], and so forth.

Next, let us give a brief introduction how to use the fuzzy*K*-NN approach to identify the interactions between the enzymes and the drug compounds in the network concerned.

Supposing that *𝕊*(*N*) = {**G**_1_, **G**_2_,…, **G**_*N*_} is a set of vectors representing *N* enzyme-drug pairs in a training set classified into two classes {*C* ^+^,  *C* ^−^}, where *C* ^+^ denotes the interactive pair class, while *C* ^−^ the noninteractive pair class; 𝕊*(**G**) = {**G**_1_*, **G**_2_*,…, **G**_K_*} ⊂ *𝕊*(*N*) is the subset of the *K* nearest neighbor pairs to the query pair **G**. Thus, the fuzzy membership value for the query pair **G** in the two classes of *𝕊*(*N*) is given by

(27) $$\begin{array}{c} \mu_{}^{+}\left(G\right)=\frac{\sum_{j=1}^{K}\mu_{}^{+}\left(G_{j}^{*}\right)d{\left(G,G_{j}^{*}\right)}^{-2/(\varphi -1)}}{\sum_{j=1}^{K}d{\left(G,G_{j}^{*}\right)}^{-2/(\varphi -1)}},\\ \mu_{}^{-}\left(G\right)=\frac{\sum_{j=1}^{K}\mu_{}^{-}\left(G_{j}^{*}\right)d{\left(G,G_{j}^{*}\right)}^{-2/(\varphi -1)}}{\sum_{j=1}^{K}d{\left(G,G_{j}^{*}\right)}^{-2/(\varphi -1)}}, \end{array}$$

where *K* is the number of the nearest neighbors counted for the query pair **G**; *μ* ^+^(**G**_j_*) and *μ* ^−^(**G**_j_*), the fuzzy membership values of the training sample **G**_j_* to the class *C* ^+^ and *C* ^−^, respectively, as will be further defined next; *d*(**G**,  **G**_j_*), the cosine distance between **G** and its *j*th nearest pair **G**_j_* in the training dataset *𝕊*(*N*); *φ*(>1), the fuzzy coefficient for determining how heavily the distance is weighted when calculating each nearest neighbor's contribution to the membership value. Note that the parameters *K* and *φ* will affect the computation result of (27), and they will be optimized by a grid-search as will be described later. Also, various other metrics can be chosen for *d*(**G**,  **G**_j_*), such as Euclidean distance, Hamming distance [108], and Mahalanobis distance [55, 109].

The quantitative definitions for the aforementioned *μ* ^+^(**G**_j_*) and *μ* ^−^(**G**_j_*) in (27) are given by

(28) μ+(Gj∗)={1,if  Gj∗∈C+0,otherwise,μ−(Gj∗)={1,if  Gj∗∈C−0,otherwise.

Substituting the results obtained by (27) into (28), it follows that if *μ* ^+^(**G**) > *μ* ^−^(**G**) then the query pair **G** is an interactive couple; otherwise, noninteractive. In other words, the outcome can be formulated as

(29) $$\begin{array}{c} G\in \{\begin{array}{cc} C^{+}, & \text{if}\;\;\mu_{}^{+}\left(G\right)>\mu_{}^{-}\left(G\right)\\ C^{-}, & \text{otherwise}. \end{array} \end{array}$$

If there is a tie between *μ* ^+^(**G**) and *μ* ^−^(**G**), the query pair **G** will be randomly assigned to one of the two classes. However, case like that is quite rare and in this study never happened.

**Figure 2:**
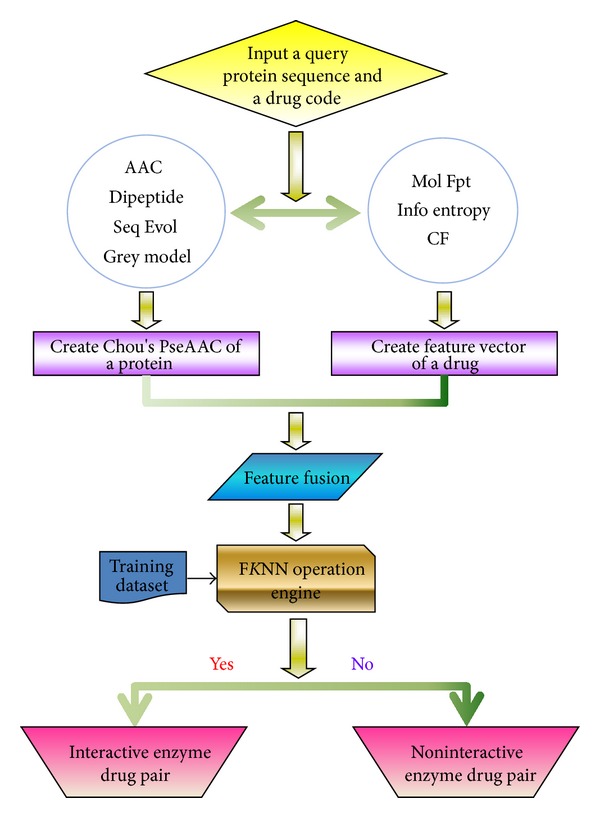
A flowchart to show the operation process of the iEzy-Drug predictor. See the text for further explanation.

The predictor, thus, established is called iEzy-Drug, where “i” means identify, and “Ezy-Drug” means the interaction between enzyme and drug. To provide an intuitive overall picture, a flowchart is provided in Figure 2 to show the process of how the classifier works in identifying enzyme-drug interactions.

### 2.4. Criteria for Performance Evaluation

In the literature, the following equation set is often used for examining the performance quality of a predictor:

(30) $$\begin{array}{c} \text{Sn}=\frac{\text{TP}}{\text{TP}+\text{FN}},\\ \text{Sp}=\frac{\text{TN}}{\text{TN}+\text{FP}},\\ \text{Acc}=\frac{\text{TP}+\text{TN}}{\text{TP}+\text{TN}+\text{FP}+\text{FN}},\\ \text{MCC}=\frac{\left(\text{TP}\times \text{TN}\right)-\left(\text{FP}\times \text{FN}\right)}{\sqrt{\left(\text{TP}+\text{FP}\right)\left(\text{TP}+\text{FN}\right)\left(\text{TN}+\text{FP}\right)\left(\text{TN}+\text{FN}\right)}}, \end{array}$$

where TP represents the true positive; TN, the true negative; FP, the false positive; FN, the false negative; Sn, the sensitivity; Sp, the specificity; Acc, the accuracy; MCC, the Mathew's correlation coefficient.

To most biologists, however, the four metrics as formulated in (30) are not quite intuitive and easier-to-understand, particularly for the Mathew's correlation coefficient. Here, let us adopt the Chou's symbols to formulate the previous four metrics. By means of Chou's symbols [111, 112], the rates of correct predictions for the interactive enzyme-drug pairs in dataset *𝕊* ^+^ and the noninteractive enzyme-drug pairs in dataset *𝕊* ^−^ are, respectively, defined by (cf. (1))

(31) Λ+ =N+−N−+N+, for  the  interactive  enzyme-drug  pairs,Λ−=N−−N+−N−, for  the  noninteractive  enzyme-drug  pairs,

where *N* ^+^ is the total number of the interactive enzyme-drug pairs investigated, while *N*_−_^+^ is the number of the interactive enzyme-drug pairs incorrectly predicted as the noninteractive enzyme-drug pairs; *N* ^−^ is the total number of the noninteractive enzyme-drug pairs investigated, while *N*_+_^−^ is the number of the noninteractive enzyme-drug pairs incorrectly predicted as the interactive enzyme-drug pairs. The overall success prediction rate is given by [113] as follows:

(32) $$\begin{array}{c} \Lambda =\frac{\Lambda^{+}N_{}^{+}+\Lambda^{-}N_{}^{-}}{N_{}^{+}+N^{-}}=1-\frac{N_{-}^{+}+N_{+}^{-}}{N^{+}+N^{-}}. \end{array}$$

It is obvious from (31)-(32) that if and only if none of the interactive enzyme-drug pairs and the noninteractive enzyme-drug pairs are mispredicted; that is, *N*_−_^+^ = *N*_+_^−^ = 0 and Λ^+^ = Λ^−^ = 1, we have the overall success rate Λ = 1. Otherwise, the overall success rate would be smaller than 1.

The relations between the symbols in (32) and those in (30) are given by

(33) $$\begin{array}{c} \text{TP}=N^{+}-N_{-}^{+},\\ \text{TN}=N^{-}-N_{+}^{-},\\ \text{FP}=N_{+}^{-},\\ \text{FN}=N_{-}^{+}. \end{array}$$

Substituting (33) into (30) and also noting (31)-(32), we obtain

(34) $$\begin{array}{c} \text{Sn}=\Lambda^{+}=1-\frac{N_{-}^{+}}{N^{+}},\\ \text{Sp}=\Lambda^{-}=1-\frac{N_{+}^{-}}{N^{-}},\\ \text{Acc}=\Lambda =1-\frac{N_{-}^{+}+N_{+}^{-}}{N^{+}+N^{-}},\\ \text{MCC}=\frac{1-\left(\left(N_{-}^{+}/N_{}^{+}\right)+\left(N_{+}^{-}/N^{-}\right)\right)}{\sqrt{\left(1+\left(N_{+}^{-}-N_{-}^{+}\right)/N^{+}\right)\left(1+\left(N_{-}^{+}-N_{+}^{-}\right)/N^{-}\right)}}. \end{array}$$

Now it is obvious to see from (34): when *N*_−_^+^ = 0 meaning none of the interactive enzyme-drug pairs was mispredicted to be a noninteractive enzyme-drug pair, we have the sensitivity Sn = 1; while *N*_−_^+^ = *N* ^+^ meaning that all the interactive enzyme-drug pairs were mispredicted to be the noninteractive enzyme-drug pairs, we have the sensitivity Sn = 0. Likewise, when *N*_+_^−^ = 0 meaning none of the noninteractive enzyme-drug pairs was mispredicted, we have the specificity Sp = 1; while *N*_+_^−^ = *N* ^−^ meaning all the noninteractive enzyme-drug pairs were incorrectly predicted as interactive enzyme-drug pairs, we have the specificity Sp = 0. When *N*_−_^+^ = *N*_+_^−^ = 0 meaning that none of the interactive enzyme-drug pairs in the dataset *𝕊* ^+^ and none of the noninteractive enzyme-drug pairs in *𝕊* ^−^ was incorrectly predicted, we have the overall accuracy Acc = Λ = 1; while *N*_−_^+^ = *N* ^+^ and *N*_+_^−^ = *N* ^−^ meaning that all the interactive enzyme-drug pairs in the dataset *𝕊* ^+^and all the noninteractive enzyme-drug pairs in *𝕊* ^−^ were mispredicted, we have the overall accuracy Acc = Λ = 0. The MCC correlation coefficient is usually used for measuring the quality of binary (two-class) classifications. When *N*_−_^+^ = *N*_+_^−^ = 0 meaning that none of the interactive enzyme-drug pairs in the dataset *𝕊* ^+^ and none of the noninteractive enzyme-drug pairs in *𝕊* ^−^ were mispredicted, we have MCC = 1; when *N*_−_^+^ = *N* ^+^/2 and *N*_+_^−^ = *N* ^−^/2, we have MCC = 0 meaning no better than random prediction; when *N*_−_^+^ = *N* ^+^ and *N*_+_^−^ = *N* ^−^, we have MCC = −1 meaning total disagreement between prediction and observation. As we can see from the previous discussion, it is much more intuitive and easier-to-understand when using (34) to examine a predictor for its sensitivity, specificity, overall accuracy, and Mathew's correlation coefficient. It is instructive to point out that the metrics as defined in (30) and (34) are valid for single label systems; for multilabel systems, a set of more complicated metrics should be used as given in [114].

## 3. Results and Discussion

### 3.1. Cross-Validation

**Figure 3:**
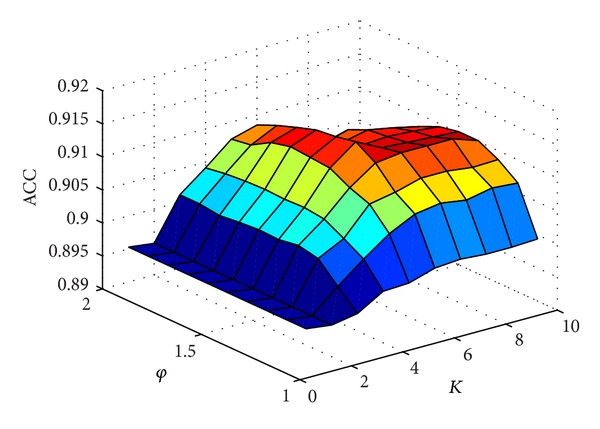
A 3D plot to show how the parameter in (27) was optimized for the iEzy-Drug predictor.

How to properly examine the prediction quality is a key for developing a new predictor and estimating its potential application value. Generally speaking, the following three cross-validation methods are often used to examine a predictor of its effectiveness in practical application: independent dataset test, subsampling or *K*-fold (such as 5-fold, 7-fold, or 10-fold) test, and jackknife test [108]. However, as elaborated by a penetrating analysis in [115], considerable arbitrariness exists in the independent dataset test. Also, as demonstrated by (27)–(29) in [33], the subsampling test (or *K*-fold cross-validation) cannot avoid arbitrariness either. Only the jackknife test is the least arbitrary that can always yield a unique result for a given benchmark dataset. Therefore, the jackknife test has been widely recognized and increasingly utilized by investigators to examine the quality of various predictors (see, e.g., [66, 71, 74, 80]). Accordingly, the success rate by the jackknife test was also used to optimize the two uncertain parameters *K* and *φ* in (27). The result, thus, obtained is shown in Figure 3, from which we obtain when *K* = 6 and *φ* = 1.5 the iEzy-Drug predictor reaches its optimized status.

The success rates thus obtained by the jackknife test in identifying interactive Enzyme-drug pairs or noninteractive enzyme-drug pairs on the benchmark dataset *𝕊* (cf. Online Supporting Information S1) are given in Table 1, where for facilitating comparison, the corresponding result by He et al. [110] is also given. As we can see from the table, the overall accuracy Acc achieved by iEzy-Drug was 91.03%, remarkably higher than 85.48%, the corresponding rate obtained by He et al. [110] on the same benchmark. Furthermore, listed in Table 1 are also the values obtained by iEzy-Drug for the other three metrics; that is, Sn = 90.81%, Sp = 91.14%, and MCC = 80.39%, indicating that the accuracy of iEzy-Drug is not only very high but also quite stale.

**Figure 4:**
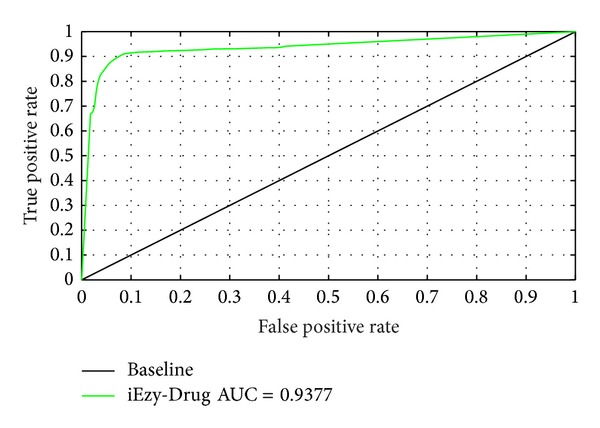
A plot for the ROC curve to quantitatively show the performance of the iEzy-Drug predictor.

To provide a graphical illustration to show the performance of the current binary classifier iEzy-Drug as its discrimination threshold is varied, a 2D plot, called Receiver Operating Characteristic (ROC) curve [116, 117], was also given (Figure 4). In the ROC curve, the vertical coordinate *Y* is for the true positive rate or Sn (cf. (34)), while horizontal coordinate *X* for the false positive rate or 1-Sp. The best possible prediction method would yield a point with the coordinate (0, 1) representing 100% true positive rate (sensitivity Sn) and 0 false positive rate or 100% specificity. Therefore, the (0, 1) point is also called a perfect classification. A completely random guess would give a point along a diagonal from the point (0, 0) to (1, 1). The area under the ROC curve, also called Area Under the ROC (AUROC), is often used to indicate the performance quality of a binary classifier; the value 0.5 of AUROC is equivalent to random prediction, while 1 of AUROC represents a perfect one. As we can see from Figure 4, the AUROC value obtained by iEzy-Drug is 0.9377.

The reason why iEzy-Drug can remarkably improve the prediction quality is that it has introduced the 2D molecular fingerprints to represent drug samples see Online Supporting Information S3 for the detailed fingerprint expressions for the drugs listed in Online Supporting Information S1 and that it has successfully used PseAAC to incorporate the features derived from the sequences of enzymes that are essential for identifying the interaction of enzymes with drugs in the cellular networking.

To enhance the value of its practical applications, the web server for iEzy-Drug has been established that can be freely accessible at http://www.jci-bioinfo.cn/iEzy-Drug/. It is anticipated that the web server will become a useful high throughput tool for both basic research and drug development in the relevant areas, or at the very least play a complementary role to the existing method [39, 110, 118] for which so far no web-server whatsoever has been provided yet.

### 3.2. The Protocol or User Guide

For the convenience of the vast majority of biologists and pharmaceutical scientists, here let us provide a step-by-step guide to show how the users can easily get the desired result by means of the web server without the need to follow the complicated mathematical equations presented in this paper for the process of developing the predictor and its integrity.

**Figure 5:**
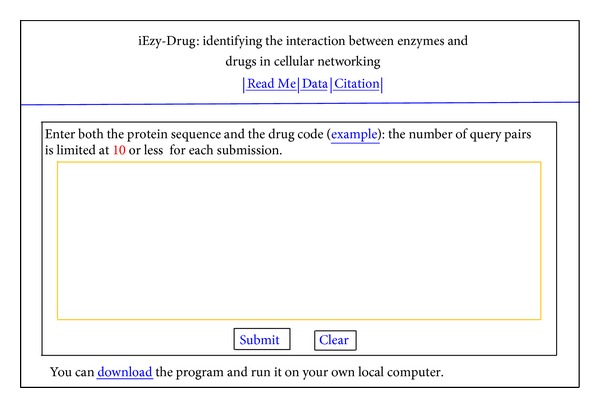
A semiscreenshot to show the top page of the iEzy-Drug web-server. Its web-site address is at http://www.jci-bioinfo.cn/iEzy-Drug/.

*Step 1.* Open the web server at the site http://www.jci-bioinfo.cn/iEzy-Drug/ and you will see the top page of the predictor on your computer screen, as shown in Figure 5. Click on the Read Me button to see a brief introduction about iEzy-Drug predictor and the caveat when using it. 

*Step 2.* Either type or copy/paste the query pairs into the input box at the center of Figure 5. Each query pair consists of two parts: one is for the protein sequence and the other for the drug. The enzyme sequence should be in FASTA format, while the drug in the KEGG code. Examples for the query pairs input can be seen by clicking on the Example button right above the input box. 

*Step 3*. Click on the Submit button to see the predicted result. For example, if you use the four query pairs in the Example window as the input, after clicking the Submit button, you will see on your screen that the “hsa: 10056” enzyme and the “D0021” drug are an interactive pair, and that the “hsa: 100” enzyme and the “D0037” drug are also an interactive pair, but that the “hsa: 3295” enzyme and the “D00889” drug are not an interactive pair, and that the “hsa: 7366” enzyme and the “D03601” drug are not an interactive pair either. All these results are fully consistent with the experimental observations. It takes about 3 minutes before the results are shown on the screen. 

*Step 4*. Click on the Citation button to find the relevant paper that documents the detailed development and algorithm of iEzy-Durg. 

*Step 5*. Click on the Data button to download the benchmark dataset used to train and test the iEzy-Durg predictor. 

*Step 6*. The program code is also available by clicking the button download on the lower panel of Figure 5.

## Supplementary Material

Online Supporting Information S1. The benchmark dataset contains 8,157 enzyme-drug pair samples, of which 2,719 are interactive and 5438 non-interactive. The codes listed here were from the KEGG database at http://www.kegg.jp/kegg/.
Online Supporting Information S1. The benchmark dataset contains 8,157 enzyme-drug pair samples, of which 2,719 are interactive and 5438 non-interactive. The codes listed here were from the KEGG database at http://www.kegg.jp/kegg/.
Online Supporting Information S3. The fingerprints for the drug codes listed in Online Supporting Information S1. Each of these fingerprints is a 256-D vector generated by the OpenBabel software downloaded from http://openbabel.org/.

## Figures and Tables

**Table 1:** The jackknife success rates obtained with iEzy-Drug in identifying interactive enzyme-drug pairs and noninteractive enzyme-drug pairs for the benchmark dataset *𝕊* (cf. Online Supporting Information S1).

Method	Acc	Sn	Sp	MCC
iEzy-Drug^a^	7425/8157 = 91.03%	2469/2719 = 90.81%	4956/5438 = 91.14%	80.39%
NN predictor^b^	85.48%	N/A	N/A	N/A

^a^See (27) where the parameters *K* = 6 and *φ* = 1.5.

^b^See [110].
