# Supplementary material for: iEzy-Drug: A Web Server for Identifying the Interaction between Enzymes and Drugs in Cellular Networking
Source: Biomed Res Int. 2013 Nov 26;2013:701317. doi: 10.1155/2013/701317 (PMC3858977; doi:10.1155/2013/701317)
Supplement: Supplementary file 1 — Online Supporting Information S1. The benchmark dataset contains 8,157 enzyme-drug pair samples, of which 2,719 are interactive and 5438 non-interactive. The codes listed here were from the KEGG database at http://www.kegg.jp/kegg/. Online Supporting Information S1. The benchmark dataset contains 8,157 enzyme-drug pair samples, of which 2,719 are interactive and 5438 non-interactive. The codes listed here were from the KEGG database at http://www.kegg.jp/kegg/. Online Supporting Information S3. The fingerprints for the drug codes listed in Online Supporting Information S1. Each of these fingerprints is a 256-D vector generated by the OpenBabel software downloaded from http://openbabel.org/. [file 701317.f1.pdf]

Online Supporting Information S1. The benchmark dataset contains 8,157 enzyme-drug pair samples, of which 2,719 are interactive and 5,438 non-interactive. All the detailed information for the codes listed here can be found in KEGG database at http://www.kegg.jp/kegg/.

Pair attribute	Enzyme code in KEGG	Drug code in KEGG
Interactive	hsa:10056	D00021
Interactive	hsa:100	D00037
Interactive	hsa:100	D00155
Interactive	hsa:10188	D01441
Interactive	hsa:10269	D00279
Interactive	hsa:10279	D00043
Interactive	hsa:10279	D00160
Interactive	hsa:10295	D00039
Interactive	hsa:10295	D00065
Interactive	hsa:10327	D00136
Interactive	hsa:10327	D02323
Interactive	hsa:1033	D00107
Interactive	hsa:1033	D00184
Interactive	hsa:10461	D01441
Interactive	hsa:10461	D01977
Interactive	hsa:10461	D03218
Interactive	hsa:10461	D03350
Interactive	hsa:10461	D04024
Interactive	hsa:10461	D04025
Interactive	hsa:10549	D00217
Interactive	hsa:10549	D00577
Interactive	hsa:10549	D03670
Interactive	hsa:1056	D01223
Interactive	hsa:1056	D04028
Interactive	hsa:10667	D00021
Interactive	hsa:10720	D01276
Interactive	hsa:10747	D00043
Interactive	hsa:10747	D00160
Interactive	hsa:107	D00002
Interactive	hsa:107	D00045
Interactive	hsa:107	D02769
Interactive	hsa:10825	D00900
Interactive	hsa:10825	D00902
Interactive	hsa:10825	D03829
Interactive	hsa:10846	D00227

Interactive	hsa:10846	D00231
Interactive	hsa:10846	D00371
Interactive	hsa:10846	D00417
Interactive	hsa:10846	D00501
Interactive	hsa:10846	D00528
Interactive	hsa:10846	D00691
Interactive	hsa:10846	D01133
Interactive	hsa:10846	D01198
Interactive	hsa:10846	D01690
Interactive	hsa:10846	D01704
Interactive	hsa:10846	D01712
Interactive	hsa:10846	D02008
Interactive	hsa:10846	D02017
Interactive	hsa:10846	D02042
Interactive	hsa:10846	D02229
Interactive	hsa:10846	D02655
Interactive	hsa:10846	D02731
Interactive	hsa:10858	D00139
Interactive	hsa:10858	D00225
Interactive	hsa:10858	D00380
Interactive	hsa:10858	D00394
Interactive	hsa:10858	D00410
Interactive	hsa:10858	D00437
Interactive	hsa:10858	D00528
Interactive	hsa:10858	D00542
Interactive	hsa:10858	D00574
Interactive	hsa:10858	D01071
Interactive	hsa:10858	D03670
Interactive	hsa:108	D00002
Interactive	hsa:10901	D02335
Interactive	hsa:10924	D00417
Interactive	hsa:10924	D00501
Interactive	hsa:10924	D00528
Interactive	hsa:10935	D00217
Interactive	hsa:10935	D00577
Interactive	hsa:10935	D03670
Interactive	hsa:10941	D01276
Interactive	hsa:109	D00002
Interactive	hsa:10	D00002
Interactive	hsa:10	D00448
Interactive	hsa:11072	D00107
Interactive	hsa:11072	D00184

Interactive	hsa:111	D00002
Interactive	hsa:11202	D00043
Interactive	hsa:11202	D00160
Interactive	hsa:11221	D00107
Interactive	hsa:11221	D00184
Interactive	hsa:11238	D00218
Interactive	hsa:11238	D00340
Interactive	hsa:11238	D00518
Interactive	hsa:11238	D00519
Interactive	hsa:11238	D00538
Interactive	hsa:11238	D00652
Interactive	hsa:11238	D00653
Interactive	hsa:11238	D00655
Interactive	hsa:11238	D01196
Interactive	hsa:11266	D00107
Interactive	hsa:11266	D00184
Interactive	hsa:112	D00002
Interactive	hsa:11330	D00160
Interactive	hsa:11330	D01346
Interactive	hsa:11343	D01223
Interactive	hsa:113	D00002
Interactive	hsa:1147	D00448
Interactive	hsa:114	D00002
Interactive	hsa:115	D00002
Interactive	hsa:116447	D01061
Interactive	hsa:116447	D01432
Interactive	hsa:116447	D01911
Interactive	hsa:116447	D02168
Interactive	hsa:116447	D02756
Interactive	hsa:116447	D04031
Interactive	hsa:1178	D01223
Interactive	hsa:1200	D00043
Interactive	hsa:1215	D00043
Interactive	hsa:1215	D00160
Interactive	hsa:124	D00002
Interactive	hsa:125	D00002
Interactive	hsa:1267	D00417
Interactive	hsa:1267	D00501
Interactive	hsa:1267	D00528
Interactive	hsa:126	D00002
Interactive	hsa:128853	D00107
Interactive	hsa:128853	D00184

Interactive	hsa:128	D00002
Interactive	hsa:129807	D00900
Interactive	hsa:129807	D00902
Interactive	hsa:129807	D03829
Interactive	hsa:130399	D01441
Interactive	hsa:1312	D00781
Interactive	hsa:1312	D00786
Interactive	hsa:131	D00002
Interactive	hsa:132160	D00107
Interactive	hsa:132160	D00184
Interactive	hsa:132	D00423
Interactive	hsa:132	D02769
Interactive	hsa:133121	D00417
Interactive	hsa:133121	D00501
Interactive	hsa:133121	D00528
Interactive	hsa:134510	D00107
Interactive	hsa:134510	D00184
Interactive	hsa:1360	D00037
Interactive	hsa:1363	D00052
Interactive	hsa:1374	D02176
Interactive	hsa:1376	D02176
Interactive	hsa:1384	D02176
Interactive	hsa:13	D01223
Interactive	hsa:13	D02356
Interactive	hsa:142679	D00107
Interactive	hsa:142679	D00184
Interactive	hsa:1429	D03798
Interactive	hsa:1431	D00037
Interactive	hsa:1432	D01840
Interactive	hsa:1432	D03115
Interactive	hsa:1432	D03736
Interactive	hsa:1436	D01441
Interactive	hsa:1436	D01977
Interactive	hsa:1436	D03218
Interactive	hsa:1436	D03350
Interactive	hsa:1436	D04024
Interactive	hsa:1436	D04025
Interactive	hsa:1445	D01441
Interactive	hsa:150290	D00107
Interactive	hsa:150290	D00184
Interactive	hsa:1504	D00160
Interactive	hsa:1504	D01346

Interactive	hsa:151531	D00584
Interactive	hsa:1537	D03670
Interactive	hsa:1543	D00217
Interactive	hsa:1543	D00225
Interactive	hsa:1543	D00380
Interactive	hsa:1543	D00394
Interactive	hsa:1543	D00437
Interactive	hsa:1543	D00528
Interactive	hsa:1543	D00542
Interactive	hsa:1543	D00569
Interactive	hsa:1543	D00574
Interactive	hsa:1543	D00771
Interactive	hsa:1543	D00960
Interactive	hsa:1543	D00964
Interactive	hsa:1543	D01071
Interactive	hsa:1543	D01425
Interactive	hsa:1543	D02451
Interactive	hsa:1543	D03670
Interactive	hsa:1543	D03778
Interactive	hsa:1543	D03781
Interactive	hsa:1543	D03784
Interactive	hsa:1544	D00139
Interactive	hsa:1544	D00217
Interactive	hsa:1544	D00225
Interactive	hsa:1544	D00283
Interactive	hsa:1544	D00380
Interactive	hsa:1544	D00394
Interactive	hsa:1544	D00410
Interactive	hsa:1544	D00437
Interactive	hsa:1544	D00454
Interactive	hsa:1544	D00528
Interactive	hsa:1544	D00542
Interactive	hsa:1544	D00563
Interactive	hsa:1544	D00569
Interactive	hsa:1544	D00574
Interactive	hsa:1544	D00771
Interactive	hsa:1544	D00960
Interactive	hsa:1544	D00964
Interactive	hsa:1544	D01071
Interactive	hsa:1544	D01425
Interactive	hsa:1544	D02451
Interactive	hsa:1544	D03670

Interactive	hsa:1544	D03778
Interactive	hsa:1544	D03781
Interactive	hsa:1544	D03784
Interactive	hsa:1545	D00139
Interactive	hsa:1545	D00225
Interactive	hsa:1545	D00380
Interactive	hsa:1545	D00394
Interactive	hsa:1545	D00410
Interactive	hsa:1545	D00437
Interactive	hsa:1545	D00528
Interactive	hsa:1545	D00542
Interactive	hsa:1545	D00574
Interactive	hsa:1545	D00960
Interactive	hsa:1545	D00964
Interactive	hsa:1545	D01071
Interactive	hsa:1545	D01425
Interactive	hsa:1545	D02451
Interactive	hsa:1545	D03670
Interactive	hsa:1545	D03778
Interactive	hsa:1545	D03781
Interactive	hsa:1545	D03784
Interactive	hsa:1548	D00139
Interactive	hsa:1548	D00225
Interactive	hsa:1548	D00380
Interactive	hsa:1548	D00394
Interactive	hsa:1548	D00410
Interactive	hsa:1548	D00437
Interactive	hsa:1548	D00528
Interactive	hsa:1548	D00542
Interactive	hsa:1548	D00574
Interactive	hsa:1548	D00960
Interactive	hsa:1548	D00964
Interactive	hsa:1548	D01071
Interactive	hsa:1548	D01425
Interactive	hsa:1548	D02451
Interactive	hsa:1548	D03670
Interactive	hsa:1548	D03778
Interactive	hsa:1548	D03781
Interactive	hsa:1548	D03784
Interactive	hsa:1549	D00139
Interactive	hsa:1549	D00225
Interactive	hsa:1549	D00380

Interactive	hsa:1549	D00394
Interactive	hsa:1549	D00410
Interactive	hsa:1549	D00437
Interactive	hsa:1549	D00542
Interactive	hsa:1549	D00574
Interactive	hsa:1549	D00771
Interactive	hsa:1549	D00960
Interactive	hsa:1549	D00964
Interactive	hsa:1549	D01071
Interactive	hsa:1549	D01425
Interactive	hsa:1549	D02451
Interactive	hsa:1549	D03670
Interactive	hsa:1549	D03778
Interactive	hsa:1549	D03781
Interactive	hsa:1549	D03784
Interactive	hsa:1551	D00139
Interactive	hsa:1551	D00225
Interactive	hsa:1551	D00380
Interactive	hsa:1551	D00394
Interactive	hsa:1551	D00410
Interactive	hsa:1551	D00437
Interactive	hsa:1551	D00528
Interactive	hsa:1551	D00542
Interactive	hsa:1551	D00574
Interactive	hsa:1551	D00960
Interactive	hsa:1551	D00964
Interactive	hsa:1551	D01071
Interactive	hsa:1551	D01425
Interactive	hsa:1551	D02451
Interactive	hsa:1551	D03670
Interactive	hsa:1551	D03778
Interactive	hsa:1551	D03781
Interactive	hsa:1551	D03784
Interactive	hsa:1553	D00139
Interactive	hsa:1553	D00225
Interactive	hsa:1553	D00380
Interactive	hsa:1553	D00394
Interactive	hsa:1553	D00410
Interactive	hsa:1553	D00437
Interactive	hsa:1553	D00528
Interactive	hsa:1553	D00542
Interactive	hsa:1553	D00574

Interactive	hsa:1553	D00960
Interactive	hsa:1553	D00964
Interactive	hsa:1553	D01071
Interactive	hsa:1553	D01425
Interactive	hsa:1553	D02451
Interactive	hsa:1553	D03670
Interactive	hsa:1553	D03778
Interactive	hsa:1553	D03781
Interactive	hsa:1553	D03784
Interactive	hsa:1555	D00139
Interactive	hsa:1555	D00225
Interactive	hsa:1555	D00380
Interactive	hsa:1555	D00394
Interactive	hsa:1555	D00410
Interactive	hsa:1555	D00437
Interactive	hsa:1555	D00528
Interactive	hsa:1555	D00542
Interactive	hsa:1555	D00574
Interactive	hsa:1555	D00960
Interactive	hsa:1555	D00964
Interactive	hsa:1555	D01071
Interactive	hsa:1555	D01425
Interactive	hsa:1555	D02451
Interactive	hsa:1555	D03670
Interactive	hsa:1555	D03778
Interactive	hsa:1555	D03781
Interactive	hsa:1555	D03784
Interactive	hsa:1557	D00139
Interactive	hsa:1557	D00225
Interactive	hsa:1557	D00283
Interactive	hsa:1557	D00293
Interactive	hsa:1557	D00322
Interactive	hsa:1557	D00380
Interactive	hsa:1557	D00394
Interactive	hsa:1557	D00410
Interactive	hsa:1557	D00416
Interactive	hsa:1557	D00437
Interactive	hsa:1557	D00512
Interactive	hsa:1557	D00528
Interactive	hsa:1557	D00533
Interactive	hsa:1557	D00536
Interactive	hsa:1557	D00542

Interactive	hsa:1557	D00574
Interactive	hsa:1557	D00882
Interactive	hsa:1557	D00960
Interactive	hsa:1557	D00964
Interactive	hsa:1557	D01071
Interactive	hsa:1557	D01425
Interactive	hsa:1557	D02451
Interactive	hsa:1557	D03670
Interactive	hsa:1557	D03778
Interactive	hsa:1557	D03781
Interactive	hsa:1557	D03784
Interactive	hsa:1558	D00139
Interactive	hsa:1558	D00225
Interactive	hsa:1558	D00252
Interactive	hsa:1558	D00380
Interactive	hsa:1558	D00394
Interactive	hsa:1558	D00410
Interactive	hsa:1558	D00437
Interactive	hsa:1558	D00528
Interactive	hsa:1558	D00542
Interactive	hsa:1558	D00574
Interactive	hsa:1558	D00596
Interactive	hsa:1558	D00960
Interactive	hsa:1558	D00964
Interactive	hsa:1558	D01071
Interactive	hsa:1558	D01425
Interactive	hsa:1558	D02356
Interactive	hsa:1558	D02451
Interactive	hsa:1558	D03670
Interactive	hsa:1558	D03778
Interactive	hsa:1558	D03781
Interactive	hsa:1558	D03784
Interactive	hsa:1558	D05341
Interactive	hsa:1559	D00126
Interactive	hsa:1559	D00139
Interactive	hsa:1559	D00225
Interactive	hsa:1559	D00322
Interactive	hsa:1559	D00380
Interactive	hsa:1559	D00394
Interactive	hsa:1559	D00410
Interactive	hsa:1559	D00416
Interactive	hsa:1559	D00512

Interactive	hsa:1559	D00528
Interactive	hsa:1559	D00542
Interactive	hsa:1559	D00567
Interactive	hsa:1559	D00574
Interactive	hsa:1559	D00593
Interactive	hsa:1559	D00882
Interactive	hsa:1559	D00960
Interactive	hsa:1559	D00964
Interactive	hsa:1559	D01071
Interactive	hsa:1559	D01425
Interactive	hsa:1559	D02451
Interactive	hsa:1559	D03670
Interactive	hsa:1559	D03778
Interactive	hsa:1559	D03781
Interactive	hsa:1559	D03784
Interactive	hsa:1562	D00139
Interactive	hsa:1562	D00225
Interactive	hsa:1562	D00380
Interactive	hsa:1562	D00394
Interactive	hsa:1562	D00410
Interactive	hsa:1562	D00437
Interactive	hsa:1562	D00528
Interactive	hsa:1562	D00542
Interactive	hsa:1562	D00574
Interactive	hsa:1562	D00960
Interactive	hsa:1562	D00964
Interactive	hsa:1562	D01071
Interactive	hsa:1562	D01425
Interactive	hsa:1562	D02451
Interactive	hsa:1562	D03670
Interactive	hsa:1562	D03778
Interactive	hsa:1562	D03781
Interactive	hsa:1562	D03784
Interactive	hsa:1565	D00139
Interactive	hsa:1565	D00217
Interactive	hsa:1565	D00225
Interactive	hsa:1565	D00234
Interactive	hsa:1565	D00274
Interactive	hsa:1565	D00283
Interactive	hsa:1565	D00300
Interactive	hsa:1565	D00380
Interactive	hsa:1565	D00394

Interactive	hsa:1565	D00410
Interactive	hsa:1565	D00437
Interactive	hsa:1565	D00454
Interactive	hsa:1565	D00503
Interactive	hsa:1565	D00521
Interactive	hsa:1565	D00528
Interactive	hsa:1565	D00542
Interactive	hsa:1565	D00563
Interactive	hsa:1565	D00574
Interactive	hsa:1565	D00960
Interactive	hsa:1565	D00964
Interactive	hsa:1565	D01071
Interactive	hsa:1565	D01164
Interactive	hsa:1565	D01425
Interactive	hsa:1565	D02342
Interactive	hsa:1565	D02451
Interactive	hsa:1565	D02671
Interactive	hsa:1565	D03670
Interactive	hsa:1565	D03778
Interactive	hsa:1565	D03781
Interactive	hsa:1565	D03784
Interactive	hsa:1571	D00131
Interactive	hsa:1571	D00139
Interactive	hsa:1571	D00217
Interactive	hsa:1571	D00225
Interactive	hsa:1571	D00380
Interactive	hsa:1571	D00394
Interactive	hsa:1571	D00410
Interactive	hsa:1571	D00437
Interactive	hsa:1571	D00528
Interactive	hsa:1571	D00543
Interactive	hsa:1571	D00544
Interactive	hsa:1571	D00547
Interactive	hsa:1571	D00574
Interactive	hsa:1571	D00771
Interactive	hsa:1571	D00960
Interactive	hsa:1571	D00964
Interactive	hsa:1571	D01071
Interactive	hsa:1571	D01425
Interactive	hsa:1571	D02451
Interactive	hsa:1571	D03670
Interactive	hsa:1571	D03778

Interactive	hsa:1571	D03781
Interactive	hsa:1571	D03784
Interactive	hsa:1572	D00139
Interactive	hsa:1572	D00225
Interactive	hsa:1572	D00380
Interactive	hsa:1572	D00394
Interactive	hsa:1572	D00410
Interactive	hsa:1572	D00437
Interactive	hsa:1572	D00528
Interactive	hsa:1572	D00542
Interactive	hsa:1572	D00574
Interactive	hsa:1572	D00960
Interactive	hsa:1572	D00964
Interactive	hsa:1572	D01071
Interactive	hsa:1572	D01425
Interactive	hsa:1572	D02451
Interactive	hsa:1572	D03670
Interactive	hsa:1572	D03778
Interactive	hsa:1572	D03781
Interactive	hsa:1572	D03784
Interactive	hsa:1573	D00139
Interactive	hsa:1573	D00225
Interactive	hsa:1573	D00380
Interactive	hsa:1573	D00394
Interactive	hsa:1573	D00410
Interactive	hsa:1573	D00437
Interactive	hsa:1573	D00528
Interactive	hsa:1573	D00542
Interactive	hsa:1573	D00574
Interactive	hsa:1573	D00960
Interactive	hsa:1573	D00964
Interactive	hsa:1573	D01071
Interactive	hsa:1573	D01425
Interactive	hsa:1573	D02451
Interactive	hsa:1573	D03670
Interactive	hsa:1573	D03778
Interactive	hsa:1573	D03781
Interactive	hsa:1573	D03784
Interactive	hsa:1576	D00136
Interactive	hsa:1576	D00139
Interactive	hsa:1576	D00225
Interactive	hsa:1576	D00234

Interactive	hsa:1576	D00252
Interactive	hsa:1576	D00274
Interactive	hsa:1576	D00283
Interactive	hsa:1576	D00293
Interactive	hsa:1576	D00364
Interactive	hsa:1576	D00380
Interactive	hsa:1576	D00387
Interactive	hsa:1576	D00394
Interactive	hsa:1576	D00410
Interactive	hsa:1576	D00434
Interactive	hsa:1576	D00437
Interactive	hsa:1576	D00503
Interactive	hsa:1576	D00521
Interactive	hsa:1576	D00528
Interactive	hsa:1576	D00542
Interactive	hsa:1576	D00550
Interactive	hsa:1576	D00563
Interactive	hsa:1576	D00574
Interactive	hsa:1576	D00960
Interactive	hsa:1576	D00964
Interactive	hsa:1576	D01071
Interactive	hsa:1576	D01425
Interactive	hsa:1576	D01973
Interactive	hsa:1576	D02356
Interactive	hsa:1576	D02451
Interactive	hsa:1576	D03670
Interactive	hsa:1576	D03778
Interactive	hsa:1576	D03781
Interactive	hsa:1576	D03784
Interactive	hsa:1577	D00139
Interactive	hsa:1577	D00225
Interactive	hsa:1577	D00380
Interactive	hsa:1577	D00394
Interactive	hsa:1577	D00410
Interactive	hsa:1577	D00437
Interactive	hsa:1577	D00528
Interactive	hsa:1577	D00542
Interactive	hsa:1577	D00574
Interactive	hsa:1577	D00960
Interactive	hsa:1577	D00964
Interactive	hsa:1577	D01071
Interactive	hsa:1577	D01425

Interactive	hsa:1577	D02451
Interactive	hsa:1577	D03670
Interactive	hsa:1577	D03778
Interactive	hsa:1577	D03781
Interactive	hsa:1577	D03784
Interactive	hsa:1579	D00139
Interactive	hsa:1579	D00225
Interactive	hsa:1579	D00380
Interactive	hsa:1579	D00394
Interactive	hsa:1579	D00410
Interactive	hsa:1579	D00437
Interactive	hsa:1579	D00528
Interactive	hsa:1579	D00542
Interactive	hsa:1579	D00574
Interactive	hsa:1579	D01071
Interactive	hsa:1580	D00139
Interactive	hsa:1580	D00225
Interactive	hsa:1580	D00380
Interactive	hsa:1580	D00394
Interactive	hsa:1580	D00410
Interactive	hsa:1580	D00437
Interactive	hsa:1580	D00528
Interactive	hsa:1580	D00542
Interactive	hsa:1580	D00574
Interactive	hsa:1580	D00960
Interactive	hsa:1580	D00964
Interactive	hsa:1580	D01071
Interactive	hsa:1580	D01425
Interactive	hsa:1580	D02451
Interactive	hsa:1580	D03670
Interactive	hsa:1580	D03778
Interactive	hsa:1580	D03781
Interactive	hsa:1580	D03784
Interactive	hsa:1581	D00139
Interactive	hsa:1581	D00225
Interactive	hsa:1581	D00380
Interactive	hsa:1581	D00394
Interactive	hsa:1581	D00410
Interactive	hsa:1581	D00437
Interactive	hsa:1581	D00528
Interactive	hsa:1581	D00542
Interactive	hsa:1581	D00574

Interactive	hsa:1581	D01071
Interactive	hsa:1581	D03670
Interactive	hsa:1582	D00139
Interactive	hsa:1582	D00225
Interactive	hsa:1582	D00380
Interactive	hsa:1582	D00394
Interactive	hsa:1582	D00410
Interactive	hsa:1582	D00437
Interactive	hsa:1582	D00528
Interactive	hsa:1582	D00542
Interactive	hsa:1582	D00574
Interactive	hsa:1582	D01071
Interactive	hsa:1582	D03670
Interactive	hsa:1583	D00139
Interactive	hsa:1583	D00225
Interactive	hsa:1583	D00380
Interactive	hsa:1583	D00394
Interactive	hsa:1583	D00437
Interactive	hsa:1583	D00528
Interactive	hsa:1583	D00542
Interactive	hsa:1583	D00574
Interactive	hsa:1583	D01071
Interactive	hsa:1583	D03670
Interactive	hsa:1584	D00139
Interactive	hsa:1584	D00225
Interactive	hsa:1584	D00380
Interactive	hsa:1584	D00394
Interactive	hsa:1584	D00410
Interactive	hsa:1584	D00437
Interactive	hsa:1584	D00528
Interactive	hsa:1584	D00542
Interactive	hsa:1584	D00574
Interactive	hsa:1584	D01071
Interactive	hsa:1584	D03670
Interactive	hsa:1585	D00139
Interactive	hsa:1585	D00225
Interactive	hsa:1585	D00380
Interactive	hsa:1585	D00394
Interactive	hsa:1585	D00528
Interactive	hsa:1585	D00542
Interactive	hsa:1585	D00574
Interactive	hsa:1585	D01071

Interactive	hsa:1585	D03670
Interactive	hsa:1586	D00139
Interactive	hsa:1586	D00225
Interactive	hsa:1586	D00380
Interactive	hsa:1586	D00394
Interactive	hsa:1586	D00410
Interactive	hsa:1586	D00437
Interactive	hsa:1586	D00528
Interactive	hsa:1586	D00542
Interactive	hsa:1586	D00574
Interactive	hsa:1586	D01071
Interactive	hsa:1586	D03670
Interactive	hsa:1586	D03781
Interactive	hsa:1586	D03784
Interactive	hsa:1588	D00139
Interactive	hsa:1588	D00153
Interactive	hsa:1588	D00225
Interactive	hsa:1588	D00380
Interactive	hsa:1588	D00394
Interactive	hsa:1588	D00410
Interactive	hsa:1588	D00437
Interactive	hsa:1588	D00528
Interactive	hsa:1588	D00542
Interactive	hsa:1588	D00574
Interactive	hsa:1588	D00960
Interactive	hsa:1588	D00963
Interactive	hsa:1588	D00964
Interactive	hsa:1588	D01071
Interactive	hsa:1588	D01425
Interactive	hsa:1588	D02451
Interactive	hsa:1588	D03670
Interactive	hsa:1588	D03778
Interactive	hsa:1588	D03781
Interactive	hsa:1588	D03784
Interactive	hsa:1589	D00139
Interactive	hsa:1589	D00225
Interactive	hsa:1589	D00380
Interactive	hsa:1589	D00394
Interactive	hsa:1589	D00410
Interactive	hsa:1589	D00437
Interactive	hsa:1589	D00528
Interactive	hsa:1589	D00542

Interactive	hsa:1589	D01071
Interactive	hsa:1589	D03670
Interactive	hsa:1593	D00139
Interactive	hsa:1593	D00188
Interactive	hsa:1593	D00225
Interactive	hsa:1593	D00380
Interactive	hsa:1593	D00394
Interactive	hsa:1593	D00410
Interactive	hsa:1593	D00437
Interactive	hsa:1593	D00528
Interactive	hsa:1593	D00542
Interactive	hsa:1593	D00574
Interactive	hsa:1593	D01071
Interactive	hsa:1593	D03670
Interactive	hsa:1594	D00139
Interactive	hsa:1594	D00187
Interactive	hsa:1594	D00225
Interactive	hsa:1594	D00380
Interactive	hsa:1594	D00394
Interactive	hsa:1594	D00410
Interactive	hsa:1594	D00437
Interactive	hsa:1594	D00528
Interactive	hsa:1594	D00542
Interactive	hsa:1594	D00574
Interactive	hsa:1594	D01071
Interactive	hsa:1594	D03670
Interactive	hsa:1595	D00139
Interactive	hsa:1595	D00225
Interactive	hsa:1595	D00380
Interactive	hsa:1595	D00394
Interactive	hsa:1595	D00410
Interactive	hsa:1595	D00437
Interactive	hsa:1595	D00528
Interactive	hsa:1595	D00542
Interactive	hsa:1595	D00574
Interactive	hsa:1595	D01071
Interactive	hsa:1595	D02556
Interactive	hsa:1595	D03670
Interactive	hsa:1610	D00005
Interactive	hsa:1610	D00038
Interactive	hsa:1621	D00131
Interactive	hsa:1621	D03787

Interactive	hsa:1633	D02368
Interactive	hsa:1636	D00198
Interactive	hsa:1636	D00251
Interactive	hsa:1636	D00362
Interactive	hsa:1636	D00383
Interactive	hsa:1636	D00421
Interactive	hsa:1636	D00459
Interactive	hsa:1636	D00620
Interactive	hsa:1636	D00621
Interactive	hsa:1636	D00622
Interactive	hsa:1636	D00623
Interactive	hsa:1636	D00624
Interactive	hsa:1636	D01069
Interactive	hsa:1636	D01119
Interactive	hsa:1636	D01549
Interactive	hsa:1636	D01667
Interactive	hsa:1636	D01900
Interactive	hsa:1636	D03077
Interactive	hsa:1636	D03440
Interactive	hsa:1636	D03752
Interactive	hsa:1636	D03753
Interactive	hsa:1636	D03756
Interactive	hsa:1636	D03758
Interactive	hsa:1636	D03760
Interactive	hsa:1636	D03763
Interactive	hsa:1636	D03765
Interactive	hsa:1636	D03767
Interactive	hsa:1636	D03769
Interactive	hsa:1636	D03772
Interactive	hsa:1636	D03773
Interactive	hsa:1636	D03775
Interactive	hsa:1636	D03776
Interactive	hsa:1645	D00136
Interactive	hsa:1675	D00043
Interactive	hsa:1675	D00160
Interactive	hsa:1719	D00142
Interactive	hsa:1719	D00145
Interactive	hsa:1719	D00285
Interactive	hsa:1719	D00488
Interactive	hsa:1719	D02115
Interactive	hsa:1719	D02487
Interactive	hsa:1719	D06238

Interactive	hsa:1723	D00055
Interactive	hsa:1725	D00002
Interactive	hsa:1728	D00005
Interactive	hsa:1728	D00208
Interactive	hsa:1728	D03798
Interactive	hsa:1800	D02194
Interactive	hsa:1803	D00043
Interactive	hsa:1806	D00005
Interactive	hsa:1806	D00027
Interactive	hsa:1806	D00584
Interactive	hsa:1843	D00107
Interactive	hsa:1843	D00184
Interactive	hsa:1844	D00107
Interactive	hsa:1844	D00184
Interactive	hsa:1845	D00107
Interactive	hsa:1845	D00184
Interactive	hsa:1846	D00107
Interactive	hsa:1846	D00184
Interactive	hsa:1847	D00107
Interactive	hsa:1847	D00184
Interactive	hsa:1848	D00107
Interactive	hsa:1848	D00184
Interactive	hsa:1849	D00107
Interactive	hsa:1849	D00184
Interactive	hsa:1850	D00107
Interactive	hsa:1850	D00184
Interactive	hsa:1852	D00107
Interactive	hsa:1852	D00184
Interactive	hsa:1890	D01223
Interactive	hsa:189	D00369
Interactive	hsa:18	D00332
Interactive	hsa:18	D00535
Interactive	hsa:191	D00002
Interactive	hsa:1956	D01441
Interactive	hsa:1956	D01977
Interactive	hsa:1956	D03218
Interactive	hsa:1956	D03350
Interactive	hsa:1956	D04024
Interactive	hsa:1956	D04025
Interactive	hsa:196883	D00002
Interactive	hsa:1969	D01441
Interactive	hsa:1969	D01977

Interactive	hsa:1969	D03218
Interactive	hsa:1969	D03350
Interactive	hsa:1969	D04024
Interactive	hsa:1969	D04025
Interactive	hsa:1990	D00160
Interactive	hsa:1991	D00160
Interactive	hsa:1991	D01918
Interactive	hsa:1991	D03788
Interactive	hsa:199974	D00139
Interactive	hsa:199974	D00225
Interactive	hsa:199974	D00380
Interactive	hsa:199974	D00394
Interactive	hsa:199974	D00410
Interactive	hsa:199974	D00437
Interactive	hsa:199974	D00528
Interactive	hsa:199974	D00542
Interactive	hsa:199974	D00574
Interactive	hsa:199974	D00960
Interactive	hsa:199974	D00964
Interactive	hsa:199974	D01071
Interactive	hsa:199974	D01425
Interactive	hsa:199974	D02451
Interactive	hsa:199974	D03670
Interactive	hsa:199974	D03778
Interactive	hsa:199974	D03781
Interactive	hsa:199974	D03784
Interactive	hsa:2041	D01441
Interactive	hsa:2041	D01977
Interactive	hsa:2041	D03218
Interactive	hsa:2041	D03350
Interactive	hsa:2041	D04024
Interactive	hsa:2041	D04025
Interactive	hsa:2042	D01441
Interactive	hsa:2042	D01977
Interactive	hsa:2042	D03218
Interactive	hsa:2042	D03350
Interactive	hsa:2042	D04024
Interactive	hsa:2042	D04025
Interactive	hsa:2043	D01441
Interactive	hsa:2043	D01977
Interactive	hsa:2043	D03218
Interactive	hsa:2043	D03350

Interactive	hsa:2043	D04024
Interactive	hsa:2043	D04025
Interactive	hsa:2044	D01441
Interactive	hsa:2044	D01977
Interactive	hsa:2044	D03218
Interactive	hsa:2044	D03350
Interactive	hsa:2044	D04024
Interactive	hsa:2044	D04025
Interactive	hsa:2045	D01441
Interactive	hsa:2045	D01977
Interactive	hsa:2045	D03218
Interactive	hsa:2045	D03350
Interactive	hsa:2045	D04024
Interactive	hsa:2045	D04025
Interactive	hsa:2046	D01441
Interactive	hsa:2046	D01977
Interactive	hsa:2046	D03218
Interactive	hsa:2046	D03350
Interactive	hsa:2046	D04024
Interactive	hsa:2046	D04025
Interactive	hsa:2047	D01441
Interactive	hsa:2047	D01977
Interactive	hsa:2047	D03218
Interactive	hsa:2047	D03350
Interactive	hsa:2047	D04024
Interactive	hsa:2047	D04025
Interactive	hsa:2048	D01441
Interactive	hsa:2048	D01977
Interactive	hsa:2048	D03218
Interactive	hsa:2048	D03350
Interactive	hsa:2048	D04024
Interactive	hsa:2048	D04025
Interactive	hsa:2049	D01441
Interactive	hsa:2049	D01977
Interactive	hsa:2049	D03218
Interactive	hsa:2049	D03350
Interactive	hsa:2049	D04024
Interactive	hsa:2049	D04025
Interactive	hsa:2050	D01441
Interactive	hsa:2050	D01977
Interactive	hsa:2050	D03218
Interactive	hsa:2050	D03350

Interactive	hsa:2050	D04024
Interactive	hsa:2050	D04025
Interactive	hsa:2051	D01441
Interactive	hsa:2051	D01977
Interactive	hsa:2051	D03218
Interactive	hsa:2051	D03350
Interactive	hsa:2051	D04024
Interactive	hsa:2051	D04025
Interactive	hsa:2058	D00035
Interactive	hsa:2064	D01441
Interactive	hsa:2064	D01977
Interactive	hsa:2064	D03218
Interactive	hsa:2064	D03350
Interactive	hsa:2064	D04024
Interactive	hsa:2064	D04025
Interactive	hsa:2065	D01441
Interactive	hsa:2065	D01977
Interactive	hsa:2065	D03218
Interactive	hsa:2065	D03350
Interactive	hsa:2065	D04024
Interactive	hsa:2065	D04025
Interactive	hsa:2066	D01441
Interactive	hsa:2066	D01977
Interactive	hsa:2066	D03218
Interactive	hsa:2066	D03350
Interactive	hsa:2066	D04024
Interactive	hsa:2066	D04025
Interactive	hsa:2098	D00043
Interactive	hsa:2098	D01223
Interactive	hsa:2135	D01828
Interactive	hsa:2147	D00270
Interactive	hsa:2147	D00494
Interactive	hsa:2147	D00560
Interactive	hsa:2147	D00733
Interactive	hsa:2147	D01981
Interactive	hsa:2147	D02335
Interactive	hsa:2147	D03722
Interactive	hsa:2147	D03728
Interactive	hsa:2155	D00043
Interactive	hsa:2155	D00160
Interactive	hsa:2155	D02335
Interactive	hsa:2158	D00043

Interactive	hsa:2158	D00160
Interactive	hsa:2158	D02335
Interactive	hsa:2159	D00043
Interactive	hsa:2159	D00160
Interactive	hsa:2159	D01844
Interactive	hsa:2159	D02335
Interactive	hsa:2159	D04029
Interactive	hsa:2160	D00043
Interactive	hsa:2160	D00160
Interactive	hsa:2161	D00043
Interactive	hsa:2161	D00160
Interactive	hsa:216	D00002
Interactive	hsa:216	D00094
Interactive	hsa:216	D00131
Interactive	hsa:217	D00002
Interactive	hsa:217	D00131
Interactive	hsa:2180	D02769
Interactive	hsa:2185	D01441
Interactive	hsa:218	D00131
Interactive	hsa:2193	D00021
Interactive	hsa:219	D00131
Interactive	hsa:2224	D01968
Interactive	hsa:2232	D00005
Interactive	hsa:2235	D03670
Interactive	hsa:223	D00131
Interactive	hsa:2241	D01441
Interactive	hsa:2242	D01441
Interactive	hsa:224	D00131
Interactive	hsa:225689	D01840
Interactive	hsa:225689	D03115
Interactive	hsa:225689	D03736
Interactive	hsa:2260	D01441
Interactive	hsa:2260	D01977
Interactive	hsa:2260	D03218
Interactive	hsa:2260	D03350
Interactive	hsa:2260	D04024
Interactive	hsa:2260	D04025
Interactive	hsa:2261	D01441
Interactive	hsa:2261	D01977
Interactive	hsa:2261	D03218
Interactive	hsa:2261	D03350
Interactive	hsa:2261	D04024

Interactive	hsa:2261	D04025
Interactive	hsa:2263	D01441
Interactive	hsa:2263	D01977
Interactive	hsa:2263	D03218
Interactive	hsa:2263	D03350
Interactive	hsa:2263	D04024
Interactive	hsa:2263	D04025
Interactive	hsa:2264	D01441
Interactive	hsa:2264	D01977
Interactive	hsa:2264	D03218
Interactive	hsa:2264	D03350
Interactive	hsa:2264	D04024
Interactive	hsa:2264	D04025
Interactive	hsa:2280	D00753
Interactive	hsa:22843	D00107
Interactive	hsa:22843	D00184
Interactive	hsa:22954	D00283
Interactive	hsa:22954	D00451
Interactive	hsa:22954	D00513
Interactive	hsa:22954	D00726
Interactive	hsa:22978	D00423
Interactive	hsa:22978	D00501
Interactive	hsa:23035	D00107
Interactive	hsa:23035	D00184
Interactive	hsa:231	D00037
Interactive	hsa:231	D00136
Interactive	hsa:231	D01688
Interactive	hsa:231	D01715
Interactive	hsa:231	D01842
Interactive	hsa:231	D02323
Interactive	hsa:231	D02328
Interactive	hsa:231	D02835
Interactive	hsa:231	D03803
Interactive	hsa:231	D03805
Interactive	hsa:231	D03806
Interactive	hsa:231	D03807
Interactive	hsa:2321	D01441
Interactive	hsa:2321	D01977
Interactive	hsa:2321	D03218
Interactive	hsa:2321	D03350
Interactive	hsa:2321	D04024
Interactive	hsa:2321	D04025

Interactive	hsa:2322	D01441
Interactive	hsa:2322	D01977
Interactive	hsa:2322	D03218
Interactive	hsa:2322	D03350
Interactive	hsa:2322	D04024
Interactive	hsa:2322	D04025
Interactive	hsa:23236	D00417
Interactive	hsa:23236	D00501
Interactive	hsa:23236	D00528
Interactive	hsa:23239	D00107
Interactive	hsa:23239	D00184
Interactive	hsa:2324	D01441
Interactive	hsa:2324	D01977
Interactive	hsa:2324	D03218
Interactive	hsa:2324	D03350
Interactive	hsa:2324	D04024
Interactive	hsa:2324	D04025
Interactive	hsa:2326	D00005
Interactive	hsa:2326	D00401
Interactive	hsa:2328	D00120
Interactive	hsa:2328	D00401
Interactive	hsa:2339	D03720
Interactive	hsa:2342	D03720
Interactive	hsa:23430	D00043
Interactive	hsa:23430	D00160
Interactive	hsa:23436	D00043
Interactive	hsa:23436	D00160
Interactive	hsa:23475	D00049
Interactive	hsa:2356	D00070
Interactive	hsa:23632	D00218
Interactive	hsa:23632	D00340
Interactive	hsa:23632	D00518
Interactive	hsa:23632	D00519
Interactive	hsa:23632	D00538
Interactive	hsa:23632	D00652
Interactive	hsa:23632	D00653
Interactive	hsa:23632	D00655
Interactive	hsa:23632	D01196
Interactive	hsa:238	D01441
Interactive	hsa:238	D01977
Interactive	hsa:238	D03218
Interactive	hsa:238	D03350

Interactive	hsa:238	D04024
Interactive	hsa:238	D04025
Interactive	hsa:239	D00097
Interactive	hsa:239	D00118
Interactive	hsa:239	D00126
Interactive	hsa:239	D00132
Interactive	hsa:239	D00141
Interactive	hsa:239	D00448
Interactive	hsa:239	D00510
Interactive	hsa:239	D01332
Interactive	hsa:240	D00097
Interactive	hsa:240	D00118
Interactive	hsa:240	D00126
Interactive	hsa:240	D00132
Interactive	hsa:240	D00141
Interactive	hsa:240	D00377
Interactive	hsa:240	D00414
Interactive	hsa:240	D00448
Interactive	hsa:240	D00510
Interactive	hsa:240	D01332
Interactive	hsa:240	D03882
Interactive	hsa:242	D00097
Interactive	hsa:242	D00118
Interactive	hsa:242	D00126
Interactive	hsa:242	D00132
Interactive	hsa:242	D00141
Interactive	hsa:242	D00448
Interactive	hsa:242	D00510
Interactive	hsa:242	D01332
Interactive	hsa:246	D00097
Interactive	hsa:246	D00118
Interactive	hsa:246	D00132
Interactive	hsa:246	D00141
Interactive	hsa:246	D00448
Interactive	hsa:246	D00510
Interactive	hsa:246	D01332
Interactive	hsa:246	D01364
Interactive	hsa:247	D00097
Interactive	hsa:247	D00118
Interactive	hsa:247	D00132
Interactive	hsa:247	D00141
Interactive	hsa:247	D00448

Interactive	hsa:247	D00510
Interactive	hsa:247	D01332
Interactive	hsa:2534	D01441
Interactive	hsa:2548	D00216
Interactive	hsa:2548	D00625
Interactive	hsa:2548	D01665
Interactive	hsa:2548	D03433
Interactive	hsa:25796	D01223
Interactive	hsa:25824	D00038
Interactive	hsa:25824	D00217
Interactive	hsa:25824	D00577
Interactive	hsa:25824	D03670
Interactive	hsa:2582	D00002
Interactive	hsa:2595	D00216
Interactive	hsa:2595	D00625
Interactive	hsa:2595	D01665
Interactive	hsa:2595	D03433
Interactive	hsa:2597	D00002
Interactive	hsa:25	D01441
Interactive	hsa:260293	D00139
Interactive	hsa:260293	D00225
Interactive	hsa:260293	D00380
Interactive	hsa:260293	D00394
Interactive	hsa:260293	D00410
Interactive	hsa:260293	D00437
Interactive	hsa:260293	D00528
Interactive	hsa:260293	D00542
Interactive	hsa:260293	D00574
Interactive	hsa:260293	D00960
Interactive	hsa:260293	D00964
Interactive	hsa:260293	D01071
Interactive	hsa:260293	D01425
Interactive	hsa:260293	D02451
Interactive	hsa:260293	D03670
Interactive	hsa:260293	D03778
Interactive	hsa:260293	D03781
Interactive	hsa:260293	D03784
Interactive	hsa:26279	D01223
Interactive	hsa:2638	D02315
Interactive	hsa:2639	D00005
Interactive	hsa:2673	D00332
Interactive	hsa:2677	D00148

Interactive	hsa:2677	D02335
Interactive	hsa:27032	D00542
Interactive	hsa:27032	D00543
Interactive	hsa:27032	D00544
Interactive	hsa:27032	D00545
Interactive	hsa:27032	D00546
Interactive	hsa:27032	D00547
Interactive	hsa:27034	D00005
Interactive	hsa:27034	D00039
Interactive	hsa:270	D02769
Interactive	hsa:27115	D00227
Interactive	hsa:27115	D00231
Interactive	hsa:27115	D00371
Interactive	hsa:27115	D00417
Interactive	hsa:27115	D00501
Interactive	hsa:27115	D00528
Interactive	hsa:27115	D00691
Interactive	hsa:27115	D01133
Interactive	hsa:27115	D01198
Interactive	hsa:27115	D01332
Interactive	hsa:27115	D01690
Interactive	hsa:27115	D01704
Interactive	hsa:27115	D01712
Interactive	hsa:27115	D02008
Interactive	hsa:27115	D02017
Interactive	hsa:27115	D02042
Interactive	hsa:27115	D02229
Interactive	hsa:27115	D02655
Interactive	hsa:27115	D02731
Interactive	hsa:2746	D00002
Interactive	hsa:2746	D00007
Interactive	hsa:2766	D03798
Interactive	hsa:279	D00216
Interactive	hsa:279	D00625
Interactive	hsa:279	D01346
Interactive	hsa:2806	D00332
Interactive	hsa:28227	D00107
Interactive	hsa:28227	D00184
Interactive	hsa:2822	D00417
Interactive	hsa:2822	D00501
Interactive	hsa:2822	D00528
Interactive	hsa:284541	D00139

Interactive	hsa:284541	D00225
Interactive	hsa:284541	D00380
Interactive	hsa:284541	D00394
Interactive	hsa:284541	D00410
Interactive	hsa:284541	D00437
Interactive	hsa:284541	D00528
Interactive	hsa:284541	D00542
Interactive	hsa:284541	D00574
Interactive	hsa:284541	D01071
Interactive	hsa:284541	D03670
Interactive	hsa:285220	D01441
Interactive	hsa:285220	D01977
Interactive	hsa:285220	D03218
Interactive	hsa:285220	D03350
Interactive	hsa:285220	D04024
Interactive	hsa:285220	D04025
Interactive	hsa:2880	D00577
Interactive	hsa:2882	D00577
Interactive	hsa:28972	D00043
Interactive	hsa:28972	D00160
Interactive	hsa:28	D00203
Interactive	hsa:28	D00222
Interactive	hsa:28	D00317
Interactive	hsa:28	D00884
Interactive	hsa:28	D00885
Interactive	hsa:2936	D00002
Interactive	hsa:2936	D00005
Interactive	hsa:2936	D00014
Interactive	hsa:2950	D00014
Interactive	hsa:29785	D00139
Interactive	hsa:29785	D00225
Interactive	hsa:29785	D00380
Interactive	hsa:29785	D00394
Interactive	hsa:29785	D00410
Interactive	hsa:29785	D00437
Interactive	hsa:29785	D00528
Interactive	hsa:29785	D00542
Interactive	hsa:29785	D00574
Interactive	hsa:29785	D00960
Interactive	hsa:29785	D00964
Interactive	hsa:29785	D01071
Interactive	hsa:29785	D01425

Interactive	hsa:29785	D02451
Interactive	hsa:29785	D03670
Interactive	hsa:29785	D03778
Interactive	hsa:29785	D03781
Interactive	hsa:29785	D03784
Interactive	hsa:29920	D00035
Interactive	hsa:29941	D01441
Interactive	hsa:29968	D00332
Interactive	hsa:3001	D00043
Interactive	hsa:3001	D00160
Interactive	hsa:3002	D00043
Interactive	hsa:3002	D00160
Interactive	hsa:3028	D00002
Interactive	hsa:3033	D00002
Interactive	hsa:3034	D00032
Interactive	hsa:3035	D00032
Interactive	hsa:3055	D01441
Interactive	hsa:3067	D00032
Interactive	hsa:306	D00417
Interactive	hsa:306	D00501
Interactive	hsa:306	D00528
Interactive	hsa:30814	D01223
Interactive	hsa:30833	D00501
Interactive	hsa:30	D00222
Interactive	hsa:30	D00317
Interactive	hsa:30	D00333
Interactive	hsa:30	D00342
Interactive	hsa:30	D00391
Interactive	hsa:30	D00398
Interactive	hsa:30	D05407
Interactive	hsa:313	D01223
Interactive	hsa:3141	D00029
Interactive	hsa:3156	D00359
Interactive	hsa:3156	D00434
Interactive	hsa:3156	D00887
Interactive	hsa:3156	D00889
Interactive	hsa:3156	D00892
Interactive	hsa:3156	D00893
Interactive	hsa:3156	D01862
Interactive	hsa:3156	D01915
Interactive	hsa:3156	D02258
Interactive	hsa:3156	D03601

Interactive	hsa:3156	D03643
Interactive	hsa:3156	D03816
Interactive	hsa:31	D00007
Interactive	hsa:31	D00027
Interactive	hsa:31	D00029
Interactive	hsa:326625	D01027
Interactive	hsa:327	D00043
Interactive	hsa:3283	D01180
Interactive	hsa:3290	D00410
Interactive	hsa:3291	D00410
Interactive	hsa:3292	D00002
Interactive	hsa:3295	D00002
Interactive	hsa:32	D00029
Interactive	hsa:3376	D00065
Interactive	hsa:339221	D00417
Interactive	hsa:339221	D00501
Interactive	hsa:339221	D00528
Interactive	hsa:3416	D00052
Interactive	hsa:3480	D01441
Interactive	hsa:3480	D01977
Interactive	hsa:3480	D03218
Interactive	hsa:3480	D03350
Interactive	hsa:3480	D04024
Interactive	hsa:3480	D04025
Interactive	hsa:349565	D00002
Interactive	hsa:34	D00005
Interactive	hsa:353	D02769
Interactive	hsa:354	D00043
Interactive	hsa:354	D00160
Interactive	hsa:3551	D00097
Interactive	hsa:3551	D00120
Interactive	hsa:3551	D00448
Interactive	hsa:35	D00005
Interactive	hsa:3612	D00501
Interactive	hsa:3614	D00423
Interactive	hsa:3614	D00752
Interactive	hsa:3615	D00002
Interactive	hsa:3615	D00752
Interactive	hsa:3643	D01441
Interactive	hsa:3643	D01977
Interactive	hsa:3643	D03218
Interactive	hsa:3643	D03350

Interactive	hsa:3643	D04024
Interactive	hsa:3643	D04025
Interactive	hsa:3643	D04966
Interactive	hsa:3645	D01441
Interactive	hsa:3645	D01977
Interactive	hsa:3645	D03218
Interactive	hsa:3645	D03350
Interactive	hsa:3645	D04024
Interactive	hsa:3645	D04025
Interactive	hsa:3645	D04966
Interactive	hsa:36	D00065
Interactive	hsa:3702	D01441
Interactive	hsa:3712	D00005
Interactive	hsa:3716	D01441
Interactive	hsa:3717	D01441
Interactive	hsa:3718	D01441
Interactive	hsa:3735	D02304
Interactive	hsa:377677	D00218
Interactive	hsa:377677	D00340
Interactive	hsa:377677	D00518
Interactive	hsa:377677	D00519
Interactive	hsa:377677	D00538
Interactive	hsa:377677	D00652
Interactive	hsa:377677	D00653
Interactive	hsa:377677	D00655
Interactive	hsa:377677	D01196
Interactive	hsa:3791	D01441
Interactive	hsa:3791	D01977
Interactive	hsa:3791	D03218
Interactive	hsa:3791	D03350
Interactive	hsa:3791	D04024
Interactive	hsa:3791	D04025
Interactive	hsa:3815	D01441
Interactive	hsa:3815	D01977
Interactive	hsa:3815	D03218
Interactive	hsa:3815	D03350
Interactive	hsa:3815	D04024
Interactive	hsa:3815	D04025
Interactive	hsa:3816	D00043
Interactive	hsa:3816	D00160
Interactive	hsa:3817	D00043
Interactive	hsa:3817	D00160

Interactive	hsa:3818	D00043
Interactive	hsa:3818	D00160
Interactive	hsa:38	D00065
Interactive	hsa:38	D00448
Interactive	hsa:3906	D00126
Interactive	hsa:3906	D00132
Interactive	hsa:3906	D00293
Interactive	hsa:3906	D00510
Interactive	hsa:390956	D00107
Interactive	hsa:3932	D01441
Interactive	hsa:3939	D00002
Interactive	hsa:3945	D00002
Interactive	hsa:3988	D04028
Interactive	hsa:3990	D01223
Interactive	hsa:3990	D04028
Interactive	hsa:3991	D01223
Interactive	hsa:4017	D00270
Interactive	hsa:4023	D00279
Interactive	hsa:4023	D01223
Interactive	hsa:4025	D00097
Interactive	hsa:4025	D00217
Interactive	hsa:4025	D00562
Interactive	hsa:4025	D00577
Interactive	hsa:4025	D03670
Interactive	hsa:4048	D00097
Interactive	hsa:4048	D00118
Interactive	hsa:4048	D00126
Interactive	hsa:4048	D00132
Interactive	hsa:4048	D00141
Interactive	hsa:4048	D00448
Interactive	hsa:4048	D00510
Interactive	hsa:4048	D01332
Interactive	hsa:4051	D00139
Interactive	hsa:4051	D00225
Interactive	hsa:4051	D00380
Interactive	hsa:4051	D00394
Interactive	hsa:4051	D00410
Interactive	hsa:4051	D00437
Interactive	hsa:4051	D00528
Interactive	hsa:4051	D00542
Interactive	hsa:4051	D00574
Interactive	hsa:4051	D01071

Interactive	hsa:4051	D03670
Interactive	hsa:4058	D01441
Interactive	hsa:4058	D01977
Interactive	hsa:4058	D03218
Interactive	hsa:4058	D03350
Interactive	hsa:4058	D04024
Interactive	hsa:4058	D04025
Interactive	hsa:4067	D01441
Interactive	hsa:4128	D00270
Interactive	hsa:4128	D00505
Interactive	hsa:4128	D00785
Interactive	hsa:4128	D00826
Interactive	hsa:4128	D00947
Interactive	hsa:4128	D01097
Interactive	hsa:4128	D01888
Interactive	hsa:4128	D02559
Interactive	hsa:4128	D02560
Interactive	hsa:4128	D02561
Interactive	hsa:4128	D02562
Interactive	hsa:4128	D02563
Interactive	hsa:4128	D02564
Interactive	hsa:4128	D02579
Interactive	hsa:4128	D02580
Interactive	hsa:4128	D02581
Interactive	hsa:4128	D03731
Interactive	hsa:4128	D03733
Interactive	hsa:4129	D00005
Interactive	hsa:4129	D00270
Interactive	hsa:4129	D00505
Interactive	hsa:4129	D00785
Interactive	hsa:4129	D00826
Interactive	hsa:4129	D01097
Interactive	hsa:4129	D01888
Interactive	hsa:4129	D02559
Interactive	hsa:4129	D02560
Interactive	hsa:4129	D02561
Interactive	hsa:4129	D02562
Interactive	hsa:4129	D02563
Interactive	hsa:4129	D02564
Interactive	hsa:4129	D02579
Interactive	hsa:4129	D02580
Interactive	hsa:4129	D02581

Interactive	hsa:4129	D03731
Interactive	hsa:4129	D03733
Interactive	hsa:4129	D05458
Interactive	hsa:4143	D04983
Interactive	hsa:4145	D01441
Interactive	hsa:4190	D00002
Interactive	hsa:4191	D00037
Interactive	hsa:4200	D00002
Interactive	hsa:4200	D02308
Interactive	hsa:4233	D01441
Interactive	hsa:4233	D01977
Interactive	hsa:4233	D03218
Interactive	hsa:4233	D03350
Interactive	hsa:4233	D04024
Interactive	hsa:4233	D04025
Interactive	hsa:4282	D00037
Interactive	hsa:4311	D00052
Interactive	hsa:4329	D00039
Interactive	hsa:4353	D00097
Interactive	hsa:4353	D00217
Interactive	hsa:4353	D00401
Interactive	hsa:4353	D00562
Interactive	hsa:4353	D00577
Interactive	hsa:43	D00043
Interactive	hsa:43	D00196
Interactive	hsa:43	D00487
Interactive	hsa:43	D00667
Interactive	hsa:43	D00670
Interactive	hsa:43	D00805
Interactive	hsa:43	D00994
Interactive	hsa:43	D00995
Interactive	hsa:43	D00998
Interactive	hsa:43	D01001
Interactive	hsa:43	D01223
Interactive	hsa:43	D01228
Interactive	hsa:43	D02068
Interactive	hsa:43	D02173
Interactive	hsa:43	D02193
Interactive	hsa:43	D02418
Interactive	hsa:43	D02558
Interactive	hsa:43	D02729
Interactive	hsa:43	D03751

Interactive	hsa:43	D03822
Interactive	hsa:43	D03823
Interactive	hsa:43	D03826
Interactive	hsa:43	D04292
Interactive	hsa:444	D00131
Interactive	hsa:4482	D04983
Interactive	hsa:4486	D01441
Interactive	hsa:4486	D01977
Interactive	hsa:4486	D03218
Interactive	hsa:4486	D03350
Interactive	hsa:4486	D04024
Interactive	hsa:4486	D04025
Interactive	hsa:4548	D01027
Interactive	hsa:4548	D04983
Interactive	hsa:4552	D01027
Interactive	hsa:4552	D04983
Interactive	hsa:4593	D00726
Interactive	hsa:4593	D01441
Interactive	hsa:4593	D01977
Interactive	hsa:4593	D03218
Interactive	hsa:4593	D03350
Interactive	hsa:4593	D04024
Interactive	hsa:4593	D04025
Interactive	hsa:4594	D01027
Interactive	hsa:4758	D00900
Interactive	hsa:4758	D00902
Interactive	hsa:4758	D03829
Interactive	hsa:4759	D00900
Interactive	hsa:4759	D00902
Interactive	hsa:4759	D03829
Interactive	hsa:476	D00298
Interactive	hsa:476	D00654
Interactive	hsa:476	D00656
Interactive	hsa:476	D00658
Interactive	hsa:476	D01240
Interactive	hsa:4835	D00005
Interactive	hsa:4835	D03798
Interactive	hsa:4837	D00049
Interactive	hsa:4860	D00054
Interactive	hsa:4860	D01370
Interactive	hsa:4881	D00515
Interactive	hsa:4881	D00516

Interactive	hsa:4881	D00630
Interactive	hsa:4907	D00501
Interactive	hsa:4914	D01441
Interactive	hsa:4914	D01977
Interactive	hsa:4914	D03218
Interactive	hsa:4914	D03350
Interactive	hsa:4914	D04024
Interactive	hsa:4914	D04025
Interactive	hsa:4915	D01441
Interactive	hsa:4915	D01977
Interactive	hsa:4915	D03218
Interactive	hsa:4915	D03350
Interactive	hsa:4915	D04024
Interactive	hsa:4915	D04025
Interactive	hsa:4916	D01441
Interactive	hsa:4916	D01977
Interactive	hsa:4916	D03218
Interactive	hsa:4916	D03350
Interactive	hsa:4916	D04024
Interactive	hsa:4916	D04025
Interactive	hsa:4919	D01441
Interactive	hsa:4919	D01977
Interactive	hsa:4919	D03218
Interactive	hsa:4919	D03350
Interactive	hsa:4919	D04024
Interactive	hsa:4919	D04025
Interactive	hsa:4920	D01441
Interactive	hsa:4920	D01977
Interactive	hsa:4920	D03218
Interactive	hsa:4920	D03350
Interactive	hsa:4920	D04024
Interactive	hsa:4920	D04025
Interactive	hsa:4921	D01441
Interactive	hsa:4921	D01977
Interactive	hsa:4921	D03218
Interactive	hsa:4921	D03350
Interactive	hsa:4921	D04024
Interactive	hsa:4921	D04025
Interactive	hsa:4942	D00332
Interactive	hsa:4942	D00475
Interactive	hsa:4953	D00829
Interactive	hsa:495	D00455

Interactive	hsa:495	D01984
Interactive	hsa:49	D00043
Interactive	hsa:49	D00160
Interactive	hsa:501	D00131
Interactive	hsa:5033	D00018
Interactive	hsa:5045	D00043
Interactive	hsa:5045	D00160
Interactive	hsa:50484	D02566
Interactive	hsa:50484	D03670
Interactive	hsa:50487	D01223
Interactive	hsa:5049	D01223
Interactive	hsa:5050	D01223
Interactive	hsa:5051	D01223
Interactive	hsa:5052	D00217
Interactive	hsa:5052	D00577
Interactive	hsa:5052	D03670
Interactive	hsa:5053	D00021
Interactive	hsa:5091	D00029
Interactive	hsa:50940	D00227
Interactive	hsa:50940	D00231
Interactive	hsa:50940	D00371
Interactive	hsa:50940	D00417
Interactive	hsa:50940	D01133
Interactive	hsa:50940	D01198
Interactive	hsa:50940	D01690
Interactive	hsa:50940	D01704
Interactive	hsa:50940	D01712
Interactive	hsa:50940	D02008
Interactive	hsa:50940	D02017
Interactive	hsa:50940	D02042
Interactive	hsa:50940	D02229
Interactive	hsa:50940	D02655
Interactive	hsa:50940	D02731
Interactive	hsa:5095	D00029
Interactive	hsa:5095	D00039
Interactive	hsa:5095	D00041
Interactive	hsa:5095	D00065
Interactive	hsa:5096	D00029
Interactive	hsa:5096	D00039
Interactive	hsa:5096	D00041
Interactive	hsa:5096	D00065
Interactive	hsa:50	D00037

Interactive	hsa:51004	D00139
Interactive	hsa:51004	D00225
Interactive	hsa:51004	D00380
Interactive	hsa:51004	D00394
Interactive	hsa:51004	D00410
Interactive	hsa:51004	D00437
Interactive	hsa:51004	D00528
Interactive	hsa:51004	D00542
Interactive	hsa:51004	D00574
Interactive	hsa:51004	D01071
Interactive	hsa:51095	D00417
Interactive	hsa:51095	D00501
Interactive	hsa:51095	D00528
Interactive	hsa:51166	D00332
Interactive	hsa:51172	D00417
Interactive	hsa:51172	D00501
Interactive	hsa:51172	D00528
Interactive	hsa:51181	D00136
Interactive	hsa:51181	D00219
Interactive	hsa:51181	D00410
Interactive	hsa:51205	D00103
Interactive	hsa:51207	D00107
Interactive	hsa:51207	D00184
Interactive	hsa:5122	D00043
Interactive	hsa:5122	D00160
Interactive	hsa:51251	D00501
Interactive	hsa:5126	D00043
Interactive	hsa:5126	D00160
Interactive	hsa:51292	D03798
Interactive	hsa:51302	D00139
Interactive	hsa:51302	D00225
Interactive	hsa:51302	D00380
Interactive	hsa:51302	D00394
Interactive	hsa:51302	D00410
Interactive	hsa:51302	D00437
Interactive	hsa:51302	D00528
Interactive	hsa:51302	D00542
Interactive	hsa:51302	D00574
Interactive	hsa:51302	D01071
Interactive	hsa:51365	D01223
Interactive	hsa:5136	D00227
Interactive	hsa:5136	D00231

Interactive	hsa:5136	D00371
Interactive	hsa:5136	D00417
Interactive	hsa:5136	D00501
Interactive	hsa:5136	D00528
Interactive	hsa:5136	D00691
Interactive	hsa:5136	D01133
Interactive	hsa:5136	D01198
Interactive	hsa:5136	D01690
Interactive	hsa:5136	D01704
Interactive	hsa:5136	D01712
Interactive	hsa:5136	D02008
Interactive	hsa:5136	D02017
Interactive	hsa:5136	D02042
Interactive	hsa:5136	D02229
Interactive	hsa:5136	D02655
Interactive	hsa:5136	D02731
Interactive	hsa:5137	D00227
Interactive	hsa:5137	D00231
Interactive	hsa:5137	D00371
Interactive	hsa:5137	D00417
Interactive	hsa:5137	D00501
Interactive	hsa:5137	D00528
Interactive	hsa:5137	D00691
Interactive	hsa:5137	D01133
Interactive	hsa:5137	D01198
Interactive	hsa:5137	D01690
Interactive	hsa:5137	D01704
Interactive	hsa:5137	D01712
Interactive	hsa:5137	D02008
Interactive	hsa:5137	D02017
Interactive	hsa:5137	D02042
Interactive	hsa:5137	D02229
Interactive	hsa:5137	D02655
Interactive	hsa:5137	D02731
Interactive	hsa:5138	D00227
Interactive	hsa:5138	D00231
Interactive	hsa:5138	D00371
Interactive	hsa:5138	D00417
Interactive	hsa:5138	D00501
Interactive	hsa:5138	D00528
Interactive	hsa:5138	D00691
Interactive	hsa:5138	D01133

Interactive	hsa:5138	D01198
Interactive	hsa:5138	D01690
Interactive	hsa:5138	D01704
Interactive	hsa:5138	D01712
Interactive	hsa:5138	D02008
Interactive	hsa:5138	D02017
Interactive	hsa:5138	D02042
Interactive	hsa:5138	D02229
Interactive	hsa:5138	D02655
Interactive	hsa:5138	D02731
Interactive	hsa:513	D00542
Interactive	hsa:513	D00543
Interactive	hsa:513	D00544
Interactive	hsa:513	D00545
Interactive	hsa:513	D00546
Interactive	hsa:513	D00547
Interactive	hsa:5140	D00227
Interactive	hsa:5140	D00231
Interactive	hsa:5140	D00371
Interactive	hsa:5140	D00417
Interactive	hsa:5140	D00501
Interactive	hsa:5140	D00528
Interactive	hsa:5140	D00691
Interactive	hsa:5140	D01133
Interactive	hsa:5140	D01198
Interactive	hsa:5140	D01690
Interactive	hsa:5140	D01704
Interactive	hsa:5140	D01712
Interactive	hsa:5140	D02008
Interactive	hsa:5140	D02017
Interactive	hsa:5140	D02042
Interactive	hsa:5140	D02229
Interactive	hsa:5140	D02655
Interactive	hsa:5140	D02731
Interactive	hsa:5141	D00227
Interactive	hsa:5141	D00231
Interactive	hsa:5141	D00371
Interactive	hsa:5141	D00417
Interactive	hsa:5141	D00501
Interactive	hsa:5141	D00528
Interactive	hsa:5141	D00691
Interactive	hsa:5141	D01133

Interactive	hsa:5141	D01198
Interactive	hsa:5141	D01690
Interactive	hsa:5141	D01704
Interactive	hsa:5141	D01712
Interactive	hsa:5141	D02008
Interactive	hsa:5141	D02017
Interactive	hsa:5141	D02042
Interactive	hsa:5141	D02229
Interactive	hsa:5141	D02655
Interactive	hsa:5141	D02731
Interactive	hsa:5142	D00227
Interactive	hsa:5142	D00231
Interactive	hsa:5142	D00371
Interactive	hsa:5142	D00417
Interactive	hsa:5142	D00501
Interactive	hsa:5142	D00528
Interactive	hsa:5142	D00691
Interactive	hsa:5142	D01133
Interactive	hsa:5142	D01198
Interactive	hsa:5142	D01690
Interactive	hsa:5142	D01704
Interactive	hsa:5142	D01712
Interactive	hsa:5142	D02008
Interactive	hsa:5142	D02017
Interactive	hsa:5142	D02042
Interactive	hsa:5142	D02229
Interactive	hsa:5142	D02655
Interactive	hsa:5142	D02731
Interactive	hsa:5143	D00227
Interactive	hsa:5143	D00231
Interactive	hsa:5143	D00371
Interactive	hsa:5143	D00417
Interactive	hsa:5143	D00501
Interactive	hsa:5143	D00528
Interactive	hsa:5143	D00691
Interactive	hsa:5143	D01133
Interactive	hsa:5143	D01198
Interactive	hsa:5143	D01690
Interactive	hsa:5143	D01704
Interactive	hsa:5143	D01712
Interactive	hsa:5143	D02008
Interactive	hsa:5143	D02017

Interactive	hsa:5143	D02042
Interactive	hsa:5143	D02229
Interactive	hsa:5143	D02655
Interactive	hsa:5143	D02731
Interactive	hsa:5144	D00227
Interactive	hsa:5144	D00231
Interactive	hsa:5144	D00371
Interactive	hsa:5144	D00417
Interactive	hsa:5144	D00501
Interactive	hsa:5144	D00528
Interactive	hsa:5144	D00691
Interactive	hsa:5144	D01133
Interactive	hsa:5144	D01198
Interactive	hsa:5144	D01690
Interactive	hsa:5144	D01704
Interactive	hsa:5144	D01712
Interactive	hsa:5144	D02008
Interactive	hsa:5144	D02017
Interactive	hsa:5144	D02042
Interactive	hsa:5144	D02229
Interactive	hsa:5144	D02655
Interactive	hsa:5144	D02731
Interactive	hsa:5145	D00417
Interactive	hsa:5145	D00501
Interactive	hsa:5145	D00528
Interactive	hsa:5146	D00417
Interactive	hsa:5146	D00501
Interactive	hsa:5146	D00528
Interactive	hsa:5147	D00227
Interactive	hsa:5147	D00231
Interactive	hsa:5147	D00371
Interactive	hsa:5147	D00417
Interactive	hsa:5147	D00501
Interactive	hsa:5147	D00528
Interactive	hsa:5147	D00691
Interactive	hsa:5147	D01133
Interactive	hsa:5147	D01198
Interactive	hsa:5147	D01690
Interactive	hsa:5147	D01704
Interactive	hsa:5147	D01712
Interactive	hsa:5147	D02008
Interactive	hsa:5147	D02017

Interactive	hsa:5147	D02042
Interactive	hsa:5147	D02229
Interactive	hsa:5147	D02655
Interactive	hsa:5147	D02731
Interactive	hsa:5148	D00227
Interactive	hsa:5148	D00231
Interactive	hsa:5148	D00371
Interactive	hsa:5148	D00417
Interactive	hsa:5148	D00501
Interactive	hsa:5148	D00528
Interactive	hsa:5148	D00691
Interactive	hsa:5148	D01133
Interactive	hsa:5148	D01198
Interactive	hsa:5148	D01690
Interactive	hsa:5148	D01704
Interactive	hsa:5148	D01712
Interactive	hsa:5148	D02008
Interactive	hsa:5148	D02017
Interactive	hsa:5148	D02042
Interactive	hsa:5148	D02229
Interactive	hsa:5148	D02655
Interactive	hsa:5148	D02731
Interactive	hsa:5149	D00227
Interactive	hsa:5149	D00231
Interactive	hsa:5149	D00371
Interactive	hsa:5149	D00417
Interactive	hsa:5149	D00501
Interactive	hsa:5149	D00528
Interactive	hsa:5149	D00691
Interactive	hsa:5149	D01133
Interactive	hsa:5149	D01198
Interactive	hsa:5149	D01690
Interactive	hsa:5149	D01704
Interactive	hsa:5149	D01712
Interactive	hsa:5149	D02008
Interactive	hsa:5149	D02017
Interactive	hsa:5149	D02042
Interactive	hsa:5149	D02229
Interactive	hsa:5149	D02655
Interactive	hsa:5149	D02731
Interactive	hsa:5150	D00227
Interactive	hsa:5150	D00231

Interactive	hsa:5150	D00371
Interactive	hsa:5150	D00417
Interactive	hsa:5150	D01133
Interactive	hsa:5150	D01198
Interactive	hsa:5150	D01332
Interactive	hsa:5150	D01690
Interactive	hsa:5150	D01704
Interactive	hsa:5150	D01712
Interactive	hsa:5150	D02008
Interactive	hsa:5150	D02017
Interactive	hsa:5150	D02042
Interactive	hsa:5150	D02229
Interactive	hsa:5150	D02655
Interactive	hsa:5150	D02731
Interactive	hsa:5151	D00227
Interactive	hsa:5151	D00231
Interactive	hsa:5151	D00371
Interactive	hsa:5151	D00417
Interactive	hsa:5151	D00501
Interactive	hsa:5151	D00528
Interactive	hsa:5151	D00691
Interactive	hsa:5151	D01133
Interactive	hsa:5151	D01198
Interactive	hsa:5151	D01690
Interactive	hsa:5151	D01704
Interactive	hsa:5151	D01712
Interactive	hsa:5151	D02008
Interactive	hsa:5151	D02017
Interactive	hsa:5151	D02042
Interactive	hsa:5151	D02229
Interactive	hsa:5151	D02655
Interactive	hsa:5151	D02731
Interactive	hsa:5152	D00227
Interactive	hsa:5152	D00231
Interactive	hsa:5152	D00371
Interactive	hsa:5152	D00417
Interactive	hsa:5152	D00501
Interactive	hsa:5152	D00528
Interactive	hsa:5152	D01133
Interactive	hsa:5152	D01198
Interactive	hsa:5152	D01690
Interactive	hsa:5152	D01704

Interactive	hsa:5152	D01712
Interactive	hsa:5152	D02008
Interactive	hsa:5152	D02017
Interactive	hsa:5152	D02042
Interactive	hsa:5152	D02229
Interactive	hsa:5152	D02655
Interactive	hsa:5152	D02731
Interactive	hsa:5156	D01441
Interactive	hsa:5156	D01977
Interactive	hsa:5156	D03218
Interactive	hsa:5156	D03350
Interactive	hsa:5156	D04024
Interactive	hsa:5156	D04025
Interactive	hsa:5158	D00417
Interactive	hsa:5158	D00501
Interactive	hsa:5158	D00528
Interactive	hsa:5159	D01441
Interactive	hsa:5159	D01977
Interactive	hsa:5159	D03218
Interactive	hsa:5159	D03350
Interactive	hsa:5159	D04024
Interactive	hsa:5159	D04025
Interactive	hsa:51645	D00107
Interactive	hsa:5167	D00043
Interactive	hsa:5167	D00417
Interactive	hsa:5167	D00423
Interactive	hsa:5167	D00501
Interactive	hsa:5167	D00528
Interactive	hsa:5168	D00417
Interactive	hsa:5168	D00501
Interactive	hsa:5168	D00528
Interactive	hsa:5169	D00417
Interactive	hsa:5169	D00501
Interactive	hsa:5169	D00528
Interactive	hsa:51727	D02368
Interactive	hsa:51	D00185
Interactive	hsa:51	D00293
Interactive	hsa:51	D00332
Interactive	hsa:51	D00530
Interactive	hsa:51	D00549
Interactive	hsa:5294	D03670
Interactive	hsa:52	D00103

Interactive	hsa:5319	D01223
Interactive	hsa:5320	D01223
Interactive	hsa:5321	D00324
Interactive	hsa:5321	D00325
Interactive	hsa:5321	D00328
Interactive	hsa:5321	D01223
Interactive	hsa:5321	D01367
Interactive	hsa:5321	D01825
Interactive	hsa:5321	D02289
Interactive	hsa:5322	D01223
Interactive	hsa:5327	D00043
Interactive	hsa:5327	D00160
Interactive	hsa:5328	D00043
Interactive	hsa:5328	D00160
Interactive	hsa:5328	D01136
Interactive	hsa:5330	D00417
Interactive	hsa:5330	D00501
Interactive	hsa:5330	D00528
Interactive	hsa:5331	D00417
Interactive	hsa:5331	D00501
Interactive	hsa:5331	D00528
Interactive	hsa:5332	D00417
Interactive	hsa:5332	D00501
Interactive	hsa:5332	D00528
Interactive	hsa:5333	D00417
Interactive	hsa:5333	D00501
Interactive	hsa:5333	D00528
Interactive	hsa:5335	D00417
Interactive	hsa:5335	D00501
Interactive	hsa:5335	D00528
Interactive	hsa:5336	D00417
Interactive	hsa:5336	D00501
Interactive	hsa:5336	D00528
Interactive	hsa:5337	D00417
Interactive	hsa:5337	D00501
Interactive	hsa:5337	D00528
Interactive	hsa:5338	D00417
Interactive	hsa:5338	D00501
Interactive	hsa:5338	D00528
Interactive	hsa:5340	D00043
Interactive	hsa:5340	D00160
Interactive	hsa:5340	D01136

Interactive	hsa:5351	D00018
Interactive	hsa:53938	D00107
Interactive	hsa:53	D00103
Interactive	hsa:5406	D01223
Interactive	hsa:5406	D01346
Interactive	hsa:5406	D04028
Interactive	hsa:5407	D01223
Interactive	hsa:5407	D04028
Interactive	hsa:5408	D01223
Interactive	hsa:5408	D04028
Interactive	hsa:5422	D01907
Interactive	hsa:5423	D00168
Interactive	hsa:5444	D00043
Interactive	hsa:5445	D00043
Interactive	hsa:5447	D00208
Interactive	hsa:5447	D00965
Interactive	hsa:54490	D01276
Interactive	hsa:54575	D01276
Interactive	hsa:54576	D01276
Interactive	hsa:54577	D01276
Interactive	hsa:54578	D01276
Interactive	hsa:54579	D01276
Interactive	hsa:54600	D01276
Interactive	hsa:54657	D01276
Interactive	hsa:54658	D01276
Interactive	hsa:54659	D01276
Interactive	hsa:54677	D02176
Interactive	hsa:5470	D00107
Interactive	hsa:5470	D00184
Interactive	hsa:5475	D00107
Interactive	hsa:5475	D00184
Interactive	hsa:5476	D00043
Interactive	hsa:5478	D00107
Interactive	hsa:5479	D00107
Interactive	hsa:5481	D00107
Interactive	hsa:54878	D00043
Interactive	hsa:5494	D00107
Interactive	hsa:5494	D00184
Interactive	hsa:5495	D00107
Interactive	hsa:5495	D00184
Interactive	hsa:5496	D00107
Interactive	hsa:5496	D00184

Interactive	hsa:5499	D00107
Interactive	hsa:5499	D00184
Interactive	hsa:54	D00103
Interactive	hsa:5500	D00107
Interactive	hsa:5500	D00184
Interactive	hsa:5501	D00107
Interactive	hsa:5501	D00184
Interactive	hsa:5515	D00107
Interactive	hsa:5515	D00184
Interactive	hsa:5516	D00107
Interactive	hsa:5516	D00184
Interactive	hsa:5523	D00107
Interactive	hsa:5523	D00184
Interactive	hsa:5530	D00107
Interactive	hsa:5530	D00184
Interactive	hsa:55312	D00050
Interactive	hsa:5531	D00107
Interactive	hsa:5531	D00184
Interactive	hsa:5532	D00107
Interactive	hsa:5532	D00184
Interactive	hsa:5533	D00107
Interactive	hsa:5533	D00184
Interactive	hsa:55359	D01441
Interactive	hsa:5536	D00107
Interactive	hsa:5536	D00184
Interactive	hsa:5537	D00107
Interactive	hsa:5537	D00184
Interactive	hsa:5538	D05341
Interactive	hsa:5547	D00043
Interactive	hsa:5550	D00043
Interactive	hsa:5550	D00160
Interactive	hsa:55512	D00417
Interactive	hsa:55512	D00501
Interactive	hsa:55512	D00528
Interactive	hsa:5562	D02769
Interactive	hsa:55775	D00417
Interactive	hsa:55775	D00501
Interactive	hsa:55775	D00528
Interactive	hsa:55811	D00002
Interactive	hsa:558	D01441
Interactive	hsa:558	D01977
Interactive	hsa:558	D03218

Interactive	hsa:558	D03350
Interactive	hsa:558	D04024
Interactive	hsa:558	D04025
Interactive	hsa:55902	D02769
Interactive	hsa:5594	D01840
Interactive	hsa:5594	D03115
Interactive	hsa:5594	D03736
Interactive	hsa:5595	D01840
Interactive	hsa:5595	D03115
Interactive	hsa:5595	D03736
Interactive	hsa:5596	D01840
Interactive	hsa:5596	D03115
Interactive	hsa:5596	D03736
Interactive	hsa:5597	D01840
Interactive	hsa:5597	D03115
Interactive	hsa:5597	D03736
Interactive	hsa:5598	D01840
Interactive	hsa:5598	D03115
Interactive	hsa:5598	D03736
Interactive	hsa:5599	D01840
Interactive	hsa:5599	D03115
Interactive	hsa:5599	D03736
Interactive	hsa:55	D00103
Interactive	hsa:5600	D01840
Interactive	hsa:5600	D03115
Interactive	hsa:5600	D03736
Interactive	hsa:5601	D01840
Interactive	hsa:5601	D03115
Interactive	hsa:5601	D03736
Interactive	hsa:5602	D01840
Interactive	hsa:5602	D03115
Interactive	hsa:5602	D03736
Interactive	hsa:5603	D01840
Interactive	hsa:5603	D03115
Interactive	hsa:5603	D03736
Interactive	hsa:5604	D01441
Interactive	hsa:5605	D01441
Interactive	hsa:5606	D01441
Interactive	hsa:5607	D01441
Interactive	hsa:5608	D01441
Interactive	hsa:5624	D02335
Interactive	hsa:5625	D00035

Interactive	hsa:5645	D00043
Interactive	hsa:5645	D00160
Interactive	hsa:5646	D00043
Interactive	hsa:5646	D00160
Interactive	hsa:5650	D00043
Interactive	hsa:5650	D00160
Interactive	hsa:5651	D00043
Interactive	hsa:5651	D00160
Interactive	hsa:5657	D00043
Interactive	hsa:5657	D00160
Interactive	hsa:56922	D00029
Interactive	hsa:57016	D00136
Interactive	hsa:57176	D00039
Interactive	hsa:5740	D00139
Interactive	hsa:5740	D00225
Interactive	hsa:5740	D00380
Interactive	hsa:5740	D00394
Interactive	hsa:5740	D00410
Interactive	hsa:5740	D00437
Interactive	hsa:5740	D00510
Interactive	hsa:5740	D00528
Interactive	hsa:5740	D00542
Interactive	hsa:5740	D00574
Interactive	hsa:5740	D01071
Interactive	hsa:5740	D03670
Interactive	hsa:5742	D00097
Interactive	hsa:5742	D00109
Interactive	hsa:5742	D00118
Interactive	hsa:5742	D00120
Interactive	hsa:5742	D00126
Interactive	hsa:5742	D00127
Interactive	hsa:5742	D00130
Interactive	hsa:5742	D00132
Interactive	hsa:5742	D00141
Interactive	hsa:5742	D00158
Interactive	hsa:5742	D00169
Interactive	hsa:5742	D00217
Interactive	hsa:5742	D00315
Interactive	hsa:5742	D00330
Interactive	hsa:5742	D00377
Interactive	hsa:5742	D00425
Interactive	hsa:5742	D00452

Interactive	hsa:5742	D00463
Interactive	hsa:5742	D00510
Interactive	hsa:5742	D00566
Interactive	hsa:5742	D00567
Interactive	hsa:5742	D00568
Interactive	hsa:5742	D00810
Interactive	hsa:5742	D00813
Interactive	hsa:5742	D00827
Interactive	hsa:5742	D00903
Interactive	hsa:5742	D00904
Interactive	hsa:5742	D00968
Interactive	hsa:5742	D00969
Interactive	hsa:5742	D00970
Interactive	hsa:5742	D01122
Interactive	hsa:5742	D01183
Interactive	hsa:5742	D01325
Interactive	hsa:5742	D01364
Interactive	hsa:5742	D01397
Interactive	hsa:5742	D01475
Interactive	hsa:5742	D01513
Interactive	hsa:5742	D01547
Interactive	hsa:5742	D01565
Interactive	hsa:5742	D01578
Interactive	hsa:5742	D01582
Interactive	hsa:5742	D01709
Interactive	hsa:5742	D01718
Interactive	hsa:5742	D01765
Interactive	hsa:5742	D01767
Interactive	hsa:5742	D01811
Interactive	hsa:5742	D01866
Interactive	hsa:5742	D01974
Interactive	hsa:5742	D02110
Interactive	hsa:5742	D02290
Interactive	hsa:5742	D02341
Interactive	hsa:5742	D02350
Interactive	hsa:5742	D02355
Interactive	hsa:5742	D02709
Interactive	hsa:5742	D03689
Interactive	hsa:5742	D03710
Interactive	hsa:5742	D03712
Interactive	hsa:5742	D03714
Interactive	hsa:5742	D03716

Interactive	hsa:5742	D03717
Interactive	hsa:5743	D00109
Interactive	hsa:5743	D00118
Interactive	hsa:5743	D00120
Interactive	hsa:5743	D00126
Interactive	hsa:5743	D00127
Interactive	hsa:5743	D00130
Interactive	hsa:5743	D00132
Interactive	hsa:5743	D00141
Interactive	hsa:5743	D00158
Interactive	hsa:5743	D00169
Interactive	hsa:5743	D00217
Interactive	hsa:5743	D00315
Interactive	hsa:5743	D00330
Interactive	hsa:5743	D00377
Interactive	hsa:5743	D00425
Interactive	hsa:5743	D00449
Interactive	hsa:5743	D00452
Interactive	hsa:5743	D00463
Interactive	hsa:5743	D00510
Interactive	hsa:5743	D00566
Interactive	hsa:5743	D00567
Interactive	hsa:5743	D00568
Interactive	hsa:5743	D00810
Interactive	hsa:5743	D00813
Interactive	hsa:5743	D00827
Interactive	hsa:5743	D00903
Interactive	hsa:5743	D00904
Interactive	hsa:5743	D00968
Interactive	hsa:5743	D00969
Interactive	hsa:5743	D00970
Interactive	hsa:5743	D01122
Interactive	hsa:5743	D01183
Interactive	hsa:5743	D01325
Interactive	hsa:5743	D01364
Interactive	hsa:5743	D01397
Interactive	hsa:5743	D01475
Interactive	hsa:5743	D01513
Interactive	hsa:5743	D01547
Interactive	hsa:5743	D01565
Interactive	hsa:5743	D01578
Interactive	hsa:5743	D01582

Interactive	hsa:5743	D01709
Interactive	hsa:5743	D01718
Interactive	hsa:5743	D01765
Interactive	hsa:5743	D01767
Interactive	hsa:5743	D01811
Interactive	hsa:5743	D01866
Interactive	hsa:5743	D01974
Interactive	hsa:5743	D02110
Interactive	hsa:5743	D02290
Interactive	hsa:5743	D02341
Interactive	hsa:5743	D02350
Interactive	hsa:5743	D02355
Interactive	hsa:5743	D02709
Interactive	hsa:5743	D03689
Interactive	hsa:5743	D03710
Interactive	hsa:5743	D03712
Interactive	hsa:5743	D03714
Interactive	hsa:5743	D03716
Interactive	hsa:5743	D03717
Interactive	hsa:5747	D01441
Interactive	hsa:5754	D01441
Interactive	hsa:5754	D01977
Interactive	hsa:5754	D03218
Interactive	hsa:5754	D03350
Interactive	hsa:5754	D04024
Interactive	hsa:5754	D04025
Interactive	hsa:57665	D00501
Interactive	hsa:58190	D00107
Interactive	hsa:58190	D00184
Interactive	hsa:5831	D00035
Interactive	hsa:5834	D02769
Interactive	hsa:5836	D02769
Interactive	hsa:5837	D02769
Interactive	hsa:5860	D00002
Interactive	hsa:586	D00039
Interactive	hsa:586	D00065
Interactive	hsa:586	D00332
Interactive	hsa:587	D00065
Interactive	hsa:587	D00332
Interactive	hsa:590	D00043
Interactive	hsa:590	D00196
Interactive	hsa:590	D00667

Interactive	hsa:590	D00670
Interactive	hsa:590	D00733
Interactive	hsa:590	D00805
Interactive	hsa:590	D01118
Interactive	hsa:590	D01223
Interactive	hsa:590	D02193
Interactive	hsa:590	D02729
Interactive	hsa:590	D03822
Interactive	hsa:5972	D03738
Interactive	hsa:5972	D03741
Interactive	hsa:5972	D03743
Interactive	hsa:5979	D01441
Interactive	hsa:5979	D01977
Interactive	hsa:5979	D03218
Interactive	hsa:5979	D03350
Interactive	hsa:5979	D04024
Interactive	hsa:5979	D04025
Interactive	hsa:6098	D01441
Interactive	hsa:6098	D01977
Interactive	hsa:6098	D03218
Interactive	hsa:6098	D03350
Interactive	hsa:6098	D04024
Interactive	hsa:6098	D04025
Interactive	hsa:613	D01441
Interactive	hsa:6240	D01907
Interactive	hsa:6240	D02368
Interactive	hsa:6240	D03670
Interactive	hsa:6241	D03670
Interactive	hsa:6259	D01441
Interactive	hsa:6259	D01977
Interactive	hsa:6259	D03218
Interactive	hsa:6259	D03350
Interactive	hsa:6259	D04024
Interactive	hsa:6259	D04025
Interactive	hsa:6300	D01840
Interactive	hsa:6300	D03115
Interactive	hsa:6300	D03736
Interactive	hsa:63036	D00043
Interactive	hsa:63036	D00160
Interactive	hsa:635	D00037
Interactive	hsa:63904	D00107
Interactive	hsa:63904	D00184

Interactive	hsa:64087	D00029
Interactive	hsa:6416	D01441
Interactive	hsa:64499	D00043
Interactive	hsa:64499	D00160
Interactive	hsa:645	D00050
Interactive	hsa:64600	D01223
Interactive	hsa:64802	D00002
Interactive	hsa:64816	D00139
Interactive	hsa:64816	D00225
Interactive	hsa:64816	D00380
Interactive	hsa:64816	D00394
Interactive	hsa:64816	D00410
Interactive	hsa:64816	D00437
Interactive	hsa:64816	D00528
Interactive	hsa:64816	D00542
Interactive	hsa:64816	D00574
Interactive	hsa:64816	D00960
Interactive	hsa:64816	D00964
Interactive	hsa:64816	D01071
Interactive	hsa:64816	D01425
Interactive	hsa:64816	D02451
Interactive	hsa:64816	D03670
Interactive	hsa:64816	D03778
Interactive	hsa:64816	D03781
Interactive	hsa:64816	D03784
Interactive	hsa:64850	D00332
Interactive	hsa:64902	D00332
Interactive	hsa:657	D01441
Interactive	hsa:658	D01441
Interactive	hsa:6609	D00417
Interactive	hsa:6609	D00501
Interactive	hsa:6609	D00528
Interactive	hsa:660	D01441
Interactive	hsa:6610	D00417
Interactive	hsa:6610	D00501
Interactive	hsa:6610	D00528
Interactive	hsa:6646	D01966
Interactive	hsa:6646	D03012
Interactive	hsa:6646	D03734
Interactive	hsa:6646	D03735
Interactive	hsa:6652	D00002
Interactive	hsa:670	D01223

Interactive	hsa:6713	D02375
Interactive	hsa:6714	D01441
Interactive	hsa:6716	D03034
Interactive	hsa:6725	D01441
Interactive	hsa:6768	D00043
Interactive	hsa:6768	D00160
Interactive	hsa:6799	D00217
Interactive	hsa:6799	D00418
Interactive	hsa:683	D00036
Interactive	hsa:686	D00029
Interactive	hsa:6897	D00041
Interactive	hsa:6898	D00021
Interactive	hsa:6898	D00332
Interactive	hsa:695	D01441
Interactive	hsa:7006	D01441
Interactive	hsa:7010	D01441
Interactive	hsa:7010	D01977
Interactive	hsa:7010	D03218
Interactive	hsa:7010	D03350
Interactive	hsa:7010	D04024
Interactive	hsa:7010	D04025
Interactive	hsa:7015	D00579
Interactive	hsa:7015	D02267
Interactive	hsa:7046	D01441
Interactive	hsa:7054	D00021
Interactive	hsa:7054	D00762
Interactive	hsa:7075	D01441
Interactive	hsa:7075	D01977
Interactive	hsa:7075	D03218
Interactive	hsa:7075	D03350
Interactive	hsa:7075	D04024
Interactive	hsa:7075	D04025
Interactive	hsa:7083	D00584
Interactive	hsa:7084	D00584
Interactive	hsa:7150	D01061
Interactive	hsa:7150	D01432
Interactive	hsa:7150	D01911
Interactive	hsa:7150	D02168
Interactive	hsa:7150	D02756
Interactive	hsa:7150	D04031
Interactive	hsa:7153	D00125
Interactive	hsa:7153	D00183

Interactive	hsa:7153	D00186
Interactive	hsa:7153	D01264
Interactive	hsa:7153	D01275
Interactive	hsa:7153	D01885
Interactive	hsa:7153	D01911
Interactive	hsa:7153	D02166
Interactive	hsa:7153	D02214
Interactive	hsa:7153	D02321
Interactive	hsa:7153	D02333
Interactive	hsa:7153	D02698
Interactive	hsa:7153	D02756
Interactive	hsa:7153	D03899
Interactive	hsa:7155	D00125
Interactive	hsa:7155	D00183
Interactive	hsa:7155	D00186
Interactive	hsa:7155	D01264
Interactive	hsa:7155	D01275
Interactive	hsa:7155	D01885
Interactive	hsa:7155	D01911
Interactive	hsa:7155	D02166
Interactive	hsa:7155	D02214
Interactive	hsa:7155	D02321
Interactive	hsa:7155	D02333
Interactive	hsa:7155	D02698
Interactive	hsa:7155	D02756
Interactive	hsa:7155	D03899
Interactive	hsa:7156	D01061
Interactive	hsa:7156	D01432
Interactive	hsa:7156	D01911
Interactive	hsa:7156	D02168
Interactive	hsa:7156	D02756
Interactive	hsa:7156	D04031
Interactive	hsa:7172	D00377
Interactive	hsa:7173	D00401
Interactive	hsa:7173	D00562
Interactive	hsa:7174	D00043
Interactive	hsa:7174	D00160
Interactive	hsa:7294	D01441
Interactive	hsa:7297	D01441
Interactive	hsa:7298	D00584
Interactive	hsa:7298	D01064
Interactive	hsa:7298	D01211

Interactive	hsa:7298	D01223
Interactive	hsa:7298	D02368
Interactive	hsa:7298	D03828
Interactive	hsa:7298	D04197
Interactive	hsa:7299	D03034
Interactive	hsa:7301	D01441
Interactive	hsa:7301	D01977
Interactive	hsa:7301	D03218
Interactive	hsa:7301	D03350
Interactive	hsa:7301	D04024
Interactive	hsa:7301	D04025
Interactive	hsa:7363	D01276
Interactive	hsa:7364	D01276
Interactive	hsa:7365	D01276
Interactive	hsa:7366	D01276
Interactive	hsa:7367	D01276
Interactive	hsa:7371	D00037
Interactive	hsa:7372	D00584
Interactive	hsa:7378	D00584
Interactive	hsa:7453	D00401
Interactive	hsa:7453	D00562
Interactive	hsa:7498	D00005
Interactive	hsa:7498	D00224
Interactive	hsa:7525	D01441
Interactive	hsa:7535	D01441
Interactive	hsa:759	D00218
Interactive	hsa:759	D00294
Interactive	hsa:759	D00340
Interactive	hsa:759	D00518
Interactive	hsa:759	D00519
Interactive	hsa:759	D00538
Interactive	hsa:759	D00650
Interactive	hsa:759	D00651
Interactive	hsa:759	D00652
Interactive	hsa:759	D00653
Interactive	hsa:759	D00654
Interactive	hsa:759	D00655
Interactive	hsa:759	D00656
Interactive	hsa:759	D00658
Interactive	hsa:759	D00703
Interactive	hsa:759	D00709
Interactive	hsa:759	D01196

Interactive	hsa:759	D01256
Interactive	hsa:759	D02356
Interactive	hsa:759	D02441
Interactive	hsa:760	D00218
Interactive	hsa:760	D00294
Interactive	hsa:760	D00340
Interactive	hsa:760	D00518
Interactive	hsa:760	D00519
Interactive	hsa:760	D00537
Interactive	hsa:760	D00538
Interactive	hsa:760	D00650
Interactive	hsa:760	D00651
Interactive	hsa:760	D00652
Interactive	hsa:760	D00653
Interactive	hsa:760	D00654
Interactive	hsa:760	D00655
Interactive	hsa:760	D00656
Interactive	hsa:760	D00658
Interactive	hsa:760	D00703
Interactive	hsa:760	D01196
Interactive	hsa:760	D01256
Interactive	hsa:761	D00218
Interactive	hsa:761	D00340
Interactive	hsa:761	D00518
Interactive	hsa:761	D00519
Interactive	hsa:761	D00538
Interactive	hsa:761	D00652
Interactive	hsa:761	D00653
Interactive	hsa:761	D00655
Interactive	hsa:761	D01196
Interactive	hsa:762	D00218
Interactive	hsa:762	D00294
Interactive	hsa:762	D00340
Interactive	hsa:762	D00518
Interactive	hsa:762	D00519
Interactive	hsa:762	D00537
Interactive	hsa:762	D00538
Interactive	hsa:762	D00650
Interactive	hsa:762	D00651
Interactive	hsa:762	D00652
Interactive	hsa:762	D00653
Interactive	hsa:762	D00654

Interactive	hsa:762	D00655
Interactive	hsa:762	D00656
Interactive	hsa:762	D00658
Interactive	hsa:762	D01196
Interactive	hsa:762	D01256
Interactive	hsa:763	D00218
Interactive	hsa:763	D00340
Interactive	hsa:763	D00518
Interactive	hsa:763	D00519
Interactive	hsa:763	D00538
Interactive	hsa:763	D00652
Interactive	hsa:763	D00653
Interactive	hsa:763	D00655
Interactive	hsa:763	D01196
Interactive	hsa:765	D00218
Interactive	hsa:765	D00340
Interactive	hsa:765	D00518
Interactive	hsa:765	D00519
Interactive	hsa:765	D00538
Interactive	hsa:765	D00652
Interactive	hsa:765	D00653
Interactive	hsa:765	D00655
Interactive	hsa:765	D01196
Interactive	hsa:766	D00218
Interactive	hsa:766	D00340
Interactive	hsa:766	D00518
Interactive	hsa:766	D00519
Interactive	hsa:766	D00538
Interactive	hsa:766	D00652
Interactive	hsa:766	D00653
Interactive	hsa:766	D00655
Interactive	hsa:766	D01196
Interactive	hsa:767	D00218
Interactive	hsa:767	D00340
Interactive	hsa:767	D00518
Interactive	hsa:767	D00519
Interactive	hsa:767	D00538
Interactive	hsa:767	D00652
Interactive	hsa:767	D00653
Interactive	hsa:767	D00655
Interactive	hsa:767	D01196
Interactive	hsa:768	D00218

Interactive	hsa:768	D00340
Interactive	hsa:768	D00518
Interactive	hsa:768	D00519
Interactive	hsa:768	D00538
Interactive	hsa:768	D00652
Interactive	hsa:768	D00653
Interactive	hsa:768	D00655
Interactive	hsa:768	D01196
Interactive	hsa:771	D00218
Interactive	hsa:771	D00340
Interactive	hsa:771	D00518
Interactive	hsa:771	D00519
Interactive	hsa:771	D00538
Interactive	hsa:771	D00652
Interactive	hsa:771	D00653
Interactive	hsa:771	D00655
Interactive	hsa:771	D01196
Interactive	hsa:780	D01441
Interactive	hsa:780	D01977
Interactive	hsa:780	D03218
Interactive	hsa:780	D03350
Interactive	hsa:780	D04024
Interactive	hsa:780	D04025
Interactive	hsa:79001	D02335
Interactive	hsa:79001	D03798
Interactive	hsa:7957	D00107
Interactive	hsa:7957	D00184
Interactive	hsa:79799	D01276
Interactive	hsa:80339	D01223
Interactive	hsa:80339	D04028
Interactive	hsa:80824	D00107
Interactive	hsa:80824	D00184
Interactive	hsa:81579	D01223
Interactive	hsa:8192	D00043
Interactive	hsa:8192	D00160
Interactive	hsa:8288	D00097
Interactive	hsa:8288	D00217
Interactive	hsa:8288	D00562
Interactive	hsa:8288	D00577
Interactive	hsa:8288	D03670
Interactive	hsa:834	D00496
Interactive	hsa:8398	D01223

Interactive	hsa:8399	D01223
Interactive	hsa:84152	D00107
Interactive	hsa:84152	D00184
Interactive	hsa:84171	D00270
Interactive	hsa:8435	D01966
Interactive	hsa:8435	D03012
Interactive	hsa:8435	D03734
Interactive	hsa:8435	D03735
Interactive	hsa:84532	D02769
Interactive	hsa:84618	D00501
Interactive	hsa:84695	D00270
Interactive	hsa:84706	D00332
Interactive	hsa:84812	D00417
Interactive	hsa:84812	D00501
Interactive	hsa:84812	D00528
Interactive	hsa:8513	D01223
Interactive	hsa:8513	D04028
Interactive	hsa:8529	D00139
Interactive	hsa:8529	D00225
Interactive	hsa:8529	D00380
Interactive	hsa:8529	D00394
Interactive	hsa:8529	D00410
Interactive	hsa:8529	D00437
Interactive	hsa:8529	D00528
Interactive	hsa:8529	D00542
Interactive	hsa:8529	D00574
Interactive	hsa:8529	D01071
Interactive	hsa:8529	D03670
Interactive	hsa:85313	D00107
Interactive	hsa:8555	D00107
Interactive	hsa:8555	D00184
Interactive	hsa:8556	D00107
Interactive	hsa:8556	D00184
Interactive	hsa:8622	D00227
Interactive	hsa:8622	D00231
Interactive	hsa:8622	D00371
Interactive	hsa:8622	D00417
Interactive	hsa:8622	D00501
Interactive	hsa:8622	D00528
Interactive	hsa:8622	D00691
Interactive	hsa:8622	D01133
Interactive	hsa:8622	D01198

Interactive	hsa:8622	D01690
Interactive	hsa:8622	D01704
Interactive	hsa:8622	D01712
Interactive	hsa:8622	D02008
Interactive	hsa:8622	D02017
Interactive	hsa:8622	D02042
Interactive	hsa:8622	D02229
Interactive	hsa:8622	D02655
Interactive	hsa:8622	D02731
Interactive	hsa:8654	D00037
Interactive	hsa:8654	D00227
Interactive	hsa:8654	D00231
Interactive	hsa:8654	D00371
Interactive	hsa:8654	D00417
Interactive	hsa:8654	D00501
Interactive	hsa:8654	D00528
Interactive	hsa:8654	D01133
Interactive	hsa:8654	D01198
Interactive	hsa:8654	D01690
Interactive	hsa:8654	D01704
Interactive	hsa:8654	D01712
Interactive	hsa:8654	D02008
Interactive	hsa:8654	D02017
Interactive	hsa:8654	D02042
Interactive	hsa:8654	D02229
Interactive	hsa:8654	D02655
Interactive	hsa:8654	D02731
Interactive	hsa:8836	D00070
Interactive	hsa:8854	D00094
Interactive	hsa:8940	D01061
Interactive	hsa:8940	D01432
Interactive	hsa:8940	D01911
Interactive	hsa:8940	D02168
Interactive	hsa:8940	D02756
Interactive	hsa:8940	D04031
Interactive	hsa:8972	D00216
Interactive	hsa:8972	D00625
Interactive	hsa:8972	D01665
Interactive	hsa:8972	D03433
Interactive	hsa:9023	D00139
Interactive	hsa:9023	D00225
Interactive	hsa:9023	D00380

Interactive	hsa:9023	D00394
Interactive	hsa:9023	D00410
Interactive	hsa:9023	D00437
Interactive	hsa:9023	D00528
Interactive	hsa:9023	D00542
Interactive	hsa:9023	D00574
Interactive	hsa:9023	D01071
Interactive	hsa:9023	D03670
Interactive	hsa:9088	D01441
Interactive	hsa:90	D01441
Interactive	hsa:91039	D00043
Interactive	hsa:9150	D00107
Interactive	hsa:9150	D00184
Interactive	hsa:91	D01441
Interactive	hsa:93650	D00103
Interactive	hsa:9388	D01223
Interactive	hsa:9388	D04028
Interactive	hsa:93	D01441
Interactive	hsa:94009	D00043
Interactive	hsa:94009	D00160
Interactive	hsa:9420	D00139
Interactive	hsa:9420	D00225
Interactive	hsa:9420	D00380
Interactive	hsa:9420	D00394
Interactive	hsa:9420	D00410
Interactive	hsa:9420	D00437
Interactive	hsa:9420	D00528
Interactive	hsa:9420	D00542
Interactive	hsa:9420	D00574
Interactive	hsa:9420	D01071
Interactive	hsa:94	D01441
Interactive	hsa:9563	D01223
Interactive	hsa:9601	D03798
Interactive	hsa:9641	D00448
Interactive	hsa:9647	D00107
Interactive	hsa:9647	D00184
Interactive	hsa:9945	D00332
Interactive	hsa:9955	D00037
Non-interactive	hsa:10056	D00503
Non-interactive	hsa:10056	D00650
Non-interactive	hsa:10056	D00733

Non-interactive	hsa:10056	D01825
Non-interactive	hsa:10056	D02451
Non-interactive	hsa:100	D00005
Non-interactive	hsa:100	D00283
Non-interactive	hsa:100	D00753
Non-interactive	hsa:100	D03765
Non-interactive	hsa:10188	D00127
Non-interactive	hsa:10188	D00216
Non-interactive	hsa:10188	D00650
Non-interactive	hsa:10188	D01240
Non-interactive	hsa:10188	D03012
Non-interactive	hsa:10188	D03717
Non-interactive	hsa:10269	D01984
Non-interactive	hsa:10279	D00394
Non-interactive	hsa:10279	D00488
Non-interactive	hsa:10279	D01578
Non-interactive	hsa:10279	D02168
Non-interactive	hsa:10279	D02560
Non-interactive	hsa:10279	D03775
Non-interactive	hsa:10295	D00771
Non-interactive	hsa:10295	D02194
Non-interactive	hsa:10295	D02258
Non-interactive	hsa:10327	D00002
Non-interactive	hsa:10327	D00070
Non-interactive	hsa:10327	D00139
Non-interactive	hsa:10327	D00423
Non-interactive	hsa:10327	D00656
Non-interactive	hsa:10327	D01256
Non-interactive	hsa:10327	D01907
Non-interactive	hsa:10327	D01973
Non-interactive	hsa:10327	D02441
Non-interactive	hsa:1033	D00196
Non-interactive	hsa:1033	D00654
Non-interactive	hsa:1033	D00893
Non-interactive	hsa:1033	D01061
Non-interactive	hsa:1033	D01825
Non-interactive	hsa:1033	D02166
Non-interactive	hsa:1033	D02451
Non-interactive	hsa:1033	D02671
Non-interactive	hsa:1033	D03738
Non-interactive	hsa:10461	D00622
Non-interactive	hsa:10461	D00630

Non-interactive	hsa:10461	D00965
Non-interactive	hsa:10461	D02328
Non-interactive	hsa:10461	D03643
Non-interactive	hsa:10461	D03778
Non-interactive	hsa:10549	D01332
Non-interactive	hsa:10549	D02418
Non-interactive	hsa:1056	D00315
Non-interactive	hsa:1056	D01974
Non-interactive	hsa:1056	D02115
Non-interactive	hsa:10667	D00018
Non-interactive	hsa:10667	D00094
Non-interactive	hsa:10667	D00398
Non-interactive	hsa:10667	D00651
Non-interactive	hsa:10667	D00965
Non-interactive	hsa:10667	D01718
Non-interactive	hsa:10667	D01866
Non-interactive	hsa:10667	D02487
Non-interactive	hsa:10720	D00107
Non-interactive	hsa:10720	D00620
Non-interactive	hsa:10720	D00947
Non-interactive	hsa:10720	D02214
Non-interactive	hsa:10720	D03743
Non-interactive	hsa:10747	D00965
Non-interactive	hsa:10747	D02671
Non-interactive	hsa:10747	D03218
Non-interactive	hsa:10747	D03805
Non-interactive	hsa:107	D00596
Non-interactive	hsa:107	D00969
Non-interactive	hsa:107	D01211
Non-interactive	hsa:107	D02258
Non-interactive	hsa:107	D02580
Non-interactive	hsa:10825	D00391
Non-interactive	hsa:10825	D00516
Non-interactive	hsa:10825	D00623
Non-interactive	hsa:10825	D02168
Non-interactive	hsa:10825	D03077
Non-interactive	hsa:10846	D00448
Non-interactive	hsa:10846	D01844
Non-interactive	hsa:10846	D02166
Non-interactive	hsa:10846	D02580
Non-interactive	hsa:10846	D03803
Non-interactive	hsa:10858	D00131

Non-interactive	hsa:10858	D00533
Non-interactive	hsa:10858	D02368
Non-interactive	hsa:10858	D02655
Non-interactive	hsa:10858	D02731
Non-interactive	hsa:10858	D03077
Non-interactive	hsa:10858	D03720
Non-interactive	hsa:108	D00537
Non-interactive	hsa:108	D03643
Non-interactive	hsa:108	D03778
Non-interactive	hsa:10901	D00148
Non-interactive	hsa:10901	D00622
Non-interactive	hsa:10901	D01061
Non-interactive	hsa:10901	D01915
Non-interactive	hsa:10901	D03710
Non-interactive	hsa:10901	D03735
Non-interactive	hsa:10901	D03743
Non-interactive	hsa:10901	D04292
Non-interactive	hsa:10924	D00005
Non-interactive	hsa:10924	D01027
Non-interactive	hsa:10924	D01441
Non-interactive	hsa:10924	D02418
Non-interactive	hsa:10924	D02769
Non-interactive	hsa:10935	D00127
Non-interactive	hsa:10935	D00187
Non-interactive	hsa:10935	D00902
Non-interactive	hsa:10935	D02173
Non-interactive	hsa:10935	D02194
Non-interactive	hsa:10941	D00142
Non-interactive	hsa:10941	D00620
Non-interactive	hsa:10941	D00947
Non-interactive	hsa:10941	D02655
Non-interactive	hsa:109	D00188
Non-interactive	hsa:109	D00437
Non-interactive	hsa:109	D00805
Non-interactive	hsa:109	D01370
Non-interactive	hsa:109	D02115
Non-interactive	hsa:109	D02731
Non-interactive	hsa:109	D03899
Non-interactive	hsa:10	D01180
Non-interactive	hsa:10	D01367
Non-interactive	hsa:10	D01844
Non-interactive	hsa:10	D02173

Non-interactive	hsa:10	D03643
Non-interactive	hsa:11072	D00120
Non-interactive	hsa:11072	D00252
Non-interactive	hsa:11072	D00519
Non-interactive	hsa:11072	D00658
Non-interactive	hsa:11072	D02375
Non-interactive	hsa:11072	D02756
Non-interactive	hsa:11072	D03798
Non-interactive	hsa:111	D00125
Non-interactive	hsa:111	D00187
Non-interactive	hsa:111	D01441
Non-interactive	hsa:111	D02333
Non-interactive	hsa:111	D03798
Non-interactive	hsa:11202	D00315
Non-interactive	hsa:11202	D00515
Non-interactive	hsa:11202	D00653
Non-interactive	hsa:11202	D02418
Non-interactive	hsa:11221	D00208
Non-interactive	hsa:11221	D00519
Non-interactive	hsa:11221	D02110
Non-interactive	hsa:11221	D03716
Non-interactive	hsa:11238	D00107
Non-interactive	hsa:11238	D00283
Non-interactive	hsa:11238	D01027
Non-interactive	hsa:11266	D00516
Non-interactive	hsa:11266	D02328
Non-interactive	hsa:11266	D03710
Non-interactive	hsa:112	D00141
Non-interactive	hsa:112	D00884
Non-interactive	hsa:112	D04029
Non-interactive	hsa:112	D04031
Non-interactive	hsa:11330	D00225
Non-interactive	hsa:11343	D00218
Non-interactive	hsa:11343	D00813
Non-interactive	hsa:11343	D00965
Non-interactive	hsa:11343	D01064
Non-interactive	hsa:11343	D03778
Non-interactive	hsa:113	D00285
Non-interactive	hsa:113	D00294
Non-interactive	hsa:113	D00325
Non-interactive	hsa:113	D00655
Non-interactive	hsa:113	D01211

Non-interactive	hsa:113	D01973
Non-interactive	hsa:1147	D00196
Non-interactive	hsa:1147	D00423
Non-interactive	hsa:1147	D00892
Non-interactive	hsa:1147	D01276
Non-interactive	hsa:1147	D01432
Non-interactive	hsa:1147	D02229
Non-interactive	hsa:1147	D02580
Non-interactive	hsa:115	D00120
Non-interactive	hsa:115	D03735
Non-interactive	hsa:116447	D00018
Non-interactive	hsa:116447	D01578
Non-interactive	hsa:116447	D01915
Non-interactive	hsa:116447	D02709
Non-interactive	hsa:1178	D00107
Non-interactive	hsa:1178	D00127
Non-interactive	hsa:1178	D00155
Non-interactive	hsa:1178	D00394
Non-interactive	hsa:1178	D01256
Non-interactive	hsa:1200	D00127
Non-interactive	hsa:1200	D00285
Non-interactive	hsa:1200	D00463
Non-interactive	hsa:1200	D01367
Non-interactive	hsa:1200	D02368
Non-interactive	hsa:1200	D04025
Non-interactive	hsa:1215	D00188
Non-interactive	hsa:1215	D00455
Non-interactive	hsa:1215	D00538
Non-interactive	hsa:1215	D01332
Non-interactive	hsa:1215	D02110
Non-interactive	hsa:124	D00283
Non-interactive	hsa:124	D00596
Non-interactive	hsa:124	D00885
Non-interactive	hsa:124	D01240
Non-interactive	hsa:124	D02441
Non-interactive	hsa:125	D00294
Non-interactive	hsa:125	D00455
Non-interactive	hsa:125	D00537
Non-interactive	hsa:1267	D00218
Non-interactive	hsa:1267	D00892
Non-interactive	hsa:1267	D00969
Non-interactive	hsa:126	D00142

Non-interactive	hsa:126	D00885
Non-interactive	hsa:126	D01332
Non-interactive	hsa:126	D02168
Non-interactive	hsa:126	D03828
Non-interactive	hsa:128853	D00148
Non-interactive	hsa:128853	D00771
Non-interactive	hsa:128853	D01907
Non-interactive	hsa:128	D00094
Non-interactive	hsa:128	D00969
Non-interactive	hsa:128	D03728
Non-interactive	hsa:129807	D00142
Non-interactive	hsa:129807	D01119
Non-interactive	hsa:130399	D00005
Non-interactive	hsa:130399	D00530
Non-interactive	hsa:130399	D00651
Non-interactive	hsa:130399	D00658
Non-interactive	hsa:130399	D01240
Non-interactive	hsa:130399	D01256
Non-interactive	hsa:130399	D04025
Non-interactive	hsa:1312	D01582
Non-interactive	hsa:1312	D04029
Non-interactive	hsa:131	D03716
Non-interactive	hsa:132160	D00070
Non-interactive	hsa:132160	D00315
Non-interactive	hsa:132160	D03218
Non-interactive	hsa:132	D00107
Non-interactive	hsa:132	D00414
Non-interactive	hsa:132	D00887
Non-interactive	hsa:132	D01275
Non-interactive	hsa:132	D03741
Non-interactive	hsa:133121	D00125
Non-interactive	hsa:134510	D00423
Non-interactive	hsa:134510	D00753
Non-interactive	hsa:134510	D03805
Non-interactive	hsa:1360	D00203
Non-interactive	hsa:1360	D00414
Non-interactive	hsa:1360	D00423
Non-interactive	hsa:1360	D00563
Non-interactive	hsa:1360	D00887
Non-interactive	hsa:1360	D01690
Non-interactive	hsa:1360	D02110
Non-interactive	hsa:1360	D03816

Non-interactive	hsa:1363	D00142
Non-interactive	hsa:1363	D00885
Non-interactive	hsa:1363	D00947
Non-interactive	hsa:1363	D02168
Non-interactive	hsa:1363	D02487
Non-interactive	hsa:1363	D03738
Non-interactive	hsa:1374	D00900
Non-interactive	hsa:1374	D01211
Non-interactive	hsa:1376	D00530
Non-interactive	hsa:1376	D01441
Non-interactive	hsa:1376	D02756
Non-interactive	hsa:1376	D03712
Non-interactive	hsa:1384	D00364
Non-interactive	hsa:1384	D01918
Non-interactive	hsa:1384	D03736
Non-interactive	hsa:1384	D03798
Non-interactive	hsa:13	D00805
Non-interactive	hsa:13	D01718
Non-interactive	hsa:13	D01765
Non-interactive	hsa:13	D02214
Non-interactive	hsa:13	D02368
Non-interactive	hsa:13	D03350
Non-interactive	hsa:142679	D00094
Non-interactive	hsa:142679	D00622
Non-interactive	hsa:142679	D00752
Non-interactive	hsa:142679	D01240
Non-interactive	hsa:142679	D01690
Non-interactive	hsa:142679	D01977
Non-interactive	hsa:142679	D03720
Non-interactive	hsa:1429	D00364
Non-interactive	hsa:1429	D00398
Non-interactive	hsa:1429	D02375
Non-interactive	hsa:1431	D00218
Non-interactive	hsa:1431	D00325
Non-interactive	hsa:1431	D01133
Non-interactive	hsa:1431	D01974
Non-interactive	hsa:1431	D02194
Non-interactive	hsa:1431	D02368
Non-interactive	hsa:1432	D00203
Non-interactive	hsa:1432	D00359
Non-interactive	hsa:1432	D00387
Non-interactive	hsa:1432	D00414

Non-interactive	hsa:1432	D00781
Non-interactive	hsa:1436	D00963
Non-interactive	hsa:1436	D02655
Non-interactive	hsa:1445	D00142
Non-interactive	hsa:1445	D00216
Non-interactive	hsa:1445	D00658
Non-interactive	hsa:1445	D00805
Non-interactive	hsa:1445	D00887
Non-interactive	hsa:1445	D01256
Non-interactive	hsa:1445	D02333
Non-interactive	hsa:1445	D02375
Non-interactive	hsa:1445	D03767
Non-interactive	hsa:1445	D03806
Non-interactive	hsa:150290	D00655
Non-interactive	hsa:1504	D00328
Non-interactive	hsa:1504	D00448
Non-interactive	hsa:1504	D00533
Non-interactive	hsa:1504	D01866
Non-interactive	hsa:1504	D03689
Non-interactive	hsa:151531	D00218
Non-interactive	hsa:151531	D00650
Non-interactive	hsa:151531	D01196
Non-interactive	hsa:151531	D02671
Non-interactive	hsa:151531	D03710
Non-interactive	hsa:1537	D00225
Non-interactive	hsa:1537	D00650
Non-interactive	hsa:1537	D01918
Non-interactive	hsa:1537	D02214
Non-interactive	hsa:1543	D00148
Non-interactive	hsa:1543	D00188
Non-interactive	hsa:1543	D00315
Non-interactive	hsa:1543	D01885
Non-interactive	hsa:1543	D03816
Non-interactive	hsa:1544	D00448
Non-interactive	hsa:1544	D00519
Non-interactive	hsa:1544	D01133
Non-interactive	hsa:1544	D02328
Non-interactive	hsa:1544	D02756
Non-interactive	hsa:1544	D03805
Non-interactive	hsa:1545	D00538
Non-interactive	hsa:1545	D00550
Non-interactive	hsa:1545	D02333

Non-interactive	hsa:1545	D02556
Non-interactive	hsa:1545	D02709
Non-interactive	hsa:1545	D04292
Non-interactive	hsa:1548	D00196
Non-interactive	hsa:1548	D00325
Non-interactive	hsa:1548	D00650
Non-interactive	hsa:1548	D01061
Non-interactive	hsa:1548	D02173
Non-interactive	hsa:1549	D00252
Non-interactive	hsa:1549	D00516
Non-interactive	hsa:1549	D02368
Non-interactive	hsa:1549	D03440
Non-interactive	hsa:1549	D03716
Non-interactive	hsa:1551	D00188
Non-interactive	hsa:1551	D00198
Non-interactive	hsa:1551	D01275
Non-interactive	hsa:1551	D01718
Non-interactive	hsa:1551	D01977
Non-interactive	hsa:1553	D00315
Non-interactive	hsa:1553	D00963
Non-interactive	hsa:1553	D00965
Non-interactive	hsa:1553	D01275
Non-interactive	hsa:1553	D01974
Non-interactive	hsa:1553	D03826
Non-interactive	hsa:1555	D00218
Non-interactive	hsa:1555	D00651
Non-interactive	hsa:1555	D01180
Non-interactive	hsa:1555	D01240
Non-interactive	hsa:1555	D01907
Non-interactive	hsa:1555	D02769
Non-interactive	hsa:1557	D02168
Non-interactive	hsa:1558	D00893
Non-interactive	hsa:1558	D00969
Non-interactive	hsa:1558	D01397
Non-interactive	hsa:1558	D01432
Non-interactive	hsa:1558	D03716
Non-interactive	hsa:1559	D03743
Non-interactive	hsa:1562	D00070
Non-interactive	hsa:1562	D00654
Non-interactive	hsa:1562	D00813
Non-interactive	hsa:1562	D00965
Non-interactive	hsa:1562	D01211

Non-interactive	hsa:1565	D00188
Non-interactive	hsa:1565	D00653
Non-interactive	hsa:1565	D01690
Non-interactive	hsa:1565	D01918
Non-interactive	hsa:1571	D00387
Non-interactive	hsa:1571	D00753
Non-interactive	hsa:1571	D01918
Non-interactive	hsa:1571	D02042
Non-interactive	hsa:1572	D00892
Non-interactive	hsa:1572	D01119
Non-interactive	hsa:1572	D01767
Non-interactive	hsa:1572	D01844
Non-interactive	hsa:1572	D03735
Non-interactive	hsa:1573	D00070
Non-interactive	hsa:1573	D00359
Non-interactive	hsa:1573	D01332
Non-interactive	hsa:1573	D01918
Non-interactive	hsa:1573	D03775
Non-interactive	hsa:1576	D00324
Non-interactive	hsa:1576	D00620
Non-interactive	hsa:1576	D01688
Non-interactive	hsa:1576	D02487
Non-interactive	hsa:1577	D00650
Non-interactive	hsa:1577	D00884
Non-interactive	hsa:1577	D01866
Non-interactive	hsa:1577	D01907
Non-interactive	hsa:1577	D03643
Non-interactive	hsa:1579	D00315
Non-interactive	hsa:1579	D03350
Non-interactive	hsa:1580	D00530
Non-interactive	hsa:1580	D00651
Non-interactive	hsa:1580	D00965
Non-interactive	hsa:1580	D01061
Non-interactive	hsa:1580	D03765
Non-interactive	hsa:1581	D00455
Non-interactive	hsa:1581	D00463
Non-interactive	hsa:1581	D01180
Non-interactive	hsa:1582	D00018
Non-interactive	hsa:1582	D00294
Non-interactive	hsa:1582	D00364
Non-interactive	hsa:1582	D00752
Non-interactive	hsa:1582	D03805

Non-interactive	hsa:1583	D00454
Non-interactive	hsa:1583	D00538
Non-interactive	hsa:1583	D01973
Non-interactive	hsa:1583	D03743
Non-interactive	hsa:1584	D00563
Non-interactive	hsa:1585	D00620
Non-interactive	hsa:1585	D00658
Non-interactive	hsa:1585	D03712
Non-interactive	hsa:1586	D00328
Non-interactive	hsa:1586	D00752
Non-interactive	hsa:1586	D00805
Non-interactive	hsa:1586	D03778
Non-interactive	hsa:1586	D04025
Non-interactive	hsa:1588	D00533
Non-interactive	hsa:1588	D00620
Non-interactive	hsa:1588	D03751
Non-interactive	hsa:1588	D03767
Non-interactive	hsa:1589	D00198
Non-interactive	hsa:1589	D00537
Non-interactive	hsa:1589	D01578
Non-interactive	hsa:1589	D01911
Non-interactive	hsa:1589	D02655
Non-interactive	hsa:1593	D00148
Non-interactive	hsa:1593	D01918
Non-interactive	hsa:1593	D03218
Non-interactive	hsa:1593	D03728
Non-interactive	hsa:1594	D00196
Non-interactive	hsa:1594	D00622
Non-interactive	hsa:1594	D00781
Non-interactive	hsa:1594	D00813
Non-interactive	hsa:1594	D01119
Non-interactive	hsa:1595	D00285
Non-interactive	hsa:1595	D01223
Non-interactive	hsa:1610	D00283
Non-interactive	hsa:1610	D00324
Non-interactive	hsa:1610	D00359
Non-interactive	hsa:1610	D02173
Non-interactive	hsa:1610	D02580
Non-interactive	hsa:1621	D00488
Non-interactive	hsa:1621	D00623
Non-interactive	hsa:1621	D02115
Non-interactive	hsa:1621	D03720

Non-interactive	hsa:1621	D03728
Non-interactive	hsa:1633	D00298
Non-interactive	hsa:1633	D00596
Non-interactive	hsa:1633	D01367
Non-interactive	hsa:1636	D00002
Non-interactive	hsa:1636	D00656
Non-interactive	hsa:1636	D02451
Non-interactive	hsa:1636	D04029
Non-interactive	hsa:1645	D00141
Non-interactive	hsa:1645	D00653
Non-interactive	hsa:1645	D00893
Non-interactive	hsa:1645	D01862
Non-interactive	hsa:1675	D00054
Non-interactive	hsa:1675	D01180
Non-interactive	hsa:1675	D03803
Non-interactive	hsa:1675	D04024
Non-interactive	hsa:1719	D00550
Non-interactive	hsa:1719	D01332
Non-interactive	hsa:1719	D03710
Non-interactive	hsa:1719	D03712
Non-interactive	hsa:1723	D00398
Non-interactive	hsa:1723	D00805
Non-interactive	hsa:1723	D01071
Non-interactive	hsa:1723	D01164
Non-interactive	hsa:1723	D01196
Non-interactive	hsa:1723	D01397
Non-interactive	hsa:1723	D03828
Non-interactive	hsa:1725	D00449
Non-interactive	hsa:1725	D00947
Non-interactive	hsa:1725	D01275
Non-interactive	hsa:1725	D01915
Non-interactive	hsa:1725	D02110
Non-interactive	hsa:1728	D00563
Non-interactive	hsa:1728	D03807
Non-interactive	hsa:1728	D03826
Non-interactive	hsa:1800	D00423
Non-interactive	hsa:1800	D00733
Non-interactive	hsa:1800	D01196
Non-interactive	hsa:1803	D00623
Non-interactive	hsa:1803	D01432
Non-interactive	hsa:1806	D00094
Non-interactive	hsa:1806	D01256

Non-interactive	hsa:1806	D02194
Non-interactive	hsa:1806	D02671
Non-interactive	hsa:1843	D00198
Non-interactive	hsa:1843	D01275
Non-interactive	hsa:1843	D01984
Non-interactive	hsa:1843	D02110
Non-interactive	hsa:1843	D02655
Non-interactive	hsa:1843	D03077
Non-interactive	hsa:1844	D00155
Non-interactive	hsa:1844	D00359
Non-interactive	hsa:1844	D00620
Non-interactive	hsa:1844	D01432
Non-interactive	hsa:1845	D00656
Non-interactive	hsa:1846	D00018
Non-interactive	hsa:1846	D00902
Non-interactive	hsa:1846	D02214
Non-interactive	hsa:1846	D02581
Non-interactive	hsa:1846	D03758
Non-interactive	hsa:1847	D01911
Non-interactive	hsa:1848	D00653
Non-interactive	hsa:1848	D03077
Non-interactive	hsa:1849	D00218
Non-interactive	hsa:1849	D02328
Non-interactive	hsa:1849	D02731
Non-interactive	hsa:1850	D00120
Non-interactive	hsa:1850	D00131
Non-interactive	hsa:1850	D00752
Non-interactive	hsa:1850	D00889
Non-interactive	hsa:1850	D01180
Non-interactive	hsa:1850	D01397
Non-interactive	hsa:1850	D02193
Non-interactive	hsa:1850	D02451
Non-interactive	hsa:1852	D01765
Non-interactive	hsa:1852	D02368
Non-interactive	hsa:1852	D03758
Non-interactive	hsa:1890	D00120
Non-interactive	hsa:1890	D00141
Non-interactive	hsa:1890	D00155
Non-interactive	hsa:1890	D00218
Non-interactive	hsa:1890	D02110
Non-interactive	hsa:189	D00593
Non-interactive	hsa:189	D00889

Non-interactive	hsa:189	D01164
Non-interactive	hsa:189	D01578
Non-interactive	hsa:189	D02229
Non-interactive	hsa:189	D02258
Non-interactive	hsa:189	D03758
Non-interactive	hsa:18	D00002
Non-interactive	hsa:18	D00294
Non-interactive	hsa:18	D00455
Non-interactive	hsa:18	D01441
Non-interactive	hsa:18	D02418
Non-interactive	hsa:191	D00142
Non-interactive	hsa:191	D00293
Non-interactive	hsa:191	D00294
Non-interactive	hsa:191	D00434
Non-interactive	hsa:1956	D00188
Non-interactive	hsa:1956	D00359
Non-interactive	hsa:1956	D01240
Non-interactive	hsa:1956	D01915
Non-interactive	hsa:196883	D00120
Non-interactive	hsa:196883	D02418
Non-interactive	hsa:196883	D02709
Non-interactive	hsa:1969	D00813
Non-interactive	hsa:1969	D01211
Non-interactive	hsa:1969	D02115
Non-interactive	hsa:1990	D03882
Non-interactive	hsa:1991	D00252
Non-interactive	hsa:1991	D01180
Non-interactive	hsa:1991	D04029
Non-interactive	hsa:199974	D00448
Non-interactive	hsa:199974	D00965
Non-interactive	hsa:199974	D03767
Non-interactive	hsa:199974	D03775
Non-interactive	hsa:2041	D00141
Non-interactive	hsa:2041	D00198
Non-interactive	hsa:2041	D00454
Non-interactive	hsa:2041	D00752
Non-interactive	hsa:2041	D01027
Non-interactive	hsa:2041	D01064
Non-interactive	hsa:2042	D00203
Non-interactive	hsa:2042	D00623
Non-interactive	hsa:2042	D00752
Non-interactive	hsa:2042	D00813

Non-interactive	hsa:2042	D01718
Non-interactive	hsa:2043	D00533
Non-interactive	hsa:2043	D00538
Non-interactive	hsa:2043	D01027
Non-interactive	hsa:2043	D02368
Non-interactive	hsa:2043	D03758
Non-interactive	hsa:2043	D03805
Non-interactive	hsa:2044	D01061
Non-interactive	hsa:2044	D01211
Non-interactive	hsa:2044	D03735
Non-interactive	hsa:2045	D00283
Non-interactive	hsa:2045	D00652
Non-interactive	hsa:2045	D02115
Non-interactive	hsa:2045	D02580
Non-interactive	hsa:2045	D03440
Non-interactive	hsa:2045	D03882
Non-interactive	hsa:2046	D00394
Non-interactive	hsa:2046	D01432
Non-interactive	hsa:2046	D01862
Non-interactive	hsa:2046	D02655
Non-interactive	hsa:2047	D00448
Non-interactive	hsa:2047	D02731
Non-interactive	hsa:2048	D00120
Non-interactive	hsa:2048	D00752
Non-interactive	hsa:2048	D02042
Non-interactive	hsa:2048	D02229
Non-interactive	hsa:2048	D03012
Non-interactive	hsa:2048	D03741
Non-interactive	hsa:2049	D00455
Non-interactive	hsa:2049	D00463
Non-interactive	hsa:2049	D02368
Non-interactive	hsa:2049	D02487
Non-interactive	hsa:2049	D03765
Non-interactive	hsa:2049	D03788
Non-interactive	hsa:2050	D00142
Non-interactive	hsa:2050	D01367
Non-interactive	hsa:2050	D01432
Non-interactive	hsa:2050	D03717
Non-interactive	hsa:2050	D03735
Non-interactive	hsa:2051	D02560
Non-interactive	hsa:2051	D02581
Non-interactive	hsa:2058	D00398

Non-interactive	hsa:2058	D00813
Non-interactive	hsa:2058	D01119
Non-interactive	hsa:2058	D01180
Non-interactive	hsa:2058	D01842
Non-interactive	hsa:2058	D02556
Non-interactive	hsa:2058	D03767
Non-interactive	hsa:2064	D00437
Non-interactive	hsa:2065	D00002
Non-interactive	hsa:2065	D00187
Non-interactive	hsa:2065	D01133
Non-interactive	hsa:2065	D02110
Non-interactive	hsa:2065	D02581
Non-interactive	hsa:2065	D03077
Non-interactive	hsa:2066	D00359
Non-interactive	hsa:2066	D00463
Non-interactive	hsa:2098	D00188
Non-interactive	hsa:2098	D00463
Non-interactive	hsa:2098	D03689
Non-interactive	hsa:2135	D01973
Non-interactive	hsa:2147	D00620
Non-interactive	hsa:2147	D02769
Non-interactive	hsa:2147	D03350
Non-interactive	hsa:2147	D03758
Non-interactive	hsa:2147	D03767
Non-interactive	hsa:2155	D00387
Non-interactive	hsa:2155	D00623
Non-interactive	hsa:2155	D00753
Non-interactive	hsa:2155	D02560
Non-interactive	hsa:2158	D01164
Non-interactive	hsa:2158	D01973
Non-interactive	hsa:2159	D00094
Non-interactive	hsa:2159	D01915
Non-interactive	hsa:2159	D03712
Non-interactive	hsa:2159	D03736
Non-interactive	hsa:2160	D00463
Non-interactive	hsa:2160	D03775
Non-interactive	hsa:2161	D00216
Non-interactive	hsa:2161	D00387
Non-interactive	hsa:2161	D01842
Non-interactive	hsa:2161	D01984
Non-interactive	hsa:216	D00120
Non-interactive	hsa:216	D02731

Non-interactive	hsa:216	D03775
Non-interactive	hsa:217	D00387
Non-interactive	hsa:217	D00434
Non-interactive	hsa:217	D01211
Non-interactive	hsa:217	D01866
Non-interactive	hsa:2180	D00414
Non-interactive	hsa:2180	D00892
Non-interactive	hsa:2180	D01061
Non-interactive	hsa:2180	D01844
Non-interactive	hsa:2180	D01918
Non-interactive	hsa:2185	D00752
Non-interactive	hsa:2185	D02328
Non-interactive	hsa:2185	D03689
Non-interactive	hsa:2185	D03720
Non-interactive	hsa:2185	D03728
Non-interactive	hsa:218	D00216
Non-interactive	hsa:218	D00423
Non-interactive	hsa:218	D02756
Non-interactive	hsa:218	D03716
Non-interactive	hsa:218	D03765
Non-interactive	hsa:2193	D00537
Non-interactive	hsa:2193	D01164
Non-interactive	hsa:2193	D01240
Non-interactive	hsa:2193	D02375
Non-interactive	hsa:219	D00139
Non-interactive	hsa:219	D00567
Non-interactive	hsa:219	D00658
Non-interactive	hsa:219	D00885
Non-interactive	hsa:219	D00969
Non-interactive	hsa:219	D01690
Non-interactive	hsa:219	D01866
Non-interactive	hsa:219	D02194
Non-interactive	hsa:219	D02709
Non-interactive	hsa:219	D03350
Non-interactive	hsa:219	D03775
Non-interactive	hsa:2224	D00515
Non-interactive	hsa:2224	D03077
Non-interactive	hsa:2232	D00781
Non-interactive	hsa:2232	D01264
Non-interactive	hsa:2232	D01885
Non-interactive	hsa:2235	D00488
Non-interactive	hsa:2235	D00813

Non-interactive	hsa:2235	D04029
Non-interactive	hsa:223	D00651
Non-interactive	hsa:223	D00965
Non-interactive	hsa:223	D03736
Non-interactive	hsa:2241	D00387
Non-interactive	hsa:2241	D03012
Non-interactive	hsa:2241	D03350
Non-interactive	hsa:2242	D00252
Non-interactive	hsa:2242	D00394
Non-interactive	hsa:2242	D01196
Non-interactive	hsa:2242	D02556
Non-interactive	hsa:2242	D03788
Non-interactive	hsa:224	D00252
Non-interactive	hsa:224	D03758
Non-interactive	hsa:225689	D00285
Non-interactive	hsa:225689	D00650
Non-interactive	hsa:225689	D01256
Non-interactive	hsa:225689	D01842
Non-interactive	hsa:2260	D00325
Non-interactive	hsa:2260	D00753
Non-interactive	hsa:2260	D00900
Non-interactive	hsa:2260	D01397
Non-interactive	hsa:2260	D02580
Non-interactive	hsa:2261	D00203
Non-interactive	hsa:2261	D02193
Non-interactive	hsa:2261	D02229
Non-interactive	hsa:2261	D02769
Non-interactive	hsa:2261	D03775
Non-interactive	hsa:2263	D00283
Non-interactive	hsa:2263	D00398
Non-interactive	hsa:2263	D02115
Non-interactive	hsa:2264	D00139
Non-interactive	hsa:2264	D00225
Non-interactive	hsa:2264	D01918
Non-interactive	hsa:2264	D01984
Non-interactive	hsa:2264	D03712
Non-interactive	hsa:2280	D01885
Non-interactive	hsa:22843	D00018
Non-interactive	hsa:22843	D03218
Non-interactive	hsa:22843	D03440
Non-interactive	hsa:22954	D00141
Non-interactive	hsa:22954	D00449

Non-interactive	hsa:22954	D01164
Non-interactive	hsa:22954	D01196
Non-interactive	hsa:22954	D01240
Non-interactive	hsa:22954	D02368
Non-interactive	hsa:22954	D02756
Non-interactive	hsa:22978	D00187
Non-interactive	hsa:22978	D00196
Non-interactive	hsa:22978	D00218
Non-interactive	hsa:22978	D00622
Non-interactive	hsa:22978	D00655
Non-interactive	hsa:22978	D01119
Non-interactive	hsa:22978	D01367
Non-interactive	hsa:22978	D03689
Non-interactive	hsa:22978	D03798
Non-interactive	hsa:22978	D03828
Non-interactive	hsa:23035	D00515
Non-interactive	hsa:23035	D02008
Non-interactive	hsa:231	D01918
Non-interactive	hsa:231	D02166
Non-interactive	hsa:231	D04029
Non-interactive	hsa:2321	D00094
Non-interactive	hsa:2321	D00893
Non-interactive	hsa:2321	D02166
Non-interactive	hsa:2322	D00893
Non-interactive	hsa:2322	D01180
Non-interactive	hsa:2322	D01767
Non-interactive	hsa:23236	D00596
Non-interactive	hsa:23236	D01370
Non-interactive	hsa:23236	D01582
Non-interactive	hsa:23236	D01974
Non-interactive	hsa:23236	D02193
Non-interactive	hsa:23236	D03712
Non-interactive	hsa:23239	D00658
Non-interactive	hsa:23239	D01973
Non-interactive	hsa:23239	D02487
Non-interactive	hsa:2324	D00120
Non-interactive	hsa:2324	D00889
Non-interactive	hsa:2324	D01715
Non-interactive	hsa:2328	D00463
Non-interactive	hsa:2328	D00658
Non-interactive	hsa:2328	D00805
Non-interactive	hsa:2328	D02418

Non-interactive	hsa:2328	D03882
Non-interactive	hsa:2339	D00623
Non-interactive	hsa:2339	D00947
Non-interactive	hsa:2339	D02194
Non-interactive	hsa:2339	D02698
Non-interactive	hsa:2342	D02333
Non-interactive	hsa:2342	D03767
Non-interactive	hsa:23430	D00969
Non-interactive	hsa:23430	D03736
Non-interactive	hsa:23430	D04292
Non-interactive	hsa:23436	D00070
Non-interactive	hsa:23436	D00142
Non-interactive	hsa:23436	D00148
Non-interactive	hsa:23436	D00294
Non-interactive	hsa:23436	D00654
Non-interactive	hsa:23436	D00902
Non-interactive	hsa:23436	D01164
Non-interactive	hsa:23436	D03751
Non-interactive	hsa:23475	D00139
Non-interactive	hsa:23475	D00620
Non-interactive	hsa:23475	D03767
Non-interactive	hsa:23475	D03882
Non-interactive	hsa:2356	D01915
Non-interactive	hsa:2356	D01974
Non-interactive	hsa:23632	D00398
Non-interactive	hsa:23632	D00455
Non-interactive	hsa:23632	D00651
Non-interactive	hsa:23632	D01264
Non-interactive	hsa:23632	D01578
Non-interactive	hsa:23632	D03736
Non-interactive	hsa:23632	D04029
Non-interactive	hsa:238	D00658
Non-interactive	hsa:238	D02214
Non-interactive	hsa:239	D00005
Non-interactive	hsa:239	D00298
Non-interactive	hsa:239	D00893
Non-interactive	hsa:239	D01223
Non-interactive	hsa:239	D01578
Non-interactive	hsa:239	D02328
Non-interactive	hsa:240	D00650
Non-interactive	hsa:240	D00656
Non-interactive	hsa:240	D03218

Non-interactive	hsa:240	D03440
Non-interactive	hsa:240	D03710
Non-interactive	hsa:242	D00127
Non-interactive	hsa:242	D00216
Non-interactive	hsa:242	D00434
Non-interactive	hsa:242	D00651
Non-interactive	hsa:242	D02008
Non-interactive	hsa:246	D00187
Non-interactive	hsa:246	D00325
Non-interactive	hsa:247	D00094
Non-interactive	hsa:247	D00208
Non-interactive	hsa:247	D00537
Non-interactive	hsa:247	D00753
Non-interactive	hsa:247	D01264
Non-interactive	hsa:247	D01885
Non-interactive	hsa:2534	D00900
Non-interactive	hsa:2534	D03689
Non-interactive	hsa:2534	D03826
Non-interactive	hsa:2548	D00002
Non-interactive	hsa:2548	D00391
Non-interactive	hsa:2548	D00885
Non-interactive	hsa:2548	D02193
Non-interactive	hsa:2548	D02556
Non-interactive	hsa:25796	D03350
Non-interactive	hsa:25796	D03734
Non-interactive	hsa:25796	D03778
Non-interactive	hsa:25824	D00005
Non-interactive	hsa:25824	D00596
Non-interactive	hsa:25824	D00805
Non-interactive	hsa:25824	D00969
Non-interactive	hsa:25824	D02258
Non-interactive	hsa:25824	D03720
Non-interactive	hsa:2582	D00005
Non-interactive	hsa:2582	D00125
Non-interactive	hsa:2582	D00900
Non-interactive	hsa:2582	D01264
Non-interactive	hsa:2582	D03798
Non-interactive	hsa:2595	D00463
Non-interactive	hsa:2595	D00654
Non-interactive	hsa:2595	D00965
Non-interactive	hsa:2595	D03440
Non-interactive	hsa:2595	D03689

Non-interactive	hsa:2595	D03828
Non-interactive	hsa:2597	D00359
Non-interactive	hsa:2597	D01061
Non-interactive	hsa:2597	D02581
Non-interactive	hsa:2597	D03788
Non-interactive	hsa:25	D00434
Non-interactive	hsa:25	D02258
Non-interactive	hsa:25	D02375
Non-interactive	hsa:25	D04031
Non-interactive	hsa:260293	D00155
Non-interactive	hsa:260293	D01765
Non-interactive	hsa:26279	D00655
Non-interactive	hsa:26279	D01180
Non-interactive	hsa:26279	D01196
Non-interactive	hsa:26279	D01264
Non-interactive	hsa:26279	D01828
Non-interactive	hsa:26279	D03218
Non-interactive	hsa:26279	D03734
Non-interactive	hsa:2638	D00216
Non-interactive	hsa:2638	D03826
Non-interactive	hsa:2639	D00127
Non-interactive	hsa:2639	D00892
Non-interactive	hsa:2639	D02671
Non-interactive	hsa:2673	D00813
Non-interactive	hsa:2673	D02731
Non-interactive	hsa:2677	D00359
Non-interactive	hsa:2677	D03710
Non-interactive	hsa:27032	D02042
Non-interactive	hsa:27034	D00437
Non-interactive	hsa:27034	D00533
Non-interactive	hsa:27034	D01844
Non-interactive	hsa:27034	D01866
Non-interactive	hsa:27034	D02731
Non-interactive	hsa:27034	D03735
Non-interactive	hsa:27034	D03882
Non-interactive	hsa:270	D00391
Non-interactive	hsa:270	D01211
Non-interactive	hsa:270	D02229
Non-interactive	hsa:2746	D00364
Non-interactive	hsa:2746	D00516
Non-interactive	hsa:2746	D00530
Non-interactive	hsa:2746	D00651

Non-interactive	hsa:2746	D01885
Non-interactive	hsa:2746	D01915
Non-interactive	hsa:2746	D03735
Non-interactive	hsa:2766	D00651
Non-interactive	hsa:2766	D00947
Non-interactive	hsa:2766	D01276
Non-interactive	hsa:2766	D01974
Non-interactive	hsa:2766	D02008
Non-interactive	hsa:279	D00771
Non-interactive	hsa:279	D00805
Non-interactive	hsa:279	D00889
Non-interactive	hsa:279	D02110
Non-interactive	hsa:279	D02580
Non-interactive	hsa:2806	D00398
Non-interactive	hsa:2806	D01911
Non-interactive	hsa:2806	D02110
Non-interactive	hsa:28227	D00328
Non-interactive	hsa:28227	D01264
Non-interactive	hsa:2822	D00328
Non-interactive	hsa:2822	D00364
Non-interactive	hsa:2822	D00550
Non-interactive	hsa:2822	D00593
Non-interactive	hsa:2822	D00650
Non-interactive	hsa:2822	D00947
Non-interactive	hsa:2822	D00963
Non-interactive	hsa:2822	D01432
Non-interactive	hsa:2822	D01582
Non-interactive	hsa:2822	D01984
Non-interactive	hsa:284541	D00315
Non-interactive	hsa:284541	D00753
Non-interactive	hsa:284541	D01825
Non-interactive	hsa:284541	D01828
Non-interactive	hsa:284541	D03798
Non-interactive	hsa:285220	D00596
Non-interactive	hsa:285220	D00752
Non-interactive	hsa:285220	D01582
Non-interactive	hsa:285220	D01866
Non-interactive	hsa:2880	D00188
Non-interactive	hsa:2880	D00771
Non-interactive	hsa:2880	D01765
Non-interactive	hsa:2882	D01196
Non-interactive	hsa:2882	D01565

Non-interactive	hsa:2882	D01915
Non-interactive	hsa:2882	D03077
Non-interactive	hsa:28972	D00658
Non-interactive	hsa:28972	D02008
Non-interactive	hsa:28972	D03735
Non-interactive	hsa:28972	D03899
Non-interactive	hsa:28	D00005
Non-interactive	hsa:28	D00414
Non-interactive	hsa:28	D00423
Non-interactive	hsa:28	D00893
Non-interactive	hsa:28	D00963
Non-interactive	hsa:28	D01064
Non-interactive	hsa:2936	D00622
Non-interactive	hsa:2936	D01578
Non-interactive	hsa:2936	D01690
Non-interactive	hsa:2950	D00519
Non-interactive	hsa:2950	D03741
Non-interactive	hsa:2950	D03778
Non-interactive	hsa:29785	D00892
Non-interactive	hsa:29785	D01275
Non-interactive	hsa:29785	D02193
Non-interactive	hsa:29785	D02655
Non-interactive	hsa:29785	D03712
Non-interactive	hsa:29941	D00733
Non-interactive	hsa:29941	D00969
Non-interactive	hsa:29968	D00252
Non-interactive	hsa:3002	D01164
Non-interactive	hsa:3002	D02580
Non-interactive	hsa:3002	D02709
Non-interactive	hsa:3002	D03803
Non-interactive	hsa:3028	D03775
Non-interactive	hsa:3033	D00216
Non-interactive	hsa:3033	D00753
Non-interactive	hsa:3033	D03716
Non-interactive	hsa:3034	D00216
Non-interactive	hsa:3034	D00294
Non-interactive	hsa:3034	D00752
Non-interactive	hsa:3034	D01275
Non-interactive	hsa:3034	D03712
Non-interactive	hsa:3035	D00533
Non-interactive	hsa:3035	D00902
Non-interactive	hsa:3035	D01578

Non-interactive	hsa:3055	D00188
Non-interactive	hsa:3055	D00394
Non-interactive	hsa:3055	D00563
Non-interactive	hsa:3055	D01885
Non-interactive	hsa:3055	D02580
Non-interactive	hsa:3067	D00537
Non-interactive	hsa:3067	D00538
Non-interactive	hsa:3067	D01973
Non-interactive	hsa:306	D00142
Non-interactive	hsa:306	D00434
Non-interactive	hsa:306	D00533
Non-interactive	hsa:306	D03736
Non-interactive	hsa:306	D03778
Non-interactive	hsa:306	D03828
Non-interactive	hsa:30814	D00414
Non-interactive	hsa:30814	D00969
Non-interactive	hsa:30833	D01332
Non-interactive	hsa:30	D00094
Non-interactive	hsa:30	D00434
Non-interactive	hsa:30	D01064
Non-interactive	hsa:30	D01133
Non-interactive	hsa:30	D01765
Non-interactive	hsa:30	D01915
Non-interactive	hsa:313	D00125
Non-interactive	hsa:313	D00364
Non-interactive	hsa:313	D01370
Non-interactive	hsa:313	D01688
Non-interactive	hsa:313	D01715
Non-interactive	hsa:313	D01974
Non-interactive	hsa:313	D02441
Non-interactive	hsa:3141	D00018
Non-interactive	hsa:3141	D00196
Non-interactive	hsa:3141	D00252
Non-interactive	hsa:3141	D01180
Non-interactive	hsa:3141	D03012
Non-interactive	hsa:3141	D03643
Non-interactive	hsa:3156	D00813
Non-interactive	hsa:3156	D03751
Non-interactive	hsa:31	D00125
Non-interactive	hsa:31	D01842
Non-interactive	hsa:326625	D00414
Non-interactive	hsa:326625	D00771

Non-interactive	hsa:326625	D00885
Non-interactive	hsa:326625	D01164
Non-interactive	hsa:326625	D01688
Non-interactive	hsa:326625	D02194
Non-interactive	hsa:326625	D03728
Non-interactive	hsa:327	D00139
Non-interactive	hsa:327	D00884
Non-interactive	hsa:327	D01844
Non-interactive	hsa:327	D02487
Non-interactive	hsa:3283	D01715
Non-interactive	hsa:3283	D03077
Non-interactive	hsa:3283	D03798
Non-interactive	hsa:3290	D02214
Non-interactive	hsa:3290	D02556
Non-interactive	hsa:3290	D04029
Non-interactive	hsa:3291	D00515
Non-interactive	hsa:3291	D00752
Non-interactive	hsa:3291	D00969
Non-interactive	hsa:3291	D01565
Non-interactive	hsa:3292	D00515
Non-interactive	hsa:3292	D00538
Non-interactive	hsa:3292	D01397
Non-interactive	hsa:3292	D03728
Non-interactive	hsa:3292	D03806
Non-interactive	hsa:3295	D00387
Non-interactive	hsa:3295	D01061
Non-interactive	hsa:3295	D01211
Non-interactive	hsa:3295	D01565
Non-interactive	hsa:3295	D02581
Non-interactive	hsa:32	D00658
Non-interactive	hsa:32	D01862
Non-interactive	hsa:32	D01866
Non-interactive	hsa:32	D02008
Non-interactive	hsa:32	D02769
Non-interactive	hsa:32	D03767
Non-interactive	hsa:3376	D00449
Non-interactive	hsa:3376	D00947
Non-interactive	hsa:3376	D01862
Non-interactive	hsa:3376	D02709
Non-interactive	hsa:3376	D03806
Non-interactive	hsa:339221	D01061
Non-interactive	hsa:339221	D01275

Non-interactive	hsa:339221	D01862
Non-interactive	hsa:339221	D03720
Non-interactive	hsa:3416	D00965
Non-interactive	hsa:3416	D02698
Non-interactive	hsa:3480	D00437
Non-interactive	hsa:3480	D01885
Non-interactive	hsa:3480	D02008
Non-interactive	hsa:3480	D03735
Non-interactive	hsa:3480	D03823
Non-interactive	hsa:349565	D00131
Non-interactive	hsa:349565	D00188
Non-interactive	hsa:349565	D00781
Non-interactive	hsa:34	D01718
Non-interactive	hsa:34	D02328
Non-interactive	hsa:34	D03767
Non-interactive	hsa:353	D00155
Non-interactive	hsa:353	D00656
Non-interactive	hsa:353	D00753
Non-interactive	hsa:353	D01915
Non-interactive	hsa:354	D00070
Non-interactive	hsa:354	D00454
Non-interactive	hsa:354	D01119
Non-interactive	hsa:354	D01332
Non-interactive	hsa:3551	D01582
Non-interactive	hsa:3551	D01918
Non-interactive	hsa:3551	D02560
Non-interactive	hsa:35	D00218
Non-interactive	hsa:35	D00434
Non-interactive	hsa:35	D01223
Non-interactive	hsa:35	D01907
Non-interactive	hsa:3612	D00622
Non-interactive	hsa:3612	D02375
Non-interactive	hsa:3614	D00503
Non-interactive	hsa:3614	D00579
Non-interactive	hsa:3615	D00620
Non-interactive	hsa:3615	D03743
Non-interactive	hsa:3615	D03775
Non-interactive	hsa:3643	D00449
Non-interactive	hsa:3643	D00567
Non-interactive	hsa:3643	D00969
Non-interactive	hsa:3645	D03440
Non-interactive	hsa:3645	D03689

Non-interactive	hsa:3645	D03823
Non-interactive	hsa:36	D00359
Non-interactive	hsa:36	D00650
Non-interactive	hsa:36	D00652
Non-interactive	hsa:36	D03012
Non-interactive	hsa:36	D03712
Non-interactive	hsa:36	D03805
Non-interactive	hsa:3702	D00142
Non-interactive	hsa:3702	D00947
Non-interactive	hsa:3702	D03778
Non-interactive	hsa:3712	D00294
Non-interactive	hsa:3712	D03738
Non-interactive	hsa:3712	D03758
Non-interactive	hsa:3716	D00488
Non-interactive	hsa:3716	D01223
Non-interactive	hsa:3716	D02168
Non-interactive	hsa:3717	D00652
Non-interactive	hsa:3717	D00752
Non-interactive	hsa:3717	D01828
Non-interactive	hsa:3717	D03826
Non-interactive	hsa:3718	D00148
Non-interactive	hsa:3718	D00391
Non-interactive	hsa:3718	D00965
Non-interactive	hsa:3718	D03720
Non-interactive	hsa:3718	D03767
Non-interactive	hsa:3735	D01767
Non-interactive	hsa:3735	D01866
Non-interactive	hsa:3735	D03798
Non-interactive	hsa:377677	D00125
Non-interactive	hsa:377677	D00285
Non-interactive	hsa:377677	D02258
Non-interactive	hsa:377677	D02671
Non-interactive	hsa:3791	D00947
Non-interactive	hsa:3791	D01844
Non-interactive	hsa:3791	D03806
Non-interactive	hsa:3791	D03826
Non-interactive	hsa:3815	D00188
Non-interactive	hsa:3815	D03738
Non-interactive	hsa:3815	D03826
Non-interactive	hsa:3816	D00887
Non-interactive	hsa:3816	D01907
Non-interactive	hsa:3816	D02418

Non-interactive	hsa:3817	D00155
Non-interactive	hsa:3817	D00283
Non-interactive	hsa:3817	D00340
Non-interactive	hsa:3817	D00359
Non-interactive	hsa:3817	D01180
Non-interactive	hsa:3817	D01911
Non-interactive	hsa:3817	D03710
Non-interactive	hsa:3817	D03743
Non-interactive	hsa:3818	D00387
Non-interactive	hsa:3818	D00530
Non-interactive	hsa:3818	D01977
Non-interactive	hsa:3818	D03806
Non-interactive	hsa:3818	D04031
Non-interactive	hsa:38	D01715
Non-interactive	hsa:38	D02731
Non-interactive	hsa:3906	D00120
Non-interactive	hsa:3906	D03440
Non-interactive	hsa:3906	D04024
Non-interactive	hsa:390956	D01397
Non-interactive	hsa:390956	D02168
Non-interactive	hsa:390956	D03218
Non-interactive	hsa:390956	D03735
Non-interactive	hsa:3932	D00653
Non-interactive	hsa:3932	D00656
Non-interactive	hsa:3932	D00884
Non-interactive	hsa:3932	D01765
Non-interactive	hsa:3932	D03710
Non-interactive	hsa:3939	D01918
Non-interactive	hsa:3939	D02709
Non-interactive	hsa:3945	D02418
Non-interactive	hsa:3988	D00141
Non-interactive	hsa:3988	D00394
Non-interactive	hsa:3988	D01133
Non-interactive	hsa:3988	D02166
Non-interactive	hsa:3988	D04029
Non-interactive	hsa:3990	D00070
Non-interactive	hsa:3990	D00398
Non-interactive	hsa:3990	D00947
Non-interactive	hsa:3990	D03440
Non-interactive	hsa:3990	D03735
Non-interactive	hsa:3990	D03741
Non-interactive	hsa:3990	D03751

Non-interactive	hsa:3990	D03765
Non-interactive	hsa:3990	D03805
Non-interactive	hsa:3991	D00315
Non-interactive	hsa:3991	D00328
Non-interactive	hsa:3991	D00437
Non-interactive	hsa:3991	D00515
Non-interactive	hsa:4017	D00252
Non-interactive	hsa:4017	D00325
Non-interactive	hsa:4017	D04029
Non-interactive	hsa:4023	D00328
Non-interactive	hsa:4023	D00463
Non-interactive	hsa:4023	D01582
Non-interactive	hsa:4023	D02166
Non-interactive	hsa:4023	D04024
Non-interactive	hsa:4025	D00107
Non-interactive	hsa:4025	D00187
Non-interactive	hsa:4025	D00203
Non-interactive	hsa:4025	D00340
Non-interactive	hsa:4025	D00449
Non-interactive	hsa:4025	D03218
Non-interactive	hsa:4048	D00753
Non-interactive	hsa:4048	D00947
Non-interactive	hsa:4048	D00969
Non-interactive	hsa:4048	D02698
Non-interactive	hsa:4051	D00018
Non-interactive	hsa:4051	D00283
Non-interactive	hsa:4051	D00298
Non-interactive	hsa:4051	D00781
Non-interactive	hsa:4051	D02194
Non-interactive	hsa:4051	D03689
Non-interactive	hsa:4051	D03823
Non-interactive	hsa:4051	D04292
Non-interactive	hsa:4058	D00315
Non-interactive	hsa:4058	D00519
Non-interactive	hsa:4058	D01844
Non-interactive	hsa:4058	D01866
Non-interactive	hsa:4058	D01907
Non-interactive	hsa:4058	D01918
Non-interactive	hsa:4058	D02451
Non-interactive	hsa:4058	D02581
Non-interactive	hsa:4058	D02756
Non-interactive	hsa:4058	D03758

Non-interactive	hsa:4058	D04031
Non-interactive	hsa:4067	D00208
Non-interactive	hsa:4067	D00538
Non-interactive	hsa:4067	D00630
Non-interactive	hsa:4067	D01688
Non-interactive	hsa:4067	D02671
Non-interactive	hsa:4067	D04024
Non-interactive	hsa:4128	D00208
Non-interactive	hsa:4128	D00283
Non-interactive	hsa:4128	D00434
Non-interactive	hsa:4128	D01133
Non-interactive	hsa:4128	D01240
Non-interactive	hsa:4128	D01367
Non-interactive	hsa:4128	D02168
Non-interactive	hsa:4129	D03828
Non-interactive	hsa:4143	D00141
Non-interactive	hsa:4143	D01275
Non-interactive	hsa:4145	D00142
Non-interactive	hsa:4145	D00364
Non-interactive	hsa:4145	D01578
Non-interactive	hsa:4145	D02731
Non-interactive	hsa:4190	D00325
Non-interactive	hsa:4190	D00364
Non-interactive	hsa:4190	D01565
Non-interactive	hsa:4190	D01973
Non-interactive	hsa:4190	D03751
Non-interactive	hsa:4191	D00503
Non-interactive	hsa:4191	D00656
Non-interactive	hsa:4191	D01275
Non-interactive	hsa:4191	D01862
Non-interactive	hsa:4191	D02173
Non-interactive	hsa:4191	D03712
Non-interactive	hsa:4191	D03716
Non-interactive	hsa:4200	D01918
Non-interactive	hsa:4200	D02168
Non-interactive	hsa:4200	D03758
Non-interactive	hsa:4233	D00127
Non-interactive	hsa:4233	D00394
Non-interactive	hsa:4233	D00771
Non-interactive	hsa:4233	D01196
Non-interactive	hsa:4233	D01367
Non-interactive	hsa:4233	D03728

Non-interactive	hsa:4282	D00752
Non-interactive	hsa:4282	D02110
Non-interactive	hsa:4282	D02560
Non-interactive	hsa:4311	D00107
Non-interactive	hsa:4311	D00216
Non-interactive	hsa:4311	D00652
Non-interactive	hsa:4311	D02173
Non-interactive	hsa:4329	D00414
Non-interactive	hsa:4329	D00781
Non-interactive	hsa:4329	D01061
Non-interactive	hsa:4329	D01825
Non-interactive	hsa:4329	D02709
Non-interactive	hsa:4329	D03689
Non-interactive	hsa:4353	D00018
Non-interactive	hsa:4353	D00423
Non-interactive	hsa:4353	D00519
Non-interactive	hsa:4353	D00947
Non-interactive	hsa:4353	D01275
Non-interactive	hsa:4353	D01688
Non-interactive	hsa:4353	D01915
Non-interactive	hsa:4353	D03806
Non-interactive	hsa:4353	D04025
Non-interactive	hsa:43	D02731
Non-interactive	hsa:43	D03736
Non-interactive	hsa:444	D00141
Non-interactive	hsa:444	D00651
Non-interactive	hsa:444	D00658
Non-interactive	hsa:444	D00805
Non-interactive	hsa:444	D00892
Non-interactive	hsa:444	D00969
Non-interactive	hsa:444	D01907
Non-interactive	hsa:444	D03077
Non-interactive	hsa:4482	D01432
Non-interactive	hsa:4486	D00203
Non-interactive	hsa:4486	D00965
Non-interactive	hsa:4486	D01918
Non-interactive	hsa:4486	D03689
Non-interactive	hsa:4548	D00414
Non-interactive	hsa:4548	D00448
Non-interactive	hsa:4548	D00515
Non-interactive	hsa:4548	D00654
Non-interactive	hsa:4548	D01974

Non-interactive	hsa:4548	D02110
Non-interactive	hsa:4548	D03643
Non-interactive	hsa:4548	D03751
Non-interactive	hsa:4552	D02328
Non-interactive	hsa:4552	D03350
Non-interactive	hsa:4593	D00620
Non-interactive	hsa:4593	D00892
Non-interactive	hsa:4593	D00963
Non-interactive	hsa:4593	D01196
Non-interactive	hsa:4593	D01866
Non-interactive	hsa:4593	D03899
Non-interactive	hsa:4594	D00125
Non-interactive	hsa:4594	D01441
Non-interactive	hsa:4594	D01690
Non-interactive	hsa:4594	D01825
Non-interactive	hsa:4594	D01915
Non-interactive	hsa:4594	D02451
Non-interactive	hsa:4594	D03798
Non-interactive	hsa:4758	D00463
Non-interactive	hsa:4758	D00893
Non-interactive	hsa:4758	D02115
Non-interactive	hsa:4759	D00596
Non-interactive	hsa:4759	D00889
Non-interactive	hsa:4759	D01064
Non-interactive	hsa:4759	D03803
Non-interactive	hsa:4759	D04292
Non-interactive	hsa:476	D00324
Non-interactive	hsa:476	D00328
Non-interactive	hsa:476	D01370
Non-interactive	hsa:4835	D01885
Non-interactive	hsa:4835	D03012
Non-interactive	hsa:4835	D04024
Non-interactive	hsa:4837	D00398
Non-interactive	hsa:4837	D01565
Non-interactive	hsa:4837	D02368
Non-interactive	hsa:4860	D00198
Non-interactive	hsa:4860	D00781
Non-interactive	hsa:4860	D00900
Non-interactive	hsa:4860	D01276
Non-interactive	hsa:4860	D01715
Non-interactive	hsa:4881	D00437
Non-interactive	hsa:4881	D01825

Non-interactive	hsa:4881	D01911
Non-interactive	hsa:4881	D01977
Non-interactive	hsa:4881	D02166
Non-interactive	hsa:4881	D02671
Non-interactive	hsa:4881	D03798
Non-interactive	hsa:4907	D00283
Non-interactive	hsa:4907	D00533
Non-interactive	hsa:4907	D00733
Non-interactive	hsa:4907	D01164
Non-interactive	hsa:4907	D01180
Non-interactive	hsa:4907	D01367
Non-interactive	hsa:4907	D02451
Non-interactive	hsa:4907	D03798
Non-interactive	hsa:4914	D00398
Non-interactive	hsa:4914	D00423
Non-interactive	hsa:4914	D00622
Non-interactive	hsa:4914	D00889
Non-interactive	hsa:4914	D03788
Non-interactive	hsa:4914	D03806
Non-interactive	hsa:4915	D00203
Non-interactive	hsa:4915	D00218
Non-interactive	hsa:4915	D00519
Non-interactive	hsa:4915	D03765
Non-interactive	hsa:4915	D03803
Non-interactive	hsa:4916	D00653
Non-interactive	hsa:4916	D01765
Non-interactive	hsa:4916	D03735
Non-interactive	hsa:4919	D00094
Non-interactive	hsa:4919	D00293
Non-interactive	hsa:4919	D00398
Non-interactive	hsa:4919	D00892
Non-interactive	hsa:4919	D03717
Non-interactive	hsa:4919	D04031
Non-interactive	hsa:4920	D00538
Non-interactive	hsa:4920	D00947
Non-interactive	hsa:4920	D01432
Non-interactive	hsa:4920	D03798
Non-interactive	hsa:4921	D00094
Non-interactive	hsa:4921	D00120
Non-interactive	hsa:4921	D00448
Non-interactive	hsa:4921	D00658
Non-interactive	hsa:4921	D02168

Non-interactive	hsa:4921	D03712
Non-interactive	hsa:4921	D03882
Non-interactive	hsa:4942	D00120
Non-interactive	hsa:4942	D00252
Non-interactive	hsa:4942	D00463
Non-interactive	hsa:4953	D00188
Non-interactive	hsa:4953	D00488
Non-interactive	hsa:4953	D01061
Non-interactive	hsa:4953	D01256
Non-interactive	hsa:4953	D01915
Non-interactive	hsa:4953	D03350
Non-interactive	hsa:4953	D03689
Non-interactive	hsa:495	D00196
Non-interactive	hsa:495	D00391
Non-interactive	hsa:495	D00752
Non-interactive	hsa:495	D02418
Non-interactive	hsa:495	D02655
Non-interactive	hsa:495	D02731
Non-interactive	hsa:49	D00781
Non-interactive	hsa:49	D01223
Non-interactive	hsa:49	D01397
Non-interactive	hsa:49	D01984
Non-interactive	hsa:49	D02008
Non-interactive	hsa:49	D03798
Non-interactive	hsa:501	D00563
Non-interactive	hsa:501	D01027
Non-interactive	hsa:501	D01397
Non-interactive	hsa:501	D02166
Non-interactive	hsa:501	D02368
Non-interactive	hsa:5033	D00196
Non-interactive	hsa:5033	D00620
Non-interactive	hsa:5033	D00885
Non-interactive	hsa:5033	D00887
Non-interactive	hsa:5033	D02166
Non-interactive	hsa:5033	D02214
Non-interactive	hsa:5033	D03788
Non-interactive	hsa:5033	D03899
Non-interactive	hsa:5045	D00018
Non-interactive	hsa:5045	D00448
Non-interactive	hsa:5045	D00651
Non-interactive	hsa:5045	D01441
Non-interactive	hsa:5045	D01688

Non-interactive	hsa:50484	D03741
Non-interactive	hsa:50487	D02671
Non-interactive	hsa:50487	D03743
Non-interactive	hsa:50487	D03758
Non-interactive	hsa:5049	D02193
Non-interactive	hsa:5049	D02756
Non-interactive	hsa:5050	D00550
Non-interactive	hsa:5050	D00887
Non-interactive	hsa:5050	D02110
Non-interactive	hsa:5050	D03077
Non-interactive	hsa:5051	D00125
Non-interactive	hsa:5051	D00449
Non-interactive	hsa:5051	D00655
Non-interactive	hsa:5052	D00298
Non-interactive	hsa:5052	D00463
Non-interactive	hsa:5052	D00965
Non-interactive	hsa:5052	D03716
Non-interactive	hsa:5052	D04029
Non-interactive	hsa:5053	D00293
Non-interactive	hsa:5053	D02173
Non-interactive	hsa:5053	D03077
Non-interactive	hsa:5091	D00654
Non-interactive	hsa:5091	D01825
Non-interactive	hsa:50940	D00216
Non-interactive	hsa:50940	D00315
Non-interactive	hsa:50940	D00753
Non-interactive	hsa:50940	D00813
Non-interactive	hsa:50940	D01578
Non-interactive	hsa:50940	D02166
Non-interactive	hsa:50940	D02368
Non-interactive	hsa:50940	D03440
Non-interactive	hsa:50940	D04029
Non-interactive	hsa:5095	D00298
Non-interactive	hsa:5095	D02709
Non-interactive	hsa:5095	D03788
Non-interactive	hsa:5096	D00398
Non-interactive	hsa:5096	D01196
Non-interactive	hsa:5096	D01715
Non-interactive	hsa:50	D00454
Non-interactive	hsa:50	D00622
Non-interactive	hsa:50	D00884
Non-interactive	hsa:50	D01276

Non-interactive	hsa:50	D02487
Non-interactive	hsa:51004	D01119
Non-interactive	hsa:51004	D02193
Non-interactive	hsa:51004	D02671
Non-interactive	hsa:51004	D03798
Non-interactive	hsa:51004	D03826
Non-interactive	hsa:51095	D00434
Non-interactive	hsa:51095	D03728
Non-interactive	hsa:51166	D00391
Non-interactive	hsa:51166	D00567
Non-interactive	hsa:51166	D01582
Non-interactive	hsa:51166	D02193
Non-interactive	hsa:51172	D03720
Non-interactive	hsa:51181	D01061
Non-interactive	hsa:51181	D03716
Non-interactive	hsa:51205	D00454
Non-interactive	hsa:51205	D00887
Non-interactive	hsa:51205	D00900
Non-interactive	hsa:51205	D01974
Non-interactive	hsa:51205	D03899
Non-interactive	hsa:51207	D00203
Non-interactive	hsa:51207	D02698
Non-interactive	hsa:51207	D03710
Non-interactive	hsa:51207	D03798
Non-interactive	hsa:5122	D00298
Non-interactive	hsa:5122	D03788
Non-interactive	hsa:51251	D00188
Non-interactive	hsa:51251	D00622
Non-interactive	hsa:51251	D03350
Non-interactive	hsa:51251	D03805
Non-interactive	hsa:5126	D00188
Non-interactive	hsa:5126	D00364
Non-interactive	hsa:5126	D00538
Non-interactive	hsa:5126	D04024
Non-interactive	hsa:51292	D00142
Non-interactive	hsa:51292	D00325
Non-interactive	hsa:51292	D00537
Non-interactive	hsa:51292	D00620
Non-interactive	hsa:51292	D01715
Non-interactive	hsa:51292	D03440
Non-interactive	hsa:51302	D00516
Non-interactive	hsa:51302	D00593

Non-interactive	hsa:51302	D02487
Non-interactive	hsa:51365	D00188
Non-interactive	hsa:51365	D00294
Non-interactive	hsa:51365	D01885
Non-interactive	hsa:51365	D03440
Non-interactive	hsa:51365	D03716
Non-interactive	hsa:5136	D00002
Non-interactive	hsa:5136	D00148
Non-interactive	hsa:5136	D02173
Non-interactive	hsa:5137	D00125
Non-interactive	hsa:5137	D00188
Non-interactive	hsa:5137	D00567
Non-interactive	hsa:5137	D02258
Non-interactive	hsa:5137	D02368
Non-interactive	hsa:5137	D02769
Non-interactive	hsa:5137	D03828
Non-interactive	hsa:5137	D04024
Non-interactive	hsa:5138	D01211
Non-interactive	hsa:5138	D02115
Non-interactive	hsa:5138	D02441
Non-interactive	hsa:5138	D03765
Non-interactive	hsa:513	D00218
Non-interactive	hsa:513	D00579
Non-interactive	hsa:513	D02756
Non-interactive	hsa:513	D03716
Non-interactive	hsa:513	D03720
Non-interactive	hsa:5140	D00519
Non-interactive	hsa:5141	D03743
Non-interactive	hsa:5142	D00315
Non-interactive	hsa:5142	D00596
Non-interactive	hsa:5142	D03788
Non-interactive	hsa:5143	D00125
Non-interactive	hsa:5143	D00142
Non-interactive	hsa:5143	D00298
Non-interactive	hsa:5143	D01275
Non-interactive	hsa:5143	D02328
Non-interactive	hsa:5143	D03012
Non-interactive	hsa:5143	D03751
Non-interactive	hsa:5144	D02368
Non-interactive	hsa:5144	D02441
Non-interactive	hsa:5144	D03077
Non-interactive	hsa:5144	D03751

Non-interactive	hsa:5144	D03758
Non-interactive	hsa:5144	D03806
Non-interactive	hsa:5144	D03899
Non-interactive	hsa:5145	D00127
Non-interactive	hsa:5145	D01767
Non-interactive	hsa:5145	D02173
Non-interactive	hsa:5145	D02328
Non-interactive	hsa:5145	D02368
Non-interactive	hsa:5145	D03788
Non-interactive	hsa:5146	D00887
Non-interactive	hsa:5146	D02333
Non-interactive	hsa:5147	D00448
Non-interactive	hsa:5147	D00537
Non-interactive	hsa:5147	D00753
Non-interactive	hsa:5147	D01240
Non-interactive	hsa:5147	D01767
Non-interactive	hsa:5147	D02556
Non-interactive	hsa:5147	D03738
Non-interactive	hsa:5147	D03899
Non-interactive	hsa:5147	D04024
Non-interactive	hsa:5148	D00538
Non-interactive	hsa:5148	D00753
Non-interactive	hsa:5148	D01974
Non-interactive	hsa:5149	D00283
Non-interactive	hsa:5149	D00325
Non-interactive	hsa:5149	D00449
Non-interactive	hsa:5149	D01974
Non-interactive	hsa:5149	D02258
Non-interactive	hsa:5149	D03716
Non-interactive	hsa:5149	D03738
Non-interactive	hsa:5150	D00340
Non-interactive	hsa:5150	D00596
Non-interactive	hsa:5150	D01825
Non-interactive	hsa:5150	D02368
Non-interactive	hsa:5151	D01211
Non-interactive	hsa:5151	D02487
Non-interactive	hsa:5151	D03743
Non-interactive	hsa:5151	D03765
Non-interactive	hsa:5151	D03806
Non-interactive	hsa:5152	D00567
Non-interactive	hsa:5152	D01027
Non-interactive	hsa:5152	D01885

Non-interactive	hsa:5152	D02769
Non-interactive	hsa:5152	D03806
Non-interactive	hsa:5156	D00394
Non-interactive	hsa:5156	D00463
Non-interactive	hsa:5156	D00533
Non-interactive	hsa:5156	D02556
Non-interactive	hsa:5156	D03734
Non-interactive	hsa:5158	D00449
Non-interactive	hsa:5158	D01240
Non-interactive	hsa:5158	D02258
Non-interactive	hsa:5158	D04031
Non-interactive	hsa:5159	D00094
Non-interactive	hsa:5159	D00188
Non-interactive	hsa:5159	D02556
Non-interactive	hsa:5159	D03643
Non-interactive	hsa:51645	D00142
Non-interactive	hsa:51645	D00203
Non-interactive	hsa:51645	D00387
Non-interactive	hsa:51645	D02115
Non-interactive	hsa:5167	D01767
Non-interactive	hsa:5168	D00120
Non-interactive	hsa:5168	D00198
Non-interactive	hsa:5168	D00283
Non-interactive	hsa:5168	D00387
Non-interactive	hsa:5168	D00437
Non-interactive	hsa:5168	D00655
Non-interactive	hsa:5168	D01825
Non-interactive	hsa:5168	D03712
Non-interactive	hsa:5168	D03778
Non-interactive	hsa:5169	D00537
Non-interactive	hsa:5169	D01432
Non-interactive	hsa:5169	D01828
Non-interactive	hsa:51727	D00340
Non-interactive	hsa:51727	D00623
Non-interactive	hsa:51727	D03012
Non-interactive	hsa:51727	D03440
Non-interactive	hsa:51727	D04292
Non-interactive	hsa:51	D00753
Non-interactive	hsa:51	D00969
Non-interactive	hsa:51	D01164
Non-interactive	hsa:51	D03738
Non-interactive	hsa:5294	D00364

Non-interactive	hsa:5294	D00434
Non-interactive	hsa:5294	D00805
Non-interactive	hsa:5294	D01211
Non-interactive	hsa:5294	D01276
Non-interactive	hsa:5294	D02418
Non-interactive	hsa:5319	D00364
Non-interactive	hsa:5319	D00567
Non-interactive	hsa:5319	D00771
Non-interactive	hsa:5319	D01911
Non-interactive	hsa:5320	D00654
Non-interactive	hsa:5320	D01578
Non-interactive	hsa:5320	D01866
Non-interactive	hsa:5320	D04025
Non-interactive	hsa:5321	D00900
Non-interactive	hsa:5321	D01915
Non-interactive	hsa:5321	D01984
Non-interactive	hsa:5322	D01164
Non-interactive	hsa:5322	D01397
Non-interactive	hsa:5322	D01911
Non-interactive	hsa:5322	D03077
Non-interactive	hsa:5322	D03899
Non-interactive	hsa:5327	D00538
Non-interactive	hsa:5327	D00567
Non-interactive	hsa:5327	D01842
Non-interactive	hsa:5327	D03012
Non-interactive	hsa:5327	D03728
Non-interactive	hsa:5327	D03734
Non-interactive	hsa:5327	D03775
Non-interactive	hsa:5328	D01064
Non-interactive	hsa:5328	D01844
Non-interactive	hsa:5328	D02581
Non-interactive	hsa:5328	D03012
Non-interactive	hsa:5328	D03806
Non-interactive	hsa:5330	D01715
Non-interactive	hsa:5331	D00437
Non-interactive	hsa:5331	D01432
Non-interactive	hsa:5332	D00654
Non-interactive	hsa:5332	D00884
Non-interactive	hsa:5332	D00889
Non-interactive	hsa:5332	D01974
Non-interactive	hsa:5332	D02671
Non-interactive	hsa:5332	D03803

Non-interactive	hsa:5333	D00437
Non-interactive	hsa:5333	D03751
Non-interactive	hsa:5335	D00002
Non-interactive	hsa:5335	D00139
Non-interactive	hsa:5335	D00324
Non-interactive	hsa:5335	D00593
Non-interactive	hsa:5335	D00733
Non-interactive	hsa:5335	D01180
Non-interactive	hsa:5335	D02214
Non-interactive	hsa:5335	D04029
Non-interactive	hsa:5336	D01256
Non-interactive	hsa:5336	D02487
Non-interactive	hsa:5336	D03826
Non-interactive	hsa:5336	D04025
Non-interactive	hsa:5337	D00656
Non-interactive	hsa:5337	D00781
Non-interactive	hsa:5337	D01027
Non-interactive	hsa:5338	D00198
Non-interactive	hsa:5338	D01367
Non-interactive	hsa:5340	D00324
Non-interactive	hsa:5340	D00752
Non-interactive	hsa:5340	D00781
Non-interactive	hsa:5340	D01397
Non-interactive	hsa:5340	D01718
Non-interactive	hsa:5340	D03823
Non-interactive	hsa:5351	D00216
Non-interactive	hsa:5351	D00455
Non-interactive	hsa:5351	D00623
Non-interactive	hsa:5351	D00655
Non-interactive	hsa:5351	D02110
Non-interactive	hsa:5351	D02698
Non-interactive	hsa:53938	D00148
Non-interactive	hsa:53938	D00325
Non-interactive	hsa:53938	D00394
Non-interactive	hsa:53938	D00596
Non-interactive	hsa:53938	D00650
Non-interactive	hsa:53938	D03828
Non-interactive	hsa:53	D02441
Non-interactive	hsa:53	D02560
Non-interactive	hsa:53	D03741
Non-interactive	hsa:53	D03751
Non-interactive	hsa:5406	D00018

Non-interactive	hsa:5406	D00285
Non-interactive	hsa:5406	D00325
Non-interactive	hsa:5406	D01582
Non-interactive	hsa:5406	D01767
Non-interactive	hsa:5406	D01907
Non-interactive	hsa:5407	D00515
Non-interactive	hsa:5407	D02581
Non-interactive	hsa:5408	D01332
Non-interactive	hsa:5422	D01061
Non-interactive	hsa:5422	D01441
Non-interactive	hsa:5422	D01977
Non-interactive	hsa:5423	D00208
Non-interactive	hsa:5423	D00771
Non-interactive	hsa:5423	D01256
Non-interactive	hsa:5423	D03716
Non-interactive	hsa:5423	D03798
Non-interactive	hsa:5444	D00488
Non-interactive	hsa:5444	D00596
Non-interactive	hsa:5444	D03798
Non-interactive	hsa:5445	D02328
Non-interactive	hsa:5447	D00002
Non-interactive	hsa:5447	D00434
Non-interactive	hsa:5447	D00652
Non-interactive	hsa:5447	D01240
Non-interactive	hsa:5447	D02375
Non-interactive	hsa:5447	D04292
Non-interactive	hsa:54490	D00141
Non-interactive	hsa:54490	D00294
Non-interactive	hsa:54490	D03734
Non-interactive	hsa:54490	D04031
Non-interactive	hsa:54575	D00455
Non-interactive	hsa:54575	D00733
Non-interactive	hsa:54575	D01275
Non-interactive	hsa:54575	D02166
Non-interactive	hsa:54576	D00391
Non-interactive	hsa:54576	D00567
Non-interactive	hsa:54576	D00652
Non-interactive	hsa:54576	D02418
Non-interactive	hsa:54577	D00225
Non-interactive	hsa:54577	D00887
Non-interactive	hsa:54577	D00889
Non-interactive	hsa:54577	D02110

Non-interactive	hsa:54577	D03012
Non-interactive	hsa:54578	D00294
Non-interactive	hsa:54578	D03767
Non-interactive	hsa:54578	D04292
Non-interactive	hsa:54579	D03798
Non-interactive	hsa:54579	D03803
Non-interactive	hsa:54579	D03828
Non-interactive	hsa:54600	D00488
Non-interactive	hsa:54600	D02173
Non-interactive	hsa:54657	D01688
Non-interactive	hsa:54657	D01984
Non-interactive	hsa:54657	D02441
Non-interactive	hsa:54657	D02556
Non-interactive	hsa:54657	D02580
Non-interactive	hsa:54658	D00449
Non-interactive	hsa:54659	D00018
Non-interactive	hsa:54659	D00070
Non-interactive	hsa:54659	D00293
Non-interactive	hsa:54659	D02168
Non-interactive	hsa:54659	D02671
Non-interactive	hsa:54659	D02756
Non-interactive	hsa:54677	D00359
Non-interactive	hsa:54677	D00892
Non-interactive	hsa:54677	D00893
Non-interactive	hsa:5470	D00070
Non-interactive	hsa:5470	D00294
Non-interactive	hsa:5470	D00387
Non-interactive	hsa:5470	D03720
Non-interactive	hsa:5475	D00328
Non-interactive	hsa:5475	D00652
Non-interactive	hsa:5475	D00884
Non-interactive	hsa:5475	D01064
Non-interactive	hsa:5475	D01240
Non-interactive	hsa:5475	D03689
Non-interactive	hsa:5476	D00216
Non-interactive	hsa:5476	D00550
Non-interactive	hsa:5476	D03440
Non-interactive	hsa:5478	D00131
Non-interactive	hsa:5478	D01061
Non-interactive	hsa:5479	D00005
Non-interactive	hsa:5479	D00753
Non-interactive	hsa:5479	D00969

Non-interactive	hsa:5479	D02556
Non-interactive	hsa:5479	D03077
Non-interactive	hsa:5481	D00394
Non-interactive	hsa:5481	D00550
Non-interactive	hsa:5481	D00563
Non-interactive	hsa:5481	D02110
Non-interactive	hsa:5481	D02168
Non-interactive	hsa:5481	D02368
Non-interactive	hsa:5481	D03738
Non-interactive	hsa:54878	D01765
Non-interactive	hsa:54878	D02580
Non-interactive	hsa:54878	D03643
Non-interactive	hsa:54878	D03767
Non-interactive	hsa:54878	D03775
Non-interactive	hsa:5494	D00002
Non-interactive	hsa:5494	D00094
Non-interactive	hsa:5494	D01119
Non-interactive	hsa:5494	D01825
Non-interactive	hsa:5495	D00125
Non-interactive	hsa:5495	D00203
Non-interactive	hsa:5495	D00650
Non-interactive	hsa:5495	D02451
Non-interactive	hsa:5495	D03734
Non-interactive	hsa:5496	D00538
Non-interactive	hsa:5496	D00654
Non-interactive	hsa:5496	D02333
Non-interactive	hsa:5496	D03643
Non-interactive	hsa:5496	D03798
Non-interactive	hsa:5499	D00141
Non-interactive	hsa:5499	D01180
Non-interactive	hsa:5499	D01718
Non-interactive	hsa:5499	D02166
Non-interactive	hsa:54	D02580
Non-interactive	hsa:54	D04025
Non-interactive	hsa:5500	D00394
Non-interactive	hsa:5500	D00771
Non-interactive	hsa:5500	D01688
Non-interactive	hsa:5500	D01718
Non-interactive	hsa:5500	D02560
Non-interactive	hsa:5500	D03767
Non-interactive	hsa:5501	D00127
Non-interactive	hsa:5501	D00141

Non-interactive	hsa:5501	D03736
Non-interactive	hsa:5501	D03775
Non-interactive	hsa:5515	D02229
Non-interactive	hsa:5515	D03788
Non-interactive	hsa:5516	D00315
Non-interactive	hsa:5516	D02166
Non-interactive	hsa:5523	D00620
Non-interactive	hsa:5523	D00653
Non-interactive	hsa:5523	D02229
Non-interactive	hsa:5523	D03728
Non-interactive	hsa:5530	D00203
Non-interactive	hsa:5530	D00596
Non-interactive	hsa:5530	D00965
Non-interactive	hsa:55312	D00298
Non-interactive	hsa:55312	D00651
Non-interactive	hsa:55312	D02328
Non-interactive	hsa:55312	D03735
Non-interactive	hsa:5531	D00198
Non-interactive	hsa:5531	D00216
Non-interactive	hsa:5531	D00298
Non-interactive	hsa:5531	D01196
Non-interactive	hsa:5531	D02173
Non-interactive	hsa:5531	D03805
Non-interactive	hsa:5532	D00198
Non-interactive	hsa:5532	D00225
Non-interactive	hsa:5532	D00391
Non-interactive	hsa:5532	D00434
Non-interactive	hsa:5532	D00622
Non-interactive	hsa:5532	D00623
Non-interactive	hsa:5532	D02769
Non-interactive	hsa:5532	D03775
Non-interactive	hsa:5533	D00196
Non-interactive	hsa:5533	D00884
Non-interactive	hsa:5533	D01397
Non-interactive	hsa:5533	D02333
Non-interactive	hsa:55359	D00293
Non-interactive	hsa:55359	D00656
Non-interactive	hsa:55359	D00965
Non-interactive	hsa:55359	D01842
Non-interactive	hsa:5536	D00394
Non-interactive	hsa:5536	D01718
Non-interactive	hsa:5537	D00005

Non-interactive	hsa:5537	D00188
Non-interactive	hsa:5537	D00328
Non-interactive	hsa:5537	D01276
Non-interactive	hsa:5537	D01842
Non-interactive	hsa:5538	D00538
Non-interactive	hsa:5538	D00969
Non-interactive	hsa:5538	D01180
Non-interactive	hsa:5538	D02487
Non-interactive	hsa:5538	D03803
Non-interactive	hsa:5547	D00283
Non-interactive	hsa:5547	D00394
Non-interactive	hsa:5547	D01977
Non-interactive	hsa:5547	D02556
Non-interactive	hsa:5547	D02655
Non-interactive	hsa:5547	D03440
Non-interactive	hsa:5547	D04024
Non-interactive	hsa:5550	D00018
Non-interactive	hsa:5550	D00437
Non-interactive	hsa:5550	D00538
Non-interactive	hsa:5550	D02193
Non-interactive	hsa:55512	D00398
Non-interactive	hsa:55512	D03738
Non-interactive	hsa:5562	D00148
Non-interactive	hsa:5562	D00530
Non-interactive	hsa:5562	D01256
Non-interactive	hsa:5562	D02487
Non-interactive	hsa:55775	D00196
Non-interactive	hsa:55775	D01915
Non-interactive	hsa:55775	D03710
Non-interactive	hsa:55775	D03823
Non-interactive	hsa:55811	D00387
Non-interactive	hsa:55811	D01240
Non-interactive	hsa:55811	D03803
Non-interactive	hsa:55811	D03805
Non-interactive	hsa:558	D03440
Non-interactive	hsa:558	D03712
Non-interactive	hsa:558	D03743
Non-interactive	hsa:55902	D00455
Non-interactive	hsa:55902	D01885
Non-interactive	hsa:55902	D02110
Non-interactive	hsa:55902	D02560
Non-interactive	hsa:55902	D03218

Non-interactive	hsa:55902	D03899
Non-interactive	hsa:5594	D01256
Non-interactive	hsa:5594	D01582
Non-interactive	hsa:5595	D00733
Non-interactive	hsa:5595	D02229
Non-interactive	hsa:5595	D03728
Non-interactive	hsa:5596	D00203
Non-interactive	hsa:5596	D00364
Non-interactive	hsa:5596	D00965
Non-interactive	hsa:5596	D01240
Non-interactive	hsa:5597	D00148
Non-interactive	hsa:5597	D01275
Non-interactive	hsa:5598	D02258
Non-interactive	hsa:5598	D03738
Non-interactive	hsa:5598	D03778
Non-interactive	hsa:5599	D00294
Non-interactive	hsa:5599	D00623
Non-interactive	hsa:55	D00550
Non-interactive	hsa:55	D00892
Non-interactive	hsa:55	D01765
Non-interactive	hsa:55	D03823
Non-interactive	hsa:5600	D00364
Non-interactive	hsa:5600	D00455
Non-interactive	hsa:5600	D01582
Non-interactive	hsa:5600	D02168
Non-interactive	hsa:5600	D02441
Non-interactive	hsa:5601	D00563
Non-interactive	hsa:5601	D00902
Non-interactive	hsa:5601	D02110
Non-interactive	hsa:5601	D02229
Non-interactive	hsa:5601	D03077
Non-interactive	hsa:5601	D03712
Non-interactive	hsa:5602	D00359
Non-interactive	hsa:5602	D00652
Non-interactive	hsa:5602	D01397
Non-interactive	hsa:5602	D02368
Non-interactive	hsa:5602	D02655
Non-interactive	hsa:5603	D00455
Non-interactive	hsa:5603	D01582
Non-interactive	hsa:5603	D02655
Non-interactive	hsa:5603	D02731
Non-interactive	hsa:5604	D00293

Non-interactive	hsa:5604	D00394
Non-interactive	hsa:5604	D00596
Non-interactive	hsa:5604	D00947
Non-interactive	hsa:5604	D01370
Non-interactive	hsa:5604	D02580
Non-interactive	hsa:5604	D03736
Non-interactive	hsa:5606	D00656
Non-interactive	hsa:5606	D03758
Non-interactive	hsa:5607	D00454
Non-interactive	hsa:5607	D00563
Non-interactive	hsa:5607	D01918
Non-interactive	hsa:5607	D02655
Non-interactive	hsa:5607	D04029
Non-interactive	hsa:5608	D00139
Non-interactive	hsa:5608	D00423
Non-interactive	hsa:5608	D00593
Non-interactive	hsa:5608	D00781
Non-interactive	hsa:5608	D02173
Non-interactive	hsa:5624	D00131
Non-interactive	hsa:5624	D00196
Non-interactive	hsa:5624	D01715
Non-interactive	hsa:5625	D00325
Non-interactive	hsa:5625	D00454
Non-interactive	hsa:5625	D00488
Non-interactive	hsa:5625	D01133
Non-interactive	hsa:5625	D02168
Non-interactive	hsa:5625	D03806
Non-interactive	hsa:5645	D03778
Non-interactive	hsa:5646	D01064
Non-interactive	hsa:5650	D00203
Non-interactive	hsa:5650	D00448
Non-interactive	hsa:5650	D00449
Non-interactive	hsa:5650	D00455
Non-interactive	hsa:5650	D00519
Non-interactive	hsa:5650	D00733
Non-interactive	hsa:5650	D01275
Non-interactive	hsa:5650	D02368
Non-interactive	hsa:5651	D00449
Non-interactive	hsa:5651	D00516
Non-interactive	hsa:5657	D00315
Non-interactive	hsa:5657	D00593
Non-interactive	hsa:5657	D00885

Non-interactive	hsa:5657	D01133
Non-interactive	hsa:5657	D01842
Non-interactive	hsa:5657	D02258
Non-interactive	hsa:5657	D03741
Non-interactive	hsa:56922	D00155
Non-interactive	hsa:56922	D00203
Non-interactive	hsa:56922	D00455
Non-interactive	hsa:56922	D00563
Non-interactive	hsa:56922	D00902
Non-interactive	hsa:56922	D01064
Non-interactive	hsa:56922	D01718
Non-interactive	hsa:56922	D02556
Non-interactive	hsa:56922	D02698
Non-interactive	hsa:57016	D00965
Non-interactive	hsa:57016	D01984
Non-interactive	hsa:57016	D03751
Non-interactive	hsa:57176	D00516
Non-interactive	hsa:57176	D00653
Non-interactive	hsa:57176	D01211
Non-interactive	hsa:57176	D02110
Non-interactive	hsa:57176	D02671
Non-interactive	hsa:5740	D00434
Non-interactive	hsa:5740	D00449
Non-interactive	hsa:5740	D03765
Non-interactive	hsa:5740	D04024
Non-interactive	hsa:5740	D04025
Non-interactive	hsa:5742	D00018
Non-interactive	hsa:5742	D00139
Non-interactive	hsa:5742	D00187
Non-interactive	hsa:5742	D01984
Non-interactive	hsa:5743	D00094
Non-interactive	hsa:5743	D00630
Non-interactive	hsa:5743	D01180
Non-interactive	hsa:5743	D04025
Non-interactive	hsa:5747	D00567
Non-interactive	hsa:5747	D02008
Non-interactive	hsa:5754	D02487
Non-interactive	hsa:5754	D02560
Non-interactive	hsa:57665	D00283
Non-interactive	hsa:57665	D00325
Non-interactive	hsa:57665	D00394
Non-interactive	hsa:57665	D00623

Non-interactive	hsa:57665	D01332
Non-interactive	hsa:57665	D02560
Non-interactive	hsa:57665	D03012
Non-interactive	hsa:57665	D03765
Non-interactive	hsa:58190	D00900
Non-interactive	hsa:58190	D03751
Non-interactive	hsa:5831	D00120
Non-interactive	hsa:5831	D00298
Non-interactive	hsa:5831	D00448
Non-interactive	hsa:5831	D00455
Non-interactive	hsa:5831	D01718
Non-interactive	hsa:5834	D00208
Non-interactive	hsa:5834	D00771
Non-interactive	hsa:5834	D00900
Non-interactive	hsa:5836	D00094
Non-interactive	hsa:5836	D00550
Non-interactive	hsa:5836	D00622
Non-interactive	hsa:5836	D00813
Non-interactive	hsa:5836	D01332
Non-interactive	hsa:5836	D02194
Non-interactive	hsa:5837	D00094
Non-interactive	hsa:5837	D01027
Non-interactive	hsa:5837	D03816
Non-interactive	hsa:5837	D03828
Non-interactive	hsa:5837	D03899
Non-interactive	hsa:5860	D00294
Non-interactive	hsa:5860	D00448
Non-interactive	hsa:5860	D01582
Non-interactive	hsa:5860	D01977
Non-interactive	hsa:5860	D02214
Non-interactive	hsa:5860	D03741
Non-interactive	hsa:586	D01276
Non-interactive	hsa:586	D01973
Non-interactive	hsa:586	D04031
Non-interactive	hsa:587	D00002
Non-interactive	hsa:587	D00252
Non-interactive	hsa:587	D00294
Non-interactive	hsa:587	D00650
Non-interactive	hsa:590	D01027
Non-interactive	hsa:590	D01918
Non-interactive	hsa:590	D02671
Non-interactive	hsa:590	D03734

Non-interactive	hsa:5972	D00463
Non-interactive	hsa:5972	D01915
Non-interactive	hsa:5972	D02110
Non-interactive	hsa:5972	D02166
Non-interactive	hsa:5972	D03758
Non-interactive	hsa:5979	D00198
Non-interactive	hsa:5979	D01690
Non-interactive	hsa:5979	D01974
Non-interactive	hsa:5979	D03738
Non-interactive	hsa:6098	D00216
Non-interactive	hsa:6098	D00969
Non-interactive	hsa:6098	D01061
Non-interactive	hsa:6098	D02229
Non-interactive	hsa:6098	D04029
Non-interactive	hsa:613	D00892
Non-interactive	hsa:6240	D00218
Non-interactive	hsa:6240	D01844
Non-interactive	hsa:6240	D01866
Non-interactive	hsa:6240	D01977
Non-interactive	hsa:6240	D02194
Non-interactive	hsa:6241	D00449
Non-interactive	hsa:6241	D00630
Non-interactive	hsa:6241	D00733
Non-interactive	hsa:6241	D01688
Non-interactive	hsa:6241	D01825
Non-interactive	hsa:6241	D01907
Non-interactive	hsa:6241	D01977
Non-interactive	hsa:6241	D03716
Non-interactive	hsa:6259	D03012
Non-interactive	hsa:6259	D03689
Non-interactive	hsa:6300	D01690
Non-interactive	hsa:6300	D01974
Non-interactive	hsa:63036	D00142
Non-interactive	hsa:63036	D00596
Non-interactive	hsa:63036	D01885
Non-interactive	hsa:63036	D02451
Non-interactive	hsa:63036	D02709
Non-interactive	hsa:63036	D03720
Non-interactive	hsa:635	D00394
Non-interactive	hsa:635	D01240
Non-interactive	hsa:635	D02008
Non-interactive	hsa:635	D03805

Non-interactive	hsa:63904	D00622
Non-interactive	hsa:64087	D00155
Non-interactive	hsa:64087	D00188
Non-interactive	hsa:64087	D00550
Non-interactive	hsa:64087	D00655
Non-interactive	hsa:64087	D00733
Non-interactive	hsa:64087	D01061
Non-interactive	hsa:64087	D02173
Non-interactive	hsa:64087	D03218
Non-interactive	hsa:64087	D03643
Non-interactive	hsa:6416	D00294
Non-interactive	hsa:6416	D00359
Non-interactive	hsa:6416	D00488
Non-interactive	hsa:6416	D00593
Non-interactive	hsa:6416	D00900
Non-interactive	hsa:6416	D01196
Non-interactive	hsa:6416	D03689
Non-interactive	hsa:6416	D03767
Non-interactive	hsa:645	D00131
Non-interactive	hsa:645	D01973
Non-interactive	hsa:645	D03735
Non-interactive	hsa:645	D03775
Non-interactive	hsa:64600	D00018
Non-interactive	hsa:64600	D00094
Non-interactive	hsa:64600	D00142
Non-interactive	hsa:64600	D00887
Non-interactive	hsa:64600	D02115
Non-interactive	hsa:64600	D02368
Non-interactive	hsa:64802	D00887
Non-interactive	hsa:64802	D01866
Non-interactive	hsa:64802	D02581
Non-interactive	hsa:64802	D02655
Non-interactive	hsa:64816	D01718
Non-interactive	hsa:64850	D00141
Non-interactive	hsa:64850	D00198
Non-interactive	hsa:64850	D01844
Non-interactive	hsa:64850	D01907
Non-interactive	hsa:64850	D03440
Non-interactive	hsa:64850	D03689
Non-interactive	hsa:64902	D00120
Non-interactive	hsa:64902	D00325
Non-interactive	hsa:64902	D00488

Non-interactive	hsa:64902	D00969
Non-interactive	hsa:64902	D01027
Non-interactive	hsa:64902	D01918
Non-interactive	hsa:64902	D02333
Non-interactive	hsa:657	D01578
Non-interactive	hsa:658	D00652
Non-interactive	hsa:658	D00658
Non-interactive	hsa:658	D03803
Non-interactive	hsa:6609	D00752
Non-interactive	hsa:6609	D01885
Non-interactive	hsa:6609	D01915
Non-interactive	hsa:660	D00359
Non-interactive	hsa:660	D00563
Non-interactive	hsa:660	D02193
Non-interactive	hsa:660	D03689
Non-interactive	hsa:6610	D01825
Non-interactive	hsa:6646	D00139
Non-interactive	hsa:6646	D00893
Non-interactive	hsa:6652	D00018
Non-interactive	hsa:6652	D00203
Non-interactive	hsa:6652	D00394
Non-interactive	hsa:6652	D00658
Non-interactive	hsa:6652	D01211
Non-interactive	hsa:6652	D01432
Non-interactive	hsa:6652	D01866
Non-interactive	hsa:6652	D01918
Non-interactive	hsa:6652	D02581
Non-interactive	hsa:670	D00148
Non-interactive	hsa:670	D01264
Non-interactive	hsa:6713	D01256
Non-interactive	hsa:6713	D02008
Non-interactive	hsa:6713	D02333
Non-interactive	hsa:6713	D03350
Non-interactive	hsa:6716	D00324
Non-interactive	hsa:6716	D03767
Non-interactive	hsa:6716	D04024
Non-interactive	hsa:6725	D01918
Non-interactive	hsa:6725	D02698
Non-interactive	hsa:6725	D03765
Non-interactive	hsa:6725	D03816
Non-interactive	hsa:6725	D03882
Non-interactive	hsa:6725	D04024

Non-interactive	hsa:6768	D00294
Non-interactive	hsa:6768	D00387
Non-interactive	hsa:6768	D00651
Non-interactive	hsa:6768	D00781
Non-interactive	hsa:6768	D01582
Non-interactive	hsa:6768	D01828
Non-interactive	hsa:6768	D03077
Non-interactive	hsa:6768	D03767
Non-interactive	hsa:6799	D00148
Non-interactive	hsa:6799	D00651
Non-interactive	hsa:6799	D01133
Non-interactive	hsa:6799	D02115
Non-interactive	hsa:6799	D03816
Non-interactive	hsa:683	D00884
Non-interactive	hsa:683	D01885
Non-interactive	hsa:686	D00070
Non-interactive	hsa:686	D00120
Non-interactive	hsa:686	D00148
Non-interactive	hsa:686	D00285
Non-interactive	hsa:686	D00650
Non-interactive	hsa:686	D01027
Non-interactive	hsa:686	D02008
Non-interactive	hsa:686	D03689
Non-interactive	hsa:6897	D00139
Non-interactive	hsa:6897	D00148
Non-interactive	hsa:6897	D00208
Non-interactive	hsa:6897	D00550
Non-interactive	hsa:6897	D01196
Non-interactive	hsa:6897	D02368
Non-interactive	hsa:6897	D02580
Non-interactive	hsa:6898	D00394
Non-interactive	hsa:6898	D00652
Non-interactive	hsa:6898	D00654
Non-interactive	hsa:6898	D02375
Non-interactive	hsa:6898	D03823
Non-interactive	hsa:6898	D03882
Non-interactive	hsa:695	D00650
Non-interactive	hsa:695	D00781
Non-interactive	hsa:695	D01715
Non-interactive	hsa:7006	D02333
Non-interactive	hsa:7010	D00002
Non-interactive	hsa:7010	D00293

Non-interactive	hsa:7010	D00753
Non-interactive	hsa:7010	D00885
Non-interactive	hsa:7010	D00902
Non-interactive	hsa:7015	D00018
Non-interactive	hsa:7015	D00107
Non-interactive	hsa:7015	D00252
Non-interactive	hsa:7015	D00423
Non-interactive	hsa:7015	D01264
Non-interactive	hsa:7015	D02655
Non-interactive	hsa:7015	D03734
Non-interactive	hsa:7046	D00094
Non-interactive	hsa:7046	D00567
Non-interactive	hsa:7046	D00884
Non-interactive	hsa:7046	D00889
Non-interactive	hsa:7046	D03803
Non-interactive	hsa:7054	D00394
Non-interactive	hsa:7054	D00434
Non-interactive	hsa:7075	D02115
Non-interactive	hsa:7075	D02173
Non-interactive	hsa:7075	D02451
Non-interactive	hsa:7084	D00125
Non-interactive	hsa:7084	D00324
Non-interactive	hsa:7084	D00463
Non-interactive	hsa:7084	D00887
Non-interactive	hsa:7084	D01915
Non-interactive	hsa:7084	D02110
Non-interactive	hsa:7150	D00107
Non-interactive	hsa:7150	D00225
Non-interactive	hsa:7150	D00653
Non-interactive	hsa:7150	D01256
Non-interactive	hsa:7150	D01828
Non-interactive	hsa:7150	D01974
Non-interactive	hsa:7150	D04292
Non-interactive	hsa:7153	D00653
Non-interactive	hsa:7155	D00963
Non-interactive	hsa:7155	D01180
Non-interactive	hsa:7155	D01842
Non-interactive	hsa:7155	D01984
Non-interactive	hsa:7155	D02581
Non-interactive	hsa:7156	D00620
Non-interactive	hsa:7156	D00884
Non-interactive	hsa:7156	D02580

Non-interactive	hsa:7172	D00652
Non-interactive	hsa:7173	D00963
Non-interactive	hsa:7173	D01133
Non-interactive	hsa:7173	D03798
Non-interactive	hsa:7174	D00208
Non-interactive	hsa:7174	D00394
Non-interactive	hsa:7174	D00658
Non-interactive	hsa:7174	D00885
Non-interactive	hsa:7174	D01918
Non-interactive	hsa:7294	D00188
Non-interactive	hsa:7294	D00781
Non-interactive	hsa:7294	D01180
Non-interactive	hsa:7294	D01973
Non-interactive	hsa:7294	D03218
Non-interactive	hsa:7294	D03743
Non-interactive	hsa:7297	D02333
Non-interactive	hsa:7298	D00225
Non-interactive	hsa:7298	D00434
Non-interactive	hsa:7298	D00455
Non-interactive	hsa:7299	D00325
Non-interactive	hsa:7299	D00651
Non-interactive	hsa:7299	D01825
Non-interactive	hsa:7299	D01862
Non-interactive	hsa:7299	D01885
Non-interactive	hsa:7301	D00324
Non-interactive	hsa:7301	D01223
Non-interactive	hsa:7301	D01565
Non-interactive	hsa:7301	D03643
Non-interactive	hsa:7301	D03738
Non-interactive	hsa:7363	D03218
Non-interactive	hsa:7364	D00216
Non-interactive	hsa:7364	D00889
Non-interactive	hsa:7364	D02375
Non-interactive	hsa:7365	D00120
Non-interactive	hsa:7365	D00567
Non-interactive	hsa:7365	D00884
Non-interactive	hsa:7365	D03689
Non-interactive	hsa:7366	D00630
Non-interactive	hsa:7366	D01842
Non-interactive	hsa:7366	D02368
Non-interactive	hsa:7367	D00187
Non-interactive	hsa:7367	D00563

Non-interactive	hsa:7367	D00733
Non-interactive	hsa:7367	D00884
Non-interactive	hsa:7367	D03788
Non-interactive	hsa:7371	D00463
Non-interactive	hsa:7371	D03350
Non-interactive	hsa:7371	D03758
Non-interactive	hsa:7372	D00107
Non-interactive	hsa:7372	D00148
Non-interactive	hsa:7372	D00423
Non-interactive	hsa:7372	D01119
Non-interactive	hsa:7372	D01718
Non-interactive	hsa:7372	D02173
Non-interactive	hsa:7372	D04024
Non-interactive	hsa:7378	D00753
Non-interactive	hsa:7378	D02258
Non-interactive	hsa:7453	D00018
Non-interactive	hsa:7453	D00120
Non-interactive	hsa:7453	D00187
Non-interactive	hsa:7453	D00969
Non-interactive	hsa:7453	D01256
Non-interactive	hsa:7453	D02258
Non-interactive	hsa:7453	D03882
Non-interactive	hsa:7498	D00107
Non-interactive	hsa:7498	D00142
Non-interactive	hsa:7498	D00324
Non-interactive	hsa:7498	D03728
Non-interactive	hsa:7498	D03743
Non-interactive	hsa:7498	D04031
Non-interactive	hsa:7525	D00293
Non-interactive	hsa:7525	D01240
Non-interactive	hsa:7525	D02194
Non-interactive	hsa:7525	D03077
Non-interactive	hsa:7525	D03218
Non-interactive	hsa:7535	D00155
Non-interactive	hsa:7535	D00298
Non-interactive	hsa:7535	D00658
Non-interactive	hsa:7535	D00887
Non-interactive	hsa:7535	D00965
Non-interactive	hsa:7535	D02581
Non-interactive	hsa:759	D00516
Non-interactive	hsa:759	D00893
Non-interactive	hsa:759	D02115

Non-interactive	hsa:760	D00423
Non-interactive	hsa:760	D01907
Non-interactive	hsa:760	D01977
Non-interactive	hsa:760	D02042
Non-interactive	hsa:761	D00198
Non-interactive	hsa:761	D00813
Non-interactive	hsa:761	D00889
Non-interactive	hsa:761	D00963
Non-interactive	hsa:762	D00813
Non-interactive	hsa:762	D01690
Non-interactive	hsa:762	D03823
Non-interactive	hsa:762	D04031
Non-interactive	hsa:763	D00142
Non-interactive	hsa:763	D00203
Non-interactive	hsa:763	D00298
Non-interactive	hsa:763	D02487
Non-interactive	hsa:765	D00771
Non-interactive	hsa:765	D01767
Non-interactive	hsa:765	D03826
Non-interactive	hsa:766	D00437
Non-interactive	hsa:766	D00537
Non-interactive	hsa:766	D01276
Non-interactive	hsa:766	D01718
Non-interactive	hsa:766	D03710
Non-interactive	hsa:766	D03720
Non-interactive	hsa:767	D00148
Non-interactive	hsa:767	D01332
Non-interactive	hsa:767	D02756
Non-interactive	hsa:767	D03643
Non-interactive	hsa:767	D03689
Non-interactive	hsa:767	D03806
Non-interactive	hsa:768	D01240
Non-interactive	hsa:768	D02110
Non-interactive	hsa:771	D00216
Non-interactive	hsa:771	D00455
Non-interactive	hsa:771	D00533
Non-interactive	hsa:771	D00887
Non-interactive	hsa:771	D03741
Non-interactive	hsa:780	D00434
Non-interactive	hsa:780	D00448
Non-interactive	hsa:780	D01256
Non-interactive	hsa:780	D03826

Non-interactive	hsa:79001	D00125
Non-interactive	hsa:79001	D00652
Non-interactive	hsa:79001	D01133
Non-interactive	hsa:79001	D01907
Non-interactive	hsa:79001	D03643
Non-interactive	hsa:7957	D00315
Non-interactive	hsa:7957	D00519
Non-interactive	hsa:7957	D00623
Non-interactive	hsa:7957	D00753
Non-interactive	hsa:7957	D01367
Non-interactive	hsa:79799	D00285
Non-interactive	hsa:79799	D00771
Non-interactive	hsa:79799	D01180
Non-interactive	hsa:79799	D01441
Non-interactive	hsa:80339	D00359
Non-interactive	hsa:80339	D00653
Non-interactive	hsa:80339	D00658
Non-interactive	hsa:80339	D03720
Non-interactive	hsa:80339	D03816
Non-interactive	hsa:80339	D03823
Non-interactive	hsa:80824	D00454
Non-interactive	hsa:80824	D01690
Non-interactive	hsa:81579	D00893
Non-interactive	hsa:81579	D00902
Non-interactive	hsa:81579	D01866
Non-interactive	hsa:81579	D02194
Non-interactive	hsa:81579	D03775
Non-interactive	hsa:8192	D00620
Non-interactive	hsa:8192	D01223
Non-interactive	hsa:8192	D01911
Non-interactive	hsa:8192	D03735
Non-interactive	hsa:8192	D03751
Non-interactive	hsa:8192	D03823
Non-interactive	hsa:8288	D00283
Non-interactive	hsa:8288	D01027
Non-interactive	hsa:8288	D02115
Non-interactive	hsa:8288	D03751
Non-interactive	hsa:834	D00283
Non-interactive	hsa:834	D00516
Non-interactive	hsa:834	D01397
Non-interactive	hsa:834	D03828
Non-interactive	hsa:8398	D00771

Non-interactive	hsa:8398	D01565
Non-interactive	hsa:8398	D01984
Non-interactive	hsa:8398	D02115
Non-interactive	hsa:84152	D00437
Non-interactive	hsa:84152	D01071
Non-interactive	hsa:84152	D02581
Non-interactive	hsa:84152	D03767
Non-interactive	hsa:84152	D03828
Non-interactive	hsa:84171	D00387
Non-interactive	hsa:84171	D02008
Non-interactive	hsa:84171	D02709
Non-interactive	hsa:84171	D03788
Non-interactive	hsa:8435	D00002
Non-interactive	hsa:8435	D00359
Non-interactive	hsa:8435	D00519
Non-interactive	hsa:8435	D00653
Non-interactive	hsa:8435	D01441
Non-interactive	hsa:8435	D02333
Non-interactive	hsa:8435	D02487
Non-interactive	hsa:84532	D00622
Non-interactive	hsa:84532	D01276
Non-interactive	hsa:84532	D01844
Non-interactive	hsa:84532	D03077
Non-interactive	hsa:84618	D00324
Non-interactive	hsa:84618	D03643
Non-interactive	hsa:84618	D03710
Non-interactive	hsa:84695	D01027
Non-interactive	hsa:84695	D01211
Non-interactive	hsa:84695	D03805
Non-interactive	hsa:84706	D00398
Non-interactive	hsa:84706	D00414
Non-interactive	hsa:84706	D00887
Non-interactive	hsa:84706	D01578
Non-interactive	hsa:84706	D01825
Non-interactive	hsa:84706	D02709
Non-interactive	hsa:84706	D03826
Non-interactive	hsa:84812	D00752
Non-interactive	hsa:84812	D01027
Non-interactive	hsa:84812	D02008
Non-interactive	hsa:84812	D02333
Non-interactive	hsa:84812	D02487
Non-interactive	hsa:8513	D00131

Non-interactive	hsa:8513	D00203
Non-interactive	hsa:8513	D02110
Non-interactive	hsa:8513	D03218
Non-interactive	hsa:8529	D00094
Non-interactive	hsa:8529	D02214
Non-interactive	hsa:8529	D02333
Non-interactive	hsa:8529	D02655
Non-interactive	hsa:8529	D03077
Non-interactive	hsa:8529	D03734
Non-interactive	hsa:85313	D00293
Non-interactive	hsa:85313	D00884
Non-interactive	hsa:85313	D01119
Non-interactive	hsa:85313	D02756
Non-interactive	hsa:85313	D03440
Non-interactive	hsa:8555	D00550
Non-interactive	hsa:8555	D00889
Non-interactive	hsa:8555	D00969
Non-interactive	hsa:8555	D02560
Non-interactive	hsa:8555	D03012
Non-interactive	hsa:8556	D00650
Non-interactive	hsa:8556	D00902
Non-interactive	hsa:8556	D01119
Non-interactive	hsa:8556	D01915
Non-interactive	hsa:8622	D00519
Non-interactive	hsa:8622	D00623
Non-interactive	hsa:8622	D00652
Non-interactive	hsa:8622	D01196
Non-interactive	hsa:8622	D02368
Non-interactive	hsa:8654	D00198
Non-interactive	hsa:8654	D00885
Non-interactive	hsa:8654	D02115
Non-interactive	hsa:8854	D00947
Non-interactive	hsa:8854	D01767
Non-interactive	hsa:8940	D00005
Non-interactive	hsa:8940	D00752
Non-interactive	hsa:8940	D03828
Non-interactive	hsa:8940	D04024
Non-interactive	hsa:8972	D00125
Non-interactive	hsa:8972	D00414
Non-interactive	hsa:8972	D00947
Non-interactive	hsa:8972	D01223
Non-interactive	hsa:8972	D01765

Non-interactive	hsa:8972	D03788
Non-interactive	hsa:9023	D00127
Non-interactive	hsa:9023	D00892
Non-interactive	hsa:9023	D00965
Non-interactive	hsa:9023	D03689
Non-interactive	hsa:9023	D04292
Non-interactive	hsa:9088	D00203
Non-interactive	hsa:9088	D00391
Non-interactive	hsa:9088	D00434
Non-interactive	hsa:9088	D00563
Non-interactive	hsa:9088	D00596
Non-interactive	hsa:90	D00131
Non-interactive	hsa:90	D00196
Non-interactive	hsa:90	D00593
Non-interactive	hsa:90	D01765
Non-interactive	hsa:91039	D00131
Non-interactive	hsa:91039	D01064
Non-interactive	hsa:91039	D02115
Non-interactive	hsa:91039	D02487
Non-interactive	hsa:9150	D00630
Non-interactive	hsa:9150	D01027
Non-interactive	hsa:9150	D01180
Non-interactive	hsa:9150	D01828
Non-interactive	hsa:9150	D02193
Non-interactive	hsa:9150	D02418
Non-interactive	hsa:91	D00216
Non-interactive	hsa:91	D00225
Non-interactive	hsa:91	D01397
Non-interactive	hsa:91	D01688
Non-interactive	hsa:91	D03643
Non-interactive	hsa:91	D03716
Non-interactive	hsa:93650	D00005
Non-interactive	hsa:93650	D00887
Non-interactive	hsa:93650	D01767
Non-interactive	hsa:93650	D02556
Non-interactive	hsa:9388	D00198
Non-interactive	hsa:9388	D00538
Non-interactive	hsa:9388	D00550
Non-interactive	hsa:9388	D01196
Non-interactive	hsa:9388	D03823
Non-interactive	hsa:9388	D04029
Non-interactive	hsa:93	D00018

Non-interactive	hsa:93	D00125
Non-interactive	hsa:93	D00188
Non-interactive	hsa:93	D00550
Non-interactive	hsa:93	D02193
Non-interactive	hsa:93	D03218
Non-interactive	hsa:94009	D00208
Non-interactive	hsa:94009	D02655
Non-interactive	hsa:94009	D03828
Non-interactive	hsa:9420	D00455
Non-interactive	hsa:9420	D00593
Non-interactive	hsa:9420	D03741
Non-interactive	hsa:9420	D03816
Non-interactive	hsa:9420	D04292
Non-interactive	hsa:94	D00530
Non-interactive	hsa:94	D00538
Non-interactive	hsa:94	D00658
Non-interactive	hsa:94	D01276
Non-interactive	hsa:94	D01715
Non-interactive	hsa:94	D01911
Non-interactive	hsa:94	D02556
Non-interactive	hsa:9563	D00139
Non-interactive	hsa:9563	D00155
Non-interactive	hsa:9563	D03738
Non-interactive	hsa:9601	D00142
Non-interactive	hsa:9601	D00294
Non-interactive	hsa:9601	D00359
Non-interactive	hsa:9601	D00463
Non-interactive	hsa:9601	D00538
Non-interactive	hsa:9601	D03720
Non-interactive	hsa:9641	D00139
Non-interactive	hsa:9641	D00142
Non-interactive	hsa:9641	D00198
Non-interactive	hsa:9641	D00391
Non-interactive	hsa:9641	D02333
Non-interactive	hsa:9641	D02418
Non-interactive	hsa:9641	D02756
Non-interactive	hsa:9647	D00218
Non-interactive	hsa:9647	D01918
Non-interactive	hsa:9647	D02731
Non-interactive	hsa:9945	D00530
Non-interactive	hsa:9945	D01578
Non-interactive	hsa:9945	D02042

Non-interactive	hsa:9955	D00125
Non-interactive	hsa:9955	D00203
Non-interactive	hsa:9955	D00805
Non-interactive	hsa:9955	D01133
Non-interactive	hsa:5537	D03720
Non-interactive	hsa:2135	D02375
Non-interactive	hsa:7150	D00434
Non-interactive	hsa:1728	D01027
Non-interactive	hsa:5331	D04024
Non-interactive	hsa:240	D00187
Non-interactive	hsa:51645	D02333
Non-interactive	hsa:5330	D01164
Non-interactive	hsa:3988	D00622
Non-interactive	hsa:2326	D03689
Non-interactive	hsa:5743	D01918
Non-interactive	hsa:7365	D03823
Non-interactive	hsa:51727	D02756
Non-interactive	hsa:5478	D00884
Non-interactive	hsa:79799	D01984
Non-interactive	hsa:1800	D01828
Non-interactive	hsa:2046	D01688
Non-interactive	hsa:5495	D03712
Non-interactive	hsa:5143	D00889
Non-interactive	hsa:3791	D00198
Non-interactive	hsa:1843	D00155
Non-interactive	hsa:1551	D00653
Non-interactive	hsa:6768	D01825
Non-interactive	hsa:6646	D03643
Non-interactive	hsa:4128	D00455
Non-interactive	hsa:150290	D02328
Non-interactive	hsa:5607	D02115
Non-interactive	hsa:6646	D01370
Non-interactive	hsa:5422	D03823
Non-interactive	hsa:1545	D01862
Non-interactive	hsa:5337	D04024
Non-interactive	hsa:6646	D00533
Non-interactive	hsa:586	D03736
Non-interactive	hsa:55811	D02168
Non-interactive	hsa:3643	D00398
Non-interactive	hsa:2534	D00550
Non-interactive	hsa:2066	D00969
Non-interactive	hsa:4593	D00900

Non-interactive	hsa:270	D03882
Non-interactive	hsa:1360	D00002
Non-interactive	hsa:5126	D00325
Non-interactive	hsa:5600	D03689
Non-interactive	hsa:124	D00225
Non-interactive	hsa:5562	D02328
Non-interactive	hsa:10279	D01973
Non-interactive	hsa:3291	D00216
Non-interactive	hsa:9641	D02110
Non-interactive	hsa:8622	D04029
Non-interactive	hsa:1544	D03712
Non-interactive	hsa:5533	D00437
Non-interactive	hsa:613	D00753
Non-interactive	hsa:54578	D01973
Non-interactive	hsa:5515	D04025
Non-interactive	hsa:5331	D00434
Non-interactive	hsa:23436	D00018
Non-interactive	hsa:476	D03218
Non-interactive	hsa:3035	D01973
Non-interactive	hsa:191	D02258
Non-interactive	hsa:5045	D04031
Non-interactive	hsa:7372	D00650
Non-interactive	hsa:1845	D00218
Non-interactive	hsa:3645	D03775
Non-interactive	hsa:5327	D01688
Non-interactive	hsa:2746	D00391
Non-interactive	hsa:1594	D00216
Non-interactive	hsa:1543	D00884
Non-interactive	hsa:2048	D00449
Non-interactive	hsa:10295	D03012
Non-interactive	hsa:11221	D02756
Non-interactive	hsa:4486	D00107
Non-interactive	hsa:2242	D00654
Non-interactive	hsa:2044	D00448
Non-interactive	hsa:3480	D00252
Non-interactive	hsa:6646	D00630
Non-interactive	hsa:5601	D00900
Non-interactive	hsa:51365	D01973
Non-interactive	hsa:3939	D00364
Non-interactive	hsa:57176	D02756
Non-interactive	hsa:126	D03734
Non-interactive	hsa:25	D01240

Non-interactive	hsa:2232	D01918
Non-interactive	hsa:3002	D03775
Non-interactive	hsa:54659	D01974
Non-interactive	hsa:2766	D00885
Non-interactive	hsa:84618	D00391
Non-interactive	hsa:5050	D00892
Non-interactive	hsa:5328	D00533
Non-interactive	hsa:5406	D00293
Non-interactive	hsa:225689	D01578
Non-interactive	hsa:113	D00340
Non-interactive	hsa:11072	D00892
Non-interactive	hsa:1033	D00652
Non-interactive	hsa:2161	D01578
Non-interactive	hsa:25	D03218
Non-interactive	hsa:2066	D02698
Non-interactive	hsa:2242	D01264
Non-interactive	hsa:4921	D03751
Non-interactive	hsa:834	D03798
Non-interactive	hsa:2161	D01765
Non-interactive	hsa:134510	D00519
Non-interactive	hsa:5478	D00120
Non-interactive	hsa:1576	D00391
Non-interactive	hsa:645	D03807
Non-interactive	hsa:2047	D02375
Non-interactive	hsa:2260	D00293
Non-interactive	hsa:1806	D00463
Non-interactive	hsa:5747	D01565
Non-interactive	hsa:115	D00125
Non-interactive	hsa:2264	D02333
Non-interactive	hsa:3614	D00139
Non-interactive	hsa:54	D00889
Non-interactive	hsa:4311	D03765
Non-interactive	hsa:7378	D01915
Non-interactive	hsa:51181	D03743
Non-interactive	hsa:4920	D00414
Non-interactive	hsa:2155	D00002
Non-interactive	hsa:7957	D03743
Non-interactive	hsa:1562	D01275
Non-interactive	hsa:11221	D01715
Non-interactive	hsa:247	D00965
Non-interactive	hsa:3292	D00969
Non-interactive	hsa:6725	D03012

Non-interactive	hsa:5747	D00537
Non-interactive	hsa:285220	D00963
Non-interactive	hsa:9945	D01767
Non-interactive	hsa:1589	D00127
Non-interactive	hsa:2044	D00449
Non-interactive	hsa:1551	D01866
Non-interactive	hsa:26279	D01911
Non-interactive	hsa:5836	D00141
Non-interactive	hsa:51727	D02580
Non-interactive	hsa:7006	D00516
Non-interactive	hsa:131	D03734
Non-interactive	hsa:10327	D01441
Non-interactive	hsa:27032	D01688
Non-interactive	hsa:1562	D00294
Non-interactive	hsa:2046	D00969
Non-interactive	hsa:1565	D02194
Non-interactive	hsa:7006	D00805
Non-interactive	hsa:1200	D03778
Non-interactive	hsa:85313	D01276
Non-interactive	hsa:4145	D02042
Non-interactive	hsa:1621	D00423
Non-interactive	hsa:9647	D03806
Non-interactive	hsa:7153	D01027
Non-interactive	hsa:239	D00454
Non-interactive	hsa:50487	D01915
Non-interactive	hsa:7294	D01275
Non-interactive	hsa:2328	D01211
Non-interactive	hsa:2066	D00652
Non-interactive	hsa:5837	D01973
Non-interactive	hsa:6897	D01842
Non-interactive	hsa:5979	D00885
Non-interactive	hsa:5608	D01061
Non-interactive	hsa:5533	D02756
Non-interactive	hsa:4486	D00530
Non-interactive	hsa:4594	D03716
Non-interactive	hsa:57176	D00018
Non-interactive	hsa:767	D00434
Non-interactive	hsa:1728	D03716
Non-interactive	hsa:5143	D01688
Non-interactive	hsa:1033	D00342
Non-interactive	hsa:1432	D01718
Non-interactive	hsa:1675	D02258

Non-interactive	hsa:5550	D01240
Non-interactive	hsa:5625	D03743
Non-interactive	hsa:1595	D03803
Non-interactive	hsa:613	D02375
Non-interactive	hsa:51172	D00208
Non-interactive	hsa:1267	D01180
Non-interactive	hsa:2548	D00567
Non-interactive	hsa:55	D00963
Non-interactive	hsa:5550	D03717
Non-interactive	hsa:4353	D01690
Non-interactive	hsa:8288	D01825
Non-interactive	hsa:11072	D00196
Non-interactive	hsa:56922	D01973
Non-interactive	hsa:10056	D00018
Non-interactive	hsa:34	D00885
Non-interactive	hsa:2746	D04292
Non-interactive	hsa:1544	D00359
Non-interactive	hsa:7083	D00519
Non-interactive	hsa:1549	D03767
Non-interactive	hsa:4837	D01866
Non-interactive	hsa:7957	D01264
Non-interactive	hsa:5601	D00488
Non-interactive	hsa:5608	D00127
Non-interactive	hsa:1553	D00364
Non-interactive	hsa:109	D01565
Non-interactive	hsa:3376	D00654
Non-interactive	hsa:50940	D02451
Non-interactive	hsa:4051	D01718
Non-interactive	hsa:5406	D00449
Non-interactive	hsa:3033	D01276
Non-interactive	hsa:10	D00125
Non-interactive	hsa:51172	D00455
Non-interactive	hsa:10	D00293
Non-interactive	hsa:54579	D00324
Non-interactive	hsa:3791	D03710
Non-interactive	hsa:2936	D02042
Non-interactive	hsa:6610	D00094
Non-interactive	hsa:5122	D00488
Non-interactive	hsa:3416	D03798
Non-interactive	hsa:5979	D00563
Non-interactive	hsa:1178	D00593
Non-interactive	hsa:5051	D00293

Non-interactive	hsa:5052	D01180
Non-interactive	hsa:5608	D00094
Non-interactive	hsa:2050	D01397
Non-interactive	hsa:8940	D00414
Non-interactive	hsa:5091	D03350
Non-interactive	hsa:1844	D03716
Non-interactive	hsa:3906	D00593
Non-interactive	hsa:5167	D02671
Non-interactive	hsa:64816	D01825
Non-interactive	hsa:5144	D00813
Non-interactive	hsa:4329	D02441
Non-interactive	hsa:2261	D00893
Non-interactive	hsa:3067	D00623
Non-interactive	hsa:5516	D00127
Non-interactive	hsa:54	D00391
Non-interactive	hsa:56922	D00094
Non-interactive	hsa:54576	D03734
Non-interactive	hsa:501	D03798
Non-interactive	hsa:30833	D01133
Non-interactive	hsa:5562	D01332
Non-interactive	hsa:81579	D01565
Non-interactive	hsa:1553	D00550
Non-interactive	hsa:766	D00398
Non-interactive	hsa:1847	D00654
Non-interactive	hsa:3815	D02194
Non-interactive	hsa:8555	D01332
Non-interactive	hsa:339221	D01164
Non-interactive	hsa:5476	D00198
Non-interactive	hsa:22843	D01064
Non-interactive	hsa:2321	D02769
Non-interactive	hsa:765	D01397
Non-interactive	hsa:9563	D03710
Non-interactive	hsa:1846	D00656
Non-interactive	hsa:4915	D00969
Non-interactive	hsa:246	D03734
Non-interactive	hsa:30833	D00324
Non-interactive	hsa:3290	D01332
Non-interactive	hsa:613	D01767
Non-interactive	hsa:1847	D02441
Non-interactive	hsa:761	D00515
Non-interactive	hsa:4953	D00900
Non-interactive	hsa:2058	D01061

Non-interactive	hsa:64902	D02698
Non-interactive	hsa:5608	D00969
Non-interactive	hsa:128853	D01061
Non-interactive	hsa:7366	D00567
Non-interactive	hsa:5136	D01027
Non-interactive	hsa:151531	D01370
Non-interactive	hsa:1991	D02375
Non-interactive	hsa:5495	D00225
Non-interactive	hsa:10327	D00805
Non-interactive	hsa:2880	D00593
Non-interactive	hsa:3033	D03767
Non-interactive	hsa:54577	D00651
Non-interactive	hsa:4548	D03728
Non-interactive	hsa:7046	D01977
Non-interactive	hsa:5603	D00208
Non-interactive	hsa:1577	D02214
Non-interactive	hsa:3988	D02258
Non-interactive	hsa:7084	D01164
Non-interactive	hsa:5136	D00203
Non-interactive	hsa:760	D02709
Non-interactive	hsa:3141	D03899
Non-interactive	hsa:2326	D00652
Non-interactive	hsa:1583	D00653
Non-interactive	hsa:834	D00463
Non-interactive	hsa:3480	D02671
Non-interactive	hsa:1572	D00900
Non-interactive	hsa:7153	D00196
Non-interactive	hsa:5595	D01984
Non-interactive	hsa:1579	D00596
Non-interactive	hsa:513	D01582
Non-interactive	hsa:5624	D03823
Non-interactive	hsa:1728	D00434
Non-interactive	hsa:81579	D04025
Non-interactive	hsa:7297	D03689
Non-interactive	hsa:2161	D04031
Non-interactive	hsa:4916	D00414
Non-interactive	hsa:10056	D03788
Non-interactive	hsa:5136	D02756
Non-interactive	hsa:5447	D02655
Non-interactive	hsa:2882	D03882
Non-interactive	hsa:5538	D00651
Non-interactive	hsa:2041	D03805

Non-interactive	hsa:5050	D03012
Non-interactive	hsa:7957	D00434
Non-interactive	hsa:128853	D00651
Non-interactive	hsa:1581	D00218
Non-interactive	hsa:54677	D03743
Non-interactive	hsa:2322	D02487
Non-interactive	hsa:4129	D03741
Non-interactive	hsa:5447	D00070
Non-interactive	hsa:5052	D02441
Non-interactive	hsa:4486	D00658
Non-interactive	hsa:1581	D00652
Non-interactive	hsa:8654	D00449
Non-interactive	hsa:5351	D00340
Non-interactive	hsa:3645	D01907
Non-interactive	hsa:100	D01027
Non-interactive	hsa:9945	D00139
Non-interactive	hsa:55312	D00947
Non-interactive	hsa:54600	D00131
Non-interactive	hsa:3615	D00391
Non-interactive	hsa:238	D02333
Non-interactive	hsa:7006	D04025
Non-interactive	hsa:5598	D00448
Non-interactive	hsa:2766	D02769
Non-interactive	hsa:191	D00887
Non-interactive	hsa:1800	D03767
Non-interactive	hsa:124	D03816
Non-interactive	hsa:1991	D02166
Non-interactive	hsa:1583	D02368
Non-interactive	hsa:35	D01974
Non-interactive	hsa:54677	D01844
Non-interactive	hsa:132	D00139
Non-interactive	hsa:5444	D00885
Non-interactive	hsa:4942	D03738
Non-interactive	hsa:216	D03218
Non-interactive	hsa:5475	D03882
Non-interactive	hsa:217	D00538
Non-interactive	hsa:635	D00538
Non-interactive	hsa:9420	D00070
Non-interactive	hsa:4058	D01367
Non-interactive	hsa:6416	D01828
Non-interactive	hsa:55312	D03736
Non-interactive	hsa:27115	D03716

Non-interactive	hsa:2041	D00107
Non-interactive	hsa:5537	D03218
Non-interactive	hsa:3290	D01370
Non-interactive	hsa:3614	D06238
Non-interactive	hsa:2044	D03899
Non-interactive	hsa:23430	D01974
Non-interactive	hsa:5604	D03823
Non-interactive	hsa:3001	D00550
Non-interactive	hsa:657	D01765
Non-interactive	hsa:5158	D02769
Non-interactive	hsa:93650	D02698
Non-interactive	hsa:1579	D00423
Non-interactive	hsa:9641	D01275
Non-interactive	hsa:1431	D00519
Non-interactive	hsa:1576	D00141
Non-interactive	hsa:7367	D00283
Non-interactive	hsa:29785	D02115
Non-interactive	hsa:3551	D00283
Non-interactive	hsa:5495	D00653
Non-interactive	hsa:129807	D00188
Non-interactive	hsa:5331	D00887
Non-interactive	hsa:7046	D02375
Non-interactive	hsa:5338	D02115
Non-interactive	hsa:11238	D00364
Non-interactive	hsa:1847	D00283
Non-interactive	hsa:2193	D00454
Non-interactive	hsa:4915	D02581
Non-interactive	hsa:1267	D00120
Non-interactive	hsa:2098	D03350
Non-interactive	hsa:5650	D00969
Non-interactive	hsa:196883	D03710
Non-interactive	hsa:5531	D02451
Non-interactive	hsa:2050	D00070
Non-interactive	hsa:246	D01432
Non-interactive	hsa:1675	D02709
Non-interactive	hsa:2158	D00394
Non-interactive	hsa:1890	D00892
Non-interactive	hsa:10188	D00963
Non-interactive	hsa:63036	D01565
Non-interactive	hsa:5516	D00094
Non-interactive	hsa:55775	D01441
Non-interactive	hsa:1565	D00315

Non-interactive	hsa:8854	D00218
Non-interactive	hsa:2232	D00963
Non-interactive	hsa:2161	D03806
Non-interactive	hsa:613	D00965
Non-interactive	hsa:84812	D01164
Non-interactive	hsa:1432	D03689
Non-interactive	hsa:3988	D00516
Non-interactive	hsa:5550	D02229
Non-interactive	hsa:5531	D01164
Non-interactive	hsa:5051	D01765
Non-interactive	hsa:51727	D03736
Non-interactive	hsa:1723	D04031
Non-interactive	hsa:132160	D01064
Non-interactive	hsa:100	D00198
Non-interactive	hsa:1675	D02166
Non-interactive	hsa:1586	D03803
Non-interactive	hsa:7957	D02560
Non-interactive	hsa:5169	D01911
Non-interactive	hsa:5138	D02451
Non-interactive	hsa:1852	D01977
Non-interactive	hsa:1846	D03899
Non-interactive	hsa:54579	D00094
Non-interactive	hsa:2339	D03717
Non-interactive	hsa:5538	D01828
Non-interactive	hsa:57176	D00139
Non-interactive	hsa:5294	D00203
Non-interactive	hsa:5562	D01984
Non-interactive	hsa:58190	D03735
Non-interactive	hsa:32	D02560
Non-interactive	hsa:4353	D00488
Non-interactive	hsa:1846	D00414
Non-interactive	hsa:3002	D02368
Non-interactive	hsa:4353	D02418
Non-interactive	hsa:1588	D00002
Non-interactive	hsa:8836	D03823
Non-interactive	hsa:5144	D00630
Non-interactive	hsa:2042	D03735
Non-interactive	hsa:134510	D03775
Non-interactive	hsa:10549	D01164
Non-interactive	hsa:11221	D03743
Non-interactive	hsa:54576	D03805
Non-interactive	hsa:3643	D01332

Non-interactive	hsa:9641	D00781
Non-interactive	hsa:25824	D00947
Non-interactive	hsa:1504	D02368
Non-interactive	hsa:5147	D03741
Non-interactive	hsa:5476	D01907
Non-interactive	hsa:7453	D00434
Non-interactive	hsa:7156	D01828
Non-interactive	hsa:5152	D00107
Non-interactive	hsa:55512	D02709
Non-interactive	hsa:1584	D01133
Non-interactive	hsa:1557	D01064
Non-interactive	hsa:2639	D01918
Non-interactive	hsa:3001	D03440
Non-interactive	hsa:8836	D00900
Non-interactive	hsa:5532	D02008
Non-interactive	hsa:1803	D00651
Non-interactive	hsa:5495	D00519
Non-interactive	hsa:5657	D01164
Non-interactive	hsa:5337	D03765
Non-interactive	hsa:111	D02193
Non-interactive	hsa:635	D00654
Non-interactive	hsa:130399	D00328
Non-interactive	hsa:3001	D00516
Non-interactive	hsa:35	D01866
Non-interactive	hsa:1645	D04025
Non-interactive	hsa:84532	D03738
Non-interactive	hsa:5602	D00437
Non-interactive	hsa:354	D00889
Non-interactive	hsa:3001	D00463
Non-interactive	hsa:5351	D00488
Non-interactive	hsa:7083	D03798
Non-interactive	hsa:613	D01397
Non-interactive	hsa:5479	D00515
Non-interactive	hsa:191	D04024
Non-interactive	hsa:23436	D02008
Non-interactive	hsa:2263	D01276
Non-interactive	hsa:242	D00805
Non-interactive	hsa:2882	D00325
Non-interactive	hsa:10056	D02110
Non-interactive	hsa:1431	D03767
Non-interactive	hsa:6609	D00449
Non-interactive	hsa:2595	D01133

Non-interactive	hsa:5150	D02110
Non-interactive	hsa:353	D02731
Non-interactive	hsa:1056	D00781
Non-interactive	hsa:3990	D02698
Non-interactive	hsa:2065	D01027
Non-interactive	hsa:2193	D03787
Non-interactive	hsa:38	D00315
Non-interactive	hsa:2042	D00733
Non-interactive	hsa:5150	D00892
Non-interactive	hsa:4017	D03806
Non-interactive	hsa:2158	D02008
Non-interactive	hsa:3817	D00285
Non-interactive	hsa:8435	D00515
Non-interactive	hsa:2677	D02756
Non-interactive	hsa:3291	D01984
Non-interactive	hsa:151531	D01582
Non-interactive	hsa:5445	D00225
Non-interactive	hsa:112	D01196
Non-interactive	hsa:2746	D01842
Non-interactive	hsa:1545	D03758
Non-interactive	hsa:150290	D04025
Non-interactive	hsa:2595	D03798
Non-interactive	hsa:28	D02756
Non-interactive	hsa:10825	D00437
Non-interactive	hsa:23430	D01211
Non-interactive	hsa:1800	D00550
Non-interactive	hsa:2224	D01918
Non-interactive	hsa:2260	D00658
Non-interactive	hsa:6241	D01578
Non-interactive	hsa:5330	D00315
Non-interactive	hsa:2066	D00387
Non-interactive	hsa:5052	D03803
Non-interactive	hsa:28	D00519
Non-interactive	hsa:10269	D00449
Non-interactive	hsa:3939	D04031
Non-interactive	hsa:2066	D01973
Non-interactive	hsa:112	D00889
Non-interactive	hsa:8972	D02756
Non-interactive	hsa:5747	D04025
Non-interactive	hsa:124	D00963
Non-interactive	hsa:339221	D03440
Non-interactive	hsa:7957	D00218

Non-interactive	hsa:124	D00658
Non-interactive	hsa:4881	D00340
Non-interactive	hsa:2822	D01164
Non-interactive	hsa:1543	D01844
Non-interactive	hsa:5328	D02328
Non-interactive	hsa:5137	D00298
Non-interactive	hsa:2582	D02375
Non-interactive	hsa:5599	D02168
Non-interactive	hsa:3551	D02258
Non-interactive	hsa:51166	D00155
Non-interactive	hsa:1990	D00002
Non-interactive	hsa:132160	D00285
Non-interactive	hsa:6652	D00155
Non-interactive	hsa:5607	D00394
Non-interactive	hsa:4282	D02173
Non-interactive	hsa:53	D01767
Non-interactive	hsa:51205	D03788
Non-interactive	hsa:64902	D02368
Non-interactive	hsa:2339	D00437
Non-interactive	hsa:51166	D00651
Non-interactive	hsa:25796	D02655
Non-interactive	hsa:2098	D00650
Non-interactive	hsa:1582	D03689
Non-interactive	hsa:3645	D03826
Non-interactive	hsa:5150	D00781
Non-interactive	hsa:2065	D03788
Non-interactive	hsa:29920	D03743
Non-interactive	hsa:11238	D00125
Non-interactive	hsa:5602	D03823
Non-interactive	hsa:5607	D00283
Non-interactive	hsa:5515	D00652
Non-interactive	hsa:3716	D01825
Non-interactive	hsa:1584	D00656
Non-interactive	hsa:51207	D00622
Non-interactive	hsa:28972	D01866
Non-interactive	hsa:1580	D01164
Non-interactive	hsa:9945	D00293
Non-interactive	hsa:30814	D00463
Non-interactive	hsa:5860	D02731
Non-interactive	hsa:1360	D00515
Non-interactive	hsa:30833	D01885
Non-interactive	hsa:29941	D01915

Non-interactive	hsa:225689	D02194
Non-interactive	hsa:10327	D03720
Non-interactive	hsa:54659	D01565
Non-interactive	hsa:29785	D04025
Non-interactive	hsa:5515	D03716
Non-interactive	hsa:4953	D02173
Non-interactive	hsa:10935	D02333
Non-interactive	hsa:2050	D00515
Non-interactive	hsa:270	D00127
Non-interactive	hsa:224	D00434
Non-interactive	hsa:8288	D00947
Non-interactive	hsa:7363	D03758
Non-interactive	hsa:23239	D00394
Non-interactive	hsa:5050	D00538
Non-interactive	hsa:219	D00434
Non-interactive	hsa:63904	D01332
Non-interactive	hsa:7535	D03805
Non-interactive	hsa:4835	D02655
Non-interactive	hsa:84532	D00488
Non-interactive	hsa:326625	D04031
Non-interactive	hsa:1586	D00488
Non-interactive	hsa:142679	D02173
Non-interactive	hsa:513	D00630
Non-interactive	hsa:80824	D03077
Non-interactive	hsa:5470	D00969
Non-interactive	hsa:4282	D01370
Non-interactive	hsa:52	D03012
Non-interactive	hsa:1588	D04024
Non-interactive	hsa:22954	D00387
Non-interactive	hsa:93650	D00463
Non-interactive	hsa:5602	D04029
Non-interactive	hsa:5052	D00414
Non-interactive	hsa:112	D00596
Non-interactive	hsa:1956	D00623
Non-interactive	hsa:5747	D00550
Non-interactive	hsa:5531	D03736
Non-interactive	hsa:2280	D00196
Non-interactive	hsa:242	D03738
Non-interactive	hsa:8654	D00533
Non-interactive	hsa:7298	D00550
Non-interactive	hsa:260293	D00448
Non-interactive	hsa:94009	D00131

Non-interactive	hsa:6713	D00622
Non-interactive	hsa:377677	D01828
Non-interactive	hsa:285220	D00813
Non-interactive	hsa:3988	D00885
Non-interactive	hsa:658	D00449
Non-interactive	hsa:5602	D00455
Non-interactive	hsa:5562	D00533
Non-interactive	hsa:8529	D00414
Non-interactive	hsa:5167	D00530
Non-interactive	hsa:5330	D02333
Non-interactive	hsa:2534	D02194
Non-interactive	hsa:2261	D01064
Non-interactive	hsa:30814	D00155
Non-interactive	hsa:1800	D00218
Non-interactive	hsa:3291	D00620
Non-interactive	hsa:2595	D01240
Non-interactive	hsa:4860	D00654
Non-interactive	hsa:5033	D01690
Non-interactive	hsa:111	D00285
Non-interactive	hsa:9420	D00094
Non-interactive	hsa:8288	D02671
Non-interactive	hsa:5143	D00131
Non-interactive	hsa:2180	D01196
Non-interactive	hsa:4482	D00519
Non-interactive	hsa:18	D00620
Non-interactive	hsa:768	D00131
Non-interactive	hsa:56922	D04031
Non-interactive	hsa:2044	D01276
Non-interactive	hsa:1845	D02451
Non-interactive	hsa:7957	D01275
Non-interactive	hsa:3002	D02655
Non-interactive	hsa:1549	D00969
Non-interactive	hsa:84152	D01690
Non-interactive	hsa:5138	D00563
Non-interactive	hsa:8940	D00519
Non-interactive	hsa:2263	D01842
Non-interactive	hsa:660	D00892
Non-interactive	hsa:495	D00398
Non-interactive	hsa:51365	D02110
Non-interactive	hsa:5145	D00294
Non-interactive	hsa:5422	D00902
Non-interactive	hsa:327	D03765

Non-interactive	hsa:115	D03806
Non-interactive	hsa:30	D03717
Non-interactive	hsa:5494	D01565
Non-interactive	hsa:7155	D00094
Non-interactive	hsa:9388	D00359
Non-interactive	hsa:218	D00752
Non-interactive	hsa:53	D01915
Non-interactive	hsa:54878	D00198
Non-interactive	hsa:765	D03823
Non-interactive	hsa:5445	D00813
Non-interactive	hsa:6716	D03712
Non-interactive	hsa:1056	D00884
Non-interactive	hsa:4067	D02328
Non-interactive	hsa:5052	D03882
Non-interactive	hsa:51365	D00328
Non-interactive	hsa:115	D02173
Non-interactive	hsa:7046	D02556
Non-interactive	hsa:5608	D01276
Non-interactive	hsa:5423	D00900
Non-interactive	hsa:7010	D00148
Non-interactive	hsa:5650	D02671
Non-interactive	hsa:645	D01064
Non-interactive	hsa:5837	D03689
Non-interactive	hsa:1800	D03736
Non-interactive	hsa:10269	D00387
Non-interactive	hsa:5148	D04029
Non-interactive	hsa:2042	D01367
Non-interactive	hsa:30833	D00515
Non-interactive	hsa:29920	D03805
Non-interactive	hsa:5831	D00515
Non-interactive	hsa:5407	D00892
Non-interactive	hsa:55775	D02731
Non-interactive	hsa:5144	D04025
Non-interactive	hsa:64902	D01828
Non-interactive	hsa:29920	D03826
Non-interactive	hsa:7075	D00107
Non-interactive	hsa:3028	D00218
Non-interactive	hsa:91	D01825
Non-interactive	hsa:133121	D00516
Non-interactive	hsa:2066	D02451
Non-interactive	hsa:10549	D03012
Non-interactive	hsa:5837	D01765

Non-interactive	hsa:7957	D00630
Non-interactive	hsa:51	D02229
Non-interactive	hsa:34	D04029
Non-interactive	hsa:1584	D03736
Non-interactive	hsa:7301	D02671
Non-interactive	hsa:766	D00216
Non-interactive	hsa:5550	D02556
Non-interactive	hsa:4129	D02451
Non-interactive	hsa:8836	D01918
Non-interactive	hsa:54878	D00364
Non-interactive	hsa:55811	D01264
Non-interactive	hsa:3990	D00188
Non-interactive	hsa:8836	D02375
Non-interactive	hsa:5972	D01332
Non-interactive	hsa:28972	D00294
Non-interactive	hsa:6713	D00394
Non-interactive	hsa:2180	D02698
Non-interactive	hsa:5538	D00596
Non-interactive	hsa:7365	D02487
Non-interactive	hsa:4953	D00414
Non-interactive	hsa:683	D03720
Non-interactive	hsa:6898	D03717
Non-interactive	hsa:151531	D02731
Non-interactive	hsa:50484	D00455
Non-interactive	hsa:353	D00391
Non-interactive	hsa:2882	D00519
Non-interactive	hsa:3906	D02709
Non-interactive	hsa:4190	D01397
Non-interactive	hsa:51205	D00563
Non-interactive	hsa:94009	D02229
Non-interactive	hsa:5333	D04024
Non-interactive	hsa:51251	D01862
Non-interactive	hsa:57016	D00563
Non-interactive	hsa:660	D03788
Non-interactive	hsa:7298	D03758
Non-interactive	hsa:1033	D00965
Non-interactive	hsa:4593	D00225
Non-interactive	hsa:54677	D01223
Non-interactive	hsa:4190	D01828
Non-interactive	hsa:4145	D00423
Non-interactive	hsa:6713	D03728
Non-interactive	hsa:5322	D03738

Non-interactive	hsa:51727	D03350
Non-interactive	hsa:2064	D00892
Non-interactive	hsa:10056	D01715
Non-interactive	hsa:2639	D03778
Non-interactive	hsa:327	D00969
Non-interactive	hsa:28227	D02709
Non-interactive	hsa:1549	D00813
Non-interactive	hsa:1571	D00538
Non-interactive	hsa:11072	D01441
Non-interactive	hsa:3614	D01862
Non-interactive	hsa:9647	D00630
Non-interactive	hsa:6897	D03816
Non-interactive	hsa:670	D00216
Non-interactive	hsa:29968	D00387
Non-interactive	hsa:7010	D03643
Non-interactive	hsa:4128	D03805
Non-interactive	hsa:9150	D01196
Non-interactive	hsa:216	D00537
Non-interactive	hsa:1549	D03882
Non-interactive	hsa:10461	D00018
Non-interactive	hsa:7372	D00285
Non-interactive	hsa:5834	D00394
Non-interactive	hsa:1432	D03350
Non-interactive	hsa:2242	D00623
Non-interactive	hsa:1990	D00550
Non-interactive	hsa:4552	D00398
Non-interactive	hsa:124	D00902
Non-interactive	hsa:7364	D01119
Non-interactive	hsa:64816	D00463
Non-interactive	hsa:5479	D01690
Non-interactive	hsa:2339	D03689
Non-interactive	hsa:91039	D01211
Non-interactive	hsa:2328	D03734
Non-interactive	hsa:2326	D03350
Non-interactive	hsa:7172	D00005
Non-interactive	hsa:28227	D00753
Non-interactive	hsa:3035	D03778
Non-interactive	hsa:6713	D01432
Non-interactive	hsa:54600	D00391
Non-interactive	hsa:5096	D00364
Non-interactive	hsa:8940	D00196
Non-interactive	hsa:5168	D01715

Non-interactive	hsa:54490	D00315
Non-interactive	hsa:54490	D01565
Non-interactive	hsa:8399	D02756
Non-interactive	hsa:1548	D00550
Non-interactive	hsa:189	D03828
Non-interactive	hsa:5979	D03899
Non-interactive	hsa:32	D01984
Non-interactive	hsa:7535	D00969
Non-interactive	hsa:5657	D02375
Non-interactive	hsa:2638	D00530
Non-interactive	hsa:3001	D00218
Non-interactive	hsa:9088	D00537
Non-interactive	hsa:94009	D02115
Non-interactive	hsa:1215	D00139
Non-interactive	hsa:128	D03788
Non-interactive	hsa:93	D02008
Non-interactive	hsa:31	D00198
Non-interactive	hsa:3945	D02487
Non-interactive	hsa:2322	D01718
Non-interactive	hsa:11330	D04031
Non-interactive	hsa:51727	D02487
Non-interactive	hsa:8622	D00283
Non-interactive	hsa:5151	D04024
Non-interactive	hsa:5601	D00325
Non-interactive	hsa:93	D00141
Non-interactive	hsa:1557	D01196
Non-interactive	hsa:54657	D00298
Non-interactive	hsa:2950	D00142
Non-interactive	hsa:5605	D03823
Non-interactive	hsa:1543	D02487
Non-interactive	hsa:9150	D00463
Non-interactive	hsa:4837	D00455
Non-interactive	hsa:5147	D02173
Non-interactive	hsa:55	D03899
Non-interactive	hsa:4916	D02258
Non-interactive	hsa:242	D03712
Non-interactive	hsa:1445	D00127
Non-interactive	hsa:6768	D00884
Non-interactive	hsa:2280	D00516
Non-interactive	hsa:218	D00654
Non-interactive	hsa:5053	D01973
Non-interactive	hsa:768	D00423

Non-interactive	hsa:8836	D02194
Non-interactive	hsa:3818	D02418
Non-interactive	hsa:5152	D02194
Non-interactive	hsa:8513	D01133
Non-interactive	hsa:5533	D03767
Non-interactive	hsa:3932	D01027
Non-interactive	hsa:7046	D01915
Non-interactive	hsa:5406	D00488
Non-interactive	hsa:219	D01842
Non-interactive	hsa:1431	D00423
Non-interactive	hsa:4953	D01578
Non-interactive	hsa:84152	D03788
Non-interactive	hsa:100	D00752
Non-interactive	hsa:111	D01256
Non-interactive	hsa:8622	D00070
Non-interactive	hsa:4311	D02441
Non-interactive	hsa:9420	D00328
Non-interactive	hsa:7156	D00900
Non-interactive	hsa:10720	D00448
Non-interactive	hsa:238	D03788
Non-interactive	hsa:43	D01275
Non-interactive	hsa:4482	D01211
Non-interactive	hsa:35	D01133
Non-interactive	hsa:27032	D01825
Non-interactive	hsa:5494	D00771
Non-interactive	hsa:30814	D00094
Non-interactive	hsa:5050	D00208
Non-interactive	hsa:7367	D00285
Non-interactive	hsa:2328	D00449
Non-interactive	hsa:5495	D03882
Non-interactive	hsa:30	D04029
Non-interactive	hsa:5053	D00198
Non-interactive	hsa:8836	D00107
Non-interactive	hsa:5747	D02769
Non-interactive	hsa:2328	D01133
Non-interactive	hsa:5152	D01907
Non-interactive	hsa:64802	D01977
Non-interactive	hsa:125	D01276
Non-interactive	hsa:7010	D00596
Non-interactive	hsa:834	D00885
Non-interactive	hsa:64087	D01866
Non-interactive	hsa:10941	D00398

Non-interactive	hsa:51251	D00651
Non-interactive	hsa:55811	D01885
Non-interactive	hsa:1436	D02556
Non-interactive	hsa:5336	D00902
Non-interactive	hsa:8556	D00387
Non-interactive	hsa:84152	D00449
Non-interactive	hsa:4835	D04031
Non-interactive	hsa:1571	D00651
Non-interactive	hsa:6799	D00887
Non-interactive	hsa:8622	D00293
Non-interactive	hsa:2260	D03826
Non-interactive	hsa:5562	D01974
Non-interactive	hsa:36	D00885
Non-interactive	hsa:1360	D03643
Non-interactive	hsa:115	D04031
Non-interactive	hsa:112	D00325
Non-interactive	hsa:5547	D01256
Non-interactive	hsa:284541	D03743
Non-interactive	hsa:4907	D00324
Non-interactive	hsa:5499	D00449
Non-interactive	hsa:111	D00654
Non-interactive	hsa:5140	D00889
Non-interactive	hsa:5834	D04025
Non-interactive	hsa:5602	D00753
Non-interactive	hsa:4023	D02580
Non-interactive	hsa:51166	D02375
Non-interactive	hsa:5595	D02769
Non-interactive	hsa:79799	D00294
Non-interactive	hsa:7172	D00148
Non-interactive	hsa:2185	D01180
Non-interactive	hsa:6716	D01767
Non-interactive	hsa:3791	D00620
Non-interactive	hsa:28	D00328
Non-interactive	hsa:13	D00516
Non-interactive	hsa:1803	D00141
Non-interactive	hsa:3645	D00131
Non-interactive	hsa:6240	D00884
Non-interactive	hsa:5319	D03806
Non-interactive	hsa:444	D00094
Non-interactive	hsa:5052	D00887
Non-interactive	hsa:6610	D03716
Non-interactive	hsa:6652	D00567

Non-interactive	hsa:2242	D00141
Non-interactive	hsa:5335	D00538
Non-interactive	hsa:590	D00620
Non-interactive	hsa:50	D00218
Non-interactive	hsa:216	D00533
Non-interactive	hsa:1376	D00454
Non-interactive	hsa:4128	D00298
Non-interactive	hsa:5422	D00455
Non-interactive	hsa:1549	D02110
Non-interactive	hsa:2064	D00293
Non-interactive	hsa:10056	D02418
Non-interactive	hsa:1549	D03743
Non-interactive	hsa:1579	D01133
Non-interactive	hsa:5140	D00448
Non-interactive	hsa:43	D03788
Non-interactive	hsa:84152	D03716
Non-interactive	hsa:2147	D00884
Non-interactive	hsa:5140	D00887
Non-interactive	hsa:57176	D00449
Non-interactive	hsa:7015	D00094
Non-interactive	hsa:5156	D03803
Non-interactive	hsa:686	D00216
Non-interactive	hsa:5407	D02556
Non-interactive	hsa:8192	D00596
Non-interactive	hsa:5320	D00050
Non-interactive	hsa:5597	D00448
Non-interactive	hsa:3001	D00002
Non-interactive	hsa:2638	D00623
Non-interactive	hsa:765	D00969
Non-interactive	hsa:55902	D00593
Non-interactive	hsa:5053	D01397
Non-interactive	hsa:5335	D01765
Non-interactive	hsa:3141	D03767
Non-interactive	hsa:4025	D00752
Non-interactive	hsa:128	D02194
Non-interactive	hsa:30833	D01715
Non-interactive	hsa:54576	D01164
Non-interactive	hsa:5294	D01264
Non-interactive	hsa:8556	D03805
Non-interactive	hsa:5754	D00567
Non-interactive	hsa:10279	D03798
Non-interactive	hsa:2882	D00131

Non-interactive	hsa:5033	D03826
Non-interactive	hsa:54657	D00364
Non-interactive	hsa:10667	D00437
Non-interactive	hsa:501	D01211
Non-interactive	hsa:2822	D00781
Non-interactive	hsa:1848	D00018
Non-interactive	hsa:1360	D01718
Non-interactive	hsa:3141	D01973
Non-interactive	hsa:4881	D01275
Non-interactive	hsa:7153	D00567
Non-interactive	hsa:1723	D03716
Non-interactive	hsa:349565	D00315
Non-interactive	hsa:4311	D00394
Non-interactive	hsa:64902	D03765
Non-interactive	hsa:2806	D03823
Non-interactive	hsa:5033	D00654
Non-interactive	hsa:64499	D02333
Non-interactive	hsa:5447	D03826
Non-interactive	hsa:5530	D01240
Non-interactive	hsa:2065	D04029
Non-interactive	hsa:36	D00203
Non-interactive	hsa:5501	D00188
Non-interactive	hsa:54878	D00131
Non-interactive	hsa:1583	D00120
Non-interactive	hsa:767	D00805
Non-interactive	hsa:5650	D00900
Non-interactive	hsa:7365	D00893
Non-interactive	hsa:5595	D02214
Non-interactive	hsa:54878	D00813
Non-interactive	hsa:5144	D00652
Non-interactive	hsa:1374	D01844
Non-interactive	hsa:32	D02328
Non-interactive	hsa:1991	D00969
Non-interactive	hsa:1445	D00002
Non-interactive	hsa:5294	D02258
Non-interactive	hsa:7174	D03689
Non-interactive	hsa:5096	D00622
Non-interactive	hsa:4915	D00455
Non-interactive	hsa:1719	D02698
Non-interactive	hsa:43	D00148
Non-interactive	hsa:558	D00070
Non-interactive	hsa:1565	D02441

Non-interactive	hsa:7301	D00127
Non-interactive	hsa:32	D01432
Non-interactive	hsa:5479	D00653
Non-interactive	hsa:5147	D00902
Non-interactive	hsa:1545	D02368
Non-interactive	hsa:4311	D00654
Non-interactive	hsa:125	D01688
Non-interactive	hsa:10667	D01397
Non-interactive	hsa:1845	D03077
Non-interactive	hsa:3932	D02560
Non-interactive	hsa:5494	D02769
Non-interactive	hsa:6416	D00733
Non-interactive	hsa:23475	D01196
Non-interactive	hsa:9088	D01844
Non-interactive	hsa:5604	D03077
Non-interactive	hsa:5049	D01690
Non-interactive	hsa:3295	D00652
Non-interactive	hsa:2356	D03743
Non-interactive	hsa:5837	D01715
Non-interactive	hsa:8622	D00131
Non-interactive	hsa:54677	D00651
Non-interactive	hsa:6416	D00656
Non-interactive	hsa:5979	D03803
Non-interactive	hsa:247	D00107
Non-interactive	hsa:657	D02671
Non-interactive	hsa:11221	D00196
Non-interactive	hsa:3645	D00298
Non-interactive	hsa:5495	D02731
Non-interactive	hsa:23632	D01276
Non-interactive	hsa:2049	D00198
Non-interactive	hsa:2155	D04292
Non-interactive	hsa:224	D01825
Non-interactive	hsa:5608	D00655
Non-interactive	hsa:10667	D02581
Non-interactive	hsa:2047	D03805
Non-interactive	hsa:84152	D03734
Non-interactive	hsa:4067	D00387
Non-interactive	hsa:5607	D01275
Non-interactive	hsa:3791	D01275
Non-interactive	hsa:1376	D00620
Non-interactive	hsa:85313	D01973
Non-interactive	hsa:2280	D01974

Non-interactive	hsa:771	D00963
Non-interactive	hsa:9088	D00187
Non-interactive	hsa:51251	D01256
Non-interactive	hsa:7075	D00947
Non-interactive	hsa:8622	D03728
Non-interactive	hsa:29785	D02698
Non-interactive	hsa:6716	D03803
Non-interactive	hsa:5050	D00656
Non-interactive	hsa:7299	D00298
Non-interactive	hsa:5142	D00002
Non-interactive	hsa:7525	D02110
Non-interactive	hsa:5650	D00965
Non-interactive	hsa:1636	D04025
Non-interactive	hsa:1593	D03751
Non-interactive	hsa:196883	D02769
Non-interactive	hsa:5531	D02418
Non-interactive	hsa:64802	D04292
Non-interactive	hsa:246	D01180
Non-interactive	hsa:1580	D02168
Non-interactive	hsa:57176	D00414
Non-interactive	hsa:80339	D00887
Non-interactive	hsa:8940	D03077
Non-interactive	hsa:1890	D00884
Non-interactive	hsa:5337	D00651
Non-interactive	hsa:1573	D01264
Non-interactive	hsa:10188	D00969
Non-interactive	hsa:1843	D03012
Non-interactive	hsa:5142	D02441
Non-interactive	hsa:5422	D00203
Non-interactive	hsa:1576	D01885
Non-interactive	hsa:55775	D03758
Non-interactive	hsa:2045	D00298
Non-interactive	hsa:2232	D00885
Non-interactive	hsa:5053	D00947
Non-interactive	hsa:5144	D00550
Non-interactive	hsa:247	D01180
Non-interactive	hsa:1584	D02328
Non-interactive	hsa:613	D00947
Non-interactive	hsa:51292	D00752
Non-interactive	hsa:1432	D03710
Non-interactive	hsa:5550	D02042
Non-interactive	hsa:63904	D00364

Non-interactive	hsa:5051	D01915
Non-interactive	hsa:51727	D03735
Non-interactive	hsa:5478	D00530
Non-interactive	hsa:1723	D04024
Non-interactive	hsa:100	D00196
Non-interactive	hsa:1675	D01977
Non-interactive	hsa:1586	D03775
Non-interactive	hsa:7957	D02441
Non-interactive	hsa:5169	D01862
Non-interactive	hsa:5138	D02375
Non-interactive	hsa:1852	D01918
Non-interactive	hsa:2339	D02580
Non-interactive	hsa:2582	D03218
Non-interactive	hsa:5294	D00188
Non-interactive	hsa:5562	D01918
Non-interactive	hsa:58190	D03734
Non-interactive	hsa:4353	D00516
Non-interactive	hsa:3002	D02375
Non-interactive	hsa:2766	D02193
Non-interactive	hsa:5601	D00654
Non-interactive	hsa:8836	D03826
Non-interactive	hsa:2042	D03710
Non-interactive	hsa:2673	D02193
Non-interactive	hsa:10549	D01133
Non-interactive	hsa:54576	D03765
Non-interactive	hsa:7172	D03751
Non-interactive	hsa:9641	D00771
Non-interactive	hsa:128	D03816
Non-interactive	hsa:5147	D03728
Non-interactive	hsa:23475	D00188
Non-interactive	hsa:5033	D02258
Non-interactive	hsa:5476	D01715
Non-interactive	hsa:7453	D00437
Non-interactive	hsa:3712	D03803
Non-interactive	hsa:7156	D01767
Non-interactive	hsa:1990	D04025
Non-interactive	hsa:1584	D01061
Non-interactive	hsa:683	D00733
Non-interactive	hsa:2639	D01842
Non-interactive	hsa:6799	D03735
Non-interactive	hsa:8836	D00889
Non-interactive	hsa:5532	D01977

Non-interactive	hsa:58190	D01918
Non-interactive	hsa:8555	D00623
Non-interactive	hsa:1803	D00653
Non-interactive	hsa:84706	D01688
Non-interactive	hsa:111	D02214
Non-interactive	hsa:4953	D01825
Non-interactive	hsa:635	D00630
Non-interactive	hsa:130399	D00325
Non-interactive	hsa:84706	D00516
Non-interactive	hsa:3001	D00515
Non-interactive	hsa:1645	D03806
Non-interactive	hsa:84532	D03717
Non-interactive	hsa:354	D00965
Non-interactive	hsa:3001	D00455
Non-interactive	hsa:3717	D00781
Non-interactive	hsa:7083	D03803
Non-interactive	hsa:1719	D00963
Non-interactive	hsa:191	D03806
Non-interactive	hsa:23436	D01718
Non-interactive	hsa:242	D00650
Non-interactive	hsa:2882	D00285
Non-interactive	hsa:51	D02709
Non-interactive	hsa:10056	D02168
Non-interactive	hsa:1431	D03765
Non-interactive	hsa:2595	D00963
Non-interactive	hsa:353	D03440
Non-interactive	hsa:1056	D00753
Non-interactive	hsa:3990	D02709
Non-interactive	hsa:54677	D01866
Non-interactive	hsa:51205	D02328
Non-interactive	hsa:2065	D00947
Non-interactive	hsa:1573	D02333
Non-interactive	hsa:2193	D03758
Non-interactive	hsa:2042	D00652
Non-interactive	hsa:51727	D02451
Non-interactive	hsa:2158	D01907
Non-interactive	hsa:115	D01862
Non-interactive	hsa:5516	D00892
Non-interactive	hsa:81579	D03734
Non-interactive	hsa:112	D01240
Non-interactive	hsa:2746	D01690
Non-interactive	hsa:55775	D00285

Non-interactive	hsa:2595	D00437
Non-interactive	hsa:6646	D00947
Non-interactive	hsa:7075	D00187
Non-interactive	hsa:2224	D01825
Non-interactive	hsa:2260	D00596
Non-interactive	hsa:5330	D00298
Non-interactive	hsa:23475	D01061
Non-interactive	hsa:28	D00463
Non-interactive	hsa:476	D01180
Non-interactive	hsa:2065	D01061
Non-interactive	hsa:2822	D00488
Non-interactive	hsa:10269	D00454
Non-interactive	hsa:3939	D04292
Non-interactive	hsa:8972	D02731
Non-interactive	hsa:2224	D00654
Non-interactive	hsa:54578	D00107
Non-interactive	hsa:5747	D03882
Non-interactive	hsa:339221	D03689
Non-interactive	hsa:2324	D00340
Non-interactive	hsa:124	D00654
Non-interactive	hsa:1719	D00652
Non-interactive	hsa:1543	D01866
Non-interactive	hsa:5137	D00285
Non-interactive	hsa:2582	D02229
Non-interactive	hsa:5599	D01974
Non-interactive	hsa:5447	D00414
Non-interactive	hsa:218	D00530
Non-interactive	hsa:85313	D00902
Non-interactive	hsa:51166	D00187
Non-interactive	hsa:8836	D00142
Non-interactive	hsa:1969	D03882
Non-interactive	hsa:5657	D00538
Non-interactive	hsa:4282	D02258
Non-interactive	hsa:53	D01825
Non-interactive	hsa:5979	D00656
Non-interactive	hsa:5423	D00285
Non-interactive	hsa:2339	D00340
Non-interactive	hsa:2135	D00141
Non-interactive	hsa:2098	D00620
Non-interactive	hsa:1582	D03077
Non-interactive	hsa:3818	D00070
Non-interactive	hsa:327	D00449

Non-interactive	hsa:11238	D00131
Non-interactive	hsa:5602	D03816
Non-interactive	hsa:5607	D00285
Non-interactive	hsa:3716	D01918
Non-interactive	hsa:1584	D00650
Non-interactive	hsa:51181	D00359
Non-interactive	hsa:9641	D01119
Non-interactive	hsa:51207	D00652
Non-interactive	hsa:5645	D00488
Non-interactive	hsa:1580	D01027
Non-interactive	hsa:9945	D00298
Non-interactive	hsa:7365	D00340
Non-interactive	hsa:2638	D00651
Non-interactive	hsa:3290	D00448
Non-interactive	hsa:5052	D00005
Non-interactive	hsa:30833	D01866
Non-interactive	hsa:1384	D00593
Non-interactive	hsa:3551	D00398
Non-interactive	hsa:6610	D02581
Non-interactive	hsa:29941	D01862
Non-interactive	hsa:225689	D01974
Non-interactive	hsa:10327	D03728
Non-interactive	hsa:6609	D02193
Non-interactive	hsa:54659	D01275
Non-interactive	hsa:2582	D02173
Non-interactive	hsa:26279	D00516
Non-interactive	hsa:2950	D00437
Non-interactive	hsa:2050	D00463
Non-interactive	hsa:224	D00387
Non-interactive	hsa:5444	D00359
Non-interactive	hsa:23239	D00293
Non-interactive	hsa:219	D00391
Non-interactive	hsa:7535	D03816
Non-interactive	hsa:84532	D00437
Non-interactive	hsa:1586	D00455
Non-interactive	hsa:2160	D00315
Non-interactive	hsa:513	D00654
Non-interactive	hsa:80824	D02581
Non-interactive	hsa:5470	D00889
Non-interactive	hsa:52	D02769
Non-interactive	hsa:768	D00196
Non-interactive	hsa:9088	D00127

Non-interactive	hsa:22954	D00340
Non-interactive	hsa:4143	D03826
Non-interactive	hsa:31	D02328
Non-interactive	hsa:2147	D00753
Non-interactive	hsa:112	D00623
Non-interactive	hsa:1593	D03736
Non-interactive	hsa:5531	D03735
Non-interactive	hsa:242	D03218
Non-interactive	hsa:27032	D00188
Non-interactive	hsa:5601	D00454
Non-interactive	hsa:6646	D00196
Non-interactive	hsa:27032	D00094
Non-interactive	hsa:2185	D02193
Non-interactive	hsa:6713	D00620
Non-interactive	hsa:377677	D01885
Non-interactive	hsa:84695	D00900
Non-interactive	hsa:5530	D01264
Non-interactive	hsa:3988	D00893
Non-interactive	hsa:5319	D00325
Non-interactive	hsa:5602	D00448
Non-interactive	hsa:2534	D01915
Non-interactive	hsa:2261	D00947
Non-interactive	hsa:377677	D00900
Non-interactive	hsa:3480	D02580
Non-interactive	hsa:38	D00018
Non-interactive	hsa:30814	D00148
Non-interactive	hsa:189	D03741
Non-interactive	hsa:1571	D02193
Non-interactive	hsa:4914	D00434
Non-interactive	hsa:54878	D00139
Non-interactive	hsa:1800	D00216
Non-interactive	hsa:768	D00963
Non-interactive	hsa:2595	D01061
Non-interactive	hsa:5033	D01844
Non-interactive	hsa:1543	D01715
Non-interactive	hsa:111	D00283
Non-interactive	hsa:2339	D01862
Non-interactive	hsa:2180	D01064
Non-interactive	hsa:18	D00593
Non-interactive	hsa:31	D03823
Non-interactive	hsa:56922	D03899
Non-interactive	hsa:2044	D01133

Non-interactive	hsa:1845	D02418
Non-interactive	hsa:84152	D01432
Non-interactive	hsa:5138	D00550
Non-interactive	hsa:2263	D01690
Non-interactive	hsa:660	D00813
Non-interactive	hsa:55	D02008
Non-interactive	hsa:658	D03736
Non-interactive	hsa:7498	D02110
Non-interactive	hsa:5148	D03736
Non-interactive	hsa:495	D00434
Non-interactive	hsa:5126	D00969
Non-interactive	hsa:5332	D00653
Non-interactive	hsa:23236	D00515
Non-interactive	hsa:5145	D00283
Non-interactive	hsa:5537	D00155
Non-interactive	hsa:327	D03778
Non-interactive	hsa:115	D03803
Non-interactive	hsa:5156	D02333
Non-interactive	hsa:109	D03775
Non-interactive	hsa:30	D03643
Non-interactive	hsa:5494	D01397
Non-interactive	hsa:28	D00434
Non-interactive	hsa:1845	D03720
Non-interactive	hsa:53938	D00139
Non-interactive	hsa:218	D00651
Non-interactive	hsa:5169	D02556
Non-interactive	hsa:1675	D01441
Non-interactive	hsa:5494	D04029
Non-interactive	hsa:377677	D00902
Non-interactive	hsa:54878	D00155
Non-interactive	hsa:765	D03803
Non-interactive	hsa:6716	D03717
Non-interactive	hsa:115	D02166
Non-interactive	hsa:84695	D03741
Non-interactive	hsa:7046	D02487
Non-interactive	hsa:4860	D00216
Non-interactive	hsa:5423	D00813
Non-interactive	hsa:7173	D04292
Non-interactive	hsa:7010	D00131
Non-interactive	hsa:64802	D00018
Non-interactive	hsa:1562	D01441
Non-interactive	hsa:10327	D00752

Non-interactive	hsa:5650	D02581
Non-interactive	hsa:5646	D02560
Non-interactive	hsa:1800	D03735
Non-interactive	hsa:10269	D00394
Non-interactive	hsa:5148	D03828
Non-interactive	hsa:2042	D01211
Non-interactive	hsa:5331	D00963
Non-interactive	hsa:5831	D00516
Non-interactive	hsa:3290	D00884
Non-interactive	hsa:132160	D00324
Non-interactive	hsa:1890	D00537
Non-interactive	hsa:55775	D02581
Non-interactive	hsa:10941	D00563
Non-interactive	hsa:5144	D03828
Non-interactive	hsa:79001	D01688
Non-interactive	hsa:29920	D03798
Non-interactive	hsa:2639	D02698
Non-interactive	hsa:3028	D00225
Non-interactive	hsa:91	D01765
Non-interactive	hsa:3551	D00298
Non-interactive	hsa:10549	D02769
Non-interactive	hsa:1584	D03728
Non-interactive	hsa:231	D00900
Non-interactive	hsa:8854	D01276
Non-interactive	hsa:10667	D00315
Non-interactive	hsa:54579	D01027
Non-interactive	hsa:4758	D02731
Non-interactive	hsa:11266	D03720
Non-interactive	hsa:10269	D03012
Non-interactive	hsa:5595	D02581
Non-interactive	hsa:247	D03806
Non-interactive	hsa:8854	D00885
Non-interactive	hsa:5550	D01862
Non-interactive	hsa:1636	D02709
Non-interactive	hsa:2064	D01196
Non-interactive	hsa:9945	D03077
Non-interactive	hsa:313	D00218
Non-interactive	hsa:5321	D03882
Non-interactive	hsa:686	D00208
Non-interactive	hsa:57016	D03689
Non-interactive	hsa:8529	D02769
Non-interactive	hsa:284541	D00651

Non-interactive	hsa:1645	D00887
Non-interactive	hsa:11221	D00342
Non-interactive	hsa:4593	D03736
Non-interactive	hsa:1553	D01064
Non-interactive	hsa:2046	D00550
Non-interactive	hsa:93	D00630
Non-interactive	hsa:3615	D00448
Non-interactive	hsa:51095	D00107
Non-interactive	hsa:54878	D01432
Non-interactive	hsa:224	D00781
Non-interactive	hsa:129807	D01688
Non-interactive	hsa:189	D00622
Non-interactive	hsa:1537	D00398
Non-interactive	hsa:5141	D04025
Non-interactive	hsa:2155	D02580
Non-interactive	hsa:1376	D01688
Non-interactive	hsa:53	D03716
Non-interactive	hsa:2064	D03758
Non-interactive	hsa:93650	D00294
Non-interactive	hsa:108	D03899
Non-interactive	hsa:1565	D03826
Non-interactive	hsa:5601	D00893
Non-interactive	hsa:6241	D01690
Non-interactive	hsa:128853	D03826
Non-interactive	hsa:5422	D02451
Non-interactive	hsa:279	D02193
Non-interactive	hsa:5836	D00449
Non-interactive	hsa:5494	D03743
Non-interactive	hsa:8622	D01332
Non-interactive	hsa:64850	D00196
Non-interactive	hsa:3614	D03350
Non-interactive	hsa:11221	D02258
Non-interactive	hsa:2185	D00120
Non-interactive	hsa:34	D01211
Non-interactive	hsa:5141	D02110
Non-interactive	hsa:111	D00139
Non-interactive	hsa:5140	D02214
Non-interactive	hsa:63036	D00391
Non-interactive	hsa:5096	D01866
Non-interactive	hsa:4190	D00005
Non-interactive	hsa:5331	D01866
Non-interactive	hsa:84532	D01973

Non-interactive	hsa:1969	D00884
Non-interactive	hsa:9023	D01973
Non-interactive	hsa:4191	D03899
Non-interactive	hsa:107	D00437
Non-interactive	hsa:3718	D00530
Non-interactive	hsa:5151	D02418
Non-interactive	hsa:128853	D00208
Non-interactive	hsa:55811	D03767
Non-interactive	hsa:1848	D02258
Non-interactive	hsa:2261	D03823
Non-interactive	hsa:4129	D04031
Non-interactive	hsa:132160	D00620
Non-interactive	hsa:10188	D03720
Non-interactive	hsa:10667	D00285
Non-interactive	hsa:84706	D04031
Non-interactive	hsa:7366	D03440
Non-interactive	hsa:9601	D02709
Non-interactive	hsa:5650	D00630
Non-interactive	hsa:4919	D01582
Non-interactive	hsa:64600	D00127
Non-interactive	hsa:3034	D00125
Non-interactive	hsa:1844	D00208
Non-interactive	hsa:57665	D00893
Non-interactive	hsa:5142	D00622
Non-interactive	hsa:2161	D03758
Non-interactive	hsa:26279	D00120
Non-interactive	hsa:54490	D00753
Non-interactive	hsa:3990	D01565
Non-interactive	hsa:767	D00593
Non-interactive	hsa:5122	D00364
Non-interactive	hsa:3612	D00218
Non-interactive	hsa:51205	D03882
Non-interactive	hsa:2677	D01862
Non-interactive	hsa:8622	D00805
Non-interactive	hsa:2639	D01180
Non-interactive	hsa:94009	D00298
Non-interactive	hsa:9023	D00298
Non-interactive	hsa:768	D00198
Non-interactive	hsa:189	D01974
Non-interactive	hsa:5516	D00394
Non-interactive	hsa:114	D00434
Non-interactive	hsa:53938	D01718

Non-interactive	hsa:7294	D01240
Non-interactive	hsa:29920	D00423
Non-interactive	hsa:5322	D02110
Non-interactive	hsa:1267	D00325
Non-interactive	hsa:6646	D03751
Non-interactive	hsa:29920	D00127
Non-interactive	hsa:6725	D00423
Non-interactive	hsa:670	D03736
Non-interactive	hsa:9388	D01984
Non-interactive	hsa:3939	D01885
Non-interactive	hsa:224	D00364
Non-interactive	hsa:4282	D00630
Non-interactive	hsa:5150	D02418
Non-interactive	hsa:8972	D00893
Non-interactive	hsa:762	D00196
Non-interactive	hsa:5049	D02731
Non-interactive	hsa:3988	D00414
Non-interactive	hsa:683	D00252
Non-interactive	hsa:54576	D01984
Non-interactive	hsa:761	D01397
Non-interactive	hsa:3001	D04025
Non-interactive	hsa:115	D02418
Non-interactive	hsa:3815	D01565
Non-interactive	hsa:54577	D00753
Non-interactive	hsa:5625	D02194
Non-interactive	hsa:63904	D02441
Non-interactive	hsa:128	D02698
Non-interactive	hsa:1800	D00515
Non-interactive	hsa:7297	D00771
Non-interactive	hsa:2051	D00963
Non-interactive	hsa:34	D02166
Non-interactive	hsa:3028	D01240
Non-interactive	hsa:64802	D01918
Non-interactive	hsa:586	D00125
Non-interactive	hsa:199974	D02258
Non-interactive	hsa:81579	D01367
Non-interactive	hsa:80339	D01911
Non-interactive	hsa:10056	D00342
Non-interactive	hsa:3291	D03882
Non-interactive	hsa:2185	D00516
Non-interactive	hsa:5479	D00208
Non-interactive	hsa:2322	D00070

Non-interactive	hsa:635	D03806
Non-interactive	hsa:7298	D00324
Non-interactive	hsa:4942	D00002
Non-interactive	hsa:52	D00650
Non-interactive	hsa:6300	D01223
Non-interactive	hsa:1537	D00655
Non-interactive	hsa:2064	D00752
Non-interactive	hsa:3817	D02698
Non-interactive	hsa:5481	D02328
Non-interactive	hsa:5601	D00131
Non-interactive	hsa:1675	D03012
Non-interactive	hsa:5597	D01828
Non-interactive	hsa:4920	D00655
Non-interactive	hsa:2638	D01974
Non-interactive	hsa:10720	D04031
Non-interactive	hsa:5599	D03735
Non-interactive	hsa:5499	D02229
Non-interactive	hsa:116447	D02581
Non-interactive	hsa:3291	D00892
Non-interactive	hsa:54578	D00359
Non-interactive	hsa:80824	D00963
Non-interactive	hsa:132	D03735
Non-interactive	hsa:5126	D00593
Non-interactive	hsa:29968	D00448
Non-interactive	hsa:1056	D02229
Non-interactive	hsa:63036	D01977
Non-interactive	hsa:2766	D00005
Non-interactive	hsa:26279	D01164
Non-interactive	hsa:50484	D00813
Non-interactive	hsa:5327	D00434
Non-interactive	hsa:3718	D00563
Non-interactive	hsa:5140	D00198
Non-interactive	hsa:4200	D03741
Non-interactive	hsa:23430	D02110
Non-interactive	hsa:2155	D02556
Non-interactive	hsa:2185	D00889
Non-interactive	hsa:780	D03828
Non-interactive	hsa:53	D03743
Non-interactive	hsa:23436	D03218
Non-interactive	hsa:5122	D00752
Non-interactive	hsa:1850	D04292
Non-interactive	hsa:6714	D00656

Non-interactive	hsa:2147	D00651
Non-interactive	hsa:5754	D00655
Non-interactive	hsa:2242	D03882
Non-interactive	hsa:125	D00454
Non-interactive	hsa:3939	D00488
Non-interactive	hsa:657	D00563
Non-interactive	hsa:8654	D00283
Non-interactive	hsa:240	D01275
Non-interactive	hsa:645	D00654
Non-interactive	hsa:28227	D00538
Non-interactive	hsa:2066	D00900
Non-interactive	hsa:3614	D01223
Non-interactive	hsa:116447	D01370
Non-interactive	hsa:5320	D01844
Non-interactive	hsa:2263	D01918
Non-interactive	hsa:7371	D00656
Non-interactive	hsa:1849	D00550
Non-interactive	hsa:10279	D01432
Non-interactive	hsa:1565	D00018
Non-interactive	hsa:5053	D01180
Non-interactive	hsa:5148	D00002
Non-interactive	hsa:1200	D00155
Non-interactive	hsa:5096	D00656
Non-interactive	hsa:4758	D02655
Non-interactive	hsa:476	D00753
Non-interactive	hsa:834	D01211
Non-interactive	hsa:64816	D00902
Non-interactive	hsa:5475	D00550
Non-interactive	hsa:25796	D03012
Non-interactive	hsa:5470	D00620
Non-interactive	hsa:7298	D03899
Non-interactive	hsa:5608	D03765
Non-interactive	hsa:2746	D03899
Non-interactive	hsa:4594	D00656
Non-interactive	hsa:8435	D03218
Non-interactive	hsa:2950	D02166
Non-interactive	hsa:326625	D00187
Non-interactive	hsa:1545	D00656
Non-interactive	hsa:762	D00141
Non-interactive	hsa:3028	D02110
Non-interactive	hsa:4919	D01767
Non-interactive	hsa:7075	D03816

Non-interactive	hsa:1847	D00892
Non-interactive	hsa:130399	D01027
Non-interactive	hsa:3283	D00218
Non-interactive	hsa:768	D01275
Non-interactive	hsa:1376	D02731
Non-interactive	hsa:2042	D03720
Non-interactive	hsa:7367	D00652
Non-interactive	hsa:63036	D00414
Non-interactive	hsa:4353	D00900
Non-interactive	hsa:4128	D01688
Non-interactive	hsa:1557	D00771
Non-interactive	hsa:55312	D03826
Non-interactive	hsa:4145	D01718
Non-interactive	hsa:1723	D00218
Non-interactive	hsa:4058	D00892
Non-interactive	hsa:2159	D03806
Non-interactive	hsa:5646	D00187
Non-interactive	hsa:270	D01180
Non-interactive	hsa:7173	D02194
Non-interactive	hsa:5601	D00651
Non-interactive	hsa:64902	D03440
Non-interactive	hsa:1633	D00005
Non-interactive	hsa:4191	D00315
Non-interactive	hsa:130399	D00630
Non-interactive	hsa:5340	D00070
Non-interactive	hsa:5141	D03736
Non-interactive	hsa:5337	D00658
Non-interactive	hsa:1584	D00018
Non-interactive	hsa:5407	D02166
Non-interactive	hsa:5860	D02194
Non-interactive	hsa:1728	D00781
Non-interactive	hsa:1586	D01578
Non-interactive	hsa:1586	D00449
Non-interactive	hsa:11202	D01825
Non-interactive	hsa:1445	D03720
Non-interactive	hsa:1545	D00630
Non-interactive	hsa:5053	D02214
Non-interactive	hsa:501	D00537
Non-interactive	hsa:1845	D01828
Non-interactive	hsa:6897	D00813
Non-interactive	hsa:6300	D01180
Non-interactive	hsa:5604	D01715

Non-interactive	hsa:1579	D02441
Non-interactive	hsa:54579	D02110
Non-interactive	hsa:5096	D00515
Non-interactive	hsa:25796	D00488
Non-interactive	hsa:116447	D04025
Non-interactive	hsa:1990	D02487
Non-interactive	hsa:2328	D00771
Non-interactive	hsa:2936	D03735
Non-interactive	hsa:377677	D03012
Non-interactive	hsa:7535	D00885
Non-interactive	hsa:2045	D00537
Non-interactive	hsa:2766	D01441
Non-interactive	hsa:1553	D00889
Non-interactive	hsa:90	D01240
Non-interactive	hsa:1363	D03720
Non-interactive	hsa:1845	D03805
Non-interactive	hsa:3817	D01397
Non-interactive	hsa:6416	D01578
Non-interactive	hsa:5742	D00325
Non-interactive	hsa:5499	D01397
Non-interactive	hsa:132160	D00654
Non-interactive	hsa:5516	D00884
Non-interactive	hsa:217	D00155
Non-interactive	hsa:686	D04025
Non-interactive	hsa:3615	D00155
Non-interactive	hsa:2321	D00538
Non-interactive	hsa:2321	D02560
Non-interactive	hsa:6098	D00752
Non-interactive	hsa:54	D00656
Non-interactive	hsa:10720	D01332
Non-interactive	hsa:56922	D00651
Non-interactive	hsa:5499	D00530
Non-interactive	hsa:2235	D00252
Non-interactive	hsa:151531	D01915
Non-interactive	hsa:10295	D00423
Non-interactive	hsa:5331	D03803
Non-interactive	hsa:3292	D00188
Non-interactive	hsa:133121	D01061
Non-interactive	hsa:1573	D00005
Non-interactive	hsa:5754	D03717
Non-interactive	hsa:2806	D01715
Non-interactive	hsa:4058	D02258

Non-interactive	hsa:5600	D01196
Non-interactive	hsa:5831	D03758
Non-interactive	hsa:2065	D02214
Non-interactive	hsa:131	D00437
Non-interactive	hsa:5319	D01275
Non-interactive	hsa:5141	D00654
Non-interactive	hsa:5515	D00900
Non-interactive	hsa:8288	D00141
Non-interactive	hsa:2746	D02769
Non-interactive	hsa:285220	D00651
Non-interactive	hsa:327	D01240
Non-interactive	hsa:3615	D01256
Non-interactive	hsa:7372	D01240
Non-interactive	hsa:10846	D00283
Non-interactive	hsa:5837	D02229
Non-interactive	hsa:64902	D03717
Non-interactive	hsa:7015	D00120
Non-interactive	hsa:5145	D00630
Non-interactive	hsa:5478	D00005
Non-interactive	hsa:35	D00892
Non-interactive	hsa:7054	D01432
Non-interactive	hsa:25796	D00537
Non-interactive	hsa:49	D00658
Non-interactive	hsa:2160	D03736
Non-interactive	hsa:11343	D03765
Non-interactive	hsa:55811	D00623
Non-interactive	hsa:9023	D03805
Non-interactive	hsa:2045	D02487
Non-interactive	hsa:4916	D00216
Non-interactive	hsa:5319	D00298
Non-interactive	hsa:1725	D03816
Non-interactive	hsa:5494	D02368
Non-interactive	hsa:54579	D03751
Non-interactive	hsa:1593	D01441
Non-interactive	hsa:270	D00283
Non-interactive	hsa:1571	D00902
Non-interactive	hsa:2597	D03767
Non-interactive	hsa:5747	D01842
Non-interactive	hsa:79001	D01223
Non-interactive	hsa:4023	D03828
Non-interactive	hsa:54575	D01441
Non-interactive	hsa:5052	D00141

Non-interactive	hsa:107	D04025
Non-interactive	hsa:1178	D02560
Non-interactive	hsa:6652	D03758
Non-interactive	hsa:3817	D00391
Non-interactive	hsa:11266	D02214
Non-interactive	hsa:6609	D00900
Non-interactive	hsa:284541	D03350
Non-interactive	hsa:5836	D01765
Non-interactive	hsa:129807	D03728
Non-interactive	hsa:6300	D03899
Non-interactive	hsa:5646	D02756
Non-interactive	hsa:1576	D00623
Non-interactive	hsa:1572	D01196
Non-interactive	hsa:5523	D00225
Non-interactive	hsa:4353	D03735
Non-interactive	hsa:1728	D00203
Non-interactive	hsa:2193	D01565
Non-interactive	hsa:7174	D00005
Non-interactive	hsa:1553	D00449
Non-interactive	hsa:1548	D03805
Non-interactive	hsa:2880	D03728
Non-interactive	hsa:1848	D02418
Non-interactive	hsa:4548	D01432
Non-interactive	hsa:1621	D00593
Non-interactive	hsa:5096	D00449
Non-interactive	hsa:55811	D03775
Non-interactive	hsa:590	D04292
Non-interactive	hsa:129807	D02709
Non-interactive	hsa:5422	D03735
Non-interactive	hsa:11072	D00187
Non-interactive	hsa:1633	D01432
Non-interactive	hsa:5330	D02756
Non-interactive	hsa:3283	D00283
Non-interactive	hsa:6646	D00454
Non-interactive	hsa:111	D03689
Non-interactive	hsa:5605	D01915
Non-interactive	hsa:28227	D00630
Non-interactive	hsa:1636	D00141
Non-interactive	hsa:5126	D00127
Non-interactive	hsa:1384	D01565
Non-interactive	hsa:1445	D00449
Non-interactive	hsa:240	D01180

Non-interactive	hsa:84152	D01862
Non-interactive	hsa:64600	D02008
Non-interactive	hsa:5422	D00454
Non-interactive	hsa:3141	D00653
Non-interactive	hsa:613	D02655
Non-interactive	hsa:7365	D03758
Non-interactive	hsa:1969	D03728
Non-interactive	hsa:8555	D00142
Non-interactive	hsa:55811	D00656
Non-interactive	hsa:55	D02709
Non-interactive	hsa:339221	D00187
Non-interactive	hsa:2041	D02115
Non-interactive	hsa:7294	D01715
Non-interactive	hsa:5049	D01974
Non-interactive	hsa:3033	D00141
Non-interactive	hsa:3991	D03826
Non-interactive	hsa:5335	D00752
Non-interactive	hsa:11202	D02168
Non-interactive	hsa:25796	D01842
Non-interactive	hsa:2235	D01256
Non-interactive	hsa:2159	D01119
Non-interactive	hsa:4353	D00094
Non-interactive	hsa:124	D00434
Non-interactive	hsa:2185	D00519
Non-interactive	hsa:761	D03350
Non-interactive	hsa:476	D00563
Non-interactive	hsa:2597	D00328
Non-interactive	hsa:7054	D00398
Non-interactive	hsa:4051	D00127
Non-interactive	hsa:339221	D00218
Non-interactive	hsa:6098	D03728
Non-interactive	hsa:1595	D00218
Non-interactive	hsa:1543	D02375
Non-interactive	hsa:7301	D00947
Non-interactive	hsa:10461	D01264
Non-interactive	hsa:1621	D03823
Non-interactive	hsa:5516	D00781
Non-interactive	hsa:1584	D01582
Non-interactive	hsa:1200	D00516
Non-interactive	hsa:108	D01984
Non-interactive	hsa:5322	D01767
Non-interactive	hsa:1844	D03778

Non-interactive	hsa:54490	D02655
Non-interactive	hsa:8529	D03440
Non-interactive	hsa:513	D00752
Non-interactive	hsa:2673	D00781
Non-interactive	hsa:6799	D02168
Non-interactive	hsa:686	D01264
Non-interactive	hsa:6609	D00437
Non-interactive	hsa:11266	D00423
Non-interactive	hsa:6897	D03710
Non-interactive	hsa:5604	D00203
Non-interactive	hsa:5169	D01866
Non-interactive	hsa:2261	D00387
Non-interactive	hsa:1849	D00148
Non-interactive	hsa:3815	D00488
Non-interactive	hsa:5145	D01441
Non-interactive	hsa:223	D00630
Non-interactive	hsa:32	D03736
Non-interactive	hsa:11202	D02560
Non-interactive	hsa:4486	D01907
Non-interactive	hsa:6713	D00018
Non-interactive	hsa:2135	D00965
Non-interactive	hsa:4233	D03803
Non-interactive	hsa:134510	D01885
Non-interactive	hsa:50940	D00141
Non-interactive	hsa:18	D03826
Non-interactive	hsa:5600	D03743
Non-interactive	hsa:5743	D00593
Non-interactive	hsa:128	D00537
Non-interactive	hsa:2673	D00893
Non-interactive	hsa:1576	D00530
Non-interactive	hsa:10858	D02756
Non-interactive	hsa:7083	D00893
Non-interactive	hsa:6240	D03734
Non-interactive	hsa:4915	D00753
Non-interactive	hsa:1312	D00622
Non-interactive	hsa:1850	D01885
Non-interactive	hsa:54578	D02042
Non-interactive	hsa:109	D00455
Non-interactive	hsa:11343	D02451
Non-interactive	hsa:9955	D00563
Non-interactive	hsa:1573	D00516
Non-interactive	hsa:377677	D00963

Non-interactive	hsa:3034	D00655
Non-interactive	hsa:27032	D03805
Non-interactive	hsa:3295	D03728
Non-interactive	hsa:5407	D01973
Non-interactive	hsa:5536	D01690
Non-interactive	hsa:6259	D00107
Non-interactive	hsa:326625	D00969
Non-interactive	hsa:2677	D03758
Non-interactive	hsa:2180	D03689
Non-interactive	hsa:54	D00285
Non-interactive	hsa:1636	D01367
Non-interactive	hsa:11266	D03751
Non-interactive	hsa:5834	D00198
Non-interactive	hsa:10846	D02556
Non-interactive	hsa:3932	D04029
Non-interactive	hsa:55359	D00596
Non-interactive	hsa:7525	D00593
Non-interactive	hsa:10327	D00884
Non-interactive	hsa:10188	D00218
Non-interactive	hsa:1843	D02556
Non-interactive	hsa:4920	D02214
Non-interactive	hsa:4145	D03440
Non-interactive	hsa:2548	D03350
Non-interactive	hsa:84152	D02580
Non-interactive	hsa:10825	D00359
Non-interactive	hsa:43	D03734
Non-interactive	hsa:1431	D02769
Non-interactive	hsa:6416	D00448
Non-interactive	hsa:4758	D00120
Non-interactive	hsa:771	D00454
Non-interactive	hsa:30814	D03826
Non-interactive	hsa:51207	D03734
Non-interactive	hsa:586	D01828
Non-interactive	hsa:51181	D01907
Non-interactive	hsa:5335	D00889
Non-interactive	hsa:2595	D00434
Non-interactive	hsa:91039	D01984
Non-interactive	hsa:7298	D00252
Non-interactive	hsa:13	D00454
Non-interactive	hsa:270	D03826
Non-interactive	hsa:54658	D02214
Non-interactive	hsa:5516	D01767

Non-interactive	hsa:35	D03077
Non-interactive	hsa:5336	D00107
Non-interactive	hsa:84532	D01984
Non-interactive	hsa:5144	D01907
Non-interactive	hsa:1589	D02731
Non-interactive	hsa:6416	D02560
Non-interactive	hsa:51172	D01885
Non-interactive	hsa:4051	D00434
Non-interactive	hsa:8940	D00225
Non-interactive	hsa:5607	D00771
Non-interactive	hsa:10295	D03899
Non-interactive	hsa:5594	D00530
Non-interactive	hsa:1890	D03741
Non-interactive	hsa:5331	D01180
Non-interactive	hsa:761	D01370
Non-interactive	hsa:2936	D02769
Non-interactive	hsa:6714	D02418
Non-interactive	hsa:23035	D00125
Non-interactive	hsa:3712	D00141
Non-interactive	hsa:54490	D00070
Non-interactive	hsa:5335	D01441
Non-interactive	hsa:5407	D00437
Non-interactive	hsa:246	D00622
Non-interactive	hsa:64087	D00753
Non-interactive	hsa:1432	D02756
Non-interactive	hsa:8398	D00448
Non-interactive	hsa:327	D01264
Non-interactive	hsa:10188	D00630
Non-interactive	hsa:11266	D00094
Non-interactive	hsa:9023	D01164
Non-interactive	hsa:3716	D00889
Non-interactive	hsa:1645	D01974
Non-interactive	hsa:2746	D03728
Non-interactive	hsa:2639	D00364
Non-interactive	hsa:10188	D00187
Non-interactive	hsa:3988	D00437
Non-interactive	hsa:4329	D00454
Non-interactive	hsa:128	D00463
Non-interactive	hsa:683	D01844
Non-interactive	hsa:30833	D01844
Non-interactive	hsa:2048	D02756
Non-interactive	hsa:1588	D01256

Non-interactive	hsa:5340	D00651
Non-interactive	hsa:1675	D00364
Non-interactive	hsa:7174	D03751
Non-interactive	hsa:5322	D00533
Non-interactive	hsa:11072	D00537
Non-interactive	hsa:1548	D01977
Non-interactive	hsa:6716	D00965
Non-interactive	hsa:3295	D00538
Non-interactive	hsa:247	D04031
Non-interactive	hsa:51181	D02333
Non-interactive	hsa:29920	D00887
Non-interactive	hsa:216	D03735
Non-interactive	hsa:2066	D01397
Non-interactive	hsa:2049	D01918
Non-interactive	hsa:128853	D03218
Non-interactive	hsa:3416	D01718
Non-interactive	hsa:476	D00155
Non-interactive	hsa:29968	D01432
Non-interactive	hsa:124	D03736
Non-interactive	hsa:5159	D00002
Non-interactive	hsa:25796	D00969
Non-interactive	hsa:658	D00620
Non-interactive	hsa:2232	D04292
Non-interactive	hsa:5595	D01397
Non-interactive	hsa:5536	D01180
Non-interactive	hsa:36	D00294
Non-interactive	hsa:444	D00139
Non-interactive	hsa:5126	D00885
Non-interactive	hsa:2263	D00752
Non-interactive	hsa:238	D00018
Non-interactive	hsa:5158	D00285
Non-interactive	hsa:29785	D00650
Non-interactive	hsa:55359	D00630
Non-interactive	hsa:116447	D03758
Non-interactive	hsa:22843	D02709
Non-interactive	hsa:8435	D01688
Non-interactive	hsa:1033	D00563
Non-interactive	hsa:1555	D01027
Non-interactive	hsa:51	D00622
Non-interactive	hsa:5338	D01397
Non-interactive	hsa:5657	D03751
Non-interactive	hsa:5444	D01974

Non-interactive	hsa:5501	D02451
Non-interactive	hsa:2638	D03798
Non-interactive	hsa:5516	D00655
Non-interactive	hsa:5979	D00002
Non-interactive	hsa:586	D00142
Non-interactive	hsa:10825	D02258
Non-interactive	hsa:5624	D00900
Non-interactive	hsa:1852	D00515
Non-interactive	hsa:761	D03712
Non-interactive	hsa:4759	D02581
Non-interactive	hsa:5423	D03738
Non-interactive	hsa:1621	D01264
Non-interactive	hsa:52	D00142
Non-interactive	hsa:1586	D02560
Non-interactive	hsa:100	D00324
Non-interactive	hsa:9023	D00455
Non-interactive	hsa:29785	D00298
Non-interactive	hsa:5146	D00515
Non-interactive	hsa:2880	D00005
Non-interactive	hsa:50	D00515
Non-interactive	hsa:2534	D00131
Non-interactive	hsa:107	D03823
Non-interactive	hsa:51645	D00325
Non-interactive	hsa:1846	D01688
Non-interactive	hsa:260293	D02769
Non-interactive	hsa:780	D03736
Non-interactive	hsa:1360	D02655
Non-interactive	hsa:760	D00884
Non-interactive	hsa:32	D00391
Non-interactive	hsa:4482	D03440
Non-interactive	hsa:3551	D01367
Non-interactive	hsa:3141	D00437
Non-interactive	hsa:84532	D02258
Non-interactive	hsa:657	D03077
Non-interactive	hsa:109	D00448
Non-interactive	hsa:4129	D00387
Non-interactive	hsa:23035	D03788
Non-interactive	hsa:64850	D00315
Non-interactive	hsa:1806	D00398
Non-interactive	hsa:84618	D00127
Non-interactive	hsa:63904	D02168
Non-interactive	hsa:6416	D00285

Non-interactive	hsa:93650	D03882
Non-interactive	hsa:5320	D00155
Non-interactive	hsa:7083	D00538
Non-interactive	hsa:327	D00620
Non-interactive	hsa:6716	D02110
Non-interactive	hsa:695	D00216
Non-interactive	hsa:5740	D00902
Non-interactive	hsa:4058	D03440
Non-interactive	hsa:1376	D00394
Non-interactive	hsa:5495	D00902
Non-interactive	hsa:1849	D02580
Non-interactive	hsa:4145	D00298
Non-interactive	hsa:686	D00187
Non-interactive	hsa:5834	D03775
Non-interactive	hsa:590	D03717
Non-interactive	hsa:1573	D00813
Non-interactive	hsa:5501	D01907
Non-interactive	hsa:4919	D03712
Non-interactive	hsa:5479	D02451
Non-interactive	hsa:5167	D01911
Non-interactive	hsa:5445	D01370
Non-interactive	hsa:2322	D00884
Non-interactive	hsa:8435	D04024
Non-interactive	hsa:8836	D01885
Non-interactive	hsa:63036	D03823
Non-interactive	hsa:50	D02375
Non-interactive	hsa:2193	D01441
Non-interactive	hsa:5754	D00537
Non-interactive	hsa:84171	D00753
Non-interactive	hsa:134510	D03736
Non-interactive	hsa:247	D03765
Non-interactive	hsa:2260	D03758
Non-interactive	hsa:94009	D02556
Non-interactive	hsa:5049	D00414
Non-interactive	hsa:2155	D00324
Non-interactive	hsa:57176	D01907
Non-interactive	hsa:4048	D00813
Non-interactive	hsa:8555	D00423
Non-interactive	hsa:52	D00519
Non-interactive	hsa:51004	D03738
Non-interactive	hsa:354	D01984
Non-interactive	hsa:5596	D03716

Non-interactive	hsa:6714	D02173
Non-interactive	hsa:52	D02194
Non-interactive	hsa:218	D01915
Non-interactive	hsa:8854	D00324
Non-interactive	hsa:3551	D02418
Non-interactive	hsa:5052	D02368
Non-interactive	hsa:10935	D00656
Non-interactive	hsa:5599	D03899
Non-interactive	hsa:5321	D01844
Non-interactive	hsa:377677	D02756
Non-interactive	hsa:107	D00434
Non-interactive	hsa:10188	D00771
Non-interactive	hsa:5601	D02368
Non-interactive	hsa:313	D01256
Non-interactive	hsa:7957	D00969
Non-interactive	hsa:10269	D03710
Non-interactive	hsa:85313	D00567
Non-interactive	hsa:84618	D02581
Non-interactive	hsa:5169	D00148
Non-interactive	hsa:1991	D03787
Non-interactive	hsa:6898	D00752
Non-interactive	hsa:25	D00005
Non-interactive	hsa:150290	D02731
Non-interactive	hsa:495	D01977
Non-interactive	hsa:10747	D00620
Non-interactive	hsa:90	D02042
Non-interactive	hsa:1562	D00463
Non-interactive	hsa:10825	D00753
Non-interactive	hsa:1562	D01164
Non-interactive	hsa:23236	D03350
Non-interactive	hsa:5523	D01690
Non-interactive	hsa:5603	D00963
Non-interactive	hsa:5646	D00454
Non-interactive	hsa:51181	D04292
Non-interactive	hsa:2936	D00805
Non-interactive	hsa:3416	D01918
Non-interactive	hsa:2339	D00622
Non-interactive	hsa:7378	D00045
Non-interactive	hsa:6609	D03816
Non-interactive	hsa:80824	D01825
Non-interactive	hsa:2321	D02581
Non-interactive	hsa:6098	D00655

Non-interactive	hsa:23430	D00900
Non-interactive	hsa:2160	D01715
Non-interactive	hsa:1633	D01164
Non-interactive	hsa:7173	D03728
Non-interactive	hsa:2042	D00414
Non-interactive	hsa:8854	D03778
Non-interactive	hsa:9641	D01164
Non-interactive	hsa:223	D00969
Non-interactive	hsa:8940	D01973
Non-interactive	hsa:4023	D03741
Non-interactive	hsa:5151	D00537
Non-interactive	hsa:1200	D00423
Non-interactive	hsa:7367	D01825
Non-interactive	hsa:1147	D04025
Non-interactive	hsa:5979	D03720
Non-interactive	hsa:63036	D03728
Non-interactive	hsa:55811	D00188
Non-interactive	hsa:51207	D01915
Non-interactive	hsa:23035	D03689
Non-interactive	hsa:128853	D02214
Non-interactive	hsa:199974	D00550
Non-interactive	hsa:4282	D00516
Non-interactive	hsa:1583	D00519
Non-interactive	hsa:4311	D01027
Non-interactive	hsa:3643	D00965
Non-interactive	hsa:22978	D02580
Non-interactive	hsa:5495	D00463
Non-interactive	hsa:1890	D01432
Non-interactive	hsa:7015	D01862
Non-interactive	hsa:766	D00364
Non-interactive	hsa:11330	D02556
Non-interactive	hsa:13	D02731
Non-interactive	hsa:55512	D00218
Non-interactive	hsa:4919	D00218
Non-interactive	hsa:6098	D03735
Non-interactive	hsa:3906	D03882
Non-interactive	hsa:2066	D03767
Non-interactive	hsa:191	D01885
Non-interactive	hsa:5860	D00630
Non-interactive	hsa:1537	D04025
Non-interactive	hsa:2548	D00298
Non-interactive	hsa:635	D00448

Non-interactive	hsa:22954	D02556
Non-interactive	hsa:5330	D02229
Non-interactive	hsa:5445	D01918
Non-interactive	hsa:5475	D03218
Non-interactive	hsa:29968	D01061
Non-interactive	hsa:50487	D00148
Non-interactive	hsa:108	D00394
Non-interactive	hsa:8288	D03734
Non-interactive	hsa:5600	D00394
Non-interactive	hsa:51095	D01918
Non-interactive	hsa:54575	D01765
Non-interactive	hsa:4953	D03736
Non-interactive	hsa:834	D00208
Non-interactive	hsa:444	D00188
Non-interactive	hsa:5152	D03736
Non-interactive	hsa:1571	D03712
Non-interactive	hsa:28972	D02769
Non-interactive	hsa:5537	D02214
Non-interactive	hsa:107	D03077
Non-interactive	hsa:4311	D02556
Non-interactive	hsa:64816	D01578
Non-interactive	hsa:5834	D00781
Non-interactive	hsa:5536	D03882
Non-interactive	hsa:1033	D00398
Non-interactive	hsa:4837	D01974
Non-interactive	hsa:2065	D04031
Non-interactive	hsa:6898	D00463
Non-interactive	hsa:834	D00187
Non-interactive	hsa:2595	D02709
Non-interactive	hsa:4067	D03743
Non-interactive	hsa:80824	D00805
Non-interactive	hsa:686	D01842
Non-interactive	hsa:35	D01275
Non-interactive	hsa:5645	D02333
Non-interactive	hsa:51207	D00387
Non-interactive	hsa:28227	D01133
Non-interactive	hsa:5476	D01984
Non-interactive	hsa:10549	D01915
Non-interactive	hsa:109	D04031
Non-interactive	hsa:660	D00294
Non-interactive	hsa:645	D03826
Non-interactive	hsa:2064	D02655

Non-interactive	hsa:2746	D02193
Non-interactive	hsa:231	D02451
Non-interactive	hsa:3643	D01397
Non-interactive	hsa:5624	D00885
Non-interactive	hsa:5149	D01071
Non-interactive	hsa:23436	D01240
Non-interactive	hsa:225689	D01133
Non-interactive	hsa:1847	D03717
Non-interactive	hsa:1543	D01915
Non-interactive	hsa:55312	D01223
Non-interactive	hsa:4907	D00252
Non-interactive	hsa:306	D03743
Non-interactive	hsa:285220	D03765
Non-interactive	hsa:1585	D00781
Non-interactive	hsa:7015	D03743
Non-interactive	hsa:635	D01180
Non-interactive	hsa:11238	D03717
Non-interactive	hsa:111	D00198
Non-interactive	hsa:354	D02258
Non-interactive	hsa:2356	D00965
Non-interactive	hsa:216	D01765
Non-interactive	hsa:1537	D00187
Non-interactive	hsa:128853	D00298
Non-interactive	hsa:5321	D01885
Non-interactive	hsa:8972	D00455
Non-interactive	hsa:6713	D01211
Non-interactive	hsa:10667	D03751
Non-interactive	hsa:7174	D00515
Non-interactive	hsa:5645	D00187
Non-interactive	hsa:4067	D00414
Non-interactive	hsa:5294	D02655
Non-interactive	hsa:3034	D01828
Non-interactive	hsa:1571	D00315
Non-interactive	hsa:768	D00070
Non-interactive	hsa:10295	D03816
Non-interactive	hsa:5033	D01164
Non-interactive	hsa:3615	D01844
Non-interactive	hsa:1363	D00889
Non-interactive	hsa:1565	D01984
Non-interactive	hsa:4233	D00519
Non-interactive	hsa:4143	D00893
Non-interactive	hsa:5550	D00188

Non-interactive	hsa:5831	D03077
Non-interactive	hsa:11330	D02731
Non-interactive	hsa:2046	D02698
Non-interactive	hsa:339221	D00094
Non-interactive	hsa:64816	D02168
Non-interactive	hsa:7297	D00567
Non-interactive	hsa:9955	D00519
Non-interactive	hsa:5834	D00813
Non-interactive	hsa:2232	D01276
Non-interactive	hsa:6646	D00655
Non-interactive	hsa:5599	D00325
Non-interactive	hsa:3818	D01196
Non-interactive	hsa:1543	D03751
Non-interactive	hsa:1582	D00196
Non-interactive	hsa:5407	D00187
Non-interactive	hsa:3702	D03712
Non-interactive	hsa:4025	D00969
Non-interactive	hsa:5138	D01862
Non-interactive	hsa:23239	D02176
Non-interactive	hsa:1576	D02729
Non-interactive	hsa:5137	D00126
Non-interactive	hsa:5595	D01709
Non-interactive	hsa:4486	D00148
Non-interactive	hsa:1429	D00325
Non-interactive	hsa:142679	D02017
Non-interactive	hsa:55902	D00070
Non-interactive	hsa:5531	D01715
Non-interactive	hsa:5137	D00900
Non-interactive	hsa:5602	D00035
Non-interactive	hsa:1553	D00658
Non-interactive	hsa:9601	D03752
Non-interactive	hsa:2043	D03643
Non-interactive	hsa:7015	D02835
Non-interactive	hsa:349565	D02289
Non-interactive	hsa:1847	D02355
Non-interactive	hsa:8854	D03752
Non-interactive	hsa:5742	D01276
Non-interactive	hsa:53938	D00118
Non-interactive	hsa:4953	D00549
Non-interactive	hsa:2224	D00569
Non-interactive	hsa:151531	D00826
Non-interactive	hsa:7174	D01211

Non-interactive	hsa:134510	D01367
Non-interactive	hsa:5445	D00487
Non-interactive	hsa:306	D00065
Non-interactive	hsa:7299	D00882
Non-interactive	hsa:7083	D00733
Non-interactive	hsa:765	D01180
Non-interactive	hsa:18	D00584
Non-interactive	hsa:7371	D01981
Non-interactive	hsa:1585	D01136
Non-interactive	hsa:6897	D00332
Non-interactive	hsa:80339	D00449
Non-interactive	hsa:771	D01332
Non-interactive	hsa:5594	D00533
Non-interactive	hsa:2135	D02328
Non-interactive	hsa:8854	D00132
Non-interactive	hsa:5481	D03722
Non-interactive	hsa:762	D03736
Non-interactive	hsa:32	D02176
Non-interactive	hsa:9023	D00577
Non-interactive	hsa:2677	D03829
Non-interactive	hsa:760	D03350
Non-interactive	hsa:240	D03077
Non-interactive	hsa:1800	D02068
Non-interactive	hsa:10667	D00251
Non-interactive	hsa:5340	D00455
Non-interactive	hsa:1548	D02355
Non-interactive	hsa:8940	D00049
Non-interactive	hsa:216	D01862
Non-interactive	hsa:1267	D00039
Non-interactive	hsa:51302	D00328
Non-interactive	hsa:124	D03077
Non-interactive	hsa:54578	D00501
Non-interactive	hsa:2155	D00094
Non-interactive	hsa:1846	D00537
Non-interactive	hsa:23239	D02561
Non-interactive	hsa:10858	D00535
Non-interactive	hsa:5126	D00035
Non-interactive	hsa:590	D02356
Non-interactive	hsa:1580	D00827
Non-interactive	hsa:2322	D00596
Non-interactive	hsa:10825	D00530
Non-interactive	hsa:7371	D00496

Non-interactive	hsa:28	D02323
Non-interactive	hsa:4311	D00158
Non-interactive	hsa:224	D00650
Non-interactive	hsa:5330	D00963
Non-interactive	hsa:763	D00829
Non-interactive	hsa:6300	D03765
Non-interactive	hsa:189	D04983
Non-interactive	hsa:5337	D00036
Non-interactive	hsa:2673	D00293
Non-interactive	hsa:90	D03784
Non-interactive	hsa:7153	D02559
Non-interactive	hsa:4190	D02350
Non-interactive	hsa:54490	D01547
Non-interactive	hsa:279	D04292
Non-interactive	hsa:5599	D00127
Non-interactive	hsa:54	D02042
Non-interactive	hsa:1573	D01665
Non-interactive	hsa:587	D00136
Non-interactive	hsa:5476	D01690
Non-interactive	hsa:8399	D00560
Non-interactive	hsa:51251	D01842
Non-interactive	hsa:57016	D00185
Non-interactive	hsa:2321	D05407
Non-interactive	hsa:1593	D00546
Non-interactive	hsa:38	D00418
Non-interactive	hsa:11343	D00037
Non-interactive	hsa:25	D01974
Non-interactive	hsa:7015	D00285
Non-interactive	hsa:2548	D03773
Non-interactive	hsa:25	D01767
Non-interactive	hsa:8398	D00488
Non-interactive	hsa:4914	D04029
Non-interactive	hsa:5294	D01513
Non-interactive	hsa:5494	D02556
Non-interactive	hsa:51365	D00670
Non-interactive	hsa:5515	D00691
Non-interactive	hsa:8398	D02229
Non-interactive	hsa:5052	D00359
Non-interactive	hsa:5599	D00401
Non-interactive	hsa:1559	D03710
Non-interactive	hsa:130399	D01977
Non-interactive	hsa:1432	D00475

Non-interactive	hsa:51292	D02559
Non-interactive	hsa:1436	D00417
Non-interactive	hsa:1991	D03826
Non-interactive	hsa:5168	D03736
Non-interactive	hsa:5445	D03716
Non-interactive	hsa:5333	D01180
Non-interactive	hsa:1890	D00625
Non-interactive	hsa:7367	D00650
Non-interactive	hsa:5606	D00533
Non-interactive	hsa:3001	D02017
Non-interactive	hsa:5051	D00227
Non-interactive	hsa:51181	D01565
Non-interactive	hsa:2321	D01264
Non-interactive	hsa:2049	D03670
Non-interactive	hsa:8836	D03765
Non-interactive	hsa:91039	D03734
Non-interactive	hsa:5340	D00380
Non-interactive	hsa:2356	D02193
Non-interactive	hsa:5447	D00574
Non-interactive	hsa:4128	D01432
Non-interactive	hsa:64802	D03773
Non-interactive	hsa:2673	D00970
Non-interactive	hsa:763	D01565
Non-interactive	hsa:8836	D00410
Non-interactive	hsa:4953	D00328
Non-interactive	hsa:9955	D03763
Non-interactive	hsa:9641	D00109
Non-interactive	hsa:54657	D02579
Non-interactive	hsa:23430	D01180
Non-interactive	hsa:260293	D03012
Non-interactive	hsa:5141	D01180
Non-interactive	hsa:8622	D01276
Non-interactive	hsa:1056	D00501
Non-interactive	hsa:4914	D00027
Non-interactive	hsa:1056	D02709
Non-interactive	hsa:5333	D00054
Non-interactive	hsa:767	D03733
Non-interactive	hsa:54878	D02418
Non-interactive	hsa:7297	D02731
Non-interactive	hsa:25824	D02562
Non-interactive	hsa:1544	D03736
Non-interactive	hsa:2235	D03741

Non-interactive	hsa:5138	D01064
Non-interactive	hsa:2595	D00036
Non-interactive	hsa:8622	D03899
Non-interactive	hsa:3939	D03714
Non-interactive	hsa:2044	D03773
Non-interactive	hsa:686	D03717
Non-interactive	hsa:5533	D02487
Non-interactive	hsa:1595	D03752
Non-interactive	hsa:11266	D00805
Non-interactive	hsa:1543	D03822
Non-interactive	hsa:7299	D00630
Non-interactive	hsa:111	D02323
Non-interactive	hsa:3791	D00359
Non-interactive	hsa:11238	D01915
Non-interactive	hsa:51172	D01136
Non-interactive	hsa:90	D00538
Non-interactive	hsa:3817	D00656
Non-interactive	hsa:219	D00970
Non-interactive	hsa:5151	D00294
Non-interactive	hsa:2534	D00786
Non-interactive	hsa:5330	D01712
Non-interactive	hsa:27032	D02671
Non-interactive	hsa:7173	D01718
Non-interactive	hsa:2147	D00947
Non-interactive	hsa:1178	D05458
Non-interactive	hsa:5045	D00519
Non-interactive	hsa:7173	D00394
Non-interactive	hsa:3615	D01069
Non-interactive	hsa:5126	D00342
Non-interactive	hsa:3945	D02290
Non-interactive	hsa:80824	D03760
Non-interactive	hsa:635	D00900
Non-interactive	hsa:5136	D02304
Non-interactive	hsa:64499	D00542
Non-interactive	hsa:5045	D00141
Non-interactive	hsa:1363	D03773
Non-interactive	hsa:2051	D03829
Non-interactive	hsa:2098	D00566
Non-interactive	hsa:5336	D00785
Non-interactive	hsa:5515	D00781
Non-interactive	hsa:5550	D00097
Non-interactive	hsa:3028	D03717

Non-interactive	hsa:7010	D00065
Non-interactive	hsa:2180	D00494
Non-interactive	hsa:113	D00889
Non-interactive	hsa:5740	D01718
Non-interactive	hsa:216	D00650
Non-interactive	hsa:1376	D01325
Non-interactive	hsa:2041	D00371
Non-interactive	hsa:2050	D00039
Non-interactive	hsa:5651	D01513
Non-interactive	hsa:242	D00521
Non-interactive	hsa:7172	D00298
Non-interactive	hsa:1636	D01425
Non-interactive	hsa:4860	D00398
Non-interactive	hsa:2936	D00785
Non-interactive	hsa:3290	D01275
Non-interactive	hsa:354	D02304
Non-interactive	hsa:6799	D02355
Non-interactive	hsa:5605	D01276
Non-interactive	hsa:7378	D03440
Non-interactive	hsa:2339	D00049
Non-interactive	hsa:5147	D00155
Non-interactive	hsa:4921	D00512
Non-interactive	hsa:10056	D00563
Non-interactive	hsa:284541	D01332
Non-interactive	hsa:390956	D03643
Non-interactive	hsa:1581	D01915
Non-interactive	hsa:313	D01118
Non-interactive	hsa:7372	D01715
Non-interactive	hsa:7301	D00593
Non-interactive	hsa:5321	D00029
Non-interactive	hsa:8972	D01475
Non-interactive	hsa:279	D00005
Non-interactive	hsa:3283	D03738
Non-interactive	hsa:64816	D00827
Non-interactive	hsa:9601	D03753
Non-interactive	hsa:1504	D00579
Non-interactive	hsa:5624	D00359
Non-interactive	hsa:5095	D00577
Non-interactive	hsa:4914	D00417
Non-interactive	hsa:5320	D00126
Non-interactive	hsa:3791	D00752
Non-interactive	hsa:2673	D00543

Non-interactive	hsa:223	D00781
Non-interactive	hsa:5481	D01973
Non-interactive	hsa:51207	D04024
Non-interactive	hsa:57016	D03829
Non-interactive	hsa:2264	D00038
Non-interactive	hsa:51645	D00158
Non-interactive	hsa:10747	D01441
Non-interactive	hsa:1573	D00658
Non-interactive	hsa:4051	D04025
Non-interactive	hsa:5167	D03601
Non-interactive	hsa:9601	D01119
Non-interactive	hsa:1429	D00455
Non-interactive	hsa:84618	D02229
Non-interactive	hsa:5091	D00623
Non-interactive	hsa:260293	D00656
Non-interactive	hsa:150290	D02581
Non-interactive	hsa:11330	D00781
Non-interactive	hsa:29920	D01984
Non-interactive	hsa:2582	D02580
Non-interactive	hsa:3001	D00528
Non-interactive	hsa:5338	D00903
Non-interactive	hsa:150290	D00781
Non-interactive	hsa:94	D02258
Non-interactive	hsa:2263	D02335
Non-interactive	hsa:64802	D01549
Non-interactive	hsa:6241	D03722
Non-interactive	hsa:3718	D00533
Non-interactive	hsa:9601	D00094
Non-interactive	hsa:3055	D00274
Non-interactive	hsa:3939	D03710
Non-interactive	hsa:2185	D00227
Non-interactive	hsa:79799	D01432
Non-interactive	hsa:2950	D03712
Non-interactive	hsa:635	D00107
Non-interactive	hsa:5494	D03775
Non-interactive	hsa:2048	D01665
Non-interactive	hsa:7172	D00132
Non-interactive	hsa:260293	D00217
Non-interactive	hsa:4921	D00131
Non-interactive	hsa:5475	D00145
Non-interactive	hsa:223	D00036
Non-interactive	hsa:240	D00550

Non-interactive	hsa:1846	D02290
Non-interactive	hsa:113	D02562
Non-interactive	hsa:1582	D04025
Non-interactive	hsa:1562	D02579
Non-interactive	hsa:7294	D00315
Non-interactive	hsa:5478	D00620
Non-interactive	hsa:1846	D01325
Non-interactive	hsa:4920	D00168
Non-interactive	hsa:2242	D00217
Non-interactive	hsa:5294	D03643
Non-interactive	hsa:55811	D00186
Non-interactive	hsa:134510	D00362
Non-interactive	hsa:51727	D00014
Non-interactive	hsa:3295	D00904
Non-interactive	hsa:8940	D00283
Non-interactive	hsa:5408	D04197
Non-interactive	hsa:5328	D04197
Non-interactive	hsa:2326	D00566
Non-interactive	hsa:501	D03803
Non-interactive	hsa:5501	D01097
Non-interactive	hsa:2822	D00274
Non-interactive	hsa:1558	D00970
Non-interactive	hsa:5646	D03763
Non-interactive	hsa:64087	D01565
Non-interactive	hsa:3035	D02017
Non-interactive	hsa:5407	D03218
Non-interactive	hsa:7083	D01667
Non-interactive	hsa:1429	D00052
Non-interactive	hsa:1562	D00183
Non-interactive	hsa:3945	D01885
Non-interactive	hsa:10	D01119
Non-interactive	hsa:1178	D00131
Non-interactive	hsa:7299	D00889
Non-interactive	hsa:10747	D00279
Non-interactive	hsa:4921	D00185
Non-interactive	hsa:1846	D01547
Non-interactive	hsa:5152	D02556
Non-interactive	hsa:1728	D01549
Non-interactive	hsa:1594	D00475
Non-interactive	hsa:1595	D00324
Non-interactive	hsa:2098	D05407
Non-interactive	hsa:2051	D04031

Non-interactive	hsa:57665	D01122
Non-interactive	hsa:7365	D01665
Non-interactive	hsa:4593	D00322
Non-interactive	hsa:54659	D03882
Non-interactive	hsa:9945	D00964
Non-interactive	hsa:2235	D02562
Non-interactive	hsa:5536	D03805
Non-interactive	hsa:1890	D00650
Non-interactive	hsa:84171	D01027
Non-interactive	hsa:54878	D00568
Non-interactive	hsa:5742	D00270
Non-interactive	hsa:2160	D03741
Non-interactive	hsa:23239	D02729
Non-interactive	hsa:131	D01974
Non-interactive	hsa:2261	D00410
Non-interactive	hsa:9088	D00142
Non-interactive	hsa:4058	D00203
Non-interactive	hsa:4907	D00900
Non-interactive	hsa:93650	D00153
Non-interactive	hsa:84532	D00624
Non-interactive	hsa:3735	D04029
Non-interactive	hsa:5646	D00414
Non-interactive	hsa:4759	D00109
Non-interactive	hsa:1633	D01690
Non-interactive	hsa:8529	D00160
Non-interactive	hsa:4025	D02556
Non-interactive	hsa:6300	D00550
Non-interactive	hsa:501	D02333
Non-interactive	hsa:2263	D00324
Non-interactive	hsa:2806	D00324
Non-interactive	hsa:5033	D00252
Non-interactive	hsa:5657	D00530
Non-interactive	hsa:64499	D02698
Non-interactive	hsa:2051	D02562
Non-interactive	hsa:1990	D02441
Non-interactive	hsa:128	D02229
Non-interactive	hsa:4233	D02333
Non-interactive	hsa:1581	D01888
Non-interactive	hsa:100	D00043
Non-interactive	hsa:231	D00234
Non-interactive	hsa:9647	D00070
Non-interactive	hsa:7365	D00887

Non-interactive	hsa:50	D00650
Non-interactive	hsa:4758	D02194
Non-interactive	hsa:13	D00333
Non-interactive	hsa:2342	D01715
Non-interactive	hsa:18	D00434
Non-interactive	hsa:240	D00032
Non-interactive	hsa:196883	D00630
Non-interactive	hsa:5601	D00027
Non-interactive	hsa:1584	D03735
Non-interactive	hsa:10720	D00994
Non-interactive	hsa:2044	D01183
Non-interactive	hsa:4920	D00153
Non-interactive	hsa:58190	D02176
Non-interactive	hsa:349565	D02194
Non-interactive	hsa:1803	D01915
Non-interactive	hsa:5562	D00579
Non-interactive	hsa:10188	D03763
Non-interactive	hsa:6259	D03769
Non-interactive	hsa:2880	D01475
Non-interactive	hsa:8192	D02441
Non-interactive	hsa:3480	D01276
Non-interactive	hsa:1633	D01981
Non-interactive	hsa:27032	D00136
Non-interactive	hsa:2235	D03743
Non-interactive	hsa:111	D01844
Non-interactive	hsa:1991	D01907
Non-interactive	hsa:5747	D00340
Non-interactive	hsa:5979	D00998
Non-interactive	hsa:55312	D00733
Non-interactive	hsa:10825	D01183
Non-interactive	hsa:2135	D01974
Non-interactive	hsa:5530	D01981
Non-interactive	hsa:5470	D00183
Non-interactive	hsa:683	D00537
Non-interactive	hsa:1363	D00505
Non-interactive	hsa:3702	D00186
Non-interactive	hsa:5594	D01346
Non-interactive	hsa:22954	D03823
Non-interactive	hsa:64499	D03741
Non-interactive	hsa:23236	D01276
Non-interactive	hsa:1675	D03720
Non-interactive	hsa:5625	D03803

Non-interactive	hsa:4051	D01332
Non-interactive	hsa:5091	D00887
Non-interactive	hsa:2339	D00045
Non-interactive	hsa:1555	D03805
Non-interactive	hsa:224	D02290
Non-interactive	hsa:51292	D00545
Non-interactive	hsa:7153	D01582
Non-interactive	hsa:6646	D00593
Non-interactive	hsa:5595	D03440
Non-interactive	hsa:353	D02441
Non-interactive	hsa:4190	D01984
Non-interactive	hsa:100	D02564
Non-interactive	hsa:3716	D00285
Non-interactive	hsa:1621	D02769
Non-interactive	hsa:3643	D03752
Non-interactive	hsa:6098	D00196
Non-interactive	hsa:3615	D03826
Non-interactive	hsa:1583	D00041
Non-interactive	hsa:1675	D01828
Non-interactive	hsa:5136	D02560
Non-interactive	hsa:10549	D00032
Non-interactive	hsa:1969	D04029
Non-interactive	hsa:3614	D01136
Non-interactive	hsa:3002	D00041
Non-interactive	hsa:8398	D00065
Non-interactive	hsa:51172	D00434
Non-interactive	hsa:231	D00398
Non-interactive	hsa:5156	D01966
Non-interactive	hsa:4593	D01211
Non-interactive	hsa:7046	D00703
Non-interactive	hsa:6652	D00007
Non-interactive	hsa:5479	D00691
Non-interactive	hsa:56922	D01840
Non-interactive	hsa:1728	D00998
Non-interactive	hsa:5332	D00521
Non-interactive	hsa:23475	D02729
Non-interactive	hsa:5141	D04029
Non-interactive	hsa:660	D00387
Non-interactive	hsa:9955	D00709
Non-interactive	hsa:1633	D01966
Non-interactive	hsa:5406	D03034
Non-interactive	hsa:5050	D00094

Non-interactive	hsa:10935	D02315
Non-interactive	hsa:2180	D00333
Non-interactive	hsa:7299	D02451
Non-interactive	hsa:5335	D00691
Non-interactive	hsa:196883	D00691
Non-interactive	hsa:1384	D01667
Non-interactive	hsa:10924	D00410
Non-interactive	hsa:10941	D01228
Non-interactive	hsa:56922	D01665
Non-interactive	hsa:63904	D03712
Non-interactive	hsa:657	D00475
Non-interactive	hsa:7525	D00423
Non-interactive	hsa:260293	D00459
Non-interactive	hsa:100	D01888
Non-interactive	hsa:5494	D00118
Non-interactive	hsa:91	D00300
Non-interactive	hsa:3906	D00703
Non-interactive	hsa:51302	D00726
Non-interactive	hsa:5095	D01275
Non-interactive	hsa:81579	D00892
Non-interactive	hsa:7054	D02835
Non-interactive	hsa:767	D02355
Non-interactive	hsa:5651	D01977
Non-interactive	hsa:9088	D02564
Non-interactive	hsa:5837	D00882
Non-interactive	hsa:759	D01432
Non-interactive	hsa:4548	D03670
Non-interactive	hsa:8555	D02451
Non-interactive	hsa:3033	D00904
Non-interactive	hsa:3791	D00391
Non-interactive	hsa:7364	D00340
Non-interactive	hsa:760	D03689
Non-interactive	hsa:1969	D00387
Non-interactive	hsa:2597	D00274
Non-interactive	hsa:131	D00103
Non-interactive	hsa:1549	D02258
Non-interactive	hsa:3028	D01276
Non-interactive	hsa:6652	D00709
Non-interactive	hsa:223	D00070
Non-interactive	hsa:695	D00449
Non-interactive	hsa:3615	D00328
Non-interactive	hsa:7371	D00653

Non-interactive	hsa:8836	D00452
Non-interactive	hsa:5595	D03767
Non-interactive	hsa:6898	D03826
Non-interactive	hsa:3712	D00434
Non-interactive	hsa:5159	D00826
Non-interactive	hsa:109	D01425
Non-interactive	hsa:7010	D02562
Non-interactive	hsa:5319	D00139
Non-interactive	hsa:55811	D01071
Non-interactive	hsa:5501	D03743
Non-interactive	hsa:51302	D02166
Non-interactive	hsa:5137	D03440
Non-interactive	hsa:3988	D01441
Non-interactive	hsa:1384	D01198
Non-interactive	hsa:5533	D03828
Non-interactive	hsa:91039	D00391
Non-interactive	hsa:5523	D03781
Non-interactive	hsa:2638	D00653
Non-interactive	hsa:4953	D01765
Non-interactive	hsa:217	D01718
Non-interactive	hsa:2264	D03788
Non-interactive	hsa:5122	D00656
Non-interactive	hsa:54577	D00380
Non-interactive	hsa:7298	D01918
Non-interactive	hsa:3612	D00566
Non-interactive	hsa:55312	D00452
Non-interactive	hsa:4837	D01064
Non-interactive	hsa:2534	D00902
Non-interactive	hsa:1537	D02229
Non-interactive	hsa:109	D03751
Non-interactive	hsa:5860	D00018
Non-interactive	hsa:5095	D02560
Non-interactive	hsa:5743	D01715
Non-interactive	hsa:5599	D00562
Non-interactive	hsa:93	D00752
Non-interactive	hsa:5330	D00651
Non-interactive	hsa:10825	D03722
Non-interactive	hsa:4759	D00960
Non-interactive	hsa:4837	D00654
Non-interactive	hsa:1585	D00650
Non-interactive	hsa:10747	D01709
Non-interactive	hsa:657	D03218

Non-interactive	hsa:6652	D00283
Non-interactive	hsa:5406	D01367
Non-interactive	hsa:5516	D02323
Non-interactive	hsa:57176	D03433
Non-interactive	hsa:11330	D00298
Non-interactive	hsa:5340	D00196
Non-interactive	hsa:5602	D00279
Non-interactive	hsa:1544	D02729
Non-interactive	hsa:4482	D00533
Non-interactive	hsa:54600	D00650
Non-interactive	hsa:23475	D01984
Non-interactive	hsa:7367	D03722
Non-interactive	hsa:5532	D00829
Non-interactive	hsa:5050	D01907
Non-interactive	hsa:5033	D03743
Non-interactive	hsa:7366	D03601
Non-interactive	hsa:3295	D00889
