# Supplementary material for: iEzy-Drug: A Web Server for Identifying the Interaction between Enzymes and Drugs in Cellular Networking
Source: Biomed Res Int. 2013 Nov 26;2013:701317. doi: 10.1155/2013/701317 (PMC3858977; doi:10.1155/2013/701317)
Supplement: Supplementary file 2 [file 701317.f2.pdf]

Online Supporting Information S2. The fingerprints for the drug codes listed in Online Supporting Information S1. Each of these fingerprints is a 256-D vectors generated by the OpenBabel software downloaded from http://openbabel.org/. See the text of the main paper for further explanation.

```
>D00120
00080200  01841200  00920b81  02910200  60300000  00802013
810010c1  00000403  00000840  00f829c0  08180300  7000b008
04008000  00211000  80280006  0008000e  040c0400  02100080
40600088  00102801  80100110  18000a00  0180c840  08400019
01000800  80200001  80402000  c0200021  005000a0  08028006
04000040  05010620

>D00118
00000000  00000008  20000108  00010200  00001040  00000000
02000000  00000000  00000000  00080840  08000000  40009000
01008002  00000000  41040001  00080008  08000000  02000000
00000000  00020000  00000000  08000a08  01000000  00400010
00000000  80000000  00000000  00000000  00000200  00020002
00002000  00000400

>D00109
04000000  00000808  20000108  00010200  00001000  00024000
02000000  00000800  00000100  00000840  00000000  40008020
02808002  00000000  01010001  0008200c  00020000  02008000
00000000  00420000  00000000  08020a20  10000000  00400004
00000000  80000000  00000800  00000001  00000300  00020000
c0004000  10000000

>D00107
00050040  81005000  00900100  80810700  803d00c0  00200800
02002840  28000442  04002040  011000a8  40202000  5041f000
1602802a  02880002  80081408  28289008  10a20000  80000000
33c02010  00002201  00000120  10040e10  17008080  0810001c
10201401  00000400  00c04000  b0000021  00000200  8004000b
24224000  00802210

>D00103
00040000  00000000  00000000  00000000  00200000  00000000
00000000  00000000  00000000  00000000  00000000  40005000
14008000  00000000  00000000  00080008  00000000  00000000
01400000  00000001  00000000  00000a00  05000000  00000010
00000000  00000000  00000000  00000000  00000002  00000000
00000000  00000000
```

>D00097
00000000 00000808 20000100 00010200 00000000 00024000
02000000 00000800 00000100 00000840 00000000 40008000
00008002 00000000 00000000 00082008 00000000 02000000
00000000 00020000 00000000 08020a20 00000000 00400004
00000000 80000000 00000000 00000000 00000000 00020000
c0000000 00000000

>D00094
00000400 01040020 00000000 00000000 40000000 00000001
00000000 00000402 01004000 00100000 00000000 40000000
00028000 20020004 00000400 0008000c 00000000 00000020
02092090 00000001 00000000 00000a00 00408000 00000012
00201000 00000000 00404000 80000000 00000000 00000000
24820000 00000000

>D00070
1003c00a 01a05800 00180902 98010e10 001a10c0 0010c000
0000f004 22001801 16400100 000208e0 41210004 4000b000
06008822 02408000 008e0440 000c010c 18040000 0200c000
08400000 40200201 40100080 1c068e40 23810840 0070001c
00140000 c0000001 68008000 b0040084 0000c10c 00461801
e0044000 80800a80

>D00065
00010000 01000000 00000000 00000400 00080000 00000000
00000000 00000000 00000000 00000080 00000000 40003000
04008000 00000000 00000000 00080008 00000000 00000000
00400000 00000001 00000000 10000a00 03000000 00000010
00000000 00000000 00000000 00000000 00000000 00040000
00000000 00000000

>D00055
00000002 00001800 00000100 02040600 00000000 0000c000
00000800 00000404 06400300 40040020 04080004 40000800
00008000 00700000 00220000 000c000c 00008000 03008000
00400000 00000000 00000000 00000a00 20800040 00100104
00040000 c0000080 20000028 00040010 00004004 08420001
c0800000 000000a0

>D00054
0000000a 01c01800 80080000 80010600 003800e0 0000c140
00008c04 00000000 06400100 818000b0 0c00800c 40018000

00009000 03c80100 00081080 001ec00c 00200020 00008000
19c00000 00001001 80080120 10000050 27800040 0c300014
10000400 c0200002 60000200 10040070 00004200 00460000
c0280000 00000280

>D00052
00000000 00000000 00000000 04000400 00000080 00000000
00000000 00000000 00000000 00000000 00202000 40002000
02008002 00002003 00300000 00080008 10000000 00000000
00000000 00000000 00000000 20000a00 02000000 02000000
00000000 00000000 00000000 00000000 08000000 00040000
00004000 00000200

>D00050
0000002a 01800800 80080100 02050600 003c0060 0000c040
00008006 00000000 06400100 808008e0 0c00000c 40018000
80008800 01e00000 00ac00c0 000cc00c 08000000 0280c004
09e00000 00000001 c0000080 18008070 27800040 84700014
00040000 c0000000 60008040 00040030 00404000 00460040
c00c0000 000002a0

>D00049
00000002 00800800 00004100 00000210 00041000 00004000
00000000 00000000 00000100 00000040 00000004 40000000
00008800 00000000 00000000 000c000c 00000000 02000004
00200000 00000000 c0000000 08048a40 20800000 0040000c
00040000 00000000 08000000 00000000 00400000 00020000
c0000000 00000000

>D00045
0000000a 01c01800 80080000 88090620 003800e0 0000c000
00008c00 00000800 00400100 018000b0 0c00800c 40019004
00001000 03880100 00081080 101ec00c 00200020 00000000
19c00000 80201001 80080020 10000050 27800040 0c000014
10000400 40000002 00000200 10000070 00000208 00460000
c0280000 000000c0

>D00043
00000001 00000000 00000000 00000000 00000004 00080000
40004000 00000000 00000000 00000000 00000000 40000000
00000000 00020020 00000000 00080008 00000060 00800000
20000000 00000002 00000000 04000800 00000000 00000010
00000000 00000000 02000000 00010000 00010000 00008000
00000000 00008200

>D00041
00000000 00000000 00000000 00000400 00000000 00000000
00000000 00000000 00000000 00000000 00000000 40003000
14008000 00000000 00000000 00080008 00000000 00000000
01400000 00000001 00000000 10000a00 03000000 00000010
00000000 00000000 00000000 00000000 00000000 00040000
00000000 00000000

>D00039
00000000 00000000 00000000 00000400 00000000 00000000
00000000 00000000 00000000 00000000 00000000 40003000
04008000 00000000 00000000 00080008 00000000 00000000
00400000 00000001 00000000 10000a00 03000000 00000010
00000000 00000000 00000000 00000000 00000000 00040000
00000000 00000000

>D00038
00000000 00000800 00000100 00010200 00000000 00004000
00000000 00000000 00000100 00000840 00000000 40008000
00008000 00000000 00000000 00080008 00000000 02000000
00000000 00000000 00000000 08020a20 00000000 00400004
00000000 80000000 00000000 00000000 00000000 00020000
c0000000 00000000

>D00037
00050000 01000000 00000000 00000000 00280000 00000000
00000000 00000000 00000000 00020000 00000000 40005000
14008020 02000000 00000000 00080008 00000000 00000000
01400000 00000001 00000000 00000a00 05000000 00000010
00000000 00000000 00000000 00000000 00000002 00000000
00000000 00000000

>D00036
00000002 00808800 00004100 00000610 00041000 00000000
00004000 00001000 00000000 00000040 01000004 40000000
00008800 00000000 00000000 00040004 00000000 02000004
00200000 00000000 c0000000 08048600 20800000 0040000c
00040000 00000000 08000000 00000000 00400000 00060000
80000000 80000000

>D00035
00010000 01000000 00100000 00000400 00080080 00000010
00000000 00000000 00000000 00000080 00200000 40013000

06008012 01000000 80080000 00080008 10000000 00000000
00400000 00000201 00000000 10000a00 03000000 00000010
00000000 00000000 00000000 00000000 00000000 00040000
00004000 00000200

>D00032
00000002 00000000 00000000 01800410 04000040 00000000
00002000 00000000 00400900 00090200 20008000 50013800
04008000 00100800 00020000 000c000c 00000000 0e000000
00400018 40000001 00000c00 10000a00 230000c0 00000010
00041001 00000200 00000000 00000000 04000004 00460000
40000000 20000000

>D00029
00010000 47003000 20100000 00100c40 00080000 00000000
00000004 00002000 04200000 020040c0 00200000 40071000
0401900c 00840100 00000000 00080008 04001000 00002000
00402060 00000101 c0000100 30050e10 01000300 08300018
00010000 00000000 00004000 10000020 08000000 00040021
000a0000 03010200

>D00027
00000002 00000000 00000000 02000600 00000000 00004000
00000000 00000000 06000100 00000020 04000004 00000000
00008000 00400000 00200000 000c0004 00000000 00008000
00400000 00000000 00000000 00000000 20800040 00100000
00040000 c0000000 20000000 00040000 00004000 00420000
40000000 00000080

>D00021
00000000 00000000 00000100 00010600 00000000 00000000
00000000 00000000 00000800 00080840 08100000 4000b000
04008000 00000000 00000000 00080008 00000000 02000000
00400008 00000001 00000100 18000a00 03000040 81400010
00000800 80000000 00000000 00000000 04000000 00060002
00000000 00000400

>D00018
00040008 80000000 00000500 10010200 00600040 00000000
02000280 28001400 01000000 01500000 00000300 4000c000
88028000 00000001 00080004 000a8008 00020010 00000000
01454010 00002001 00000020 01000a80 05008000 08000010
00001400 00000400 00400000 80000020 00014200 00000008
14000000 00800010

>D00014
00030000 01006000 00100000 04100400 00080080 00000000
00000000 20000008 00200000 02020080 00200000 40033000
06008032 01042001 80180000 00080008 10000000 00000000
00400000 00400301 40000000 30000e00 03000201 06100018
00000000 00000000 00000000 10000000 08000000 00040021
20004000 03010200

>D00007
00030000 01000000 00000000 00000400 00180000 00000000
00000000 00000000 00000000 00020080 00000000 40003000
04008020 02000000 00000000 00080008 00000000 00000000
00400000 00000001 00000000 10000a00 03000000 00000010
00000000 00000000 00000000 00000000 00000000 00040000
00000000 00000000

>D00005
0000002a 01c01800 80480510 8b0d0e20 003c00e4 0010c040
0080cc06 00000800 06404100 818008f0 0c00800c 40019004
80009800 03ea0120 04ac10c0 101eca0c 092a0020 0280c00e
19e00000 80205003 c00800a0 1c008870 27800040 8c700014
50040400 c0000102 6e008240 10050070 00404208 00460054
c16c0000 4000a2e2

>D00002
0800000a 01c09800 80484510 89090e30 003c10e4 0010c000
0080cc00 00001800 00404100 018000f0 0d00800c 40019004
00009800 038a0120 04081080 101eca0c 012a0020 8200000e
19e02000 80205003 c4080020 1c148e50 27800040 0c50001c
50040400 40000102 0e000200 10050070 00400208 40460054
c1780000 c000a0c2

>D00300
00000000 00000000 00000100 80010600 00000090 00004000
00000000 01000000 00000100 00080840 08201000 40008000
00000000 00010000 00001008 02080008 00000000 02000000
00000000 00000000 00000000 08000020 12000000 00400010
00000400 a0002000 00080000 40000000 00000280 00060002
40000000 00000600

>D00298
00000040 81000000 00000100 00810300 80280040 00000800
00002880 08000440 01202000 01100028 40000100 40008000

00028000 02800001 00080400 00289008 00820010 00000000
1341e010 00002001 00100020 01000a80 05008000 08000010
00001400 00000400 00404000 a4000020 00000200 01000002
24a20000 00800010

>D00294
02008016 00822008 21000000 01b10600 00080003 00010210
10000010 00002c01 00800010 00080843 80006004 62008840
0202000c 40000209 802000c0 00000008 21002d00 00000000
03500400 01046000 c0002000 39010102 20000004 08520008
02086000 08100044 41880420 08000c2c 00080400 22860000
04004100 06130160

>D00293
0000c002 06440000 00200160 0c010600 40000062 04080164
14000002 01240005 00000300 20090e45 08280024 4000ac00
02108006 00108800 80060140 00180002 0224c800 03400003
08008000 00000000 40102010 3c000740 20002003 00400012
10002881 a0000000 40080018 45001080 08088098 00068403
c0000080 22020600

>D00285
18000013 42802008 211a018a 4ab90610 0a00101a 00484040
4204a000 01002800 00460100 00080840 28002004 6000b000
00044602 20001041 812a1041 000c800e 00012400 02000405
00480000 50326a01 c0802000 2e001c00 a0840840 084a0098
00040000 80000000 02120020 4841004d 0000068a 2046000a
40000000 600104e0

>D00283
08208246 01840000 00100100 0c210600 00020001 80100004
18c00010 02301044 10012700 8003c843 12202004 44018c30
0200080e c010a809 040604c0 18180080 04658808 13084002
09000002 30801200 40022000 38028100 e3004020 02401000
01082000 c8000180 42880410 9d011880 08888918 01a60000
40040081 52020200

>D00279
04008000 00000008 20000008 00010200 00001000 00000000
02000000 00000040 00002000 00000840 00040000 4000d800
02808006 00000000 01200001 00080008 00020000 80000400
00000002 00020000 00000020 08000b00 05000000 08000010
00002400 00000000 00100008 00800020 00000200 00020000
00000000 00020000

>D00274
0400800a 89005c08 20100108 82010700 00101080 02020110
06000000 22001400 00000000 001009e8 08200004 50018800
02808806 08001000 81081441 00082008 10200000 02000400
00c00000 00420601 40000000 180a8700 33001080 0050001c
00002001 c0000400 00900a00 90000001 00000300 00068811
a4040000 00022a40

>D00270
0020c002 00820200 00120000 01100600 20040000 08002000
08010010 00000000 00022000 00400d60 00244004 61008800
02000004 00000001 20020040 02000002 04841000 20000000
08200002 02024000 e2100000 18000900 20800000 00180019
00006401 10000100 12204010 00000080 00008200 01060010
0000a000 00030220

>D00252
00000006 00000000 42000100 00010e00 00000000 04000002
80000090 00122449 00002004 00400840 00280104 40008000
00008000 00100000 80020042 00020002 00044610 02400000
08001002 00000100 40200000 18060400 20022010 00408000
00100800 82000000 02432018 40009280 00408000 00260c21
04000084 00004200

>D00251
00010000 01005000 00100000 00100400 00080080 00000010
00000000 20000000 00000000 00000080 00200000 40013000
06008012 01040000 80080000 00080008 10000000 00000000
00400000 00400201 40000000 10040e00 03000200 00100018
00000000 00000000 00000000 10000000 00000000 00040021
20004000 01012200

>D00234
00000006 01800608 20100108 02010600 000c1040 00004012
02048000 20101000 00100190 200809e0 0920800c 50018000
00001802 00401000 012000c1 000c000c 00000000 02004100
00600000 00b20201 c0000000 5c208030 21800841 81700010
00000000 80000000 02128000 10000001 05000218 0046981a
400c1000 00002680

>D00231
00002002 00800800 00004100 00000230 00041004 04000040
00000000 00000000 06000000 40000060 04000004 60000000

80018800 00200000 00100000 80040004 00020000 0200000c
04200000 00000002 40000000 88028000 30804000 00500418
00040001 40080000 08000200 00000040 00400000 00460000
00000000 000000a0

>D00227
0000002a 00801800 00080000 02010600 00080040 0000c000
00008000 00000000 06400100 800000a0 0c00800c 40000000
00009000 00400000 00280080 000c000c 00800000 00008000
00400000 00000000 80000100 30000040 21800041 00300004
00040000 c0200000 60000000 00040000 00004000 00460000
c0000000 00000280

>D00225
0080800a 06840019 402c0500 98010780 41000040 00880026
14840000 01201401 10000300 00090845 09010134 44068c00
02100884 08088a08 80068940 080c258c 0a208800 020a0207
00600408 00260000 40122000 2801a100 61001002 08508110
10802010 e00005c0 40084040 45021001 0000009c 00860c02
c00c0080 a4360400

>D00224
0000400a 00000018 60001000 00000600 00080040 0000c008
04000000 00000000 00000100 00000040 01000004 08000000
00000006 00000000 00000000 000c000c 00000000 00000008
00600000 40260000 40020000 08000020 21800840 00000000
00104000 00000000 08020000 00000000 00000000 00420000
40000000 082004a0

>D00222
0000000a 00c01800 00080000 80000600 00080040 0000c100
0000bc04 00000800 06400100 810000f0 0c00800c 40009000
00009000 00480000 00081080 101c000c 00200020 00008000
10400000 00201000 80080100 1c000040 25800840 08300804
00000000 c0200002 60000200 10040040 00004208 00461000
c0200000 80000280

>D00219
00000010 01801800 01100100 02b10e00 00000008 00000000
08000000 00003000 00000000 000828c0 08202000 e0018400
00009000 00000041 80200000 00000000 02002000 02000001
02402000 00006001 80002000 18070610 00000000 0850001c
00000000 80000000 80000000 1000042c 00000400 20060003
80080000 00010660

```
>D00218
00440000  00040009  00000040  00380400  00000000  00800401
00028100  10000010  00000100  10808002  00302000  40000480
90008404  00060001  80042000  0000400d  40000000  00004200
00020020  00204000  00000080  00301690  20400000  00008000
20000000  2c000000  24000004  0001044c  10000008  00050009
00040200  00010268

>D00217
00004002  00000008  20000000  00000600  00000000  00000000
02000000  00000000  00000000  00000840  00280004  40008000
00008022  00100000  00000040  00000008  00040000  00000000
08000000  00020000  40100000  08000600  20002000  00000000
00000000  00000000  00040000  00000080  00008000  00060001
00000000  00000200

>D00216
00000050  81008020  00101800  80810500  803800c0  00000000
00103800  28000442  00206000  011000a8  00200200  c0018000
00020102  02810004  0028140c  002b9108  30800040  00000120
13c1a010  00002201  00100020  10000010  17008080  08100010
10001401  00008c00  00484200  b4000021  00000202  00040000
04a20000  02002a00

>D00208
00470002  09400421  00100910  04490f00  401c0080  04338014
000803a1  a2000402  11800600  01100188  00300600  4501b020
0e02821a  01812101  80101414  001a0048  10a34000  01080166
01e72010  00028221  00000801  b0001ef0  1300a0e1  02100a10
04a00007  20000002  00426a00  90040521  1c100200  0044141e
a6034004  40802a00

>D00203
00050440  81040000  00000100  80810700  c03c0140  00a00001
02002800  08000400  00200000  011000a8  40000200  40009000
1c028028  028a0000  00000404  002a900c  00a20000  00000000
31c0a090  00002001  00100020  10000a00  07408000  08000012
00001400  00000000  00404000  a4000020  00000240  0004000a
24e20000  04000010

>D00198
0005000b  4100c808  2210050a  51010f10  203c1020  00300002
c7042080  22003449  01c02880  010908d0  48318004  50919000
```

1c00c8da 01e08000 01020463 020c910c 00820600 06000126
3369a418 00060a01 40120120 18218a18 25804001 99500010
00143400 c0000001 08426200 d00004a0 04400306 0086010a
24060200 20246f30

>D00196
00004026 42000208 60100108 00010e00 20001000 c0002012
02002004 00002009 04200a80 000808c0 08300004 40028000
00009222 20000000 01030141 00140008 00068000 82400020
08080018 00020241 40100100 18218c10 30020000 81402011
00151000 80010002 00040000 90001080 04008200 0006080b
00080080 30004600

>D00188
00000400 81040000 00000000 00000000 40080000 00000001
00000000 00000400 00000000 00100008 00000200 40000000
00020000 00800000 00000404 0008000c 00000000 00000000
02402080 00000001 00000000 00000000 01008000 00000012
00000000 00000000 00404000 80000000 00000000 01000000
24820000 04000000

>D00187
00000400 81040000 00000000 00000000 40080000 00000001
00000000 00000402 00000000 00100008 00000200 40000000
00020000 00800000 00000404 0008000c 00000000 00000000
02402080 00000001 00000000 00000000 01008000 00000012
00200000 00000000 00404000 80000000 00000000 01000000
24820000 04000000

>D00186
00004002 00809800 00104320 06010610 00041002 0000c070
00008000 00000000 02000100 c0000ac0 00200004 50018000
00008802 01603000 000000c0 000c000d 00040400 82004004
08200000 00400000 c0100000 38068a78 60800024 8250001e
00040000 80000000 08008080 10040081 08408000 00078040
c0040000 00000240

>D00185
00000000 63000008 20000102 00010200 00080000 40000002
02000000 00000001 00000800 00080840 08100000 40008000
00000002 02a00000 00000020 00080008 00000200 02000000
02402000 00020201 00000100 08000000 05004000 00400010
00000000 80000001 00120000 00000000 00000002 01020002
00020000 00000400

>D00184
00010000 81007000 00100000 04000400 00000080 00000000
00000000 20000408 00000000 00100080 00200000 40013000
1602800a 00002001 80180400 00080008 10000000 00000000
00c12000 00002201 00000000 30040610 03008083 02100018
00000001 00000000 00404000 90000000 08000000 00040001
24004000 00002200

>D00183
00000002 01809800 00004100 10010610 00041022 0000c040
00000000 00000000 02000100 c00000c0 0100000c 40018800
00009800 00f00000 00020040 020c000c 00000000 8200c004
00600000 40200000 c0000000 18068a78 21800001 8050010c
00040000 80000000 08000040 10040000 00400005 00460040
c00c0000 40000088

>D00169
00008002 00000800 00000100 00010200 00000000 00014020
00000010 08000040 00802100 00000851 00210004 40009800
06008004 00000400 00020040 00080008 00040000 02000000
0800800a 00000000 41000000 08020b20 20000000 00400004
00042000 80000600 02040000 00001080 00408008 00260000
c0000000 00020800

>D00168
00000002 01400000 80000000 88090600 003800e0 00004140
00009400 00000800 04000102 818000b0 0400000c 40019004
00009000 03c80110 00281080 001ec00c 00200000 00000000
09c00000 00201001 80080020 18000010 27800041 0c600810
10000400 40000002 00000200 10000070 00000208 01460000
40280000 000000c0

>D00160
00010000 01000000 00000000 00000400 00080000 00000000
00000000 00000000 00000000 00000080 00000000 40011000
04008008 00800000 00000000 00080008 00000000 00000000
00402000 00000001 00000000 10000a10 01000000 00000010
00000000 00000000 00000000 00000000 00000000 00040000
00000000 00000000

>D00158
0000000a 00004800 00001100 50010600 000000e0 00080000
00000000 01101100 00000100 000808c0 08088024 40819800

03008000 00100000 40060400 000c008c 18008000 02000005
00200000 00000000 00000000 18068a00 21802000 0044041c
00000000 e0000080 08000010 40001000 00080084 00060043
80007200 20000c00

>D00155
19004446 31401080 00502800 81810438 041a03e2 00008401
00502880 04800842 00600340 018d02b6 34a08008 44019a20
00001600 03981108 150a1080 105c800c 00209828 8b400020
1bd05010 e4201041 86082820 11a12050 27000015 18700414
112d1410 60008202 40810000 10021060 0400061d 00c60800
c0080000 20008308

>D00153
00010042 01000020 00000100 00490300 c0080000 04000000
00000080 02000402 01000000 00100000 00100000 40009000
04028028 00800000 00000410 00081008 00820000 00000020
02412000 00020001 00000021 00000a80 01008000 00000810
00200400 00000000 00404000 80000000 00000200 00000002
24020000 00000000

>D00148
00000002 01000828 00000102 00090200 20000000 0080800a
20000001 00000403 81000800 00980840 08100000 40009000
04028000 00200000 00000420 00080000 00000604 02100020
00312000 02020801 00400101 080202c0 0010c000 00400814
01200000 84002001 00404000 80000000 00000000 80220002
a4024000 00000410

>D00145
08000003 42000008 20000188 08090610 02001008 00084000
4204a000 01000800 00400100 00080840 28000004 4000b000
00004002 20000000 01020001 000c800c 00010000 02000005
00480000 40220a00 00000000 0c000400 20800840 00420010
00040000 80000000 00120000 40000000 00000288 0046000a
40000000 200004c0

>D00142
1003c00a 01a05800 00180902 98090e30 0a1a10c8 0010c000
0000e000 22001801 10400100 000208c0 41210004 4000b000
06008822 02108000 008e0440 100c010c 18040000 02000000
08400000 40200201 40100000 1c068e40 23810840 0040001c
00140000 c0000001 08008000 9000008c 0000810c 00461801
e0044000 80800ac0

```
>D00141
0000800a  00000a08  20100188  50010718  00045060  00000000
060c2000  00001000  80500000  000808c0  08000004  4001b800
03008866  00608000  c10e0041  020c000c  18810000  83800004
01280400  00020200  40000000  18028f18  21804000  81500014
00042000  c0000100  08160000  10000000  04000204  0006084b
80042200  00020420

>D00139
08000000  00200008  24080008  00000200  00011a14  00400020
02400000  40200200  02024000  40000840  00000000  00408000
4000c402  00200080  01000081  00000009  00000000  00000000
08000000  00020002  00000000  08000408  00000008  00180000
08400100  00000000  00000050  08100000  0a002200  01020000
00000000  00000000

>D00136
00028000  01000a00  00100102  02010600  00000000  00004010
00000000  20000000  00100900  000808c0  08300000  50019800
060080a6  00201000  00200000  000c0008  00000000  02000080
00800000  00100201  00000100  18020320  01000080  00500014
00002001  80000000  00040000  10000001  00000000  0006800a
c0000000  00822600

>D00132
00000000  00000800  00000100  00010200  00000041  00000000
00000000  01000000  00000000  00080a40  08000000  40009200
01008000  00000000  40040000  08080008  08000000  02000000
00000000  00000000  00000000  08020a00  01000000  00400014
00000000  a0000000  00000000  40804004  00000080  00020002
80002000  00000400

>D00131
00000000  00000010  00004000  40100400  00000400  00000108
00000000  20000000  00200000  04000000  00200000  40000000
00801082  00080000  00000000  00000000  00000000  00000200
00000000  00040200  00020080  00000000  00000000  00004000
00000800  00000000  00000000  04000000  00200000  00040000
00080000  00010208

>D00130
00002000  00000808  20100100  02010200  00800001  00024010
02000000  00000800  00000100  00010840  00000000  c0008000
```

00008012 00001000 00000000 08082008 00020200 02001000
04000000 40120000 00000000 08020a20 10000000 00400004
00000000 80000000 00020000 00000041 10014000 00028000
c0000000 00000200

>D00127
00424292 80808000 05484120 11f10601 000c8001 00104208
80030090 00046c00 81800580 20508a40 00386204 6020a008
a010a800 0011a103 8030c002 001c900c 20042c80 12004101
08678020 90a36400 c0140000 3e008ea1 20886800 2860002a
02002000 8c20800d 40442090 400501cc 0842d418 20161803
d400500a 403103e8

>D00126
00000000 00000000 00000100 00010200 00000040 00000000
00000000 00000000 00000900 00080840 08100000 40009800
01008000 00000000 40040000 00080008 08000000 02000000
00000000 00000001 00000100 08000a00 01000000 00400010
00000000 80000000 00000000 00000000 00000000 00020002
00002000 00000400

>D00125
00050041 6b000008 a000010a 00810300 88281050 4000440e
43943c00 0d000041 00202b00 01080868 4c100000 40009800
0400c002 22a10000 01080025 022a9108 00820200 02000104
1358a008 00020a03 00140720 18002a20 05004040 08420410
40000c00 e0002201 005a0000 64000020 03000283 0002000a
40a00000 02410400

>D00501
0001002a 01801800 00080000 02010700 00080040 0000c040
00008000 00000000 06400100 800000a0 0c00800c 40011000
04009010 00c00000 00280080 000c000c 00800000 00088000
00402000 00000001 80000100 10000250 21800041 00300014
00040000 c0200000 60000000 00040000 00004000 00460000
c0080000 00000280

>D00496
00000000 00000000 00000000 00100400 00000000 00000000
00000000 00000000 00000000 00000000 00000000 40003000
04008000 00040000 00000000 00080008 00000000 00000000
00400000 00400001 00000000 10000a00 03000400 00000010
00000000 00000000 00000000 00000000 00000000 00040000
00000000 03010000

>D00494
00204002 00820200 00120020 00100600 20040000 00002000
00010010 00000000 00022000 00400e40 00244004 61008000
00000000 00002000 20020040 02000002 04841000 20000000
08200002 02020000 c2100000 38000000 20800000 0018001b
00004401 10000300 02204010 00000080 08008200 01060000
0000a000 00010220

>D00488
00008002 00000801 00000180 480d0610 0700000c 00004008
0000a000 00080800 00400100 000c0840 00008004 40009800
02000004 00300000 20020000 800c0004 00020800 02002000
04400000 40200000 00000000 0c000100 20804840 00400110
00842001 80080200 00040200 00000040 0001400c 00460003
40000800 210200e0

>D00487
00000002 00000008 60004008 00000600 00041010 00000004
04080000 00200000 0c000200 000000c0 00200004 00050000
00008804 08000000 01030000 0014000c 00020000 00002000
00200000 00060200 40000000 18008c10 30800000 0000a000
00002400 00110002 00004400 00000000 00000200 00060001
00000008 30000000

>D00475
00000010 00801800 01100100 01b10600 00080008 00004000
00000000 00000000 00000100 00400840 00202000 60008000
00008002 00000001 80200000 00080008 00002000 02000001
00400000 00006600 80000000 18120a20 00000000 1840001c
02000000 80000004 00000000 0000002c 00000400 20060000
c0000000 00012260

>D00463
0040000a 00001011 80080100 10210250 00000001 10004008
00000400 008c0044 00004100 48020850 00008881 50019802
04008480 00200008 20020020 800d000c 00028000 07002800
04400218 40008009 04201000 08000a28 21004080 04500230
00040001 a0084201 04000208 18041040 80004004 02a30010
400408a0 20000000

>D00459
00018008 01805200 02100102 94010700 200800a0 00000000
01001000 22001004 00400880 000908d0 08302000 5001b000

060088c2 00202003 80181488 000c1008 10820200 02000020
00400008 00000201 80020120 38208e00 33004041 83400018
00000c00 c0000001 08004000 90000001 0c000300 0086000b
20044000 00422600

>D00455
00000002 44008028 2000652a 10502211 00041060 00004000
16080104 00000008 04020100 0001485d 01008004 42008000
00001806 40640000 81020041 022e200c 24000008 0200030c
80280820 00064000 42100002 1800aa20 21800000 01480000
00242000 90018000 48000048 00000000 00400304 00c60020
40080000 002580e0

>D00454
08210246 43842800 02100904 0c200600 00120003 80100004
10c80190 10301054 10016600 80030843 12202204 440584b0
0000081a c010a809 040634c0 18180088 08658809 1308401a
09000002 30809281 40022000 78029080 e7104120 02401000
03088000 4c000180 4a800410 9d011c84 08988918 01ae0000
20000081 50e10201

>D00452
00010040 00000800 00000100 40010200 00000040 00000000
00000080 01002001 00000100 00480840 08000200 c0009880
01008010 00000000 40042000 00080008 09010800 0200000a
00100000 00000004 00000800 480a0a80 01000000 10400014
02000000 a4000000 00000000 40000004 00000180 00020002
80002000 00c10400

>D00451
00000002 04000820 00102100 41310610 00041000 00000000
00002004 00008000 00400100 00090844 0820240c 40008800
10001820 00440001 80000040 0004000c 00000018 02000104
00200400 00004000 40000000 18a08008 20800000 91400030
00042000 80000000 08000040 0000000c 04400000 00060002
000c0200 00010660

>D00449
00004006 04800200 007a0000 00900600 20000000 02002100
00000221 00000000 84004100 000049e8 0078000c 70049000
0c018400 00340000 a6220040 00000040 04040000 00000000
0c004034 00202001 e0100000 1c000601 60806900 08240019
00002000 00000000 04000600 10000080 01008000 000e0013
10000000 01010328

>D00448
00000012 00882808 61004102 00b10200 00050012 00024000
02000000 00000800 00000300 00040840 00002104 60008000
00048c02 08000001 80280050 102e200c 0000a000 02800000
00600000 80226001 c0000000 0e229a20 20840846 0860200c
00050480 c1000000 80040080 0400004c 00500408 20060000
c0000080 00010060

>D00437
00410002 81000019 20200100 80030700 00080040 00900000
02002821 00000400 15400c00 009a0860 4e300004 40019000
0c03e0a2 02002000 20120140 10098159 00830008 03100000
00430018 06006801 40020101 08100a00 31048040 00c00890
84803800 c0000000 00500000 90040101 00100200 80060203
04200000 64000610

>D00434
00010040 81040020 00000900 80010300 c0080040 00000001
00000000 00000402 00004000 01100008 00000200 40009000
14028028 00800004 00000404 0008100c 00820000 00000020
03402080 0000a001 00000020 00001a00 0100a000 00000012
00200400 00000000 00406200 80000000 00000200 0000000a
24020000 00000000

>D00425
00000000 01000008 2000010a 00010200 00001000 00000000
02000000 00000000 00000800 00080840 08100000 40009000
04008002 00200000 01000001 00000008 00000000 02000000
00000008 00020001 00000100 08000208 00000000 00400010
00000800 80000000 00000000 00000000 00000200 00020002
00000000 00000400

>D00423
00000004 01400008 c0100000 80010400 013800c0 00008004
00c00000 00000401 00000110 818000a0 08190028 44018810
00008000 03a00100 002a1080 001aa00c 00008000 00000002
19d00000 00201201 80180020 10000650 27014000 0e000410
10200410 40000402 00000800 31020060 01000305 00040001
80000080 00008108

>D00421
00010000 01005200 00100102 84010700 00080080 00000010
00000000 20000000 00000800 000808c0 08302000 4001b000

0600801a 01a02003 80181008 000c1008 10820000 02000000
00402000 00000201 00000120 38040e10 13000001 02500018
00000400 80000000 00000000 10000001 08000200 0106000b
20024000 00402600

>D00418
01010002 01400001 00100000 48080600 22000008 00006100
0000a010 40100800 00000100 00000080 00200004 50011000
04000002 000000c0 00020080 000c0014 00041000 00014000
00400000 00200201 00902000 14060000 20804840 00100010
00080000 00100000 00000400 108000c0 00000008 00460000
40001000 000002c0

>D00417
00002002 00000801 00004100 10000210 00041022 04010040
10000800 00000000 06000100 40810070 04000004 60800000
80018800 00200000 00120000 8204000c 00420000 02001004
04a00000 00200002 40000000 88028000 30804401 02500418
00040001 c0080000 08000640 00000041 00400004 00020000
00000000 00000220

>D00416
00008002 00000200 80000100 80010600 00080050 00004020
00000000 00004000 00100100 000808c0 08009008 40018900
020010a4 00890400 00001080 020c800c 00201000 02000200
10400000 00000000 80001408 18000120 23000000 85400010
00002400 80002200 000c0200 00000030 04000200 0046000a
40080000 00020400

>D00414
00010000 11800010 c2004100 00010e00 10080000 00000004
00000080 0220b000 00000000 004808c0 09200200 c0018080
00008810 00000020 00002080 00000010 00005001 02000008
00200000 0000000c 80001800 48098488 00000080 00400018
02000402 84010000 00000000 80000004 00000340 00060007
00040000 80c10600

>D00410
00008002 00804800 00004100 00800210 00041000 00000000
00002000 00000000 00400800 00080040 00100004 40001000
03008800 00000800 40000000 00040004 00000000 02000004
00200400 00000001 c0000100 08148200 20800000 0840001c
00040000 00000000 08000000 00000000 00400000 00020002
80002000 00000500

>D00401
00040002 00000000 00000000 00000400 00000040 00000000
02000000 00000000 00000100 00080000 00008008 00000000
00001000 00000000 00000000 000c0004 00000000 02000000
00400020 00000000 80000000 10000000 21000000 00000000
00000000 00000000 00000000 00000000 00000000 00470000
40000000 00010008

>D00398
0000000a 00c01800 00080408 80010700 00080040 0000c100
0000bc04 00000840 06402100 810000f0 0c00800c 4000b010
04009000 00480000 00081080 101d900c 00220020 00008000
10400000 00201001 80080120 1c000a40 27800840 08300814
00000400 c0200002 60000200 10040060 00004208 00461000
c0200000 80020280

>D00394
08004002 00000000 00180100 00010600 20000020 00002040
01000010 00000040 00102800 08480960 08340004 40808000
00000400 00000000 00020040 08000002 0004c200 02000001
0800000a 00000001 65100100 18100000 20000000 08500010
00000800 80000000 02000008 00001080 00088000 00a60012
00000080 20000700

>D00391
08000002 01400000 00100000 92010610 00180062 00004140
00000c00 00000000 06000100 818000b0 0c00000c 40018000
00009000 0bc80110 00a81080 001c800c 00200120 82008000
09c00000 40001001 84080020 10000010 25800041 08300410
10040404 c0000002 20000200 90040060 00004200 01460000
40280004 00000280

>D00387
0080800a 06840019 402c0500 98010780 41004040 00880026
14840000 01201401 10000300 00290845 09010134 44068c00
02100884 08088a08 80068940 080c258c 0a208800 128a0207
00600408 00260000 40122400 2801a100 61001002 08508110
10802010 e00007c0 40084040 45021081 0000009c 00860c02
c40c0080 a4360400

>D00383
00010000 01005200 00100102 84010700 00080080 00000010
00000000 20000000 00000800 000808c0 08302000 4001b000

0600801a 01a02003 80181008 000c1008 10820000 02000000
02402000 00000201 00000120 38040e10 13000001 02500018
00000400 80000000 00000000 10000001 08000200 0006000b
20024000 00402600

>D00380
00000010 00801000 01100100 02b10e00 00000008 00000000
08000000 00003000 00000000 000028c0 00202000 e0008400
00009000 00000041 80200000 00000000 02002000 02000000
00400000 00006001 80002000 18050400 00000000 08400018
00000000 80000000 80000000 1000042c 00000400 20060001
00080000 00010260

>D00377
00000002 00000808 20000100 00010200 00000000 00024000
02000000 00000800 00000100 00000840 00000004 40008000
00008002 00000000 00000040 10082008 00000000 02000000
00000000 80020000 40000000 08020a20 20000000 00400004
00040000 80000000 00040000 00000000 00400000 00060000
c0000000 00000000

>D00371
0000002a 00801800 00080000 02010600 00080040 0000c000
00008000 00000000 06400100 800000a0 0c00800c 00000000
00009000 00400000 00280080 000c000c 00800000 00008000
00400000 00000000 80000100 10000040 21800041 00300004
00040000 c0200000 60000000 00040000 00004000 00460000
c0000000 00000280

>D00369
0004c002 00000800 00002100 40010600 00000000 00000000
00000000 00000000 00000800 00080840 08300004 4000b000
04009002 00008000 00000040 00080008 20040000 02000100
08400008 00000001 40100100 18000b00 23000040 81400010
00000800 88000000 00000000 00000080 04008000 00060002
00000000 00020600

>D00364
0008800a 0d405000 00104300 10810610 2a061020 0008a002
84082480 09001409 04400a00 001a08c8 08308004 50818a00
02008826 00200800 82020022 021e208e 01020400 0a080045
40340c00 00000201 40100100 18148d50 29c0c000 08550818
01042000 a2000083 08442040 50a08200 02400286 00060c03
84020000 20030700

>D00362
00010000 01005200 00100102 04010600 00080080 00000010
00000000 20000000 00000800 000808c0 08302000 4001b000
0600801a 01202003 80180000 000c0008 10000000 02000000
00402000 00000201 00000100 38040e30 03000001 02500018
00000000 80000000 00000000 10000000 08000000 0006000b
20004000 00402600

>D00359
00010040 81040020 00000900 80010300 c0080040 00000001
00000000 00000402 00004000 01100008 00000200 40009000
14028028 00800004 00000404 0008100c 00820000 00000020
03402080 0000a001 00000020 00001a00 0100a000 00000012
00200400 00000000 00406200 80000000 00000200 0000000a
24020000 00000000

>D00342
00000002 01400000 00200400 82030600 00180060 00004140
00000402 04000000 06000100 818000b0 0c00000c 40018000
00009280 03c80110 00a81080 001c800c 00200000 00008000
09c00100 00001001 800a00a0 10000010 25800041 08300010
10050400 c0000002 20000200 10040060 00004200 01460000
40280010 00000080

>D00340
0000c012 00802200 07000000 02b00a20 00000010 14000000
08000000 00002009 40000010 00007840 00202004 a200c800
42008006 00000045 90220044 00008000 01a42100 00000000
0a400200 00066002 c0002000 08010100 20000000 08500008
00106010 00080040 100000a0 000004cc 00008400 60060080
0000c000 00234260

>D00333
0000000a 00c01800 00080000 80000600 00080040 0000c100
0000bc04 00000800 06400100 810000f0 0c00800c 40009000
00009000 00480000 00081080 101c000c 00200020 00008000
11400000 00201000 80080100 1c000040 25800840 08300814
00000000 c0200002 60000200 10040040 00004208 00461000
c0200000 80000280

>D00332
00030000 01000000 00000000 00000400 00180000 00000000
00000000 00000000 00000000 00000080 00000000 40011000

```
04008008  00800000  00000000  00080008  00000000  00000000
02402000  00000001  00000000  10000a10  01000000  00000010
00000000  00000000  00000000  00000000  00000000  00040000
00020000  00000000

>D00330
00002000  00000000  00100100  02010200  00000040  00000010
00000000  00000000  00000100  00082840  08000000  50009800
01008000  00001000  40040000  00080008  08020200  02000000
04000000  10000000  00000000  08000a00  11000000  00400010
00000004  80000000  80000000  00000041  00404000  00028002
00002000  00000600

>D00328
00010000  81000020  00100000  00010100  003c1440  00200004
00000880  08000402  01204000  01108020  00000000  4000d000
14028028  02800004  00000400  08281008  00800000  80000020
1341a000  00002001  00000020  00000280  05008000  08100014
00200400  00000000  00404020  b4000020  00000200  03000000
24a20000  00000200

>D00325
00010002  81400020  00100100  00491300  403c1440  04200004
00000880  0a000402  01200000  01100020  00100001  4000d000
14028028  02800000  00000410  08281008  00820000  80000020
1341a000  00022001  00000021  10000a80  05008000  08520814
00200400  00000022  00404020  b4000020  00000200  1300000a
24a20000  02000200

>D00324
00010002  81000020  00100000  00490100  403c1440  04200004
00000880  0a000402  01200000  01100020  00100001  4000d000
14028028  02800000  00000410  08281008  00800000  80000020
1341a000  00022001  00000021  00000280  05008000  08100814
00200400  00000020  00404020  b4000020  00000200  03000000
24a20000  00000200

>D00322
00000004  00800208  c0100100  02010600  00180400  0000c014
00000000  00000000  00000100  000909c0  08000008  c0008000
00000000  01001000  00200080  0008000c  05108200  02000280
18400000  00300000  80000000  18000060  23000040  8d400010
00000000  80000060  00000100  01000021  04000000  0006800a
c0000000  00000740
```

```
>D00317
0000000a  00801800  00080100  00010700  00180040  0000c000
0000a000  00000800  00400100  010000c0  0000800c  40019000
00009000  00800000  00080080  100c100c  00020000  00000000
09400000  00200001  80000020  1c000a50  21800840  00400014
00000400  00000000  00000200  00000000  00000208  0046100a
c0080000  00000080

>D00315
0001004a  03004808  02000500  50210310  800c5071  02204002
11003000  00000041  00400b00  01082850  08108004  50819800
14008880  00618000  000e0040  020c000c  18008000  06800004
0568041a  40400001  44010120  08008a68  a18050c2  80400110
0004ac00  80002201  08080040  00000000  00000205  00820c02
40040200  20000420

>D00805
04080001  00000a80  00000400  00000000  00000004  08000000
00004000  00000000  00000000  00000000  00000000  40000000
00000000  01020000  00000000  00080008  00000028  00000000
20000002  00000000  00000000  05500100  00000000  00000000
00000040  08000000  02000000  00014000  00000000  00000000
00000000  00028005

>D00786
08000002  c2000818  20001101  40010200  00000000  00000000
02002000  01000000  14400100  00080860  0c000004  40008800
00018202  00000000  20100140  3000c018  00000000  02000000
00000000  00020600  40020000  08020200  20000000  00421414
00040000  e0000000  00120000  4000a000  80400080  00060002
80000000  24000400

>D00785
00000000  00000000  00100100  00010600  00402000  00000000
00000000  00100000  00000880  00080840  08300000  40008000
00000002  00000000  00100000  00000000  00000000  02000000
00000000  00000201  00000100  18200000  00010000  81400010
00000000  80000000  00000000  00000000  04000000  00060002
00000020  00020600

>D00781
00000006  c0008018  20100100  00010620  00000004  00080000
82102080  00000400  15800000  00400860  04200004  40129000
```

00018202 00010000 20100142 0000c018 20400400 02000100
00018000 00820602 40020000 18000600 60000401 00401418
00040000 c0000000 004a2100 4001a000 00480000 000e0101
04000000 0c008280

>D00771
00018002 00000010 00080000 00000200 00240200 00000000
00000404 00000200 00084420 00000850 00000005 0000a800
42000414 01600000 20000140 0604000c 00000000 40000020
00200080 40000000 40020000 08000100 20810000 04980000
00006000 00000000 00000010 18000000 00002000 01060000
00140000 04420000

>D00762
00000000 00000008 20000100 00010600 00000000 00000000
02000000 00000000 00000800 00080840 08100000 4000b000
04008002 00000000 00000000 00080008 00000000 02000000
00400008 00020001 00000100 18000a00 03000040 81400010
00000800 80000000 00120000 00000000 04000000 00060002
00000000 00000400

>D00753
00010440 81045000 02900900 80010700 c00c00c0 00200001
00000840 28000402 04000040 011080a8 40202200 5001f000
1602800a 028a0002 8008140c 0828100c 10a20000 80000000
03c02090 0200a201 00000020 10040e10 1740a080 0810001e
10201401 00000400 00c0c000 b0000001 00000240 80040003
24a24000 00002210

>D00752
04010001 c9000808 20000309 80050702 00081091 0882c000
42140001 00000e02 00040100 00980a48 28200000 40009800
0402c022 00210000 0128100d 0a083108 10860800 02100200
00680000 42022a01 00000020 28020b20 13008000 00400014
10200400 c0002000 004a0000 80000001 00000301 80060002
c4000004 00000600

>D00733
04000002 44003818 20004108 04010700 80041000 02008100
06000000 0200100c 00010200 00010840 00240004 40008000
80808802 00402080 31004541 201c200c 10000008 02000000
00680000 00020001 40020040 28028608 21800000 8244801c
00004000 c0000000 00000000 80000000 09000300 00060001
80048000 04000220

>D00726
00008002 88002808 20000108 84010600 00001000 00020100
06000000 02001408 00000000 00110848 08200004 40008800
02008806 08002000 81000441 00002008 00000000 02000400
00000000 00020400 40000000 28028700 20000000 0240000c
00002000 c0000400 00100800 80000000 08000300 00060801
84040000 00020200

>D00709
00020000 00003000 00100000 00000400 00000000 00000010
00000000 20000000 00000000 00000080 00200000 40003000
06008002 00000000 80000000 00000000 00000000 00000000
00000000 00000201 00000000 30000600 00000001 02000018
00000000 00000000 00000000 10000000 08000000 00040001
20004000 00802200

>D00703
00000000 01400000 00000100 00010300 00482000 00000000
00000001 80000000 00000000 00000000 00000080 40008000
00008000 00800100 00000000 00190008 00820000 00000000
02402000 00000001 00000008 00000c00 01000000 00000010
00000000 00000102 00000000 00000008 00000200 00040202
00020800 00000000

>D00691
0000002a 00801800 80080000 02010700 00080060 0000c140
00008000 00000000 06400100 808000a0 0c00800c 40010000
00009000 00c00000 00280080 000c800c 00800000 00088000
00c00000 00000000 80000100 10000040 27800041 04300014
00040000 c0200000 60000000 00040030 00004000 00460000
c0080000 00000280

>D00670
00010000 43000808 2010010b 00010600 00001000 40008000
02040000 22001000 00000800 000808c0 08300000 50019000
0400c80a 20201000 01004421 20028008 00000000 02000000
00082008 00020201 00400100 98028250 00004000 00500014
00000800 c0000001 00120000 90000000 00000302 0106000a
a0060000 00020600

>D00667
00000002 81001008 60100008 00000600 00001000 08008000
02000000 00000000 04000200 000008c0 00200004 40018000

00008402 08000000 01010041 00120008 00060000 00080000
08002000 00022601 40000000 18040c50 30000000 00102018
00000000 00010022 00000000 10000080 00008200 00060001
80020008 10020200

>D00658
0004c012 00802202 07002000 03b00e20 00080012 14000000
08000000 00002009 40080030 00007840 00202004 e200c800
42009006 00000045 90220044 00008000 01a42100 00040100
0a400200 00066002 c0102000 08010100 20000000 08500008
02106058 08080044 100000a0 000004cc 00008400 62060080
0000e000 00234260

>D00656
0004c016 00802202 07002000 03b00e20 00080012 14000000
08000000 00002009 40080030 00007840 00202004 e200c800
42009006 00000045 90220044 00008000 01a42100 00040100
0a400200 00066002 c0102100 08010100 20020000 08500008
02106018 08080044 100000a0 000004cc 00008400 62060080
0000e000 00234260

>D00655
00070004 40000008 22001265 00780600 00001000 00100001
00021000 00000014 00000000 00008002 00002000 60000080
8440944c 00040005 80006800 00082a0c 00020c00 00000201
00820020 00a04000 00000000 a0201690 22100240 00804010
00000000 2800c000 40001002 0021004c 10001000 00050008
000c0000 01010068

>D00654
00004012 00803000 07100100 02b10a20 00000018 04000000
08000400 00012009 40000010 00003850 0020200c e200c004
40008000 09000045 90320044 00008000 01a42180 02000000
0a400200 00066012 c0002000 08210000 20000000 08500008
00146010 80000040 000000a0 900004ec 00408400 60060080
0010c000 00214260

>D00653
10010010 05401200 01106b06 81b80640 00140103 00210020
18420000 24001000 05880840 000ac0c0 00702201 e41ac0e0
1b21021e 04261301 82001400 10004100 0c000081 02022000
00010030 0000e201 60000000 58849083 00800360 1a040219
0c02f000 74002000 00000800 9401000d 00600100 20070032
10008000 01c34a61

>D00652
000100d0 20440000 01106b04 87b90600 001c1102 00610008
34030001 04041008 018810c0 00c8a800 00602201 e01cc0c0
1a00021f 08022201 81101400 20288108 08202081 02402000
00e10060 0000f605 00000000 78a09080 01180181 3b000238
4200e001 64002004 00000800 8001000c 0c000700 24064042
00010002 00c16b61

>D00651
0200c016 40822208 21002508 01b10620 00088013 14010200
18001010 00002c01 40800010 8008c843 80006004 62008840
4242100e 40040009 802200c0 20018008 25002d08 02040100
03500400 01047000 c0002000 39010100 20000204 08524408
060c6000 88080044 518804a0 08000c7c 00080400 62860000
04004102 06030160

>D00650
00004052 20803002 47100100 03b10e20 20080018 04000000
08010400 00012009 40000010 00483850 0820200c e200c004
40008001 09000045 90320044 00008000 01a42180 02000000
0a400200 00066412 c0102000 18210000 20000000 99500019
02146018 80000144 000000a0 900004ec 04408400 62060082
0010e000 00214660

>D00630
00000040 01010040 00000000 00010000 00280060 00000000
00000008 00000440 1000a000 03000000 00000002 42808000
80018800 02800000 00080000 140a8018 00000000 00000000
21402000 00000001 00000020 10000000 05000050 1d000010
00000400 00000000 00000000 00000220 24004200 00000000
00000000 10000000

>D00625
00000000 01000000 00100000 00000400 00380080 00000000
00000000 20000000 00000000 00000080 00200000 50010000
00000002 02800000 00280000 00080008 10000000 00000000
01c02000 00000201 00000000 10000010 07000080 00100010
00000001 00000000 00000000 10000000 00000000 00040000
00000000 00000a00

>D00624
00010000 01005000 00100000 84010700 00080080 00000010
00000000 20000000 00000000 00000080 00202000 4001b000

0600801a 01802003 80181008 00081008 10820000 00000000
02402000 00000201 00000020 30040e10 13000001 02100018
00000400 00000000 00000000 10000001 08000200 00040001
20024000 00002200

>D00623
00018008 47805208 2210010a 94010700 200810a0 c0000000
03041000 22001004 00400880 000908d0 08302000 5001b000
0600c8c2 20202003 81181489 000c9008 10820200 02000020
00480008 00020201 80020120 38208e00 33004041 83448018
00000c00 c0000001 08324000 90000001 0c000300 0086000b
20044000 00422600

>D00622
00010440 03005208 0010050a 00050e20 00080081 00000010
00004900 30000000 0000c800 000808e0 09300001 4011b000
0600801a 01a200b0 82095000 20281608 100a0028 02100003
c2406010 00000201 01004120 19562e10 03c00000 80508218
00002402 80000100 22000400 38214000 008c0200 01060303
30864000 0000ce21

>D00621
00010000 01005200 00100102 84010700 00080080 00000010
00000000 20000400 01000800 000808c0 08302000 4001b000
06008012 01202003 80181008 000c1008 10820000 02000000
00610010 00000201 00000120 38040e00 13000001 02500018
00001400 80001000 00400000 10000001 08000200 0006000b
24004000 00402600

>D00620
00014002 01004200 40101122 84010708 20080180 00002040
03000000 20100100 00100800 08080ac0 08382024 4001b000
06008002 00302803 801a1048 080c100a 10a68000 02000000
08400008 80000201 44100120 38000e10 33002001 02d0001a
00000400 80000080 48000010 10001081 08088200 0006000b
20005080 20422600

>D00596
04006002 00001328 20107308 00110606 00051080 00000800
02040000 00000400 01400800 230948c1 08340005 40009300
0481b802 000c0000 01001001 000c000c 04040410 02004200
08210018 00a20101 40120108 1c048e04 32800900 02600018
00001800 80001020 08520080 140000c0 00008208 0006081b
04005010 83010600

```
>D00593
00000010  01809000  01100110  02b10e00  00000048  08020010
08000024  20003404  01000000  011828c0  08202000  e0019400
04009302  00010041  80200000  00000000  22002008  02000500
02412040  00006201  80022100  18250710  00008000  89500018
00010000  88000000  80400000  1000052c  04000400  20060003
0c0a0004  00012660

>D00584
00000002  00000000  00100000  06000600  00000000  00004000
00000000  00000000  06000100  00000020  04000004  00000000
00008000  00c01000  00220000  000c0004  00000000  1000c000
00400000  00000000  00000000  00000000  20800040  00100000
00040000  c0000000  20000000  00040800  00004000  00420000
40000000  00000280

>D00579
00000000  00000800  00000000  00000000  00000000  00000000
00000000  00000000  00000000  00000000  00000000  00000000
00008000  00020000  00000000  00000008  00000028  00080004
00000000  00000000  00000000  05400800  00000000  00000000
00000000  00000000  00000000  00014000  00000000  00000000
04000000  00008804

>D00577
00000000  00000008  20000300  00010200  00000000  00000000
82000080  00000402  00000800  00180840  08100100  40008000
00000002  00000000  00000002  00000008  00000400  02000000
00000000  00020001  00000100  18000000  00008001  00400010
01000000  80000000  00522000  40000000  00400000  00020002
04000000  00000400

>D00574
00010002  01001800  00100102  40010600  00000000  00000000
00000000  20000004  00100880  000a08c0  08300004  50019000
0500801a  00200000  40000040  00000000  00000000  02000000
00002000  00000201  40020100  18240610  20000000  81500018
00000000  80000000  00000000  10000000  04000000  00060003
00002000  00402600

>D00569
04004002  00000008  20000008  00000600  00001000  00000000
02000000  00000008  00000000  00000840  00280004  40008000
```

00808022 00100000 01000041 00080008 00040000 00000000
08000000 00020000 40100000 08000600 20002000 00000000
00000000 00000000 00040000 00000080 00008200 00060001
00000000 00000200

>D00568
00000040 00801200 00020100 00910200 00100040 00000000
80000080 00000440 01800000 00080840 48002100 60009000
05008000 00010001 c02c0002 00080008 0c020410 02000000
4441c018 00002000 80010020 19040a00 01800000 08400019
00001400 80000000 00402000 40000420 00400200 00020002
04002000 00810430

>D00567
00006036 00802208 a1100100 00b10600 01080010 00004002
00440400 00001400 00000104 000108f0 0900a004 6000800a
00000810 02000201 80200040 000c200c 00020000 02801000
04400000 10222008 c0100000 1801a020 30800800 8b404008
00000000 c0000040 30c20000 90000869 04004104 20460008
40260200 800126a2

>D00566
00000000 00000808 20000100 00010200 00000000 00024000
02000000 00000800 00000100 00000840 00000000 40008000
00008002 00000000 00000000 00082008 00000000 02000000
00000000 00020000 00000000 08020a20 00000000 00400004
00000000 80000000 00000000 00000000 00000000 00020000
c0000000 00000800

>D00563
0800400a 00004002 40104120 14010618 00041020 04082040
11002401 23001041 00500180 38090a40 08200004 4080c000
01000ca2 00202000 00020400 021c0086 04259200 02004005
08300400 00a00200 45120008 3e208400 21814821 83740012
00040080 e0000080 08004088 c0001080 0e488198 00860803
200610a0 20010600

>D00562
00000002 00000800 00000100 40040600 04000400 00014000
02000000 00000c00 06800900 00080020 00108004 60000800
08008000 00500000 00020000 000c0004 00000000 07008000
00400028 00010001 00000400 00000000 20800044 00100110
00002000 80000200 20000020 00040000 00004004 02430002
40000000 200100a8

>D00560
00000002 01000400 00100102 02010600 000c0040 00004090
00000000 21000000 04300900 800809e0 0d30800c 50018000
00009002 01e41000 003000c0 000c0004 00100000 02000080
00600000 00100201 c0000100 18000030 21800001 00700010
00000000 e0000000 00000000 50000001 00000080 00468012
40080000 00002680

>D00550
0080804a 06840011 403c0500 9a010790 40000060 00080032
1404d000 01201001 10000300 00091855 4903801c 40868c00
021018a4 08189a08 80868940 080c0184 0b200a08 020a0203
80e00408 40020000 c0122001 38018100 610010c2 99508910
10842000 e00005c0 4008c000 45001001 0400089c 00ce8c02
400c0084 a4320600

>D00549
00000000 00000008 20000100 00010200 00000001 00000000
02000000 00000200 00000000 00080840 08000000 40008000
00000002 00000000 00000000 00082008 00000000 02000000
00000000 00020000 00000000 08000000 00000000 00400010
00000000 80000000 00000000 00800000 00000000 00020002
00000000 00000400

>D00547
00000000 00000000 00100000 00000000 00000000 00000000
00000000 00000000 00000000 00000000 00000000 40008000
00000000 00000000 00000000 00080008 00000000 00000000
00100000 00000000 00000000 00000000 00000000 00500010
00000000 00000000 00000100 d0000000 02000200 00000000
00000000 00100600

>D00546
00000000 00000000 00100000 00000000 00000000 00000000
00000000 00000000 00000000 00000000 00000000 40008000
00000000 00000000 00000000 00080008 00000000 00000000
00900000 00000000 00000000 00000000 00000000 00c00000
00000000 00000000 00000000 d0000000 02000200 00000000
00000000 00100600

>D00545
00100000 00000000 00100000 00000000 00000000 00400000
00000000 00000000 00000000 00000000 00000000 40008000

00000000 00000000 00000000 00080008 00000000 00000000
00100000 00000000 00000000 00000100 00000200 00400000
00000000 28000000 00000000 d0000000 02000200 00000000
00000000 00120600

>D00544
00180000 00000000 00100000 00000000 02000000 00000000
00000000 00000000 00000000 00000000 00000000 40008000
00000000 00000000 00000000 00080008 00000000 00000000
00000000 00000000 00000000 00000100 00000000 00000000
00000040 08000000 00000000 d0000000 02000200 00000000
00000000 00020200

>D00543
00180000 00000000 00100000 00000000 02000000 00000000
00000000 00000000 00000000 00000000 00000000 40008000
00000000 00000000 00000000 00080008 00000000 00000000
00900000 00000000 00000000 00000100 00000000 00c00000
00000000 48000000 00000000 d0000200 02000200 00000000
00000000 00120600

>D00542
00100000 00008000 00100000 00000000 00000000 00000000
00000000 00000000 00000000 00000000 00000000 40000000
00080100 00000000 00000000 00000000 00000000 00000000
00000000 00000000 00001000 00000100 00000000 00000000
00000000 08000000 00000000 90000000 00000000 00000000
00000008 00020200

>D00538
00004080 04000020 001a2100 22310200 00040200 00100010
00000004 20008000 04020000 0002484c 00002400 40008000
00005020 00441041 80000000 0004000c 01000208 02020300
00200000 00000000 00802008 08010208 00c00000 00490080
00200000 80010000 00000030 08000109 0000000c 05028200
00000000 c0010030

>D00537
00000000 01020000 00000000 00e10100 80280140 00000004
80002800 08000440 00202000 010000a0 00002000 40018000
00004000 02800003 00000002 002a9008 00800000 0000a000
11c0a000 00000001 10100020 10000010 050000a0 08100010
05000405 00000001 40000000 24000420 00028201 00000000
00a00008 00010060

>D00536
00000000 00400000 00000100 00010200 000000c0 00000000
00000000 00000040 00000000 01080840 08000000 40008000
00008000 00000100 00040000 00180008 08020000 02000000
05000000 00000000 00000000 18000c00 01000000 00400010
00000000 80040002 00000000 00000000 00000200 0006000a
00000000 00000400

>D00535
00030000 01000000 00000000 00000400 00180000 00000000
00000000 00000400 00000000 00108088 00000000 40001000
04008000 00000000 00000400 00080008 00000000 00000100
00400000 00000001 00000000 10000a00 01000000 00000010
00000000 00000000 00000000 80000000 00000000 00040000
04000000 00000000

>D00533
08000002 00000800 00080100 00010a00 00000020 00012002
01000010 00082041 00902800 08480840 083c0024 40809000
01008400 00100000 40020040 08000002 0004c200 02000001
0800800a 00000041 45000500 08120600 20002000 08500014
00100800 80000200 02000018 00001080 00088000 00a60003
80002080 20004501

>D00530
0000e002 40000808 e0000180 18050e10 05005020 00004006
00401000 00280800 00000100 10010850 00290004 60018810
83808806 08100000 00020840 000c200c 00668000 02802000
0cc00000 00620000 41100000 0c028600 30802c41 82404108
00142400 80080000 18200200 000008f0 0040c004 00460401
40000a00 000042a0

>D00528
0000002a 00801800 00080000 02010700 00080040 0000c000
00008000 00000000 06400100 800000a0 0c00800c 00000000
00009000 00400000 00280080 000c000c 00800000 00088000
00400000 00000000 80000100 10000040 21800041 00300004
00040000 c0200000 60000000 00040000 00004000 00460000
c0000000 00000280

>D00521
00000000 01000000 00100102 00050600 00100000 00004000
00000000 21000000 00100900 000808c0 08300000 50018800

00000002 00200000 00000000 00080008 00000000 02000000
00400000 00000201 00000100 18000020 01000000 00500010
00000000 a0000000 00000000 50000000 00000080 00060002
40000000 00002e00

>D00519
0200c016 00822208 21000000 00b10220 00008012 14010200
08000000 00002c01 40000010 0008c842 80006004 22008840
0202000e 40000009 802200c0 00000008 25002d00 00000000
03500400 01046000 c0002000 39010100 20000004 08520008
00086000 08080040 518800a0 08000c4c 00000400 60860000
04004100 04030160

>D00518
0000c010 00800200 01000000 00b00220 00000000 14000010
00000000 00000000 00000000 00004840 00002000 20008800
02000006 00000201 80220000 00000000 00000000 00000000
00000000 00002000 80000000 08000102 00000000 08500008
00002000 00180000 10000080 00000008 00000000 60020000
00000000 00030020

>D00516
00000040 01010040 00000000 00010000 00280060 00000000
00000008 00000440 1000a000 03000000 00000002 42808000
80018800 02800000 00080000 140a8018 00000000 00000000
21402000 00000001 00000020 10000000 05000050 1d000010
00000400 00000000 00000000 00000220 24004200 00000000
00000000 10000000

>D00515
00000000 00020040 00000000 00000000 00000020 00000000
00000008 00000000 10008000 02000000 00000002 42800000
80010800 00000000 00000000 14080018 00000000 00000000
01000000 00000000 00000000 00000000 05000040 14000010
00000000 00000000 00000000 00000400 44000000 00000000
00000000 00000000

>D00513
04000002 00000008 20100108 00010600 00041080 02000000
06080000 00000008 00000000 00000840 00200004 40008000
00800806 00400000 01200041 000c200c 10200000 00000004
00a00000 00060200 40000000 18008008 27800080 88000010
00002001 00000000 00000000 00800000 00000200 00060000
00040200 00200240

>D00512
00000040 00000800 00000100 00010601 00080100 00010000
00010000 03005000 00000240 00180852 18200010 4000b001
09008801 00000008 40000000 80100008 00040802 06002002
00000000 00080000 00200100 38018600 80000010 81400014
00000080 a0000000 40001000 d0000000 0c200190 00060002
8c046000 20c00600

>D00510
00014006 01000000 00580000 00000600 20000000 00002000
00000221 00000000 00004100 000009e8 0038000c 70019000
0c008408 00300000 200200c0 00000040 00040000 00000000
0c002034 00200001 e0100000 1c000610 60006800 00300010
00000000 00000000 04000000 10000080 01008000 000e0013
00000000 00000308

>D00505
00000004 00000000 00000100 00410600 00000000 00000000
00008000 00000800 00000000 00080840 08002008 40008000
00000000 00000002 00000000 00000000 00000000 02000000
00000000 00000000 00000000 18000000 40000000 81400010
00000000 80000000 00008000 00000000 04000000 000e0002
00000000 00010420

>D00503
0020c002 00820600 00120000 05100600 20040080 08002000
08010010 00000000 00022000 00400d60 00244004 61008800
02000006 00002001 20120040 0208000a 14841000 20000000
08200002 02024200 e2100000 38000900 22800021 02180019
00006401 10000100 12204010 00000080 08008200 01060010
0000a000 00030220

>D01276
0000200e 09007400 00106142 14410600 000401a0 00018000
00010000 2208181c 03900b80 50080de8 0830a01c 4001b000
8c008802 00202002 201a0480 021e000c 10020000 02082000
04700010 10400201 c0000100 38288e10 73814041 83500018
00000000 c0000002 10000040 90000060 0d004304 004e0413
a0044800 000526a8

>D01275
00050040 6b000808 2000050a 40050700 88281051 4002c402
17103c40 09000a01 00200900 01080ae8 2c100000 4000db40

```
04008002  22a90000  01044021  2a28b008  08a00a00  02001100
13d8a008  40020201  00101520  b8020260  07084068  0a420414
00800c00  e0002201  00080800  64800024  02000383  00074802
c2e00004  02420400

>D01264
00050040  6b000808  2000050a  40050700  88281051  4002c402
17103c40  09000a01  00200900  01080ae8  2c100000  4000db40
04008002  22a90000  01044021  2a28b008  08a00a00  02001100
13d8a008  40020201  00101520  b8020260  07084068  0a420414
00800c00  e0002201  00080800  64800024  02000383  00074802
c2e00004  02420400

>D01256
0000c012  01802222  07100000  03b00e20  20080010  14002000
08000000  0000240b  50004010  005078c0  00202004  e201c800
42028006  00000045  90620444  00008000  01ac6100  00000020
0a412200  02066403  c0102000  18010110  20008000  18500018
02306018  00280144  104040a0  900004cc  04008420  63060080
2402e000  00234270

>D01240
00000040  81000000  00000100  00810300  80280040  00000800
00002880  08000440  01202000  01100028  40000100  40008000
00028000  02800001  00080400  002a9008  00820010  00000000
1341e010  00002001  00100020  01000a80  05008000  08000010
00001400  00000400  00404000  a4000020  00000200  01000002
24a20000  00800010

>D01228
00000002  01001008  60104008  00000600  00041010  00008004
04080000  00200000  0c000200  000000c0  00200004  40050000
00008804  08000000  01030000  0016000c  00020000  00082000
00202000  00060201  40000000  18048c50  30800000  0010a018
00002400  00110022  00004400  10000000  00000200  00060001
80000008  30000200

>D01223
00000002  01400020  80140300  8c090700  001800e0  000041e0
00009400  80000800  04000306  818000b0  0438000c  40019004
06009001  01d81110  002a1080  001e400c  00b78440  10804000
09c00800  00601001  800c0020  18050c10  27804045  0c600810
50000410  40001002  00000204  100008f2  00000208  81470003
40281000  000003c0
```

>D01211
0003c00a 01007801 00180121 c8090620 2218008c 00004020
0000b000 22301828 06000100 40020be0 00282004 4000b200
06008822 0260a000 00060441 001c040c 140c0000 0200c000
08400000 80201203 e0100000 3e068e00 23804860 0250001e
00000002 c0100000 20000460 900400c0 0802c118 02460013
e0845000 80900ac0

>D01198
0400000a 44000008 20104108 00010600 00041080 02000060
020c0000 02001000 06000000 40000860 04200004 40008000
0080c806 00600000 01300441 000ca00c 10200000 02000000
00a80000 00020200 40000000 18008008 27800080 88548010
00002001 c0000000 00220000 80800000 00000300 00060008
20040000 00000240

>D01196
00440000 00040009 00000040 00380400 00000000 00800401
00028100 10000010 00000100 10808002 00302000 40000480
90008404 00060001 80042000 0000400d 40000000 00004200
00020020 00204000 00000080 00301690 20400000 00008000
20000000 2c000000 24000004 0001044c 10000008 00050009
00040200 00010a68

>D01183
00008002 00000800 00000100 00010200 00000000 00014000
00000010 00000040 00802100 00000850 00200004 40009800
06008004 00000000 00020040 00080008 00040000 02000000
0800800a 00000000 41000000 08020b20 20000000 00400004
00042000 80000200 02080000 00001080 00408000 00260000
c0000000 02020000

>D01180
00040048 81000020 00000800 81010520 00080080 00000800
02010000 08000402 00004002 0050400c 40040200 40008002
80020000 00800004 00000405 01089008 02c00000 00000220
23402018 00002021 00000020 10001000 05008400 08000010
00201402 00000400 00404800 800000a0 000c4200 01040000
24020000 80040090

>D01164
0400c002 80000208 22100128 05810700 30101000 06002040
0a040000 28000000 00400800 00080ad0 08380024 50019800

06808006 08102001 11020041 000c2008 00048080 12000000
08400008 00026601 40100100 38200700 21002021 03410016
00002800 81100400 00120210 10001080 88008200 0006000b
00000000 20020e00

>D01136
00010000 01000000 00000000 00000400 00080000 00000000
00000000 00000000 00000000 00000080 00000000 40011000
04008008 00800000 00000000 00080008 00000000 00000000
02402000 00000001 00000000 10000a10 01000000 00000010
00000000 00000000 00000000 00000000 00000000 00040000
00020000 00000000

>D01133
0081000e 01044808 2000010a 50850e80 00441068 00004800
02040001 01084002 00100900 100808c1 0911800c 40039004
8c008022 00608008 010204c1 021c000c 10008800 02003004
02200400 00c20801 c0080100 3c080620 61840000 00400014
00202000 80400800 40020040 00000000 0140020d 004f0c02
40200800 004484a0

>D01122
00000002 00000000 02004100 10010200 00040070 00004000
00000000 00000040 00000b00 00080840 08100004 40009800
01008800 00010000 40060000 020c000c 08028000 02800000
04200000 00400001 40010120 18008a20 a1801002 00400110
00000400 80002000 00080040 00000000 04000204 00020402
40002000 02000400

>D01119
00010002 47005200 24122d0a 8c110740 00080080 00000000
80001080 20000004 00400880 024e48c0 08302200 c004b080
0601b016 40242103 82183008 100c5008 14820000 02400108
60404000 00401305 40200920 78280e80 13020201 87400018
06042600 84000000 00004040 10000405 0c000200 0006002b
30004000 03c32600

>D01118
00204002 00820200 00120020 04100600 20040000 00002000
00010010 00000000 00022000 00400e40 00244004 61008000
00000002 00002000 20020040 02000002 04841000 20000000
08200002 02020000 c2100000 38000000 20800000 0218001b
00004401 10000300 02204010 00000080 08008200 01060000
0000a000 00030220

>D01097
00008002 00002800 00004100 10000e00 00040020 00100000
01000000 02003001 00000000 00090040 00298004 40000800
0600884a 00100004 00020400 02040104 00000000 02000002
00200008 00000000 40000000 28028700 20802000 0240000c
00100800 c0000200 00040040 80000000 08000104 00060001
80040000 00024200

>D01071
00010000 09001020 00900000 00000c00 00000000 00000000
00080044 00002402 00004140 001001a0 00200000 41011000
0d029008 00000004 00000400 00000000 00000000 00000061
42002010 00000001 00020100 100d0610 00008000 00100018
00212000 00000000 00404000 90000000 00000000 00040611
040a0000 00000200

>D01069
00010004 01005200 00102142 84010700 00080180 00000000
00010000 20000000 02200800 00080cc0 08302018 4003b004
06008012 01202003 a0181188 000c1008 10820000 02000000
04500000 00000a01 80000120 38040e20 53004001 02500018
00000400 80000100 10010000 10000001 09000200 000e000b
2000e000 00442608

>D01064
0003c04a 0100d800 02100100 90010600 001c84a0 00004020
00081980 3204501a 06000100 400208e2 01200204 4000f480
06008832 02608021 00022440 021c000c 110d1000 0200801c
08600000 00008209 40101000 58049ea8 63814040 1050401c
02000000 c4000102 20000000 90040484 00404304 004e0001
e0044000 40e10a80

>D01061
0000600a 03009e09 e1104108 50250710 800c547a 0200c440
020c4800 20181040 9e500b00 700809f0 0d308004 5085d800
8d008926 00618200 a11b0041 0a1f300c 08020260 9688f004
04698002 50620201 41110628 180c8ed8 3d885008 8970241c
0004a402 c021aba3 081a0060 90045075 0643420d 0a060c5b
c0043800 d2022620

>D01027
00432002 05415842 803c4150 85050e00 023c00e6 04184090
10084e68 30003402 10204d21 21918cf7 0521800c c0039002

```
0412b01a  0bea2121  800a14c0  003ececc  102f08b0  43808206
39f12000  4202726b  c00a00a1  3c030f33  27808001  0e540818
14220484  f8020002  4e404240  f2450d78  1e500210  8046805b
759b000a  48aba2a0

>D01001
00008000  00002000  00000100  04010600  00000000  00000000
00000000  02001008  04000000  00010840  00200000  4000a800
02008806  00002001  00000400  00000000  00000000  02000000
00000000  00000000  00000800  28008700  00000002  02400008
00002000  c0000200  00000000  80000100  08000100  00060001
08044000  00020200

>D00998
00000002  80000008  60000008  00400200  00001000  08000000
82000000  00000800  04000200  00000840  00202004  00008000
00008402  08000002  01010041  00100008  00060800  00000000
08800000  00022600  40000000  08000c10  30000000  00002000
00000000  00010002  00000000  00000080  00008200  00060001
00000000  10030020

>D00995
00000002  80000008  60000008  00000200  00001000  08000000
02000000  00000000  04000200  00000840  00200004  00008000
00008402  08000000  01010041  00100008  00060000  00000000
08000000  00022600  40000000  08000c10  30000000  00002000
00000000  00010002  00000000  00000080  00008200  00060001
00000008  10020000

>D00994
00004002  80000008  20000000  00000600  00000000  00000000
02000000  00000000  00000000  00000840  00200004  40008000
00000002  00000000  00000040  00000008  00040080  00000000
08000000  00022400  40100000  08000000  20000000  00000000
00000000  00000000  00000000  00000080  00008000  00060000
00000000  00020200

>D00970
00000000  00000008  20000108  00010200  00001040  00000000
02000000  00000000  00000000  00080840  08000000  40009000
01008002  00000000  41040001  00080008  08000000  02000000
00000000  00020000  00000000  08000a08  01000000  00400010
00000000  80000000  00000000  00000000  00000200  00020002
00002000  00000c00
```

>D00969
00430092 80808020 05480180 11f10613 00088001 00104608
80038190 1006cc08 81800580 20508842 00306a00 7020a400
a810a000 0001a103 8030e802 001c900c 20002c80 12004181
00678030 90a36400 80040000 3a081ea1 60084800 38414028
02042000 8c21880d 40442010 400505cc 0842d438 201e3003
d400500a 442103e8

>D00968
00000000 42000008 20000108 00010200 00001041 00000000
02000000 00000000 00000000 00080840 08000000 40009000
01008002 20000000 41040001 00080008 08000000 02000000
00082000 00020200 00400000 08000a00 01000000 00400010
00000000 80000000 00000000 00210000 00000280 00020002
00002400 00100400

>D00965
00014006 01003810 40100120 00010e00 24000000 80000000
00000c04 00002009 14000000 00000a70 04280004 4000b004
04019080 09102000 20120140 00000010 00040000 02402000
08000000 00000011 48120100 38050600 20022000 0040001a
00140000 c0000000 00000000 900000a0 0a408000 00060001
20104000 04004200

>D00964
00000004 00800008 40000100 00010600 00000060 00008004
00008000 01001000 00400100 00880840 08000008 40808800
80000800 00000000 00040000 0008000c 08408000 02000000
00c00000 00200000 80000000 18008040 21000440 81400010
00000000 a0000000 00008000 40000000 04000080 00060402
80040000 00000500

>D00963
00010002 01040020 00000000 00480000 40010000 04000001
00000080 02000402 01000000 00100000 00100000 40401000
04028008 00000000 00000410 00000000 00000000 00000020
02092000 00020001 00002001 00000280 00008000 00000810
00200000 00000000 00404000 80000000 00000000 01000000
24020000 00000000

>D00960
00000004 00800008 40000100 00010610 00001041 00008004
00008000 00001000 00400100 00080840 08000008 40008000

00000800 00000000 00040000 000c000c 08408000 02000000
00400400 00200000 80000000 18008040 21800440 81400010
00000000 80000000 08008000 00800004 04000000 00060002
80040000 00000580

>D00947
00004002 00405400 00101200 82000600 20000180 00008010
00000000 00000000 14000204 00004960 002c2004 60008000
00018002 00101200 00021048 00180108 10068400 00800000
08000800 00400200 60100000 180c0e90 b2002002 00000018
00000400 00080002 18800000 00000081 00008200 80068011
80000000 00000200

>D00904
00008002 00000000 00000100 00010200 00000040 00000020
00000010 08000040 00002000 00080841 08210004 40009800
03008004 00000400 40060040 00080008 08048000 02000000
08000002 00000000 40000400 48000b00 21000000 00400010
00002000 80000600 02000008 00001080 00008008 00261002
00002000 20020c00

>D00903
00008002 00000000 00000100 00010200 00000040 00000020
00000010 08000040 00002000 00080841 08210004 40009800
03008004 00000400 40060040 00080008 08048000 02000000
08000002 00000000 40000400 48000b00 21000000 00400010
00002000 80000600 02000008 00001080 00008008 00261002
00002000 200a0400

>D00902
00800000 8904f200 00100101 84010d01 803c00c1 00200200
00080180 38a02c08 01000600 011000ca 80200040 4001c000
8002b002 01892000 02001408 391b9008 34210814 80000540
51c16018 0000e201 00000020 70152fb0 17008080 0a10001c
00021403 88808c00 40c04000 90000121 0800c250 00040401
ac0e0000 00204b01

>D00900
00070040 09007020 00100000 80010700 00180080 00000800
02080180 38000402 11004000 00100088 40200000 41019001
0402801a 018a0004 00001408 00081008 10a24020 00000060
62c12010 00000201 00000020 34040e80 13008000 02100018
10001400 00001400 00c04200 90010001 0c000200 00040401
04020000 00008210

>D00893
00010040 81040020 00000900 80010300 40080040 00000001
00000000 28000402 00004000 01100008 00000200 40009000
14028028 02820004 00000404 0008100c 00820000 00000020
03402090 0000a001 00000020 00001a00 0140a000 00000012
00201400 00000400 00406200 80000000 00000200 0000000a
24020000 00000800

>D00892
00092002 81000801 00100300 12010610 228c1020 04102018
800000a0 08000408 00000004 001008c0 00000084 60019000
1c028828 02e09000 21020442 820c004c 00020400 8a800004
05602018 00102001 40100008 18028a18 31808020 80510018
01041001 c2080000 08406200 d0008641 00404004 80068040
24040200 00800a20

>D00889
0009a00a 81000801 00104300 12050210 228c1025 00004410
90000200 00000400 10000100 00582850 08008084 60809800
960288a8 02a19000 20020402 0a4c000e 00028400 02001004
05612018 40102003 41100008 08028a40 3180c020 0040001c
01041000 80002280 88406040 c0001041 00484205 00228002
e4000004 20800e22

>D00887
1801600a 01805801 00100102 12010610 21880020 0400a018
10004000 20081044 00400100 108808d0 01288084 6081d800
140090a8 03b09000 200200c0 880c000c 00068008 82803004
0de02000 10500003 c1120000 18068e50 31816020 0050051c
00044001 c0080080 08000208 900010c1 1000c104 00268443
800a8a20 a0100620

>D00885
00008003 00000240 00000100 00050600 00090040 10004020
00000000 00000000 101881a8 000d08d1 09028008 40018d00
020118a4 00800400 00000080 000d001c 00002000 02a40000
00404000 00800410 80001408 18008122 21004000 81400010
00002000 80000304 40040000 02000040 04000008 0046000a
400c0000 40020400

>D00884
00050440 81040000 00000100 80810700 c03c01c0 00a00801
02002a80 28000400 01200000 011000a8 40000200 40009001

1c028028 028a0000 00000404 002a900c 00a20000 00000000
31c1a090 0000a001 00100020 10001a80 0740a000 08000012
00001400 00001400 00406000 a4000020 00000240 00040000
24e20000 04000010

>D00882
00008002 00000240 80000100 80010600 00080050 00004020
00000000 00004000 10100100 000808c0 08009008 40018900
020110a4 00890400 00001080 020c801c 00201000 02000200
10400000 00000000 80001408 18000120 23000000 85400010
00002400 80002200 000c0200 00000030 04000200 0046000a
40080000 00020400

>D00829
00010000 01000000 00100000 00000400 00080800 00000000
00000000 00000000 00000000 00000080 00000000 40033000
04008010 01000000 00000000 00080008 00000000 00000000
00400000 00000001 00000000 10000a00 03000000 00380010
00000000 00000000 000000a0 90000000 00000000 02040000
00000000 00020200

>D00827
00000000 00000c08 a0000100 00410200 00000020 00024000
02000000 00000800 00000100 00800840 00000800 40008000
00048002 00000008 20000020 00082008 00002000 02000000
04800000 00020000 00000000 08020a20 00000000 00400004
00000040 80008000 00000000 00000000 01000000 00020410
c0000000 00041000

>D00826
00000000 00000200 00000100 00410600 00000000 00000000
00000000 00000800 00000800 000808c0 08102000 40008000
00000000 01000002 00000000 00000000 00000000 02000000
00000000 00000001 00000100 18000000 00000000 81400010
00000000 80000000 00000000 00000000 04000000 0006000a
00000000 00010420

>D00813
0002000a 00005a00 00101100 50010600 001000e0 00080010
00002000 21101100 00100900 100808c0 08188024 40819800
07008002 00100000 c00e0400 000c008c 18008000 86000085
09600008 00000001 00000100 18068a10 2b802000 0054041c
00000000 e0000080 08000010 50001000 04080084 0086404b
a0007280 20000400

>D00810
00000000 00000808 20000100 00010600 00000080 00024000
02000000 00000800 00000100 00000840 00200000 40008000
00008002 00000000 00000000 00082008 00000000 02000000
00000000 00020000 00000000 08020a20 02000000 00400004
00000000 80000000 00000000 00000000 00000000 00060000
c0000000 00000200

>D01984
00000002 44008028 2000652a 10502211 00041060 00004000
16080104 00000008 04020100 0001485d 01008004 42008000
00001806 40640000 81020041 022e200c 24000008 0200030c
80280820 00064000 42100002 1800aa20 21800000 01480000
00242000 90018000 48000048 00000000 00400304 00c60020
40080000 002590e0

>D01981
00030000 01803010 40100100 84010600 00000080 00000004
00000000 22201008 00200100 000108c3 00202000 4003b800
8600880a 00002003 80181409 00080018 10020000 02000000
02002000 00000201 80000000 38048e10 12000003 02500018
00000400 c0000000 40000000 90000001 08000308 00060001
60064000 00802200

>D01977
04008002 00000008 20100108 8a290600 01041080 02004010
0e088010 00000808 00002100 00000840 01300004 40009800
06804026 00401001 01081049 020ca10c 10240000 10004008
08e0000a 10264600 40000000 18041928 338040c0 00000010
00002401 00001000 02040000 00000081 00008208 00468008
40000000 002202c0

>D01974
00400012 01801011 01180102 12b10210 00020009 10004018
01004400 00840014 0400c900 480a2850 0000a001 70008002
20028400 40201001 a0220020 90040004 00028020 06000000
06003208 4000a009 84300000 08000228 20004000 0c500218
00850001 a0084001 04000200 180010e9 60004207 20028000
400600a0 20010220

>D01973
00000002 05804a00 00120102 41910610 00041100 40000810
00002000 00000000 00410980 010948c0 08702004 60018800

18010802 00640800 82200040 00040004 04000000 02008004
00202400 00002201 c0000100 18208018 20800000 9b540019
00042000 80000000 08000040 14000000 04400002 00060002
10040208 01012620

>D01968
00001002 00040a00 80400000 00000401 000c00c0 00000400
00000000 00000000 00000100 00000080 30008008 40000000
00001000 00020000 00000080 000c800c 00000028 00000000
00600000 24000000 80000000 15500000 23800000 04000000
00000200 00000000 00000000 00014030 20080000 00460000
60080000 00008005

>D01966
00014002 01801208 20100102 02010600 21080000 00006010
02000000 22001000 00000900 000808c0 08380004 5001d000
0c00882a 01b01000 00020441 000c0008 00040000 02000280
08402018 00320201 c0100100 18048630 21012000 00500018
00000800 c0000000 00320000 90000081 00008102 0006800b
60060000 00000600

>D01918
0480801a 00884808 2100090a 00b10600 00001080 02110002
02000000 02001000 00000000 00100840 00202004 6000b000
02848c02 80000001 812b0441 000a2008 10022000 02000002
00400800 00026000 c0000000 08028e00 33040040 0840001c
00200080 c0000000 00800000 8000008c 00000700 20060001
a0044000 50010a60

>D01915
0009a002 81080801 80100180 42350610 27880004 00004010
80002200 00080400 02000180 001c2840 0020a084 60009800
16028028 02b01001 a0020c02 000c000e 00020000 02007000
05402010 40b06003 00100008 0e623a00 3180e864 00400118
01043000 81000280 80604000 8400006d 0000400c 00468802
64000800 209102e0

>D01911
00050040 63000808 21500502 c0850700 882800d1 4002c402
12103c40 09000a41 00202900 01081ae0 2c300000 4000da40
1500802a 02a90000 40041028 2a28b008 08a20a40 82001100
17d0a000 40020201 00101520 b8020a60 170840e8 08420414
10800400 e0002301 00080000 74814024 02000283 00074802
c2e02004 02020600

>D01907
0000000a 01c01800 80180510 89090e20 003800e4 0000c000
5000cc03 000008c0 00404100 018000b0 0c00800c 40019004
00001000 038a2120 00081080 101eca0c 002a0020 00000020
19c00000 80205003 80080020 14802850 27800040 0c000014
10008400 40100002 0e000200 10010070 00000208 00460014
c1680000 000082c0

>D01900
00010000 01007100 00120100 04110644 00000080 00000010
00000000 2000000c 00000880 000848c1 08300000 4005b100
06018012 00042001 80180000 00080008 14000000 02000000
00400008 00400201 40000100 38240e20 03000241 83500018
00000a00 80000000 00000000 10000000 0c000000 00060023
20004002 01012600

>D01888
00000004 43800208 2000010a 00010600 00001000 4000000c
02040000 00000000 00200a00 000808e0 48100008 40008000
00004002 20200000 01000001 00208108 00000000 02000000
00080000 00020203 80040300 18002000 40000000 08400010
40000000 80000000 00120000 20000000 01000200 000e000a
00000000 00030400

>D01885
00050040 6b000808 2000050a c0850700 88281051 4002c402
17103c40 09000a41 00202900 01080ae8 2c100000 4000db40
04008002 22a90000 01045021 2a28b008 08a00a00 02001100
13d8a008 40020201 00101520 b8020260 07084068 0a420414
10800c00 e0002201 00080800 64800024 02000383 00074802
c2e00004 02400400

>D01866
0043c292 84408000 04484120 11f02609 002cc101 0012420c
80834890 00046c00 81840580 6056a240 00386264 642028a8
a230b890 0015a703 8410e002 001e900c 22046ca0 32804109
88678020 b0e36450 40142000 7e00cf83 20986800 22600022
06002000 0c30800d 40463080 c00501cc 0842d018 20161803
d400500a 60f303e8

>D01862
0009200a 81000801 00104300 12010210 228c1024 00002010
80000080 00000400 00000800 00182840 08108084 60809000

94028828 03e09000 20020442 024c000e 00020400 06001004
05602018 00102003 40100108 08028a08 3180c020 80400018
01041000 80000081 88406040 c0000041 00404004 00828002
24040000 20900620

>D01844
00050040 05020000 00000000 81e10500 803c0040 00200000
a0002800 08000c40 00202000 011000a0 00002000 4001d000
14008028 03884003 01401402 282b9008 00a00804 8000a000
11c0a000 0800c005 00100020 30000a00 070000a0 5a000054
17000427 00000001 40000a02 a4000420 78028200 00040000
84e00008 00010860

>D01842
04074000 03803018 2010010a 02210f02 80181000 82000110
02000004 0a003644 00310800 002a28c0 09340000 5003f0c0
05809812 00201000 51001221 00082008 98204008 8202a100
00c80000 00420001 80020164 38358620 030010c0 81500018
00010006 c0000401 80000208 90000001 2c300300 0006c003
240c6000 80c00e40

>D01840
00000092 00800400 01104000 05b00600 000c0000 00000000
00020000 00000000 00000000 00400960 00202004 60008000
00000802 00402001 80e00040 00048004 00002000 00000000
00600000 00007e03 c8000000 38008008 20800001 ba400038
02000000 0000010c 00000000 0000000c 08000400 20060010
00040002 00030260

>D01828
00000002 00a40800 00084100 00010210 00041600 80014060
00000400 40000200 02084120 40080850 08000024 4000b002
40008c00 01600088 00020050 000c000c 00000000 42004004
04200080 00600000 c8000000 0e068a68 20a04800 8078001c
08040050 80000005 88000090 18140000 00402008 01860002
c0140000 80000400

>D01825
00010002 81400020 00100000 00491100 403c1440 04200004
00000880 0a000402 01200000 01100020 00100001 4000d000
14028028 02800000 00000410 08281008 00800000 80000020
1341a000 00022001 00000021 00000280 05008000 08520814
00200400 00000022 00404020 b4000020 00000200 13000000
24a20000 00000200

>D01811
00000000 00000808 20000100 00010600 00000000 00020000
02000000 00001400 00000000 00000840 00000000 40008000
00008802 00000000 00000000 00002008 00000000 02000000
00000000 00020000 00000000 08028600 00000000 00400004
00000000 80000000 00000000 00000000 00000000 00060000
80040000 00000000

>D01767
00434292 84408000 04484120 11f02609 002cc101 0010420c
80834890 00046c00 81840580 2054a240 00386264 642020a8
a030a810 0015a503 8410e002 001e900c 22046ca0 32804109
88678020 b0a36450 40142000 7e00ce81 20986800 20600022
02002000 0c20800d 40462080 c00501cc 0842d018 20161803
d400500a 60f103e8

>D01765
0003804a 01003a08 20100388 d4010718 001c50e0 00000000
060c2000 22001448 81500000 000b38c0 08200004 4001b800
07008876 0168a000 c11e1449 020c100c 18a70000 83800004
05e90410 00020201 40010020 38068f18 338040a1 8350001c
10043401 c0001100 08560200 90000001 0c000304 0006084f
a4046200 00822e20

>D01718
00002000 00001009 241a0108 22010219 00021041 00100088
02041000 20000004 02020120 00082a40 00004080 40409880
01008032 00201040 a1080021 804c008e 08020200 42001480
04000000 00120000 00802206 28000a00 11844208 04480092
00a02001 80000980 00020020 08404045 08004204 02020802
00002000 40000020

>D01715
04000002 43001108 20106508 00310704 00091000 82000000
02040000 00000004 00400000 03084841 08200001 40009200
0180f002 600c0080 51000001 0208a008 04800408 02000300
00480000 00020301 00020000 18200604 01000000 83400018
00000000 80000020 40120400 00010040 04000200 0086000b
08206010 83010640

>D01712
0000002a 00801800 00080000 02010600 00080040 0000c000
00008000 00000000 06400100 800000a0 0c00800c 40000000

00009000 00400000 00280080 000c000c 00800000 00008000
00400000 00000000 80000100 10000a40 21800041 00300004
00040000 c0200000 60000000 00040000 00004000 00460000
c0000000 00000a80

>D01709
00010000 01000000 00000102 00010200 00000040 00000000
00000000 00000000 00000900 00080840 08100000 40009800
05008008 00200000 40040000 00080008 08000000 02000000
00002008 00000001 00000100 08000a00 01000000 00400010
00000800 80000000 00000000 00000000 00000002 01020002
00022000 00000c00

>D01704
04010000 43000008 2000010a 00210700 00081000 42000000
02040000 00000000 00000800 00080840 08100000 40009000
0480c00a 20a00080 11000001 020ca00c 00c00000 02000000
02482000 00020201 00000100 18000a00 01800400 00400010
00000000 80000000 00120000 00000000 00000202 0106000a
04020000 000004c0

>D01690
00004002 44003a08 62100129 04810600 20001000 00002040
02040000 02001008 00400800 00090ad0 08380024 50019000
0400c802 00102800 01024441 20048008 00048000 02000000
08080008 00020201 44100100 38268600 20002020 0344801e
00040800 c1000000 00220010 90001080 88408300 0006000b
80040000 20000600

>D01688
00000030 0004c400 20004140 08110600 00010080 01400801
84300280 20001400 01101100 8a184840 40241800 c000a800
12088106 200d0000 00000002 001a000c 34000400 02000300
000900e0 00040208 00000010 08000e84 42008040 04408018
00000008 90000020 00486000 40000050 00e00000 481e0001
24004010 83090288

>D01667
00010000 01005200 00100102 84010700 000800a0 00000000
00000000 20000004 00000880 000808c0 08302000 4000b000
06008002 00203003 80181008 000e1008 10820200 02000000
00400000 00000201 00000120 b8200e00 13004001 83400018
00000400 80000000 00000000 10000001 0c000200 0106000b
20004000 00422600

>D01665
00000000 01000000 00100000 00000400 00380080 00000000
00000000 20000000 00000000 00000080 00200000 40010000
00000002 03800000 00280000 00080008 10000000 00000000
03c02000 00000201 00000000 10000010 07000080 00100010
00000001 00000000 00000000 10000000 00000000 00040000
00020000 00000a00

>D01582
0000800a 00000a08 20100588 50010718 00045060 00000000
060c2000 00001040 80500000 000838c0 08000004 4001f800
03008866 00608000 c10e0041 0a0c800c 18830000 83800004
05280400 00020200 40010020 18028f18 25804000 89500014
00042400 c0000100 08160000 10000020 04000204 0006084b
80042200 02020420

>D01578
08004002 00004038 20004108 00018310 80041041 02000102
06042400 01400200 00400004 001c0850 08000004 40009240
01008802 00000000 61042101 001c200c 18010000 02008004
00282400 00021010 41420000 08008e00 21800040 06504010
00040000 e0000000 08120020 50a04004 006023a0 10020002
02022400 04000400

>D01565
0004800a 01000a08 20100188 50010718 00155060 00000800
060c2000 08001442 80500000 001838c0 48000004 4041b800
03028866 00608000 c10e0441 020c000c 18830000 83800004
25280410 00020201 40010020 18028f18 2180c000 81500014
00243400 c0000500 08564000 90000000 04000204 0006084b
84042200 00820430

>D01549
00010010 01007200 00100102 84010f00 00080080 00000000
00000004 20002008 00000800 000a08c0 08302000 4000b000
0e00b002 00202003 80181008 000c1008 30820010 02002001
00c00050 00000201 00020120 38110e00 13000083 02400018
00012402 80000000 00000000 10000001 08000200 0006000b
20004000 00420600

>D01547
00000000 00c01a00 00020100 00110200 00060041 00000020
00000000 00004002 00000a00 100a1840 08100400 70009000

09008000 00010000 50040000 101c0008 0c009000 02000004
0020c010 00040001 88000100 08160a00 01800000 0060001d
01003000 80000800 20008120 00804004 00800240 01030002
80002100 00010520

>D01513
00004202 00000800 00100100 40010600 20000040 00000000
00000000 00000400 00000000 00080840 08200004 60009000
01008000 00018000 40060040 00080008 08040000 02000100
08000000 00000000 40100000 18000a00 21000000 00400010
00000000 80000000 00000000 00000080 00008000 00060002
04002000 00000600

>D01475
00002000 00000000 00100100 02010a00 00000040 00000010
00000800 08002040 00200100 00082860 08000000 50009800
01008000 00001000 40040000 00280008 08020220 02000000
44008000 10000000 00010020 08010a00 11000000 00400010
00000404 80000000 80000000 24000041 00404200 00028002
00002000 00000600

>D01441
0001e202 00010800 80004100 44550610 09041004 04004000
00002800 02081200 00000100 00010840 00282004 6000d810
82008802 0010a002 80020440 800c0004 00060000 12006004
0c600000 10a00006 40100000 2c02a600 31812860 0240011c
000c0003 c0180000 08000600 800110e0 08c0810c 00460801
c0040800 200102a2

>D01432
1005007a 29809a00 03105112 52710f10 801c1021 00320810
131843c0 3e203601 01010940 003828ca 4931a204 5081d801
9c02899a 01e99002 800604e4 420e914c 24a6020c 06000146
70e12c1a 0000ca01 e0920120 18158e98 2580e0e0 88540018
011c7401 c0209c01 8848ce44 908000a3 00500306 0087840f
24060000 a0a16e31

>D01425
04012000 01007408 2010010a 04010e02 00081081 00000000
02000004 2000200c 00210880 000a09e0 08340000 4003f000
0780b002 00202000 81281001 00082008 10000010 82028000
00c02040 00020201 00000100 383d0610 130000c1 83500018
00010001 80000000 00000008 10000001 0c000302 00060013
20024000 00002600

>D01397
00424292 80808000 05484120 11f10e01 080c8011 00104208
84030090 08846c00 81a08580 20508ae0 04386204 6021a008
a010a806 0011a103 8230c002 023c900c 28062c80 12004101
9867c020 90a36400 c0140000 3e018eb1 20886800 2870022a
02002420 cc20a00d 604c3091 640501cc 0842d658 20161803
de00500a 403103e8

>D01370
0000800a 15c01800 00080000 88090620 00180060 0000c000
00008c00 00000800 00400100 018000b0 0c00800c 40019004
03001008 03880100 c0081480 101c800c 00200020 00000000
19c00000 80203001 80080020 10000150 25800040 08000014
10000400 40000202 00000200 10000060 00000208 00460000
c0282000 000200c0

>D01367
00010002 81400020 00100000 00481000 40281400 04000000
00000080 02000402 01000000 00100000 00100000 40005000
04028028 00800000 00000410 00080008 00000000 00000020
02412000 00022001 00000001 00000280 01008000 00520810
00200000 00000002 00404020 90000000 00000000 03000000
24020000 00000200

>D01364
0000000a 01000800 00004102 30000600 00040020 00000040
01000800 00000000 06000820 40080060 0c108004 40800000
80008800 02208040 00120000 020c001c 00000000 06000000
02202008 00200001 40b02100 08018000 23820000 00d00010
00000000 c0000000 00000060 00400090 00000006 00860002
00220000 a0000420

>D01346
0000c002 00003a08 20100120 84090600 20000000 00006000
02000000 0200100c 00000980 00090ac0 08380004 4000b000
06008802 0010a000 00020440 000c0008 00040000 02000000
08000008 00020201 40100100 38228e20 20002000 8340001e
00000800 c0000000 00120000 90000080 0c008100 0006000b
e0044000 00000600

>D01332
14090040 09000800 00500b02 80010600 2a120004 0000a002
800804c0 01000401 05000800 005848c8 08300200 d0019280

0101801a 00200001 41003022 0008200a 05030400 82000048
00352010 00004205 00100900 5c980ac0 0940c000 10500814
03005000 a4001181 00413000 50800004 02c00082 00060c02
8c022020 00c14f10

>D01325
00010040 00800800 00000100 40011201 00004240 00020000
00001080 01002001 00020100 00480840 08000200 c0009880
01008010 00000000 40042000 00080008 09010800 1201000a
00100000 00000004 00000802 480a0a80 01000000 10400014
02000000 a4000000 00000a00 40000004 00000180 00020002
a0002000 00c10400

>D02698
00050041 6f000008 a000010a 20810300 88281050 4000440e
43943c80 0d000041 00202b00 a1080868 4c100200 40009880
0400c012 22a10000 01082025 062a9108 00820200 0200010c
1358a008 00820a13 00142720 58002aa0 05104040 08420410
42000c00 e4002201 005a0000 e4000024 03000283 0002080a
40a00000 02c10400

>D02671
00205002 01c20600 00120000 00900600 20040200 00022000
00011010 20000000 00022000 00400de0 00644004 71018020
00000002 00000100 a0220140 02000002 04841000 20000000
08202002 02022201 e2100002 18000010 20800000 08180019
00004401 10000100 02204810 10000080 00008200 01060010
0102a000 00012220

>D02655
00002006 01801c08 60004100 080a0610 010c1010 04014004
02008002 01204800 11000100 000000c1 0000000c c0019000
08009847 00810000 000200c0 120d000c 00028000 02800404
00610400 40220200 c0080020 1c058a70 71800843 8260011c
00042401 80002000 480d8000 80000800 04400200 000e0440
c0080004 00020060

>D02581
04004002 00405009 20101708 00410600 200010a1 0000c000
02000000 00000008 00002304 00800840 002c0004 40018010
00808022 00110000 01021041 02180008 00668000 02800000
08a00800 40420000 40100000 18100d30 37002482 08c01018
00000000 80002002 18040000 000000c0 00008200 80060401
c0000004 00000200

```
>D02580
00002004  08400000  001a0100  22011600  00000400  00100000
00000000  20001000  00020100  40000848  00110000  40008000
00008840  00201040  70000000  0804000e  02000200  02020400
06000000  09100000  00802000  08008608  40800200  60480084
00000000  80000002  08800020  08401001  00000004  024e0003
80040000  400400a0

>D02579
00000006  08001800  00004100  00000600  00040000  00000000
00000000  00001004  00000000  00000048  00100004  40008000
80008800  00200000  20000000  00040004  00000008  02000000
04200000  00000000  40000000  08008600  60800000  00000014
00004000  00000000  00000000  00000000  00000000  004e0002
80010000  000400a0

>D02566
00000000  21000000  00100102  00010600  00000000  00000002
10000400  01000001  00100800  000808c0  08300000  40018000
00000000  00200000  00000020  00080000  00000200  02001000
02102000  00000001  00000100  18000010  00084000  00500010
00800000  a0000001  00000000  50000000  02000082  00060002
00020000  00000600

>D02564
00000000  00000000  00000100  00010600  00402000  00000000
00000000  02101000  00000000  00000840  00200000  40008000
00000802  00000000  00100400  00000000  00000000  02000000
00000000  00000000  00000000  08008000  00010000  00600000
00000000  c0000000  00000000  80000000  00000100  00060000
00040000  00020200

>D02563
04004002  00405009  20101708  00010700  20001080  02008000
02000000  00000008  00002204  01008840  002c0004  40018010
00808022  00100000  11021041  00182008  00268000  00800000
09c00800  00420001  40100000  18100d10  37002082  0a500018
00000000  00000002  18040020  900000c0  00008200  88060001
80000000  00000200

>D02562
00000008  01000200  00100102  10510600  00402000  00000000
00000000  22101200  00000800  000808c0  08302000  40008000
```

00000882 00201002 80100400 00060008 00000000 02000000
00000000 00000201 00020100 98008000 00014000 00600010
00000000 c0000000 00004000 90000000 00000100 0106000a
00040020 00010620

>D02561
00008000 00002800 00000100 84010600 00000080 00000000
00000000 02001008 00000000 00090840 00200000 40008800
02008846 00002000 00101408 00080108 10000000 02000000
00000000 00000000 00000000 28028700 12000000 0240000c
00002400 c0000000 00040000 80000001 08000300 00060001
80040000 00020200

>D02560
00414000 01208208 a0180108 00010600 00081200 0000000a
02004000 60200201 00004900 000808c0 083000a2 d0018800
40080412 00000080 01000021 00040008 00004000 02000008
08000000 00020205 00000900 18000008 00000000 00480010
08000010 80000200 04020014 18040000 00002200 0116020a
08000008 08400600

>D02559
00004002 00405001 40101700 00010600 24004080 00008000
00000000 00000000 00000204 00000850 002c0004 40018000
00008000 00100000 00021040 00180008 00068000 02800000
08800800 00400000 40100000 18100d10 37002082 08400018
00040000 80000002 18000000 000000c0 00408200 80060001
80000000 00000200

>D02558
00040000 46000008 60000108 00010600 00201000 00000000
02000000 02001000 04000200 00080840 08200000 4000d000
14008a02 20000000 01010001 001a0008 00020000 02000000
01480000 00020201 00000000 08008e10 15000000 0044a018
00000000 80010022 00000000 80000000 00000302 00060003
80040000 10000600

>D02556
0202000e 00800208 c0101900 12010601 000806a0 0000c014
40000080 000d81c8 00020d00 00c948c0 08308a08 d0818600
04000050 00801001 01a22081 000c000c 05428200 06002280
04c02018 10300001 a0000900 580000e0 23004448 8d410010
02801800 8d100064 08200008 01001365 04004004 04a6940a
e8000880 20010f11

>D02487
00008002 00000010 00000000 00000e00 02000000 00000000
00000000 01002001 00000000 00000842 10200034 40008800
02000004 00000008 00000040 00000000 00060800 00000000
08000000 00000000 40120004 28000900 30010000 00000010
00502000 00010000 40080000 00002080 00208090 00060000
00000000 06024000

>D02451
00000002 01800200 00101102 00810610 04080160 00000000
01002000 00001000 80500900 108818c0 28108008 50818800
80001800 00b00800 00030080 000c0004 00408000 06000000
00c82008 40010201 80100500 18008010 21000440 81400414
00040000 c0000208 00000000 10000080 84000006 0046040a
400c0004 20020400

>D02441
04030002 10000008 2000084a 00380200 004c1000 20808000
020a0000 00008018 00400000 00009844 00002a04 50008000
00800226 02620003 810020c1 000e040c 00000000 00000080
00200010 01024000 40000000 48000880 60800080 02010200
00042000 2c006000 40000000 0001080c 00000200 004a0000
00000000 01c10460

>D02418
00004026 42000a08 60100108 00010e00 20001000 c0026012
02002004 00002809 04200b80 000808c0 08300004 40028000
00009222 20000000 01030141 001c2008 00068000 82400020
08080018 00020241 40100100 18238e30 30020000 81402015
00151000 80010002 00040000 90001080 04008200 0006080b
c0080080 30004600

>D02375
00000200 00008000 00000100 00010600 00402000 00400000
00100000 02001500 00000000 00000840 40200000 40008008
00000802 00010000 00000400 00000000 20000000 02000100
00000000 00000000 00000000 08008008 00000000 00400010
00000000 c0200000 80480200 82800000 00100100 00060000
04040800 00000200

>D02368
00000002 01400000 08100000 88090600 00180060 00004140
00009412 00000800 04000100 818000b0 0400000c 40019004

00009000 03c80110 00281080 001c800c 00200000 00000000
09c00000 00201001 800801a0 18000010 25800041 08700810
10000400 40200002 000002a0 90000060 20000208 09460000
40280020 000006c0

>D02356
00000000 43000008 2010010a 00010600 00001000 c0000000
02040000 20000000 00100880 000808c0 08300000 40018000
00004002 20200000 01000001 0004800c 00400000 02000000
00082000 00020201 00000100 38200010 00800400 81500010
00000000 80000000 00120000 10000000 04000200 0006000a
04000000 00002680

>D02355
0000000a 00004800 00001100 50010600 000000e0 00080000
00000000 01101100 00000100 000808c0 08088024 40819800
03008000 00100000 40060400 000c008c 18008000 02000005
00200000 00000000 00000000 18068a00 21802000 0044041c
00000000 e0000080 08000010 40001000 00080084 00060043
80007200 20000400

>D02350
00000000 42000008 20000108 00010200 00001041 00000000
02000000 00000000 00000000 00080840 08000000 40009000
01008002 20000000 41040001 00080008 08000000 02000000
00082000 00020200 00400000 08000a00 01000000 00400010
00000000 80000000 00000000 00210000 00000280 00020002
00002400 00000400

>D02342
04000000 00000008 a0100108 00010600 00001090 02004000
02040000 00000040 00002100 00000840 00200000 40008000
00800002 00010000 01200001 02082008 10200000 02000000
00800000 00020200 00000000 18000020 07000080 08400010
00000401 80002000 004a0000 00800020 00000200 00060000
40000000 00000240

>D02341
00008002 00000800 00000100 00010200 00000000 00014020
00000010 08000040 00802100 00000851 00210004 40009800
06008004 00000400 00020040 00080008 00040000 02000000
0800800a 00000000 41000000 08020b20 20000000 00400004
00042000 80000600 02040000 00001080 00408008 00260000
c0000000 00020000

>D02335
00000000 00000828 00000102 00010200 20000000 0080800a
20000001 00000403 81000800 00980840 08100000 40009000
00028000 00200000 00000400 00080000 00000604 02100020
00312000 00000801 00400100 080202c0 0010c000 00400014
01000000 84000001 00400000 00000000 00000000 00220002
84004000 00000410

>D02333
14004042 01a0b809 a208050a 50c50f10 842e1277 0250c920
12806800 4200124d 02286310 41000c70 41290144 4060e010
40808c02 0ab18480 112200c1 0a2efd8c 048680c8 6e808044
19e8a800 00421803 c0160020 18048e48 a5a1300a 0a58811c
08144440 80006001 280a00d5 ac9410a0 0340a304 01060c0b
c0a0c2a0 82014228

>D02328
00030006 10004c08 22163128 30010701 810c00e0 800080e2
02000480 00018209 06420000 d00c18d0 08018a0c 70019000
0100b0b3 01e08020 400620c0 020d240e 08200000 0200008c
00200010 00620260 40001020 dc010ad8 61804801 02710090
06100002 cc000101 58000040 90080004 01800104 00161642
00306000 00415620

>D02323
00008008 00000008 20100108 00010600 00001080 01000000
02000400 32001000 00001300 00000850 00200000 4000a000
82008802 01000000 01000401 00082008 10008000 02000000
00000000 00020300 10000000 08008a08 02000000 00420000
00000000 c0000000 00000100 90000000 00300300 00060000
80144000 00010208

>D02321
00000002 80001008 2000440a 00300200 80041000 00000000
02008010 00000000 00002000 20000840 8030a004 40008000
00000c02 08400001 81080041 0006400c 00040000 00000000
08200002 00024400 40000000 08048808 20800000 80800000
00000080 00000000 22008000 0000008c 00008208 00060000
00040000 00010160

>D02315
00010000 01000000 00000000 00000000 00080000 00000000
00000000 00000402 00000000 00100000 00000000 40001000

04028008 00800000 00000400 00080008 00000000 00000000
00402000 00000001 00000000 00000a00 01008000 00000010
00200000 00000000 00404000 80000000 00000000 00000000
24020000 00000000

>D02308
00000000 00000000 00000000 00000000 00000000 00000000
00000000 00000400 01000000 00000000 00000000 40000000
00008000 00000000 00000000 00080008 00000000 00000000
00210010 00000000 00000000 00000a00 00000000 00000000
00001000 00001000 00400000 00000000 00000000 00000000
04000000 00000000

>D02304
00010000 01000000 00000000 00000400 00080000 00000000
00000000 00000000 00000000 00000080 00000000 40013000
04008008 00800000 00000000 00080008 00000000 00000000
00402000 00000001 00000000 10000a30 03000000 00000010
00000000 00000000 00000000 00000000 00000000 00040000
00000000 00000000

>D02290
00002000 00000000 00100100 02010200 00000040 00000010
00000000 00000000 00000100 00082840 08000000 50009800
01008000 00001000 40040000 00080008 08020200 02000000
04000000 10000000 00000000 08000a00 11000000 00400010
00000004 80000000 80000000 00000041 00404000 00028002
00002000 00000e00

>D02289
00010000 81000020 00000000 00000000 00080000 00000000
00000080 00000402 01004000 00108000 00000000 40001000
04028028 00800004 00000400 00080008 00000000 00000020
02412000 00002001 00000000 00000280 01008000 00000010
00200000 00000000 00404000 80000000 00000000 01000000
24020000 00000000

>D02267
00000000 00000800 00000000 00000000 00000000 00000000
00000000 00000000 00000000 00000000 00000000 00000000
00008000 00020000 00000000 00000008 00000028 00080004
00000000 00000000 00000000 05400800 00000000 00000000
00000000 00000000 00000000 00014000 00000000 00000000
04000000 00008804

>D02258
1801600a 01805801 00100102 12010610 21880020 0400a018
10004000 20081044 00400100 108808d0 01288084 6081d800
140090a8 03b09000 200200c0 880c000c 00068008 82803004
0de02000 10500003 c1120000 18068e50 31816020 0050051c
00044001 c0080080 08000208 900010c1 1000c104 00268443
800a8a20 a0100620

>D02229
0485009e 01804408 21001108 15b10610 01285160 0001c002
06033800 01080300 06400900 900e18f0 a130a004 6000d040
1c808022 02602801 81220081 020ca10c 10002020 0680a00c
01600c00 40666041 c5000102 38010a60 a5804860 2a700438
06142002 c0000005 28930800 0004108c 08404706 20460c02
41000802 402506e0

>D02214
00050040 6b000808 2000050a 40050700 88281051 4002c402
17103c40 09000a01 00200900 01080ae8 2c100000 4000db40
04008002 22a90000 01044021 2a28b008 08a00a00 02001100
13d8a008 40020201 00101520 b8020260 07084068 0a420414
00800c00 e0002201 00080800 64800024 02000383 00074802
c2e00004 02420400

>D02194
00410000 43004000 20120020 00100400 00000082 00100000
000002a0 28000404 05000400 001040c0 00200040 40033000
06038002 01040000 80080400 00080048 14010000 01000002
00010010 00400201 40008000 10000e80 03008700 00300018
04001200 00000400 00404000 90040500 00101000 00040021
24004000 43010a00

>D02193
00000000 00000000 00000000 20140400 00000004 00080000
00004000 00000000 00000000 02000020 00200000 40000010
00000000 00060424 00000000 00080068 00000020 00800001
20000000 02000402 00000020 06000a00 00000000 00000000
00480400 00040004 02008000 20010000 00002000 00040000
00200000 02018200

>D02176
00020000 00000000 00100000 00000400 00100080 00000000
00000000 00000000 00000000 00000080 00200000 40001000

14008000 00000000 00000000 00080008 00000000 00000000
01400000 00000001 00000000 10000a00 03000000 00000010
00000000 00000000 00000000 10000000 00000000 00040000
00000000 00800a00

>D02173
04001008 c7000628 6010090a 90210702 00181041 c2802000
12040001 2a001642 90044880 019818c8 08300308 40018040
00824882 20a10884 110e0425 120ca018 08004000 26108020
0f682410 8002aa01 04020100 18018010 0100e000 02d48410
00201480 c0004409 00624200 90000000 04000302 0086c80a
64060008 00002e40

>D02168
00006002 03008a09 a1104100 50250710 800c547b 0200c440
02004800 02181440 9e500900 700808f0 05308004 4005d800
8d008926 00618200 a01a0040 0a0d300c 08020068 9688f004
04698002 50620201 41110628 18008a58 3d805008 89700414
0004e402 c020ab81 08080060 90045075 0643430d 0a060c4a
c404b800 c2020220

>D02166
18004002 42001808 60000122 04010600 000000a0 000b8040
12000440 01000201 00c00000 00080a50 08200004 40008000
00008002 00202000 00120040 30082008 10248200 02001001
08102000 00020200 44100000 28060240 22084018 02420016
00840000 a0000001 00080000 40001080 2a488080 00060002
82000000 20030600

>D02115
1003c00a 01a05800 00180902 98090e30 0a1a10c8 0010c000
0000e000 22001801 10400100 000208c0 41210004 4000b000
06008822 02108000 008e0440 100c010c 18040000 02000000
08400000 40200201 40100000 1c068e40 23810840 0040001c
00140000 c0000001 08008000 9000008c 0000810c 00461801
e0044000 80800ac0

>D02110
0000800a 00000a08 20100188 50010718 00045060 00000000
060c2000 00001000 80500000 000808c0 08000004 4001b800
03008866 00608000 c10e0041 020c000c 18810000 83800004
01280400 00020200 40000000 18028f18 21804000 81500014
00042000 c0000100 08160000 10000000 04000204 0006084b
80042200 00020c20

>D02068
0000000a 21004000 00004102 10810210 00041120 00000000
0100a000 00000001 10400800 00080840 08108004 40808000
00000800 00600800 00020040 020c0004 00000200 06000004
02202408 00000001 40100100 08008008 20804000 80400010
00040000 80000001 08008040 00000080 00400006 00860002
00040000 20020440

>D02042
00002002 00000001 00004100 10000210 00041062 04004048
10008801 00000000 06000100 40810070 05008004 40800000
00018800 00a00000 20160041 820c000c 08420000 02001004
04a00000 40280002 40000000 88008020 31804401 02500400
00840001 c0080000 08008640 00000045 00418004 00460000
40040000 00020280

>D02017
0000002a 00801800 00080000 02010600 000800c0 0000c000
00008000 00000000 06400100 800000a0 0c20800c 40000000
00009000 00400000 00280080 000c000c 00800000 00008000
00400000 00000000 80000100 10000040 23800041 00300004
00040000 c0200000 60000000 00040000 00004000 00460000
c0000000 00000280

>D02008
0001800a 4680fa08 22100108 55810e10 20041220 0018000e
03042400 2300300d 00600a80 000908f0 48318004 5081b000
1e00c8c2 2060a801 810204c1 0224818c 00000200 02000027
00380408 00020a03 c0060300 3823a608 20804021 9b468018
40143800 e0000001 08324000 f0000000 0f000384 0086000b
20044200 20014620

>D03773
00018008 01805200 02100102 14010600 200800a0 00000000
01001000 22001004 00400880 000908d0 08302000 5001b000
060088c2 00202003 80180480 000c0008 10000200 02000020
00400008 00000201 80020100 38208e00 23004041 83400018
00000800 c0000001 08004000 90000000 0c000100 0086000b
20044000 00402600

>D03772
00010400 03005200 00100102 00050620 00080080 00000010
00000000 20000000 00000800 000808c0 09300001 4011b000

0600801a 01a20000 82081000 00081008 10000028 02100003
c2406010 00000201 00000100 19140e10 03c00000 80500018
00002002 80000000 00000400 10014000 000c0200 00060303
30864000 0000c621

>D03769
00010000 01005200 00100102 04010600 00080080 00000010
00000000 20000000 00000800 000808c0 08302000 4001b000
06008012 01202003 80180000 000c0008 10000000 02000000
00400000 00000201 00000100 38040e00 03000001 02500018
00000000 80000000 00000000 10000000 08000000 0006000b
20004000 00402600

>D03767
00010002 01805b00 20122508 08110644 00080080 82002010
80000000 20000000 84400000 020a48c1 00600000 600db100
06019012 41040100 82080000 00080008 14001008 02000100
00404000 00420301 c0000000 18060e00 03800300 0450001d
00042200 80000000 00004000 10000000 00000000 00060021
b0004002 03112220

>D03765
00010000 c1005200 20100102 8c110740 000800c0 02002010
80000000 20000000 84080800 020e48c0 08302000 400db000
06418016 11242103 80181008 400c1008 14821000 02000000
00400000 00006301 40000120 38040e00 13000301 06504018
00000400 80000000 00004000 10000001 08000200 0006002b
20004004 03432600

>D03763
00000000 01005100 00120000 00100444 00000080 00000000
00000000 20000000 00000000 000040c1 00200000 40053100
02018002 00040000 80080000 00080008 14000000 00000000
00000000 00400201 40000000 10040e00 02000200 00100018
00000200 00000000 00000000 10000000 00000000 01040021
20004002 01012200

>D03760
00014022 01005000 00101100 00010700 20080080 00002050
02002000 20100104 00000880 000a08c0 08380024 4001b000
06008032 01100000 800a0140 0008100a 10868000 82000000
08400008 00000201 40100120 18208e00 23002040 81500019
00040c00 80000080 08000010 90001080 04088200 00060003
20005080 20002600

>D03758
00014002 01004200 40101122 04010608 20080180 00002040
03000000 20100100 00100800 08080ac0 08382024 4001b000
0600800a 00b02803 801a0040 080c000a 10248000 02000000
08402008 80000201 44100100 38000e30 23002001 02d0001a
00000000 80000080 48000010 10001080 08088000 0006000b
20005080 20402600

>D03756
00010000 01005200 00100102 84010700 00080080 00000010
00000000 20000000 00000800 000808c0 08302000 4001b000
0600801a 01a02003 80181008 000c1008 10820000 02000000
02402000 00000201 00000120 38040e10 13000001 02500018
00000400 80000000 00000000 10000001 08000200 0006000b
20024000 00422600

>D03753
00010000 01005000 00100000 84010700 00080080 00000010
00000000 20000000 00000000 00000080 00202000 4001b000
0600801a 01802003 80181008 00081008 10820000 00000000
02402000 00000201 00000020 30040e10 13000001 02100018
00000400 00000000 00000000 10000001 08000200 00040001
20024000 00002200

>D03752
00018008 01805200 02100102 94010700 200800a0 00000000
01001000 22001004 00400880 000908d0 08302000 5001b000
060088c2 00202003 80181488 000c1008 10820200 02000020
00400008 00000201 80020120 38208e00 33004041 83400018
00000c00 c0000001 08004000 90000001 0c000300 0086000b
20044000 00402600

>D03751
000040a2 01004000 001a0102 62014602 20040200 00100110
00002000 22001400 05060900 000808c0 08388024 50019820
01009802 00701040 40020540 208c000c 01040200 86030006
0c212010 00800201 40902102 18218e18 20c02000 81580091
00041210 c0011200 004000b0 98001081 04608106 01068203
24042000 e0000610

>D03743
00010010 01007200 20100100 8c310f40 00080080 00000000
00000004 2000200c 00000800 020e48c0 08300000 4001f000

0e01b00a 008c2101 80181008 000c1108 34a20018 02000001
02c02058 00400301 40000120 38350e10 17000283 8f500018
00011e01 80000000 00804000 10000020 0c000200 0006002b
20024000 03012600

>D03741
04050002 01c07200 00104100 15810e10 041c02e0 00118010
00002020 2200300d 00c00b80 00090ac0 28318004 5001b800
0e00881a 00102801 811a0400 021e010c 10020000 0e080002
00602818 40000a01 e0000d00 38278e70 23800003 93500018
00141800 c0000202 00000040 90200120 0c000304 0046000b
e0064000 20006e00

>D03738
04000002 01007608 20100108 05810610 040812c0 00000000
02042020 2000000c 00400980 00090bc0 28308000 5001b800
0e008002 00902801 811a0001 000c000c 10000000 0e000000
02c02018 40020201 20000d00 38340610 27000083 93500018
00041801 80000200 00120000 10000100 0c000204 0046001b
60024000 20002600

>D03736
04002007 00000a08 20000108 80058e42 05085080 00004002
02800000 00003209 00400104 500d08d0 09388004 40008010
80808a22 08300000 01211049 001c610c 10040000 0a800000
88400000 00220040 44000180 18058428 33826800 83410030
00342400 c0000000 20170000 110008c1 0400831c 0046010b
40060000 800046e0

>D03735
00008006 01001001 00100b20 02110e10 01000040 00000018
00000104 30082001 04424100 000148c2 00288084 c0038800
02019003 00341000 20021040 800c0004 040e0400 02412000
0c002020 42200201 40280100 18050410 21022100 00780018
00150001 91080000 00000208 100000c1 0001c004 00468001
400a0800 01016240

>D03734
00400006 01000008 20100100 00810e00 00150241 00b00000
02800a24 22003601 01600d00 010908f0 0b380004 40419800
98009822 08000000 00000440 00282048 00064001 02400000
49032210 00020201 440001a0 18118e10 21020000 00500010
84152400 c0000000 00040000 b4840580 00408300 20060002
240c0000 80004610

>D03733
00000000 01000000 00100102 02010600 00000000 00000010
00000000 00000400 00100800 001808c2 08100000 40018000
00000000 00201000 00000000 00000000 06000000 02000180
00000000 04100001 00000100 18000000 00000000 20400110
00000000 80000000 00000000 80000001 00000000 00068402
04000000 00030600

>D03731
00000000 00000000 00100100 00010600 00402000 00000000
00000000 00100000 00000880 00080840 08300000 40008000
00000002 00000000 00100000 00000000 00000000 02000000
00000000 00000201 00000100 18200000 00010000 81400010
00000000 80000000 00000000 00000000 04000000 00060002
00000020 00000600

>D03728
00020090 01805400 01100000 01b00e00 00088080 00000000
00020000 20002000 00200200 004009e2 00202010 7003b000
0e009002 01000001 80b80000 00188008 10006004 00000000
00600000 00007603 80040000 380d0e29 02000001 3a500038
02000000 0000000c 40000000 1000000c 08000412 20060011
a0184002 00812260

>D03722
00010000 01003200 00100100 04410e00 00000000 00000010
00000000 20002808 00200a00 000808c2 08302000 4003b000
06009012 00002003 80000000 00140000 00000000 02000000
00000008 00000201 00000100 38250620 00000003 83500018
00000800 80000000 40000000 10000000 0c000010 0006000b
a0084000 00012620

>D03720
0800a002 0000c800 00004100 00810610 04040040 00082060
04006000 05001001 06400100 400808e0 6c01800c 60018800
82009864 00700000 80120440 000c0204 00070000 02800005
84600000 50002000 c0000000 18038518 31800000 80700418
00042000 e0000600 00040020 40000050 00404084 00460042
40040004 a0020420

>D03717
00002010 00801001 051a0100 23b10619 000a0009 00100088
00001000 20001004 02020100 00402a40 00002080 60409080

00008000 00201041 a0200020 8044008e 02022200 02001400
04400000 00106400 80802000 18000600 10800208 1c480098
02000001 80000004 00000020 0840006d 08004404 22060000
00000000 40010a60

>D03716
00002010 00801001 051a0100 23b10619 000a0009 00100088
00001000 20001004 02020100 00402a40 00002080 60409080
00008000 00201041 a0200020 8044008e 02022200 02001400
04400000 00106400 80802000 18000600 10800208 1c480098
02000001 80000004 00000020 0840006d 08004404 22060000
00000000 40010260

>D03714
00008002 00000804 00100304 02010200 00000041 00000010
20000010 08000040 00002000 00080841 08210004 40009800
03008004 00009004 40060040 000c0008 08048400 02000000
08000002 00000000 40000400 48000b00 21000000 00400010
00002000 80000600 02000008 00005085 00008008 00269002
00002000 20020600

>D03712
00000000 00801200 00020100 10910301 00400041 00000000
82000080 00005440 01802800 00180840 c810a100 6000c000
84008000 01010001 823c0003 09089008 0c020414 82000000
6441c018 00002001 80010120 09040a80 05808000 0840001d
00001400 80000400 20402000 40000420 00404240 20020002
0400a000 00810430

>D03710
0002a002 00811201 00024100 10910210 00041020 04000008
01000001 00480004 00000100 10000850 0000a084 60008810
06004808 00210000 a0220000 820c0004 04020000 02003004
04200008 10402006 c0000000 08008100 31800000 08400009
00040803 80080200 08040240 00000060 0040c004 00020400
00000820 01030020

>D03689
00006036 00802208 a1100108 02b10600 01081010 00004012
02440400 00001400 06000104 000128f0 09002004 7000800a
00018832 02001201 81200041 000c200c 00020800 02801000
04400000 00222008 c0100000 3801a020 30800800 8b404008
00000000 c0000040 30c24000 90000869 04004304 20468008
40260200 800126a2

>D03670
00020000 01001010 40100000 00000400 00000000 00000000
0c000000 20000000 00200000 00000080 00200000 40031000
04008006 00000000 00000000 00000010 00000000 00000000
00000000 00040201 00000000 10040620 00000000 00100018
00000000 00000000 00000000 10000000 00000002 00040005
00000000 02802200

>D03643
00050000 81040020 00100302 02010300 c0180041 00000811
82000080 08000402 00004800 01180840 48100000 40009000
14028028 00a21004 00080402 0008100c 00020600 020000a0
23402090 00102001 00000130 08000a00 01408020 00400012
01201400 80000400 00406000 c0000001 00404242 0822800a
24020000 01800610

>D03601
00030040 01005000 00100100 80010700 00080080 00000010
00000000 00000000 00000000 00020080 00200000 4001b000
06008032 01800000 80081008 00081008 10820000 00000000
02402000 00000201 00000020 10000e00 13000000 00000018
10000400 00000000 00800000 00000001 00000200 00040003
20024000 00000200

>D03440
00010000 0100520a 4010050a 00050600 00080084 00000010
00004000 20000000 00000810 000808c0 08300000 4001f000
0680801a 812200b2 822a0000 00082608 100e00a8 02100000
00406000 00003203 01000100 1d140e10 23400000 00500018
00000008 80000100 02000000 10014000 00040000 01060287
30404000 0000a601

>D03433
00000040 01000000 00100700 80010700 003800c0 00000010
00000000 20000000 00000000 01000080 00200000 50019000
04008002 03800000 00281008 00089008 10860000 00000000
01c02000 00000201 00000020 10000a10 17000080 08100010
10000401 00000000 00000200 10000021 00000200 0004000a
00020000 00022a00

>D03350
0400c202 00801008 20100108 8a090600 81041080 02004010
0e008010 00000c08 01012100 00000840 013c0004 60009800

06808006 00511001 01081049 000c650c 10240000 10004108
08e0000a 10264604 40100000 18041f28 33c060c0 00008010
00002401 00001000 22000040 00400081 00008208 00468001
44000000 002202c0

>D03218
00000002 01800a08 20024100 10111e00 01240020 002c0022
c2020080 0a005408 00000200 00000840 00200104 60008000
10028802 00400000 90020082 0204200c 04241408 02000004
10620400 00220000 d0000000 1e028608 20800801 0061001d
00102000 92000001 00422040 c0000200 00c10304 00060001
84040000 00114220

>D03115
00000092 00800400 01104000 05b00600 000c0000 00000000
00020000 00000000 00000000 00400960 00202004 60008000
00000802 00402001 80e00040 00048004 00002000 00000000
00600000 00007e03 c8000000 38008008 20800001 ba400038
02000000 0000010c 00000000 0000000c 08000400 20060010
00040002 00030260

>D03077
00014002 01004200 40101122 04010608 20080180 00002040
03000000 20100100 00100800 08080ac0 08382024 4001b000
06008002 00302803 801a0040 080c000a 10248000 02000000
08400008 80000201 44100100 38000e10 23002001 02d0001a
00000000 80000080 48000010 10001080 08088000 0006000b
20005080 20402600

>D03034
00010000 01000000 00000000 00000000 00080000 00000000
00000000 00000000 00000000 00000000 00000000 40001000
04008008 00800000 00000000 00080008 00000000 00000000
00402000 00000001 00000000 00000a00 01000000 00000010
00000000 00000000 00000000 00000000 00000000 00000000
00020000 00000000

>D03012
00000040 44020008 20400300 01610600 20000021 04000000
82010001 00001601 00004100 00980840 08002200 40849800
01008003 08200003 40000000 2008a008 08000000 02002004
00a00000 00024000 00000000 1a000600 00004000 91408011
00000000 80000100 00010200 80800004 04002000 00060402
04002002 00010660

>D02835
00000002 00000000 00004000 02000600 00040000 00002060
00000000 00000000 06000002 400008e0 04000004 4001a000
00008800 00600000 00100040 040c400c 00000000 40000000
00200000 00000000 40000000 18008a18 22800000 84300002
00000000 40000000 00000000 00000010 00000000 00060000
00040000 00000800

>D02769
0000000a 01c01800 80080510 89090e20 003800e4 0000c000
0000cc00 00000800 00404100 018000b0 0c00800c 40019004
00001000 038a0120 00081080 101eca0c 002a0020 00000000
19c00000 80205003 80080020 14000850 27800040 0c000014
10000400 40000002 0e000200 10010070 00000208 00460014
c1680000 000080c0

>D02756
00050040 63000808 21500502 c0850700 882800d1 4002c402
12103c40 09000a41 00202900 01081ae0 2c300000 4000da40
1500802a 02a90000 40041028 2a28b008 08a20a40 82001100
17d0a000 40020201 00101520 b8020a60 170840e8 08420414
10800400 e0002301 00080000 74814024 02000283 00074802
c2e02004 02000600

>D02731
0481009a 40804808 e1101588 1db50610 01085160 0001c006
02c39800 01280b00 16400100 11060871 8020a004 62808840
0080c082 0050a001 81220881 000ca10c 0000a820 0780a000
00600808 40666001 80000102 28402040 a5800864 2a704178
02040402 80200004 30800820 100418ac 0800870c 20c61c00
43000a82 602706e2

>D02729
04002000 44800808 20000109 00010602 00001080 00000000
02040000 02001000 00000000 00000840 00200000 40008000
0080c802 00000000 01005401 20088008 00002000 02000000
00080000 00020200 80000000 08068600 12000000 0044800c
00000000 c0000000 00220000 80000000 00000300 00060009
80040000 00020200

>D02709
00002010 00801001 051a0100 22b10219 00020009 00100088
00001000 20000004 02020100 00002a40 00002080 60408080

00008000 00201041 a0200020 8044008e 00020200 02001400
04000000 00102000 80802000 08000000 10800208 0c480088
00000001 80000000 00000020 08400069 08004004 22020000
00000000 40010020

>D06238
00004003 80008808 20000108 980d0600 0204100c 00004000
4200a000 02001808 00000100 00000840 01200004 4000d000
00004822 08600000 01000041 000c800c 00040080 02000004
08600000 00222c00 40120000 0c008028 20810840 00600000
00040000 80000000 00040040 80000080 00408308 00460008
40040020 000202e0

>D05458
00000000 00000000 00000100 00010600 00000000 00000000
00000000 00000000 00000800 00080840 08100000 40008000
00000000 00000000 00000000 00000000 00000000 02000000
00000000 00000001 00000100 18000000 00000000 81400010
00000000 80000000 00000000 00000000 04000000 00060002
00000000 00000400

>D05407
0000000a 00801800 00080000 00000600 00180040 0000c040
0000b004 00000800 06400100 800000e0 0000800c 40011000
00009000 00c00000 00080080 100c000c 00000000 00008000
09400000 00200001 80000100 1c000050 21800840 00700014
00000000 80200000 60000000 00040000 00004008 00461000
c0080000 80000280

>D05341
00010000 01000000 00000000 00000000 00080000 00000000
00000000 00000000 00000000 00000000 00000000 40001000
04008008 00800000 00000000 00080008 00000000 00000000
00402000 00000001 00000000 00000a00 01000000 00000010
00000000 00000000 00000000 00000000 00000000 00000000
00020000 00000000

>D04983
00000000 00000000 00000000 00100440 00000000 02002000
00000000 00000000 84000000 00004000 00000000 40083000
04018000 00040000 00000000 00080008 04000000 00000000
00400000 00000001 00000000 10000a00 03000300 00000010
00000000 00000000 00000000 00000000 00000000 00040000
00000000 01010000

>D04966
00000000 00000000 00000000 00000800 00000000 00000000
00000000 00002000 00000000 00000002 00200000 00000002
00000000 00000000 00000000 00200000 00000000 00000000
00000000 00000000 00000100 00020000 00000000 00000000
00000000 00000000 40000000 20000000 00000010 00040000
00000001 00000000

>D04292
04001008 c7000628 6010090a 90210702 00181041 c2802000
12040001 2a001642 90044880 019818c8 08300308 40018040
00824882 20a10884 110e0425 120ca018 08004000 26108020
0f682410 8002aa01 04020100 18018010 0100e000 02d48410
00201480 c0004409 00624200 90000000 04000302 0086c80a
64060000 00002e40

>D04197
00000002 01400000 00140000 86010600 00180060 000041e0
00000400 00000000 06000100 818000b0 0c00000c 40018000
00009000 03c81110 00aa1080 001c800c 00200000 1000c000
09c00000 00001001 80080020 10000010 25800041 08300010
10040400 c0000002 20000200 10040862 00004200 01470000
40281000 00000280

>D04031
00006022 03008a19 81504100 50250710 800c547a 0200c440
0000c800 00181040 9e500900 700808f0 0d118004 4045d810
8d0189a2 00618200 e01a0140 0a0d301c 08020060 9688f004
246b8002 50600001 41130629 98188a58 3d805008 89700494
0004a402 c038ab81 08088260 90047075 0643420d 0a060e4a
c0443800 c6000060

>D04029
000040a6 00a29208 201a0b00 52080e00 010c0240 00104012
10048400 0000140d 04066904 000158f0 0939806c 6010c002
0000ba01 02501240 00060040 080c210c 09040408 02c00008
08600000 0022000c c0902001 1805a668 21c12840 8308008c
00104010 c0012041 28228030 98010881 0400c10c 01468b0b
c4268008 c01227b0

>D04028
00010040 01005000 00100100 80010700 000800c0 00000000
00000000 00000000 00000000 01000080 00200000 4000b000

14008028 02800000 00001008 00081008 10820000 00000000
01402000 00000001 00000020 10040e00 13000000 00000018
10000400 00000000 00800000 10000000 00000200 0004000b
20024000 00000200

>D04025
14040002 81000038 601a010a 10010e00 00081001 04000040
a6040484 08000420 00904b00 180a08d0 08180101 40098800
02800406 01a10000 81020023 400c000c 04008404 02080000
00404000 40060001 05020100 18000018 a1015000 04511110
00200000 82004001 04522110 d8001240 00420224 00060002
04144080 04008600

>D04024
0441a082 04e01808 20180108 0ad90600 04041080 00004010
0a008010 02a41a08 00026100 0a086840 01302034 70109800
c6800c26 0045900b 81200661 020c000c 04064000 12014c04
0c60020a 1022e002 c0640000 08069928 30804040 0cc81008
00802020 c000a802 06144010 c80000f1 0240c308 0046c000
42000040 020302e0

>D03899
00050040 6b000808 2000050a 40050700 88281051 4002c402
17103c40 09000a01 00200900 01080ae8 2c100000 4000db40
04008002 22a90000 01044021 2a28b008 08a00a00 02001100
13d8a008 40020201 00101520 b8020260 07084068 0a420414
00800c00 e0002201 00080800 64800024 02000383 00074802
c2e00004 02400400

>D03882
00010202 81000020 00000000 00480000 40402000 04000000
00200080 02000402 01000000 00100000 00100080 40001008
04028008 00000000 00000410 40090008 00000000 00000020
02212000 00020001 00000009 00000280 00008802 00000810
00200000 00000000 00404000 80000000 00000000 01000200
24020804 00000000

>D03829
00030000 01001400 00100000 00000c00 00180000 00000000
00000000 20002000 00000200 000001a2 00200000 40011000
1400901a 01800000 00000000 00180008 00000000 00000000
01c02000 00000201 00000000 101d2e10 01000080 00100018
00000003 00000000 40000000 10000000 00000010 01040011
801a0000 00800a00

>D03828
0003800a 01805800 00180100 48850610 00181484 00004000
00003000 22001800 06400900 800a08e0 28300004 4000b800
8e008822 02600800 00000400 000c000c 10000000 0200c000
00600400 40200201 40000100 1c168e60 23800840 0870001c
02042000 c0000000 68000020 90040000 00004108 02460003
e0044200 80800fa0

>D03826
00004026 42000208 60100108 00410e00 20001000 c0002012
02002004 00002809 04200a80 000808c0 08302004 40028000
00009222 20000002 01030141 00140008 00068000 82400020
08080018 00020241 40100100 18218c10 30020000 81402011
00151000 80010002 00040000 90001080 04008200 0006080b
00080080 30014620

>D03823
0000402e 42801a08 62100108 90010e00 20001020 c000a012
02002004 0200300d 04600a80 000808d0 08300004 50038000
00009ae2 20200000 010305c1 08160008 00068200 82480020
08080018 00020241 c0120100 18218c10 30024000 8140201d
00151000 c0010022 08040000 90001080 04008300 0006080b
a00c0080 30004600

>D03822
00000000 46000008 60000108 00010600 00001000 00000000
02000000 02001000 04000200 00080840 08200000 40008000
00008a02 20000000 01010001 00120008 00020000 02000000
00080000 00020200 00000000 08008c10 10000000 0044a018
00000000 80010022 00000000 80000000 00000300 00060003
80040000 1000060

>D03816
00010040 81040020 00000900 80010300 40080000 00000001
00000000 00000402 00004000 00100008 00000200 40009000
14028028 02800004 00000404 0008100c 00820000 00000020
03402080 0000a001 00000020 00001a00 0100a000 00000012
00200400 00000000 00406200 80000000 00000200 00000002
24020000 00100000

>D03807
0001c002 00808a00 80100100 02010700 001c0000 00006070
00000000 00001002 06100102 c00028e0 0d00000c 4001a800

0201981c 00e01000 003200c0 0c0cd00c 02800200 4200c800
00600000 00000000 c0000000 18008b38 23802041 85700006
00046000 c0000000 20120000 00040031 04004000 00468008
400c0008 0a420280

>D03806
00004004 0000c608 20102100 02010700 010c00c0 80000032
02008000 00001202 06100000 c00c68c0 0800800c 60019000
01009812 00e01000 40040000 000d200c 0a200208 02004804
00200000 00220240 00000000 1c018a18 01806801 83700010
04104000 c0000101 10128000 00040001 05000104 0006804a
00046008 08004600

>D03805
00024088 00809e00 02900100 12010600 20000020 00008010
01000000 22001006 00600980 000829f0 08300004 50039000
0d0088d2 00201000 40200480 02040000 02002210 02000921
00000018 00100201 80c20100 182e8640 20004000 a140001c
00001804 c0000101 88124000 90000001 04300102 0186801b
a0042008 08800600

>D03803
0003040a 10000800 0010010a 56010618 000c5020 00088010
00002080 0001f00b 00400044 004918c0 08008a04 d081a000
00008870 04401008 000220c0 060c448c 00001800 c28000c3
0030041c 40800000 40400800 58098ab8 22800022 86510016
03042002 8c000000 48100000 11400017 00000504 c0079002
20048200 20410620

>D03798
00000000 00202008 20080100 00010200 00040213 00400120
02000000 41000228 02084000 c0080840 08000020 40408000
40008402 00200080 00002080 10002008 00000020 02000042
00000000 20020000 00000000 0a200008 00020028 00580010
08000012 a0004000 00400090 4a900000 02002080 01020802
02000000 00000400

>D03788
0480801a 00884808 2100090a 00b10600 00001080 02110002
02000000 02001000 00000000 00100840 00202004 6000b000
02848c02 80000001 812b0441 000a2008 10022000 02000002
00400800 00026000 c0000000 08028e00 33040040 0840001c
00200080 c0000000 00800000 8000008c 00000700 20060001
a0044000 50010260

>D03787
00040012 21000200 10100102 02010610 00080260 00020010
02004000 00001001 00100900 000928c0 48118008 f0818800
00001000 00b01000 00020080 020c0004 21000200 02000180
82402020 40900001 80000106 18010010 21004000 81500010
00040000 c0000401 80000000 10000001 04300006 0047c00a
40080104 80030608

>D03784
00008002 0c80ca00 00000100 40050600 000c0040 00004000
04000000 01001000 00000100 000808c4 0900800c 40008800
02001804 00400000 800000c0 000c0004 00000008 82000004
00600000 00000000 c0000000 18008120 21800040 81400010
00006000 a0000000 00000040 40000000 04400080 0046000a
400c4000 000204a0

>D03781
00008002 0c80ca00 00000100 40050600 000c0040 00004000
04000000 01001400 01000100 000808c4 0900800c 40008800
02009804 00400000 800000c0 000c000c 00000008 82000004
00610010 00000000 c0000000 18008b20 21800040 81400010
00007000 a0001000 00400040 40000000 04400080 0046000a
440c4000 000204a0

>D03778
00000002 01800200 00101102 00810610 04080160 00000000
01002000 00001000 80500900 108818c0 28108008 50818800
80001800 00b00800 00030080 000c0004 00408000 06000000
00c82008 40010201 80100500 18008010 21000440 81400414
00040000 c0000208 00000000 10000080 84000006 0046040a
400c0004 20020400

>D03776
00010000 01805200 20120000 08100e40 00080080 82002010
80000000 20002000 84200200 020a48c2 00600000 600bb000
06019012 01040100 82080000 00180008 14001000 00000000
00404000 00420301 c0000000 18050e00 03800300 04100019
00002000 00000000 40004000 10000000 00000010 00060021
b0084000 03012220

>D03775
00010000 c1005200 20100102 0c110640 000800c0 02002010
80000000 20000000 84080800 020e48c0 08302000 400db000

06418016 11242103 80180000 400c0008 14001000 02000000
00400000 00006301 40000100 38040e00 03000301 06504018
00000000 80000000 00004000 10000000 08000000 0006002b
20004004 03412600
