# Supplementary material for: iEzy-Drug: A Web Server for Identifying the Interaction between Enzymes and Drugs in Cellular Networking
Source: Biomed Res Int. 2013 Nov 26;2013:701317. doi: 10.1155/2013/701317 (PMC3858977; doi:10.1155/2013/701317)
Supplement: Supplementary file 3 [file 701317.f3.pdf]

Online Supporting Information S3. The sequences for the enzymes listed in Online Supporting Information S1.

>hsa:10
MDIEAYFERIGYKNSRNKLDLETLTDILEHQIRAVPFENLNMHCGQAMELGLEAIFDHIV
RRNRGGWCLQVNQLLYWALTTIGFQTTMLGGYFYIPPVNKYSTGMVHLLLQVTIDGRNYI
VDAGSGSSSQMWQPLELISGKDQPQVPCIFCLTEERGIWYLDQIRREQYITNKEFLNSHL
LPKKKHQKIYLFTLEPRTIEDFESMNTYLQTSPTSSFITTSFCSLQTPEGVYCLVGFILT
YRKFNYKDNTDLVEFKTLTEEEVEEVLRNIFKISLGRNLVPKPGDGSLTI
>hsa:13
MGRKSLYLLIVGILIAYYIYTPLPDNVEEPWRMMWINAHLKTIQNLATFVELLGLHHFMD
SFKVVGSFDEVPPTSDENVTVTETKFNNILVRVYVPKRKSEALRRGLFYIHGGGWCVGSA
ALSGYDLLSRWTADRLDAVVVSTNYRLAPKYHFPIQFEDVYNALRWFLRKKVLAKYGVNP
ERIGISGDSAGGNLAAAVTQQLLDDPDVKIKLKIQSLIYPALQPLDVDLPSYQENSNFLF
LSKSLMVRFWSEYFTTDRSLEKAMLSRQHVPVESSHLFKFVNWSSLLPERFIKGHVYNNP
NYGSSELAKKYPGFLDVRAAPLLADDNKLRGLPLTYVITCQYDLLRDDGLMYVTRLRNTG
VQVTHNHVEDGFHGAFSFLGLKISHRLINQYIEWLKENL
>hsa:18
MASMLLAQRLACSFQHSYRLLVPGSRHISQAAAKVDVEFDYDGPLMKTEVPGPRSQELMK
QLNIIQNAEAVHFFCNYEESRGNYLVDVDGNRMLDLYSQISSVPIGYSHPALLKLIQQPQ
NASMFVNRPALGILPPENFVEKLRQSLLSVAPKGMSQLITMACGSCSNENALKTIFMWYR
SKERGQRGFSQEELETCMINQAPGCPDYSILSFMGAFHGRTMGCLATTHSKAIHKIDIPS
FDWPIAPFPRLKYPLEEFVKENQQEEARCLEEVEDLIVKYRKKKKTVAGIIVEPIQSEGG
DNHASDDFFRKLRDIARKHGCAFLVDEVQTGGGCTGKFWAHEHWGLDDPADVMTFSKKMM
TGGFFHKEEFRPNAPYRIFNTWLGDPSKNLLLAEVINIIKREDLLNNAAHAGKALLTGLL
DLQARYPQFISRVRGRGTFCSFDTPDDSIRNKLILIARNKGVVLGGCGDKSIRFRPTLVF
RDHHAHLFLNIFSDILADFK
>hsa:100
MAQTPAFDKPKVELHVHLDGSIKPETILYYGRRRGIALPANTAEGLLNVIGMDKPLTLPD
FLAKFDYYMPAIAGCREAIKRIAYEFVEMKAKEGVVYVEVRYSPHLLANSKVEPIPWNQA
EGDLTPDEVVALVGQGLQEGERDFGVKARSILCCMRHQPNWSPKVVELCKKYQQQTVVAI
DLAGDETIPGSSLLPGHVQAYQEAVKSGIHRTVHAGEVGSAEVVKEAVDILKTERLGHGY
HTLEDQALYNRLRQENMHFEICPWSSYLTGAWKPDTEHAVIRLKNDQANYSLNTDDPLIF
KSTLDTDYQMTKRDMGFTEEEFKRLNINAAKSSFLPEDEKRELLDLLYKAYGMPPSASAG
QNL
>hsa:10056
MPTVSVKRDLLFQALGRTYTDEEFDELCFEFGLELDEITSEKEIISKEQGNVKAAGASDV
VLYKIDVPANRYDLLCLEGLVRGLQVFKERIKAPVYKRVMPDGKIQKLIITEETAKIRPF
AVAAVLRNIKFTKDRYDSFIELQEKLHQNICRKRALVAIGTHDLDTLSGPFTYTAKRPSD
IKFKPLNKTKEYTACELMNIYKTDNHLKHYLHIIENKPLYPVIYDSNGVVLSMPPIINGD
HSRITVNTRNIFIECTGTDFTKAKIVLDIIVTMFSEYCENQFTVEAAEVVFPNGKSHTFP
ELAYRKEMVRADLINKKVGIRETPENLAKLLTRMYLKSEVIGDGNQIEIEIPPTRADIIH
ACDIVEDAAIAYGYNNIQMTLPKTYTIANQFPLNKLTELLRHDMAAAGFTEALTFALCSQ
EDIADKLGVDISATKAVHISNPKTAEFQVARTTLLPGLLKTIAANRKMPLPLKLFEISDI

VIKDSNTDVGAKNYRHLCAVYYNKNPGFEIIHGLLDRIMQLLDVPPGEDKGGYVIKASEG
PAFFPGRCAEIFARGQSVGKLGVLHPDVITKFELTMPCSSLEINVGPFL
>hsa:10188
MLEARPPRTQGSDAAGAAAGRGLRALLLSLTAAAGIWGSMGERSAYQRLAGGEEGPQRLG
GGRMQPEEGTGWLLELLSEVQLQQYFLRLRDDLNVTRLSHFEYVKNEDLEKIGMGRPGQR
RLWEAVKRRKALCKRKSWMSKVFSGKRLEAEFPPHHSQSTFRKTSPAPGGPAGEGPLQSL
TCLIGEKDLRLLEKLGDGSFGVVRRGEWDAPSGKTVSVAVKCLKPDVLSQPEAMDDFIRE
VNAMHSLDHRNLIRLYGVVLTPPMKMVTELAPLGSLLDRLRKHQGHFLLGTLSRYAVQVA
EGMGYLESKRFIHRDLAARNLLLATRDLVKIGDFGLMRALPQNDDHYVMQEHRKVPFAWC
APESLKTRTFSHASDTWMFGVTLWEMFTYGQEPWIGLNGSQILHKIDKEGERLPRPEDCP
QDIYNVMVQCWAHKPEDRPTFVALRDFLLEAQPTDMRALQDFEEPDKLHIQMNDVITVIE
GRAENYWWRGQNTRTLCVGPFPRNVVTSVAGLSAQDISQPLQNSFIHTGHGDSDPRHCWG
FPDRIDELYLGNPMDPPDLLSVELSTSRPPQHLGGVKREPPPRPPQPAFFTQKPTYDPVS
EDQDPLSSDFKRLGLRKPGLPRGLWLAKPSARVPGTKASRGSGAEVTLIDFGEEPVVPAL
RPCAPSLAQLAMDACSLLDETPPQSPTRALPRPLHPTPVVDWDARPLPPPPAYDDVAQDE
DDFEICSINSTLVGAGVPAGPSQGQTNYAFVPEQARPPPPLEDNLFLPPQGGGKPPSSAQ
TAEIFQALQQECMRQLQAPAGSPAPSPSPGGDDKPQVPPRVPIPPRPTRPHVQLSPAPPG
EEETSQWPGPASPPRVPPREPLSPQGSRTPSPLVPPGSSPLPPRLSSSPGKTMPTTQSFA
SDPKYATPQVIQAPGPRAGPCILPIVRDGKKVSSTHYYLLPERPSYLERYQRFLREAQSP
EEPTPLPVPLLLPPPSTPAPAAPTATVRPMPQAALDPKANFSTNNSNPGARPPPPRATAR
LPQRGCPGDGPEAGRPADKIQMVEQLFGLGLRPRGECHKVLEMFDWNLEQAGCHLLGSWG
PAHHKR
>hsa:10269
MGMWASLDALWEMPAEKRIFGAVLLFSWTVYLWETFLAQRQRRIYKTTTHVPPELGQIMD
SETFEKSRLYQLDKSTFSFWSGLYSETEGTLILLFGGIPYLWRLSGRFCGYAGFGPEYEI
TQSLVFLLLATLFSALTGLPWSLYNTFVIEEKHGFNQQTLGFFMKDAIKKFVVTQCILLP
VSSLLLYIIKIGGDYFFIYAWLFTLVVSLVLVTIYADYIAPLFDKFTPLPEGKLKEEIEV
MAKSIDFPLTKVYVVEGSKRSSHSNAYFYGFFKNKRIVLFDTLLEEYSVLNKDIQEDSGM
EPRNEEEGNSEEIKAKVKNKKQGCKNEEVLAVLGHELGHWKLGHTVKNIIISQMNSFLCF
FLFAVLIGRKELFAAFGFYDSQPTLIGLLIIFQFIFSPYNEVLSFCLTVLSRRFEFQADA
FAKKLGKAKDLYSALIKLNKDNLGFPVSDWLFSMWHYSHPPLLERLQALKTMKQH
>hsa:10279
MAVWLAQWLGPLLLVSLWGLLAPASLLRRLGEHIQQFQESSAQGLGLSLGPGAAALPKVG
WLEQLLDPFNVSDRRSFLQRYWVNDQHWVGQDGPIFLHLGGEGSLGPGSVMRGHPAALAP
AWGALVISLEHRFYGLSIPAGGLEMAQLRFLSSRLALADVVSARLALSRLFNISSSSSPWI
CFGGSYAGSLAAWARLKFPHLIFASVASSAPVRAVLDFSEYNDVVSRSLMSTAIGGSLEC
RAAVSVAFAEVERRLRSGGAAQAALRTELSACGPLGRAENQAELLGALQALVGGVVQYDG
QTGAPLSVRQLCGLLLGGGGNRSHSTPYCGLRRAVQIVLHSLGQKCLSFSRAETVAQLRS
TEPQLSGVGDRQWLYQTCTEFGFYVTCENPRCPFSQLPALPSQLDLCEQVFGLSALSVAQ
AVAQTNSYYGGQTPGANKVLFVNGDTDPWHVLSVTQALGSSESTLLIRTGSHCLDMAPER
PSDSPSLRLGRQNIFQQLQTWLKLAKESQIKGEV
>hsa:10295
MILASVLRSGPGGGLPLRPLLGPALALRARSTSATDTHHVEMARERSKTVTSFYNQSAID
AAAEKPSVRLTPTMMLYAGRSQDGSHLLKSARYLQQELPVRIAHRIKGFRCLPFIIGCNP

TILHVHELYIRAFQKLTDFPPIKDQADEAQYCQLVRQLLDDHKDVVTLLAEGLRESRKHI
EDEKLVRYFLDKTLTSRLGIRMLATHHLALHEDKPDFVGIICTRLSPKKIIEKWVDFARR
LCEHKYGNAPRVRINGHVAARFPFIPMPLDYILPELLKNAMRATMESHLDTPYNVPDVVI
TIANNDVDLIIRISDRGGGIAHKDLDRVMDYHFTTAEASTQDPRISPLFGHLDMHSGAQS
GPMHG
>hsa:10327
MAASCVLLHTGQKMPLIGLGTWKSEPGQVKAAVKYALSVGYRHIDCAAIYGNEPEIGEAL
KEDVGPGKAVPREELFVTSKLWNTKHHPEDVEPALRKTLADLQLEYLDLYLMHWPYAFER
GDNPFPKNADGTICYDSTHYKETWKALEALVAKGLVQALGLSNFNSRQIDDILSVASVRP
AVLQVECHPYLAQNELIAHCQARGLEVTAYSPLGSSDRAWRDPDEPVLLEEPVVLALAEK
YGRSPAQILLRWQVQRKVICIPKSITPSRILQNIKVFDFTFSPEEMKQLNALNKNWRYIV
PMLTVDGKRVPRDAGHPLYPFNDPY
>hsa:1033
MKPPSSIQTSCKFKDVRRNVQKDTEELKSCGIQDIFVFCTRGELSKYRVPNLLDLYQQCG
IITHHHPIADGGTPDIASCCEIMEELTTCLKNYRKTLIHCYGGLGRSCLVAACLLLYLSD
TISPEQAIDSLRDLRGSGAIQTIKQYNYLHEFRDKLAAHLSSRDSQSRSVSR
>hsa:10461
MGPAPLPLLLGLFLPALWRRAITEAREEAKPYPLFPGPFPGSLQTDHTPLLSLPHASGYQ
PALMFSPTQPGRPHTGNVAIPQVTSVESKPLPPLAFKHTVGHIILSEHKGVKFNCSISVP
NIYQDTTISWWKDGKELLGAHHAITQFYPDDEVTAIIASFSITSVQRSDNGSYICKMKIN
NEEIVSDPIYIEVQGLPHFTKQPESMNVTRNTAFNLTCQAVGPPEPVNIFWVQNSSRVNE
QPEKSPSVLTVPGLTEMAVFSCEAHNDKGLTVSKGVQINIKAIPSPPTEVSIRNSTAHSI
LISWVPGFDGYSPFRNCSIQVKEADPLSNGSVMIFNTSALPHLYQIKQLQALANYSIGVS
CMNEIGWSAVSPWILASTTEGAPSVAPLNVTVFLNESSDNVDIRWMKPPTKQQDGELVGY
RISHVWQSAGISKELLEEVGQNGSRARISVQVHNATCTVRIAAVTRGGVGPFSDPVKIFI
PAHGWVDYAPSSTPAPGNADPVLIIFGCFCGFILIGLILYISLAIRKRVQETKFGNAFTE
EDSELVVNYIAKKSFCRRAIELTLHSLGVSEELQNKLEDVVIDRNLLILGKILGEGEFGS
VMEGNLKQEDGTSLKVAVKTMKLDNSSQREIEEFLSEAACMKDFSHPNVIRLLGVCIEMS
SQGIPKPMVILPFMKYGDLHTYLLYSRLETGPKHIPLQTLLKFMVDIALGMEYLSNRNFL
HRDLAARNCMLRDDMTVCVADFGLSKKIYSGDYYRQGRIAKMPVKWIAIESLADRVYTSK
SDVWAFGVTMWEIATRGMTPYPGVQNHEMYDYLLHGHRLKQPEDCLDELYEIMYSCWRTD
PLDRPTFSVLRLQLEKLLESLPDVRNQADVIYVNTQLLESSEGLAQGSTLAPLDLNIDPD
SIIASCTPRAAISVVTAEVHDSKPHEGRYILNGGSEEWEDLTSAPSAAVTAEKNSVLPGE
RLVRNGVSWSHSSMLPLGSSLPDELLFADDSSEGSEVLM
>hsa:10549
MEALPLLAATTPDHGRHRRLLLLPLLLFLLPAGAVQGWETEERPRTREEECHFYAGGQVY
PGEASRVSVADHSLHLSKAKISKPAPYWEGTAVIDGEFKELKLTDYRGKYLVFFFYPLDF
TFVCPTEIIAFGDRLEEFRSINTEVVACSVDSQFTHLAWINTPRRQGGLGPIRIPLLSDL
THQISKDYGVYLEDSGHTLRGLFIIDDKGILRQITLNDLPVGRSVDETLRLVQAFQYTDK
HGEVCPAGWKPGSETIIPDPAGKLKYFDKLN
>hsa:1056
MLTMGRLQLVVLGLTCCWAVASAAKLGAVYTEGGFVEGVNKKLGLLGDSVDIFKGIPFAA
PTKALENPQPHPGWQGTLKAKNFKKRCLQATITQDSTYGDEDCLYLNIWVPQGRKQVSRD
LPVMIWIYGGAFLMGSGHGANFLNNYLYDGEEIATRGNVIVVTFNYRVGPLGFLSTGDAN

LPGNYGLRDQHMAIAWVKRNIAAFGGDPNNITLFGESAGGASVSLQTLSPYNKGLIRRAI
SQSGVALSPWVIQKNPLFWAKKVAEKVGCPVGDAARMAQCLKVTDPRALTLAYKVPLAGL
EYPMLHYVGFVPVIDGDFIPADPINLYANAADIDYIAGTNNMDGHIFASIDMPAINKGNK
KVTEEDFYKLVSEFTITKGLRGAKTTFDVYTESWAQDPSQENKKKTVVDFETDVLFLVPT
EIALAQHRANAKSAKTYAYLFSHPSRMPVYPKWVGADHADDIQYVFGKPFATPTGYRPQD
RTVSKAMIAYWTNFAKTGDPNMGDSAVPTHWEPYTTENSGYLEITKKMGSSSMKRSLRTN
FLRYWTLTYLALPTVTDQEATPVPPTGDSEATPVPPTGDSETAPVPPTGDSGAPPVPPTG
DSGAPPVPPTGDSGAPPVPPTGDSGAPPVPPTGDSGAPPVPPTGDSGAPPVPPTGDSGAP
PVPPTGDSGAPPVPPTGDAGPPPVPPTGDSGAPPVPPTGDSGAPPVTPTGDSETAPVPPT
GDSGAPPVPPTGDSEAAPVPPTDDSKEAQMPAVIRF

>hsa:10667

MVGSALRRGAHAYVYLVSKASHISRGHQHQAWGSRPPAAECATQRAPGSVVELLGKSYPQ
DDHSNLTRKVLTRVGRNLHNQQHHPLWLIKERVKEHFYKQYVGRFGTPLFSVYDNLSPVV
TTWQNFDSLLIPADHPSRKKGDNYYLNRTHMLRAHTSAHQWDLLHAGLDAFLVVGDVYRR
DQIDSQHYPIFHQLEAVRLFSKHELFAGIKDGESLQLFEQSSRSAHKQETHTMEAVKLVE
FDLKQTLTRLMAHLFGDELEIRWVDCYFPFTHPSFEMEINFHGEWLEVLGCGVMEQQLVN
SAGAQDRIGWAFGLGLERLAMILYDIPDIRLFWCEDERFLKQFCVSNINQKVKFQPLSKY
PAVINDISFWLPSENYAENDFYDLVRTIGGDLVEKVDLIDKFVHPKTHKTSHCYRITYRH
MERTLSQREVRHIHQALQEAAVQLLGVEGRF

>hsa:107

MAGAPRGGGGGGGGAGEPGGAERAAGTSRRRGLRACDEEFACPELEALFRGYTLRLEQAA
TLKALAVLSLLAGALALAELLGAPGPAPGLAKGSHPVHCVLFLALLVVTNVRSLQVPQLQ
QVGQLALLFSLTFALLCCPFALGGPARGSAGAAGGPATAEQGVWQLLLVTFVSYALLPVR
SLLAIGFGLVVAASHLLVTATLVPAKRPRLWRTLGANALLFVGVNMYGVFVRILTERSQR
KAFLQARSCIEDRLRLEDENEKQERLLMSLLPRNVAMEMKEDFLKPPERIFHKIYIQRHD
NVSILFADIVGFTGLASQCTAQELVKLLNELFGKFDELATENHCRRIKILGDCYYCVSGL
TQPKTDHAHCCVEMGLDMIDTITSVAEATEVDLNMRVGLHTGRVLCGVLGLRKWQYDVWS
NDVTLANVMEAAGLPGKVHITKTTLACLNGDYEVEPGYGHERNSFLKTHNIETFFIVPSH
RRKIFPGLILSDIKPAKRMKFKTVCYLLVQLMHCRKMFKAEIPFSNVMTCEDDDKRRALR
TASEKLRNRSSFSTNVVYTTPGTRVNRYISRLLEARQTELEMADLNFFTLKYKHVEREQK
YHQLQDEYFTSAVVLTLILAALFGLVYLLIFPQSVVVLLLLVFCICFLVACVLYLHITRV
QCFPGCLTIQIRTVLCIFIVVLIYSVAQGCVVGCLPWAWSSKPNSSLVVLSSGGQRTALP
TLPCESTHHALLCCLVGTLPLAIFFRVSSLPKMILLSGLTTSYILVLELSGYTRTGGGAV
SGRSYEPIVAILLFSCALALHARQVDIRLRLDYLWAAQAEEEREDMEKVKLDNRRILFNL
LPAHVAQHFLMSNPRNMDLYYQSYSQVGVMFASIPNFNDFYIELDGNNMGVECLRLLNEI
IADFDELMEKDFYKDIEKIKTIGSTYMAAVGLAPTSGTKAKKSISSHLSTLADFAIEMFD
VLDEINYQSYNDFVLRVGINVGPVVAGVIGARRPQYDIWGNTVNVASRMDSTGVQGRIQV
TEEVHRLLRRCPYHFVCRGKVSVKGKGEMLTYFLEGRTDGNGSQIRSLGLDRKMCPFGRA
GLQGRRPPVCPMPGVSVRAGLPPHSPGQYLPSAAAGKEA

>hsa:10720

MTLKWTSVLLLIHLSCYFSSGSCGKVLVWAAEYSHWMNMKTILKELVQRGHEVTVLASSA
SILFDPNDASTLKFEVYPTSLTKTEFENIIMQQVKRWSDIRKDSFWLYFSQEQEILWELY
DIFRNFCKDVVSNKKVMKKLQESRFDIVFADAVFPCGELLAALLNIRFVYSLRFTPGYTI
ERHSGGLIFPPSYIPIVMSKLSDQMTFMERVKNMIYVLYFDFWFQMSDMKKWDQFYSEVL

GRPTTLFETMGKADIWLMRNSWSFQFPHPFLPNVDFVGGFHCKPAKPLPKEMEEFVQSSG
ENGVVVFSLGSVISNMTAERANVIATALAKIPQKVLWRFDGNKPDALGLNTRLYKWIPQN
DLLGHPKTRAFITHGGANGIYEAIYHGIPMVGIPLFFDQPDNIAHMKAKGAAVRLDFNTM
SSTDLLNALKTVINDPLYKENIMKLSRIQHDQPVKPLDRAVFWIEFVMPHKGAKHLRVAA
HDLTWFQYHSLDVIGFLLACVATVIFIITKFCLFCFWKFARKGKKGKRD
>hsa:10747
MRLLTLLGLLCGSVATPLGPKWPEPVFGRLASPGFPGEYANDQERRWTLTAPPGYRLRLY
FTHFDLELSHLCEYDFVKLSSGAKVLATLCGQESTDTERAPGKDTFYSLGSSLDITFRSD
YSNEKPFTGFEAFYAAEDIDECQVAPGEAPTCDHHCHNHLGGFYCSCRAGYVLHRNKRTC
SALCSGQVFTQRSGELSSPEYPRPYPKLSSCTYSISLEEGFSVILDFVESFDVETHPETL
CPYDFLKIQTDREEHGPFCGKTLPHRIETKSNTVTITFVTDESGDHTGWKIHYTSTAQPC
PYPMAPPNGHVSPVQAKYILKDSFSIFCETGYELLQGHLPLKSFTAVCQKDGSWDRPMPA
CSIVDCGPPDDLPSGRVEYITGPGVTTYKAVIQYSCEETFYTMKVNDGKYVCEADGFWTS
SKGEKSLPVCEPVCGLSARTTGGRIYGGQKAKPGDFPWQVLILGGTTAAGALLYDNWVLT
AAHAVYEQKHDASALDIRMGTLKRLSPHYTQAWSEAVFIHEGYTHDAGFDNDIALIKLNN
KVVINSNITPICLPRKEAESFMRTDDIGTASGWGLTQRGFLARNLMYVDIPIVDHQKCTA
AYEKPPYPRGSVTANMLCAGLESGGKDSCRGDSGGALVFLDSETERWFVGGIVSWGSMNC
GEAGQYGVYTKVINYIPWIENIISDF
>hsa:108
MWQEAMRRRRYLRDRSEEAAGGGDGLPRSRDWLYESYYCMSQQHPLIVFLLLIVMGSCLA
LLAVFFALGLEVEDHVAFLITVPTALAIFFAIFILVCIESVFKKLLRLFSLVIWICLVAM
GYLFMCFGGTVSPWDQVSFFLFIIFVVYTMLPFNMRDAIIASVLTSSSHTIVLSVCLSAT
PGGKEHLVWQILANVIIFICGNLAGAYHKHLMELALQQTYQDTCNCIKSRIKLEFEKRQQ
ERLLLSLLPAHIAMEMKAEIIQRLQGPKAGQMENTNNFHNLYVKRHTNVSILYADIVGFT
RLASDCSPGELVHMLNELFGKFDQIAKENECMRIKILGDCYYCVSGLPISLPNHAKNCVK
MGLDMCEAIKKVRDATGVDINMRVGVHSGNVLCGVIGLQKWQYDVWSHDVTLANHMEAGG
VPGRVHISSVTLEHLNGAYKVEEGDGDIRDPYLKQHLVKTYFVINPKGERRSPQHLFRPR
HTLDGAKMRASVRMTRYLESWGAAKPFAHLHHRDSMTTENGKISTTDVPMGQHNFQNRTL
RTKSQKKRFEEELNERMIQAIDGINAQKQWLKSEDIQRISLLFYNKVLEKEYRATALPAF
KYYVTCACLIFFCIFIVQILVLPKTSVLGISFGAAFLLLAFILFVCFAGQLLQCSKKASP
LLMWLLKSSGIIANRPWPRISLTIITTAIILMMAVFNMFFLSDSEETIPPTANTTNTSFS
ASNNQVAILRAQNLFFLPYFIYSCILGLISCSVFLRVNYELKMLIMMVALVGYNTILLHT
HAHVLGDYSQVLFERPGIWKDLKTMGSVSLSIFFITLLVLGRQNEYYCRLDFLWKNKFKK
EREEIETMENLNRVLLENVLPAHVAEHFLARSLKNEELYHQSYDCVCVMFASIPDFKEFY
TESDVNKEGLECLRLLNEIIADFDDLLSKPKFSGVEKIKTIGSTYMAATGLSAVPSQEHS
QEPERQYMHIGTMVEFAFALVGKLDAINKHSFNDFKLRVGINHGPVIAGVIGAQKPQYDI
WGNTVNVASRMDSTGVLDKIQVTEETSLVLQTLGYTCTCRGIINVKGKGDLKTYFVNTEM
SRSLSQSNVAS
>hsa:10825
MRPADLPPRPMEESPASSSAPTETEEPGSSAEVMEEVTTCSFNSPLFRQEDDRGITYRIP
ALLYIPPTHTFLAFAEKRSTRRDEDALHLVLRRGLRIGQLVQWGPLKPLMEATLPGHRTM
NPCPVWEQKSGCVFLFFICVRGHVTERQQIVSGRNAARLCFIYSQDAGCSWSEVRDLTEE
VIGSELKHWATFAVGPGHGIQLQSGRLVIPAYTYYIPSWFFCFQLPCKTRPHSLMIYSDD
LGVTWHHGRLIRPMVTVECEVAEVTGRAGHPVLYCSARTPNRCRAEALSTDHGEGFQRLA

LSRQLCEPPHGCQGSVVSFRPLEIPHRCQDSSSKDAPTIQQSSPGSSLRLEEEAGTPSES
WLLYSHPTSRKQRVDLGIYLNQTPLEAACWSRPWILHCGPCGYSDLAALEEEGLFGCLFE
CGTKQECEQIAFRLFTHREILSHLQGDCTSPGRNPSQFKSN
>hsa:10846
MEDGPSNNASCFRRLTECFLSPSLTDEKVKAYLSLHPQVLDEFVSESVSAETVEKWLKRK
NNKSEDESAPKEVSRYQDTNMQGVVYELNSYIEQRLDTGGDNQLLLYELSSIIKIATKAD
GFALYFLGECNNSLCIFTPPGIKEGKPRLIPAGPITQGTTVSAYVAKSRKTLLVEDILGD
ERFPRGTGLESGTRIQSVLCLPIVTAIGDLIGILELYRHWGKEAFCLSHQEVATANLAWA
SVAIHQVQVCRGLAKQTELNDFLLDVSKTYFDNIVAIDSLLEHIMIYAKNLVNADRCALF
QVDHKNKELYSDLFDIGEEKEGKPVFKKTKEIRFSIEKGIAGQVARTGEVLNIPDAYADP
RFNREVDLYTGYTTRNILCMPIVSRGSVIGVVQMVNKISGSAFSKTDENNFKMFAVFCAL
ALHCANMYHRIRHSECIYRVTMEKLSYHSICTSEEWQGLMQFTLPVRLCKEIELFHFDIG
PFENMWPGIFVYMVHRSCGTSCFELEKLCRFIMSVKKNYRRVPYHNWKHAVTVAHCMYAI
LQNNHTLFTDLERKGLLIACLCHDLDHRGFSNSYLQKFDHPLAALYSTSTMEQHHFSQTV
SILQLEGHNIFSTLSSSEYEQVLEIIRKAIIATDLALYFGNRKQLEEMYQTGSLNLNNQS
HRDRVIGLMMTACDLCSVTKLWPVTKLTANDIYAEFWAEGDEMKKLGIQPIPMMDRDKKD
EVPQGQLGFYNAVAIPCYTTLTQILPPTEPLLKACRDNLSQWEKVIRGEETATWISSPSV
AQKAAASED
>hsa:10858
MSPGLLLLGSAVLLAFGLCCTFVHRARSRYEHIPGPPRPSFLLGHLPCFWKKDEVGGRVL
QDVFLDWAKKYGPVVRVNVFHKTSVIVTSPESVKKFLMSTKYNKDSKMYRALQTVFGERL
FGQGLVSECNYERWHKQRRVIDLAFSRSSLVSLMETFNEKAEQLVEILEAKADGQTPVSM
QDMLTYTAMDILAKAAFGMETSMLLGAQKPLSQAVKLMLEGITASRNTLAKFLPGKRKQL
REVRESIRFLRQVGRDWVQRRREALKRGEEVPADILTQILKAEEGAQDDEGLLDNFVTFF
IAGHETSANHLAFTVMELSRQPEIVARLQAEVDEVIGSKRYLDFEDLGRLQYLSQVLKES
LRLYPPAWGTFRLLEEETLIDGVRVPGNTPLLFSTYVMGRMDTYFEDPLTFNPDRFGPGA
PKPRFTYFPFSLGHRSCIGQQFAQMEVKVVMAKLLQRLEFRLVPGQRFGLQEQATLKPLD
PVLCTLRPRGWQPAPPPPPC
>hsa:109
MPRNQGFSEPEYSAEYSAEYSVSLPSDPDRGVGRTHEISVRNSGSCLCLPRFMRLTFVPE
SLENLYQTYFKRQRHETLLVLVVFAALFDCYVVVMCAVVFSSDKLASLAVAGIGLVLDII
LFVLCKKGLLPDRVTRRVLPYVLWLLITAQIFSYLGLNFARAHAASDTVGWQVFFVFSFF
ITLPLSLSPIVIISVVSCVVHTLVLGVTVAQQQQEELKGMQLLREILANVFLYLCAIAVG
IMSYYMADRKHRKAFLEARQSLEVKMNLEEQSQQQENLMLSILPKHVADEMLKDMKKDES
QKDQQQFNTMYMYRHENVSILFADIVGFTQLSSACSAQELVKLLNELFARFDKLAAKYHQ
LRIKILGDCYYCICGLPDYREDHAVCSILMGLAMVEAISYVREKTKTGVDMRVGVHTGTV
LGGVLGQKRWQYDVWSTDVTVANKMEAGGIPGRVHISQSTMDCLKGEFDVEPGDGGSRCD
YLEEKGIETYLIIASKPEVKKTATQNGLNGSALPNGAPASSKSSSPALIETKEPNGSAHS
SGSTSEKPEEQDAQADNPSFPNPRRRLRLQDLADRVVDASEDEHELNQLLNEALLERESA
QVVKKRNTFLLSMRFMDPEMETRYSVEKEKQSGAAFSCSCVVLLCTALVEILIDPWLMTN
YVTFMVGEILLLILTICSLAAIFPRAFPKKLVAFSTWIDRTRWARNTWAMLAIFILVMAN
VVDMLSCLQYYTGPSNATAGMETEGSCLENPKYYNYVAVLSLIATIMLVQVSHMVKLTLM
LLVAGAVATINLYAWRPVFDEYDHKRFREHDLPMVALEQMQGFNPGLNGTDRLPLVPSKY
SMTVMVFLMMLSFYYFSRHVEKLARTLFLWKIEVHDQKERVYEMRRWNEALVTNMLPEHV

ARHFLGSKKRDEELYSQTYDEIGVMFASLPNFADFYTEESINNGGIECLRFLNEIISDFD
SLLDNPKFRVITKIKTIGSTYMAASGVTPDVNTNGFASSNKEDKSERERWQHLADLADFA
LAMKDTLTNINNQSFNNFMLRIGMNKGGVLAGVIGARKPHYDIWGNTVNVASRMESTGVM
GNIQVVEETQVILREYGFRFVRRGPIFVKGKGELLTFFLKGRDKLATFPNGPSVTLPHQV
VDNS
>hsa:10901
MHKAGLLGLCARAWNSVRMASSGMTRRDPLANKVALVTASTDGIGFAIARRLAQDGAHVV
VSSRKQQNVDQAVATLQGEGLSVTGTVCHVGKAEDRERLVATAVKLHGGIDILVSNAAVN
PFFGSIMDVTEEVWDKTLDINVKAPALMTKAVVPEMEKRGGGSVVIVSSIAAFSPSPGFS
PYNVSKTALLGLTKTLAIELAPRNIRVNCLAPGLIKTSFSRMLWMDKEKEESMKETLRIR
RLGEPEDCAGIVSFLCSEDASYITGETVVVGGGTPSRL
>hsa:10924
MALVRALVCCLLTAWHCRSGLGLPVAPAGGRNPPPAIGQFWHVTDLHLDPTYHITDDHTK
VCASSKGANASNPGPFGDVLCDSPYQLILSAFDFIKNSGQEASFMIWTGDSPPHVPVPEL
STDTVINVITNMTTTIQSLFPNLQVFPALGNHDYWPQDQLPVVTSKVYNAVANLWKPWLD
EEAISTLRKGGFYSQKVTTNPNLRIISLNTNLYYGPNIMTLNKTDPANQFEWLESTLNNS
QQNKEKVYIIAHVPVGYLPSSQNITAMREYYNEKLIDIFQKYSDVIAGQFYGHTHRDSIM
VLSDKKGSPVNSLFVAPAVTPVKSVLEKQTNNPGIRLFQYDPRDYKLLDMLQYYLNLTEA
NLKGESIWKLEYILTQTYDIEDLQPESLYGLAKQFTILDSKQFIKYYNYFFVSYDSSVTC
DKTCKAFQICAIMNLDNISYADCLKQLYIKHNY
>hsa:10935
MAAAVGRLLRASVARHVSAIPWGISATAALRPAACGRTSLTNLLCSGSSQAKLFSTSSSC
HAPAVTQHAPYFKGTAVVNGEFKDLSLDDFKGKYLVLFFYPLDFTFVCPTEIVAFSDKAN
EFHDVNCEVVAVSVDSHFSHLAWINTPRKNGGLGHMNIALLSDLTKQISRDYGVLLEGSG
LALRGLFIIDPNGVIKHLSVNDLPVGRSVEETLRLVKAFQYVETHGEVCPANWTPDSPTI
KPSPAASKEYFQKVNQ
>hsa:10941
MLNNLLLFSLQISLIGTTLGGNVLIWPMEGSHWLNVKIIIDELIKKEHNVTVLVASGALF
ITPTSNPSLTFEIYRVPFGKERIEGVIKDFVLTWLENRPSPSTIWRFYQEMAKVIKDFHM
VSQEICDGVLKNQQLMAKLKKSKFEVLVSDPVFPCGDIVALKLGIPFMYSLRFSPASTVE
KHCGKVPYPPSYVPAVLSELTDQMSFTDRIRNFISYHLQDYMFETLWKSWDSYYSKALDG
SHWLNIKIILEELIQRNHNVTVLASSATLFINSNPDSPVNFEVIPVSYKKSNIDSLIEHM
IMLWIDHRPTPLTIWAFYKELGKLLDTFFQINIQLCDGVLKNPKLMARLQKGGFDVLVAD
PVTICGDLVALKLGIPFMYTLRFSPASTVERHCGKIPAPVSYVPAALSELTDQMTFGERI
KNTISYSLQDYIFQSYWGEWNSYYSKILGRPTTLCETMGKAEIWLIRTYWDFEFPRPYLP
NFEFVGGLHCKPAKPLPKVLWRYKGKKPATLGNNTQLFDWIPQNDLLGHPKTKAFITHGG
TNGIYEAIYHGVPMVGVPMFADQPDNIAHMKAKGAAVEVNLNTMTSVDLLSALRTVINEP
SYKENAMRLSRIHHDQPVKPLDRAVFWIEFVMRHKGAKHLRVAAHDLTWFQYHSLDVIGF
LLVCVTTAIFLVIQCCLFSCQKFGKIGKKKKRE
>hsa:11072
MSSRGHSTLPRTLMAPRMISEGDIGGIAQITSSLFLGRGSVASNRHLLQARGITCIVNAT
IEIPNFNWPQFEYVKVPLADMPHAPIGLYFDTVADKIHSVSRKHGATLVHCAAGVSRSAT
LCIAYLMKFHNVCLLEAYNWVKARRPVIRPNVGFWRQLIDYERQLFGKSTVKMVQTPYGI
VPDVYEKESRHLMPYWGI

>hsa:111
MKSQKEGCCSRGDLSIQTGPGGEWAPRRLVSNVLIFSCTNIVGVCTHYPAEVSQRQAFQE
TRECIQARLHSQRENQQQERLLLSVLPRHVAMEMKADINAKQEDMMFHKIYIQKHDNVSI
LFADIEGFTSLASQCTAQELVMTLNELFARFDKLAAENHCLRIKILGDCYYCVSGLPEAR
ADHAHCCVEMGMDMIEAISLVREVTGVNVNMRVGIHSGRVHCGVLGLRKWQFDVWSNDVT
LANHMEAGGKAGRIHITKATLNYLNGDYEVEPGCGGERNAYLKEHSIETFLILRCTQKRK
EEKAMIAKMNRQRTNSIGHNPPHWGAERPFYNHLGGNQVSKEMKRMGFEDPKDKNAQESA
NPEDEVDEFLGRAIDARSIDRLRSEHVRKFLLTFREPDLEKKYSKQVDDRFGAYVACASL
VFLFICFVQITIVPHSIFMLSFYLTCSLLLTLVVFVSVIYSCVKLFPSPLQTLSRKIVRS
KMNSTLVGVFTITLVFLAAFVNMFTCNSRDLLGCLAQEHNISASQVNACHVAESAVNYSL
GDEQGFCGSPWPNCNFPEYFTYSVLLSLLACSVFLQISCIGKLVLMLAIELIYVLIVEVP
GVTLFDNADLLVTANAIDFFNNGTSQCPEHATKVALKVVTPIIISVFVLALYLHAQQVES
TARLDFLWKLQATEEKEEMEELQAYNRRLLHNILPKDVAAHFLARERRNDELYYQSCECV
AVMFASIANFSEFYVELEANNEGVECLRLLNEIIADFDEIISEDRFRQLEKIKTIGSTYM
AASGLNDSTYDKVGKTHIKALADFAMKLMDQMKYINEHSFNNFQMKIGLNIGPVVAGVIG
ARKPQYDIWGNTVNVASRMDSTGVPDRIQVTTDMYQVLAANTYQLECRGVVKVKGKGEMM
TYFLNGGPPLS
>hsa:112
MSWFSGLLVPKVDERKTAWGERNGQKRSRRRGTRAGGFCTPRYMSCLRDAEPPSPTPAGP
PRCPWQDDAFIRRGGPGKGKELGLRAVALGFEDTEVTTTAGGTAEVAPDAVPRSGRSCWR
RLVQVFQSKQFRSAKLERLYQRYFFQMNQSSLTLLMAVLVLLTAVLLAFHAAPARPQPAY
VALLACAAALFVGLMVVCNRHSFRQDSMWVVSYVVLGILAAVQVGGALAADPRSPSAGLW
CPVFFVYIAYTLLPIRMRAAVLSGLGLSTLHLILAWQLNRGDAFLWKQLGANVLLFLCTN
VIGICTHYPAEVSQRQAFQETRGYIQARLHLQHENRQQERLLLSVLPQHVAMEMKEDINT
KKEDMMFHKIYIQKHDNVSILFADIEGFTSLASQCTAQELVMTLNELFARFDKLAAENHC
LRIKILGDCYYCVSGLPEARADHAHCCVEMGVDMIEAISLVREVTGVNVNMRVGIHSGRV
HCGVLGLRKWQFDVWSNDVTLANHMEAGGRAGRIHITRATLQYLNGDYEVEPGRGGERNA
YLKEQHIETFLILGASQKRKEEKAMLAKLQRTRANSMEGLMPRWVPDRAFSRTKDSKAFR
QMGIDDSSKDNRGTQDALNPEDEVDEFLSRAIDARSIDQLRKDHVRRFLLTFQREDLEKK
YSRKVDPRFGAYVACALLVFCFICFIQLLIFPHSTLMLGIYASIFLLLLITVLICAVYSC
GSLFPKALQRLSRSIVRSRAHSTAVGIFSVLLVFTSAIANMFTCNHTPIRSCAARMLNLT
PADITACHLQQLNYSLGLDAPLCEGTMPTCSFPEYFIGNMLLSLLASSVFLHISSIGKLA
MIFVLGLIYLVLLLLGPPATIFDNYDLLLGVHGLASSNETFDGLDCPAAGRVALKYMTPV
ILLVFALALYLHAQQVESTARLDFLWKLQATGEKEEMEELQAYNRRLLHNILPKDVAAHF
LARERRNDELYYQSCECVAVMFASIANFSEFYVELEANNEGVECLRLLNEIIADFDEIIS
EERFRQLEKIKTIGSTYMAASGLNASTYDQVGRSHITALADYAMRLMEQMKHINEHSFNN
FQMKIGLNMGPVVAGVIGARKPQYDIWGNTVNVSSRMDSTGVPDRIQVTTDLYQVLAAKG
YQLECRGVVKVKGKGEMTTYFLNGGPSS
>hsa:11202
MGRPRPRAAKTWMFLLLLGGAWAGHSRAQEDKVLGGHECQPHSQPWQAALFQGQQLLCGG
VLVGGNWVLTAAHCKKPKYTVRLGDHSLQNKDGPEQEIPVVQSIPHPCYNSSDVEDHNHD
LMLLQLRDQASLGSKVKPISLADHCTQPGQKCTVSGWGTVTSPRENFPDTLNCAEVKIFP
QKKCEDAYPGQITDGMVCAGSSKGADTCQGDSGGPLVCDGALQGITSWGSDPCGRSDKPG
VYTNICRYLDWIKKIIGSKG

>hsa:11221
MPPSPLDDRVVVALSRPVRPQDLNLCLDSSYLGSANPGSNSHPPVIATTVVSLKAANLTY
MPSSSGSARSLNCGCSSASCCTVATYDKDNQAQTQAIAAGTTTTAIGTSTTCPANQMVNN
NENTGSLSPSSGVGSPVSGTPKQLASIKIIYPNDLAKKMTKCSKSHLPSQGPVIIDCRPF
MEYNKSHIQGAVHINCADKISRRRLQQGKITVLDLISCREGKDSFKRIFSKEIIVYDENT
NEPSRVMPSQPLHIVLESLKREGKEPLVLKGGLSSFKQNHENLCDNSLQLQECREVGGGA
SAASSLLPQPIPTTPDIENAELTPILPFLFLGNEQDAQDLDTMQRLNIGYVINVTTHLPL
YHYEKGLFNYKRLPATDSNKQNLRQYFEEAFEFIEEAHQCGKGLLIHCQAGVSRSATIVI
AYLMKHTRMTMTDAYKFVKGKRPIISPNLNFMGQLLEFEEDLNNGVTPRILTPKLMGVET
VV
>hsa:11238
MVVMNSLRVILQASPGKLLWRKFQIPRFMPARPCSLYTCTYKTRNRALHPLWESVDLVPG
GDRQSPINIRWRDSVYDPGLKPLTISYDPATCLHVWNNGYSFLVEFEDSTDKSVIKGGPL
EHNYRLKQFHFHWGAIDAWGSEHTVDSKCFPAELHLVHWNAVRFENFEDAALEENGLAVI
GVFLKLGKHHKELQKLVDTLPSIKHKDALVEFGSFDPSCLMPTCPDYWTYSGSLTTPPLS
ESVTWIIKKQPVEVDHDQLEQFRTLLFTSEGEKEKRMVDNFRPLQPLMNRTVRSSFRHDY
VLNVQAKPKPATSQATP
>hsa:11266
MLEAPGPSDGCELSNPSASRVSCAGQMLEVQPGLYFGGAAAVAEPDHLREAGITAVLTVD
SEEPSFKAGPGVEDLWRLFVPALDKPETDLLSHLDRCVAFIGQARAEGRAVLVHCHAGVS
RSVAIITAFLMKTDQLPFEKAYEKLQILKPEAKMNEGFEWQLKLYQAMGYEVDTSSAIYK
QYRLQKVTEKYPELQNLPQELFAVDPTTVSQGLKDEVLYKCRKCRRSLFRSSSILDHREG
SGPIAFAHKRMTPSSMLTTGRQAQCTSYFIEPVQWMESALLGVMDGQLLCPKCSAKLGSF
NWYGEQCSCGRWITPAFQIHKNRVDEMKILPVLGSQTGKI
>hsa:113
MPAKGRYFLNEGEEGPDQDALYEKYQLTSQHGPLLLTLLLVAATACVALIIIAFSQGDPS
RHQAILGMAFLVLAVFAALSVLMYVECLLRRWLRALALLTWACLVALGYVLVFDAWTKAA
CAWEQVPFFLFIVFVVYTLLPFSMRGAVAVGAVSTASHLLVLGSLMGGFTTPSVRVGLQL
LANAVIFLCGNLTGAFHKHQMQDASRDLFTYTVKCIQIRRKLRIEKRQQENLLLSVLPAH
ISMGMKLAIIERLKEHGDRRCMPDNNFHSLYVKRHQNVSILYADIVGFTQLASDCSPKEL
VVVLNELFGKFDQIAKANECMRIKILGDCYYCVSGLPVSLPTHARNCVKMGLDMCQAIKQ
VREATGVDINMRVGIHSGNVLCGVIGLRKWQYDVWSHDVSLANRMEAAGVPGRVHITEAT
LKHLDKAYEVEDGHGQQRDPYLKEMNIRTYLVIDPRSQQPPPPSQHLPRPKGDAALKMRA
SVRMTRYLESWGAARPFAHLNHRESVSSGETHVPNGRRPKSVPQRHRRTPDRSMSPKGRS
EDDSYDDEMLSAIEGLSSTRPCCSKSDDFYTFGSIFLEKGFEREYRLAPIPRARHDFACA
SLIFVCILLVHVLLMPRTAALGVSFGLVACVLGLVLGLCFATKFSRCCPARGTLCTISER
VETQPLLRLTLAVLTIGSLLTVAIINLPLMPFQVPELPVGNETGLLAASSKTRALCEPLP
YYTCSCVLGFIACSVFLRMSLEPKVVLLTVALVAYLVLFNLSPCWQWDCCGQGLGNLTKP
NGTTSGTPSCSWKDLKTMTNFYLVLFYITLLTLSRQIDYYCRLDCLWKKKFKKEHEEFET
MENVNRLLLENVLPAHVAAHFIGDKLNEDWYHQSYDCVCVMFASVPDFKVFYTECDVNKE
GLECLRLLNEIIADFDELLLKPKFSGVEKIKTIGSTYMAAAGLSVASGHENQELERQHAH
IGVMVEFSIALMSKLDGINRHSFNSFRLRVGINHGPVIAGVIGARKPQYDIWGNTVNVAS
RMESTGELGKIQVTEETCTILQGLGYSCECRGLINVKGKGELRTYFVCTDTAKFQGLGLN
>hsa:11330

MLGITVLAALLACASSCGVPSFPPNLSARVVGGEDARPHSWPWQISLQYLKNDTWRHTCG
GTLIASNFVLTAAHCISNTRTYRVAVGKNNLEVEDEEGSLFVGVDTIHVHKRWNALLLRN
DIALIKLAEHVELSDTIQVACLPEKDSLLPKDYPCYVTGWGRLWTNGPIADKLQQGLQPV
VDHATCSRIDWWGFRVKKTMVCAGGDGVISACNGDSGGPLNCQLENGSWEVFGIVSFGSR
RGCNTRKKPVVYTRVSAYIDWINEKMQL
>hsa:11343
MPEESSPRRTPQSIPYQDLPHLVNADGQYLFCRYWKPTGTPKALIFVSHGAGEHSGRYEE
LARMLMGLDLLVFAHDHVGHGQSEGERMVVSDFHVFVRDVLQHVDSMQKDYPGLPVFLLG
HSMGGAIAILTAAERPGHFAGMVLISPLVLANPESATTFKVLAAKVLNLVLPNLSLGPID
SSVLSRNKTEVDIYNSDPLICRAGLKVCFGIQLLNAVSRVERALPKLTVPFLLLQGSADR
LCDSKGAYLLMELAKSQDKTLKIYEGAYHVLHKELPEVTNSVFHEINMWVSQRTATAGTA
SPP
>hsa:114
MELSDVRCLTGSEELYTIHPTPPAGDGRSASRPQRLLWQTAVRHITEQRFIHGHRGGSGS
GSGGSGKASDPAGGGPNHHAPQLSGDSALPLYSLGPGERAHSTCGTKVFPERSGSGSASG
SGGGGDLGFLHLDCAPSNSDFFLNGGYSYRGVIFPTLRNSFKSRDLERLYQRYFLGQRRK
SEVVMNVLDVLTKLTLLVLHLSLASAPMDPLKGILLGFFTGIEVVICALVVVRKDTTSHT
YLQYSGVVTWVAMTTQILAAGLGYGLLGDGIGYVLFTLFATYSMLPLPLTWAILAGLGTS
LLQVILQVVIPRLAVISINQVVAQAVLFMCMNTAGIFISYLSDRAQRQAFLETRRCVEAR
LRLETENQRQERLVLSVLPRFVVLEMINDMTNVEDEHLQHQFHRIYIHRYENVSILFADV
KGFTNLSTTLSAQELVRMLNELFARFDRLAHEHHCLRIKILGDCYYCVSGLPEPRQDHAH
CCVEMGLSMIKTIRYVRSRTKHDVDMRIGIHSGSVLCGVLGLRKWQFDVWSWDVDIANKL
ESGGIPGRIHISKATLDCLNGDYNVEEGHGKERNEFLRKHNIETYLIKQPEDSLLSLPED
IVKESVSSSDRRNSGATFTEGSWSPELPFDNIVGKQNTLAALTRNSINLLPNHLAQALHV
QSGPEEINKRIEHTIDLRSGDKLRREHIKPFSLMFKDSSLEHKYSQMRDEVFKSNLVCAF
IVLLFITAIQSLLPSSRVMPMTIQFSILIMLHSALVLITTAEDYKCLPLILRKTCCWINE
TYLARNVIIFASILINFLGAILNILWCDFDKSIPLKNLTFNSSAVFTDICSYPEYFVFTG
VLAMVTCAVFLRLNSVLKLAVLLIMIAIYALLTETVYAGLFLRYDNLNHSGEDFLGTKEV
SLLLMAMFLLAVFYHGQQLEYTARLDFLWRVQAKEEINEMKELREHNENMLRNILPSHVA
RHFLEKDRDNEELYSQSYDAVGVMFASIPGFADFYSQTEMNNQGVECLRLLNEIIADFDE
LLGEDRFQDIEKIKTIGSTYMAVSGLSPEKQQCEDKWGHLCALADFSLALTESIQEINKH
SFNNFELRIGISHGSVVAGVIGAKKPQYDIWGKTVNLASRMDSTGVSGRIQVPEETYLIL
KDQGFAFDYRGEIYVKGISEQEGKIKTYFLLGRVQPNPFILPPRRLPGQYSLAAVVLGLV
QSLNRQRQKQLLNENNNTGIIKGHYNRRTLLSPSGTEPGAQAEGTDKSDLP
>hsa:1147
MERPPGLRPGAGGPWEMRERLGTGGFGNVCLYQHRELDLKIAIKSCRLELSTKNRERWCH
EIQIMKKLNHANVVKACDVPEELNILIHDVPLLAMEYCSGGDLRKLLNKPENCCGLKESQ
ILSLLSDIGSGIRYLHENKIIHRDLKPENIVLQDVGGKIIHKIIDLGYAKDVDQGSLCTS
FVGTLQYLAPELFENKPYTATVDYWSFGTMVFECIAGYRPFLHHLQPFTWHEKIKKKDPK
CIFACEEMSGEVRFSSHLPQPNSLCSLVVEPMENWLQLMLNWDPQQRGGPVDLTLKQPRC
FVLMDHILNLKIVHILNMTSAKIISFLLPPDESLHSLQSRIERETGINTGSQELLSETGI
SLDPRKPASQCVLDGVRGCDSYMVYLFDKSKTVYEGPFASRSLSDCVNYIVQDSKIQLPI
IQLRKVWAEAVHYVSGLKEDYSRLFQGQRAAMLSLLRYNANLTKMKNTLISASQQLKAKL
EFFHKSIQLDLERYSEQMTYGISSEKMLKAWKEMEEKAIHYAEVGVIGYLEDQIMSLHAE

IMELQKSPYGRRQGDLMESLEQRAIDLYKQLKHRPSDHSYSDSTEMVKIIVHTVQSQDRV
LKELFGHLSKLLGCKQKIIDLLPKVEVALSNIKEADNTVMFMQGKRQKEIWHLLKIACTQ
SSARSLVGSSLEGAVTPQTSAWLPPTSAEHDHSLSCVVTPQDGETSAQMIEENLNCLGHL
STIIHEANEEQGNSMMNLDWSWLTE
>hsa:115
MASPPHQQLLHHHSTEVSCDSSGDSNSVRVKINPKQLSSNSHPKHCKYSISSSCSSSGDS
GGVPRRVGGGGRLRRQKKLPQLFERASSRWWDPKFDSVNLEEACLERCFPQTQRRFRYAL
FYIGFACLLWSIYFAVHMRSRLIVMVAPALCFLLVCVGFFLFTFTKLYARHYAWTSLALT
LLVFALTLAAQFQVLTPVSGRGDSSNLTATARPTDTCLSQVGSFSMCIEVLFLLYTVMHL
PLYLSLCLGVAYSVLFETFGYHFRDEACFPSPGAGALHWELLSRGLLHGCIHAIGVHLFV
MSQVRSRSTFLKVGQSIMHGKDLEVEKALKERMIHSVMPRIIADDLMKQGDEESENSVKR
HATSSPKNRKKKSSIQKAPIAFRPFKMQQIEEVSILFADIVGFTKMSANKSAHALVGLLN
DLFGRFDRLCEETKCEKISTLGDCYYCVAGCPEPRADHAYCCIEMGLGMIKAIEQFCQEK
KEMVNMRVGVHTGTVLCGILGMRRFKFDVWSNDVNLANLMEQLGVAGKVHISEATAKYLD
DRYEMEDGKVIERLGQSVVADQLKGLKTYLISGQRAKESRCSCAEALLSGFEVIDGSQVS
SGPRGQGTASSGNVSDLAQTVKTFDNLKTCPSCGITFAPKSEAGAEGGAPQNGCQDEHKN
STKASGGPNPKTQNGLLSPPQEEKLTNSQTSLCEILQEKGRWAGVSLDQSALLPLRFKNI
REKTDAHFVDVIKEDSLMKDYFFKPPINQFSLNFLDQELERSYRTSYQEEVIKNSPVKTF
ASPTFSSLLDVFLSTTVFLTLSTTCFLKYEAATVPPPPAALAVFSAALLLEVLSLAVSIR
MVFFLEDVMACTKRLLEWIAGWLPRHCIGAILVSLPALAVYSHVTSEYETNIHFPVFTGS
AALIAVVHYCNFCQLSSWMRSSLATVVGAGPLLLLYVSLCPDSSVLTSPLDAVQNFSSER
NPCNSSVPRDLRRPASLIGQEVVLVFFLLLLLVWFLNREFEVSYRLHYHGDVEADLHRTK
IQSMRDQADWLLRNIIPYHVAEQLKVSQTYSKNHDSGGVIFASIVNFSEFYEENYEGGKE
CYRVLNELIGDFDELLSKPDYSSIEKIKTIGATYMAASGLNTAQAQDGSHPQEHLQILFE
FAKEMMRVVDDFNNNMLWFNFKLRVGFNHGPLTAGVIGTTKLLYDIWGDTVNIASRMDTT
GVECRIQVSEESYRVLSKMGYDFDYRGTVNVKGKGQMKTYLYPKCTDHRVIPQHQLSISP
DIRVQVDGSIGRSPTDEIANLVPSVQYVDKTSLGSDSSTQAKDAHLSPKRPWKEPVKAEE
RGRFGKAIEKDDCDETGIEEANELTKLNVSKSV
>hsa:116447
MLDHEYTTKEVFRKNFFNDWRKEMAVEEREVIKSLDKCDFTEIHRYFVDKAAARKVLSRE
EKQKLKEEAEKLQQEFGYCILDGHQEKIGNFKIEPPGLFRGRGDHPKMGMLKRRITPEDV
VINCSRDSKIPEPPAGHQWKEVRSDNTVTWLAAWTESVQNSIKYIMLNPCSKLKGETAWQ
KFETARRLRGFVDEIRSQYRADWKSREMKTRQRAVALYFIDKLALRAGNEKEDGEAADTV
GCCSLRVEHVQLHPEADGCQHVVEFDFLGKDCIRYYNRVPVEKPVYKNLQLFMENKDPRD
DLFDRLTTTSLNKHLQELMDGLTAKVFRTYNASITLQEQLRALTRAEDSIAAKILSYNRA
NRVVAILCNHQRATPSTFEKSMQNLQTKIQAKKEQVAEARAELRRARAEHKAQGDGKSRS
VLEKKRRLLEKLQEQLAQLSVQATDKEENKQVALGTSKLNYLDPRISIAWCKRFRVPVEK
IYSKTQRERFAWALAMAGEDFEF
>hsa:1178
MSLLPVPYTEAASLSTGSTVTIKGRPLACFLNEPYLQVDFHTEMKEESDIVFHFQVCFGR
RVVMNSREYGAWKQQVESKNMPFQDGQEFELSISVLPDKYQVMVNGQSSYTFDHRIKPEA
VKMVQVWRDISLTKFNVSYLKR
>hsa:1200
MGLQACLLGLFALILSGKCSYSPEPDQRRTLPPGWVSLGRADPEEELSLTFALRQQNVER

LSELVQAVSDPSSPQYGKYLTLENVADLVRPSPLTLHTVQKWLLAAGAQKCHSVITQDFL
TCWLSIRQAELLLPGAEFHHYVGGPTETHVVRSPHPYQLPQALAPHVDFVGGLHRFPPTS
SLRQRPEPQVTGTVGLHLGVTPSVIRKRYNLTSQDVGSGTSNNSQACAQFLEQYFHDSDL
AQFMRLFGGNFAHQASVARVVGQQGRGRAGIEASLDVQYLMSAGANISTWVYSSPGRHEG
QEPFLQWLMLLSNESALPHVHTVSYGDDEDSLSSAYIQRVNTELMKAAARGLTLLFASGD
SGAGCWSVSGRHQFRPTFPASSPYVTTVGGTSFQEPFLITNEIVDYISGGGFSNVFPRPS
YQEEAVTKFLSSSPHLPPSSYFNASGRAYPDVAALSDGYWVVSNRVPIPWVSGTSASTPV
FGGILSLINEHRILSGRPPLGFLNPRLYQQHGAGLFDVTRGCHESCLDEEVEGQGFCSGP
GWDPVTGWGTPNFPALLKTLLNP
>hsa:1215
MLLLPLPLLLFLLCSRAEAGEIIGGTECKPHSRPYMAYLEIVTSNGPSKFCGGFLIRRNF
VLTAAHCAGRSITVTLGAHNITEEEDTWQKLEVIKQFRHPKYNTSTLHHDIMLLKLKEKA
SLTLAVGTLPFPSQFNFVPPGRMCRVAGWGRTGVLKPGSDTLQEVKLRLMDPQACSHFRD
FDHNLQLCVGNPRKTKSAFKGDSGGPLLCAGVAQGIVSYGRSDAKPPAVFTRISHYRPWI
NQILQAN
>hsa:124
MSTAGKVIKCKAAVLWELKKPFSIEEVEVAPPKAHEVRIKMVAVGICGTDDHVVSGTMVT
PLPVILGHEAAGIVESVGEGVTTVKPGDKVIPLAIPQCGKCRICKNPESNYCLKNDVSNP
QGTLQDGTSRFTCRRKPIHHFLGISTFSQYTVVDENAVAKIDAASPLEKVCLIGCGFSTG
YGSAVNVAKVTPGSTCAVFGLGGVGLSAIMGCKAAGAARIIAVDINKDKFAKAKELGATE
CINPQDYKKPIQEVLKEMTDGGVDFSFEVIGRLDTMMASLLCCHEACGTSVIVGVPPDSQ
NLSMNPMLLLTGRTWKGAILGGFKSKECVPKLVADFMAKKFSLDALITHVLPFEKINEGF
DLLHSGKSIRTILMF
>hsa:125
MSTAGKVIKCKAAVLWEVKKPFSIEDVEVAPPKAYEVRIKMVAVGICHTDDHVVSGNLVT
PLPVILGHEAAGIVESVGEGVTTVKPGDKVIPLFTPQCGKCRVCKNPESNYCLKNDLGNP
RGTLQDGTRRFTCRGKPIHHFLGTSTFSQYTVVDENAVAKIDAASPLEKVCLIGCGFSTG
YGSAVNVAKVTPGSTCAVFGLGGVGLSAVMGCKAAGAARIIAVDINKDKFAKAKELGATE
CINPQDYKKPIQEVLKEMTDGGVDFSFEVIGRLDTMMASLLCCHEACGTSVIVGVPPASQ
NLSINPMLLLTGRTWKGAVYGGFKSKEGIPKLVADFMAKKFSLDALITHVLPFEKINEGF
DLLHSGKSIRTVLTF
>hsa:126
MSTAGKVIKCKAAVLWELKKPFSIEEVEVAPPKAHEVRIKMVAAGICRSDEHVVSGNLVT
PLPVILGHEAAGIVESVGEGVTTVKPGDKVIPLFTPQCGKCRICKNPESNYCLKNDLGNP
RGTLQDGTRRFTCSGKPIHHFVGVSTFSQYTVVDENAVAKIDAASPLEKVCLIGCGFSTG
YGSAVKVAKVTPGSTCAVFGLGGVGLSVVMGCKAAGAARIIAVDINKDKFAKAKELGATE
CINPQDYKKPIQEVLKEMTDGGVDFSFEVIGRLDTMMASLLCCHEACGTSVIVGVPPDSQ
NLSINPMLLLTGRTWKGAIFGGFKSKESVPKLVADFMAKKFSLDALITNILPFEKINEGF
DLLRSGKSIRTVLTF
>hsa:1267
MNRGFSRKSHTFLPKIFFRKMSSSGAKDKPELQFPFLQDEDTVATLLECKTLFILRGLPG
SGKSTLARVIVDKYRDGTKMVSADAYKITPGARGAFSEEYKRLDEDLAAYCRRRDIRILV
LDDTNHERERLEQLFEMADQYQYQVVLVEPKTAWRLDCAQLKEKNQWQLSADDLKKLKPG
LEKDFLPLYFGWFLTKKSSETLRKAGQVFLEELGNHKAFKKELRQFVPGDEPREKMDLVT

YFGKRPPGVLHCTTKFCDYGKAPGAEEYAQQDVLKKSYSKAFTLTISALFVTPKTTGARV
ELSEQQLQLWPSDVDKLSPTDNLPRGSRAHITLGCAADVEAVQTGLDLLEILRQEKGGSR
GEEVGELSRGKLYSLGNGRWMLTLAKNMEVRAIFTGYYGKGKPVPTQGSRKGGALQSCTI
I
>hsa:128
MANEVIKCKAAVAWEAGKPLSIEEIEVAPPKAHEVRIKIIATAVCHTDAYTLSGADPEGC
FPVILGHEGAGIVESVGEGVTKLKAGDTVIPLYIPQCGECKFCLNPKTNLCQKIRVTQGK
GLMPDGTSRFTCKGKTILHYMGTSTFSEYTVVADISVAKIDPLAPLDKVCLLGCGISTGY
GAAVNTAKLEPGSVCAVFGLGGVGLAVIMGCKVAGASRIIGVDINKDKFARAKEFGATEC
INPQDFSKPIQEVLIEMTDGGVDYSFECIGNVKVMRAALEACHKGWGVSVVVGVAASGEE
IATRPFQLVTGRTWKGTAFGGWKSVESVPKLVSEYMSKKIKVDEFVTHNLSFDEINKAFE
LMHSGKSIRTVVKI
>hsa:128853
MTVTGLGWRDVLEAIKATRPIANPNPGFRQQLEEFGWASSQKLRRQLEERFGESPFRDEE
ELRALLPLCKRCRQGSATSASSAGPHSAASEGTVQRLVPRTPREAHRPLPLLARVKQTFS
CLPRCLSRKGGK
>hsa:129807
MMSSAAFPRWLQSMGVPRTPSRTVLFERERTGLTYRVPSLLPVPPGPTLLAFVEQRLSPD
DSHAHRLVLRRGTLAGGSVRWGALHVLGTAALAEHRSMNPCPVHDAGTGTVFLFFIAVLG
HTPEAVQIATGRNAARLCCVASRDAGLSWGSARDLTEEAIGGAVQDWATFAVGPGHGVQL
PSGRLLVPAYTYRVDRRECFGKICRTSPHSFAFYSDDHGRTWRCGGLVPNLRSGECQLAA
VDGGQAGSFLYCNARSPLGSRVQALSTDEGTSFLPAERVASLPETAWGCQGSIVGFPAPA
PNRPRDDSWSVGPGSPLQPPLLGPGVHEPPEEAAVDPRGGQVPGGPFSRLQPRGDGPRQP
GPRPGVSGDVGSWTLALPMPFAAPPQSPTWLLYSHPVGRRARLHMGIRLSQSPLDPRSWT
EPWVIYEGPSGYSDLASIGPAPEGGLVFACLYESGARTSYDEISFCTFSLREVLENVPAS
PKPPNLGDKPRGCCWPS
>hsa:130399
MLTNGKEQVIKSCVSLPELNAQVFCHSSNNVTKTECCFTDFCNNITLHLPTASPNAPKLG
PMELAIIITVPVCLLSIAAMLTVWACQGRQCSYRKKKRPNVEEPLSECNLVNAGKTLKDL
IYDVTASGSGSGLPLLVQRTIARTIVLQEIVGKGRFGEVWHGRWCGEDVAVKIFSSRDER
SWFREAEIYQTVMLRHENILGFIAADNKDNGTWTQLWLVSEYHEQGSLYDYLNRNIVTVA
GMIKLALSIASGLAHLHMEIVGTQGKPAIAHRDIKSKNILVKKCETCAIADLGLAVKHDS
ILNTIDIPQNPKVGTKRYMAPEMLDDTMNVNIFESFKRADIYSVGLVYWEIARRCSVGGI
VEEYQLPYYDMVPSDPSIEEMRKVVCDQKFRPSIPNQWQSCEALRVMGRIMRECWYANGA
ARLTALRIKKTISQLCVKEDCKA
>hsa:131
MFAEIQIQDKDRMGTAGKVIKCKAAVLWEQKQPFSIEEIEVAPPKTKEVRIKILATGICR
TDDHVIKGTMVSKFPVIVGHEATGIVESIGEGVTTVKPGDKVIPLFLPQCRECNACRNPD
GNLCIRSDITGRGVLADGTTRFTCKGKPVHHFMNTSTFTEYTVVDESSVAKIDDAAPPEK
VCLIGCGFSTGYGAAVKTGKVKPGSTCVVFGLGGVGLSVIMGCKSAGASRIIGIDLNKDK
FEKAMAVGATECISPKDSTKPISEVLSEMTGNNVGYTFEVIGHLETMIDALASCHMNYGT
SVVVGVPPSAKMLTYDPMLLFTGRTWKGCVFGGLKSRDDVPKLVTEFLAKKFDLDQLITH
VLPFKKISEGFELLNSGQSIRTVLTF
>hsa:1312

MPEAPPLLLAAVLLGLVLLVVLLLLLRHWGWGLCLIGWNEFILQPIHNLLMGDTKEQRIL
NHVLQHAEPGNAQSVLEAIDTYCEQKEWAMNVGDKKGKIVDAVIQEHQPSVLLELGAYCG
YSAVRMARLLSPGARLITIEINPDCAAITQRMVDFAGVKDKVTLVVGASQDIIPQLKKKY
DVDTLDMVFLDHWKDRYLPDTLLLEECGLLRKGTVLLADNVICPGAPDFLAHVRGSSCFE
CTHYQSFLEYREVVDGLEKAIYKGPGSEAGP
>hsa:132
MTSVRENILFGMGNPLLDISAVVDKDFLDKYSLKPNDQILAEDKHKELFDELVKKFKVEY
HAGGSTQNSIKVAQWMIQQPHKAATFFGCIGIDKFGEILKRKAAEAHVDAHYYEQNEQPT
GTCAACITGDNRSLIANLAAANCYKKEKHLDLEKNWMLVEKARVCYIAGFFLTVSPESVL
KVAHHASENNRIFTLNLSAPFISQFYKESLMKVMPYVDILFGNETEAATFAREQGFETKD
IKEIAKKTQALPKMNSKRQRIVIFTQGRDDTIMATESEVTAFAVLDQDQKEIIDTNGAGD
AFVGGFLSQLVSDKPLTECIRAGHYAASIIIRRTGCTFPEKPDFH
>hsa:132160
MGGCTALVAVSLQGKLYMANAGDSRAILVRRDEIRPLSFEFTPETERQRIQQLAFVYPEL
LAGEFTRLEFPRRLKGDDLGQKVLFRDHHMSGWSYKRVEKSDLKYPLIHGQGRQARLLGT
LAVSRGLGDHQLRVLDTNIQLKPFLLSVPQVTVLDVDQLELQEDDVVVMATDGLWDVLSN
EQVAWLVRSFLPGNQEDPHRFSKLAQMLIHSTQGKEDSLTEEGQVSYDDVSVFVIPLHSQ
GQESSDH
>hsa:133121
MAVKLGTLLLALALGLAQPASARRKLLVFLLDGFRSDYISDEALESLPGFKEIVSRGVKV
DYLTPDFPSLSYPNYYTLMTGRHCEVHQMIGNYMWDPTTNKSFDIGVNKDSLMPLWWNGS
EPLWVTLTKAKRKVYMYYWPGCEVEILGVRPTYCLEYKNVPTDINFANAVSDALDSFKSG
RADLAAIYHERIDVEGHHYGPASPQRKDALKAVDTVLKYMTKWIQERGLQDRLNVIIFSD
HGMTDIFWMDKVIELNKYISLNDLQQVKDRGPVVSLWPAPGKHSEIYNKLSTVEHMTVYE
KEAIPSRFYYKKGKFVSPLTLVADEGWFITENREMLPFWMNSTGRREGWQRGWHGYDNEL
MDMRGIFLAFGPDFKSNFRAAPIRSVDVYNVMCNVVGITPLPNNGSWSRVMCMLKGRAST
APPVWPSHCALALILLFLLA
>hsa:134510
MALPIIVKWGGQEYSVTTLSEDDTVLDLKQFLKTLTGVLPERQKLLGLKVKGKPAENDVK
LGALKLKPNTKIMMMGTREESLEDVLGPPPDNDDVVNDFDIEDEVVEVENREENLLKISR
RVKEYKVEILNPPREGKKLLVLDVDYTLFDHRSCAETGVELMRPYLHEFLTSAYEDYDIV
IWSATNMKWIEAKMKELGVSTNANYKITFMLDSAAMITVHTPRRGLIDVKPLGVIWGKFS
EFYSKKNTIMFDDIGRNFLMNPQNGLKIRPFMKAHLNRDKDKELLKLTQYLKEIAKLDDF
LDLNHKYWERYLSKKQGQ
>hsa:1360
MLALLVLVTVALASAHHGGEHFEGEKVFRVNVEDENHINIIRELASTTQIDFWKPDSVTQ
IKPHSTVDFRVKAEDTVTVENVLKQNELQYKVLISNLRNVVEAQFDSRVRATGHSYEKYN
KWETIEAWTQQVATENPALISRSVIGTTFEGRAIYLLKVGKAGQNKPAIFMDCGFHAREW
ISPAFCQWFVREAVRTYGREIQVTELLDKLDFYVLPVLNIDGYIYTWTKSRFWRKTRSTH
TGSSCIGTDPNRNFDAGWCEIGASRNPCDETYCGPAAESEKETKALADFIRNKLSSIKAY
LTIHSYSQMMIYPYSYAYKLGENNAELNALAKATVKELASLHGTKYTYGPGATTIYPAAG
GSDDWAYDQGIRYSFTFELRDTGRYGFLLPESQIRATCEETFLAIKYVASYVLEHLY
>hsa:1363
MAGRGGSALLALCGALAACGWLLGAEAQEPGAPAAGMRRRRRLQQEDGISFEYHRYPELR

EALVSVWLQCTAISRIYTVGRSFEGRELLVIELSDNPGVHEPGEPEFKYIGNMHGNEAVG
RELLIFLAQYLCNEYQKGNETIVNLIHSTRIHIMPSLNPDGFEKAASQPGELKDWFVGRS
NAQGIDLNRNFPDLDRIVYVNEKEGGPNNHLLKNMKKIVDQNTKLAPETKAVIHWIMDIP
FVLSANLHGGDLVANYPYDETRSGSAHEYSSSPDDAIFQSLARAYSSFNPAMSDPNRPPC
RKNDDDSSFVDGTTNGGAWYSVPGGMQDFNYLSSNCFEITVELSCEKFPPEETLKTYWED
NKNSLISYLEQIHRGVKGFVRDLQGNPIANATISVEGIDHDVTSAKDGDYWRLLIPGNYK
LTASAPGYLAITKKVAVPYSPAAGVDFELESFSERKEEEKEELMEWWKMMSETLNF
>hsa:1374
MAEAHQAVAFQFTVTPDGIDLRLSHEALRQIYLSGLHSWKKKFIRFKNGIITGVYPASPS
SWLIVVVGVMTTMYAKIDPSLGIIAKINRTLETANCMSSQTKNVVSGVLFGTGLWVALIV
TMRYSLKVLLSYHGWMFTEHGKMSRATKIWMGMVKIFSGRKPMLYSFQTSLPRLPVPAVK
DTVNRYLQSVRPLMKEEDFKRMTALAQDFAVGLGPRLQWYLKLKSWWATNYVSDWWEEYI
YLRGRGPLMVNSNYYAMDLLYILPTHIQAARAGNAIHAILLYRRKLDREEIKPIRLLGST
IPLCSAQWERMFNTSRIPGEETDTIQHMRDSKHIVVYHRGRYFKVWLYHDGRLLKPREME
QQMQRILDNTSEPQPGEARLAALTAGDRVPWARCRQAYFGRGKNKQSLDAVEKAAFFVTL
DETEEGYRSEDPDTSMDSYAKSLLHGRCYDRWFDKSFTFVVFKNGKMGLNAEHSWADAPI
VAHLWEYVMSIDSLQLGYAEDGHCKGDINPNIPYPTRLQWDIPGECQEVIETSLNTANLL
ANDVDFHSFPFVAFGKGIIKKCRTSPDAFVQLALQLAHYKDMGKFCLTYEASMTRLFREG
RTETVRSCTTESCDFVRAMVDPAQTVEQRLKLFKLASEKHQHMYRLAMTGSGIDRHLFCL
YVVSKYLAVESPFLKEVLSEPWRLSTSQTPQQQVELFDLENNPEYVSSGGGFGPVADDGY
GVSYILVGENLINFHISSKFSCPETGIISQGPSSDT
>hsa:1376
MVPRLLLRAWPRGPAVGPGAPSRPLSAGSGPGQYLQRSIVPTMHYQDSLPRLPIPKLEDT
IRRYLSAQKPLLNDGQFRKTEQFCKSFENGIGKELHEQLVALDKQNKHTSYISGPWFDMY
LSARDSVVLNFNPFMAFNPDPKSEYNDQLTRATNMTVSAIRFLKTLRAGLLEPEVFHLNP
AKSDTITFKRLIRFVPSSLSWYGAYLVNAYPLDMSQYFRLFNSTRLPKPSRDELFTDDKA
RHLLVLRKGNFYIFDVLDQDGNIVSPSEIQAHLKYILSDSSPAPEFPLAYLTSENRDIWA
ELRQKLMSSGNEESLRKVDSAVFCLCLDDFPIKDLVHLSHNMLHGDGTNRWFDKSFNLII
AKDGSTAVHFEHSWGDGVAVLRFFNEVFKDSTQTPAVTPQSQPATTDSTVTVQKLNFELT
DALKTGITAAKEKFDATMKTLTIDCVQFQRGGKEFLKKQKLSPDAVAQLAFQMAFLRQYG
QTVATYESCSTAAFKHGRTETIRPASVYTKRCSEAFVREPSRHSAGELQQMMVECSKYHG
QLTKEAAMGQGFDRHLFALRHLAAAKGIILPELYLDPAYGQINHNVLSTSTLSSPAVNLG
GFAPVVSDGFGVGYAVHDNWIGCNVSSYPGRNAREFLQCVEKALEDMFDALEGKSIKS
>hsa:1384
MLAFAARTVVKPLGFLKPFSLMKASSRFKAHQDALPRLPVPPLQQSLDHYLKALQPIVSE
EEWAHTKQLVDEFQASGGVGERLQKGLERRARKTENWLSEWWLKTAYLQYRQPVVIYSSP
GVMLPKQDFVDLQGQLRFAAKLIEGVLDFKVMIDNETLPVEYLGGKPLCMNQYYQILSSC
RVPGPKQDTVSNFSKTKKPPTHITVVHNYQFFELDVYHSDGTPLTADQIFVQLEKIWNSS
LQTNKEPVGILTSNHRNSWAKAYNTLIKDKVNRDSVRSIQKSIFTVCLDATMPRVSEDVY
RSHVAGQMLHGGGSRLNSGNRWFDKTLQFIVAEDGSCGLVYEHAAAEGPPIVTLLDYVIE
YTKKPELVRSPMVPLPMPKKLRFNITPEIKSDIEKAKQNLSIMIQDLDITVMVFHHFGKD
FPKSEKLSPDAFIQMALQLAYYRIYGQACATYESASLRMFHLGRTDTIRSASMDSLTFVK
AMDDSSVTEHQKVELLRKAVQAHRGYTDRAIRGEAFDRHLLGLKLQAIEDLVSMPDIFMD
TSYAIAMHFHLSTSQVPAKTDCVMFFGPVVPDGYGVCYNPMEAHINFSLSAYNSCAETNA

ARLAHYLEKALLDMRALLQSHPRAKL
>hsa:142679
MYSLNQEIKAFSRNNLRKQCTRVTTLTGKKIIETWKDARIHVVEEVEPSSGGGCGYVQDL
SSDLQVGVIKPWLLLGSQDAAHDLDTLKKNKDGVVLVHCNAGVSRAAAIVIGFLMNSEQT
SFTSAFSLVKNARPSICPNSGFMEQLRTYQEGKESNKCDRIQENSS
>hsa:1429
MATGQKLMRAVRVFEFGGPEVLKLRSDIAVPIPKDHQVLIKVHACGVNPVETYIRSGTYS
RKPLLPYTPGSDVAGVIEAVGDNASAFKKGDRVFTSSTISGGYAEYALAADHTVYKLPEK
LDFKQGAAIGIPYFTAYRALIHSACVKAGESVLVHGASGGVGLAACQIARAYGLKILGTA
GTEEGQKIVLQNGAHEVFNHREVNYIDKIKKYVGEKGIDIIIEMLANVNLSKDLSLLSHG
GRVIVVGSRGTIEINPRDTMAKESSIIGVTLFSSTKEEFQQYAAALQAGMEIGWLKPVIG
SQYPLEKVAEAHENIIHGSGATGKMILLL
>hsa:1431
MALLTAAARLLGTKNASCLVLAARHASASSTNLKDILADLIPKEQARIKTFRQQHGKTVV
GQITVDMMYGGMRGMKGLVYETSVLDPDEGIRFRGFSIPECQKLLPKAKGGEEPLPEGLF
WLLVTGHIPTEEQVSWLSKEWAKRAALPSHVVTMLDNFPTNLHPMSQLSAAVTALNSESN
FARAYAQGISRTKYWELIYEDSMDLIAKLPCVAAKIYRNLYREGSGIGAIDSNLDWSHNF
TNMLGYTDHQFTELTRLYLTIHSDHEGGNVSAHTSHLVGSALSDPYLSFAAAMNGLAGPL
HGLANQEVLVWLTQLQKEVGKDVSDEKLRDYIWNTLNSGRVVPGYGHAVLRKTDPRYTCQ
REFALKHLPNDPMFKLVAQLYKIVPNVLLEQGKAKNPWPNVDAHSGVLLQYYGMTEMNYY
TVLFGVSRALGVLAQLIWSRALGFPLERPKSMSTEGLMKFVDSKSG
>hsa:1432
MSQERPTFYRQELNKTIWEVPERYQNLSPVGSGAYGSVCAAFDTKTGLRVAVKKLSRPFQ
SIIHAKRTYRELRLLKHMKHENVIGLLDVFTPARSLEEFNDVYLVTHLMGADLNNIVKCQ
KLTDDHVQFLIYQILRGLKYIHSADIIHRDLKPSNLAVNEDCELKILDFGLARHTDDEMT
GYVATRWYRAPEIMLNWMHYNQTVDIWSVGCIMAELLTGRTLFPGTDHINQLQQIMRLTG
TPPAYLINRMPSHEARNYIQSLTQMPKMNFANVFIGANPLAVDLLEKMLVLDSDKRITAA
QALAHAYFAQYHDPDDEPVADPYDQSFESRDLLIDEWKSLTYDEVISFVPPPLDQEEMES
>hsa:1436
MGPGVLLLLLVATAWHGQGIPVIEPSVPELVVKPGATVTLRCVGNGSVEWDGPPSPHWTL
YSDGSSSILSTNNATFQNTGTYRCTEPGDPLGGSAAIHLYVKDPARPWNVLAQEVVVFED
QDALLPCLLTDPVLEAGVSLVRVRGRPLMRHTNYSFSPWHGFTIHRAKFIQSQDYQCSAL
MGGRKVMSISIRLKVQKVIPGPPALTLVPAELVRIRGEAAQIVCSASSVDVNFDVFLQHN
NTKLAIPQQSDFHNNRYQKVLTLNLDQVDFQHAGNYSCVASNVQGKHSTSMFFRVVESAY
LNLSSEQNLIQEVTVGEGLNLKVMVEAYPGLQGFNWTYLGPFSDHQPEPKLANATTKDTY
RHTFTLSLPRLKPSEAGRYSFLARNPGGWRALTFELTLRYPPEVSVIWTFINGSGTLLCA
ASGYPQPNVTWLQCSGHTDRCDEAQVLQVWDDPYPEVLSQEPFHKVTVQSLLTVETLEHN
QTYECRAHNSVGSGSWAFIPISAGAHTHPPDEFLFTPVVVACMSIMALLLLLLLLLLYKY
KQKPKYQVRWKIIESYEGNSYTFIDPTQLPYNEKWEFPRNNLQFGKTLGAGAFGKVVEAT
AFGLGKEDAVLKVAVKMLKSTAHADEKEALMSELKIMSHLGQHENIVNLLGACTHGGPVL
VITEYCCYGDLLNFLRRKAEAMLGPSLSPGQDPEGGVDYKNIHLEKKYVRRDSGFSSQGV
DTYVEMRPVSTSSNDSFSEQDLDKEDGRPLELRDLLHFSSQVAQGMAFLASKNCIHRDVA
ARNVLLTNGHVAKIGDFGLARDIMNDSNYIVKGNARLPVKWMAPESIFDCVYTVQSDVWS
YGILLWEIFSLGLNPYPGILVNSKFYKLVKDGYQMAQPAFAPKNIYSIMQACWALEPTHR

PTFQQICSFLQEQAQEDRRERDYTNLPSSSRSGGSGSSSSELEEESSSEHLTCCEQGDIA
QPLLQPNNYQFC
>hsa:1445
MSAIQAAWPSGTECIAKYNFHGTAEQDLPFCKGDVLTIVAVTKDPNWYKAKNKVGREGII
PANYVQKREGVKAGTKLSLMPWFHGKITREQAERLLYPPETGLFLVRESTNYPGDYTLCV
SCDGKVEHYRIMYHASKLSIDEEVYFENLMQLVEHYTSDADGLCTRLIKPKVMEGTVAAQ
DEFYRSGWALNMKELKLLQTIGKGEFGDVMLGDYRGNKVAVKCIKNDATAQAFLAEASVM
TQLRHSNLVQLLGVIVEEKGGLYIVTEYMAKGSLVDYLRSRGRSVLGGDCLLKFSLDVCE
AMEYLEGNNFVHRDLAARNVLVSEDNVAKVSDFGLTKEASSTQDTGKLPVKWTAPEALRE
KKFSTKSDVWSFGILLWEIYSFGRVPYPRIPLKDVVPRVEKGYKMDAPDGCPPAVYEVMK
NCWHLDAAMRPSFLQLREQLEHIKTHELHL
>hsa:150290
MTAPSCAFPVQFRQPSVSGLSQITKSLYISNGVAANNKLMLSSNQITMVINVSVEVVNTL
YEDIQYMQVPVADSPNSRLCDFFDPIADHIHSVEMKQGRTLLHCAAGVSRSAALCLAYLM
KYHAMSLLDAHTWTKSCRPIIRPNSGFWEQLIHYEFQLFGKNTVHMVSSPVGMIPDIYEK
EVRLMIPL
>hsa:1504
MASLWLLSCFSLVGAAFGCGVPAIHPVLSGLSRIVNGEDAVPGSWPWQVSLQDKTGFHFC
GGSLISEDWVVTAAHCGVRTSDVVVAGEFDQGSDEENIQVLKIAKVFKNPKFSILTVNND
ITLLKLATPARFSQTVSAVCLPSADDDFPAGTLCATTGWGKTKYNANKTPDKLQQAALPL
LSNAECKKSWGRRITDVMICAGASGVSSCMGDSGGPLVCQKDGAWTLVGIVSWGSDTCST
SSPGVYARVTKLIPWVQKILAAN
>hsa:151531
MLAPGCELDPDQEVVRTRPEDVPASPSTSTMIVSVLRPPSHASCTACGTVTFHIVERMAS
VIPASNRSMRSDRNTYVGKRFVHVKNPYLDLMDEDILYHLDLGTKTHNLPAMFGDVKFVC
VGGSPNRMKAFALFMHKELGFEEAEEDIKDICAGTDRYCMYKTGPVLAISHGMGIPSISI
MLHELIKLLHHARCCDVTIIRIGTSGGIGIAPGTVVITDIAVDSFFKPRFEQVILDNIVT
RSTELDKELSEELFNCSKEIPNFPTLVGHTMCTYDFYEGQGRLDGALCSFSREKKLDYLK
RAFKAGVRNIEMESTVFAAMCGLCGLKAAVVCVTLLDRLDCDQINLPHDVLVEYQQRPQL
LISNFIRRRLGLCD
>hsa:1537
MAAAAASLRGVVLGPRGAGLPGARARGLLCSARPGQLPLRTPQAVALSSKSGLSRGRKVM
LSALGMLAAGGAGLAVALHSAVSASDLELHPPSYPWSHRGLLSSLDHTSIRRGFQVYKQV
CASCHSMDFVAYRHLVGVCYTEDEAKELAAEVEVQDGPNEDGEMFMRPGKLFDYFPKPYP
NSEAARAANNGALPPDLSYIVRARHGGEDYVFSLLTGYCEPPTGVSLREGLYFNPYFPGQ
AIAMAPPIYTDVLEFDDGTPATMSQIAKDVCTFLRWASEPEHDHRKRMGLKMLMMMALLV
PLVYTIKRHKWSVLKSRKLAYRPPK
>hsa:1543
MLFPISMSATEFLLASVIFCLVFWVIRASRPQVPKGLKNPPGPWGWPLIGHMLTLGKNPH
LALSRMSQQYGDVLQIRIGSTPVVVLSGLDTIRQALVRQGDDFKGRPDLYTFTLISNGQS
MSFSPDSGPVWAARRRLAQNGLKSFSIASDPASSTSCYLEEHVSKEAEVLISTLQELMAG
PGHFNPYRYVVVSVTNVICAICFGRRYDHNHQELLSLVNLNNNFGEVVGSGNPADFIPIL
RYLPNPSLNAFKDLNEKFYSFMQKMVKEHYKTFEKGHIRDITDSLIEHCQEKQLDENANV
QLSDEKIINIVLDLFGAGFDTVTTAISWSLMYLVMNPRVQRKIQEELDTVIGRSRRPRLS

DRSHLPYMEAFILETFRHSSFVPFTIPHSTTRDTSLKGFYIPKGRCVFVNQWQINHDQKL
WVNPSEFLPERFLTPDGAIDKVLSEKVIIFGMGKRKCIGETIARWEVFLFLAILLQRVEF
SVPLGVKVDMTPIYGLTMKHACCEHFQMQLRS
>hsa:1544
MALSQSVPFSATELLLASAIFCLVFWVLKGLRPRVPKGLKSPPEPWGWPLLGHVLTLGKN
PHLALSRMSQRYGDVLQIRIGSTPVLVLSRLDTIRQALVRQGDDFKGRPDLYTSTLITDG
QSLTFSTDSGPVWAARRRLAQNALNTFSIASDPASSSSCYLEEHVSKEAKALISRLQELM
AGPGHFDPYNQVVVSVANVIGAMCFGQHFPESSDEMLSLVKNTHEFVETASSGNPLDFFP
ILRYLPNPALQRFKAFNQRFLWFLQKTVQEHYQDFDKNSVRDITGALFKHSKKGPRASGN
LIPQEKIVNLVNDIFGAGFDTVTTAISWSLMYLVTKPEIQRKIQKELDTVIGRERRPRLS
DRPQLPYLEAFILETFRHSSFLPFTIPHSTTRDTTLNGFYIPKKCCVFVNQWQVNHDPEL
WEDPSEFRPERFLTADGTAINKPLSEKMMLFGMGKRRCIGEVLAKWEIFLFLAILLQQLE
FSVPPGVKVDLTPIYGLTMKHARCEHVQARLRFSIN
>hsa:1545
MGTSLSPNDPWPLNPLSIQQTTLLLLLSVLATVHVGQRLLRQRRRQLRSAPPGPFAWPLI
GNAAAVGQAAHLSFARLARRYGDVFQIRLGSCPIVVLNGERAIHQALVQQGSAFADRPAF
ASFRVVSGGRSMAFGHYSEHWKVQRRAAHSMMRNFFTRQPRSRQVLEGHVLSEARELVAL
LVRGSADGAFLDPRPLTVVAVANVMSAVCFGCRYSHDDPEFRELLSHNEEFGRTVGAGSL
VDVMPWLQYFPNPVRTVFREFEQLNRNFSNFILDKFLRHCESLRPGAAPRDMMDAFILSA
EKKAAGDSHGGGARLDLENVPATITDIFGASQDTLSTALQWLLLLFTRYPDVQTRVQAEL
DQVVGRDRLPCMGDQPNLPYVLAFLYEAMRFSSFVPVTIPHATTANTSVLGYHIPKDTVV
FVNQWSVNHDPLKWPNPENFDPARFLDKDGLINKDLTSRVMIFSVGKRRCIGEELSKMQL
FLFISILAHQCDFRANPNEPAKMNFSYGLTIKPKSFKVNVTLRESMELLDSAVQNLQAKE
TCQ
>hsa:1548
MLASGMLLVALLVCLTVMVLMSVWQQRKSKGKLPPGPTPLPFIGNYLQLNTEQMYNSLMK
ISERYGPVFTIHLGPRRVVVLCGHDAVREALVDQAEEFSGRGEQATFDWVFKGYGVVFSN
GERAKQLRRFSIATLRDFGVGKRGIEERIQEEAGFLIDALRGTGGANIDPTFFLSRTVSN
VISSIVFGDRFDYKDKEFLSLLRMMLGIFQFTSTSTGQLYEMFSSVMKHLPGPQQQAFQL
LQGLEDFIAKKVEHNQRTLDPNSPRDFIDSFLIRMQEEEKNPNTEFYLKNLVMTTLNLFI
GGTETVSTTLRYGFLLLMKHPEVEAKVHEEIDRVIGKNRQPKFEDRAKMPYMEAVIHEIQ
RFGDVIPMSLARRVKKDTKFRDFFLPKGTEVYPMLGSVLRDPSFFSNPQDFNPQHFLNEK
GQFKKSDAFVPFSIGKRNCFGEGLARMELFLFFTTVMQNFRLKSSQSPKDIDVSPKHVGF
ATIPRNYTMSFLPR
>hsa:1549
MLASGLLLVALLACLTVMVLMSVWQQRKSRGKLPPGPTPLPFIGNYLQLNTEHICDSIMK
FSECYGPVFTIHLGPRRVVVLCGHDAVREALVDQAEEFSGRGEQATFDWVFKGYGVAFSN
GERAKQLLRFAIATLRDFGVGKRGIEERIQEESGFLIEAIRSTHGANIDPTFFLSRTVSN
VISSIVFGDRFDYEDKEFLSLLSMMLGIFQFTSTSTGQLYEMFSSVMKHLPGPQQQAFKL
LQGLEDFIAKKVEHNQRTLDPNSPQDFIDSFLIHMQEEEKNPNTEFYLKNLMMSTLNLFI
AGTETVSTTLRYGFLLLMKHPEVEAKVHEEIDRVIGKNRQPKFEDRTKMPYMEAVIHEIQ
RFGDVIPMSLARRVKKDTKFRDFFLPKGTEVFPMLGSVLRDPSFFSNPQDFNPQHFLDDK
GQFKKSDAFVPFSIGKRNCFGEGLARMELFLFFTTVMQNFRLKSSQSPKDIDVSPKHVVF
ATIPRNYTMSFLPR

>hsa:1551
MDLIPNLAVETWLLLAVSLILLYLYGTRTHGLFKKLGIPGPTPLPFLGNALSFRKGYWTF
DMECYKKYRKVWGIYDCQQPMLAITDPDMIKTVLVKECYSVFTNRRPFGPVGFMKNAISI
AEDEEWKRIRSLLSPTFTSGKLKEMVPIIAQYGDVLVRNLRREAETGKPVTLKHVFGAYS
MDVITSTSFGVSIDSLNNPQDPFVENTKKLLRFNPLDPFVLSIKVFPFLTPILEALNITV
FPRKVISFLTKSVKQIKEGRLKETQKHRVDFLQLMIDSQNSKDSETHKALSDLELMAQSI
IFIFAGYETTSSVLSFIIYELATHPDVQQKVQKEIDTVLPNKAPPTYDTVLQLEYLDMVV
NETLRLFPVAMRLERVCKKDVEINGMFIPKGVVVMIPSYVLHHDPKYWREPEKFLPERFS
KKNKDNIDPYIYTPFGSGPRNCIGMRFALVNMKLALVRVLQNFSFKPCKETQIPLKLRFG
GLLLTEKPIVLKAESRDETVSGA
>hsa:1553
MLASGLLLVTLLACLTVMVLMSVWRQRKSRGKLPPGPTPLPFIGNYLQLNTEQMYNSLMK
ISERYGPVFTIHLGPRRVVVLCGHDAVKEALVDQAEEFSGRGEQATFDWLFKGYGVAFSN
GERAKQLRRFSIATLRGFGVGKRGIEERIQEEAGFLIDALRGTHGANIDPTFFLSRTVSN
VISSIVFGDRFDYEDKEFLSLLRMMLGSFQFTATSTGQLYEMFSSVMKHLPGPQQQAFKE
LQGLEDFIAKKVEHNQRTLDPNSPRDFIDSFLIRMQEEEKNPNTEFYLKNLVMTTLNLFF
AGTETVSTTLRYGFLLLMKHPEVEAKVHEEIDRVIGKNRQPKFEDRAKMPYTEAVIHEIQ
RFGDMLPMGLAHRVNKDTKFRDFFLPKGTEVFPMLGSVLRDPRFFSNPRDFNPQHFLDKK
GQFKKSDAFVPFSIGKRYCFGEGLARMELFLFFTTIMQNFRFKSPQSPKDIDVSPKHVGF
ATIPRNYTMSFLPR
>hsa:1555
MELSVLLFLALLTGLLLLLVQRHPNTHDRLPPGPRPLPLLGNLLQMDRRGLLKSFLRFRE
KYGDVFTVHLGPRPVVMLCGVEAIREALVDKAEAFSGRGKIAMVDPFFRGYGVIFANGNR
WKVLRRFSVTTMRDFGMGKRSVEERIQEEAQCLIEELRKSKGALMDPTFLFQSITANIIC
SIVFGKRFHYQDQEFLKMLNLFYQTFSLISSVFGQLFELFSGFLKYFPGAHRQVYKNLQE
INAYIGHSVEKHRETLDPSAPKDLIDTYLLHMEKEKSNAHSEFSHQNLNLNTLSLFFAGT
ETTSTTLRYGFLLMLKYPHVAERVYREIEQVIGPHRPPELHDRAKMPYTEAVIYEIQRFS
DLLPMGVPHIVTQHTSFRGYIIPKDTEVFLILSTALHDPHYFEKPDAFNPDHFLDANGAL
KKTEAFIPFSLGKRICLGEGIARAELFLFFTTILQNFSMASPVAPEDIDLTPQECGVGKI
PPTYQIRFLPR
>hsa:1557
MDPFVVLVLCLSCLLLLSIWRQSSGRGKLPPGPTPLPVIGNILQIDIKDVSKSLTNLSKI
YGPVFTLYFGLERMVVLHGYEVVKEALIDLGEEFSGRGHFPLAERANRGFGIVFSNGKRW
KEIRRFSLMTLRNFGMGKRSIEDRVQEEARCLVEELRKTKASPCDPTFILGCAPCNVICS
IIFQKRFDYKDQQFLNLMEKLNENIRIVSTPWIQICNNFPTIIDYFPGTHNKLLKNLAFM
ESDILEKVKEHQESMDINNPRDFIDCFLIKMEKEKQNQQSEFTIENLVITAADLLGAGTE
TTSTTLRYALLLLLKHPEVTAKVQEEIERVIGRNRSPCMQDRGHMPYTDAVVHEVQRYID
LIPTSLPHAVTCDVKFRNYLIPKGTTILTSLTSVLHDNKEFPNPEMFDPRHFLDEGGNFK
KSNYFMPFSAGKRICVGEGLARMELFLFLTFILQNFNLKSLIDPKDLDTTPVVNGFASVP
PFYQLCFIPV
>hsa:1558
MEPFVVLVLCLSFMLLFSLWRQSCRRRKLPPGPTPLPIIGNMLQIDVKDICKSFTNFSKV
YGPVFTVYFGMNPIVVFHGYEAVKEALIDNGEEFSGRGNSPISQRITKGLGIISSNGKRW
KEIRRFSLTTLRNFGMGKRSIEDRVQEEAHCLVEELRKTKASPCDPTFILGCAPCNVICS

VVFQKRFDYKDQNFLTLMKRFNENFRILNSPWIQVCNNFPLLIDCFPGTHNKVLKNVALT
RSYIREKVKEHQASLDVNNPRDFIDCFLIKMEQEKDNQKSEFNIENLVGTVADLFVAGTE
TTSTTLRYGLLLLLKHPEVTAKVQEEIDHVIGRHRSPCMQDRSHMPYTDAVVHEIQRYSD
LVPTGVPHAVTTDTKFRNYLIPKGTTIMALLTSVLHDDKEFPNPNIFDPGHFLDKNGNFK
KSDYFMPFSAGKRICAGEGLARMELFLFLTTILQNFNLKSVDDLKNLNTTAVTKGIVSLP
PSYQICFIPV
>hsa:1559
MDSLVVLVLCLSCLLLLSLWRQSSGRGKLPPGPTPLPVIGNILQIGIKDISKSLTNLSKV
YGPVFTLYFGLKPIVVLHGYEAVKEALIDLGEEFSGRGIFPLAERANRGFGIVFSNGKKW
KEIRRFSLMTLRNFGMGKRSIEDRVQEEARCLVEELRKTKASPCDPTFILGCAPCNVICS
IIFHKRFDYKDQQFLNLMEKLNENIKILSSPWIQICNNFSPIIDYFPGTHNKLLKNVAFM
KSYILEKVKEHQESMDMNNPQDFIDCFLMKMEKEKHNQPSEFTIESLENTAVDLFGAGTE
TTSTTLRYALLLLLKHPEVTAKVQEEIERVIGRNRSPCMQDRSHMPYTDAVVHEVQRYID
LLPTSLPHAVTCDIKFRNYLIPKGTTILISLTSVLHDNKEFPNPEMFDPHHFLDEGGNFK
KSKYFMPFSAGKRICVGEALAGMELFLFLTSILQNFNLKSLVDPKNLDTTPVVNGFASVP
PFYQLCFIPV
>hsa:1562
MDPAVALVLCLSCLFLLSLWRQSSGRGRLPSGPTPLPIIGNILQLDVKDMSKSLTNFSKV
YGPVFTVYFGLKPIVVLHGYEAVKEALIDHGEEFSGRGSFPVAEKVNKGLGILFSNGKRW
KEIRRFCLMTLRNFGMGKRSIEDRVQEEARCLVEELRKTNASPCDPTFILGCAPCNVICS
VIFHDRFDYKDQRFLNLMEKFNENLRILSSPWIQVCNNFPALIDYLPGSHNKIAENFAYI
KSYVLERIKEHQESLDMNSARDFIDCFLIKMEQEKHNQQSEFTVESLIATVTDMFGAGTE
TTSTTLRYGLLLLLKYPEVTAKVQEEIECVVGRNRSPCMQDRSHMPYTDAVVHEIQRYID
LLPTNLPHAVTCDVKFKNYLIPKGTTIITSLTSVLHNDKEFPNPEMFDPGHFLDKSGNFK
KSDYFMPFSAGKRMCMGEGLARMELFLFLTTILQNFNLKSQVDPKDIDITPIANAFGRVP
PLYQLCFIPV
>hsa:1565
MGLEALVPLAVIVAIFLLLVDLMHRRQRWAARYPPGPLPLPGLGNLLHVDFQNTPYCFDQ
LRRRFGDVFSLQLAWTPVVVLNGLAAVREALVTHGEDTADRPPVPITQILGFGPRSQGVF
LARYGPAWREQRRFSVSTLRNLGLGKKSLEQWVTEEAACLCAAFANHSGRPFRPNGLLDK
AVSNVIASLTCGRRFEYDDPRFLRLLDLAQEGLKEESGFLREVLNAVPVLLHIPALAGKV
LRFQKAFLTQLDELLTEHRMTWDPAQPPRDLTEAFLAEMEKAKGNPESSFNDENLRIVVA
DLFSAGMVTTSTTLAWGLLLMILHPDVQRRVQQEIDDVIGQVRRPEMGDQAHMPYTTAVI
HEVQRFGDIVPLGVTHMTSRDIEVQGFRIPKGTTLITNLSSVLKDEAVWEKPFRFHPEHF
LDAQGHFVKPEAFLPFSAGRRACLGEPLARMELFLFFTSLLQHFSFSVPTGQPRPSHHGV
FAFLVSPSPYELCAVPR
>hsa:1571
MSALGVTVALLVWAAFLLLVSMWRQVHSSWNLPPGPFPLPIIGNLFQLELKNIPKSFTRL
AQRFGPVFTLYVGSQRMVVMHGYKAVKEALLDYKDEFSGRGDLPAFHAHRDRGIIFNNGP
TWKDIRRFSLTTLRNYGMGKQGNESRIQREAHFLLEALRKTQGQPFDPTFLIGCAPCNVI
ADILFRKHFDYNDEKFLRLMYLFNENFHLLSTPWLQLYNNFPSFLHYLPGSHRKVIKNVA
EVKEYVSERVKEHHQSLDPNCPRDLTDCLLVEMEKEKHSAERLYTMDGITVTVADLFFAG
TETTSTTLRYGLLILMKYPEIEEKLHEEIDRVIGPSRIPAIKDRQEMPYMDAVVHEIQRF
ITLVPSNLPHEATRDTIFRGYLIPKGTVVVPTLDSVLYDNQEFPDPEKFKPEHFLNENGK

FKYSDYFKPFSTGKRVCAGEGLARMELFLLLCAILQHFNLKPLVDPKDIDLSPIHIGFGC
IPPRYKLCVIPRS
>hsa:1572
MDSISTAILLLLLALVCLLLTLSSRDKGKLPPGPRPLSILGNLLLLCSQDMLTSLTKLSK
EYGSMYTVHLGPRRVVVLSGYQAVKEALVDQGEEFSGRGDYPAFFNFTKGNGIAFSSGDR
WKVLRQFSIQILRNFGMGKRSIEERILEEGSFLLAELRKTEGEPFDPTFVLSRSVSNIIC
SVLFGSRFDYDDERLLTIIRLINDNFQIMSSPWGELYDIFPSLLDWVPGPHQRIFQNFKC
LRDLIAHSVHDHQASLDPRSPRDFIQCFLTKMAEEKEDPLSHFHMDTLLMTTHNLLFGGT
KTVSTTLHHAFLALMKYPKVQARVQEEIDLVVGRARLPALKDRAAMPYTDAVIHEVQRFA
DIIPMNLPHRVTRDTAFRGFLIPKGTDVITLLNTVHYDPSQFLTPQEFNPEHFLDANQSF
KKSPAFMPFSAGRRLCLGESLARMELFLYLTAILQSFSLQPLGAPEDIDLTPLSSGLGNL
PRPFQLCLRPR
>hsa:1573
MLAAMGSLAAALWAVVHPRTLLLGTVAFLLAADFLKRRRPKNYPPGPWRLPFLGNFFLVD
FEQSHLEVQLFVKKYGNLFSLELGDISAVLITGLPLIKEALIHMDQNFGNRPVTPMREHI
FKKNGLIMSSGQAWKEQRRFTLTALRNFGLGKKSLEERIQEEAQHLTEAIKEENGQPFDP
HFKINNAVSNIICSITFGERFEYQDSWFQQLLKLLDEVTYLEASKTCQLYNVFPWIMKFL
PGPHQTLFSNWKKLKLFVSHMIDKHRKDWNPAETRDFIDAYLKEMSKHTGNPTSSFHEEN
LICSTLDLFFAGTETTSTTLRWALLYMALYPEIQEKVQAEIDRVIGQGQQPSTAARESMP
YTNAVIHEVQRMGNIIPLNVPREVTVDTTLAGYHLPKGTMILTNLTALHRDPTEWATPDT
FNPDHFLENGQFKKREAFMPFSIGKRACLGEQLARTELFIFFTSLMQKFTFRPPNNEKLS
LKFRMGITISPVSHRLCAVPQV
>hsa:1576
MALIPDLAMETWLLLAVSLVLLYLYGTHSHGLFKKLGIPGPTPLPFLGNILSYHKGFCMF
DMECHKKYGKVWGFYDGQQPVLAITDPDMIKTVLVKECYSVFTNRRPFGPVGFMKSAISI
AEDEEWKRLRSLLSPTFTSGKLKEMVPIIAQYGDVLVRNLRREAETGKPVTLKDVFGAYS
MDVITSTSFGVNIDSLNNPQDPFVENTKKLLRFDFLDPFFLSIIFPFLIPILEVLNICVF
PREVTNFLRKSVKRMKESRLEDTQKHRVDFLQLMIDSQNSKETESHKALSDLELVAQSII
FIFAGYETTSSVLSFIMYELATHPDVQQKLQEEIDAVLPNKAPPTYDTVLQMEYLDMVVN
ETLRLFPIAMRLERVCKKDVEINGMFIPKGVVVMIPSYALHRDPKYWTEPEKFLPERFSK
KNKDNIDPYIYTPFGSGPRNCIGMRFALMNMKLALIRVLQNFSFKPCKETQIPLKLSLGG
LLQPEKPVVLKVESRDGTVSGA
>hsa:1577
MDLIPNLAVETWLLLAVSLVLLYLYGTRTHGLFKRLGIPGPTPLPLLGNVLSYRQGLWKF
DTECYKKYGKMWGTYEGQLPVLAITDPDVIRTVLVKECYSVFTNRRSLGPVGFMKSAISL
AEDEEWKRIRSLLSPTFTSGKLKEMFPIIAQYGDVLVRNLRREAEKGKPVTLKDIFGAYS
MDVITGTSFGVNIDSLNNPQDPFVESTKKFLKFGFLDPLFLSIILFPFLTPVFEALNVSL
FPKDTINFLSKSVNRMKKSRLNDKQKHRLDFLQLMIDSQNSKETESHKALSDLELAAQSI
IFIFAGYETTSSVLSFTLYELATHPDVQQKLQKEIDAVLPNKAPPTYDAVVQMEYLDMVV
NETLRLFPVAIRLERTCKKDVEINGVFIPKGSMVVIPTYALHHDPKYWTEPEEFRPERFS
KKKDSIDPYIYTPFGTGPRNCIGMRFALMNMKLALIRVLQNFSFKPCKETQIPLKLDTQG
LLQPEKPIVLKVDSRDGTLSGE
>hsa:1579
MSVSVLSPSRLLGDVSGILQAASLLILLLLLIKAVQLYLHRQWLLKALQQFPCPPSHWLF

GHIQELQQDQELQRIQKWVETFPSACPHWLWGGKVRVQLYDPDYMKVILGRSDPKSHGSY
RFLAPWIGYGLLLLNGQTWFQHRRMLTPAFHYDILKPYVGLMADSVRVMLDKWEELLGQD
SPLEVFQHVSLMTLDTIMKCAFSHQGSIQVDRNSQSYIQAISDLNNLVFSRVRNAFHQND
TIYSLTSAGRWTHRACQLAHQHTDQVIQLRKAQLQKEGELEKIKRKRHLDFLDILLLAKM
ENGSILSDKDLRAEVDTFMFEGHDTTASGISWILYALATHPKHQERCREEIHSLLGDGAS
ITWNHLDQMPYTTMCIKEALRLYPPVPGIGRELSTPVTFPDGRSLPKGIMVLLSIYGLHH
NPKVWPNPEVFDPFRFAPGSAQHSHAFLPFSGGSRNCIGKQFAMNELKVATALTLLRFEL
LPDPTRIPIPIARLVLKSKNGIHLRLRRLPNPCEDKDQL
>hsa:1580
MVPSFLSLSFSSLGLWASGLILVLGFLKLIHLLLRRQTLAKAMDKFPGPPTHWLFGHALE
IQETGSLDKVVSWAHQFPYAHPLWFGQFIGFLNIYEPDYAKAVYSRGDPKAPDVYDFFLQ
WIGRGLLVLEGPKWLQHRKLLTPGFHYDVLKPYVAVFTESTRIMLDKWEEKAREGKSFDI
FCDVGHMALNTLMKCTFGRGDTGLGHRDSSYYLAVSDLTLLMQQRLVSFQYHNDFIYWLT
PHGRRFLRACQVAHDHTDQVIRERKAALQDEKVRKKIQNRRHLDFLDILLGARDEDDIKL
SDADLRAEVDTFMFEGHDTTTSGISWFLYCMALYPEHQHRCREEVREILGDQDFFQWDDL
GKMTYLTMCIKESFRLYPPVPQVYRQLSKPVTFVDGRSLPAGSLISMHIYALHRNSAVWP
DPEVFDSLRFSTENASKRHPFAFMPFSAGPRNCIGQQFAMSEMKVVTAMCLLRFEFSLDP
SRLPIKMPQLVLRSKNGFHLHLKPLGPGSGK
>hsa:1581
MMTTSLIWGIAIAACCCLWLILGIRRRQTGEPPLENGLIPYLGCALQFGANPLEFLRANQ
RKHGHVFTCKLMGKYVHFITNPLSYHKVLCHGKYFDWKKFHFATSAKAFGHRSIDPMDGN
TTENINDTFIKTLQGHALNSLTESMMENLQRIMRPPVSSNSKTAAWVTEGMYSFCYRVMF
EAGYLTIFGRDLTRRDTQKAHILNNLDNFKQFDKVFPALVAGLPIHMFRTAHNAREKLAE
SLRHENLQKRESISELISLRMFLNDTLSTFDDLEKAKTHLVVLWASQANTIPATFWSLFQ
MIRNPEAMKAATEEVKRTLENAGQKVSLEGNPICLSQAELNDLPVLDSIIKESLRLSSAS
LNIRTAKEDFTLHLEDGSYNIRKDDIIALYPQLMHLDPEIYPDPLTFKYDRYLDENGKTK
TTFYCNGLKLKYYYMPFGSGATICPGRLFAIHEIKQFLILMLSYFELELIEGQAKCPPLD
QSRAGLGILPPLNDIEFKYKFKHL
>hsa:1582
MVLWGPVLGALLVVIAGYLCLPGMLRQRRPWEPPLDKGTVPWLGHAMAFRKNMFEFLKRM
RTKHGDVFTVQLGGQYFTFVMDPLSFGSILKDTQRKLDFGQYAKKLVLKVFGYRSVQGDH
EMIHSASTKHLRGDGLKDLNETMLDSLSFVMLTSKGWSLDASCWHEDSLFRFCYYILFTA
GYLSLFGYTKDKEQDLLQAGELFMEFRKFDLLFPRFVYSLLWPREWLEVGRLQRLFHKML
SVSHSQEKEGISNWLGNMLQFLREQGVPSAMQDKFNFMMLWASQGNTGPTSFWALLYLLK
HPEAIRAVREEATQVLGEARLETKQSFAFKLGALQHTPVLDSVVEETLRLRAAPTLLRLV
HEDYTLKMSSGQEYLFRHGDILALFPYLSVHMDPDIHPEPTVFKYDRFLNPNGSRKVDFF
KTGKKIHHYTMPWGSGVSICPGRFFALSEVKLFILLMVTHFDLELVDPDTPLPHVDPQRW
GFGTMQPSHDVRFRYRLHPTE
>hsa:1583
MLAKGLPPRSVLVKGCQTFLSAPREGLGRLRVPTGEGAGISTRSPRPFNEIPSPGDNGWL
NLYHFWRETGTHKVHLHHVQNFQKYGPIYREKLGNVESVYVIDPEDVALLFKSEGPNPER
FLIPPWVAYHQYYQRPIGVLLKKSAAWKKDRVALNQEVMAPEATKNFLPLLDAVSRDFVS
VLHRRIKKAGSGNYSGDISDDLFRFAFESITNVIFGERQGMLEEVVNPEAQRFIDAIYQM
FHTSVPMLNLPPDLFRLFRTKTWKDHVAAWDVIFSKADIYTQNFYWELRQKGSVHHDYRG

ILYRLLGDSKMSFEDIKANVTEMLAGGVDTTSMTLQWHLYEMARNLKVQDMLRAEVLAAR
HQAQGDMATMLQLVPLLKASIKETLRLHPISVTLQRYLVNDLVLRDYMIPAKTLVQVAIY
ALGREPTFFFDPENFDPTRWLSKDKNITYFRNLGFGWGVRQCLGRRIAELEMTIFLINML
ENFRVEIQHLSDVGTTFNLILMPEKPISFTFWPFNQEATQQ

>hsa:1584

MALRAKAEVCMAVPWLSLQRAQALGTRAARVPRTVLPFEAMPRRPGNRWLRLLQIWREQG
YEDLHLEVHQTFQELGPIFRYDLGGAGMVCVMLPEDVEKLQQVDSLHPHRMSLEPWVAYR
QHRGHKCGVFLLNGPEWRFNRLRLNPEVLSPNAVQRFLPMVDAVARDFSQALKKKVLQNA
RGSLTLDVQPSIFHYTIEASNLALFGERLGLVGHSPSSASLNFLHALEVMFKSTVQLMFM
PRSLSRWTSPKVWKEHFEAWDCIFQYGDNCIQKIYQELAFSRPQQYTSIVAELLLNAELS
PDAIKANSMELTAGSVDTTVFPLLMTLFELARNPNVQQALRQESLAAAASISEHPQKATT
ELPLLRAALKETLRLYPVGLFLERVASSDLVLQNYHIPAGTLVRVFLYSLGRNPALFPRP
ERYNPQRWLDIRGSGRNFYHVPFGFGMRQCLGRRLAEAEMLLLLHHVLKHLQVETLTQED
IKMVYSFILRPSMFPLLTFRAIN

>hsa:1585

MALRAKAEVCVAAPWLSLQRARALGTRAARAPRTVLPFEAMPQHPGNRWLRLLQIWREQG
YEHLHLEMHQTFQELGPIFRYNLGGPRMVCVMLPEDVEKLQQVDSLHPCRMILEPWVAYR
QHRGHKCGVFLLNGPEWRFNRLRLNPDVLSPKAVQRFLPMVDAVARDFSQALKKKVLQNA
RGSLTLDVQPSIFHYTIEASNLALFGERLGLVGHSPSSASLNFLHALEVMFKSTVQLMFM
PRSLSRWISPKVWKEHFEAWDCIFQYGDNCIQKIYQELAFNRPQHYTGIVAELLLKAELS
LEAIKANSMELTAGSVDTTAFPLLMTLFELARNPDVQQILRQESLAAAASISEHPQKATT
ELPLLRAALKETLRLYPVGLFLERVVSSDLVLQNYHIPAGTLVQVFLYSLGRNAALFPRP
ERYNPQRWLDIRGSGRNFHHVPFGFGMRQCLGRRLAEAEMLLLLHHVLKHFLVETLTQED
IKMVYSFILRPGTSPLLTFRAIN

>hsa:1586

MWELVALLLLTLAYLFWPKRRCPGAKYPKSLLSLPLVGSLPFLPRHGHMHNNFFKLQKKY
GPIYSVRMGTKTTVIVGHHQLAKEVLIKKGKDFSGRPQMATLDIASNNRKGIAFADSGAH
WQLHRRLAMATFALFKDGDQKLEKIICQEISTLCDMLATHNGQSIDISFPVFVAVTNVIS
LICFNTSYKNGDPELNVIQNYNEGIIDNLSKDSLVDLVPWLKIFPNKTLEKLKSHVKIRN
DLLNKILENYKEKFRSDSITNMLDTLMQAKMNSDNGNAGPDQDSELLSDNHILTTIGDIF
GAGVETTTSVVKWTLAFLLHNPQVKKKLYEEIDQNVGFSRTPTISDRNRLLLLEATIREV
LRLRPVAPMLIPHKANVDSSIGEFAVDKGTEVIINLWALHHNEKEWHQPDQFMPERFLNP
AGTQLISPSVSYLPFGAGPRSCIGEILARQELFLIMAWLLQRFDLEVPDDGQLPSLEGIP
KVVFLIDSFKVKIKVRQAWREAQAEGST

>hsa:1588

MVLEMLNPIHYNITSIVPEAMPAATMPVLLLTGLFLLVWNYEGTSSIPGPGYCMGIGPLI
SHGRFLWMGIGSACNYYNRVYGEFMRVWISGEETLIISKSSSMFHIMKHNHYSSRFGSKL
GLQCIGMHEKGIIFNNNPELWKTTRPFFMKALSGPGLVRMVTVCAESLKTHLDRLEEVTN
ESGYVDVLTLLRRVMLDTSNTLFLRIPLDESAIVVKIQGYFDAWQALLIKPDIFFKISWL
YKKYEKSVKDLKDAIEVLIAEKRRRISTEEKLEECMDFATELILAEKRGDLTRENVNQCI
LEMLIAAPDTMSVSLFFMLFLIAKHPNVEEAIIKEIQTVIGERDIKIDDIQKLKVMENFI
YESMRYQPVVDLVMRKALEDDVIDGYPVKKGTNIILNIGRMHRLEFFPKPNEFTLENFAK
NVPYRYFQPFGFGPRGCAGKYIAMVMMKAILVTLLRRFHVKTLQGQCVESIQKIHDLSLH
PDETKNMLEMIFTPRNSDRCLEH

>hsa:1589
MLLLGLLLLLPLLAGARLLWNWWKLRSLHLPPLAPGFLHLLQPDLPIYLLGLTQKFGPIY
RLHLGLQDVVVLNSKRTIEEAMVKKWADFAGRPEPLTYKLVSRNYPDLSLGDYSLLWKAH
KKLTRSALLLGIRDSMEPVVEQLTQEFCERMRAQPGTPVAIEEEFSLLTCSIICYLTFGD
KIKDDNLMPAYYKCIQEVLKTWSHWSIQIVDVIPFLRFFPNPGLRRLKQAIEKRDHIVEM
QLRQHKESLVAGQWRDMMDYMLQGVAQPSMEEGSGQLLEGHVHMAAVDLLIGGTETTANT
LSWAVVFLLHHPEIQQRLQEELDHELGPGASSSRVPYKDRARLPLLNATIAEVLRLRPVV
PLALPHRTTRPSSISGYDIPEGTVIIPNLQGAHLDETVWERPHEFWPDRFLEPGKNSRAL
AFGCGARVCLGEPLARLELFVVLTRLLQAFTLLPSGDALPSLQPLPHCSVILKMQPFQVR
LQPRGMGAHSPGQSQ
>hsa:1593
MAALGCARLRWALRGAGRGLCPHGARAKAAIPAALPSDKATGAPGAGPGVRRRQRSLEEI
PRLGQLRFFFQLFVQGYALQLHQLQVLYKAKYGPMWMSYLGPQMHVNLASAPLLEQVMRQ
EGKYPVRNDMELWKEHRDQHDLTYGPFTTEGHHWYQLRQALNQRLLKPAEAALYTDAFNE
VIDDFMTRLDQLRAESASGNQVSDMAQLFYYFALEAICYILFEKRIGCLQRSIPEDTVTF
VRSIGLMFQNSLYATFLPKWTRPVLPFWKRYLDGWNAIFSFGKKLIDEKLEDMEAQLQAA
GPDGIQVSGYLHFLLASGQLSPREAMGSLPELLMAGVDTTSNTLTWALYHLSKDPEIQEA
LHEEVVGVVPAGQVPQHKDFAHMPLLKAVLKETLRLYPVVPTNSRIIEKEIEVDGFLFPK
NTQFVFCHYVVSRDPTAFSEPESFQPHRWLRNSQPATPRIQHPFGSVPFGYGVRACLGRR
IAELEMQLLLARLIQKYKVVLAPETGELKSVARIVLVPNKKVGLQFLQRQC
>hsa:1594
MTQTLKYASRVFHRVRWAPELGASLGYREYHSARRSLADIPGPSTPSFLAELFCKGGLSR
LHELQVQGAAHFGPVWLASFGTVRTVYVAAPALVEELLRQEGPRPERCSFSPWTEHRRCR
QRACGLLTAEGEEWQRLRSLLAPLLLRPQAAARYAGTLNNVVCDLVRRLRRQRGRGTGPP
ALVRDVAGEFYKFGLEGIAAVLLGSRLGCLEAQVPPDTETFIRAVGSVFVSTLLTMAMPH
WLRHLVPGPWGRLCRDWDQMFAFAQRHVERREAEAAMRNGGQPEKDLESGAHLTHFLFRE
ELPAQSILGNVTELLLAGVDTVSNTLSWALYELSRHPEVQTALHSEITAALSPGSSAYPS
ATVLSQLPLLKAVVKEVLRLYPVVPGNSRVPDKDIHVGDYIIPKNTLVTLCHYATSRDPA
QFPEPNSFRPARWLGEGPTPHPFASLPFGFGKRSCMGRRLAELELQMALAQILTHFEVQP
EPGAAPVRPKTRTVLVPERSINLQFLDR
>hsa:1595
MAAAAGMLLLGLLQAGGSVLGQAMEKVTGGNLLSMLLIACAFTLSLVYLIRLAAGHLVQL
PAGVKSPPYIFSPIPFLGHAIAFGKSPIEFLENAYEKYGPVFSFTMVGKTFTYLLGSDAA
ALLFNSKNEDLNAEDVYSRLTTPVFGKGVAYDVPNPVFLEQKKMLKSGLNIAHFKQHVSI
IEKETKEYFESWGESGEKNVFEALSELIILTASHCLHGKEIRSQLNEKVAQLYADLDGGF
SHAAWLLPGWLPLPSFRRRDRAHREIKDIFYKAIQKRRQSQEKIDDILQTLLDATYKDGR
PLTDDEVAGMLIGLLLAGQHTSSTTSAWMGFFLARDKTLQKKCYLEQKTVCGENLPPLTY
DQLKDLNLLDRCIKETLRLRPPIMIMMRMARTPQTVAGYTIPPGHQVCVSPTVNQRLKDS
WVERLDFNPDRYLQDNPASGEKFAYVPFGAGRHRCIGENFAYVQIKTIWSTMLRLYEFDL
IDGYFPTVNYTTMIHTPENPVIRYKRRSK
>hsa:1610
MRVVVIGAGVIGLSTALCIHERYHSVLQPLDIKVYADRFTPLTTTDVAAGLWQPYLSDPN
NPQEADWSQQTFDYLLSHVHSPNAENLGLFLISGYNLFHEAIPDPSWKDTVLGFRKLTPR
ELDMFPDYGYGWFHTSLILEGKNYLQWLTERLTERGVKFFQRKVESFEEVAREGADVIVN

CTGVWAGALQRDPLLQPGRGQIMKVDAPWMKHFILTHDPERGIYNSPYIIPGTQTVTLGG
IFQLGNWSELNNIQDHNTIWEGCCRLEPTLKNARIIGERTGFRPVRPQIRLEREQLRTGP
SNTEVIHNYGHGGYGLTIHWGCALEAAKLFGRILEEKKLSRMPPSHL
>hsa:1621
MPALSRWASLPGPSMREAAFMYSTAVAIFLVILVAALQGSAPRESPLPYHIPLDPEGSLE
LSWNVSYTQEAIHFQLLVRRLKAGVLFGMSDRGELENADLVVLWTDGDTAYFADAWSDQK
GQIHLDPQQDYQLLQVQRTPEGLTLLFKRPFGTCDPKDYLIEDGTVHLVYGILEEPFRSL
EAINGSGLQMGLQRVQLLKPNIPEPELPSDACTMEVQAPNIQIPSQETTYWCYIKELPKG
FSRHHIIKYEPIVTKGNEALVHHMEVFQCAPEMDSVPHFSGPCDSKMKPDRLNYCRHVLA
AWALGAKAFYYPEEAGLAFGGPGSSRYLRLEVHYHNPLVIEGRNDSSGIRLYYTAKLRRF
NAGIMELGLVYTPVMAIPPRETAFILTGYCTDKCTQLALPPSGIHIFASQLHTHLTGRKV
VTVLVRDGREWEIVNQDNHYSPHFQEIRMLKKVVSVHPGDVLITSCTYNTEDRELATVGG
FGILEEMCVNYVHYYPQTQLELCKSAVDAGFLQKYFHLINRFNNEDVCTCPQASVSQQFT
SVPWNSFNRDVLKALYSFAPISMHCNKSSAVRFQGEWNLQPLPKVISTLEEPTPQCPTSQ
GRSPAGPTVVSIGGGKG
>hsa:1633
MATPPKRSCPSFSASSEGTRIKKISIEGNIAAGKSTFVNILKQLCEDWEVVPEPVARWCN
VQSTQDEFEELTMSQKNGGNVLQMMYEKPERWSFTFQTYACLSRIRAQLASLNGKLKDAE
KPVLFFERSVYSDRYIFASNLYESECMNETEWTIYQDWHDWMNNQFGQSLELDGIIYLQA
TPETCLHRIYLRGRNEEQGIPLEYLEKLHYKHESWLLHRTLKTNFDYLQEVPILTLDVNE
DFKDKYESLVEKVKEFLSTL
>hsa:1636
MGAASGRRGPGLLLPLPLLLLLPPQPALALDPGLQPGNFSADEAGAQLFAQSYNSSAEQV
LFQSVAASWAHDTNITAENARRQEEAALLSQEFAEAWGQKAKELYEPIWQNFTDPQLRRI
IGAVRTLGSANLPLAKRQQYNALLSNMSRIYSTAKVCLPNKTATCWSLDPDLTNILASSR
SYAMLLFAWEGWHNAAGIPLKPLYEDFTALSNEAYKQDGFTDTGAYWRSWYNSPTFEDDL
EHLYQQLEPLYLNLHAFVRRALHRRYGDRYINLRGPIPAHLLGDMWAQSWENIYDMVVPF
PDKPNLDVTSTMLQQGWNATHMFRVAEEFFTSLELSPMPPEFWEGSMLEKPADGREVVCH
ASAWDFYNRKDFRIKQCTRVTMDQLSTVHHEMGHIQYYLQYKDLPVSLRRGANPGFHEAI
GDVLALSVSTPEHLHKIGLLDRVTNDTESDINYLLKMALEKIAFLPFGYLVDQWRWGVFS
GRTPPSRYNFDWWYLRTKYQGICPPVTRNETHFDAGAKFHVPNVTPYIRYFVSFVLQFQF
HEALCKEAGYEGPLHQCDIYRSTKAGAKLRKVLQAGSSRPWQEVLKDMVGLDALDAQPLL
KYFQPVTQWLQEQNQQNGEVLGWPEYQWHPPLPDNYPEGIDLVTDEAEASKFVEEYDRTS
QVVWNEYAEANWNYNTNITTETSKILLQKNMQIANHTLKYGTQARKFDVNQLQNTTIKRI
IKKVQDLERAALPAQELEEYNKILLDMETTYSVATVCHPNGSCLQLEPDLTNVMATSRKY
EDLLWAWEGWRDKAGRAILQFYPKYVELINQAARLNGYVDAGDSWRSMYETPSLEQDLER
LFQELQPLYLNLHAYVRRALHRHYGAQHINLEGPIPAHLLGNMWAQTWSNIYDLVVPFPS
APSMDTTEAMLKQGWTPRRMFKEADDFFTSLGLLPVPPEFWNKSMLEKPTDGREVVCHAS
AWDFYNGKDFRIKQCTTVNLEDLVVAHHEMGHIQYFMQYKDLPVALREGANPGFHEAIGD
VLALSVSTPKHLHSLNLLSSEGGSDEHDINFLMKMALDKIAFIPFSYLVDQWRWRVFDGS
ITKENYNQEWWSLRLKYQGLCPPVPRTQGDFDPGAKFHIPSSVPYIRYFVSFIIQFQFHE
ALCQAAGHTGPLHKCDIYQSKEAGQRLATAMKLGFSRPWPEAMQLITGQPNMSASAMLSY
FKPLLDWLRTENELHGEKLGWPQYNWTPNSARSEGPLPDSGRVSFLGLDLDAQQARVGQW
LLLFLGIALLVATLGLSQRLFSIRHRSLHRHSHGPQFGSEVELRHS

>hsa:1645
MDSKYQCVKLNDGHFMPVLGFGTYAPAEVPKSKALEATKLAIEAGFRHIDSAHLYNNEEQ
VGLAIRSKIADGSVKREDIFYTSKLWCNSHRPELVRPALERSLKNLQLDYVDLYLIHFPV
SVKPGEEVIPKDENGKILFDTVDLCATWEAVEKCKDAGLAKSIGVSNFNRRQLEMILNKP
GLKYKPVCNQVECHPYFNQRKLLDFCKSKDIVLVAYSALGSHREEPWVDPNSPVLLEDPV
LCALAKKHKRTPALIALRYQLQRGVVVLAKSYNEQRIRQNVQVFEFQLTSEEMKAIDGLN
RNVRYLTLDIFAGPPNYPFSDEY
>hsa:1675
MHSWERLAVLVLLGAAACAAPPRGRILGGREAEAHARPYMASVQLNGAHLCGGVLVAEQW
VLSAAHCLEDAADGKVQVLLGAHSLSQPEPSKRLYDVLRAVPHPDSQPDTIDHDLLLLQL
SEKATLGPAVRPLPWQRVDRDVAPGTLCDVAGWGIVNHAGRRPDSLQHVLLPVLDRATCN
RRTHHDGAITERLMCAESNRRDSCKGDSGGPLVCGGVLEGVVTSGSRVCGNRKKPGIYTR
VASYAAWIDSVLA
>hsa:1719
MVGSLNCIVAVSQNMGIGKNGDLPWPPLRNEFRYFQRMTTTSSVEGKQNLVIMGKKTWFS
IPEKNRPLKGRINLVLSRELKEPPQGAHFLSRSLDDALKLTEQPELANKVDMVWIVGGSS
VYKEAMNHPGHLKLFVTRIMQDFESDTFFPEIDLEKYKLLPEYPGVLSDVQEEKGIKYKF
EVYEKND
>hsa:1723
MAWRHLKKRAQDAVIILGGGGLLFASYLMATGDERFYAEHLMPTLQGLLDPESAHRLAVR
FTSLGLLPRARFQDSDMLEVRVLGHKFRNPVGIAAGFDKHGEAVDGLYKMGFGFVEIGSV
TPKPQEGNPRPRVFRLPEDQAVINRYGFNSHGLSVVEHRLRARQQKQAKLTEDGLPLGVN
LGKNKTSVDAAEDYAEGVRVLGPLADYLVVNVSSPNTAGLRSLQGKAELRRLLTKVLQER
DGLRRVHRPAVLVKIAPDLTSQDKEDIASVVKELGIDGLIVTNTTVSRPAGLQGALRSET
GGLSGKPLRDLSTQTIREMYALTQGRVPIIGVGGVSSGQDALEKIRAGASLVQLYTALTF
WGPPVVGKVKRELEALLKEQGFGGVTDAIGADHRR
>hsa:1725
MPIIPAFWEAEAGGSREEEFETSLANMIEKKLEPLSQDEDQHADLTQSRRPLTSCTIFLG
YTSNLISSGIRETIRYLVQHNMVDVLVTTAGGVEEDLIKCLAPTYLGEFSLRGKELRENG
INRIGNLLVPNENYCKFEDWLMPILDQMVMEQNTEGVKWTPSKMIARLGKEINNPESVYY
WAQKNHIPVFSPALTDGSLGDMIFFHSYKNPGLVLDIVEDLRLINTQAIFAKCTGMIILG
GGVVKHHIANANLMRNGADYAVYINTAQEFDGSDSGARPDEAVSWGKIRVDAQPVKVYAD
ASLVFPLLVAETFAQKMDAFMHEKNED
>hsa:1728
MVGRRALIVLAHSERTSFNYAMKEAAAAALKKKGWEVVESDLYAMNFNPIISRKDITGKL
KDPANFQYPAESVLAYKEGHLSPDIVAEQKKLEAADLVIFQFPLQWFGVPAILKGWFERV
FIGEFAYTYAAMYDKGPFRSKKAVLSITTGGSGSMYSLQGIHGDMNVILWPIQSGILHFC
GFQVLEPQLTYSIGHTPADARIQILEGWKKRLENIWDETPLYFAPSSLFDLNFQAGFLMK
KEVQDEEKNKKFGLSVGHHLGKSIPTDNQIKARK
>hsa:1800
MWSGWWLWPLVAVCTADFFRDEAERIMRDSPVIDGHNDLPWQLLDMFNNRLQDERANLTT
LAGTHTNIPKLRAGFVGGQFWSVYTPCDTQNKDAVRRTLEQMDVVHRMCRMYPETFLYVT
SSAGIRQAFREGKVASLIGVEGGHSIDSSLGVLRALYQLGMRYLTLTHSCNTPWADNWLV
DTGDSEPQSQGLSPFGQRVVKELNRLGVLIDLAHVSVATMKATLQLSRAPVIFSHSSAYS

VCASRRNVPDDVLRLVKQTDSLVMVNFYNNYISCTNKANLSQVADHLDHIKEVAGARAVG
FGGDFDGVPRVPEGLEDVSKYPDLIAELLRRNWTEAEVKGALADNLLRVFEAVEQASNLT
QAPEEEPIPLDQLGGSCRTHYGYSSGASSLHRHWGLLLASLAPLVLCLSLL
>hsa:1803
MKTPWKVLLGLLGAAALVTIITVPVVLLNKGTDDATADSRKTYTLTDYLKNTYRLKLYSL
RWISDHEYLYKQENNILVFNAEYGNSSVFLENSTFDEFGHSINDYSISPDGQFILLEYNY
VKQWRHSYTASYDIYDLNKRQLITEERIPNNTQWVTWSPVGHKLAYVWNNDIYVKIEPNL
PSYRITWTGKEDIIYNGITDWVYEEEVFSAYSALWWSPNGTFLAYAQFNDTEVPLIEYSF
YSDESLQYPKTVRVPYPKAGAVNPTVKFFVVNTDSLSSVTNATSIQITAPASMLIGDHYL
CDVTWATQERISLQWLRRIQNYSVMDICDYDESSGRWNCLVARQHIEMSTTGWVGRFRPS
EPHFTLDGNSFYKIISNEEGYRHICYFQIDKKDCTFITKGTWEVIGIEALTSDYLYYISN
EYKGMPGGRNLYKIQLSDYTKVTCLSCELNPERCQYYSVSFSKEAKYYQLRCSGPGLPLY
TLHSSVNDKGLRVLEDNSALDKMLQNVQMPSKKLDFIILNETKFWYQMILPPHFDKSKKY
PLLLDVYAGPCSQKADTVFRLNWATYLASTENIIVASFDGRGSGYQGDKIMHAINRRLGT
FEVEDQIEAARQFSKMGFVDNKRIAIWGWSYGGYVTSMVLGSGSGVFKCGIAVAPVSRWE
YYDSVYTERYMGLPTPEDNLDHYRNSTVMSRAENFKQVEYLLIHGTADDNVHFQQSAQIS
KALVDVGVDFQAMWYTDEDHGIASSTAHQHIYTHMSHFIKQCFSLP
>hsa:1806
MAPVLSKDSADIESILALNPRTQTHATLCSTSAKKLDKKHWKRNPDKNCFNCEKLENNFD
DIKHTTLGERGALREAMRCLKCADAPCQKSCPTNLDIKSFITSIANKNYYGAAKMIFSDN
PLGLTCGMVCPTSDLCVGGCNLYATEEGPINIGGLQQFATEVFKAMSIPQIRNPSLPPPE
KMSEAYSAKIALFGAGPASISCASFLARLGYSDITIFEKQEYVGGLSTSEIPQFRLPYDV
VNFEIELMKDLGVKIICGKSLSVNEMTLSTLKEKGYKAAFIGIGLPEPNKDAIFQGLTQD
QGFYTSKDFLPLVAKGSKAGMCACHSPLPSIRGVVIVLGAGDTAFDCATSALRCGARRVF
IVFRKGFVNIRAVPEEMELAKEEKCEFLPFLSPRKVIVKGGRIVAMQFVRTEQDETGKWN
EDEDQMVHLKADVVISAFGSVLSDPKVKEALSPIKFNRWGLPEVDPETMQTSEAWVFAGG
DVVGLANTTVESVNDGKQASWYIHKYVQSQYGASVSAKPELPLFYTPIDLVDISVEMAGL
KFINPFGLASATPATSTSMIRRAFEAGWGFALTKTFSLDKDIVTNVSPRIIRGTTSGPMY
GPGQSSFLNIELISEKTAAYWCQSVTELKADFPDNIVIASIMCSYNKNDWTELAKKSEDS
GADALELNLSCPHGMGERGMGLACGQDPELVRNICRWVRQAVQIPFFAKLTPNVTDIVSI
ARAAKEGGANGVTATNTVSGLMGLKSDGTPWPAVGIAKRTTYGGVSGTAIRPIALRAVTS
IARALPGFPILATGGIDSAESGLQFLHSGASVLQVCSAIQNQDFTVIEDYCTGLKALLYL
KSIEELQDWDGQSPATVSHQKGKPVPRIAELMDKKLPSFGPYLEQRKKIIAENKIRLKEQ
NVAFSPLKRNCFIPKRPIPTIKDVIGKALQYLGTFGELSNVEQVVAMIDEEMCINCGKCY
MTCNDSGYQAIQFDPETHLPTITDTCTGCTLCLSVCPIVDCIKMVSRTTPYEPKRGVPLS
VNPVC
>hsa:1843
MVMEVGTLDAGGLRALLGERAAQCLLLDCRSFFAFNAGHIAGSVNVRFSTIVRRRAKGAM
GLEHIVPNAELRGRLLAGAYHAVVLLDERSAALDGAKRDGTLALAAGALCREARAAQVFF
LKGGYEAFSASCPELCSKQSTPMGLSLPLSTSVPDSAESGCSSCSTPLYDQGGPVEILPF
LYLGSAYHASRKDMLDALGITALINVSANCPNHFEGHYQYKSIPVEDNHKADISSWFNEA
IDFIDSIKNAGGRVFVHCQAGISRSATICLAYLMRTNRVKLDEAFEFVKQRRSIISPNFS
FMGQLLQFESQVLAPHCSAEAGSPAMAVLDRGTSTTTVFNFPVSIPVHSTNSALSYLQSP
ITTSPSC

>hsa:1844
MGLEAARELECAALGTLLRDPREAERTLLLDCRPFLAFCRRHVRAARPVPWNALLRRRAR
GPPAAVLACLLPDRALRTRLVRGELARAVVLDEGSASVAELRPDSPAHVLLAALLHETRA
GPTAVYFLRGGFDGFQGCCPDLCSEAPAPALPPTGDKTSRSDSRAPVYDQGGPVEILPYL
FLGSCSHSSDLQGLQACGITAVLNVSASCPNHFEGLFRYKSIPVEDNQMVEISAWFQEAI
GFIDWVKNSGGRVLVHCQAGISRSATICLAYLMQSRRVRLDEAFDFVKQRRGVISPNFSF
MGQLLQFETQVLCH
>hsa:1845
MSGSFELSVQDLNDLLSDGSGCYSLPSQPCNEVTPRIYVGNASVAQDIPKLQKLGITHVL
NAAEGRSFMHVNTNANFYKDSGITYLGIKANDTQEFNLSAYFERAADFIDQALAQKNGRV
LVHCREGYSRSPTLVIAYLMMRQKMDVKSALSIVRQNREIGPNDGFLAQLCQLNDRLAKE
GKLKP
>hsa:1846
MVTMEELREMDCSVLKRLMNRDENGGGAGGSGSHGTLGLPSGGKCLLLDCRPFLAHSAGY
ILGSVNVRCNTIVRRRAKGSVSLEQILPAEEEVRARLRSGLYSAVIVYDERSPRAESLRE
DSTVSLVVQALRRNAERTDICLLKGGYERFSSEYPEFCSKTKALAAIPPPVPPSATEPLD
LGCSSCGTPLHDQGGPVEILPFLYLGSAYHAARRDMLDALGITALLNVSSDCPNHFEGHY
QYKCIPVEDNHKADISSWFMEAIEYIDAVKDCRGRVLVHCQAGISRSATICLAYLMMKKR
VRLEEAFEFVKQRRSIISPNFSFMGQLLQFESQVLATSCAAEAASPSGPLRERGKTPATP
TSQFVFSFPVSVGVHSAPSSLPYLHSPITTSPSC
>hsa:1847
MKVTSLDGRQLRKMLRKEAAARCVVLDCRPYLAFAASNVRGSLNVNLNSVVLRRARGGAV
SARYVLPDEAARARLLQEGGGGVAAVVVLDQGSRHWQKLREESAARVVLTSLLACLPAGP
RVYFLKGGYETFYSEYPECCVDVKPISQEKIESERALISQCGKPVVNVSYRPAYDQGGPV
EILPFLYLGSAYHASKCEFLANLHITALLNVSRRTSEACATHLHYKWIPVEDSHTADISS
HFQEAIDFIDCVREKGGKVLVHCEAGISRSPTICMAYLMKTKQFRLKEAFDYIKQRRSMV
SPNFGFMGQLLQYESEILPSTPNPQPPSCQGEAAGSSLIGHLQTLSPDMQGAYCTFPASV
LAPVPTHSTVSELSRSPVATATSC
>hsa:1848
MIDTLRPVPFASEMAISKTVAWLNEQLELGNERLLLMDCRPQELYESSHIESAINVAIPG
IMLRRLQKGNLPVRALFTRGEDRDRFTRRCGTDTVVLYDESSSDWNENTGGESVLGLLLK
KLKDEGCRAFYLEGGFSKFQAEFSLHCETNLDGSCSSSSPPLPVLGLGGLRISSDSSSDI
ESDLDRDPNSATDSDGSPLSNSQPSFPVEILPFLYLGCAKDSTNLDVLEEFGIKYILNVT
PNLPNLFENAGEFKYKQIPISDHWSQNLSQFFPEAISFIDEARGKNCGVLVHCLAGISRS
VTVTVAYLMQKLNLSMNDAYDIVKMKKSNISPNFNFMGQLLDFERTLGLSSPCDNRVPAQ
QLYFTTPSNQNVYQVDSLQST
>hsa:1849
MKNQLRGPPARAHMSTSGAAAAGGTRAGSEPGAGSGSGAGTGAGAATGAGAMPCKSAEWL
QEELEARGGASLLLLDCRPHELFESSHIETAINLAIPGLMLRRLRKGNLPIRSIIPNHAD
KERFATRCKAATVLLYDEATAEWQPEPGAPASVLGLLLQKLRDDGCQAYYLQGGFNKFQT
EYSEHCETNVDSSSSPSSSPPTSVLGLGGLRISSDCSDGESDRELPSSATESDGSPVPSS
QPAFPVQILPYLYLGCAKDSTNLDVLGKYGIKYILNVTPNLPNAFEHGGEFTYKQIPISD
HWSQNLSQFFPEAISFIDEARSKKCGVLVHCLAGISRSVTVTVAYLMQKMNLSLNDAYDF
VKRKKSNISPNFNFMGQLLDFERTLGLSSPCDNHASSEQLYFSTPTNHNLFPLNTLEST

>hsa:1850
MAGDRLPRKVMDAKKLASLLRGGPGGPLVIDSRSFVEYNSWHVLSSVNICCSKLVKRRLQ
QGKVTIAELIQPAARSQVEATEPQDVVVYDQSTRDASVLAADSFLSILLSKLDGCFDSVA
ILTGGFATFSSCFPGLCEGKPAALLPMSLSQPCLPVPSVGLTRILPHLYLGSQKDVLNKD
LMTQNGISYVLNASNSCPKPDFICESRFMRVPINDNYCEKLLPWLDKSIEFIDKAKLSSC
QVIVHCLAGISRSATIAIAYIMKTMGMSSDDAYRFVKDRRPSISPNFNFLGQLLEYERSL
KLLAALQGDPGTPSGTPEPPPSPAAGAPLPRLPPPTSESAATGNAAAREGGLSAGGEPPA
PPTPPATSALQQGLRGLHLSSDRLQDTNRLKRSFSLDIKSAYAPSRRPDGPGPPDPGEAP
KLCKLDSPSGAALGLSSPSPDSPDAAPEARPRPRRRPRPPAGSPARSPAHSLGLNFGDAA
RQTPRHGLSALSAPGLPGPGQPAGPGAWAPPLDSPGTPSPDGPWCFSPEGAQGAGGVLFA
PFGRAGAPGPGGGSDLRRREAARAEPRDARTGWPEEPAPETQFKRRSCQMEFEEGMVEGR
ARGEELAALGKQASFSGSVEVIEVS
>hsa:1852
MEGLGRSCLWLRRELSPPRPRLLLLDCRSRELYESARIGGALSVALPALLLRRLRRGSLS
VRALLPGPPLQPPPPAPVLLYDQGGGRRRRGEAEAEAEEWEAESVLGTLLQKLREEGYLA
YYLQGGFSRFQAECPHLCETSLAGRAGSSMAPVPGPVPVVGLGSLCLGSDCSDAESEADR
DSMSCGLDSEGATPPPVGLRASFPVQILPNLYLGSARDSANLESLAKLGIRYILNVTPNL
PNFFEKNGDFHYKQIPISDHWSQNLSRFFPEAIEFIDEALSQNCGVLVHCLAGVSRSVTV
TVAYLMQKLHLSLNDAYDLVKRKKSNISPNFNFMGQLLDFERSLRLEERHSQEQGSGGQA
SAASNPPSFFTTPTSDGAFELAPT
>hsa:189
MASHKLLVTPPKALLKPLSIPNQLLLGPGPSNLPPRIMAAGGLQMIGSMSKDMYQIMDEI
KEGIQYVFQTRNPLTLVISGSGHCALEAALVNVLEPGDSFLVGANGIWGQRAVDIGERIG
ARVHPMTKDPGGHYTLQEVEEGLAQHKPVLLFLTHGESSTGVLQPLDGFGELCHRYKCLL
LVDSVASLGGTPLYMDRQGIDILYSGSQKALNAPPGTSLISFSDKAKKKMYSRKTKPFSF
YLDIKWLANFWGCDDQPRMYHHTIPVISLYSLRESLALIAEQGLENSWRQHREAAAYLHG
RLQALGLQLFVKDPALRLPTVTTVAVPAGYDWRDIVSYVIDHFDIEIMGGLGPSTGKVLR
IGLLGCNATRENVDRVTEALRAALQHCPKKKL
>hsa:1890
MAALMTPGTGAPPAPGDFSGEGSQGLPDPSPEPKQLPELIRMKRDGGRLSEADIRGFVAA
VVNGSAQGAQIGAMLMAIRLRGMDLEETSVLTQALAQSGQQLEWPEAWRQQLVDKHSTGG
VGDKVSLVLAPALAACGCKVPMISGRGLGHTGGTLDKLESIPGFNVIQSPEQMQVLLDQA
GCCIVGQSEQLVPADGILYAARDVTATVDSLPLITASILSKKLVEGLSALVVDVKFGGAA
VFPNQEQARELAKTLVGVGASLGLRVAAALTAMDKPLGRCVGHALEVEEALLCMDGAGPP
DLRDLVTTLGGALLWLSGHAGTQAQGAARVAAALDDGSALGRFERMLAAQGVDPGLARAL
CSGSPAERRQLLPRAREQEELLAPADGTVELVRALPLALVLHELGAGRSRAGEPLRLGVG
AELLVDVGQRLRRGTPWLRVHRDGPALSGPQSRALQEALVLSDRAPFAAPSPFAELVLPP
QQ
>hsa:191
MSDKLPYKVADIGLAAWGRKALDIAENEMPGLMRMRERYSASKPLKGARIAGCLHMTVET
AVLIETLVTLGAEVQWSSCNIFSTQDHAAAAIAKAGIPVYAWKGETDEEYLWCIEQTLYF
KDGPLNMILDDGGDLTNLIHTKYPQLLPGIRGISEETTTGVHNLYKMMANGILKVPAINV
NDSVTKSKFDNLYGCRESLIDGIKRATDVMIAGKVAVVAGYGDVGKGCAQALRGFGARVI
ITEIDPINALQAAMEGYEVTTMDEACQEGNIFVTTTGCIDIILGRHFEQMKDDAIVCNIG

HFDVEIDVKWLNENAVEKVNIKPQVDRYRLKNGRRIILLAEGRLVNLGCAMGHPSFVMSN
SFTNQVMAQIELWTHPDKYPVGVHFLPKKLDEAVAEAHLGKLNVKLTKLTEKQAQYLGMS
CDGPFKPDHYRY
>hsa:1956
MRPSGTAGAALLALLAALCPASRALEEKKVCQGTSNKLTQLGTFEDHFLSLQRMFNNCEV
VLGNLEITYVQRNYDLSFLKTIQEVAGYVLIALNTVERIPLENLQIIRGNMYYENSYALA
VLSNYDANKTGLKELPMRNLQEILHGAVRFSNNPALCNVESIQWRDIVSSDFLSNMSMDF
QNHLGSCQKCDPSCPNGSCWGAGEENCQKLTKIICAQQCSGRCRGKSPSDCCHNQCAAGC
TGPRESDCLVCRKFRDEATCKDTCPPLMLYNPTTYQMDVNPEGKYSFGATCVKKCPRNYV
VTDHGSCVRACGADSYEMEEDGVRKCKKCEGPCRKVCNGIGIGEFKDSLSINATNIKHFK
NCTSISGDLHILPVAFRGDSFTHTPPLDPQELDILKTVKEITGFLLIQAWPENRTDLHAF
ENLEIIRGRTKQHGQFSLAVVSLNITSLGLRSLKEISDGDVIISGNKNLCYANTINWKKL
FGTSGQKTKIISNRGENSCKATGQVCHALCSPEGCWGPEPRDCVSCRNVSRGRECVDKCN
LLEGEPREFVENSECIQCHPECLPQAMNITCTGRGPDNCIQCAHYIDGPHCVKTCPAGVM
GENNTLVWKYADAGHVCHLCHPNCTYGCTGPGLEGCPTNGPKIPSIATGMVGALLLLLVV
ALGIGLFMRRRHIVRKRTLRRLLQERELVEPLTPSGEAPNQALLRILKETEFKKIKVLGS
GAFGTVYKGLWIPEGEKVKIPVAIKELREATSPKANKEILDEAYVMASVDNPHVCRLLGI
CLTSTVQLITQLMPFGCLLDYVREHKDNIGSQYLLNWCVQIAKGMNYLEDRRLVHRDLAA
RNVLVKTPQHVKITDFGLAKLLGAEEKEYHAEGGKVPIKWMALESILHRIYTHQSDVWSY
GVTVWELMTFGSKPYDGIPASEISSILEKGERLPQPPICTIDVYMIMVKCWMIDADSRPK
FRELIIEFSKMARDPQRYLVIQGDERMHLPSPTDSNFYRALMDEEDMDDVVDADEYLIPQ
QGFFSSPSTSRTPLLSSLSATSNNSTVACIDRNGLQSCPIKEDSFLQRYSSDPTGALTED
SIDDTFLPVPEYINQSVPKRPAGSVQNPVYHNQPLNPAPSRDPHYQDPHSTAVGNPEYLN
TVQPTCVNSTFDSPAHWAQKGSHQISLDNPDYQQDFFPKEAKPNGIFKGSTAENAEYLRV
APQSSEFIGA
>hsa:196883
MARLFSPRPPPSEDLFYETYYSLSQQYPLLLLLLGIVLCALAALLAVAWASGRELTSDPS
FLTTVLCALGGFSLLLGLASREQRLQRWTRPLSGLVWVALLALGHAFLFTGGVVSAWDQV
SYFLFVIFTAYAMLPLGMRDAAVAGLASSLSHLLVLGLYLGPQPDSRPALLPQLAANAVL
FLCGNVAGVYHKALMERALRATFREALSSLHSRRRLDTEKKHQEHLLLSILPAYLAREMK
AEIMARLQAGQGSRPESTNNFHSLYVKRHQGVSVLYADIVGFTRLASECSPKELVLMLNE
LFGKFDQIAKEHECMRIKILGDCYYCVSGLPLSLPDHAINCVRMGLDMCRAIRKLRAATG
VDINMRVGVHSGSVLCGVIGLQKWQYDVWSHDVTLANHMEAGGVPGRVHITGATLALLAG
AYAVEDAGMEHRDPYLRELGEPTYLVIDPRAEEEDEKGTAGGLLSSLEGLKMRPSLLMTR
YLESWGAAKPFAHLSHGDSPVSTSTPLPEKTLASFSTQWSLDRSRTPRGLDDELDTGDAK
FFQVIEQLNSQKQWKQSKDFNPLTLYFREKEMEKEYRLSAIPAFKYYEACTFLVFLSNFI
IQMLVTNRPPALAITYSITFLLFLLILFVCFSEDLMRCVLKGPKMLHWLPALSGLVATRP
GLRIALGTATILLVFAMAITSLFFFPTSSDCPFQAPNVSSMISNLSWELPGSLPLISVPY
SMHCCTLGFLSCSLFLHMSFELKLLLLLLWLAASCSLFLHSHAWLSECLIVRLYLGPLDS
RPGVLKEPKLMGAISFFIFFFTLLVLARQNEYYCRLDFLWKKKLRQEREETETMENLTRL
LLENVLPAHVAPQFIGQNRRNEDLYHQSYECVCVLFASVPDFKEFYSESNINHEGLECLR
LLNEIIADFDELLSKPKFSGVEKIKTIGSTYMAATGLNATSGQDAQQDAERSCSHLGTMV
EFAVALGSKLDVINKHSFNNFRLRVGLNHGPVVAGVIGAQKPQYDIWGNTVNVASRMEST
GVLGKIQVTEETAWALQSLGYTCYSRGVIKVKGKGQLCTYFLNTDLTRTGPPSATLG

>hsa:1969
MELQAARACFALLWGCALAAAAAAQGKEVVLLDFAAAGGELGWLTHPYGKGWDLMQNIMN
DMPIYMYSVCNVMSGDQDNWLRTNWVYRGEAERIFIELKFTVRDCNSFPGGASSCKETFN
LYYAESDLDYGTNFQKRLFTKIDTIAPDEITVSSDFEARHVKLNVEERSVGPLTRKGFYL
AFQDIGACVALLSVRVYYKKCPELLQGLAHFPETIAGSDAPSLATVAGTCVDHAVVPPGG
EEPRMHCAVDGEWLVPIGQCLCQAGYEKVEDACQACSPGFFKFEASESPCLECPEHTLPS
PEGATSCECEEGFFRAPQDPASMPCTRPPSAPHYLTAVGMGAKVELRWTPPQDSGGREDI
VYSVTCEQCWPESGECGPCEASVRYSEPPHGLTRTSVTVSDLEPHMNYTFTVEARNGVSG
LVTSRSFRTASVSINQTEPPKVRLEGRSTTSLSVSWSIPPPQQSRVWKYEVTYRKKGDSN
SYNVRRTEGFSVTLDDLAPDTTYLVQVQALTQEGQGAGSKVHEFQTLSPEGSGNLAVIGG
VAVGVVLLLVLAGVGFFIHRRRKNQRARQSPEDVYFSKSEQLKPLKTYVDPHTYEDPNQA
VLKFTTEIHPSCVTRQKVIGAGEFGEVYKGMLKTSSGKKEVPVAIKTLKAGYTEKQRVDF
LGEAGIMGQFSHHNIIRLEGVISKYKPMMIITEYMENGALDKFLREKDGEFSVLQLVGML
RGIAAGMKYLANMNYVHRDLAARNILVNSNLVCKVSDFGLSRVLEDDPEATYTTSGGKIP
IRWTAPEAISYRKFTSASDVWSFGIVMWEVMTYGERPYWELSNHEVMKAINDGFRLPTPM
DCPSAIYQLMMQCWQQERARRPKFADIVSILDKLIRAPDSLKTLADFDPRVSIRLPSTSG
SEGVPFRTVSEWLESIKMQQYTEHFMAAGYTAIEKVVQMTNDDIKRIGVRLPGHQKRIAY
SLLGLKDQVNTVGIPI
>hsa:1990
MLVLYGHSTQDLPETNARVVGGTEAGRNSWPSQISLQYRSGGSRYHTCGGTLIRQNWVMT
AAHCVDYQKTFRVVAGDHNLSQNDGTEQYVSVQKIVVHPYWNSDNVAAGYDIALLRLAQS
VTLNSYVQLGVLPQEGAILANNSPCYITGWGKTKTNGQLAQTLQQAYLPSVDYAICSSSS
YWGSTVKNTMVCAGGDGVRSGCQGDSGGPLHCLVNGKYSVHGVTSFVSSRGCNVSRKPTV
FTQVSAYISWINNVIASN
>hsa:1991
MTLGRRLACLFLACVLPALLLGGTALASEIVGGRRARPHAWPFMVSLQLRGGHFCGATLI
APNFVMSAAHCVANVNVRAVRVVLGAHNLSRREPTRQVFAVQRIFENGYDPVNLLNDIVI
LQLNGSATINANVQVAQLPAQGRRLGNGVQCLAMGWGLLGRNRGIASVLQELNVTVVTSL
CRRSNVCTLVRGRQAGVCFGDSGSPLVCNGLIHGIASFVRGGCASGLYPDAFAPVAQFVN
WIDSIIQRSEDNPCPHPRDPDPASRTH
>hsa:199974
MEPSWLQELMAHPFLLLILLCMSLLLFQVIRLYQRRRWMIRALHLFPAPPAHWFYGHKEF
YPVKEFEVYHKLMEKYPCAVPLWVGPFTMFFSVHDPDYAKILLKRQDPKSAVSHKILESW
VGRGLVTLDGSKWKKHRQIVKPGFNISILKIFITMMSESVRMMLNKWEEHIAQNSRLELF
QHVSLMTLDSIMKCAFSHQGSIQLDSTLDSYLKAVFNLSKISNQRMNNFLHHNDLVFKFS
SQGQIFSKFNQELHQFTEKVIQDRKESLKDKLKQDTTQKRRWDFLDILLSAKSENTKDFS
EADLQAEVKTFMFAGHDTTSSAISWILYCLAKYPEHQQRCRDEIRELLGDGSSITWEHLS
QMPYTTMCIKECLRLYAPVVNISRLLDKPITFPDGRSLPAGITVFINIWALHHNPYFWED
PQVFNPLRFSRENSEKIHPYAFIPFSAGLRNCIGQHFAIIECKVAVALTLLRFKLAPDHS
RPPQPVRQVVLKSKNGIHVFAKKVC
>hsa:2041
MERRWPLGLGLVLLLCAPLPPGARAKEVTLMDTSKAQGELGWLLDPPKDGWSEQQQILNG
TPLYMYQDCPMQGRRDTDHWLRSNWIYRGEEASRVHVELQFTVRDCKSFPGGAGPLGCKE
TFNLLYMESDQDVGIQLRRPLFQKVTTVAADQSFTIRDLVSGSVKLNVERCSLGRLTRRG

LYLAFHNPGACVALVSVRVFYQRCPETLNGLAQFPDTLPGPAGLVEVAGTCLPHARASPR
PSGAPRMHCSPDGEWLVPVGRCHCEPGYEEGGSGEACVACPSGSYRMDMDTPHCLTCPQQ
STAESEGATICTCESGHYRAPGEGPQVACTGPPSAPRNLSFSASGTQLSLRWEPPADTGG
RQDVRYSVRCSQCQGTAQDGGPCQPCGVGVHFSPGARGLTTPAVHVNGLEPYANYTFNVE
AQNGVSGLGSSGHASTSVSISMGHAESLSGLSLRLVKKEPRQLELTWAGSRPRSPGANLT
YELHVLNQDEERYQMVLEPRVLLTELQPDTTYIVRVRMLTPLGPGPFSPDHEFRTSPPVS
RGLTGGEIVAVIFGLLLGAALLLGILVFRSRRAQRQRQQRQRDRATDVDREDKLWLKPYV
DLQAYEDPAQGALDFTRELDPAWLMVDTVIGEGEFGEVYRGTLRLPSQDCKTVAIKTLKD
TSPGGQWWNFLREATIMGQFSHPHILHLEGVVTKRKPIMIITEFMENGALDAFLREREDQ
LVPGQLVAMLQGIASGMNYLSNHNYVHRDLAARNILVNQNLCCKVSDFGLTRLLDDFDGT
YETQGGKIPIRWTAPEAIAHRIFTTASDVWSFGIVMWEVLSFGDKPYGEMSNQEVMKSIE
DGYRLPPPVDCPAPLYELMKNCWAYDRARRPHFQKLQAHLEQLLANPHSLRTIANFDPRM
TLRLPSLSGSDGIPYRTVSEWLESIRMKRYILHFHSAGLDTMECVLELTAEDLTQMGITL
PGHQKRILCSIQGFKD
>hsa:2042
MDCQLSILLLLSCSVLDSFGELIPQPSNEVNLLDSKTIQGELGWISYPSHGWEEISGVDE
HYTPIRTYQVCNVMDHSQNNWLRTNWVPRNSAQKIYVELKFTLRDCNSIPLVLGTCKETF
NLYYMESDDDHGVKFREHQFTKIDTIAADESFTQMDLGDRILKLNTEIREVGPVNKKGFY
LAFQDVGACVALVSVRVYFKKCPFTVKNLAMFPDTVPMDSQSLVEVRGSCVNNSKEEDPP
RMYCSTEGEWLVPIGKCSCNAGYEERGFMCQACRPGFYKALDGNMKCAKCPPHSSTQEDG
SMNCRCENNYFRADKDPPSMACTRPPSSPRNVISNINETSVILDWSWPLDTGGRKDVTFN
IICKKCGWNIKQCEPCSPNVRFLPRQFGLTNTTVTVTDLLAHTNYTFEIDAVNGVSELSS
PPRQFAAVSITTNQAAPSPVLTIKKDRTSRNSISLSWQEPEHPNGIILDYEVKYYEKQEQ
ETSYTILRARGTNVTISSLKPDTIYVFQIRARTAAGYGTNSRKFEFETSPDSFSISGESS
QVVMIAISAAVAIILLTVVIYVLIGRFCGYKSKHGADEKRLHFGNGHLKLPGLRTYVDPH
TYEDPTQAVHEFAKELDATNISIDKVVGAGEFGEVCSGRLKLPSKKEISVAIKTLKVGYT
EKQRRDFLGEASIMGQFDHPNIIRLEGVVTKSKPVMIVTEYMENGSLDSFLRKHDAQFTV
IQLVGMLRGIASGMKYLSDMGYVHRDLAARNILINSNLVCKVSDFGLSRVLEDDPEAAYT
TRGGKIPIRWTSPEAIAYRKFTSASDVWSYGIVLWEVMSYGERPYWEMSNQDVIKAVDEG
YRLPPPMDCPAALYQLMLDCWQKDRNNRPKFEQIVSILDKLIRNPGSLKIITSAAARPSN
LLLDQSNVDITTFRTTGDWLNGVWTAHCKEIFTGVEYSSCDTIAKISTDDMKKVGVTVVG
PQKKIISSIKALETQSKNGPVPV
>hsa:2043
MAGIFYFALFSCLFGICDAVTGSRVYPANEVTLLDSRSVQGELGWIASPLEGGWEEVSIM
DEKNTPIRTYQVCNVMEPSQNNWLRTDWITREGAQRVYIEIKFTLRDCNSLPGVMGTCKE
TFNLYYYESDNDKERFIRENQFVKIDTIAADESFTQVDIGDRIMKLNTEIRDVGPLSKKG
FYLAFQDVGACIALVSVRVFYKKCPLTVRNLAQFPDTITGADTSSLVEVRGSCVNNSEEK
DVPKMYCGADGEWLVPIGNCLCNAGHEERSGECQACKIGYYKALSTDATCAKCPPHSYSV
WEGATSCTCDRGFFRADNDAASMPCTRPPSAPLNLISNVNETSVNLEWSSPQNTGGRQDI
SYNVVCKKCGAGDPSKCRPCGSGVHYTPQQNGLKTTKVSITDLLAHTNYTFEIWAVNGVS
KYNPNPDQSVSVTVTTNQAAPSSIALVQAKEVTRYSVALAWLEPDRPNGVILEYEVKYYE
KDQNERSYRIVRTAARNTDIKGLNPLTSYVFHVRARTAAGYGDFSEPLEVTTNTVPSRII
GDGANSTVLLVSVSGSVVLVVILIAAFVISRRRSKYSKAKQEADEEKHLNQGVRTYVDPF
TYEDPNQAVREFAKEIDASCIKIEKVIGVGEFGEVCSGRLKVPGKREICVAIKTLKAGYT

DKQRRDFLSEASIMGQFDHPNIIHLEGVVTKCKPVMIITEYMENGSLDAFLRKNDGRFTV
IQLVGMLRGIGSGMKYLSDMSYVHRDLAARNILVNSNLVCKVSDFGMSRVLEDDPEAAYT
TRGGKIPIRWTAPEAIAYRKFTSASDVWSYGIVMWEVMSYGERPYWDMSNQDVIKAIEEG
YRLPPPMDCPIALHQLMLDCWQKERSDRPKFGQIVNMLDKLIRNPNSLKRTGTESSRPNT
ALLDPSSPEFSAVVSVGDWLQAIKMDRYKDNFTAAGYTTLEAVVHVNQEDLARIGITAIT
HQNKILSSVQAMRTQMQQMHGRMVPV
>hsa:2044
MRGSGPRGAGRRRPPSGGGDTPITPASLAGCYSAPRRAPLWTCLLLCAALRTLLASPSNE
VNLLDSRTVMGDLGWIAFPKNGWEEIGEVDENYAPIHTYQVCKVMEQNQNNWLLTSWISN
EGASRIFIELKFTLRDCNSLPGGLGTCKETFNMYYFESDDQNGRNIKENQYIKIDTIAAD
ESFTELDLGDRVMKLNTEVRDVGPLSKKGFYLAFQDVGACIALVSVRVYYKKCPSVVRHL
AVFPDTITGADSSQLLEVSGSCVNHSVTDEPPKMHCSAEGEWLVPIGKCMCKAGYEEKNG
TCQVCRPGFFKASPHIQSCGKCPPHSYTHEEASTSCVCEKDYFRRESDPPTMACTRPPSA
PRNAISNVNETSVFLEWIPPADTGGRKDVSYYIACKKCNSHAGVCEECGGHVRYLPRQSG
LKNTSVMMVDLLAHTNYTFEIEAVNGVSDLSPGARQYVSVNVTTNQAAPSPVTNVKKGKI
AKNSISLSWQEPDRPNGIILEYEIKYFEKDQETSYTIIKSKETTITAEGLKPASVYVFQI
RARTAAGYGVFSRRFEFETTPVFAASSDQSQIPVIAVSVTVGVILLAVVIGVLLSGSCCE
CGCGRASSLCAVAHPSLIWRCGYSKAKQDPEEEKMHFHNGHIKLPGVRTYIDPHTYEDPN
QAVHEFAKEIEASCITIERVIGAGEFGEVCSGRLKLPGKRELPVAIKTLKVGYTEKQRRD
FLGEASIMGQFDHPNIIHLEGVVTKSKPVMIVTEYMENGSLDTFLKKNDGQFTVIQLVGM
LRGISAGMKYLSDMGYVHRDLAARNILINSNLVCKVSDFGLSRVLEDDPEAAYTTRGGKI
PIRWTAPEAIAFRKFTSASDVWSYGIVMWEVVSYGERPYWEMTNQDVIKAVEEGYRLPSP
MDCPAALYQLMLDCWQKERNSRPKFDEIVNMLDKLIRNPSSLKTLVNASCRVSNLLAEHS
PLGSGAYRSVGEWLEAIKMGRYTEIFMENGYSSMDAVAQVTLEDLRRLGVTLVGHQKKIM
NSLQEMKVQLVNGMVPL
>hsa:2045
MVFQTRYPSWIILCYIWLLRFAHTGEAQAAKEVLLLDSKAQQTELEWISSPPNGWEEISG
LDENYTPIRTYQVCQVMEPNQNNWLRTNWISKGNAQRIFVELKFTLRDCNSLPGVLGTCK
ETFNLYYYETDYDTGRNIRENLYVKIDTIAADESFTQGDLGERKMKLNTEVREIGPLSKK
GFYLAFQDVGACIALVSVKVYYKKCWSIIENLAIFPDTVTGSEFSSLVEVRGTCVSSAEE
EAENAPRMHCSAEGEWLVPIGKCICKAGYQQKGDTCEPCGRGFYKSSSQDLQCSRCPTHS
FSDKEGSSRCECEDGYYRAPSDPPYVACTRPPSAPQNLIFNINQTTVSLEWSPPADNGGR
NDVTYRILCKRCSWEQGECVPCGSNIGYMPQQTGLEDNYVTVMDLLAHANYTFEVEAVNG
VSDLSRSQRLFAAVSITTGQAAPSQVSGVMKERVLQRSVELSWQEPEHPNGVITEYEIKY
YEKDQRERTYSTVKTKSTSASINNLKPGTVYVFQIRAFTAAGYGNYSPRLDVATLEEATG
KMFEATAVSSEQNPVIIIAVVAVAGTIILVFMVFGFIIGRRHCGYSKADQEGDEELYFHF
KFPGTKTYIDPETYEDPNRAVHQFAKELDASCIKIERVIGAGEFGEVCSGRLKLPGKRDV
AVAIKTLKVGYTEKQRRDFLCEASIMGQFDHPNVVHLEGVVTRGKPVMIVIEFMENGALD
AFLRKHDGQFTVIQLVGMLRGIAAGMRYLADMGYVHRDLAARNILVNSNLVCKVSDFGLS
RVIEDDPEAVYTTTGGKIPVRWTAPEAIQYRKFTSASDVWSYGIVMWEVMSYGERPYWDM
SNQDVIKAIEEGYRLPAPMDCPAGLHQLMLDCWQKERAERPKFEQIVGILDKMIRNPNSL
KTPLGTCSRPISPLLDQNTPDFTTFCSVGEWLQAIKMERYKDNFTAAGYNSLESVARMTI
EDVMSLGITLVGHQKKIMSSIQTMRAQMLHLHGTGIQV
>hsa:2046

MAPARGRLPPALWVVTAAAAAATCVSAARGEVNLLDTSTIHGDWGWLTYPAHGWDSINEV
DESFQPIHTYQVCNVMSPNQNNWLRTSWVPRDGARRVYAEIKFTLRDCNSMPGVLGTCKE
TFNLYYLESDRDLGASTQESQFLKIDTIAADESFTGADLGVRRLKLNTEVRSVGPLSKRG
FYLAFQDIGACLAILSLRIYYKKCPAMVRNLAAFSEAVTGADSSSLVEVRGQCVRHSEER
DTPKMYCSAEGEWLVPIGKCVCSAGYEERRDACVACELGFYKSAPGDQLCARCPPHSHSA
APAAQACHCDLSYYRAALDPPSSACTRPPSAPVNLISSVNGTSVTLEWAPPLDPGGRSDI
TYNAVCRRCPWALSRCEACGSGTRFVPQQTSLVQASLLVANLLAHMNYSFWIEAVNGVSD
LSPEPRRAAVVNITTNQAGRRRNSVPQRPGPPASPASDPSRDQSSAGDVLWAFRQVPLWP
CAPHQDPELEALHCL
>hsa:2047
MALDYLLLLLLASAVAAMEETLMDTRTATAELGWTANPASGWEEVSGYDENLNTIRTYQV
CNVFEPNQNNWLLTTFINRRGAHRIYTEMRFTVRDCSSLPNVPGSCKETFNLYYYETDSV
IATKKSAFWSEAPYLKVDTIAADESFSQVDFGGRLMKVNTEVRSFGPLTRNGFYLAFQDY
GACMSLLSVRVFFKKCPSIVQNFAVFPETMTGAESTSLVIARGTCIPNAEEVDVPIKLYC
NGDGEWMVPIGRCTCKPGYEPENSVACKACPAGTFKASQEAEGCSHCPSNSRSPAEASPI
CTCRTGYYRADFDPPEVACTSVPSGPRNVISIVNETSIILEWHPPRETGGRDDVTYNIIC
KKCRADRRSCSRCDDNVEFVPRQLGLTECRVSISSLWAHTPYTFDIQAINGVSSKSPFPP
QHVSVNITTNQAAPSTVPIMHQVSATMRSITLSWPQPEQPNGIILDYEIRYYEKEHNEFN
SSMARSQTNTARIDGLRPGMVYVVQVRARTVAGYGKFSGKMCFQTLTDDDYKSELREQLP
LIAGSAAAGVVFVVSLVAISIVCSRKRAYSKEAVYSDKLQHYSTGRGSPGMKIYIDPFTY
EDPNEAVREFAKEIDVSFVKIEEVIGAGEFGEVYKGRLKLPGKREIYVAIKTLKAGYSEK
QRRDFLSEASIMGQFDHPNIIRLEGVVTKSRPVMIITEFMENGALDSFLRQNDGQFTVIQ
LVGMLRGIAAGMKYLAEMNYVHRDLAARNILVNSNLVCKVSDFGLSRYLQDDTSDPTYTS
SLGGKIPVRWTAPEAIAYRKFTSASDVWSYGIVMWEVMSFGERPYWDMSNQDVINAIEQD
YRLPPPMDCPAALHQLMLDCWQKDRNSRPRFAEIVNTLDKMIRNPASLKTVATITAVPSQ
PLLDRSIPDFTAFTTVDDWLSAIKMVQYRDSFLTAGFTSLQLVTQMTSEDLLRIGITLAG
HQKKILNSIHSMRVQISQSPTAMA
>hsa:2048
MALRRLGAALLLLPLLAAVEETLMDSTTATAELGWMVHPPSGWEEVSGYDENMNTIRTYQ
VCNVFESSQNNWLRTKFIRRRGAHRIHVEMKFSVRDCSSIPSVPGSCKETFNLYYYEADF
DSATKTFPNWMENPWVKVDTIAADESFSQVDLGGRVMKINTEVRSFGPVSRSGFYLAFQD
YGGCMSLIAVRVFYRKCPRIIQNGAIFQETLSGAESTSLVAARGSCIANAEEVDVPIKLY
CNGDGEWLVPIGRCMCKAGFEAVENGTVCRGCPSGTFKANQGDEACTHCPINSRTTSEGA
TNCVCRNGYYRADLDPLDMPCTTIPSAPQAVISSVNETSLMLEWTPPRDSGGREDLVYNI
ICKSCGSGRGACTRCGDNVQYAPRQLGLTEPRIYISDLLAHTQYTFEIQAVNGVTDQSPF
SPQFASVNITTNQAAPSAVSIMHQVSRTVDSITLSWSQPDQPNGVILDYELQYYEKELSE
YNATAIKSPTNTVTVQGLKAGAIYVFQVRARTVAGYGRYSGKMYFQTMTEAEYQTSIQEK
LPLIIGSSAAGLVFLIAVVVIAIVCNRRRGFERADSEYTDKLQHYTSGHMTPGMKIYIDP
FTYEDPNEAVREFAKEIDISCVKIEQVIGAGEFGEVCSGHLKLPGKREIFVAIKTLKSGY
TEKQRRDFLSEASIMGQFDHPNVIHLEGVVTKSTPVMIITEFMENGSLDSFLRQNDGQFT
VIQLVGMLRGIAAGMKYLADMNYVHRDLAARNILVNSNLVCKVSDFGLSRFLEDDTSDPT
YTSALGGKIPIRWTAPEAIQYRKFTSASDVWSYGIVMWEVMSYGERPYWDMTNQDVINAI
EQDYRLPPPMDCPSALHQLMLDCWQKDRNHRPKFGQIVNTLDKMIRNPNSLKAMAPLSSG
INLPLLDRTIPDYTSFNTVDEWLEAIKMGQYKESFANAGFTSFDVVSQMMMEDILRVGVT

LAGHQKKILNSIQVMRAQMNQIQSVEV
>hsa:2049
MARARPPPPPSPPPGLLPLLPPLLLLPLLLLPAGCRALEETLMDTKWVTSELAWTSHPES
GWEEVSGYDEAMNPIRTYQVCNVRESSQNNWLRTGFIWRRDVQRVYVELKFTVRDCNSIP
NIPGSCKETFNLFYYEADSDVASASSPFWMENPYVKVDTIAPDESFSRLDAGRVNTKVRS
FGPLSKAGFYLAFQDQGACMSLISVRAFYKKCASTTAGFALFPETLTGAEPTSLVIAPGT
CIPNAVEVSVPLKLYCNGDGEWMVPVGACTCATGHEPAAKESQCRPCPPGSYKAKQGEGP
CLPCPPNSRTTSPAASICTCHNNFYRADSDSADSACTTVPSPPRGVISNVNETSLILEWS
EPRDLGGRDDLLYNVICKKCHGAGGASACSRCDDNVEFVPRQLGLTERRVHISHLLAHTR
YTFEVQAVNGVSGKSPLPPRYAAVNITTNQAAPSEVPTLRLHSSSGSSLTLSWAPPERPN
GVILDYEMKYFEKSEGIASTVTSQMNSVQLDGLRPDARYVVQVRARTVAGYGQYSRPAEF
ETTSERGSGAQQLQEQLPLIVGSATAGLVFVVAVVVIAIVCLRKQRHGSDSEYTEKLQQY
IAPGMKVYIDPFTYEDPNEAVREFAKEIDVSCVKIEEVIGAGEFGEVCRGRLKQPGRREV
FVAIKTLKVGYTERQRRDFLSEASIMGQFDHPNIIRLEGVVTKSRPVMILTEFMENCALD
SFLRLNDGQFTVIQLVGMLRGIAAGMKYLSEMNYVHRDLAARNILVNSNLVCKVSDFGLS
RFLEDDPSDPTYTSSLGGKIPIRWTAPEAIAYRKFTSASDVWSYGIVMWEVMSYGERPYW
DMSNQDVINAVEQDYRLPPPMDCPTALHQLMLDCWVRDRNLRPKFSQIVNTLDKLIRNAA
SLKVIASAQSGMSQPLLDRTVPDYTTFTTVGDWLDAIKMGRYKESFVSAGFASFDLVAQM
TAEDLLRIGVTLAGHQKKILSSIQDMRLQMNQTLPVQV
>hsa:2050
MELRVLLCWASLAAALEETLLNTKLETADLKWVTFPQVDGQWEELSGLDEEQHSVRTYEV
CDVQRAPGQAHWLRTGWVPRRGAVHVYATLRFTMLECLSLPRAGRSCKETFTVFYYESDA
DTATALTPAWMENPYIKVDTVAAEHLTRKRPGAEATGKVNVKTLRLGPLSKAGFYLAFQD
QGACMALLSLHLFYKKCAQLTVNLTRFPETVPRELVVPVAGSCVVDAVPAPGPSPSLYCR
EDGQWAEQPVTGCSCAPGFEAAEGNTKCRACAQGTFKPLSGEGSCQPCPANSHSNTIGSA
VCQCRVGYFRARTDPRGAPCTTPPSAPRSVVSRLNGSSLHLEWSAPLESGGREDLTYALR
CRECRPGGSCAPCGGDLTFDPGPRDLVEPWVVVRGLRPDFTYTFEVTALNGVSSLATGPV
PFEPVNVTTDREVPPAVSDIRVTRSSPSSLSLAWAVPRAPSGAVLDYEVKYHEKGAEGPS
SVRFLKTSENRAELRGLKRGASYLVQVRARSEAGYGPFGQEHHSQTQLDESEGWREQLAL
IAGTAVVGVVLVLVVIVVAVLCLRKQSNGREAEYSDKHGQYLIGHGTKVYIDPFTYEDPN
EAVREFAKEIDVSYVKIEEVIGAGEFGEVCRGRLKAPGKKESCVAIKTLKGGYTERQRRE
FLSEASIMGQFEHPNIIRLEGVVTNSMPVMILTEFMENGALDSFLRLNDGQFTVIQLVGM
LRGIASGMRYLAEMSYVHRDLAARNILVNSNLVCKVSDFGLSRFLEENSSDPTYTSSLGG
KIPIRWTAPEAIAFRKFTSASDAWSYGIVMWEVMSFGERPYWDMSNQDVINAIEQDYRLP
PPPDCPTSLHQLMLDCWQKDRNARPRFPQVVSALDKMIRNPASLKIVARENGGASHPLLD
QRQPHYSAFGSVGEWLRAIKMGRYEESFAAAGFGSFELVSQISAEDLLRIGVTLAGHQKK
ILASVQHMKSQAKPGTPGGTGGPAPQY
>hsa:2051
MATEGAAQLGNRVAGMVCSLWVLLLVSSVLALEEVLLDTTGETSEIGWLTYPPGGWDEVS
VLDDQRRLTRTFEACHVAGAPPGTGQDNWLQTHFVERRGAQRAHIRLHFSVRACSSLGVS
GGTCRETFTLYYRQAEEPDSPDSVSSWHLKRWTKVDTIAADESFPSSSSSSSSSSSAAWA
VGPHGAGQRAGLQLNVKERSFGPLTQRGFYVAFQDTGACLALVAVRLFSYTCPAVLRSFA
SFPETQASGAGGASLVAAVGTCVAHAEPEEDGVGGQAGGSPPRLHCNGEGKWMVAVGGCR
CQPGYQPARGDKACQACPRGLYKSSAGNAPCSPCPARSHAPNPAAPVCPCLEGFYRASSD

PPEAPCTGPPSAPQELWFEVQGSALMLHWRLPRELGGRGDLLFNVVCKECEGRQEPASGG
GGTCHRCRDEVHFDPRQRGLTESRVLVGGLRAHVPYILEVQAVNGVSELSPDPPQAAAIN
VSTSHEVPSAVPVVHQVSRASNSITVSWPQPDQTNGNILDYQLRYYDQAEDESHSFTLTS
ETNTATVTQLSPGHIYGFQVRARTAAGHGPYGGKVYFQTLPQGELSSQLPERLSLVIGSI
LGALAFLLLAAITVLAVVFQRKRRGTGYTEQLQQYSSPGLGVKYYIDPSTYEDPCQAIRE
LAREVDPAYIKIEEVIGTGSFGEVRQGRLQPRGRREQTVAIQALWAGGAESLQMTFLGRA
AVLGQFQHPNILRLEGVVTKSRPLMVLTEFMELGPLDSFLRQREGQFSSLQLVAMQRGVA
AAMQYLSSFAFVHRSLSAHSVLVNSHLVCKVARLGHSPQGPSCLLRWAAPEVIAHGKHTT
SSDVWSFGILMWEVMSYGERPYWDMSEQEVLNAIEQEFRLPPPPGCPPGLHLLMLDTWQK
DRARRPHFDQLVAAFDKMIRKPDTLQAGGDPGERPSQALLTPVALDFPCLDSPQAWLSAI
GLECYQDNFSKFGLCTFSDVAQLSLEDLPALGITLAGHQKKLLHHIQLLQQHLRQQGSVE
V
>hsa:2058
MATLSLTVNSGDPPLGALLAVEHVKDDVSISVEEGKENILHVSENVIFTDVNSILRYLAR
VATTAGLYGSNLMEHTEIDHWLEFSATKLSSCDSFTSTINELNHCLSLRTYLVGNSLSLA
DLCVWATLKGNAAWQEQLKQKKAPVHVKRWFGFLEAQQAFQSVGTKWDVSTTKARVAPEK
KQDVGKFVELPGAEMGKVTVRFPPEASGYLHIGHAKAALLNQHYQVNFKGKLIMRFDDTN
PEKEKEDFEKVILEDVAMLHIKPDQFTYTSDHFETIMKYAEKLIQEGKAYVDDTPAEQMK
AEREQRIDSKHRKNPIEKNLQMWEEMKKGSQFGQSCCLRAKIDMSSNNGCMRDPTLYRCK
IQPHPRTGNKYNVYPTYDFACPIVDSIEGVTHALRTTEYHDRDEQFYWIIEALGIRKPYI
WEYSRLNLNNTVLSKRKLTWFVNEGLVDGWDDPRFPTVRGVLRRGMTVEGLKQFIAAQGS
SRSVVNMEWDKIWAFNKKVIDPVAPRYVALLKKEVIPVNVPEAQEEMKEVAKHPKNPEVG
LKPVWYSPKVFIEGADAETFSEGEMVTFINWGNLNITKIHKNADGKIISLDAKLNLENKD
YKKTTKVTWLAETTHALPIPVICVTYEHLITKPVLGKDEDFKQYVNKNSKHEELMLGDPC
LKDLKKGDIIQLQRRGFFICDQPYEPVSPYSCKEAPCVLIYIPDGHTKEMPTSGSKEKTK
VEATKNETSAPFKERPTPSLNNNCTTSEDSLVLYNRVAVQGDVVRELKAKKAPKEDVDAA
VKQLLSLKAEYKEKTGQEYKPGNPPAEIGQNISSNSSASILESKSLYDEVAAQGEVVRKL
KAEKSPKAKINEAVECLLSLKAQYKEKTGKEYIPGQPPLSQSSDSSPTRNSEPAGLETPE
AKVLFDKVASQGEVVRKLKTEKAPKDQVDIAVQELLQLKAQYKSLIGVEYKPVSATGAED
KDKKKKEKENKSEKQNKPQKQNDGQRKDPSKNQGGGLSSSGAGEGQGPKKQTRLGLEAKK
EENLADWYSQVITKSEMIEYHDISGCYILRPWAYAIWEAIKDFFDAEIKKLGVENCYFPM
FVSQSALEKEKTHVADFAPEVAWVTRSGKTELAEPIAIRPTSETVMYPAYAKWVQSHRDL
PIKLNQWCNVVRWEFKHPQPFLRTREFLWQEGHSAFATMEEAAEEVLQILDLYAQVYEEL
LAIPVVKGRKTEKEKFAGGDYTTTIEAFISASGRAIQGGTSHHLGQNFSKMFEIVFEDPK
IPGEKQFAYQNSWGLTTRTIGVMTMVHGDNMGLVLPPRVACVQVVIIPCGITNALSEEDK
EALIAKCNDYRRRLLSVNIRVRADLRDNYSPGWKFNHWELKGVPIRLEVGPRDMKSCQFV
AVRRDTGEKLTVAENEAETKLQAILEDIQVTLFTRASEDLKTHMVVANTMEDFQKILDSG
KIVQIPFCGEIDCEDWIKKTTARDQDLEPGAPSMGAKSLCIPFKPLCELQPGAKCVCGKN
PAKYYTLFGRSY
>hsa:2064
MKLRLPASPETHLDMLRHLYQGCQVVQGNLELTYLPTNASLSFLQDIQEVQGYVLIAHNQ
VRQVPLQRLRIVRGTQLFEDNYALAVLDNGDPLNNTTPVTGASPGGLRELQLRSLTEILK
GGVLIQRNPQLCYQDTILWKDIFHKNNQLALTLIDTNRSRACHPCSPMCKGSRCWGESSE
DCQSLTRTVCAGGCARCKGPLPTDCCHEQCAAGCTGPKHSDCLACLHFNHSGICELHCPA

LVTYNTDTFESMPNPEGRYTFGASCVTACPYNYLSTDVGSCTLVCPLHNQEVTAEDGTQR
CEKCSKPCARVCYGLGMEHLREVRAVTSANIQEFAGCKKIFGSLAFLPESFDGDPASNTA
PLQPEQLQVFETLEEITGYLYISAWPDSLPDLSVFQNLQVIRGRILHNGAYSLTLQGLGI
SWLGLRSLRELGSGLALIHHNTHLCFVHTVPWDQLFRNPHQALLHTANRPEDECVGEGLA
CHQLCARGHCWGPGPTQCVNCSQFLRGQECVEECRVLQGLPREYVNARHCLPCHPECQPQ
NGSVTCFGPEADQCVACAHYKDPPFCVARCPSGVKPDLSYMPIWKFPDEEGACQPCPINC
THSCVDLDDKGCPAEQRASPLTSIISAVVGILLVVVLGVVFGILIKRRQQKIRKYTMRRL
LQETELVEPLTPSGAMPNQAQMRILKETELRKVKVLGSGAFGTVYKGIWIPDGENVKIPV
AIKVLRENTSPKANKEILDEAYVMAGVGSPYVSRLLGICLTSTVQLVTQLMPYGCLLDHV
RENRGRLGSQDLLNWCMQIAKGMSYLEDVRLVHRDLAARNVLVKSPNHVKITDFGLARLL
DIDETEYHADGGKVPIKWMALESILRRRFTHQSDVWSYGVTVWELMTFGAKPYDGIPARE
IPDLLEKGERLPQPPICTIDVYMIMVKCWMIDSECRPRFRELVSEFSRMARDPQRFVVIQ
NEDLGPASPLDSTFYRSLLEDDDMGDLVDAEEYLVPQQGFFCPDPAPGAGGMVHHRHRSS
STRSGGGDLTLGLEPSEEEAPRSPLAPSEGAGSDVFDGDLGMGAAKGLQSLPTHDPSPLQ
RYSEDPTVPLPSETDGYVAPLTCSPQPEYVNQPDVRPQPPSPREGPLPAARPAGATLERP
KTLSPGKNGVVKDVFAFGGAVENPEYLTPQGGAAPQPHPPPAFSPAFDNLYYWDQDPPER
GAPPSTFKGTPTAENPEYLGLDVPV

>hsa:2065

MRANDALQVLGLLFSLARGSEVGNSQAVCPGTLNGLSVTGDAENQYQTLYKLYERCEVVM
GNLEIVLTGHNADLSFLQWIREVTGYVLVAMNEFSTLPLPNLRVVRGTQVYDGKFAIFVM
LNYNTNSSHALRQLRLTQLTGQFPMVPSGLTPQPAQDWYLLDDDPRLLTLSASSKVPVTL
AAV

>hsa:2066

MKPATGLWVWVSLLVAAGTVQPSDSQSVCAGTENKLSSLSDLEQQYRALRKYYENCEVVM
GNLEITSIEHNRDLSFLRSVREVTGYVLVALNQFRYLPLENLRIIRGTKLYEDRYALAIF
LNYRKDGNFGLQELGLKNLTEILNGGVYVDQNKFLCYADTIHWQDIVRNPWPSNLTLVST
NGSSGCGRCHKSCTGRCWGPTENHCQTLTRTVCAEQCDGRCYGPYVSDCCHRECAGGCSG
PKDTDCFACMNFNDSGACVTQCPQTFVYNPTTFQLEHNFNAKYTYGAFCVKKCPHNFVVD
SSSCVRACPSSKMEVEENGIKMCKPCTDICPKACDGIGTGSLMSAQTVDSSNIDKFINCT
KINGNLIFLVTGIHGDPYNAIEAIDPEKLNVFRTVREITGFLNIQSWPPNMTDFSVFSNL
VTIGGRVLYSGLSLLILKQQGITSLQFQSLKEISAGNIYITDNSNLCYYHTINWTTLFST
INQRIVIRDNRKAENCTAEGMVCNHLCSSDGCWGPGPDQCLSCRRFSRGRICIESCNLYD
GEFREFENGSICVECDPQCEKMEDGLLTCHGPGPDNCTKCSHFKDGPNCVEKCPDGLQGA
NSFIFKYADPDRECHPCHPNCTQGCNGPTSHDCIYYPWTGHSTLPQHARTPLIAAGVIGG
LFILVIVGLTFAVYVRRKSIKKKRALRRFLETELVEPLTPSGTAPNQAQLRILKETELKR
VKVLGSGAFGTVYKGIWVPEGETVKIPVAIKILNETTGPKANVEFMDEALIMASMDHPHL
VRLLGVCLSPTIQLVTQLMPHGCLLEYVHEHKDNIGSQLLLNWCVQIAKGMMYLEERRLV
HRDLAARNVLVKSPNHVKITDFGLARLLEGDEKEYNADGGKMPIKWMALECIHYRKFTHQ
SDVWSYGVTIWELMTFGGKPYDGIPTREIPDLLEKGERLPQPPICTIDVYMVMVKCWMID
ADSRPKFKELAAEFSRMARDPQRYLVIQGDDRMKLPSPNDSKFFQNLLDEEDLEDMMDAE
EYLVPQAFNIPPPIYTSRARIDSNRNQFVYRDGGFAAEQGVSVPYRAPTSTIPEAPVAQG
ATAEIFDDSCCNGTLRKPVAPHVQEDSSTQRYSADPTVFAPERSPRGELDEEGYMTPMRD
KPKQEYLNPVEENPFVSRRKNGDLQALDNPEYHNASNGPPKAEDEYVNEPLYLNTFANTL
GKAEYLKNNILSMPEKAKKAFDNPDYWNHSLPPRSTLQHPDYLQEYSTKYFYKQNGRIRP

IVAENPEYLSEFSLKPGTVLPPPPYRHRNTVV
>hsa:2098
MALKQISSNKCFGGLQKVFEHDSVELNCKMKFAVYLPPKAETGKCPALYWLSGLTCTEQN
FISKSGYHQSASEHGLVVIAPDTSPRGCNIKGEDESWDFGTGAGFYVDATEDPWKTNYRM
YSYVTEELPQLINANFPVDPQRMSIFGHSMGGHGALICALKNPGKYKSVSAFAPICNPVL
CPWGKKAFSGYLGTDQSKWKAYDATHLVKSYPGSQLDILIDQGKDDQFLLDGQLLPDNFI
AACTEKKIPVVFRLQEGYDHSYYFIATFITDHIRHHAKYLNA
>hsa:2135
MRCCHICKLPGRVMGIRVLRLSLVVILVLLLVAGALTALLPSVKEDKMLMLRREIKSQGK
STMDSFTLIMQTYNRTDLLLKLLNHYQAVPNLHKVIVVWNNIGEKAPDELWNSLGPHPIP
VIFKQQTANRMRNRLQVFPELETNAVLMVDDDTLISTPDLVFAFSVWQQFPDQIVGFVPR
KHVSTSSGIYSYGSFEMQAPGSGNGDQYSMVLIGASFFNSKYLELFQRQPAAVHALIDDT
QNCDDIAMNFIIAKHIGKTSGIFVKPVNMDNLEKETNSGYSGMWHRAEHALQRSYCINKL
VNIYDSMPLRYSNIMISQFGFPYANYKRKI
>hsa:2147
MAHVRGLQLPGCLALAALCSLVHSQHVFLAPQQARSLLQRVRRANTFLEEVRKGNLEREC
VEETCSYEEAFEALESSTATDVFWAKYTACETARTPRDKLAACLEGNCAEGLGTNYRGHV
NITRSGIECQLWRSRYPHKPEINSTTHPGADLQENFCRNPDSSTTGPWCYTTDPTVRRQE
CSIPVCGQDQVTVAMTPRSEGSSVNLSPPLEQCVPDRGQQYQGRLAVTTHGLPCLAWASA
QAKALSKHQDFNSAVQLVENFCRNPDGDEEGVWCYVAGKPGDFGYCDLNYCEEAVEEETG
DGLDEDSDRAIEGRTATSEYQTFFNPRTFGSGEADCGLRPLFEKKSLEDKTERELLESYI
DGRIVEGSDAEIGMSPWQVMLFRKSPQELLCGASLISDRWVLTAAHCLLYPPWDKNFTEN
DLLVRIGKHSRTRYERNIEKISMLEKIYIHPRYNWRENLDRDIALMKLKKPVAFSDYIHP
VCLPDRETAASLLQAGYKGRVTGWGNLKETWTANVGKGQPSVLQVVNLPIVERPVCKDST
RIRITDNMFCAGYKPDEGKRGDACEGDSGGPFVMKSPFNNRWYQMGIVSWGEGCDRDGKY
GFYTHVFRLKKWIQKVIDQFGE
>hsa:2155
MVSQALRLLCLLLGLQGCLAAGGVAKASGGETRDMPWKPGPHRVFVTQEEAHGVLHRRRR
ANAFLEELRPGSLERECKEEQCSFEEAREIFKDAERTKLFWISYSDGDQCASSPCQNGGS
CKDQLQSYICFCLPAFEGRNCETHKDDQLICVNENGGCEQYCSDHTGTKRSCRCHEGYSL
LADGVSCTPTVEYPCGKIPILEKRNASKPQGRIVGGKVCPKGECPWQVLLLVNGAQLCGG
TLINTIWVVSAAHCFDKIKNWRNLIAVLGEHDLSEHDGDEQSRRVAQVIIPSTYVPGTTN
HDIALLRLHQPVVLTDHVVPLCLPERTFSERTLAFVRFSLVSGWGQLLDRGATALELMVL
NVPRLMTQDCLQQSRKVGDSPNITEYMFCAGYSDGSKDSCKGDSGGPHATHYRGTWYLTG
IVSWGQGCATVGHFGVYTRVSQYIEWLQKLMRSEPRPGVLLRAPFP
>hsa:2158
MQRVNMIMAESPGLITICLLGYLLSAECTVFLDHENANKILNRPKRYNSGKLEEFVQGNL
ERECMEEKCSFEEAREVFENTERTTEFWKQYVDGDQCESNPCLNGGSCKDDINSYECWCP
FGFEGKNCELDVTCNIKNGRCEQFCKNSADNKVVCSCTEGYRLAENQKSCEPAVPFPCGR
VSVSQTSKLTRAETVFPDVDYVNSTEAETILDNITQSTQSFNDFTRVVGGEDAKPGQFPW
QVVLNGKVDAFCGGSIVNEKWIVTAAHCVETGVKITVVAGEHNIEETEHTEQKRNVIRII
PHHNYNAAINKYNHDIALLELDEPLVLNSYVTPICIADKEYTNIFLKFGSGYVSGWGRVF
HKGRSALVLQYLRVPLVDRATCLRSTKFTIYNNMFCAGFHEGGRDSCQGDSGGPHVTEVE
GTSFLTGIISWGEECAMKGKYGIYTKVSRYVNWIKEKTKLT

>hsa:2159
MGRPLHLVLLSASLAGLLLLGESLFIRREQANNILARVTRANSFLEEMKKGHLERECMEE
TCSYEEAREVFEDSDKTNEFWNKYKDGDQCETSPCQNQGKCKDGLGEYTCTCLEGFEGKN
CELFTRKLCSLDNGDCDQFCHEEQNSVVCSCARGYTLADNGKACIPTGPYPCGKQTLERR
KRSVAQATSSSGEAPDSITWKPYDAADLDPTENPFDLLDFNQTQPERGDNNLTRIVGGQE
CKDGECPWQALLINEENEGFCGGTILSEFYILTAAHCLYQAKRFKVRVGDRNTEQEEGGE
AVHEVEVVIKHNRFTKETYDFDIAVLRLKTPITFRMNVAPACLPERDWAESTLMTQKTGI
VSGFGRTHEKGRQSTRLKMLEVPYVDRNSCKLSSSFIITQNMFCAGYDTKQEDACQGDSG
GPHVTRFKDTYFVTGIVSWGEGCARKGKYGIYTKVTAFLKWIDRSMKTRGLPKAKSHAPE
VITSSPLK
>hsa:216
MSSSGTPDLPVLLTDLKIQYTKIFINNEWHDSVSGKKFPVFNPATEEELCQVEEGDKEDV
DKAVKAARQAFQIGSPWRTMDASERGRLLYKLADLIERDRLLLATMESMNGGKLYSNAYL
NDLAGCIKTLRYCAGWADKIQGRTIPIDGNFFTYTRHEPIGVCGQIIPWNFPLVMLIWKI
GPALSCGNTVVVKPAEQTPLTALHVASLIKEAGFPPGVVNIVPGYGPTAGAAISSHMDID
KVAFTGSTEVGKLIKEAAGKSNLKRVTLELGGKSPCIVLADADLDNAVEFAHHGVFYHQG
QCCIAASRIFVEESIYDEFVRRSVERAKKYILGNPLTPGVTQGPQIDKEQYDKILDLIES
GKKEGAKLECGGGPWGNKGYFVQPTVFSNVTDEMRIAKEEIFGPVQQIMKFKSLDDVIKR
ANNTFYGLSAGVFTKDIDKAITISSALQAGTVWVNCYGVVSAQCPFGGFKMSGNGRELGE
YGFHEYTEVKTVTVKISQKNS
>hsa:2160
MIFLYQVVHFILFTSVSGECVTQLLKDTCFEGGDITTVFTPSAKYCQVVCTYHPRCLLFT
FTAESPSEDPTRWFTCVLKDSVTETLPRVNRTAAISGYSFKQCSHQISACNKDIYVDLDM
KGINYNSSVAKSAQECQERCTDDVHCHFFTYATRQFPSLEHRNICLLKHTQTGTPTRITK
LDKVVSGFSLKSCALSNLACIRDIFPNTVFADSNIDSVMAPDAFVCGRICTHHPGCLFFT
FFSQEWPKESQRNLCLLKTSESGLPSTRIKKSKALSGFSLQSCRHSIPVFCHSSFYHDTD
FLGEELDIVAAKSHEACQKLCTNAVRCQFFTYTPAQASCNEGKGKCYLKLSSNGSPTKIL
HGRGGISGYTLRLCKMDNECTTKIKPRIVGGTASVRGEWPWQVTLHTTSPTQRHLCGGSI
IGNQWILTAAHCFYGVESPKILRVYSGILNQSEIKEDTSFFGVQEIIIHDQYKMAESGYD
IALLKLETTVNYTDSQRPICLPSKGDRNVIYTDCWVTGWGYRKLRDKIQNTLQKAKIPLV
TNEECQKRYRGHKITHKMICAGYREGGKDACKGDSGGPLSCKHNEVWHLVGITSWGEGCA
QRERPGVYTNVVEYVDWILEKTQAV
>hsa:2161
MRALLLLGFLLVSLESTLSIPPWEAPKEHKYKAEEHTVVLTVTGEPCHFPFQYHRQLYHK
CTHKGRPGPQPWCATTPNFDQDQRWGYCLEPKKVKDHCSKHSPCQKGGTCVNMPSGPHCL
CPQHLTGNHCQKEKCFEPQLLRFFHKNEIWYRTEQAAVARCQCKGPDAHCQRLASQACRT
NPCLHGGRCLEVEGHRLCHCPVGYTGAFCDVDTKASCYDGRGLSYRGLARTTLSGAPCQP
WASEATYRNVTAEQARNWGLGGHAFCRNPDNDIRPWCFVLNRDRLSWEYCDLAQCQTPTQ
AAPPTPVSPRLHVPLMPAQPAPPKPQPTTRTPPQSQTPGALPAKREQPPSLTRNGPLSCG
QRLRKSLSSMTRVVGGLVALRGAHPYIAALYWGHSFCAGSLIAPCWVLTAAHCLQDRPAP
EDLTVVLGQERRNHSCEPCQTLAVRSYRLHEAFSPVSYQHDLALLRLQEDADGSCALLSP
YVQPVCLPSGAARPSETTLCQVAGWGHQFEGAEEYASFLQEAQVPFLSLERCSAPDVHGS
SILPGMLCAGFLEGGTDACQGDSGGPLVCEDQAAERRLTLQGIISWGSGCGDRNKPGVYT
DVAYYLAWIREHTVS

>hsa:217
MLRAAARFGPRLGRRLLSAAATQAVPAPNQQPEVFCNQIFINNEWHDAVSRKTFPTVNPS
TGEVICQVAEGDKEDVDKAVKAARAAFQLGSPWRRMDASHRGRLLNRLADLIERDRTYLA
ALETLDNGKPYVISYLVDLDMVLKCLRYYAGWADKYHGKTIPIDGDFFSYTRHEPVGVCG
QIIPWNFPLLMQAWKLGPALATGNVVVMKVAEQTPLTALYVANLIKEAGFPPGVVNIVPG
FGPTAGAAIASHEDVDKVAFTGSTEIGRVIQVAAGSSNLKRVTLELGGKSPNIIMSDADM
DWAVEQAHFALFFNQGQCCCAGSRTFVQEDIYDEFVERSVARAKSRVVGNPFDSKTEQGP
QVDETQFKKILGYINTGKQEGAKLLCGGGIAADRGYFIQPTVFGDVQDGMTIAKEEIFGP
VMQILKFKTIEEVVGRANNSTYGLAAAVFTKDLDKANYLSQALQAGTVWVNCYDVFGAQS
PFGGYKMSGSGRELGEYGLQAYTEVKTVTVKVPQKNS
>hsa:218
MSKISEAVKRARAAFSSGRTRPLQFRIQQLEALQRLIQEQEQELVGALAADLHKNEWNAY
YEEVVYVLEEIEYMIQKLPEWAADEPVEKTPQTQQDELYIHSEPLGVVLVIGTWNYPFNL
TIQPMVGAIAAGNSVVLKPSELSENMASLLATIIPQYLDKDLYPVINGGVPETTELLKER
FDHILYTGSTGVGKIIMTAAAKHLTPVTLELGGKSPCYVDKNCDLDVACRRIAWGKFMNS
GQTCVAPDYILCDPSIQNQIVEKLKKSLKEFYGEDAKKSRDYGRIISARHFQRVMGLIEG
QKVAYGGTGDAATRYIAPTILTDVDPQSPVMQEEIFGPVLPIVCVRSLEEAIQFINQREK
PLALYMFSSNDKVIKKMIAETSSGGVAANDVIVHITLHSLPFGGVGNSGMGSYHGKKSFE
TFSHRRSCLVRPLMNDEGLKVRYPPSPAKMTQH
>hsa:2180
MQAHELFRYFRMPELVDFRQYVRTLPTNTLMGFGAFAALTTFWYATRPKPLKPPCDLSMQ
SVEVAGSGGARRSALLDSDEPLVYFYDDVTTLYEGFQRGIQVSNNGPCLGSRKPDQPYEW
LSYKQVAELSECIGSALIQKGFKTAPDQFIGIFAQNRPEWVIIEQGCFAYSMVIVPLYDT
LGNEAITYIVNKAELSLVFVDKPEKAKLLLEGVENKLIPGLKIIVVMDAYGSELVERGQR
CGVEVTSMKAMEDLGRANRRKPKPPAPEDLAVICFTSGTTGNPKGAMVTHRNIVSDCSAF
VKATENTVNPCPDDTLISFLPLAHMFERVVECVMLCHGAKIGFFQGDIRLLMDDLKVLQP
TVFPVVPRLLNRMFDRIFGQANTTLKRWLLDFASKRKEAELRSGIIRNNSLWDRLIFHKV
QSSLGGRVRLMVTGAAPVSATVLTFLRAALGCQFYEGYGQTECTAGCCLTMPGDWTAGHV
GAPMPCNLIKLVDVEEMNYMAAEGEGEVCVKGPNVFQGYLKDPAKTAEALDKDGWLHTGD
IGKWLPNGTLKIIDRKKHIFKLAQGEYIAPEKIENIYMRSEPVAQVFVHGESLQAFLIAI
VVPDVETLCSWAQKRGFEGSFEELCRNKDVKKAILEDMVRLGKDSGLKPFEQVKGITLHP
ELFSIDNGLLTPTMKAKRPELRNYFRSQIDDLYSTIKV
>hsa:2185
MSGVSEPLSRVKLGTLRRPEGPAEPMVVVPVDVEKEDVRILKVCFYSNSFNPGKNFKLVK
CTVQTEIREIITSILLSGRIGPNIRLAECYGLRLKHMKSDEIHWLHPQMTVGEVQDKYEC
LHVEAEWRYDLQIRYLPEDFMESLKEDRTTLLYFYQQLRNDYMQRYASKVSEGMALQLGC
LELRRFFKDMPHNALDKKSNFELLEKEVGLDLFFPKQMQENLKPKQFRKMIQQTFQQYAS
LREEECVMKFFNTLAGFANIDQETYRCELIQGWNITVDLVIGPKGIRQLTSQDAKPTCLA
EFKQIRSIRCLPLEEGQAVLQLGIEGAPQALSIKTSSLAEAENMADLIDGYCRLQGEHQG
SLIIHPRKDGEKRNSLPQIPMLNLEARRSHLSESCSIESDIYAEIPDETLRRPGGPQYGI
AREDVVLNRILGEGFFGEVYEGVYTNHKGEKINVAVKTCKKDCTLDNKEKFMSEAVIMKN
LDHPHIVKLIGIIEEEPTWIIMELYPYGELGHYLERNKNSLKVLTLVLYSLQICKAMAYL
ESINCVHRDIAVRNILVASPECVKLGDFGLSRYIEDEDYYKASVTRLPIKWMSPESINFR
RFTTASDVWMFAVCMWEILSFGKQPFFWLENKDVIGVLEKGDRLPKPDLCPPVLYTLMTR

CWDYDPSDRPRFTELVCSLSDVYQMEKDIAMEQERNARYRTPKILEPTAFQEPPPKPSRP
KYRPPPQTNLLAPKLQFQVPEGLCASSPTLTSPMEYPSPVNSLHTPPLHRHNVFKRHSMR
EEDFIQPSSREEAQQLWEAEKVKMRQILDKQQKQMVEDYQWLRQEEKSLDPMVYMNDKSP
LTPEKEVGYLEFTGPPQKPPRLGAQSIQPTANLDRTDDLVYLNVMELVRAVLELKNELCQ
LPPEGYVVVVKNVGLTLRKLIGSVDDLLPSLPSSSRTEIEGTQKLLNKDLAELINKMRLA
QQNAVTSLSEECKRQMLTASHTLAVDAKNLLDAVDQAKVLANLAHPPAE
>hsa:219
MLRFLAPRLLSLQGRTARYSSAAALPSPILNPDIPYNQLFINNEWQDAVSKKTFPTVNPT
TGEVIGHVAEGDRADVDRAVKAAREAFRLGSPWRRMDASERGRLLNRLADLVERDRVYLA
SLETLDNGKPFQESYALDLDEVIKVYRYFAGWADKWHGKTIPMDGQHFCFTRHEPVGVCG
QIIPWNFPLVMQGWKLAPALATGNTVVMKVAEQTPLSALYLASLIKEAGFPPGVVNIITG
YGPTAGAAIAQHVDVDKVAFTGSTEVGHLIQKAAGDSNLKRVTLELGGKSPSIVLADADM
EHAVEQCHEALFFNMGQCCCAGSRTFVEESIYNEFLERTVEKAKQRKVGNPFELDTQQGP
QVDKEQFERVLGYIQLGQKEGAKLLCGGERFGERGFFIKPTVFGGVQDDMRIAKEEIFGP
VQPLFKFKKIEEVVERANNTRYGLAAAVFTRDLDKAMYFTQALQAGTVWVNTYNIVTCHT
PFGGFKESGNGRELGEDGLKAYTEVKTVTIKVPQKNS
>hsa:2193
MADGQVAELLLRRLEASDGGLDSAELAAELGMEHQAVVGAVKSLQALGEVIEAELRSTKH
WELTAEGEEIAREGSHEARVFRSIPPEGLAQSELMRLPSGKVGFSKAMSNKWIRVDKSAA
DGPRVFRVVDSMEDEVQRRLQLVRGGQAEKLGEKERSELRKRKLLAEVTLKTYWVSKGSA
FSTSISKQETELSPEMISSGSWRDRPFKPYNFLAHGVLPDSGHLHPLLKVRSQFRQIFLE
MGFTEMPTDNFIESSFWNFDALFQPQQHPARDQHDTFFLRDPAEALQLPMDYVQRVKRTH
SQGGYGSQGYKYNWKLDEARKNLLRTHTTSASARALYRLAQKKPFTPVKYFSIDRVFRNE
TLDATHLAEFHQIEGVVADHGLTLGHLMGVLREFFTKLGITQLRFKPAYNPYTEPSMEVF
SYHQGLKKWVEVGNSGVFRPEMLLPMGLPENVSVIAWGLSLERPTMIKYGINNIRELVGH
KVNLQMVYDSPLCRLDAEPRPPPTQEAA
>hsa:2224
MPLSRWLRSVGVFLLPAPYWAPRERWLGSLRRPSLVHGYPVLAWHSARCWCQAWTEEPRA
LCSSLRMNGDQNSDVYAQEKQDFVQHFSQIVRVLTEDEMGHPEIGDAIARLKEVLEYNAI
GGKYNRGLTVVVAFRELVEPRKQDADSLQRAWTVGWCVELLQAFFLVADDIMDSSLTRRG
QICWYQKPGVGLDAINDANLLEACIYRLLKLYCREQPYYLNLIELFLQSSYQTEIGQTLD
LLTAPQGNVDLVRFTEKRYKSIVKYKTAFYSFYLPIAAAMYMAGIDGEKEHANAKKILLE
MGEFFQIQDDYLDLFGDPSVTGKIGTDIQDNKCSWLVVQCLQRATPEQYQILKENYGQKE
AEKVARVKALYEELDLPAVFLQYEEDSYSHIMALIEQYAAPLPPAVFLGLARKIYKRRK
>hsa:223
MFLRAGLAALSPLLRSLRPSPVAAMSTGTFVVSQPLNYRGGARVEPADASGTEKAFEPAT
GRVIATFTCSGEKEVNLAVQNAKAAFKIWSQKSGMERCRILLEAARIIREREDEIATMEC
INNGKSIFEARLDIDISWQCLEYYAGLAASMAGEHIQLPGGSFGYTRREPLGVCVGIGAW
NYPFQIASWKSAPALACGNAMVFKPSPFTPVSALLLAEIYSEAGVPPGLFNVVQGGAATG
QFLCQHPDVAKVSFTGSVPTGMKIMEMSAKGIKPVTLELGGKSPLIIFSDCDMNNAVKGA
LMANFLTQGQVCCNGTRVFVQKEILDKFTEEVVKQTQRIKIGDPLLEDTRMGPLINRPHL
ERVLGFVKVAKEQGAKVLCGGDIYVPEDPKLKDGYYMRPCVLTNCRDDMTCVKEEIFGPV
MSILSFDTEAEVLERANDTTFGLAAGVFTRDIQRAHRVVAELQAGTCFINNYNVSPVELP
FGGYKKSGFGRENGRVTIEYYSQLKTVCVEMGDVESAF

>hsa:2232
MASRCWRWWGWSAWPRTRLPPAGSTPSFCHHFSTQEKTPQICVVGSGPAGFYTAQHLLKR
VEALCSQPRVLNSPALSGEGEDLGASQPLSLDPTSCHPVPQQHPQAHVDIYEKQPVPFGL
VRFGVAPDHPEVKNVINTFTQTAHSGRCAFWGNVEVGRDVTVPELREAYHAVVLSYGAED
HRALEIPGEELPGVCSARAFVGWYNGLPENQELEPDLSCDTAVILGQGNVALDVARILLT
PPEHLERTDITKAALGVLRQSRVKTVWLVGRRGPLQVAFTIKELREMIQLPGARPILDPV
DFLGLQDKIKEVPRPRKRLTELLLRTATEKPGPAEAARQASASRAWGLRFFRSPQQVLPS
PDGRRAAGVRLAVTRLEGVDEATRAVPTGDMEDLPCGLVLSSIGYKSRPVDPSVPFDSKL
GVIPNVEGRVMDVPGLYCSGWVKRGPTGVIATTMTDSFLTGQMLLQDLKAGLLPSGPRPG
YAAIQALLSSRGVRPVSFSDWEKLDAEEVARGQGTGKPREKLVDPQEMLRLLGH
>hsa:2235
MRSLGANMAAALRAAGVLLRDPLASSSWRVCQPWRWKSGAAAAAVTTETAQHAQGAKPQV
QPQKRKPKTGILMLNMGGPETLGDVHDFLLRLFLDRDLMTLPIQNKLAPFIAKRRTPKIQ
EQYRRIGGGSPIKIWTSKQGEGMVKLLDELSPNTAPHKYYIGFRYVHPLTEEAIEEMERD
GLERAIAFTQYPQYSCSTTGSSLNAIYRYYNQVGRKPTMKWSTIDRWPTHHLLIQCFADH
ILKELDHFPLEKRSEVVILFSAHSLPMSVVNRGDPYPQEVSATVQKVMERLEYCNPYRLV
WQSKVGPMPWLGPQTDESIKGLCERGRKNILLVPIAFTSDHIETLYELDIEYSQVLAKEC
GVENIRRAESLNGNPLFSKALADLVHSHIQSNELCSKQLTLSCPLCVNPVCRETKSFFTS
QQL
>hsa:224
MELEVRRVRQAFLSGRSRPLRFRLQQLEALRRMVQEREKDILTAIAADLCKSEFNVYSQE
VITVLGEIDFMLENLPEWVTAKPVKKNVLTMLDEAYIQPQPLGVVLIIGAWNYPFVLTIQ
PLIGAIAAGNAVIIKPSELSENTAKILAKLLPQYLDQDLYIVINGGVEETTELLKQRFDH
IFYTGNTAVGKIVMEAAAKHLTPVTLELGGKSPCYIDKDCDLDIVCRRITWGKYMNCGQT
CIAPDYILCEASLQNQIVWKIKETVKEFYGENIKESPDYERIINLRHFKRILSLLEGQKI
AFGGETDEATRYIAPTVLTDVDPKTKVMQEEIFGPILPIVPVKNVDEAINFINEREKPLA
LYVFSHNHKLIKRMIDETSSGGVTGNDVIMHFTLNSFPFGGVGSSGMGAYHGKHSFDTFS
HQRPCLLKSLKREGANKLRYPPNSQSKVDWGKFFLLKRFNKEKLGLLLLTFLGIVAAVLV
KAEYY
>hsa:2241
MGFGSDLKNSHEAVLKLQDWELRLLETVKKFMALRIKSDKEYASTLQNLCNQVDKESTVQ
MNYVSNVSKSWLLMIQQTEQLSRIMKTHAEDLNSGPLHRLTMMIKDKQQVKKSYIGVHQQ
IEAEMIKVTKTELEKLKCSYRQLIKEMNSAKEKYKEALAKGKETEKAKERYDKATMKLHM
LHNQYVLALKGAQLHQNQYYDITLPLLLDSLQKMQEEMIKALKGIFDEYSQITSLVTEEI
VNVHKEIQMSVEQIDPSTEYNNFIDVHRTTAAKEQEIEFDTSLLEENENLQANEIMWNNL
TAESLQVMLKTLAEELMQTQQMLLNKEEAVLELEKRIEESSETCEKKSDIVLLLSQKQAL
EELKQSVQQLRCTEAKFSAQKELLEQKVQENDGKEPPPVVNYEEDARSVTSMERKERLSK
FESIRHSIAGIIRSPKSALGSSALSDMISISEKPLAEQDWYHGAIPRIEAQELLKKQGDF
LVRESHGKPGEYVLSVYSDGQRRHFIIQYVDNMYRFEGTGFSNIPQLIDHHYTTKQVITK
KSGVVLLNPIPKDKKWILSHEDVILGELLGKGNFGEVYKGTLKDKTSVAVKTCKEDLPQE
LKIKFLQEAKILKQYDHPNIVKLIGVCTQRQPVYIIMELVSGGDFLTFLRRKKDELKLKQ
LVKFSLDAAAGMLYLESKNCIHRDLAARNCLVGENNVLKISDFGMSRQEDGGVYSSSGLK
QIPIKWTAPEALNYGRYSSESDVWSFGILLWETFSLGVCPYPGMTNQQAREQVERGYRMS
APQHCPEDISKIMMKCWDYKPENRPKFSELQKELTIIKRKLT

>hsa:2242
MGFSSELCSPQGHGVLQQMQEAELRLLEGMRKWMAQRVKSDREYAGLLHHMSLQDSGGQS
RAISPDSPISQTHSQDIEKLKSQYRALARDSAQAKRKYQEASKDKDRDKAKDKYVRSLWK
LFAHHNRYVLGVRAAQLHHQHHHQLLLPGLLRSLQDLHEEMACILKEILQEYLEISSLVQ
DEVVAIHREMAAAAARIQPEAEYQGFLRQYGSAPDVPPCVTFDESLLEEGEPLEPGELQL
NELTVESVQHTLTSVTDELAVATEMVFRRQEMVTQLQQELRNEEENTHPRERVQLLGKRQ
VLQEALQGLQVALCSQAKLQAQQELLQTKLEHLGPGEPPPVLLLQDDRHSTSSSEQEREG
GRTPTLEILKSHISGIFRPKFSLPPPLQLIPEVQKPLHEQLWYHGAIPRAEVAELLVHSG
DFLVRESQGKQEYVLSVLWDGLPRHFIIQSLDNLYRLEGEGFPSIPLLIDHLLSTQQPLT
KKSGVVLHRAVPKDKWVLNHEDLVLGEQIGRGNFGEVFSGRLRADNTLVAVKSCRETLPP
DLKAKFLQEARILKQYSHPNIVRLIGVCTQKQPIYIVMELVQGGDFLTFLRTEGARLRVK
TLLQMVGDAAAGMEYLESKCCIHRDLAARNCLVTEKNVLKISDFGMSREEADGVYAASGG
LRQVPVKWTAPEALNYGRYSSESDVWSFGILLWETFSLGASPYPNLSNQQTREFVEKGGR
LPCPELCPDAVFRLMEQCWAYEPGQRPSFSTIYQELQSIRKRHR
>hsa:225689
MCTVVDPRIVRRYLLRRQLGQGAYGIVWKAVDRRTGEVVAIKKIFDAFRDKTDAQRTFRE
ITLLQEFGDHPNIISLLDVIRAENDRDIYLVFEFMDTDLNAVIRKGGLLQDVHVRSIFYQ
LLRATRFLHSGHVVHRDQKPSNVLLDANCTVKLCDFGLARSLGDLPEGPEDQAVTEYVAT
RWYRAPEVLLSSHRYTLGVDMWSLGCILGEMLRGRPLFPGTSTLHQLELILETIPPPSEE
DLLALGSGCRASVLHQLGSRPRQTLDALLPPDTSPEALDLLRRLLVFAPDKRLSATQALQ
HPYVQRFHCPSDEWAREADVRPRAHEGVQLSVPEYRSRVYQMILECGGSSGTSREKGPEG
VSPSQAHLHKPRADPQLPSRTPVQGPRPRPQSSPGHDPAEHESPRAAKNVPRQNSAPLLQ
TALLGNGERPPGAKEAPPLTLSLVKPSGRGAAPSLTSQAAAQVANQALIRGDWNRGGGVR
VASVQQVPPRLPPEARPGRRMFSTSALQGAQGGARALLGGYSQAYGTVCHSALGHLPLLE
GHHV
>hsa:2260
MWSWKCLLFWAVLVTATLCTARPSPTLPEQAQPWGAPVEVESFLVHPGDLLQLRCRLRDD
VQSINWLRDGVQLAESNRTRITGEEVEVQDSVPADSGLYACVTSSPSGSDTTYFSVNVSD
ALPSSEDDDDDDDSSSEEKETDNTKPNRMPVAPYWTSPEKMEKKLHAVPAAKTVKFKCPS
SGTPNPTLRWLKNGKEFKPDHRIGGYKVRYATWSIIMDSVVPSDKGNYTCIVENEYGSIN
HTYQLDVVERSPHRPILQAGLPANKTVALGSNVEFMCKVYSDPQPHIQWLKHIEVNGSKI
GPDNLPYVQILKTAGVNTTDKEMEVLHLRNVSFEDAGEYTCLAGNSIGLSHHSAWLTVLE
ALEERPAVMTSPLYLEIIIYCTGAFLISCMVGSVIVYKMKSGTKKSDFHSQMAVHKLAKS
IPLRRQVSADSSASMNSGVLLVRPSRLSSSGTPMLAGVSEYELPEDPRWELPRDRLVLGK
PLGEGCFGQVVLAEAIGLDKDKPNRVTKVAVKMLKSDATEKDLSDLISEMEMMKMIGKHK
NIINLLGACTQDGPLYVIVEYASKGNLREYLQARRPPGLEYCYNPSHNPEEQLSSKDLVS
CAYQVARGMEYLASKKCIHRDLAARNVLVTEDNVMKIADFGLARDIHHIDYYKKTTNGRL
PVKWMAPEALFDRIYTHQSDVWSFGVLLWEIFTLGGSPYPGVPVEELFKLLKEGHRMDKP
SNCTNELYMMMRDCWHAVPSQRPTFKQLVEDLDRIVALTSNQEYLDLSMPLDQYSPSFPD
TRSSTCSSGEDSVFSHEPLPEEPCLPRHPAQLANGGLKRR
>hsa:2261
MGAPACALALCVAVAIVAGASSESLGTEQRVVGRAAEVPGPEPGQQEQLVFGSGDAVELS
CPPPGGGPMGPTVWVKDGTGLVPSERVLVGPQRLQVLNASHEDSGAYSCRQRLTQRVLCH
FSVRVTDAPSSGDDEDGEDEAEDTGVDTGAPYWTRPERMDKKLLAVPAANTVRFRCPAAG

NPTPSISWLKNGREFRGEHRIGGIKLRHQQWSLVMESVVPSDRGNYTCVVENKFGSIRQT
YTLDVLERSPHRPILQAGLPANQTAVLGSDVEFHCKVYSDAQPHIQWLKHVEVNGSKVGP
DGTPYVTVLKTAGANTTDKELEVLSLHNVTFEDAGEYTCLAGNSIGFSHHSAWLVVLPAE
EELVEADEAGSVYAGILSYGVGFFLFILVVAAVTLCRLRSPPKKGLGSPTVHKISRFPLK
RQVSLESNASMSSNTPLVRIARLSSGEGPTLANVSELELPADPKWELSRARLTLGKPLGE
GCFGQVVMAEAIGIDKDRAAKPVTVAVKMLKDDATDKDLSDLVSEMEMMKMIGKHKNIIN
LLGACTQGGPLYVLVEYAAKGNLREFLRARRPPGLDYSFDTCKPPEEQLTFKDLVSCAYQ
VARGMEYLASQKCIHRDLAARNVLVTEDNVMKIADFGLARDVHNLDYYKKTTNGRLPVKW
MAPEALFDRVYTHQSDVWSFGVLLWEIFTLGGSPYPGIPVEELFKLLKEGHRMDKPANCT
HDLYMIMRECWHAAPSQRPTFKQLVEDLDRVLTVTSTDEYLDLSAPFEQYSPGGQDTPSS
SSSGDDSVFAHDLLPPAPPSSGGSRT
>hsa:2263
MVSWGRFICLVVVTMATLSLARPSFSLVEDTTLEPEEPPTKYQISQPEVYVAAPGESLEV
RCLLKDAAVISWTKDGVHLGPNNRTVLIGEYLQIKGATPRDSGLYACTASRTVDSETWYF
MVNVTDAISSGDDEDDTDGAEDFVSENSNNKRAPYWTNTEKMEKRLHAVPAANTVKFRCP
AGGNPMPTMRWLKNGKEFKQEHRIGGYKVRNQHWSLIMESVVPSDKGNYTCVVENEYGSI
NHTYHLDVVERSPHRPILQAGLPANASTVVGGDVEFVCKVYSDAQPHIQWIKHVEKNGSK
YGPDGLPYLKVLKAAGVNTTDKEIEVLYIRNVTFEDAGEYTCLAGNSIGISFHSAWLTVL
PAPGREKEITASPDYLEIAIYCIGVFLIACMVVTVILCRMKNTTKKPDFSSQPAVHKLTK
RIPLRRQVTVSAESSSSMNSNTPLVRITTRLSSTADTPMLAGVSEYELPEDPKWEFPRDK
LTLGKPLGEGCFGQVVMAEAVGIDKDKPKEAVTVAVKMLKDDATEKDLSDLVSEMEMMKM
IGKHKNIINLLGACTQDGPLYVIVEYASKGNLREYLRARRPPGMEYSYDINRVPEEQMTF
KDLVSCTYQLARGMEYLASQKCIHRDLAARNVLVTENNVMKIADFGLARDINNIDYYKKT
TNGRLPVKWMAPEALFDRVYTHQSDVWSFGVLMWEIFTLGGSPYPGIPVEELFKLLKEGH
RMDKPANCTNELYMMMRDCWHAVPSQRPTFKQLVEDLDRILTLTTNEEYLDLSQPLEQYS
PSYPDTRSSCSSGDDSVFSPDPMPYEPCLPQYPHINGSVKT
>hsa:2264
MRLLLALLGVLLSVPGPPVLSLEASEEVELEPCLAPSLEQQEQELTVALGQPVRLCCGRA
ERGGHWYKEGSRLAPAGRVRGWRGRLEIASFLPEDAGRYLCLARGSMIVLQNLTLITGDS
LTSSNDDEDPKSHRDPSNRHSYPQQAPYWTHPQRMEKKLHAVPAGNTVKFRCPAAGNPTP
TIRWLKDGQAFHGENRIGGIRLRHQHWSLVMESVVPSDRGTYTCLVENAVGSIRYNYLLD
VLERSPHRPILQAGLPANTTAVVGSDVELLCKVYSDAQPHIQWLKHIVINGSSFGADGFP
YVQVLKTADINSSEVEVLYLRNVSAEDAGEYTCLAGNSIGLSYQSAWLTVLPEEDPTWTA
AAPEARYTDIILYASGSLALAVLLLLAGLYRGQALHGRHPRPPATVQKLSRFPLARQFSL
ESGSSGKSSSSLVRGVRLSSSGPALLAGLVSLDLPLDPLWEFPRDRLVLGKPLGEGCFGQ
VVRAEAFGMDPARPDQASTVAVKMLKDNASDKDLADLVSEMEVMKLIGRHKNIINLLGVC
TQEGPLYVIVECAAKGNLREFLRARRPPGPDLSPDGPRSSEGPLSFPVLVSCAYQVARGM
QYLESRKCIHRDLAARNVLVTEDNVMKIADFGLARGVHHIDYYKKTSNGRLPVKWMAPEA
LFDRVYTHQSDVWSFGILLWEIFTLGGSPYPGIPVEELFSLLREGHRMDRPPHCPPELYG
LMRECWHAAPSQRPTFKQLVEALDKVLLAVSEEYLDLRLTFGPYSPSGGDASSTCSSSDS
VFSHDPLPLGSSSFPFGSGVQT
>hsa:2280
MGVQVETISPGDGRTFPKRGQTCVVHYTGMLEDGKKFDSSRDRNKPFKFMLGKQEVIRGW
EEGVAQMSVGQRAKLTISPDYAYGATGHPGIIPPHATLVFDVELLKLE

>hsa:22843
MAGCIPEEKTYRRFLELFLGEFRGPCGGGEPEPEPEPEPEPEPESEPEPEPELVEAEAAE
ASVEEPGEEAATVAATEEGDQEQDPEPEEEAAVEGEEEEEGAATAAAAPGHSAVPPPPPQ
LPPLPPLPRPLSERITREEVEGESLDLCLQQLYKYNCPSFLAAALARATSDEVLQSDLSA
HYIPKETDGTEGTVEIETVKLARSVFSKLHEICCSWVKDFPLRRRPQLYYETSIHAIKNM
RRKMEDKHVCIPDFNMLFNLEDQEEQAYFAVFDGHGGVDAAIYASIHLHVNLVRQEMFPH
DPAEALCRAFRVTDERFVQKAARESLRCGTTGVVTFIRGNMLHVAWVGDSQVMLVRKGQA
VELMKPHKPDREDEKQRIEALGGCVVWFGAWRVNGSLSVSRAIGDAEHKPYICGDADSAS
TVLDGTEDYLILACDGFYDTVNPDEAVKVVSDHLKENNGDSSMVAHKLVASARDAGSSDN
ITVIVVFLRDMNKAVNVSEESDWTENSFQGGQEDGGDDKENHGECKRPWPQHQCSAPADL
GYDGRVDSFTDRTSLSPGSQINVLEDPGYLDLTQIEASKPHSAQFLLPVEMFGPGAPKKA
NLINELMMEKKSVQSSLPEWSGAGEFPTAFNLGSTGEQIYRMQSLSPVCSGLENEQFKSP
GNRVSRLSHLRHHYSKKWHRFRFNPKFYSFLSAQEPSHKIGTSLSSLTGSGKRNRIRSSL
PWRQNSWKGYSENMRKLRKTHDIPCPDLPWSYKIE
>hsa:22954
MAAAAASHLNLDALREVLECPICMESFTEEQLRPKLLHCGHTICRQCLEKLLASSINGVR
CPFCSKITRITSLTQLTDNLTVLKIIDTAGLSEAVGLLMCRSCGRRLPRQFCRSCGLVLC
EPCREADHQPPGHCTLPVKEAAEERRRDFGEKLTRLRELMGELQRRKAALEGVSKDLQAR
YKAVLQEYGHEERRVQDELARSRKFFTGSLAEVEKSNSQVVEEQSYLLNIAEVQAVSRCD
YFLAKIKQADVALLEETADEEEPELTASLPRELTLQDVELLKVGHVGPLQIGQAVKKPRT
VNVEDSWAMEATASAASTSVTFREMDMSPEEVVASPRASPAKQRGPEAASNIQQCLFLKK
MGAKGSTPGMFNLPVSLYVTSQGEVLVADRGNYRIQVFTRKGFLKEIRRSPSGIDSFVLS
FLGADLPNLTPLSVAMNCQGLIGVTDSYDNSLKVYTLDGHCVACHRSQLSKPWGITALPS
GQFVVTDVEGGKLWCFTVDRGSGVVKYSCLCSAVRPKFVTCDAEGTVYFTQGLGLNLENR
QNEHHLEGGFSIGSVGPDGQLGRQISHFFSENEDFRCIAGMCVDARGDLIVADSSRKEIL
HFPKGGGYSVLIREGLTCPVGIALTPKGQLLVLDCWDHCIKIYSYHLRRYSTP
>hsa:22978
MSTSWSDRLQNAADMPANMDKHALKKYRREAYHRVFVNRSLAMEKIKCFGFDMDYTLAVY
KSPEYESLGFELTVERLVSIGYPQELLSFAYDSTFPTRGLVFDTLYGNLLKVDAYGNLLV
CAHGFNFIRGPETREQYPNKFIQRDDTERFYILNTLFNLPETYLLACLVDFFTNCPRYTS
CETGFKDGDLFMSYRSMFQDVRDAVDWVHYKGSLKEKTVENLEKYVVKDGKLPLLLSRMK
EVGKVFLATNSDYKYTDKIMTYLFDFPHGPKPGSSHRPWQSYFDLILVDARKPLFFGEGT
VLRQVDTKTGKLKIGTYTGPLQHGIVYSGGSSDTICDLLGAKGKDILYIGDHIFGDILKS
KKRQGWRTFLVIPELAQELHVWTDKSSLFEELQSLDIFLAELYKHLDSSSNERPDISSIQ
RRIKKVTHDMDMCYGMMGSLFRSGSRQTLFASQVMRYADLYAASFINLLYYPFSYLFRAA
HVLMPHESTVEHTHVDINEMESPLATRNRTSVDFKDTDYKRHQLTRSISEIKPPNLFPLA
PQEITHCHDEDDDEEEEEEEE
>hsa:23035
MKRNGSRNCLNRRSRFGSRERDWLREDVKRGCVYLYGADTTTATTTTTSSSSSSSSSS
DLHLVLCTVETPASEICAGEGRESLYLQLHGDLVRRLEPTERPLQIVYDYLSRLGFDDPV
RIQEEATNPDLGCMIRFYGEKPCHMDRLDRILLSGIYNVRKGKTQLHKWAERLVVLCGTC
LIVSSVKDCQTGKMHILPLVGGKIEEVKRRQYSLAFSSAGAQAQTYHVSFETLAEYQRWQ
RQASKVVSQRISTVDLSCYSLEEVPEHLFYSQDITYLNLRHNFMQLERPGGLDTLYKFSQ
LKGLNLSHNKLGLFPILLCEISTLTELNLSCNGFHDLPSQIGNLLNLQTLCLDGNFLTTL

PEELGNLQQLSSLGISFNNFSQIPEVYEKLTMLDRVVMAGNCLEVLNLGVLNRMNHIKHV
DLRMNHLKTMVIENLEGNKHITHVDLRDNRLTDLDLSSLCSLEQLHCGRNQLRELTLSGF
SLRTLYASSNRLTAVNVYPVPSLLTFLDLSRNLLECVPDWACEAKKIEVLDVSYNLLTEV
PVRILSSLSLRKLMLGHNHVQNLPTLVEHIPLEVLDLQHNALTRLPDTLFSKALNLRYLN
ASANSLESLPSACTGEESLSMLQLLYLTNNLLTDQCIPVLVGHLHLRILHLANNQLQTFP
ASKLNKLEQLEELNLSGNKLKTIPTTIANCKRLHTLVAHSNNISIFPEILQLPQIQFVDL
SCNDLTEILIPEALPATLQDLDLTGNTNLVLEHKTLDIFSHITTLKIDQKPLPTTDSTVT
STFWSHGLAEMAGQRNKLCVSALAMDSFAEGVGAVYGMFDGDRNEELPRLLQCTMADVLL
EEVQQSTNDTVFMANTFLVSHRKLGMAGQKLGSSALLCYIRPDTADPASSFSLTVANVGT
CQAVLCRGGKPVPLSKVFSLEQDPEEAQRVKDQKAIITEDNKVNGVTCCTRMLGCTYLYP
WILPKPHISSTPLTIQDELLILGNKALWEHLSYTEAVNAVRHVQDPLAAAKKLCTLAQSY
GCQDNVGAMVVYLNIGEEGCTCEMNGLTLPGPVGFASTTTIKDAPKPATPSSSSGIASEF
SSEMSTSEVSSEVGSTASDEHNAGGLDTALLPRPERRCSLHPTPTSGLFQRQPSSATFSS
NQSDNGLDSDDDQPVEGVITNGSKVEVEVDIHCCRGRDLENSPPLIESSPTLCSEEHARG
SCFGIRRQNSVNSGMLLPMSKDRMELQKSPSTSCLYGKKLSNGSIVPLEDSLNLIEVATE
VPKRKTGYFAAPTQMEPEDQFVVPHDLEEEVKEQMKQHQDSRLEPEPHEEDRTEPPEEFD
TAL
>hsa:231
MASRLLLNNGAKMPILGLGTWKSPPGQVTEAVKVAIDVGYRHIDCAHVYQNENEVGVAIQ
EKLREQVVKREELFIVSKLWCTYHEKGLVKGACQKTLSDLKLDYLDLYLIHWPTGFKPGK
EFFPLDESGNVVPSDTNILDTWAAMEELVDEGLVKAIGISNFNHLQVEMILNKPGLKYKP
AVNQIECHPYLTQEKLIQYCQSKGIVVTAYSPLGSPDRPWAKPEDPSLLEDPRIKAIAAK
HNKTTAQVLIRFPMQRNLVVIPKSVTPERIAENFKVFDFELSSQDMTTLLSYNRNWRVCA
LLSCTSHKDYPFHEEF
>hsa:2321
MVSYWDTGVLLCALLSCLLLTGSSSGSKLKDPELSLKGTQHIMQAGQTLHLQCRGEAAHK
WSLPEMVSKESERLSITKSACGRNGKQFCSTLTLNTAQANHTGFYSCKYLAVPTSKKKET
ESAIYIFISDTGRPFVEMYSEIPEIIHMTEGRELVIPCRVTSPNITVTLKKFPLDTLIPD
GKRIIWDSRKGFIISNATYKEIGLLTCEATVNGHLYKTNYLTHRQTNTIIDVQISTPRPV
KLLRGHTLVLNCTATTPLNTRVQMTWSYPDEKNKRASVRRRIDQSNSHANIFYSVLTIDK
MQNKDKGLYTCRVRSGPSFKSVNTSVHIYDKAFITVKHRKQQVLETVAGKRSYRLSMKVK
AFPSPEVVWLKDGLPATEKSARYLTRGYSLIIKDVTEEDAGNYTILLSIKQSNVFKNLTA
TLIVNVKPQIYEKAVSSFPDPALYPLGSRQILTCTAYGIPQPTIKWFWHPCNHNHSEARC
DFCSNNEESFILDADSNMGNRIESITQRMAIIEGKNKMASTLVVADSRISGIYICIASNK
VGTVGRNISFYITDVPNGFHVNLEKMPTEGEDLKLSCTVNKFLYRDVTWILLRTVNNRTM
HYSISKQKMAITKEHSITLNLTIMNVSLQDSGTYACRARNVYTGEEILQKKEITIRGEHC
NKKAVFSRISKFKSTRNDCTTQSNVKH
>hsa:2322
MPALARDGGQLPLLVVFSAMIFGTITNQDLPVIKCVLINHKNNDSSVGKSSSYPMVSESP
EDLGCALRPQSSGTVYEAAAVEVDVSASITLQVLVDAPGNISCLWVFKHSSLNCQPHFDL
QNRGVVSMVILKMTETQAGEYLLFIQSEATNYTILFTVSIRNTLLYTLRRPYFRKMENQD
ALVCISESVPEPIVEWVLCDSQGESCKEESPAVVKKEEKVLHELFGTDIRCCARNELGRE
CTRLFTIDLNQTPQTTLPQLFLKVGEPLWIRCKAVHVNHGFGLTWELENKALEEGNYFEM
STYSTNRTMIRILFAFVSSVARNDTGYYTCSSSKHPSQSALVTIVEKGFINATNSSEDYE

IDQYEEFCFSVRFKAYPQIRCTWTFSRKSFPCEQKGLDNGYSISKFCNHKHQPGEYIFHA
ENDDAQFTKMFTLNIRRKPQVLAEASASQASCFSDGYPLPSWTWKKCSDKSPNCTEEITE
GVWNRKANRKVFGQWVSSSTLNMSEAIKGFLVKCCAYNSLGTSCETILLNSPGPFPFIQD
NISFYATIGVCLLFIVVLTLLICHKYKKQFRYESQLQMVQVTGSSDNEYFYVDFREYEYD
LKWEFPRENLEFGKVLGSGAFGKVMNATAYGISKTGVSIQVAVKMLKEKADSSEREALMS
ELKMMTQLGSHENIVNLLGACTLSGPIYLIFEYCCYGDLLNYLRSKREKFHRTWTEIFKE
HNFSFYPTFQSHPNSSMPGSREVQIHPDSDQISGLHGNSFHSEDEIEYENQKRLEEEEDL
NVLTFEDLLCFAYQVAKGMEFLEFKSCVHRDLAARNVLVTHGKVVKICDFGLARDIMSDS
NYVVRGNARLPVKWMAPESLFEGIYTIKSDVWSYGILLWEIFSLGVNPYPGIPVDANFYK
LIQNGFKMDQPFYATEEIYIIMQSCWAFDSRKRPSFPNLTSFLGCQLADAEEAMYQNVDG
RVSECPHTYQNRRPFSREMDLGLLSPQAQVEDS

>hsa:23236

MAGAQPGVHALQLKPVCVSDSLKKGTKFVKWDDDSTIVTPIILRTDPQGFFFYWTDQNKE
TELLDLSLVKDARCGRHAKAPKDPKLRELLDVGNIGRLEQRMITVVYGPDLVNISHLNLV
AFQEEVAKEWTNEVFSLATNLLAQNMSRDAFLEKAYTKLKLQVTPEGRIPLKNIYRLFSA
DRKRVETALEACSLPSSRNDSIPQEDFTPEVYRVFLNNLCPRPEIDNIFSEFGAKSKPYL
TVDQMMDFINLKQRDPRLNEILYPPLKQEQVQVLIEKYEPNNSLARKGQISVDGFMRYLS
GEENGVVSPEKLDLNEDMSQPLSHYFINSSHNTYLTAGQLAGNSSVEMYRQVLLSGCRCV
ELDCWKGRTAEEEPVITHGFTMTTEISFKEVIEAIAECAFKTSPFPILLSFENHVDSPKQ
QAKMAEYCRLIFGDALLMEPLEKYPLESGVPLPSPMDLMYKILVKNKKKSHKSSEGSGKK
KLSEQASNTYSDSSSMFEPSSPGAGEADTESDDDDDDDDCKKSSMDEGTAGSEAMATEEM
SNLVNYIQPVKFESFEISKKRNKSFEMSSFVETKGLEQLTKSPVEFVEYNKMQLSRIYPK
GTRVDSSNYMPQLFWNAGCQMVALNFQTMDLAMQINMGMYEYNGKSGYRLKPEFMRRPDK
HFDPFTEGIVDGIVANTLSVKIISGQFLSDKKVGTYVEVDMFGLPVDTRRKAFKTKTSQG
NAVNPVWEEEPIVFKKVVLPTLACLRIAVYEEGGKFIGHRILPVQAIRPGYHYICLRNER
NQPLTLPAVFVYIEVKDYVPDTYADVIEALSNPIRYVNLMEQRAKQLAALTLEDEEEVKK
EADPGETPSEAPSEARTTPAENGVNHTTTLTPKPPSQALHSQPAPGSVKAPAKTEDLIQS
VLTEVEAQTIEELKQQKSFVKLQKKHYKEMKDLVKRHHKKTTDLIKEHTTKYNEIQNDYL
RRRAALEKSAKKDSKKKSEPSSPDHGSSTIEQDLAALDAEMTQKLIDLKDKQQQQLLNLR
QEQYYSEKYQKREHIKLLIQKLTDVAEECQNNQLKKLKEICEKEKKELKKKMDKKRQEKI
TEAKSKDKSQMEEEKTEMIRSYIQEVVQYIKRLEEAQSKRQEKLVEKHKEIRQQILDEKP
KLQVELEQEYQDKFKRLPLEILEFVQEAMKGKISEDSNHGSAPLSLSSDPGKVNHKTPSS
EELGGDIPGKEFDTPL

>hsa:23239

MEPAAAATVQRLPELGREDRASAPAAAAAAAAAAAAAAALAAAAGGGRSPEPALTPAAP
SGGNGSGSGAREEAPGEAPPGPLPGRAGGAGRRRRRGAPQPIAGGAAPVPGAGGGANSLL
LRRGRLKRNLSAAAAAASSSSSSSAAAASHSPGAAGLPASCSASASLCTRSLDRKTLLLK
HRQTLQLQPSDRDWVRHQLQRGCVHVFDRHMASTYLRPVLCTLDTTAGEVAARLLQLGHK
GGGVVKVLGQGPGAAAAREPAEPPPEAGPRLAPPEPRDSEVPPARSAPGAFGGPPRAPPA
DLPLPVGGPGGWSRRASPAPSDSSPGEPFVGGPVSSPRAPRPVVSDTESFSLSPSAESVS
DRLDPYSSGGGSSSSSEELEADAASAPTGVPGQPRRPGHPAQPLPLPQTASSPQPQQKAP
RAIDSPGGAVREGSCEEKAAAAVAPGGLQSTPGRSGVTAEKAPPPPPPPTLYVQLHGETT
RRLEAEEKPLQIQNDYLFQLGFGELWRVQEEGMDSEIGCLIRFYAGKPHSTGSSERIQLS
GMYNVRKGKMQLPVNRWTRRQVILCGTCLIVSSVKDSLTGKMHVLPLIGGKVEEVKKHQH

CLAFSSSGPQSQTYYICFDTFTEYLRWLRQVSKVASQRISSVDLSCCSLEHLPANLFYSQ
DLTHLNLKQNFLRQNPSLPAARGLNELQRFTKLKSLNLSNNHLGDFPLAVCSIPTLAELN
VSCNALRSVPAAVGVMHNLQTFLLDGNFLQSLPAELENMKQLSYLGLSFNEFTDIPEVLE
KLTAVDKLCMSGNCVETLRLQALRKMPHIKHVDLRLNVIRKLIADEVDFLQHVTQLDLRD
NKLGDLDAMIFNNIEVLHCERNQLVTLDICGYFLKALYASSNELVQLDVYPVPNYLSYMD
VSRNRLENVPEWVCESRKLEVLDIGHNQICELPARLFCNSSLRKLLAGHNQLARLPERLE
RTSVEVLDVQHNQLLELPPNLLMKADSLRFLNASANKLESLPPATLSEETNSILQELYLT
NNSLTDKCVPLLTGHPHLKILHMAYNRLQSFPASKMAKLEELEEIDLSGNKLKAIPTTIM
NCRRMHTVIAHSNCIEVFPEVMQLPEIKCVDLSCNELSEVTLPENLPPKLQELDLTGNPR
LVLDHKTLELLNNIRCFKIDQPSTGDASGAPAVWSHGYTEASGVKNKLCVAALSVNNFCD
NREALYGVFDGDRNVEVPYLLQCTMSDILAEELQKTKNEEEYMVNTFIVMQRKLGTAGQK
LGGAAVLCHIKHDPVDPGGSFTLTSANVGKCQTVLCRNGKPLPLSRSYIMSCEEELKRIK
QHKAIITEDGKVNGVTESTRILGYTFLHPSVVPRPHVQSVLLTPQDEFFILGSKGLWDSL
SVEEAVEAVRNVPDALAAAKKLCTLAQSYGCHDSISAVVVQLSVTEDSFCCCELSAGGAV
PPPSPGIFPPSVNMVIKDRPSDGLGVPSSSSGMASEISSELSTSEMSSEVGSTASDEPPP
GALSENSPAYPSEQRCMLHPICLSNSFQRQLSSATFSSAFSDNGLDSDDEEPIEGVFTNG
SRVEVEVDIHCSRAKEKEKQQHLLQVPAEASDEGIVISANEDEPGLPRKADFSAVGTIGR
RRANGSVAPQERSHNVIEVATDAPLRKPGGYFAAPAQPDPDDQFIIPPELEEEVKEIMKH
HQEQQQQQPPPPPQLQPQLPRHYQLDQLPDYYDTPL

>hsa:2324

MQRGAALCLRLWLCLGLLDGLVSGYSMTPPTLNITEESHVIDTGDSLSISCRGQHPLEWA
WPGAQEAPATGDKDSEDTGVVRDCEGTDARPYCKVLLLHEVHANDTGSYVCYYKYIKARI
EGTTAASSYVFVRDFEQPFINKPDTLLVNRKDAMWVPCLVSIPGLNVTLRSQSSVLWPDG
QEVVWDDRRGMLVSTPLLHDALYLQCETTWGDQDFLSNPFLVHITGNELYDIQLLPRKSL
ELLVGEKLVLNCTVWAEFNSGVTFDWDYPGKQAERGKWVPERRSQQTHTELSSILTIHNV
SQHDLGSYVCKANNGIQRFRESTEVIVHENPFISVEWLKGPILEATAGDELVKLPVKLAA
YPPPEFQWYKDGKALSGRHSPHALVLKEVTEASTGTYTLALWNSAAGLRRNISLELVVNV
PPQIHEKEASSPSIYSRHSRQALTCTAYGVPLPLSIQWHWRPWTPCKMFAQRSLRRRQQQ
DLMPQCRDWRAVTTQDAVNPIESLDTWTEFVEGKNKTVSKLVIQNANVSAMYKCVVSNKV
GQDERLIYFYVTTIPDGFTIESKPSEELLEGQPVLLSCQADSYKYEHLRWYRLNLSTLHD
AHGNPLLLDCKNVHLFATPLAASLEEVAPGARHATLSLSIPRVAPEHEGHYVCEVQDRRS
HDKHCHKKYLSVQALEAPRLTQNLTDLLVNVSDSLEMQCLVAGAHAPSIVWYKDERLLEE
KSGVDLADSNQKLSIQRVREEDAGRYLCSVCNAKGCVNSSASVAVEGSEDKGSMEIVILV
GTGVIAVFFWVLLLLIFCNMRRPAHADIKTGYLSIIMDPGEVPLEEQCEYLSYDASQWEF
PRERLHLGRVLGYGAFGKVVEASAFGIHKGSSCDTVAVKMLKEGATASEHRALMSELKIL
IHIGNHLNVVNLLGACTKPQGPLMVIVEFCKYGNLSNFLRAKRDAFSPCAEKSPEQRGRF
RAMVELARLDRRRPGSSDRVLFARFSKTEGGARRASPDQEAEDLWLSPLTMEDLVCYSFQ
VARGMEFLASRKCIHRDLAARNILLSESDVVKICDFGLARDIYKDPDYVRKGSARLPLKW
MAPESIFDKVYTTQSDVWSFGVLLWEIFSLGASPYPGVQINEEFCQRLRDGTRMRAPELA
TPAIRRIMLNCWSGDPKARPAFSELVEILGDLLQGRGLQEEEEVCMAPRSSQSSEEGSFS
QVSTMALHIAQADAEDSPPSLQRHSLAARYYNWVSFPGCLARGAETRGSSRMKTFEEFPM
TPTTYKGSVDNQTDSGMVLASEEFEQIESRHRQESGFR

>hsa:2326

MAKRVAIVGAGVSGLASIKCCLEEGLEPTCFERSDDLGGLWRFTEHVEEGRASLYKSVVS

NSCKEMSCYSDFPFPEDYPNYVPNSQFLEYLKMYANHFDLLKHIQFKTKVCSVTKCSDSA
VSGQWEVVTMHEEKQESAIFDAVMVCTGFLTNPYLPLDSFPGINAFKGQYFHSRQYKHPD
IFKDKRVLVIGMGNSGTDIAVEASHLAEKVFLSTTGGGWVISRIFDSGYPWDMVFMTRFQ
NMLRNSLPTPIVTWLMERKINNWLNHANYGLIPEDRTQLKEFVLNDELPGRIITGKVFIR
PSIKEVKENSVIFNNTSKEEPIDIIVFATGYTFAFPFLDESVVKVEDGQASLYKYIFPAH
LQKPTLAIIGLIKPLGSMIPTGETQARWAVRVLKGVNKLPPPSVMIEEINARKENKPSWF
GLCYCKALQSDYITYIDELLTYINAKPNLFSMLLTDPHLALTVFFGPCSPYQFRLTGPGK
WEGARNAIMTQWDRTFKVIKARVVQESPSPFESFLKVFSFLALLVAIFLIFL
>hsa:2328
MGKKVAIIGAGVSGLASIRSCLEEGLEPTCFEKSNDIGGLWKFSDHAEEGRASIYKSVFS
NSSKEMMCFPDFPFPDDFPNFMHNSKIQEYIIAFAKEKNLLKYIQFKTFVSSVNKHPDFA
TTGQWDVTTERDGKKESAVFDAVMVCSGHHVYPNLPKESFPGLNHFKGKCFHSRDYKEPG
VFNGKRVLVVGLGNSGCDIATELSRTAEQVMISSRSGSWVMSRVWDNGYPWDMLLVTRFG
TFLKNNLPTAISDWLYVKQMNARFKHENYGLMPLNGVLRKEPVFNDELPASILCGIVSVK
PNVKEFTETSAIFEDGTIFEGIDCVIFATGYSFAYPFLDESIIKSRNNEIILFKGVFPPL
LEKSTIAVIGFVQSLGAAIPTVDLQSRWAAQVIKGTCTLPSMEDMMNDINEKMEKKRKWF
GKSETIQTDYIVYMDELSSFIGAKPNIPWLFLTDPKLAMEVYFGPCSPYQFRLVGPGQWP
GARNAILTQWDRSLKPMQTRVVGRLQKPCFFFHWLKLFAIPILLIAVFLVLT
>hsa:2339
MAATEGVGEAAQGGEPGQPAQPPPQPHPPPPQQQHKEEMAAEAGEAVASPMDDGFVSLDS
PSYVLYRDRAEWADIDPVPQNDGPNPVVQIIYSDKFRDVYDYFRAVLQRDERSERAFKLT
RDAIELNAANYTVWHFRRVLLKSLQKDLHEEMNYITAIIEEQPKNYQVWHHRRVLVEWLR
DPSQELEFIADILNQDAKNYHAWQHRQWVIQEFKLWDNELQYVDQLLKEDVRNNSVWNQR
YFVISNTTGYNDRAVLEREVQYTLEMIKLVPHNESAWNYLKGILQDRGLSKYPNLLNQLL
DLQPSHSSPYLIAFLVDIYEDMLENQCDNKEDILNKALELCEILAKEKDTIRKEYWRYIG
RSLQSKHSTENDSPTNVQQ
>hsa:2342
MASPSSFTYYCPPSSSPVWSEPLYSLRPEHARERLQDDSVETVTSIEQAKVEEKIQEVFS
SYKFNHLVPRLVLQREKHFHYLKRGLRQLTDAYECLDASRPWLCYWILHSLELLDEPIPQ
IVATDVCQFLELCQSPEGGFGGGPGQYPHLAPTYAAVNALCIIGTEEAYDIINREKLLQY
LYSLKQPDGSFLMHVGGEVDVRSAYCAASVASLTNIITPDLFEGTAEWIARCQNWEGGIG
GVPGMEAHGGYTFCGLAALVILKRERSLNLKSLLQWVTSRQMRFEGGFQGRCNKLVDGCY
SFWQAGLLPLLHRALHAQGDPALSMSHWMFHQQALQEYILMCCQCPAGGLLDKPGKSRDF
YHTCYCLSGLSIAQHFGSGAMLHDVVLGVPENALQPTHPVYNIGPDKVIQATTYFLQKPV
PGFEELKDETSAEPATD
>hsa:23430
MLLLAPQMLSLLLLALPVLASPAYVAPAPGQALQQTGIVGGQEAPRSKWPWQVSLRVRGP
YWMHFCGGSLIHPQWVLTAAHCVEPDIKDLAALRVQLREQHLYYQDQLLPVSRIIVHPQF
YIIQTGADIALLELEEPVNISSHIHTVTLPPASETFPPGMPCWVTGWGDVDNNVHLPPPY
PLKEVEVPVVENHLCNAEYHTGLHTGHSFQIVRDDMLCAGSENHDSCQGDSGGPLVCKVN
GT
>hsa:23436
MMLRLLSSLLLVAVASGYGPPSSRPSSRVVNGEDAVPYSWPWQVSLQYEKSGSFYHTCGG
SLIAPDWVVTAGHCISSSRTYQVVLGEYDRAVKEGPEQVIPINSGDLFVHPLWNRSCVAC

GNDIALIKLSRSAQLGDAVQLASLPPAGDILPNETPCYITGWGRLYTNGPLPDKLQEALL
PVVDYEHCSRWNWWGSSVKKTMVCAGGDIRSGCNGDSGGPLNCPTEDGGWQVHGVTSFVS
AFGCNTRRKPTVFTRVSAFIDWIEETIASH
>hsa:23475
MDAEGLALLLPPVTLAALVDSWLREDCPGLNYAALVSGAGPSQAALWAKSPGVLAGQPFF
DAIFTQLNCQVSWFLPEGSKLVPVARVAEVRGPAHCLLLGERVALNTLARCSGIASAAAA
AVEAARGAGWTGHVAGTRKTTPGFRLVEKYGLLVGGAASHRYDLGGLVMVKDNHVVAAGG
VEKAVRAARQAADFALKVEVECSSLQEAVQAAEAGADLVLLDNFKPEELHPTATVLKAQF
PSVAVEASGGITLDNLPQFCGPHIDVISMGMLTQAAPALDFSLKLFAKEVAPVPKIH
>hsa:2356
MLNTLQTNAGYLEQVKRQRGDPQTQLEAMELYLARSGLQVEDLDRLNIIHVTGTKGKGST
CAFTECILRSYGLKTGFFSSPHLVQVRERIRINGQPISPELFTKYFWRLYHRLEETKDGS
CVSMPPYFRFLTLMAFHVFLQEKVDLAVVEVGIGGAYDCTNIIRKPVVCGVSSLGIDHTS
LLGDTVEKIAWQKGGIFKQGVPAFTVLQPEGPLAVLRDRAQQISCPLYLCPMLEALEEGG
PPLTLGLEGEHQRSNAALALQLAHCWLQRQDRHGAGEPKASRPGLLWQLPLAPVFQPTSH
MRLGLRNTEWPGRTQVLRRGPLTWYLDGAHTASSAQACVRWFRQALQGRERPSGGPEVRV
LLFNATGDRDPAALLKLLQPCQFDYAVFCPNLTEVSSTGNADQQNFTVTLDQVLLRCLEH
QQHWNHLDEEQASPDLWSAPSPEPGGSASLLLAPHPPHTCSASSLVFSCISHALQWISQG
RDPIFQPPSPPKGLLTHPVAHSGASILREAAAIHVLVTGSLHLVGGVLKLLEPALSQ
>hsa:23632
MLFSALLLEVIWILAADGGQHWTYEGPHGQDHWPASYPECGNNAQSPIDIQTDSVTFDPD
LPALQPHGYDQPGTEPLDLHNNGHTVQLSLPSTLYLGGLPRKYVAAQLHLHWGQKGSPGG
SEHQINSEATFAELHIVHYDSDSYDSLSEAAERPQGLAVLGILIEVGETKNIAYEHILSH
LHEVRHKDQKTSVPPFNLRELLPKQLGQYFRYNGSLTTPPCYQSVLWTVFYRRSQISMEQ
LEKLQGTLFSTEEEPSKLLVQNYRALQPLNQRMVFASFIQAGSSYTTGEMLSLGVGILVG
CLCLLLAVYFIARKIRKKRLENRKSVVFTSAQATTEA
>hsa:238
MGAIGLLWLLPLLLSTAAVGSGMGTGQRAGSPAAGPPLQPREPLSYSRLQRKSLAVDFVV
PSLFRVYARDLLLPPSSSELKAGRPEARGSLALDCAPLLRLLGPAPGVSWTAGSPAPAEA
RTLSRVLKGGSVRKLRRAKQLVLELGEEAILEGCVGPPGEAAVGLLQFNLSELFSWWIRQ
GEGRLRIRLMPEKKASEVGREGRLSAAIRASQPRLLFQIFGTGHSSLESPTNMPSPSPDY
FTWNLTWIMKDSFPFLSHRSRYGLECSFDFPCELEYSPPLHDLRNQSWSWRRIPSEEASQ
MDLLDGPGAERSKEMPRGSFLLLNTSADSKHTILSPWMRSSSEHCTLAVSVHRHLQPSGR
YIAQLLPHNEAAREILLMPTPGKHGWTVLQGRIGRPDNPFRVALEYISSGNRSLSAVDFF
ALKNCSEGTSPGSKMALQSSFTCWNGTVLQLGQACDFHQDCAQGEDESQMCRKLPVGFYC
NFEDGFCGWTQGTLSPHTPQWQVRTLKDARFQDHQDHALLLSTTDVPASESATVTSATFP
APIKSSPCELRMSWLIRGVLRGNVSLVLVENKTGKEQGRMVWHVAAYEGLSLWQWMVLPL
LDVSDRFWLQMVAWWGQGSRAIVAFDNISISLDCYLTISGEDKILQNTAPKSRNLFERNP
NKELKPGENSPRQTPIFDPTVHWLFTTCGASGPHGPTQAQCNNAYQNSNLSVEVGSEGPL
KGIQIWKVPATDTYSISGYGAAGGKGGKNTMMRSHGVSVLGIFNLEKDDMLYILVGQQGE
DACPSTNQLIQKVCIGENNVIEEEIRVNRSVHEWAGGGGGGGGATYVFKMKDGVPVPLII
AAGGGGRAYGAKTDTFHPERLENNSSVLGLNGNSGAAGGGGGWNDNTSLLWAGKSLQEGA
TGGHSCPQAMKKWGWETRGGFGGGGGGCSSGGGGGGYIGGNAASNNDPEMDGEDGVSFIS
PLGILYTPALKVMEGHGEVNIKHYLNCSHCEVDECHMDPESHKVICFCDHGTVLAEDGVS

CIVSPTPEPHLPLSLILSVVTSALVAALVLAFSGIMIVYRRKHQELQAMQMELQSPEYKL
SKLRTSTIMTDYNPNYCFAGKTSSISDLKEVPRKNITLIRGLGHGAFGEVYEGQVSGMPN
DPSPLQVAVKTLPEVCSEQDELDFLMEALIISKFNHQNIVRCIGVSLQSLPRFILLELMA
GGDLKSFLRETRPRPSQPSSLAMLDLLHVARDIACGCQYLEENHFIHRDIAARNCLLTCP
GPGRVAKIGDFGMARDIYRASYYRKGGCAMLPVKWMPPEAFMEGIFTSKTDTWSFGVLLW
EIFSLGYMPYPSKSNQEVLEFVTSGGRMDPPKNCPGPVYRIMTQCWQHQPEDRPNFAIIL
ERIEYCTQDPDVINTALPIEYGPLVEEEEKVPVRPKDPEGVPPLLVSQQAKREEERSPAA
PPPLPTTSSGKAAKKPTAAEISVRVPRGPAVEGGHVNMAFSQSNPPSELHKVHGSRNKPT
SLWNPTYGSWFTEKPTKKNNPIAKKEPHDRGNLGLEGSCTVPPNVATGRLPGASLLLEPS
SLTANMKEVPLFRLRHFPCGNVNYGYQQQGLPLEAATAPGAGHYEDTILKSKNSMNQPGP
>hsa:239
MGRYRIRVATGAWLFSGSYNRVQLWLVGTRGEAELELQLRPARGEEEEFDHDVAEDLGLL
QFVRLRKHHWLVDDAWFCDRITVQGPGACAEVAFPCYRWVQGEDILSLPEGTARLPGDNA
LDMFQKHREKELKDRQQIYCWATWKEGLPLTIAADRKDDLPPNMRFHEEKRLDFEWTLKA
GALEMALKRVYTLLSSWNCLEDFDQIFWGQKSALAEKVRQCWQDDELFSYQFLNGANPML
LRRSTSLPSRLVLPSGMEELQAQLEKELQNGSLFEADFILLDGIPANVIRGEKQYLAAPL
VMLKMEPNGKLQPMVIQIQPPNPSSPTPTLFLPSDPPLAWLLAKSWVRNSDFQLHEIQYH
LLNTHLVAEVIAVATMRCLPGLHPIFKFLIPHIRYTMEINTRARTQLISDGGIFDKAVST
GGGGHVQLLRRAAAQLTYCSLCPPDDLADRGLLGLPGALYAHDALRLWEIIARYVEGIVH
LFYQRDDIVKGDPELQAWCREITEVGLCQAQDRGFPVSFQSQSQLCHFLTMCVFTCTAQH
AAINQGQLDWYAWVPNAPCTMRMPPPTTKEDVTMATVMGSLPDVRQACLQMAISWHLSRR
QPDMVPLGHHKEKYFSGPKPKAVLNQFRTDLEKLEKEITARNEQLDWPYEYLKPSCIENS
VTI
>hsa:240
MPSYTVTVATGSQWFAGTDDYIYLSLVGSAGCSEKHLLDKPFYNDFERGAVDSYDVTVDE
ELGEIQLVRIEKRKYWLNDDWYLKYITLKTPHGDYIEFPCYRWITGDVEVVLRDGRAKLA
RDDQIHILKQHRRKELETRQKQYRWMEWNPGFPLSIDAKCHKDLPRDIQFDSEKGVDFVL
NYSKAMENLFINRFMHMFQSSWNDFADFEKIFVKISNTISERVMNHWQEDLMFGYQFLNG
CNPVLIRRCTELPEKLPVTTEMVECSLERQLSLEQEVQQGNIFIVDFELLDGIDANKTDP
CTLQFLAAPICLLYKNLANKIVPIAIQLNQIPGDENPIFLPSDAKYDWLLAKIWVRSSDF
HVHQTITHLLRTHLVSEVFGIAMYRQLPAVHPIFKLLVAHVRFTIAINTKAREQLICECG
LFDKANATGGGGHVQMVQRAMKDLTYASLCFPEAIKARGMESKEDIPYYFYRDDGLLVWE
AIRTFTAEVVDIYYEGDQVVEEDPELQDFVNDVYVYGMRGRKSSGFPKSVKSREQLSEYL
TVVIFTASAQHAAVNFGQYDWCSWIPNAPPTMRAPPPTAKGVVTIEQIVDTLPDRGRSCW
HLGAVWALSQFQENELFLGMYPEEHFIEKPVKEAMARFRKNLEAIVSVIAERNKKKQLPY
YYLSPDRIPNSVAI
>hsa:242
MATYKVRVATGTDLLSGTRDSISLTIVGTQGESHKQLLNHFGRDFATGAVGQYTVQCPQD
LGELIIIRLHKERYAFFPKDPWYCNYVQICAPNGRIYHFPAYQWMDGYETLALREATGKT
TADDSLPVLLEHRKEEIRAKQDFYHWRVFLPGLPSYVHIPSYRPPVRRHRNPNRPEWNGY
IPGFPILINFKATKFLNLNLRYSFLKTASFFVRLGPMALAFKVRGLLDCKHSWKRLKDIR
KIFPGKKSVVSEYVAEHWAEDTFFGYQYLNGVNPGLIRRCTRIPDKFPVTDDMVAPFLGE
GTCLQAELEKGNIYLADYRIMEGIPTVELSGRKQHHCAPLCLLHFGPEGKMMPIAIQLSQ
TPGPDCPIFLPSDSEWDWLLAKTWVRYAEFYSHEAIAHLLETHLIAEAFCLALLRNLPMC

HPLYKLLIPHTRYTVQINSIGRAVLLNEGGLSAKGMSLGVEGFAGVMVRALSELTYDSLY
LPNDFVERGVQDLPGYYYRDDSLAVWNALEKYVTEIITYYYPSDAAVEGDPELQSWVQEI
FKECLLGRESSGFPRCLRTVPELIRYVTIVIYTCSAKHAAVNTGQMEFTAWMPNFPASMR
NPPIQTKGLTTLETFMDTLPDVKTTCITLLVLWTLSREPDDRRPLGHFPDIHFVEEAPRR
SIEAFRQRLNQISHDIRQRNKCLPIPYYYLDPVLIENSISI

>hsa:246

MGLYRIRVSTGASLYAGSNNQVQLWLVGQHGEAALGKRLWPARGKETELKVEVPEYLGPL
LFVKLRKRHLLKDDAWFCNWISVQGPGAGDEVRFPCYRWVEGNGVLSLPEGTGRTVGEDP
QGLFQKHREEELEERRKLYRWGNWKDGLILNMAGAKLYDLPVDERFLEDKRVDFEVSLAK
GLADLAIKDSLNVLTCWKDLDDFNRIFWCGQSKLAERVRDSWKEDALFGYQFLNGANPVV
LRRSAHLPARLVFPPGMEELQAQLEKELEGGTLFEADFSLLDGIKANVILCSQQHLAAPL
VMLKLQPDGKLLPMVIQLQLPRTGSPPPPLFLPTDPPMAWLLAKCWVRSSDFQLHELQSH
LLRGHLMAEVIVVATMRCLPSIHPIFKLIIPHLRYTLEINVRARTGLVSDMGIFDQIMST
GGGGHVQLLKQAGAFLTYSSFCPPDDLADRGLLGVKSSFYAQDALRLWEIIYRYVEGIVS
LHYKTDVAVKDDPELQTWCREITEIGLQGAQDRGFPVSLQARDQVCHFVTMCIFTCTGQH
ASVHLGQLDWYSWVPNAPCTMRLPPPTTKDATLETVMATLPNFHQASLQMSITWQLGRRQ
PVMVAVGQHEEEYFSGPEPKAVLKKFREELAALDKEIEIRNAKLDMPYEYLRPSVVENSV
AI

>hsa:247

MAEFRVRVSTGEAFGAGTWDKVSVSIVGTRGESPPLPLDNLGKEFTAGAEEDFQVTLPED
VGRVLLLRVHKAPPVLPLLGPLAPDAWFCRWFQLTPPRGGHLLFPCYQWLEGAGTLVLQE
GTAKVSWADHHPVLQQQRQEELQARQEMYQWKAYNPGWPHCLDEKTVEDLELNIKYSTAK
NANFYLQAGSAFAEMKIKGLLDRKGLWRSLNEMKRIFNFRRTPAAEHAFEHWQEDAFFAS
QFLNGLNPVLIRRCHYLPKNFPVTDAMVASVLGPGTSLQAELEKGSLFLVDHGILSGIQT
NVINGKPQFSAAPMTLLYQSPGCGPLLPLAIQLSQTPGPNSPIFLPTDDKWDWLLAKTWV
RNAEFSFHEALTHLLHSHLLPEVFTLATLRQLPHCHPLFKSTGIGIEGFSELIQRNMKQL
NYSLLCLPEDIRTRGVEDIPGYYYRDDGMQIWGAVERFVSEIIGIYYPSDESVQDDRELQ
AWVREIFSKGFLNQESSGIPSSLETREALVQYVTMVIFTCSAKHAAVSAGQFDSCAWMPN
LPPSMQLPPPTSKGLATCEGFIATLPPVNATCDVILALWLLSKEPGDQRPLGTYPDEHFT
EEAPRRSIATFQSRLAQISRGIQERNQGLVLPYTYLDPPLIENSVSI

>hsa:25

MLEICLKLVGCKSKKGLSSSSSCYLEEALQRPVASDFEPQGLSEAARWNSKENLLAGPSE
NDPNLFVALYDFVASGDNTLSITKGEKLRVLGYNHNGEWCEAQTKNGQGWVPSNYITPVN
SLEKHSWYHGPVSRNAAEYLLSSGINGSFLVRESESSPGQRSISLRYEGRVYHYRINTAS
DGKLYVSSESRFNTLAELVHHHSTVADGLITTLHYPAPKRNKPTVYGVSPNYDKWEMERT
DITMKHKLGGGQYGEVYEGVWKKYSLTVAVKTLKEDTMEVEEFLKEAAVMKEIKHPNLVQ
LLGVCTREPPFYIITEFMTYGNLLDYLRECNRQEVNAVVLLYMATQISSAMEYLEKKNFI
HRDLAARNCLVGENHLVKVADFGLSRLMTGDTYTAHAGAKFPIKWTAPESLAYNKFSIKS
DVWAFGVLLWEIATYGMSPYPGIDLSQVYELLEKDYRMERPEGCPEKVYELMRACWQWNP
SDRPSFAEIHQAFETMFQESSISDEVEKELGKQGVRGAVSTLLQAPELPTKTRTSRRAAE
HRDTTDVPEMPHSKGQGESDPLDHEPAVSPLLPRKERGPPEGGLNEDERLLPKDKKTNLF
SALIKKKKKTAPTPPKRSSSFREMDGQPERRGAGEEEGRDISNGALAFTPLDTADPAKSP
KPSNGAGVPNGALRESGGSGFRSPHLWKKSSTLTSSRLATGEEEGGGSSSKRFLRSCSAS
CVPHGAKDTEWRSVTLPRDLQSTGRQFDSSTFGGHKSEKPALPRKRAGENRSDQVTRGTV

TPPPRLVKKNEEAADEVFKDIMESSPGSSPPNLTPKPLRRQVTVAPASGLPHKEEAGKGS
ALGTPAAAEPVTPTSKAGSGAPGGTSKGPAEESRVRRHKHSSESPGRDKGKLSRLKPAPP
PPPAASAGKAGGKPSQSPSQEAAGEAVLGAKTKATSLVDAVNSDAAKPSQPGEGLKKPVL
PATPKPQSAKPSGTPISPAPVPSTLPSASSALAGDQPSSTAFIPLISTRVSLRKTRQPPE
RIASGAITKGVVLDSTEALCLAISRNSEQMASHSAVLEAGKNLYTFCVSYVDSIQQMRNK
FAFREAINKLENNLRELQICPATAGSGPAATQDFSKLLSSVKEISDIVQR
>hsa:2534
MGCVQCKDKEATKLTEERDGSLNQSSGYRYGTDPTPQHYPSFGVTSIPNYNNFHAAGGQG
LTVFGGVNSSSHTGTLRTRGGTGVTLFVALYDYEARTEDDLSFHKGEKFQILNSSEGDWW
EARSLTTGETGYIPSNYVAPVDSIQAEEWYFGKLGRKDAERQLLSFGNPRGTFLIRESET
TKGAYSLSIRDWDDMKGDHVKHYKIRKLDNGGYYITTRAQFETLQQLVQHYSERAAGLCC
RLVVPCHKGMPRLTDLSVKTKDVWEIPRESLQLIKRLGNGQFGEVWMGTWNGNTKVAIKT
LKPGTMSPESFLEEAQIMKKLKHDKLVQLYAVVSEEPIYIVTEYMNKGSLLDFLKDGEGR
ALKLPNLVDMAAQVAAGMAYIERMNYIHRDLRSANILVGNGLICKIADFGLARLIEDNEY
TARQGAKFPIKWTAPEAALYGRFTIKSDVWSFGILLTELVTKGRVPYPGMNNREVLEQVE
RGYRMPCPQDCPISLHELMIHCWKKDPEERPTFEYLQSFLEDYFTATEPQYQPGENL
>hsa:2548
MGVRHPPCSHRLLAVCALVSLATAALLGHILLHDFLLVPRELSGSSPVLEETHPAHQQGA
SRPGPRDAQAHPGRPRAVPTQCDVPPNSRFDCAPDKAITQEQCEARGCCYIPAKQGLQGA
QMGQPWCFFPPSYPSYKLENLSSSEMGYTATLTRTTPTFFPKDILTLRLDVMMETENRLH
FTIKDPANRRYEVPLETPHVHSRAPSPLYSVEFSEEPFGVIVRRQLDGRVLLNTTVAPLF
FADQFLQLSTSLPSQYITGLAEHLSPLMLSTSWTRITLWNRDLAPTPGANLYGSHPFYLA
LEDGGSAHGVFLLNSNAMDVVLQPSPALSWRSTGGILDVYIFLGPEPKSVVQQYLDVVGY
PFMPPYWGLGFHLCRWGYSSTAITRQVVENMTRAHFPLDVQWNDLDYMDSRRDFTFNKDG
FRDFPAMVQELHQGGRRYMMIVDPAISSSGPAGSYRPYDEGLRRGVFITNETGQPLIGKV
WPGSTAFPDFTNPTALAWWEDMVAEFHDQVPFDGMWIDMNEPSNFIRGSEDGCPNNELEN
PPYVPGVVGGTLQAATICASSHQFLSTHYNLHNLYGLTEAIASHRALVKARGTRPFVISR
STFAGHGRYAGHWTGDVWSSWEQLASSVPEILQFNLLGVPLVGADVCGFLGNTSEELCVR
WTQLGAFYPFMRNHNSLLSLPQEPYSFSEPAQQAMRKALTLRYALLPHLYTLFHQAHVAG
ETVARPLFLEFPKDSSTWTVDHQLLWGEALLITPVLQAGKAEVTGYFPLGTWYDLQTVPV
EALGSLPPPPAAPREPAIHSEGQWVTLPAPLDTINVHLRAGYIIPLQGPGLTTTESRQQP
MALAVALTKGGEARGELFWDDGESLEVLERGAYTQVIFLARNNTIVNELVRVTSEGAGLQ
LQKVTVLGVATAPQQVLSNGVPVSNFTYSPDTKVLDICVSLLMGEQFLVSWC
>hsa:25796
MAAPAPGLISVFSSSQELGAALAQLVAQRAACCLAGARARFALGLSGGSLVSMLARELPA
AVAPAGPASLARWTLGFCDERLVPFDHAESTYGLYRTHLLSRLPIPESQVITINPELPVE
EAAEDYAKKLRQAFQGDSIPVFDLLILGVGPDGHTCSLFPDHPLLQEREKIVAPISDSPK
PPPQRVTLTLPVLNAARTVIFVATGEGKAAVLKRILEDQEENPLPAALVQPHTGKLCWFL
DEAAARLLTVPFEKHSTL
>hsa:2582
MAEKVLVTGGAGYIGSHTVLELLEAGYLPVVIDNFHNAFRGGGSLPESLRRVQELTGRSV
EFEEMDILDQGALQRLFKKYSFMAVIHFAGLKAVGESVQKPLDYYRVNLTGTIQLLEIMK
AHGVKNLVFSSSATVYGNPQYLPLDEAHPTGGCTNPYGKSKFFIEEMIRDLCQADKTWNA
VLLRYFNPTGAHASGCIGEDPQGIPNNLMPYVSQVAIGRREALNVFGNDYDTEDGTGVRD

YIHVVDLAKGHIAALRKLKEQCGCRIYNLGTGTGYSVLQMVQAMEKASGKKIPYKVVARR
EGDVAACYANPSLAQEELGWTAALGLDRMCEDLWRWQKQNPSGFGTQA
>hsa:25824
MGLAGVCALRRSAGYILVGGAGGQSAAAAARRCSEGEWASGGVRSFSRAAAAMAPIKVGD
AIPAVEVFEGEPGNKVNLAELFKGKKGVLFGVPGAFTPGCSKTHLPGFVEQAEALKAKGV
QVVACLSVNDAFVTGEWGRAHKAEGKVRLLADPTGAFGKETDLLLDDSLVSIFGNRRLKR
FSMVVQDGIVKALNVEPDGTGLTCSLAPNIISQL
>hsa:2595
MEAAVKEEISLEDEAVDKNIFRDCNKIAFYRRQKQWLSKKSTYQALLDSVTTDEDSTRFQ
IINEASKVPLLAEIYGIEGNIFRLKINEETPLKPRFEVPDVLTSKPSTVRLISCSGDTGS
LILADGKGDLKCHITANPFKVDLVSEEEVVISINSLGQLYFEHLQILHKQRAAKENEEET
SVDTSQENQEDLGLWEEKFGKFVDIKANGPSSIGLDFSLHGFEHLYGIPQHAESHQLKNT
GDGDAYRLYNLDVYGYQIYDKMGIYGSVPYLLAHKLGRTIGIFWLNASETLVEINTEPAV
EYTLTQMGPVAAKQKVRSRTHVHWMSESGIIDVFLLTGPTPSDVFKQYSHLTGTQAMPPL
FSLGYHQCRWNYEDEQDVKAVDAGFDEHDIPYDAMWLDIEHTEGKRYFTWDKNRFPNPKR
MQELLRSKKRKLVVISDPHIKIDPDYSVYVKAKDQGFFVKNQEGEDFEGVCWPGLSSYLD
FTNPKVREWYSSLFAFPVYQGSTDILFLWNDMNEPSVFRGPEQTMQKNAIHHGNWEHREL
HNIYGFYHQMATAEGLIKRSKGKERPFVLTRSFFAGSQKYGAVWTGDNTAEWSNLKISIP
MLLTLSITGISFCGADIGGFIGNPETELLVRWYQAGAYQPFFRGHATMNTKRREPWLFGE
EHTRLIREAIRERYGLLPYWYSLFYHAHVASQPVMRPLWVEFPDELKTFDMEDEYMLGSA
LLVHPVTEPKATTVDVFLPGSNEVWYDYKTFAHWEGGCTVKIPVALDTIPVFQRGGSVIP
IKTTVGKSTGWMTESSYGLRVALSTKGSSVGELYLDDGHSFQYLHQKQFLHRKFSFCSSV
LINSFADQRGHYPSKCVVEKILVLGFRKEPSSVTTHSSDGKDQPVAFTYCAKTSILSLEK
LSLNIATDWEVRII
>hsa:2597
MVYMFQYDSTHGKFHGTVKAENGKLVINGNPITIFQERDPSKIKWGDAGAEYVVESTGVF
TTMEKAGAHLQGGAKRVIISAPSADAPMFVMGVNHEKYDNSLKIISNASCTTNCLAPLAK
VIHDNFGIVEGLMTTVHAITATQKTVDGPSGKLWRDGRGALQNIIPASTGAAKAVGKVIP
ELNGKLTGMAFRVPTANVSVVDLTCRLEKPAKYDDIKKVVKQASEGPLKGILGYTEHQVV
SSDFNSDTHSSTFDAGAGIALNDHFVKLISWYDNEFGYSNRVVDLMAHMASKE
>hsa:260293
MEFSWLETRWARPFYLAFVFCLALGLLQAIKLYLRRQRLLRDLRPFPAPPTHWFLGHQKF
IQDDNMEKLEEIIEKYPRAFPFWIGPFQAFFCIYDPDYAKTLLSRTDPKSQYLQKFSPPL
LGKGLAALDGPKWFQHRRLLTPGFHFNILKAYIEVMAHSVKMMLDKWEKICSTQDTSVEV
YEHINSMSLDIIMKCAFSKETNCQTNSTHDPYAKAIFELSKIIFHRLYSLLYHSDIIFKL
SPQGYRFQKLSRVLNQYTDTIIQERKKSLQAGVKQDNTPKRKYQDFLDIVLSAKDESGSS
FSDIDVHSEVSTFLLAGHDTLAASISWILYCLALNPEHQERCREEVRGILGDGSSITWDQ
LGEMSYTTMCIKETCRLIPAVPSISRDLSKPLTFPDGCTLPAGITVVLSIWGLHHNPAVW
KNPKVFDPLRFSQENSDQRHPYAYLPFSAGSRNCIGQEFAMIELKVTIALILLHFRVTPD
PTRPLTFPNHFILKPKNGMYLHLKKLSEC
>hsa:26279
MELALLCGLVVMAGVIPIQGGILNLNKMVKQVTGKMPILSYWPYGCHCGLGGRGQPKDAT
DWCCQTHDCCYDHLKTQGCSIYKDYYRYNFSQGNIHCSDKGSWCEQQLCACDKEVAFCLK
RNLDTYQKRLRFYWRPHCRGQTPGC

>hsa:2638
MKRVLVLLLAVAFGHALERGRDYEKNKVCKEFSHLGKEDFTSLSLVLYSRKFPSGTFEQV
SQLVKEVVSLTEACCAEGADPDCYDTRTSALSAKSCESNSPFPVHPGTAECCTKEGLERK
LCMAALKHQPQEFPTYVEPTNDEICEAFRKDPKEYANQFMWEYSTNYGQAPLSLLVSYTK
SYLSMVGSCCTSASPTVCFLKERLQLKHLSLLTTLSNRVCSQYAAYGEKKSRLSNLIKLA
QKVPTADLEDVLPLAEDITNILSKCCESASEDCMAKELPEHTVKLCDNLSTKNSKFEDCC
QEKTAMDVFVCTYFMPAAQLPELPDVELPTNKDVCDPGNTKVMDKYTFELSRRTHLPEVF
LSKVLEPTLKSLGECCDVEDSTTCFNAKGPLLKKELSSFIDKGQELCADYSENTFTEYKK
KLAERLKAKLPDATPTELAKLVNKHSDFASNCCSINSPPLYCDSEIDAELKNIL
>hsa:2639
MALRGVSVRLLSRGPGLHVLRTWVSSAAQTEKGGRTQSQLAKSSRPEFDWQDPLVLEEQL
TTDEILIRDTFRTYCQERLMPRILLANRNEVFHREIISEMGELGVLGPTIKGYGCAGVSS
VAYGLLARELERVDSGYRSAMSVQSSLVMHPIYAYGSEEQRQKYLPQLAKGELLGCFGLT
EPNSGSDPSSMETRAHYNSSNKSYTLNGTKTWITNSPMADLFVVWARCEDGCIRGFLLEK
GMRGLSAPRIQGKFSLRASATGMIIMDGVEVPEENVLPGASSLGGPFGCLNNARYGIAWG
VLGASEFCLHTARQYALDRMQFGVPLARNQLIQKKLADMLTEITLGLHACLQLGRLKDQD
KAAPEMVSLLKRNNCGKALDIARQARDMLGGNGISDEYHVIRHAMNLEAVNTYEGTHDIH
ALILGRAITGIQAFTASK
>hsa:2673
MCGIFAYLNYHVPRTRREILETLIKGLQRLEYRGYDSAGVGFDGGNDKDWEANACKIQLI
KKKGKVKALDEEVHKQQDMDLDIEFDVHLGIAHTRWATHGEPSPVNSHPQRSDKNNEFIV
IHNGIITNYKDLKKFLESKGYDFESETDTETIAKLVKYMYDNRESQDTSFTTLVERVIQQ
LEGAFALVFKSVHFPGQAVGTRRGSPLLIGVRSEHKLSTDHIPILYRTARTQIGSKFTRW
GSQGERGKDKKGSCNLSRVDSTTCLFPVEEKAVEYYFASDASAVIEHTNRVIFLEDDDVA
AVVDGRLSIHRIKRTAGDHPGRAVQTLQMELQQIMKGNFSSFMQKEIFEQPESVVNTMRG
RVNFDDYTVNLGGLKDHIKEIQRCRRLILIACGTSYHAGVATRQVLEELTELPVMVELAS
DFLDRNTPVFRDDVCFFLSQSGETADTLMGLRYCKERGALTVGITNTVGSSISRETDCGV
HINAGPEIGVASTKAYTSQFVSLVMFALMMCDDRISMQERRKEIMLGLKRLPDLIKEVLS
MDDEIQKLATELYHQKSVLIMGRGYHYATCLEGALKIKEITYMHSEGILAGELKHGPLAL
VDKLMPVIMIIMRDHTYAKCQNALQQVVARQGRPVVICDKEDTETIKNTKRTIKVPHSVD
CLQGILSVIPLQLLAFHLAVLRGYDVDFPRNLAKSVTVE
>hsa:2677
MAVSAGSARTSPSSDKVQKDKAELISGPRQDSRIGKLLGFEWTDLSSWRRLVTLLNRPTD
PASLAVFRFLFGFLMVLDIPQERGLSSLDRKYLDGLDVCRFPLLDALRPLPLDWMYLVYT
IMFLGALGMMLGLCYRISCVLFLLPYWYVFLLDKTSWNNHSYLYGLLAFQLTFMDANHYW
SVDGLLNAHRRNAHVPLWNYAVLRGQIFIVYFIAGVKKLDADWVEGYSMEYLSRHWLFSP
FKLLLSEELTSLLVVHWGGLLLDLSAGFLLFFDVSRSIGLFFVSYFHCMNSQLFSIGMFS
YVMLASSPLFCSPEWPRKLVSYCPRRLQQLLPLKAAPQPSVSCVYKRSRGKSGQKPGLRH
QLGAAFTLLYLLEQLFLPYSHFLTQGYNNWTNGLYGYSWDMMVHSRSHQHVKITYRDGRT
GELGYLNPGVFTQSRRWKDHADMLKQYATCLSRLLPKYNVTEPQIYFDIWVSINDRFQQR
IFDPRVDIVQAAWSPFQRTSWVQPLLMDLSPWRAKLQEIKSSLDNHTEVVFIADFPGLHL
ENFVSEDLGNTSIQLLQGEVTVELVAEQKNQTLREGEKMQLPAGEYHKVYTTSPSPSCYM
YVYVNTTELALEQDLAYLQELKEKVENGSETGPLPPELQPLLEGEVKGGPEPTPLVQTFL
RRQQRLQEIERRRNTPFHERFFRFLLRKLYVFRRSFLMTCISLRNLILGRPSLEQLAQEV

TYANLRPFEAVGELNPSNTDSSHSNPPESNPDPVHSEF

>hsa:270

MNVRIFYSVSQSPHSLLSLLFYCAILESRISATMPLFKLPAEEKQIDDAMRNFAEKVFAS
EVKDEGGRQEISPFDVDEICPISHHEMQAHIFHLETLSTSTEARRKKRFQGRKTVNLSIP
LSETSSTKLSHIDEYISSSPTYQTVPDFQRVQITGDYASGVTVEDFEIVCKGLYRALCIR
EKYMQKSFQRFPKTPSKYLRNIDGEAWVANESFYPVFTPPVKKGEDPFRTDNLPENLGYH
LKMKDGVVYVYPNEAAVSKDEPKPLPYPNLDTFLDDMNFLLALIAQGPVKTYTHRRLKFL
SSKFQVHQMLNEMDELKELKNNPHRDFYNCRKVDTHIHAAACMNQKHLLRFIKKSYQIDA
DRVVYSTKEKNLTLKELFAKLKMHPYDLTVDSLDVHAGRQTFQRFDKFNDKYNPVGASEL
RDLYLKTDNYINGEYFATIIKEVGADLVEAKYQHAEPRLSIYGRSPDEWSKLSSWFVCNR
IHCPNMTWMIQVPRIYDVFRSKNFLPHFGKMLENIFMPVFEATINPQADPELSVFLKHIT
GFDSVDDESKHSGHMFSSKSPKPQEWTLEKNPSYTYYAYYMYANIMVLNSLRKERGMNTF
LFRPHCGEAGALTHLMTAFMIADDISHGLNLKKSPVLQYLFFLAQIPIAMSPLSNNSLFL
EYAKNPFLDFLQKGLMISLSTDDPMQFHFTKEPLMEEYAIAAQVFKLSTCDMCEVARNSV
LQCGISHEEKVKFLGDNYLEEGPAGNDIRRTNVAQIRMAYRYETWCYELNLIAEGLKSTE

>hsa:27032

MKVARFQKIPNGENETMIPVLTSKKASELPVSEVASILQADLQNGLNKCEVSHRRAFHGW
NEFDISEDEPLWKKYISQFKNPLIMLLLASAVISVLMHQFDDAVSITVAILIVVTVAFVQ
EYRSEKSLEELSKLVPPECHCVREGKLEHTLARDLVPGDTVCLSVGDRVPADLRLFEAVD
LSIDESSLTGETTPCSKVTAPQPAATNGDLASRSNIAFMGTLVRCGKAKGVVIGTGENSE
FGEVFKMMQAEEAPKTPLQKSMDLLGKQLSFYSFGIIGIIMLVGWLLGKDILEMFTISVS
LAVAAIPEGLPIVVTVTLALGVMRMVKKRAIVKKLPIVETLGCCNVICSDKTGTLTKNEM
TVTHIFTSDGLHAEVTGVGYNQFGEVIVDGDVVHGFYNPAVSRIVEAGCVCNDAVIRNNT
LMGKPTEGALIALAMKMGLDGLQQDYIRKAEYPFSSEQKWMAVKCVHRTQQDRPEICFMK
GAYEQVIKYCTTYQSKGQTLTLTQQQRDVYQQEKARMGSAGLRVLALASGPELGQLTFLG
LVGIIDPPRTGVKEAVTTLIASGVSIKMITGDSQETAVAIASRLGLYSKTSQSVSGEEID
AMDVQQLSQIVPKVAVFYRASPRHKMKIIKSLQKNGSVVAMTGDGVNDAVALKAADIGVA
MGQTGTDVCKEAADMILVDDDFQTIMSAIEEGKGIYNNIKNFVRFQLSTSIAALTLISLA
TLMNFPNPLNAMQILWINIIMDGPPAQSLGVEPVDKDVIRKPPRNWKDSILTKNLILKIL
VSSIIIVCGTLFVFWRELRDNVITPRDTTMTFTCFVFFDMFNALSSRSQTKSVFEIGLCS
NRMFCYAVLGSIMGQLLVIYFPPLQKVFQTESLSILGLALGEEWTAAG

>hsa:27034

MLWSGCRRFGARLGCLPGGLRVLVQTGHRSLTSCIDPSMGLNEEQKEFQKVAFDFAAREM
APNMAEWDQKELFPVDVMRKAAQLGFGGVYIQTDVGGSGLSRLDTSVIFEALATGCTSTT
AYISIHNMCAWMIDSFGNEEQRHKFCPPLCTMEKFASYCLTEPGSGSDAASLLTSAKKQG
DHYILNGSKAFISGAGESDIYVVMCRTGGPGPKGISCIVVEKGTPGLSFGKKEKKVGWNS
QPTRAVIFEDCAVPVANRIGSEGQGFLIAVRGLNGGRINIASCSLGAAHASVILTRDHLN
VRKQFGEPLASNQYLQFTLADMATRLVAARLMVRNAAVALQEERKDAVALCSMAKLFATD
ECFAICNQALQMHGGYGYLKDYAVQQYVRDSRVHQILEGSNEVMRILISRSLLQE

>hsa:27115

MSCLMVERCGEILFENPDQNAKCVCMLGDIRLRGQTGVRAERRGSYPFIDFRLLNSTTYS
GEIGTKKKVKRLLSFQRYFHASRLLRGIIPQAPLHLLDEDYLGQARHMLSKVGMWDFDIF
LFDRLTNGNSLVTLLCHLFNTHGLIHHFKLDMVTLHRFLVMVQEDYHSQNPYHNAVHAAD
VTQAMHCYLKEPKLASFLTPLDIMLGLLAAAAHDVDHPGVNQPFLIKTNHHLANLYQNMS

VLENHHWRSTIGMLRESRLLAHLPKEMTQDIEQQLGSLILATDINRQNEFLTRLKAHLHN
KDLRLEDAQDRHFMLQIALKCADICNPCRIWEMSKQWSERVCEEFYRQGELEQKFELEIS
PLCNQQKDSIPSIQIGFMSYIVEPLFREWAHFTGNSTLSENMLGHLAHNKAQWKSLLPRQ
HRSRGSSGSGPDHDHAGQGTESEEQEGDSP
>hsa:2746
MYRYLGEALLLSRAGPAALGSASADSAALLGWARGQPAAAPQPGLALAARRHYSEAVADR
EDDPNFFKMVEGFFDRGASIVEDKLVEDLRTRESEEQKRNRVRGILRIIKPCNHVLSLSF
PIRRDDGSWEVIEGYRAQHSQHRTPCKGGIRYSTDVSVDEVKALASLMTYKCAVVDVPFG
GAKAGVKINPKNYTDNELEKITRRFTMELAKKGFIGPGIDVPAPDMSTGEREMSWIADTY
ASTIGHYDINAHACVTGKPISQGGIHGRISATGRGVFHGIENFINEASYMSILGMTPGFG
DKTFVVQGFGNVGLHSMRYLHRFGAKCIAVGESDGSIWNPDGIDPKELEDFKLQHGSILG
FPKAKPYEGSILEADCDILIPAASEKQLTKSNAPRVKAKIIAEGANGPTTPEADKIFLER
NIMVIPDLYLNAGGVTVSYFEWLKNLNHVSYGRLTFKYERDSNYHLLMSVQESLERKFGK
HGGTIPIVPTAEFQDRISGASEKDIVHSGLAYTMERSARQIMRTAMKYNLGLDLRTAAYV
NAIEKVFKVYNEAGVTFT
>hsa:2766
MPRIDADLKLDFKDVLLRPKRSSLKSRAEVDLERTFTFRNSKQTYSGIPIIVANMDTVGT
FEMAAVMSQHSMFTAIHKHYSLDDWKLFATNHPECLQNVAVSSGSGQNDLEKMTSILEAV
PQVKFICLDVANGYSEHFVEFVKLVRAKFPEHTIMAGNVVTGEMVEELILSGADIIKVGV
GPGSVCTTRTKTGVGYPQLSAVIECADSAHGLKGHIISDGGCTCPGDVAKAFGAGADFVM
LGGMFSGHTECAGEVFERNGRKLKLFYGMSSDTAMNKHAGGVAEYRASEGKTVEVPYKGD
VENTILDILGGLRSTCTYVGAAKLKELSRRATFIRVTQQHNTVFS
>hsa:279
MKFFLLLFTIGFCWAQYSPNTQQGRTSIVHLFEWRWVDIALECERYLAPKGFGGVQVSPP
NENVAIYNPFRPWWERYQPVSYKLCTRSGNEDEFRNMVTRCNNVGVRIYVDAVINHMCGN
AVSAGTSSTCGSYFNPGSRDFPAVPYSGWDFNDGKCKTGSGDIENYNDATQVRDCRLTGL
LDLALEKDYVRSKIAEYMNHLIDIGVAGFRLDASKHMWPGDIKAILDKLHNLNSNWFPAG
SKPFIYQEVIDLGGEPIKSSDYFGNGRVTEFKYGAKLGTVIRKWNGEKMSYLKNWGEGWG
FVPSDRALVFVDNHDNQRGHGAGGASILTFWDARLYKMAVGFMLAHPYGFTRVMSSYRWP
RQFQNGNDVNDWVGPPNNNGVIKEVTINPDTTCGNDWVCEHRWRQIRNMVIFRNVVDGQP
FTNWYDNGSNQVAFGRGNRGFIVFNNDDWSFSLTLQTGLPAGTYCDVISGDKINGNCTGI
KIYVSDDGKAHFSISNSAEDPFIAIHAESKL
>hsa:28
MAEVLRTLAGKPKCHALRPMILFLIMLVLVLFGYGVLSPRSLMPGSLERGFCMAVREPDH
LQRVSLPRMVYPQPKVLTPCRKDVLVVTPWLAPIVWEGTFNIDILNEQFRLQNTTIGLTV
FAIKKYVAFLKLFLETAEKHFMVGHRVHYYVFTDQPAAVPRVTLGTGRQLSVLEVRAYKR
WQDVSMRRMEMISDFCERRFLSEVDYLVCVDVDMEFRDHVGVEILTPLFGTLHPGFYGSS
REAFTYERRPQSQAYIPKDEGDFYYLGGFFGGSVQEVQRLTRACHQAMMVDQANGIEAVW
HDESHLNKYLLRHKPTKVLSPEYLWDQQLLGWPAVLRKLRFTAVPKNHQAVRNP
>hsa:2806
MALLHSGRVLPGIAAAFHPGLAAAASARASSWWTHVEMGPPDPILGVTEAFKRDTNSKKM
NLGVGAYRDDNGKPYVLPSVRKAEAQIAAKNLDKEYLPIGGLAEFCKASAELALGENSEV
LKSGRFVTVQTISGTGALRIGASFLQRFFKFSRDVFLPKPTWGNHTPIFRDAGMQLQGYR
YYDPKTCGFDFTGAVEDISKIPEQSVLLLHACAHNPTGVDPRPEQWKEIATVVKKRNLFA

FFDMAYQGFASGDGDKDAWAVRHFIEQGINVCLCQSYAKNMGLYGERVGAFTMVCKDADE
AKRVESQLKILIRPMYSNPPLNGARIAAAILNTPDLRKQWLQEVKVMADRIIGMRTQLVS
NLKKEGSTHNWQHITDQIGMFCFTGLKPEQVERLIKEFSIYMTKDGRISVAGVTSSNVGY
LAHAIHQVTK
>hsa:2822
MSAFRLWPGLLIMLGSLCHRGSPCGLSTHVEIGHRALEFLQLHNGRVNYRELLLEHQDAY
QAGIVFPDCFYPSICKGGKFHDVSESTHWTPFLNASVHYIRENYPLPWEKDTEKLVAFLF
GITSHMAADVSWHSLGLEQGFLRTMGAIDFHGSYSEAHSAGDFGGDVLSQFEFNFNYLAR
RWYVPVKDLLGIYEKLYGRKVITENVIVDCSHIQFLEMYGEMLAVSKLYPTYSTKSPFLV
EQFQEYFLGGLDDMAFWSTNIYHLTSFMLENGTSDCNLPENPLFIACGGQQNHTQGSKMQ
KNDFHRNLTTSLTESVDRNINYTERGVFFSVNSWTPDSMSFIYKALERNIRTMFIGGSQL
SQKHVSSPLASYFLSFPYARLGWAMTSADLNQDGHGDLVVGAPGYSRPGHIHIGRVYLIY
GNDLGLPPVDLDLDKEAHRILEGFQPSGRFGSALAVLDFNVDGVPDLAVGAPSVGSEQLT
YKGAVYVYFGSKQGGMSSSPNITISCQDIYCNLGWTLLAADVNGDSEPDLVIGSPFAPGG
GKQKGIVAAFYSGPSLSDKEKLNVEAANWTVRGEEDFSWFGYSLHGVTVDNRTLLLVGSP
TWKNASRLGHLLHIRDEKKSLGRVYGYFPPNGQSWFTISGDKAMGKLGTSLSSGHVLMNG
TLKQVLLVGAPTYDDVSKVAFLTVTLHQGGATRMYALTSDAQPLLLSTFSGDRRFSRFGG
VLHLSDLDDDGLDEIIMAAPLRIADVTSGLIGGEDGRVYVYNGKETTLGDMTGKCKSWIT
PCPEEKAQYVLISPEASSRFGSSLITVRSKAKNQVVIAAGRSSLGARLSGALHVYSLGSD
>hsa:28227
MPPGKVLQPVLKMKVDELFLYWLSEASTQRMLQDCLRRIKAPGRDQPTPGDGEQPGAWPT
APLAAPRPSGLEPPGTPGPGPALPLGAASSPRNAPHVRGTRRSAGTRVVQTRKEEPLPPA
TSQSIPTFYFPRGRPQDSVNVDAVISKIESTFARFPHERATMDDMGLVAKACGCPLYWKG
PLFYGAGGERTGSVSVHKFVAMWRKILQNCHDDAAKFVHLLMSPGCNYLVQEDFVPFLQD
VVNTHPGLSFLKEASEFHSRYITTVIQRIFYAVNRSWSGRITCAELRRSSFLQNVALLEE
EADINQLTEFFSYEHFYVIYCKFWELDTDHDLLIDADDLARHNDHALSTKMIDRIFSGAV
TRGRKVQKEGKISYADFVWFLISEEDKKTPTSIEYWFRCMDLDGDGALSMFELEYFYEEQ
CRRLDSMAIEALPFQDCLCQMLDLVKPRTEGKITLQDLKRCKLANVFFDTFFNIEKYLDH
EQKEQISLLRDGDSGGPELSDWEKYAAEEYDILVAEETAGEPWEDGFEAELSPVEQKLSA
LRSPLAQRPFFEAPSPLGAVDLYEYACGDEDLEPL
>hsa:284541
MSVSVLSPSRRLGGVSGILQVTSLLILLLLLIKAAQLYLHRQWLLKALQQFPCPPSHWLF
GHIQEFQHDQELQRIQERVKTFPSACPYWIWGGKVRVQLYDPDYMKVILGRSDPKSHGSY
KFLAPRIGYGLLLLNGQTWFQHRRMLTPAFHNDILKPYVGLMADSVRVMLDKWEELLGQD
SPLEVFQHVSLMTLDTIMKSAFSHQGSIQVDRNSQSYIQAISDLNSLVFCCMRNAFHEND
TIYSLTSAGRWTHRACQLAHQHTDQVIQLRKAQLQKEGELEKIKRKRHLDFLDILLLAKM
ENGSILSDKDLRAEVDTFMFEGHDTTASGISWILYALATHPKHQERCREEIHGLLGDGAS
ITWNHLDQMPYTTMCIKEALRLYPPVPGIGRELSTPVTFPDGRSLPKGIMVLLSIYGLHH
NPKVWPNLEVFDPSRFAPGSAQHSHAFLPFSGGSRNCIGKQFAMNQLKVARALTLLRFEL
LPDPTRIPIPMARLVLKSKNGIHLRLRRLPNPCEDKDQL
>hsa:285220
MQFPSPPAARSSPAPQAASSSEAAAPATGQPGPSCPVPGTSRRGRPGTPPAGRVEEEEEE
EEEDVDKDPHPTQNTCLRCRHFSLRERKREPRRTMGGCEVREFLLQFGFFLPLLTAWPGD
CSHVSNNQVVLLDTTTVLGELGWKTYPLNGWDAITEMDEHNRPIHTYQVCNVMEPNQNNW

LRTNWISRDAAQKIYVEMKFTLRDCNSIPWVLGTCKETFNLFYMESDESHGIKFKPNQYT
KIDTIAADESFTQMDLGDRILKLNTEIREVGPIERKGFYLAFQDIGACIALVSVRVFYKK
CPFTVRNLAMFPDTIPRVDSSSLVEVRGSCVKSAEERDTPKLYCGADGDWLVPLGRCICS
TGYEEIEGSCHACRPGFYKAFAGNTKCSKCPPHSLTYMEATSVCQCEKGYFRAEKDPPSM
ACTRPPSAPRNVVFNINETALILEWSPPSDTGGRKDLTYSVICKKCGLDTSQCEDCGGGL
RFIPRHTGLINNSVIVLDFVSHVNYTFEIEAMNGVSELSFSPKPFTAITVTTDQDAPSLI
GVVRKDWASQNSIALSWQAPAFSNGAILDYEIKYYEKEHEQLTYSSTRSKAPSVIITGLK
PATKYVFHIRVRTATGYSGYSQKFEFETGDETSDMAAEQGQILVIATAAVGGFTLLVILT
LFFLITGRCQWYIKAKMKSEEKRRNHLQNGHLRFPGIKTYIDPDTYEDPSLAVHEFAKEI
DPSRIRIERVIGAGEFGEVCSGRLKTPGKREIPVAIKTLKGGHMDRQRRDFLREASIMGQ
FDHPNIIRLEGVVTKRSFPAIGVEAFCPSFLRAGFLNSIQAPHPVPGGGSLPPRIPAGRP
VMIVVEYMENGSLDSFLRKHDGHFTVIQLVGMLRGIASGMKYLSDMGYVHRDLAARNILV
NSNLVCKVSDFGLSRVLEDDPEAAYTTTGGKIPIRWTAPEAIAYRKFSSASDAWSYGIVM
WEVMSYGERPYWEMSNQDVILSIEEGYRLPAPMGCPASLHQLMLHCWQKERNHRPKFTDI
VSFLDKLIRNPSALHTLVEDILVMPESPGEVPEYPLFVTVGDWLDSIKMGQYKNNFVAAG
FTTFDLISRMSIDDIRRIGVILIGHQRRIVSSIQTLRLHMMHIQEKGFHV
>hsa:2880
MTTQLRVVHLLPLLLACFVQTSPKQEKMKMDCHKDEKGTIYDYEAIALNKNEYVSFKQYV
GKHILFVNVATYCGLTAQYPELNALQEELKPYGLVVLGFPCNQFGKQEPGDNKEILPGLK
YVRPGGGFVPSFQLFEKGDVNGEKEQKVFSFLKHSCPHPSEILGTFKSISWDPVKVHDIR
WNFEKFLVGPDGIPVMRWSHRATVSSVKTDILAYLKQFKTK
>hsa:2882
MVAATVAAAWLLLWAAACAQQEQDFYDFKAVNIRGKLVSLEKYRGSVSLVVNVASECGFT
DQHYRALQQLQRDLGPHHFNVLAFPCNQFGQQEPDSNKEIESFARRTYSVSFPMFSKIAV
TGTGAHPAFKYLAQTSGKEPTWNFWKYLVAPDGKVVGAWDPTVSVEEVRPQITALVRKLI
LLKREDL
>hsa:28972
MARGGDTGCTGPSETSASGAAAIALPGLEGPATDAQCQTLPLTVLKSRSPSPRSLPPALS
CPPPQPAMLEHLSSLPTQMDYKGQKLAEQMFQGIILFSAIVGFIYGYVAEQFGWTVYIVM
AGFAFSCLLTLPPWPIYRRHPLKWLPVQESSTDDKKPGERKIKRHAKNN
>hsa:2936
MALLPRALSAGAGPSWRRAARAFRGFLLLLPEPAALTRALSRAMACRQEPQPQGPPPAAG
AVASYDYLVIGGGSGGLASARRAAELGARAAVVESHKLGGTCVNVGCVPKKVMWNTAVHS
EFMHDHADYGFPSCEGKFNWRVIKEKRDAYVSRLNAIYQNNLTKSHIEIIRGHAAFTSDP
KPTIEVSGKKYTAPHILIATGGMPSTPHESQIPGASLGITSDGFFQLEELPGRSVIVGAG
YIAVEMAGILSALGSKTSLMIRHDKVLRSFDSMISTNCTEELENAGVEVLKFSQVKEVKK
TLSGLEVSMVTAVPGRLPVMTMIPDVDCLLWAIGRVPNTKDLSLNKLGIQTDDKGHIIVD
EFQNTNVKGIYAVGDVCGKALLTPVAIAAGRKLAHRLFEYKEDSKLDYNNIPTVVFSHPP
IGTVGLTEDEAIHKYGIENVKTYSTSFTPMYHAVTKRKTKCVMKMVCANKEEKVVGIHMQ
GLGCDEMLQGFAVAVKMGATKADFDNTVAIHPTSSEELVTLR
>hsa:2950
MPPYTVVYFPVRGRCAALRMLLADQGQSWKEEVVTVETWQEGSLKASCLYGQLPKFQDGD
LTLYQSNTILRHLGRTLGLYGKDQQEAALVDMVNDGVEDLRCKYISLIYTNYEAGKDDYV
KALPGQLKPFETLLSQNQGGKTFIVGDQISFADYNLLDLLLIHEVLAPGCLDAFPLLSAY

VGRLSARPKLKAFLASPEYVNLPINGNGKQ
>hsa:29785
MEATGTWALLLALALLLLLLTLALSGTRARGHLPPGPTPLPLLGNLLQLRPGALYSGLMRL
SKKYGPVFTIYLGPWRPVVVLVGQEAVREALGGQAEEFSGRGTVAMLEGTFDGHGVFFSN
GERWRQLRKFTMLALRDLGMGKREGEELIQAEARCLVETFQGTEGRPFDPSLLLAQATSN
VVCSLLFGLRFSYEDKEFQAVVRAAGGTLLGVSSQGGQTYEMFSWFLRPLPGPHKQLLHH
VSTLAAFTVRQVQQHQGNLDASGPARDLVDAFLLKMAQEEQNPGTEFTNKNMLMTVIYLL
FAGTMTVSTTVGYTLLLLMKYPHVQKWVREELNRELGAGQAPSLGDRTRLPYTDAVLHEA
QRLLALVPMGIPRTLMRTTRFRGYTLPQGTEVFPLLGSILHDPNIFKHPEEFNPDRFLDA
DGRFRKHEAFLPFSLGKRVCLGEGLAKAELFLFFTTILQAFSLESPCPPDTLSLKPTVSG
LFNIPPAFQLQVRPTDLHSTTQTR
>hsa:29920
MSVGFIGAGQLAYALARGFTAAGILSAHKIIASSPEMNLPTVSALRKMGVNLTRSNKETV
KHSDVLFLAVKPHIIPFILDEIGADVQARHIVVSCAAGVTISSVEKKLMAFQPAPKVIRC
MTNTPVVVQEGATVYATGTHALVEDGQLLEQLMSSVGFCTEVEEDLIDAVTGLSGSGPAY
AFMALDALADGGVKMGLPRRLAIQLGAQALLGAAKMLLDSEQHPCQLKDNVCSPGGATIH
ALHFLESGGFRSLLINAVEASCIRTRELQSMADQEKISPAALKKTLLDRVKLESPTVSTL
TPSSPGKLLTRSLALGGKKD
>hsa:29941
MEEGAPRQPGPSQWPPEDEKEVIRRAIQKELKIKEGVENLRRVATDRRHLGHVQQLLRSS
NRRLEQLHGELRELHARILLPGPGPGPAEPVASGPRPWAEQLRARHLEALRRQLHVELKV
KQGAENMTHTCASGTPKERKLLAAAQQMLRDSQLKVALLRMKISSLEASGSPEPGPELLA
EELQHRLHVEAAVAEGAKNVVKLLSSRRTQDRKALAEAQAQLQESSQKLDLLRLALEQLL
EQLPPAHPLRSRVTRELRAAVPGYPQPSGTPVKPTALTGTLQVRLLGCEQLLTAVPGRSP
AAALASSPSEGWLRTKAKHQRGRGELASEVLAVLKVDNRVVGQTGWGQVAEQSWDQTFVI
PLERARELEIGVHWRDWRQLCGVAFLRLEDFLDNACHQLSLSLVPQGLLFAQVTFCDPVI
ERRPRLQRQERIFSKRRGQDFLRASQMNLGMAAWGRLVMNLLPPCSSPSTISPPKGCPRT
PTTLREASDPATPSNFLPKKTPLGEEMTPPPKPPRLYLPQEPTSEETPRTKRPHMEPRTR
RGPSPPASPTRKPPRLQDFRCLAVLGRGHFGKVLLVQFKGTGKYYAIKALKKQEVLSRDE
IESLYCEKRILEAVGCTGHPFLLSLLACFQTSSHACFVTEFVPGGDLMMQIHEDVFPEPQ
ARFYVACVVLGLQFLHEKKIIYRDLKLDNLLLDAQGFLKIADFGLCKEGIGFGDRTSTFC
GTPEFLAPEVLTQEAYTRAVDWWGLGVLLYEMLVGECPFPGDTEEEVFDCIVNMDAPYPG
FLSVQGLEFIQKLLQKCPEKRLGAGEQDAEEIKVQPFFRTTNWQALLARTIQPPFVPTLC
GPADLRYFEGEFTGLPPALTPPAPHSLLTARQQAAFRDFDFVSERFLEP
>hsa:29968
MDAPRQVVNFGPGPAKLPHSVLLEIQKELLDYKGVGISVLEMSHRSSDFAKIINNTENLV
RELLAVPDNYKVIFLQGGGCGQFSAVPLNLIGLKAGRCADYVVTGAWSAKAAEEAKKFGT
INIVHPKLGSYTKIPDPSTWNLNPDASYVYYCANETVHGVEFDFIPDVKGAVLVCDMSSN
FLSKPVDVSKFGVIFAGAQKNVGSAGVTVVIVRDDLLGFALRECPSVLEYKVQAGNSSLY
NTPPCFSIYVMGLVLEWIKNNGGAAAMEKLSSIKSQTIYEIIDNSQGFYVSVGGIRASLY
NAVTIEDVQKLAAFMKKFLEMHQL
>hsa:30
MQRLQVVLGHLRGPADSGWMPQAAPCLSGAPQASAADVVVVHGRRTAICRAGRGGFKDTT
PDELLSAVMTAVLKDVNLRPEQLGDICVGNVLQPGAGAIMARIAQFLSDIPETVPLSTVN

RQCSSGLQAVASIAGGIRNGSYDIGMACGITSENVAERFGISREKQDTFALASQQKAARA
QSKGCFQAEIVPVTTTVHDDKGTKRSITVTQDEGIRPSTTMEGLAKLKPAFKKDGSTTAG
LTVSDVDIFEINEAFASQAAYCVEKLRLPPEKVNPLGGAVALGHPLGCTGARQVITLLNE
LKRRGKRAYGVVSMCIGTGMGAAAVFEYPGN
>hsa:3001
MRNSYRFLASSLSVVVSLLLIPEDVCEKIIGGNEVTPHSRPYMVLLSLDRKTICAGALIA
KDWVLTAAHCNLNKRSQVILGAHSITREEPTKQIMLVKKEFPYPCYDPATREGDLKLLQL
TEKAKINKYVTILHLPKKGDDVKPGTMCQVAGWGRTHNSASWSDTLREVNITIIDRKVCN
DRNHYNFNPVIGMNMVCAGSLRGGRDSCNGDSGSPLLCEGVFRGVTSFGLENKCGDPRGP
GVYILLSKKHLNWIIMTIKGAV
>hsa:3002
MQPILLLLAFLLLPRADAGEIIGGHEAKPHSRPYMAYLMIWDQKSLKRCGGFLIRDDFVL
TAAHCWGSSINVTLGAHNIKEQEPTQQFIPVKRPIPHPAYNPKNFSNDIMLLQLERKAKR
TRAVQPLRLPSNKAQVKPGQTCSVAGWGQTAPLGKHSHTLQEVKMTVQEDRKCESDLRHY
YDSTIELCVGDPEIKKTSFKGDSGGPLVCNKVAQGIVSYGRNNGMPPRACTKVSSFVHWI
KKTMKRY
>hsa:3028
MAAACRSVKGLVAVITGGASGLGLATAERLVGQGASAVLLDLPNSGGEAQAKKLGNNCVF
APADVTSEKDVQTALALAKGKFGRVDVAVNCAGIAVASKTYNLKKGQTHTLEDFQRVLDV
NLMGTFNVIRLVAGEMGQNEPDQGGQRGVIINTASVAAFEGQVGQAAYSASKGGIVGMTL
PIARDLAPIGLFGTPLLTSLPEKVCNFLASQVPFPSRLGDPAEYAHLVQAIIENPFLNGE
VIRLDGAIRMQP
>hsa:3033
MAFVTRQFMRSVSSSSTASASAKKIIVKHVTVIGGGLMGAGIAQVAAATGHTVVLVDQTE
DILAKSKKGIEESLRKVAKKKFAENPKAGDEFVEKTLSTIATSTDAASVVHSTDLVVEAI
VENLKVKNELFKRLDKFAAEHTIFASNTSSLQITSIANATTRQDRFAGLHFFNPVPVMKL
VEVIKTPMTSQKTFESLVDFSKALGKHPVSCKDTPGFIVNRLLVPYLMEAIRLYERDFQT
CGDSNSGLGFSLKGDASKEDIDTAMKLGAGYPMGPFELLDYVGLDTTKFIVDGWHEMDAE
NPLHQPSPSLNKLVAENKFGKKTGEGFYKYK
>hsa:3034
MLLALRINVLAKGYSGISLETLKQVIEMFNASCLPYVPEKGTVGASGDLAPLSHLALGLV
GEGKMWSPKSGWADAKYVLEAHGLKPVILKPKEGLALINGTQMITSLGCEAVERASAIAR
QADIVAALTLEVLKGTTKAFDTDIHALRPHRGQIEVAFRFRSLLDSDHHPSEIAESHRFC
DRVQDAYTLRCCPQVHGVVNDTIAFVKNIITTELNSATDNPMVFANRGETVSGGNFHGEY
PAKALDYLAIGIHELAAISERRIERLCNPSLSELPAFLVAEGGLNSGFMIAHCTAAALVS
ENKALCHPSSVDSLSTSAATEDHVSMGGWAARKALRVIEHVEQVLAIELLAACQGIEFLR
PLKTTTPLEKVYDLVRSVVRPWIKDRFMAPDIEAAHRLLLEQKVWEVAAPYIEKYRMEHI
PESRPLSPTAFSLQFLHKKSTKIPESEDL
>hsa:3035
MAERAALEELVKLQGERVRGLKQQKASAELIEEEVAKLLKLKAQLGPDESKQKFVLKTPK
ETLMGKYGEDSKLIYDLKDQGGELLSLRYDLTVPFARYLAMNKLTNIKRYHIAKVYRRDN
PAMTRGRYREFYQCDFDIAGNFDPMIPDAECLKIMCEILSSLQIGDFLVKVNDRRILDGM
FAICGVSDSKFRTICSSVDKLDKVSWEEVKNEMVGEKGLAPEVADRIGDYVQQHGGVSLV
EQLLQDPKLSQNKQALEGLGDLKLLFEYLTLFGIDDKISFDLSLARGLDYYTGVIYEAVL

LQTPAQAGEEPLGVGSVAAGGRYDGLVGMFDPKGRKVPCVGLSIGVERIFSIVEQRLEAL
EEKIRTTETQVLVASAQKKLLEERLKLVSELWDAGIKAELLYKKNPKLLNQLQYCEEAGI
PLVAIIGEQELKDGVIKLRSVTSREEVDVRREDLVEEIKRRTGQPLCIC
>hsa:3055
MGCMKSKFLQVGGNTFSKTETSASPHCPVYVPDPTSTIKPGPNSHNSNTPGIREAGSEDI
IVVALYDYEAIHHEDLSFQKGDQMVVLEESGEWWKARSLATRKEGYIPSNYVARVDSLET
EEWFFKGISRKDAERQLLAPGNMLGSFMIRDSETTKGSYSLSVRDYDPRQGDTVKHYKIR
TLDNGGFYISPRSTFSTLQELVDHYKKGNDGLCQKLSVPCMSSKPQKPWEKDAWEIPRES
LKLEKKLGAGQFGEVWMATYNKHTKVAVKTMKPGSMSVEAFLAEANVMKTLQHDKLVKLH
AVVTKEPIYIITEFMAKGSLLDFLKSDEGSKQPLPKLIDFSAQIAEGMAFIEQRNYIHRD
LRAANILVSASLVCKIADFGLARVIEDNEYTAREGAKFPIKWTAPEAINFGSFTIKSDVW
SFGILLMEIVTYGRIPYPGMSNPEVIRALERGYRMPRPENCPEELYNIMMRCWKNRPEER
PTFEYIQSVLDDFYTATESQYQQQP
>hsa:306
MASIWVGHRGTVRDYPDFSPSVDAEAIQKAIRGIGTDEKMLISILTERSNAQRQLIVKEY
QAAYGKELKDDLKGDLSGHFEHLMVALVTPPAVFDAKQLKKSMKGAGTNEDALIEILTTR
TSRQMKDISQAYYTVYKKSLGDDISSETSGDFRKALLTLADGRRDESLKVDEHLAKQDAQ
ILYKAGENRWGTDEDKFTEILCLRSFPQLKLTFDEYRNISQKDIVDSIKGELSGHFEDLL
LAIVNCVRNTPAFLAERLHRALKGIGTDEFTLNRIMVSRSEIDLLDIRTEFKKHYGYSLY
SAIKSDTSGDYEITLLKICGGDD
>hsa:3067
MMEPEEYRERGREMVDYICQYLSTVRERRVTPDVQPGYLRAQLPESAPEDPDSWDSIFGD
IERIIMPGVVHWQSPHMHAYYPALTSWPSLLGDMLADAINCLGFTWASSPACTELEMNVM
DWLAKMLGLPEHFLHHHPSSQGGGVLQSTVSESTLIALLAARKNKILEMKTSEPDADESC
LNARLVAYASDQAHSSVEKAGLISLVKMKFLPVDDNFSLRGEALQKAIEEDKQRGLVPVF
VCATLGTTGVCAFDCLSELGPICAREGLWLHIDAAYAGTAFLCPEFRGFLKGIEYADSFT
FNPSKWMMVHFDCTGFWVKDKYKLQQTFSVNPIYLRHANSGVATDFMHWQIPLSRRFRSV
KLWFVIRSFGVKNLQAHVRHGTEMAKYFESLVRNDPSFEIPAKRHLGLVVFRLKGPNCLT
ENVLKEIAKAGRLFLIPATIQDKLIIRFTVTSQFTTRDDILRDWNLIRDAATLILSQHCT
SQPSPRVGNLISQIRGARAWACGTSLQSVSGAGDDPVQARKIIKQPQRVGAGPMKRENGL
HLETLLDPVDDCFSEEAPDATKHKLSSFLFSYLSVQTKKKTVRSLSCNSVPVSAQKPLPT
EASVKNGGSSRVRIFSRFPEDMMMLKKSAFKKLIKFYSVPSFPECSSQCGLQLPCCPLQA
MV
>hsa:30814
MKSPHVLVFLCLLVALVTGNLVQFGVMIEKMTGKSALQYNDYGCYCGIGGSHWPVDQTDW
CCHAHDCCYGRLEKLGCEPKLEKYLFSVSERGIFCAGRTTCQRLTCECDKRAALCFRRNL
GTYNRKYAHYPNKLCTGPTPPC
>hsa:30833
MARSVRVLVDMDGVLADFEAGLLRGFRRRFPEEPHVPLEQRRGFLAREQYRALRPDLADK
VASVYEAPGFFLDLEPIPGALDAVREMNDLPDTQVFICTSPLLKYHHCVGEKEETPSWEH
ILFTCCHNRHLVLPPTRRRLLSWSDNWREILDSKRGAAQRE
>hsa:31
MWWSTLMSILRARSFWKWISTQTVRIIRAVRAHFGGIMDEPSPLAQPLELNQHSRFIIGS
VSEDNSEDEISNLVKLDLLEEKEGSLSPASVGSDTLSDLGISSLQDGLALHIRSSMSGLH

LVKQGRDRKKIDSQRDFTVASPAEFVTRFGGNKVIEKVLIANNGIAAVKCMRSIRRWSYE
MFRNERAIRFVVMVTPEDLKANAEYIKMADHYVPVPGGPNNNNYANVELILDIAKRIPVQ
AVWAGWGHASENPKLPELLLKNGIAFMGPPSQAMWALGDKIASSIVAQTAGIPTLPWSGS
GLRVDWQENDFSKRILNVPQELYEKGYVKDVDDGLQAAEEVGYPVMIKASEGGGGKGIRK
VNNADDFPNLFRQVQAEVPGSPIFVMRLAKQSRHLEVQILADQYGNAISLFGRDCSVQRR
HQKIIEEAPATIATPAVFEHMEQCAVKLAKMVGYVSAGTVEYLYSQDGSFYFLELNPRLQ
VEHPCTEMVADVNLPAAQLQIAMGIPLYRIKDIRMMYGVSPWGDSPIDFEDSAHVPCPRG
HVIAARITSENPDEGFKPSSGTVQELNFRSNKNVWGYFSVAAAGGLHEFADSQFGHCFSW
GENREEAISNMVVALKELSIRGDFRTTVEYLIKLLETESFQMNRIDTGWLDRLIAEKVQA
ERPDTMLGVVCGALHVADVSLRNSVSNFLHSLERGQVLPAHTLLNTVDVELIYEGVKYVL
KVTRQSPNSYVVIMNGSCVEVDVHRLSDGGLLLSYDGSSYTTYMKEEVDRYRITIGNKTC
VFEKENDPSVMRSPSAGKLIQYIVEDGGHVFAGQCYAEIEVMKMVMTLTAVESGCIHYVK
RPGAALDPGCVLAKMQLDNPSKVQQAELHTGSLPRIQSTALRGEKLHRVFHYVLDNLVNV
MNGYCLPDPFFSSKVKDWVERLMKTLRDPSLPLLELQDIMTSVSGRIPPNVEKSIKKEMA
QYASNITSVLCQFPSQQIANILDSHAATLNRKSEREVFFMNTQSIVQLVQRYRSGIRGHM
KAVVMDLLRQYLRVETQFQNGHYDKCVFALREENKSDMNTVLNYIFSHAQVTKKNLLVTM
LIDQLCGRDPTLTDELLNILTELTQLSKTTNAKVALRARQVLIASHLPSYELRHNQVESI
FLSAIDMYGHQFCIENLQKLILSETSIFDVLPNFFYHSNQVVRMAALEVYVRRAYIAYEL
NSVQHRQLKDNTCVVEFQFMLPTSHPNRGNIPTLNRMSFSSNLNHYGMTHVASVSDVLLD
NSFTPPCQRMGGMVSFRTFEDFVRIFDEVMGCFSDSPPQSPTFPEAGHTSLYDEDKVPRD
EPIHILNVAIKTDCDIEDDRLAAMFREFTQQNKATLVDHGIRRLTFLVAQKDFRKQVNYE
VDRRFHREFPKFFTFRARDKFEEDRIYRHLEPALAFQLELNRMRNFDLTAIPCANHKMHL
YLGAAKVEVGTEVTDYRFFVRAIIRHSDLVTKEASFEYLQNEGERLLLEAMDELEVAFNN
TNVRTDCNHIFLNFVPTVIMDPSKIEESVRSMVMRYGSRLWKLRVLQAELKINIRLTPTG
KAIPIRLFLTNESGYYLDISLYKEVTDSRTAQIMFQAYGDKQGPLHGMLINTPYVTKDLL
QSKRFQAQSLGTTYIYDIPEMFRQSLIKLWESMSTQAFLPSPPLPSDMLTYTELVLDDQG
QLVHMNRLPGGNEIGMVAWKMTFKSPEYPEGRDIIVIGNDITYRIGSFGPQEDLLFLRAS
ELARAEGIPRIYVSANSGARIGLAEEIRHMFHVAWVDPEDPYKGYRYLYLTPQDYKRVSA
LNSVHCEHVEDEGESRYKITDIIGKEEGIGPENLRGSGMIAGESSLAYNEIITISLVTCR
AIGIGAYLVRLGQRTIQVENSHLILTGAGALNKVLGREVYTSNNQLGGIQIMHNNGVTHC
TVCDDFEGVFTVLHWLSYMPKSVHSSVPLLNSKDPIDRIIEFVPTKTPYDPRWMLAGRPH
PTQKGQWLSGFFDYGSFSEIMQPWAQTVVVGRARLGGIPVGVVAVETRTVELSIPADPAN
LDSEAKIIQQAGQVWFPDSAFKTYQAIKDFNREGLPLMVFANWRGFSGGMKDMYDQVLKF
GAYIVDGLRECCQPVLVYIPPQAELRGGSWVVIDSSINPRHMEMYADRESRGSVLEPEGT
VEIKFRRKDLVKTMRRVDPVYIHLAERLGTPELSTAERKELENKLKEREEFLIPIYHQVA
VQFADLHDTPGRMQEKGVISDILDWKTSRTFFYWRLRRLLLEDLVKKKIHNANPELTDGQ
IQAMLRRWFVEVEGTVKAYVWDNNKDLAEWLEKQLTEEDGVHSVIEENIKCISRDYVLKQ
IRSLVQANPEVAMDSIIHMTQHISPTQRAEVIRILSTMDSPST
>hsa:313
MQSPWKILTVAPLFLLLSLQSSASPANDDQSRPSLSNGHTCVGCVLVVSVIEQLAQVHNS
TVQASMERLCSYLPEKLFLKTTCYLVIDKFGSDIIKLLSADMNADVVCHTLEFCKQNTGQ
PLCHLYPLPKETWKFTLQKARQIVKKSPILKYSRSGSDICSLPVLAKICQKIKLAMEQSV
PFKDVDSDKYSVFPTLRGYHWRGRDCNDSDESVYPGRRPNNWDVHQDSNCNGIWGVDPKD
GVPYEKKFCEGSQPRGIILLGDSAGAHFHISPEWITASQMSLNSFINLPTALTNELDWPQ

LSGATGFLDSTVGIKEKSIYLRLWKRNHCNHRDYQNISRNGASSRNLKKFIESLSRNKVL
DYPAIVIYAMIGNDVCSGKSDPVPAMTTPEKLYSNVMQTLKHLNSHLPNGSHVILYGLPD
GTFLWDNLHNRYHPLGQLNKDMTYAQLYSFLNCLQVSPCHGWMSSNKTLRTLTSERAEQL
SNTLKKIAASEKFTNFNLFYMDFAFHEIIQEWQKRGGQPWQLIEPVDGFHPNERKSILLK
GILMTSQTLDNQEWTQLTSWIKGNLTCDSRTREWLCCCWRIISGKRCSSSGPKSWERRIR
STPRLNRCLETKAGTEPLRSMHPWGAQGGRGLGKLIPQTLWGLPRHRPKGLFFSSIFAKC
LSLNEEHIWTTVQCCVLPGSVTLPLFLK
>hsa:3141
MEDRLHMDNGLVPQKIVSVHLQDSTLKEVKDQVSNKQAQILEPKPEPSLEIKPEQDGMEH
VGRDDPKALGEEPKQRRGSASGSEPAGDSDRGGGPVEHYHLHLSSCHECLELENSTIESV
KFASAENIPDLPYDYSSSLESVADETSPEREGRRVNLTGKAPNILLYVGSDSQEALGRFH
EVRSVLADCVDIDSYILYHLLEDSALRDPWTDNCLLLVIATRESIPEDLYQKFMAYLSQG
GKVLGLSSSFTFGGFQVTSKGALHKTVQNLVFSKADQSEVKLSVLSSGCRYQEGPVRLSP
GRLQGHLENEDKDRMIVHVPFGTRGGEAVLCQVHLELPPSSNIVQTPEDFNLLKSSNFRR
YEVLREILTTLGLSCDMKQVPALTPLYLLSAAEEIRDPLMQWLGKHVDSEGEIKSGQLSL
RFVSSYVSEVEITPSCIPVVTNMEAFSSEHFNLEIYRQNLQTKQLGKVILFAEVTPTTMR
LLDGLMFQTPQEMGLIVIAARQTEGKGRGGNVWLSPVGCALSTLLISIPLRSQLGQRIPF
VQHLMSVAVVEAVRSIPEYQDINLRVKWPNDIYYSDLMKIGGVLVNSTLMGETFYILIGC
GFNVTNSNPTICINDLITEYNKQHKAELKPLRADYLIARVVTVLEKLIKEFQDKGPNSVL
PLYYRYWVHSGQQVHLGSAEGPKVSIVGLDDSGFLQVHQEGGEVVTVHPDGNSFDMLRNL
ILPKRR
>hsa:3156
MLSRLFRMHGLFVASHPWEVIVGTVTLTICMMSMNMFTGNNKICGWNYECPKFEEDVLSS
DIIILTITRCIAILYIYFQFQNLRQLGSKYILGIAGLFTIFSSFVFSTVVIHFLDKELTG
LNEALPFFLLLIDLSRASTLAKFALSSNSQDEVRENIARGMAILGPTFTLDALVECLVIG
VGTMSGVRQLEIMCCFGCMSVLANYFVFMTFFPACVSLVLELSRESREGRPIWQLSHFAR
VLEEEENKPNPVTQRVKMIMSLGLVLVHAHSRWIADPSPQNSTADTSKVSLGLDENVSKR
IEPSVSLWQFYLSKMISMDIEQVITLSLALLLAVKYIFFEQTETESTLSLKNPITSPVVT
QKKVPDNCCRREPMLVRNNQKCDSVEEETGINRERKVEVIKPLVAETDTPNRATFVVGNS
SLLDTSSVLVTQEPEIELPREPRPNEECLQILGNAEKGAKFLSDAEIIQLVNAKHIPAYK
LETLMETHERGVSIRRQLLSKKLSEPSSLQYLPYRDYNYSLVMGACCENVIGYMPIPVGV
AGPLCLDEKEFQVPMATTEGCLVASTNRGCRAIGLGGGASSRVLADGMTRGPVVRLPRAC
DSAEVKAWLETSEGFAVIKEAFDSTSRFARLQKLHTSIAGRNLYIRFQSRSGDAMGMNMI
SKGTEKALSKLHEYFPEMQILAVSGNYCTDKKPAAINWIEGRGKSVVCEAVIPAKVVREV
LKTTTEAMIEVNINKNLVGSAMAGSIGGYNAHAANIVTAIYIACGQDAAQNVGSSNCITL
MEASGPTNEDLYISCTMPSIEIGTVGGGTNLLPQQACLQMLGVQGACKDNPGENARQLAR
IVCGTVMAGELSLMAALAAGHLVKSHMIHNRSKINLQDLQGACTKKTA
>hsa:32
MVLLLCLSCLIFSCLTFSWLKIWGKMTDSKPITKSKSEANLIPSQEPFPASDNSGETPQR
NGEGHTLPKTPSQAEPASHKGPKDAGRRRNSLPPSHQKPPRNPLSSSDAAPSPELQANGT
GTQGLEATDTNGLSSSARPQGQQAGSPSKEDKKQANIKRQLMTNFILGSFDDYSSDEDSV
AGSSRESTRKGSRASLGALSLEAYLTTGEAETRVPTMRPSMSGLHLVKRGREHKKLDLHR
DFTVASPAEFVTRFGGDRVIEKVLIANNGIAAVKCMRSIRRWAYEMFRNERAIRFVVMVT
PEDLKANAEYIKMADHYVPVPGGPNNNNYANVELIVDIAKRIPVQAVWAGWGHASENPKL

PELLCKNGVAFLGPPSEAMWALGDKIASTVVAQTLQVPTLPWSGSGLTVEWTEDDLQQGK
RISVPEDVYDKGCVKDVDEGLEAAERIGFPLMIKASEGGGGKGIRKAESAEDFPILFRQV
QSEIPGSPIFLMKLAQHARHLEVQILADQYGNAVSLFGRDCSIQRRHQKIVEEAPATIAP
LAIFEFMEQCAIRLAKTVGYVSAGTVEYLYSQDGSFHFLELNPRLQVEHPCTEMIADVNL
PAAQLQIAMGVPLHRLKDIRLLYGESPWGVTPISFETPSNPPLARGHVIAARITSENPDE
GFKPSSGTVQELNFRSSKNVWGYFSVAATGGLHEFADSQFGHCFSWGENREEAISNMVVA
LKELSIRGDFRTTVEYLINLLETESFQNNDIDTGWLDYLIAEKVQAEKPDIMLGVVCGAL
NVADAMFRTCMTDFLHSLERGQVLPADSLLNLVDVELIYGGVKYILKVARQSLTMFVLIM
NGCHIEIDAHRLNDGGLLLSYNGNSYTTYMKEEVDSYRITIGNKTCVFEKENDPTVLRSP
SAGKLTQYTVEDGGHVEAGSSYAEMEVMKMIMTLNVQERGRVKYIKRPGAVLEAGCVVAR
LELDDPSKVHPAEPFTGELPAQQTLPILGEKLHQVFHSVLENLTNVMSGFCLPEPVFSIK
LKEWVQKLMMTLRHPSLPLLELQEIMTSVAGRIPAPVEKSVRRVMAQYASNITSVLCQFP
SQQIATILDCHAATLQRKADREVFFINTQSIVQLVQRYRSGIRGYMKTVVLDLLRRYLRV
EHHFQQAHYDKCVINLREQFKPDMSQVLDCIFSHAQVAKKNQLVIMLIDELCGPDPSLSD
ELISILNELTQLSKSEHCKVALRARQILIASHLPSYELRHNQVESIFLSAIDMYGHQFCP
ENLKKLILSETTIFDVLPTFFYHANKVVCMASLEVYVRRGYIAYELNSLQHRQLPDGTCV
VEFQFMLPSSHPNRMTVPISITNPDLLRHSTELFMDSGFSPLCQRMGAMVAFRRFEDFTR
NFDEVISCFANVPKDTPLFSEARTSLYSEDDCKSLREEPIHILNVSIQCADHLEDEALVP
ILRTFVQSKKNILVDYGLRRITFLIAQEKEFPKFFTFRARDEFAEDRIYRHLEPALAFQL
ELNRMRNFDLTAVPCANHKMHLYLGAAKVKEGVEVTDHRFFIRAIIRHSDLITKEASFEY
LQNEGERLLLEAMDELEVAFNNTSVRTDCNHIFLNFVPTVIMDPFKIEESVRYMVMRYGS
RLWKLRVLQAEVKINIRQTTTGSAVPIRLFITNESGYYLDISLYKEVTDSRSGNIMFHSF
GNKQGPQHGMLINTPYVTKDLLQAKRFQAQTLGTTYIYDFPEMFRQALFKLWGSPDKYPK
DILTYTELVLDSQGQLVEMNRLPGGNEVGMVAFKMRFKTQEYPEGRDVIVIGNDITFRIG
SFGPGEDLLYLRASEMARAEGIPKIYVAANSGARIGMAEEIKHMFHVAWVDPEDPHKGFK
YLYLTPQDYTRISSLNSVHCKHIEEGGESRYMITDIIGKDDGLGVENLRGSGMIAGESSL
AYEEIVTISLVTCRAIGIGAYLVRLGQRVIQVENSHIILTGASALNKVLGREVYTSNNQL
GGVQIMHYNGVSHITVPDDFEGVYTILEWLSYMPKDNHSPVPIITPTDPIDREIEFLPSR
APYDPRWMLAGRPHPTLKGTWQSGFFDHGSFKEIMAPWAQTVVTGRARLGGIPVGVIAVE
TRTVEVAVPADPANLDSEAKIIQQAGQVWFPDSAYKTAQAVKDFNREKLPLMIFANWRGF
SGGMKDMYDQVLKFGAYIVDGLRQYKQPILIYIPPYAELRGGSWVVIDATINPLCIEMYA
DKESRGGVLEPEGTVEIKFRKKDLIKSMRRIDPAYKKLMEQLGEPDLSDKDRKDLEGRLK
AREDLLLPIYHQVAVQFADFHDTPGRMLEKGVISDILEWKTARTFLYWRLRRLLLEDQVK
QEILQASGELSHVHIQSMLRRWFVETEGAVKAYLWDNNQVVVQWLEQHWQAGDGPRSTIR
ENITYLKHDSVLKTIRGLVEENPEVAVDCVIYLSQHISPAERAQVVHLLSTMDSPAST
>hsa:326625
MAVCGLGSRLGLGSRLGLRGCFGAARLLYPRFQSRGPQGVEDGDRPQPSSKTPRIPKIYT
KTGDKGFSSTFTGERRPKDDQVFEAVGTTDELSSAIGFALELVTEKGHTFAEELQKIQCT
LQDVGSALATPCSSAREAHLKYTTFKAGPILELEQWIDKYTSQLPPLTAFILPSGGKISS
ALHFCRAVCRRAERRVVPLVQMGETDANVAKFLNRLSDYLFTLARYAAMKEGNQEKIYMK
NDPSAESEGL
>hsa:327
MERQVLLSEPEEAAALYRGLSRQPALSAACLGPEVTTQYGGQYRTVHTEWTQRDLERMEN
IRFCRQYLVFHDGDSVVFAGPAGNSVETRGELLSRESPSGTMKAVLRKAGGTGPGEEKQF

LEVWEKNRKLKSFNLSALEKHGPVYEDDCFGCLSWSHSETHLLYVAEKKRPKAESFFQTK
ALDVSASDDEIARLKKPDQAIKGDQFVFYEDWGENMVSKSIPVLCVLDVESGNISVLEGV
PENVSPGQAFWAPGDAGVVFVGWWHEPFRLGIRFCTNRRSALYYVDLIGGKCELLSDDSL
AVSSPRLSPDQCRIVYLQYPSLIPHHQCSQLCLYDWYTKVTSVVVDVVPRQLGENFSGIY
CSLLPLGCWSADSQRVVFDSAQRSRQDLFAVDTQVGTVTSLTAGGSGGSWKLLTIDQDLM
VAQFSTPSLPPTLKVGFLPSAGKEQSVLWVSLEEAEPIPDIHWGIRVLQPPPEQENVQYA
GLDFEAILLQPGSPPDKTQVPMVVMPHGGPHSSFVTAWMLFPAMLCKMGFAVLLVNYRGS
TGFGQDSILSLPGNVGHQDVKDVQFAVEQVLQEEHFDASHVALMGGSHGGFISCHLIGQY
PETYRACVARNPVINIASMLGSTDIPDWCVVEAGFPFSSDCLPDLSVWAEMLDKSPIRYI
PQVKTPLLLMLGQEDRRVPFKQGMEYYRALKTRNVPVRLLLYPKSTHALSEVEVESDSFM
NAVLWLRTHLGS
>hsa:3283
MTGWSCLVTGAGGFLGQRIIRLLVKEKELKEIRVLDKAFGPELREEFSKLQNKTKLTVLE
GDILDEPFLKRACQDVSVIIHTACIIDVFGVTHRESIMNVNVKGTQLLLEACVQASVPVF
IYTSSIEVAGPNSYKEIIQNGHEEEPLENTWPAPYPHSKKLAEKAVLAANGWNLKNGGTL
YTCALRPMYIYGEGSRFLSASINEALNNNGILSSVGKFSTVNPVYVGNVAWAHILALRAL
QDPKKAPSIRGQFYYISDDTPHQSYDNLNYTLSKEFGLRLDSRWSFPLSLMYWIGFLLEI
VSFLLRPIYTYRPPFNRHIVTLSNSVFTFSYKKAQRDLAYKPLYSWEEAKQKTVEWVGSL
VDRHKETLKSKTQ
>hsa:3290
MAFMKKYLLPILGLFMAYYYYSANEEFRPEMLQGKKVIVTGASKGIGREMAYHLAKMGAH
VVVTARSKETLQKVVSHCLELGAASAHYIAGTMEDMTFAEQFVAQAGKLMGGLDMLILNH
ITNTSLNLFHDDIHHVRKSMEVNFLSYVVLTVAALPMLKQSNGSIVVVSSLAGKVAYPMV
AAYSASKFALDGFFSSIRKEYSVSRVNVSITLCVLGLIDTETAMKAVSGIVHMQAAPKEE
CALEIIKGGALRQEEVYYDSSLWTTLLIRNPCRKILEFLYSTSYNMDRFINK
>hsa:3291
MERWPWPSGGAWLLVAARALLQLLRSDLRLGRPLLAALALLAALDWLCQRLLPPPAALAV
LAAAGWIALSRLARPQRLPVATRAVLITGCDSGFGKETAKKLDSMGFTVLATVLELNSPG
AIELRTCCSPRLRLLQMDLTKPGDISRVLEFTKAHTTSTGLWGLVNNAGHNEVVADAELS
PVATFRSCMEVNFFGALELTKGLLPLLRSSRGRIVTVGSPAGDMPYPCLGAYGTSKAAVA
LLMDTFSCELLPWGVKVSIIQPGCFKTESVRNVGQWEKRKQLLLANLPQELLQAYGKDYI
EHLHGQFLHSLRLAMSDLTPVVDAITDALLAARPRRRYYPGQGLGLMYFIHYYLPEGLRR
RFLQAFFISHCLPRALQPGQPGTTPPQDAAQDPNLSPGPSPAVAR
>hsa:3292
ETLQLDVRDSKSVAAARERVTEGRVDVLVCNAGLGLLGPLEALGEDAVASVLDVNVVGTV
RMLQAFLPDMKRRGSGRVLVTGSVGGLMGLPFNDVYCASKFALEGLCESLAVLLLPFGVH
LSLIECGPVHTAFMEKVLGSPEEVLDRTDIHTFHRFYQYLAHSKQVFREAAQNPEEVAEV
FLTALRAPKPTLRYFTTERFLPLLRMRLDDPSGSNYVTAMHREVFGDVPAKAEAGAEAGG
GAGPGAEDEAGRGAVGDPELGDPPAAPQ
>hsa:3295
MGSPLRFDGRVVLVTGAGAGLGRAYALAFAERGALVVVNDLGGDFKGVGKGSLAADKVVE
EIRRRGGKAVANYDSVEEGEKVVKTALDAFGRIDVVVNNAGILRDRSFARISDEDWDIIH
RVHLRGSFQVTRAAWEHMKKQKYGRIIMTSSASGIYGNFGQANYSAAKLGLLGLANSLAI
EGRKSNIHCNTIAPNAGSRMTQTVMPEDLVEALKPEYVAPLVLWLCHESCEENGGLFEVG

AGWIGKLRWERTLGAIVRQKNHPMTPEAVKANWKKICDFENASKPQSIQESTGSIIEVLS
KIDSEGGVSANHTSRATSTATSGFAGAIGQKLPPFSYAYTELEAIMYALGVGASIKDPKD
LKFIYEGSSDFSCLPTFGVIIGQKSMMGGGLAEIPGLSINFAKVLHGEQYLELYKPLPRA
GKLKCEAVVADVLDKGSGVVIIMDVYSYSEKELICHNQFSLFLVGSGGFGGKRTSDKVKV
AVAIPNRPPDAVLTDTTSLNQAALYRLSGDWNPLHIDPNFASLAGFDKPILHGLCTFGFS
ARRVLQQFADNDVSRFKAIKARFAKPVYPGQTLQTEMWKEGNRIHFQTKVQETGDIVISN
AYVDLAPTSGTSAKTPSEGGKLQSTFVFEEIGRRLKDIGPEVVKKVNAVFEWHITKGGNI
GAKWTIDLKSGSGKVYQGPAKGAADTTIILSDEDFMEVVLGKLDPQKAFFSGRLKARGNI
MLSQKLQMILKDYAKL
>hsa:3376
MLQQVPENINFPAEEEKILEFWTEFNCFQECLKQSKHKPKFTFYDGPPFATGLPHYGHIL
AGTIKDIVTRYAHQSGFHVDRRFGWDCHGLPVEYEIDKTLGIRGPEDVAKMGITEYNNQC
RAIVMRYSAEWKSTVSRLGRWIDFDNDYKTLYPQFMESVWWVFKQLYDKGLVYRGVKVMP
FSTACNTPLSNFESHQNYKDVQDPSVFVTFPLEEDETVSLVAWTTTPWTLPSNLAVCVNP
EMQYVKIKDVARGRLLILMEARLSALYKLESDYEILERFPGAYLKGKKYRPLFDYFLKCK
ENGAFTVLVDNYVKEEEGTGVVHQAPYFGAEDYRVCMDFNIIRKDSLPVCPVDASGCFTT
EVTDFAGQYVKDADKSIIRTLKEQGRLLVATTFTHSYPFCWRSDTPLIYKAVPSWFVRVE
NMVDQLLRNNDLCYWVPELVREKRFGNWLKDARDWTISRNRYWGTPIPLWVSDDFEEVVC
IGSVAELEELSGAKISDLHRESVDHLTIPSRCGKGSLHRISEVFDCWFESGSMPYAQVHY
PFENKREFEDAFPADFIAEGIDQTRGWFYTLLVLATALFGQPPFKNVIVNGLVLASDGQK
MSKRKKNYPDPVSIIQKYGADALRLYLINSPVVRAENLRFKEEGVRDVLKDVLLPWYNAY
RFLIQNVLRLQKEEEIEFLYNENTVRESPNITDRWILSFMQSLIGFFETEMAAYRLYTVV
PRLVKFVDILTNWYVRMNRRRLKGENGMEDCVMALETLFSVLLSLCRLMAPYTPFLTELM
YQNLKVLIDPVSVQDKDTLSIHYLMLPRVREELIDKKTESAVSQMQSVIELGRVIRDRKT
IPIKYPLKEIVVIHQDPEALKDIKSLEKYIIEELNVRKVTLSTDKNKYGIRLRAEPDHMV
LGKRLKGAFKAVMTSIKQLSSEELEQFQKTGTIVVEGHELHDEDIRLMYTFDQATGGTAQ
FEAHSDAQALVLLDVTPDQSMVDEGMAREVINRIQKLRKKCNLVPTDEITVYYKAKSEGT
YLNSVIESHTEFIFTTIKAPLKPYPVSPSDKVLIQEKTQLKGSELEITLTRGSSLPGPAC
AYVNLNICANGSEQGGVLLLENPKGDNRLDLLKLKSVVTSIFGVKNTELAVFHDETEIQN
QTDLLSLSGKTLCVTAGSAPSLINSSSTLLCQYINLQLLNAKPQECLMGTVGTLLLENPL
GQNGLTHQGLLYEAAKVFGLRSRKLKLFLNETQTQEITEDIPVKTLNMKTVYVSVLPTTA
DF
>hsa:339221
MRGLAVLLTVALATLLAPGAGAPVQSQGSQNKLLLVSFDGFRWNYDQDVDTPNLDAMARD
GVKARYMTPAFVTMTSPCHFTLVTGKYIENHGVVHNMYYNTTSKVKLPYHATLGIQRWWD
NGSVPIWITAQRQGLRAGSFFYPGGNVTYQGVAVTRSRKEGIAHNYKNETEWRANIDTVM
AWFTEEDLDLVTLYFGEPDSTGHRYGPESPERREMVRQVDRTVGYLRESIARNHLTDRLN
LIITSDHGMTTVDKRAGDLVEFHKFPNFTFRDIEFELLDYGPNGMLLPKEGRLEKVYDAL
KDAHPKLHVYKKEAFPEAFHYANNPRVTPLLMYSDLGYVIHGRINVQFNNGEHGFDNKDM
DMKTIFRAVGPSFRAGLEVEPFESVHVYELMCRLLGIVPEANDGHLATLLPMLHTESALP
PDGRPTLLPKGRSALPPSSRPLLVMGLLGTVILLSEVA
>hsa:34
MAAGFGRCCRVLRSISRFHWRSQHTKANRQREPGLGFSFEFTEQQKEFQATARKFAREEI
IPVAAEYDKTGEYPVPLIRRAWELGLMNTHIPENCGGLGLGTFDACLISEELAYGCTGVQ

TAIEGNSLGQMPIIIAGNDQQKKKYLGRMTEEPLMCAYCVTEPGAGSDVAGIKTKAEKKG
DEYIINGQKMWITNGGKANWYFLLARSDPDPKAPANKAFTGFIVEADTPGIQIGRKELNM
GQRCSDTRGIVFEDVKVPKENVLIGDGAGFKVAMGAFDKTRPVVAAGAVGLAQRALDEAT
KYALERKTFGKLLVEHQAISFMLAEMAMKVELARMSYQRAAWEVDSGRRNTYYASIAKAF
AGDIANQLATDAVQILGGNGFNTEYPVEKLMRDAKIYQIYEGTSQIQRLIVAREHIDKYK
N

>hsa:3416

MSKLWFKQDDKFFLPKACLNFEFFSPFAYVDPLHCNMAYLYLELLKDSLNEYAYAAELAG
LSYDLQNTIYGMYLSVKGYNDKQPILLKKIIEKMATFEIDEKRFEIIKEAYMRSLNNFRA
EQPHQHAMYYLRLLMTEVAWTKDELKEALDDVTLPRLKAFIPQLLSRLHIEALLHGNITK
QAALGIMQMVEDTLIEHAHTKPLLPSQLVRYREVQLPDRGWFVYQQRNEVHNNCGIEIYY
QTDMQSTSENMFLELFCQIISEPCFNTLRTKEQLGYIVFSGPRRANGIQGLRFIIQSEKP
PHYLESRVEAFLITMEKSIEDMTEEAFQKHIQALAIRRLDKPKKLSAECAKYWGEIISQQ
YNFDRDNTEVAYLKTLTKEDIIKFYKEMLAVDAPRRHKVSVHVLAREMDSCPVVGEFPCQ
NDINLSQAPALPQPEVIQNMTEFKRGLPLFPLVKPHINFMAAKL

>hsa:3480

MKSGSGGGSPTSLWGLLFLSAALSLWPTSGEICGPGIDIRNDYQQLKRLENCTVIEGYLH
ILLISKAEDYRSYRFPKLTVITEYLLLFRVAGLESLGDLFPNLTVIRGWKLFYNYALVIF
EMTNLKDIGLYNLRNITRGAIRIEKNADLCYLSTVDWSLILDAVSNNYIVGNKPPKECGD
LCPGTMEEKPMCEKTTINNEYNYRCWTTNRCQKMCPSTCGKRACTENNECCHPECLGSCS
APDNDTACVACRHYYYAGVCVPACPPNTYRFEGWRCVDRDFCANILSAESSDSEGFVIHD
GECMQECPSGFIRNGSQSMYCIPCEGPCPKVCEEEKKTKTIDSVTSAQMLQGCTIFKGNL
LINIRRGNNIASELENFMGLIEVVTGYVKIRHSHALVSLSFLKNLRLILGEEQLEGNYSF
YVLDNQNLQQLWDWDHRNLTIKAGKMYFAFNPKLCVSEIYRMEEVTGTKGRQSKGDINTR
NNGERASCESDVLHFTSTTTSKNRIIITWHRYRPPDYRDLISFTVYYKEAPFKNVTEYDG
QDACGSNSWNMVDVDLPPNKDVEPGILLHGLKPWTQYAVYVKAVTLTMVENDHIRGAKSE
ILYIRTNASVPSIPLDVLSASNSSSQLIVKWNPPSLPNGNLSYYIVRWQRQPQDGYLYRH
NYCSKDKIPIRKYADGTIDIEEVTENPKTEVCGGEKGPCCACPKTEAEKQAEKEEAEYRK
VFENFLHNSIFVPRPERKRRDVMQVANTTMSSRSRNTTAADTYNITDPEELETEYPFFES
RVDNKERTVISNLRPFTLYRIDIHSCNHEAEKLGCSASNFVFARTMPAEGADDIPGPVTW
EPRPENSIFLKWPEPENPNGLILMYEIKYGSQVEDQRECVSRQEYRKYGGAKLNRLNPGN
YTARIQATSLSGNGSWTDPVFFYVQAKTGYENFIHLIIALPVAVLLIVGGLVIMLYVFHR
KRNNSRLGNGVLYASVNPEYFSAADVYVPDEWEVAREKITMSRELGQGSFGMVYEGVAKG
VVKDEPETRVAIKTVNEAASMRERIEFLNEASVMKEFNCHHVVRLLGVVSQGQPTLVIME
LMTRGDLKSYLRSLRPEMENNPVLAPPSLSKMIQMAGEIADGMAYLNANKFVHRDLAARN
CMVAEDFTVKIGDFGMTRDIYETDYYRKGGKGLLPVRWMSPESLKDGVFTTYSDVWSFGV
VLWEIATLAEQPYQGLSNEQVLRFVMEGGLLDKPDNCPDMLFELMRMCWQYNPKMRPSFL
EIISSIKEEMEPGFREVSFYYSEENKLPEPEELDLEPENMESVPLDPSASSSSLPLPDRH
SGHKAENGPGPGVLVLRASFDERQPYAHMNGGRKNERALPLPQSSTC

>hsa:349565

MKSRIPVVLLACGSFNPITNMHLRMFEVARDHLHQTAVPELKLLCGADVLKTFQTPNLWK
DAHIQEIVEKFGLVCVGRVGHDPKGYIAESPILRMHQHNIHLAKEPVQNEISATYIRRAL
GQGQSVKYLIPDAVITYIKDHGLYTKGSTWKGKSTQSTEGKTS

>hsa:35

MAAALLARASGPARRALCPRAWRQLHTIYQSVELPETHQMLLQTCRDFAEKELFPIAAQV
DKEHLFPAAQVKKMGGLGLLAMDVPEELGGAGLDYLAYAIAMEEISRGCASTGVIMSVNN
SLYLGPILKFGSKEQKQAWVTPFTSGDKIGCFALSEPGNGSDAGAASTTARAEGDSWVLN
GTKAWITNAWEASAAVVFASTDRALQNKGISAFLVPMPTPGLTLGKKEDKLGIRGSSTAN
LIFEDCRIPKDSILGEPGMGFKIAMQTLDMGRIGIASQALGIAQTALDCAVNYAENRMAF
GAPLTKLQVIQFKLADMALALESARLLTWRAAMLKDNKKPFIKEAAMAKLAASEAATAIS
HQAIQILGGMGYVTEMPAERHYRDARITEIYEGTSEIQRLVIAGHLLRSYRS
>hsa:353
MADSELQLVEQRIRSFPDFPTPGVVFRDISPVLKDPASFRAAIGLLARHLKATHGGRIDY
IAGLDSRGFLFGPSLAQELGLGCVLIRKRGKLPGPTLWASYSLEYGKAELEIQKDALEPG
QRVVVVDDLLATGGTMNAACELLGRLQAEVLECVSLVELTSLKGREKLAPVPFFSLLQYE
>hsa:354
MWVPVVFLTLSVTWIGAAPLILSRIVGGWECEKHSQPWQVLVASRGRAVCGGVLVHPQWV
LTAAHCIRNKSVILLGRHSLFHPEDTGQVFQVSHSFPHPLYDMSLLKNRFLRPGDDSSHD
LMLLRLSEPAELTDAVKVMDLPTQEPALGTTCYASGWGSIEPEEFLTPKKLQCVDLHVIS
NDVCAQVHPQKVTKFMLCAGRWTGGKSTCSWVILITELTMPALPMVLHGSLVPWRGGV
>hsa:3551
MFSGGCHSPGFGRPSPAFPAPGSPPPAPRPCRQETGEQIAIKQCRQELSPRNRERWCLEI
QIMRRLTHPNVVAARDVPEGMQNLAPNDLPLLAMEYCQGGDLRKYLNQFENCCGLREGAI
LTLLSDIASALRYLHENRIIHRDLKPENIVLQQGEQRLIHKIIDLGYAKELDQGSLCTSF
VGTLQYLAPELLEQQKYTVTVDYWSFGTLAFECITGFRPFLPNWQPVQWHSKVRQKSEVD
IVVSEDLNGTVKFSSSLPYPNNLNSVLAERLEKWLQLMLMWHPRQRGTDPTYGPNGCFKA
LDDILNLKLVHILNMVTGTIHTYPVTEDESLQSLKARIQQDTGIPEEDQELLQEAGLALI
PDKPATQCISDGKLNEGHTLDMDLVFLFDNSKITYETQISPRPQPESVSCILQEPKRNLA
FFQLRKVWGQVWHSIQTLKEDCNRLQQGQRAAMMNLLRNNSCLSKMKNSMASMSQQLKAK
LDFFKTSIQIDLEKYSEQTEFGITSDKLLLAWREMEQAVELCGRENEVKLLVERMMALQT
DIVDLQRSPMGRKQGGTLDDLEEQARELYRRLREKPRDQRTEGDSQEMVRLLLQAIQSFE
KKVRVIYTQLSKTVVCKQKALELLPKVEEVVSLMNEDEKTVVRLQEKRQKELWNLLKIAC
SKVRGPVSGSPDSMNASRLSQPGQLMSQPSTASNSLPEPAKKSEELVAEAHNLCTLLENA
IQDTVREQDQSFTALDWSWLQTEEEEHSCLEQAS
>hsa:36
MEGLAVRLLRGSRLLRRNFLTCLSSWKIPPHVSKSSQSEALLNITNNGIHFAPLQTFTDE
EMMIKSSVKKFAQEQIAPLVSTMDENSKMEKSVIQGLFQQGLMGIEVDPEYGGTGASFLS
TVLVIEELAKVDASVAVFCEIQNTLINTLIRKHGTEEQKATYLPQLTTEKVGSFCLSEAG
AGSDSFALKTRADKEGDYYVLNGSKMWISSAEHAGLFLVMANVDPTIGYKGITSFLVDRD
TPGLHIGKPENKLGLRASSTCPLTFENVKVPEANILGQIGHGYKYAIGSLNEGRIGIAAQ
MLGLAQGCFDYTIPYIKERIQFGKRLFDFQGLQHQVAHVATQLEAARLLTYNAARLLEAG
KPFIKEASMAKYYASEIAGQTTSKCIEWMGGVGYTKDYPVEKYFRDAKIGTIYEGASNIQ
LNTIAKHIDAEY
>hsa:3612
MGQRPGPVLPAVAVLGQVAKRKVAWLLRWKAVTRTETAGNSSGVYGFGKMKIFVKYFQKM
ADPWQECMDYAVTLARQAGEVVCEAIKNEMNVMLKSSPVDLVTATDQKVEKMLISSIKEK
YPSHSFIGEESVAAGEKSILTDNPTWIIDPIDGTTNFVHRFPFVAVSIGFAVNKKIEFGV
VYSCVEGKMYTARKGKGAFCNGQKLQVSQQEDITKSLLVTELGSSRTPETVRMVLSNMEK

LFCIPVHGIRSVGTAAVNMCLVATGGADAYYEMGIHCWDVAGAGIIVTEAGGVLMDVTGG
PFDLMSRRVIAANNRILAERIAKEIQVIPLQRDDED
>hsa:3614
MEGPLTPPPLQGGGAAAVPEPGARQHPGHETAAQRYSARLLQAGYEPESPRLDLATHPTT
PRSELSSVVLLAGVGVQMDRLRRASMADYLISGGTGYVPEDGLTAQQLFASADGLTYNDF
LILPGFIDFIADEVDLTSALTRKITLKTPLISSPMDTVTEADMAIAMALMGGIGFIHHNC
TPEFQANEVRKVKKFEQGFITDPVVLSPSHTVGDVLEAKMRHGFSGIPITETGTMGSKLV
GIVTSRDIDFLAEKDHTTLLSEVMTPRIELVVAPAGVTLKEANEILQRSKKGKLPIVNDC
DELVAIIARTDLKKNRDYPLASKDSQKQLLCGAAVGTREDDKYRLDLLTQAGVDVIVLDS
SQGNSVYQIAMVHYIKQKYPHLQVIGGNVVTAAQAKNLIDAGVDGLRVGMGCGSICITQE
VMACGRPQGTAVYKVAEYARRFGVPIIADGGIQTVGHVVKALALGASTVMMGSLLAATTE
APGEYFFSDGVRLKKYRGMGSLDAMEKSSSSQKRYFSEGDKVKIAQGVSGSIQDKGSIQK
FVPYLIAGIQHGCQDIGARSLSVLRSMMYSGELKFEKRTMSAQIEGGVHGLHSYEKRLY
>hsa:3615
MADYLISGGTSYVPDDGLTAQQLFNCGDGLTYNDFLILPGYIDFTADQVDLTSALTKKIT
LKTPLVSSPMDTVTEAGMAIAMALTGGIGFIHHNCTPEFQANEVRKVKKYEQGFITDPVV
LSPKDRVRDVFEAKARHGFCGIPITDTGRMGSRLVGIISSRDIDFLKEEEHDCFLEEIMT
KREDLVVAPAGITLKEANEILQRSKKGKLPIVNEDDELVAIIARTDLKKNRDYPLASKDA
KKQLLCGAAIGTHEDDKYRLDLLAQAGVDVVVLDSSQGNSIFQINMIKYIKDKYPNLQVI
GGNVVTAAQAKNLIDAGVDALRVGMGSGSICITQEVLACGRPQATAVYKVSEYARRFGVP
VIADGGIQNVGHIAKALALGASTVMMGSLLAATTEAPGEYFFSDGIRLKKYRGMGSLDAM
DKHLSSQNRYFSEADKIKVAQGVSGAVQDKGSIHKFVPYLIAGIQHSCQDIGAKSLTQVR
AMMYSGELKFEKRTSSAQVEGGVHSLHSYEKRLF
>hsa:3643
MATGGRRGAAAAPLLVAVAALLLGAAGHLYPGEVCPGMDIRNNLTRLHELENCSVIEGHL
QILLMFKTRPEDFRDLSFPKLIMITDYLLLFRVYGLESLKDLFPNLTVIRGSRLFFNYAL
VIFEMVHLKELGLYNLMNITRGSVRIEKNNELCYLATIDWSRILDSVEDNYIVLNKDDNE
ECGDICPGTAKGKTNCPATVINGQFVERCWTHSHCQKVCPTICKSHGCTAEGLCCHSECL
GNCSQPDDPTKCVACRNFYLDGRCVETCPPPYYHFQDWRCVNFSFCQDLHHKCKNSRRQG
CHQYVIHNNKCIPECPSGYTMNSSNLLCTPCLGPCPKVCHLLEGEKTIDSVTSAQELRGC
TVINGSLIINIRGGNNLAAELEANLGLIEEISGYLKIRRSYALVSLSFFRKLRLIRGETL
EIGNYSFYALDNQNLRQLWDWSKHNLTITQGKLFFHYNPKLCLSEIHKMEEVSGTKGRQE
RNDIALKTNGDQASCENELLKFSYIRTSFDKILLRWEPYWPPDFRDLLGFMLFYKEAPYQ
NVTEFDGQDACGSNSWTVVDIDPPLRSNDPKSQNHPGWLMRGLKPWTQYAIFVKTLVTFS
DERRTYGAKSDIIYVQTDATNPSVPLDPISVSNSSSQIILKWKPPSDPNGNITHYLVFWE
RQAEDSELFELDYCLKGLKLPSRTWSPPFESEDSQKHNQSEYEDSAGECCSCPKTDSQIL
KELEESSFRKTFEDYLHNVVFVPRKTSSGTGAEDPRPSRKRRSLGDVGNVTVAVPTVAAF
PNTSSTSVPTSPEEHRPFEKVVNKESLVISGLRHFTGYRIELQACNQDTPEERCSVAAYV
SARTMPEAKADDIVGPVTHEIFENNVVHLMWQEPKEPNGLIVLYEVSYRRYGDEELHLCV
SRKHFALERGCRLRGLSPGNYSVRIRATSLAGNGSWTEPTYFYVTDYLDVPSNIAKIIIG
PLIFVFLFSVVIGSIYLFLRKRQPDGPLGPLYASSNPEYLSASDVFPCSVYVPDEWEVSR
EKITLLRELGQGSFGMVYEGNARDIIKGEAETRVAVKTVNESASLRERIEFLNEASVMKG
FTCHHVVRLLGVVSKGQPTLVVMELMAHGDLKSYLRSLRPEAENNPGRPPPTLQEMIQMA
AEIADGMAYLNAKKFVHRDLAARNCMVAHDFTVKIGDFGMTRDIYETDYYRKGGKGLLPV

RWMAPESLKDGVFTTSSDMWSFGVVLWEITSLAEQPYQGLSNEQVLKFVMDGGYLDQPDN
CPERVTDLMRMCWQFNPKMRPTFLEIVNLLKDDLHPSFPEVSFFHSEENKAPESEELEME
FEDMENVPLDRSSHCQREEAGGRDGGSSLGFKRSYEEHIPYTHMNGGKKNGRILTLPRSN
PS
>hsa:3645
MAVPSLWPWGACLPVIFLSLGFGLDTVEVCPSLDIRSEVAELRQLENCSVVEGHLQILLM
FTATGEDFRGLSFPRLTQVTDYLLLFRVYGLESLRDLFPNLAVIRGTRLFLGYALVIFEM
PHLRDVALPALGAVLRGAVRVEKNQELCHLSTIDWGLLQPAPGANHIVGNKLGEECADVC
PGVLGAAGEPCAKTTFSGHTDYRCWTSSHCQRVCPCPHGMACTARGECCHTECLGGCSQP
EDPRACVACRHLYFQGACLWACPPGTYQYESWRCVTAERCASLHSVPGRASTFGIHQGSC
LAQCPSGFTRNSSSIFCHKCEGLCPKECKVGTKTIDSIQAAQDLVGCTHVEGSLILNLRQ
GYNLEPQLQHSLGLVETITGFLKIKHSFALVSLGFFKNLKLIRGDAMVDGNYTLYVLDNQ
NLQQLGSWVAAGLTIPVGKIYFAFNPRLCLEHIYRLEEVTGTRGRQNKAEINPRTNGDRA
ACQTRTLRFVSNVTEADRILLRWERYEPLEARDLLSFIVYYKESPFQNATEHVGPDACGT
QSWNLLDVELPLSRTQEPGVTLASLKPWTQYAVFVRAITLTTEEDSPHQGAQSPIVYLRT
LPAAPTVPQDVISTSNSSSHLLVRWKPPTQRNGNLTYYLVLWQRLAEDGDLYLNDYCHRG
LRLPTSNNDPRFDGEDGDPEAEMESDCCPCQHPPPGQVLPPLEAQEASFQKKFENFLHNA
ITIPISPWKVTSINKSPQRDSGRHRRAAGPLRLGGNSSDFEIQEDKVPRERAVLSGLRHF
TEYRIDIHACNHAAHTVGCSAATFVFARTMPHREADGIPGKVAWEASSKNSVLLRWLEPP
DPNGLILKYEIKYRRLGEEATVLCVSRLRYAKFGGVHLALLPPGNYSARVRATSLAGNGS
WTDSVAFYILGPEEEDAGGLHVLLTATPVGLTLLIVLAALGFFYGKKRNRTLYASVNPEY
FSASDMYVPDEWEVPREQISIIRELGQGSFGMVYEGLARGLEAGEESTPVALKTVNELAS
PRECIEFLKEASVMKAFKCHHVVRLLGVVSQGQPTLVIMELMTRGDLKSHLRSLRPEAEN
NPGLPQPALGEMIQMAGEIADGMAYLAANKFVHRDLAARNCMVSQDFTVKIGDFGMTRDV
YETDYYRKGGKGLLPVRWMAPESLKDGIFTTHSDVWSFGVVLWEIVTLAEQPYQGLSNEQ
VLKFVMDGGVLEELEGCPLQLQELMSRCWQPNPRLRPSFTHILDSIQEELRPSFRLLSFY
YSPECRGARGSLPTTDAEPDSSPTPRDCSPQNGGPGH
>hsa:3702
MNNFILLEEQLIKKSQQKRRTSPSNFKVRFFVLTKASLAYFEDRHGKKRTLKGSIELSRI
KCVEIVKSDISIPCHYKYPFQVVHDNYLLYVFAPDRESRQRWVLALKEETRNNNSLVPKY
HPNFWMDGKWRCCSQLEKLATGCAQYDPTKNASKKPLPPTPEDNRRPLWEPEETVVIALY
DYQTNDPQELALRRNEEYCLLDSSEIHWWRVQDRNGHEGYVPSSYLVEKSPNNLETYEWY
NKSISRDKAEKLLLDTGKEGAFMVRDSRTAGTYTVSVFTKAVVSENNPCIKHYHIKETND
NPKRYYVAEKYVFDSIPLLINYHQHNGGGLVTRLRYPVCFGRQKAPVTAGLRYGKWVIDP
SELTFVQEIGSGQFGLVHLGYWLNKDKVAIKTIREGAMSEEDFIEEAEVMMKLSHPKLVQ
LYGVCLEQAPICLVFEFMEHGCLSDYLRTQRGLFAAETLLGMCLDVCEGMAYLEEACVIH
RDLAARNCLVGENQVIKVSDFGMTRFVLDDQYTSSTGTKFPVKWASPEVFSFSRYSSKSD
VWSFGVLMWEVFSEGKIPYENRSNSEVVEDISTGFRLYKPRLASTHVYQIMNHCWKERPE
DRPAFSRLLRQLAEIAESGL
>hsa:3712
MAEMATATRLLGWRVASWRLRPPLAGFVSQRAHSLLPVDDAINGLSEEQRQEFWKQLGNL
GVLGITAPVQYGGSGLGYLEHVLVMEEISRASGAVGLSYGAHSNLCINQLVRNGNEAQKE
KYLPKLISGEYIGALAMSEPNAGSDVVSMKLKAEKKGNHYILNGNKFWITNGPDADVLIV
YAKTDLAAVPASRGITAFIVEKGMPGFSTSKKLDKLGMRGSNTCELIFEDCKIPAANILG

HENKGVYVLMSGLDLERLVLAGGPLGLMQAVLDHTIPYLHVREAFGQKIGHFQLMQGKMA
DMYTRLMACRQYVYNVAKACDEGHCTAKDCAGVILYSAECATQVALDGIQCFGGNGYIND
FPMGRFLRDAKLYEIGAGTSEVRRLVIGRAFNADFH
>hsa:3716
MQYLNIKEDCNAMAFCAKMRSSKKTEVNLEAPEPGVEVIFYLSDREPLRLGSGEYTAEEL
CIRAAQACRISPLCHNLFALYDENTKLWYAPNRTITVDDKMSLRLHYRMRFYFTNWHGTN
DNEQSVWRHSPKKQKNGYEKKKIPDATPLLDASSLEYLFAQGQYDLVKCLAPIRDPKTEQ
DGHDIENECLGMAVLAISHYAMMKKMQLPELPKDISYKRYIPETLNKSIRQRNLLTRMRI
NNVFKDFLKEFNNKTICDSSVSTHDLKVKYLATLETLTKHYGAEIFETSMLLISSENEMN
WFHSNDGGNVLYYEVMVTGNLGIQWRHKPNVVSVEKEKNKLKRKKLENKHKKDEEKNKIR
EEWNNFSYFPEITHIVIKESVVSINKQDNKKMELKLSSHEEALSFVSLVDGYFRLTADAH
HYLCTDVAPPLIVHNIQNGCHGPICTEYAINKLRQEGSEEGMYVLRWSCTDFDNILMTVT
CFEKSEQVQGAQKQFKNFQIEVQKGRYSLHGSDRSFPSLGDLMSHLKKQILRTDNISFML
KRCCQPKPREISNLLVATKKAQEWQPVYPMSQLSFDRILKKDLVQGEHLGRGTRTHIYSG
TLMDYKDDEGTSEEKKIKVILKVLDPSHRDISLAFFEAASMMRQVSHKHIVYLYGVCVRD
VENIMVEEFVEGGPLDLFMHRKSDVLTTPWKFKVAKQLASALSYLEDKDLVHGNVCTKNL
LLAREGIDSECGPFIKLSDPGIPITVLSRQECIERIPWIAPECVEDSKNLSVAADKWSFG
TTLWEICYNGEIPLKDKTLIEKERFYESRCRPVTPSCKELADLMTRCMNYDPNQRPFFRA
IMRDINKLEEQNPDIVSEKKPATEVDPTHFEKRFLKRIRDLGEGHFGKVELCRYDPEGDN
TGEQVAVKSLKPESGGNHIADLKKEIEILRNLYHENIVKYKGICTEDGGNGIKLIMEFLP
SGSLKEYLPKNKNKINLKQQLKYAVQICKGMDYLGSRQYVHRDLAARNVLVESEHQVKIG
DFGLTKAIETDKEYYTVKDDRDSPVFWYAPECLMQSKFYIASDVWSFGVTLHELLTYCDS
DSSPMALFLKMIGPTHGQMTVTRLVNTLKEGKRLPCPPNCPDEVYQLMRKCWEFQPSNRT
SFQNLIEGFEALLK
>hsa:3717
MGMACLTMTEMEGTSTSSIYQNGDISGNANSMKQIDPVLQVYLYHSLGKSEADYLTFPSG
EYVAEEICIAASKACGITPVYHNMFALMSETERIWYPPNHVFHIDESTRHNVLYRIRFYF
PRWYCSGSNRAYRHGISRGAEAPLLDDFVMSYLFAQWRHDFVHGWIKVPVTHETQEECLG
MAVLDMMRIAKENDQTPLAIYNSISYKTFLPKCIRAKIQDYHILTRKRIRYRFRRFIQQF
SQCKATARNLKLKYLINLETLQSAFYTEKFEVKEPGSGPSGEEIFATIIITGNGGIQWSR
GKHKESETLTEQDLQLYCDFPNIIDVSIKQANQEGSNESRVVTIHKQDGKNLEIELSSLR
EALSFVSLIDGYYRLTADAHHYLCKEVAPPAVLENIQSNCHGPISMDFAISKLKKAGNQT
GLYVLRCSPKDFNKYFLTFAVERENVIEYKHCLITKNENEEYNLSGTKKNFSSLKDLLNC
YQMETVRSDNIIFQFTKCCPPKPKDKSNLLVFRTNGVSDVPTSPTLQRPTHMNQMVFHKI
RNEDLIFNESLGQGTFTKIFKGVRREVGDYGQLHETEVLLKVLDKAHRNYSESFFEAASM
MSKLSHKHLVLNYGVCVCGDENILVQEFVKFGSLDTYLKKNKNCINILWKLEVAKQLAWA
MHFLEENTLIHGNVCAKNILLIREEDRKTGNPPFIKLSDPGISITVLPKDILQERIPWVP
PECIENPKNLNLATDKWSFGTTLWEICSGGDKPLSALDSQRKLQFYEDRHQLPAPKWAEL
ANLINNCMDYEPDFRPSFRAIIRDLNSLFTPDYELLTENDMLPNMRIGALGFSGAFEDRD
PTQFEERHLKFLQQLGKGNFGSVEMCRYDPLQDNTGEVVAVKKLQHSTEEHLRDFEREIE
ILKSLQHDNIVKYKGVCYSAGRRNLKLIMEYLPYGSLRDYLQKHKERIDHIKLLQYTSQI
CKGMEYLGTKRYIHRDLATRNILVENENRVKIGDFGLTKVLPQDKEYYKVKEPGESPIFW
YAPESLTESKFSVASDVWSFGVVLYELFTYIEKSKSPPAEFMRMIGNDKQGQMIVFHLIE
LLKNNGRLPRPDGCPDEIYMIMTECWNNNVNQRPSFRDLALRVDQIRDNMAG

>hsa:3718
MAPPSEETPLIPQRSCSLLSTEAGALHVLLPARGPGPPQRLSFSFGDHLAEDLCVQAAKA
SGILPVYHSLFALATEDLSCWFPPSHIFSVEDASTQVLLYRIRFYFPNWFGLEKCHRFGL
RKDLASAILDLPVLEHLFAQHRSDLVSGRLPVGLSLKEQGECLSLAVLDLARMAREQAQR
PGELLKTVSYKACLPPSLRDLIQGLSFVTRRRIRRTVRRALRRVAACQADRHSLMAKYIM
DLERLDPAGAAETFHVGLPGALGGHDGLGLLRVAGDGGIAWTQGEQEVLQPFCDFPEIVD
ISIKQAPRVGPAGEHRLVTVTRTDNQILEAEFPGLPEALSFVALVDGYFRLTTDSQHFFC
KEVAPPRLLEEVAEQCHGPITLDFAINKLKTGGSRPGSYVLRRSPQDFDSFLLTVCVQNP
LGPDYKGCLIRRSPTGTFLLVGLSRPHSSLRELLATCWDGGLHVDGVAVTLTSCCIPRPK
EKSNLIVVQRGHSPPTSSLVQPQSQYQLSQMTFHKIPADSLEWHENLGHGSFTKIYRGCR
HEVVDGEARKTEVLLKVMDAKHKNCMESFLEAASLMSQVSYRHLVLLHGVCMAGDSTMVQ
EFVHLGAIDMYLRKRGHLVPASWKLQVVKQLAYALNYLEDKGLPHGNVSARKVLLAREGA
DGSPPFIKLSDPGVSPAVLSLEMLTDRIPWVAPECLREAQTLSLEADKWGFGATVWEVFS
GVTMPISALDPAKKLQFYEDRQQLPAPKWTELALLIQQCMAYEPVQRPSFRAVIRDLNSL
ISSDYELLSDPTPGALAPRDGLWNGAQLYACQDPTIFEERHLKYISQLGKGNFGSVELCR
YDPLGDNTGALVAVKQLQHSGPDQQRDFQREIQILKALHSDFIVKYRGVSYGPGRQSLRL
VMEYLPSGCLRDFLQRHRARLDASRLLLYSSQICKGMEYLGSRRCVHRDLAARNILVESE
AHVKIADFGLAKLLPLDKDYYVVREPGQSPIFWYAPESLSDNIFSRQSDVWSFGVVLYEL
FTYCDKSCSPSAEFLRMMGCERDVPALCRLLELLEEGQRLPAPPACPAEVHELMKLCWAP
SPQDRPSFSALGPQLDMLWSGSRGCETHAFTAHPEGKHHSLSFS
>hsa:3735
MLTQAAVRLVRGSLRKTSWAEWGHRELRLGQLAPFTAPHKDKSFSDQRSELKRRLKAEKK
VAEKEAKQKELSEKQLSQATAAATNHTTDNGVGPEEESVDPNQYYKIRSQAIHQLKVNGE
DPYPHKFHVDISLTDFIQKYSHLQPGDHLTDITLKVAGRIHAKRASGGKLIFYDLRGEGV
KLQVMANSRNYKSEEEFIHINNKLRRGDIIGVQGNPGKTKKGELSIIPYEITLLSPCLHM
LPHLHFGLKDKETRYRQRYLDLILNDFVRQKFIIRSKIITYIRSFLDELGFLEIETPMMN
IIPGGAVAKPFITYHNELDMNLYMRIAPELYHKMLVVGGIDRVYEIGRQFRNEGIDLTHN
PEFTTCEFYMAYADYHDLMEITEKMVSGMVKHITGSYKVTYHPDGPEGQAYDVDFTPPFR
RINMVEELEKALGMKLPETNLFETEETRKILDDICVAKAVECPPPRTTARLLDKLVGEFL
EVTCINPTFICDHPQIMSPLAKWHRSKEGLTERFELFVMKKEICNAYTELNDPMRQRQLF
EEQAKAKAAGDDEAMFIDENFCTALEYGLPPTAGWGMGIDRVAMFLTDSNNIKEVLLFPA
MKPEDKKENVATTDTLESTTVGTSV
>hsa:377677
MSRLSWGYREHNGPIHWKEFFPIADGDQQSPIEIKTKEVKYDSSLRPLSIKYDPSSAKII
SNSGHSFNVDFDDTENKSVLRGGPLTGSYRLRQVHLHWGSADDHGSEHIVDGVSYAAELH
VVHWNSDKYPSFVEAAHEPDGLAVLGVFLQIGEPNSQLQKITDTLDSIKEKGKQTRFTNF
DLLSLLPPSWDYWTYPGSLTVPPLLESVTWIVLKQPINISSQQLAKFRSLLCTAEGEAAA
FLVSNHRPPQPLKGRKVRASFH
>hsa:3791
MQSKVLLAVALWLCVETRAASVGLPSVSLDLPRLSIQKDILTIKANTTLQITCRGQRDLD
WLWPNNQSGSEQRVEVTECSDGLFCKTLTIPKVIGNDTGAYKCFYRETDLASVIYVYVQD
YRSPFIASVSDQHGVVYITENKNKTVVIPCLGSISNLNVSLCARYPEKRFVPDGNRISWD
SKKGFTIPSYMISYAGMVFCEAKINDESYQSIMYIVVVVGYRIYDVVLSPSHGIELSVGE
KLVLNCTARTELNVGIDFNWEYPSSKHQHKKLVNRDLKTQSGSEMKKFLSTLTIDGVTRS

DQGLYTCAASSGLMTKKNSTFVRVHEKPFVAFGSGMESLVEATVGERVRIPAKYLGYPPP
EIKWYKNGIPLESNHTIKAGHVLTIMEVSERDTGNYTVILTNPISKEKQSHVVSLVVYVP
PQIGEKSLISPVDSYQYGTTQTLTCTVYAIPPPHHIHWYWQLEEECANEPSQAVSVTNPY
PCEEWRSVEDFQGGNKIEVNKNQFALIEGKNKTVSTLVIQAANVSALYKCEAVNKVGRGE
RVISFHVTRGPEITLQPDMQPTEQESVSLWCTADRSTFENLTWYKLGPQPLPIHVGELPT
PVCKNLDTLWKLNATMFSNSTNDILIMELKNASLQDQGDYVCLAQDRKTKKRHCVVRQLT
VLERVAPTITGNLENQTTSIGESIEVSCTASGNPPPQIMWFKDNETLVEDSGIVLKDGNR
NLTIRRVRKEDEGLYTCQACSVLGCAKVEAFFIIEGAQEKTNLEIIILVGTAVIAMFFWL
LLVIILRTVKRANGGELKTGYLSIVMDPDELPLDEHCERLPYDASKWEFPRDRLKLGKPL
GRGAFGQVIEADAFGIDKTATCRTVAVKMLKEGATHSEHRALMSELKILIHIGHHLNVVN
LLGACTKPGGPLMVIVEFCKFGNLSTYLRSKRNEFVPYKTKGARFRQGKDYVGAIPVDLK
RRLDSITSSQSSASSGFVEEKSLSDVEEEEAPEDLYKDFLTLEHLICYSFQVAKGMEFLA
SRKCIHRDLAARNILLSEKNVVKICDFGLARDIYKDPDYVRKGDARLPLKWMAPETIFDR
VYTIQSDVWSFGVLLWEIFSLGASPYPGVKIDEEFCRRLKEGTRMRAPDYTTPEMYQTML
DCWHGEPSQRPTFSELVEHLGNLLQANAQQDGKDYIVLPISETLSMEEDSGLSLPTSPVS
CMEEEEVCDPKFHYDNTAGISQYLQNSKRKSRPVSVKTFEDIPLEEPEVKVIPDDNQTDS
GMVLASEELKTLEDRTKLSPSFGGMVPSKSRESVASEGSNQTSGYQSGYHSDDTDTTVYS
SEEAELLKLIEIGVQTGSTAQILQPDSGTTLSSPPV

>hsa:38

MAVLAALLRSGARSRSPLLRRLVQEIRYVERSYVSKPTLKEVVIVSATRTPIGSFLGSLS
LLPATKLGSIAIQGAIEKAGIPKEEVKEAYMGNVLQGGEGQAPTRQAVLGAGLPISTPCT
TINKVCASGMKAIMMASQSLMCGHQDVMVAGGMESMSNVPYVMNRGSTPYGGVKLEDLIV
KDGLTDVYNKIHMGSCAENTAKKLNIARNEQDAYAINSYTRSKAAWEAGKFGNEVIPVTV
TVKGQPDVVVKEDEEYKRVDFSKVPKLKTVFQKENGTVTAANASTLNDGAAALVLMTADA
AKRLNVTPLARIVAFADAAVEPIDFPIAPVYAASMVLKDVGLKKEDIAMWEVNEAFSLVV
LANIKMLEIDPQKVNINGGAVSLGHPIGMSGARIVGHLTHALKQGEYGLASICNGGGGAS
AMLIQKL

>hsa:3815

MRGARGAWDFLCVLLLLLRVQTGSSQPSVSPGEPSPPSIHPGKSDLIVRVGDEIRLLCTD
PGFVKWTFEILDETNENKQNEWITEKAEATNTGKYTCTNKHGLSNSIYVFVRDPAKLFLV
DRSLYGKEDNDTLVRCPLTDPEVTNYSLKGCQGKPLPKDLRFIPDPKAGIMIKSVKRAYH
RLCLHCSVDQEGKSVLSEKFILKVRPAFKAVPVVSVSKASYLLREGEEFTVTCTIKDVSS
SVYSTWKRENSQTKLQEKYNSWHHGDFNYERQATLTISSARVNDSGVFMCYANNTFGSAN
VTTTLEVVDKGFINIFPMINTTVFVNDGENVDLIVEYEAFPKPEHQQWIYMNRTFTDKWE
DYPKSENESNIRYVSELHLTRLKGTEGGTYTFLVSNSDVNAAIAFNVYVNTKPEILTYDR
LVNGMLQCVAAGFPEPTIDWYFCPGTEQRCSASVLPVDVQTLNSSGPPFGKLVVQSSIDS
SAFKHNGTVECKAYNDVGKTSAYFNFAFKGNNKEQIHPHTLFTPLLIGFVIVAGMMCIIV
MILTYKYLQKPMYEVQWKVVEEINGNNYVYIDPTQLPYDHKWEFPRNRLSFGKTLGAGAF
GKVVEATAYGLIKSDAAMTVAVKMLKPSAHLTEREALMSELKVLSYLGNHMNIVNLLGAC
TIGGPTLVITEYCCYGDLLNFLRRKRDSFICSKQEDHAEAALYKNLLHSKESSCSDSTNE
YMDMKPGVSYVVPTKADKRRSVRIGSYIERDVTPAIMEDDELALDLEDLLSFSYQVAKGM
AFLASKNCIHRDLAARNILLTHGRITKICDFGLARDIKNDSNYVVKGNARLPVKWMAPES
IFNCVYTFESDVWSYGIFLWELFSLGSSPYPGMPVDSKFYKMIKEGFRMLSPEHAPAEMY
DIMKTCWDADPLKRPTFKQIVQLIEKQISESTNHIYSNLANCSPNRQKPVVDHSVRINSV

GSTASSSQPLLVHDDV
>hsa:3816
MWFLVLCLALSLGGTGAAPPIQSRIVGGWECEQHSQPWQAALYHFSTFQCGGILVHRQWV
LTAAHCISDNYQLWLGRHNLFDDENTAQFVHVSESFPHPGFNMSLLENHTRQADEDYSHD
LMLLRLTEPADTITDAVKVVELPTEEPEVGSTCLASGWGSIEPENFSFPDDLQCVDLKIL
PNDECKKAHVQKVTDFMLCVGHLEGGKDTCVGDSGGPLMCDGVLQGVTSWGYVPCGTPNK
PSVAVRVLSYVKWIEDTIAENS
>hsa:3817
MWDLVLSIALSVGCTGAVPLIQSRIVGGWECEKHSQPWQVAVYSHGWAHCGGVLVHPQWV
LTAAHCLKKNSQVWLGRHNLFEPEDTGQRVPVSHSFPHPLYNMSLLKHQSLRPDEDSSHD
LMLLRLSEPAKITDVVKVLGLPTQEPALGTTCYASGWGSIEPEEFLRPRSLQCVSLHLLS
NDMCARAYSEKVTEFMLCAGLWTGGKDTCGVSHPYSQHLEGKG
>hsa:3818
MILFKQATYFISLFATVSCGCLTQLYENAFFRGGDVASMYTPNAQYCQMRCTFHPRCLLF
SFLPASSINDMEKRFGCFLKDSVTGTLPKVHRTGAVSGHSLKQCGHQISACHRDIYKGVD
MRGVNFNVSKVSSVEECQKRCTSNIRCQFFSYATQTFHKAEYRNNCLLKYSPGGTPTAIK
VLSNVESGFSLKPCALSEIGCHMNIFQHLAFSDVDVARVLTPDAFVCRTICTYHPNCLFF
TFYTNVWKIESQRNVCLLKTSESGTPSSSTPQENTISGYSLLTCKRTLPEPCHSKIYPGV
DFGGEELNVTFVKGVNVCQETCTKMIRCQFFTYSLLPEDCKEEKCKCFLRLSMDGSPTRI
AYGTQGSSGYSLRLCNTGDNSVCTTKTSTRIVGGTNSSWGEWPWQVSLQVKLTAQRHLCG
GSLIGHQWVLTAAHCFDGLPLQDVWRIYSGILNLSDITKDTPFSQIKEIIIHQNYKVSEG
NHDIALIKLQAPLNYTEFQKPICLPSKGDTSTIYTNCWVTGWGFSKEKGEIQNILQKVNI
PLVTNEECQKRYQDYKITQRMVCAGYKEGGKDACKGDSGGPLVCKHNGMWRLVGITSWGE
GCARREQPGVYTKVAEYMDWILEKTQSSDGKAQMQSPA
>hsa:3906
MRFFVPLFLVGILFPAILAKQFTKCELSQLLKDIDGYGGIALPELICTMFHTSGYDTQAI
VENNESTEYGLFQISNKLWCKSSQVPQSRNICDISCDKFLDDDITDDIMCAKKILDIKGI
DYWLAHKALCTEKLEQWLCEKL
>hsa:390956 peptidyl-prolyl cis-trans isomerase A-like (A)
MDFLHLGLEQAEYNLGCPGYRSWFSICRYREDATVNPTVFFDIAVDGEPLGHVSFELFAD
KFPKSAEKVHALSTGEKGFGYKGSCFHRIIPGFMCQGGDFTRHKDTGGKSLYREKFDDEN
FILKHTGPGILSMANAGPNTNCSQCFICTAKTEGLDGKHVVFGTVKEGMKIVEAMEYFGP
RNGKTSKEITIADCEQL
>hsa:3932
MGCGCSSHPEDDWMENIDVCENCHYPIVPLDGKGTLLIRNGSEVRDPLVTYEGSNPPASP
LQDNLVIALHSYEPSHDGDLGFEKGEQLRILEQSGEWWKAQSLTTGQEGFIPFNFVAKAN
SLEPEPWFFKNLSRKDAERQLLAPGNTHGSFLIRESESTAGSFSLSVRDFDQNQGEVVKH
YKIRNLDNGGFYISPRITFPGLHELVRHYTNASDGLCTRLSRPCQTQKPQKPWWEDEWEV
PRETLKLVERLGAGQFGEVWMGYYNGHTKVAVKSLKQGSMSPDAFLAEANLMKQLQHQRL
VRLYAVVTQEPIYIITEYMENGSLVDFLKTPSGIKLTINKLLDMAAQIAEGMAFIEERNY
IHRDLRAANILVSDTLSCKIADFGLARLIEDNEYTAREGAKFPIKWTAPEAINYGTFTIK
SDVWSFGILLTEIVTHGRIPYPGMTNPEVIQNLERGYRMVRPDNCPEELYQLMRLCWKER
PEDRPTFDYLRSVLEDFFTATEGQYQPQP
>hsa:3939

MATLKDQLIYNLLKEEQTPQNKITVVGVGAVGMACAISILMKDLADELALVDVIEDKLKG
EMMDLQHGSLFLRTPKIVSGKVDILTYVAWKISGFPKNRVIGSGCNLDSARFRYLMGERL
GVHPLSCHGWVLGEHGDSSVPVWSGMNVAGVSLKTLHPDLGTDKDKEQWKEVHKQVVESA
YEVIKLKGYTSWAIGLSVADLAESIMKNLRRVHPVSTMIKGLYGIKDDVFLSVPCILGQN
GISDLVKVTLTSEEEARLKKSADTLWGIQKELQF
>hsa:3945
MATLKEKLIAPVAEEEATVPNNKITVVGVGQVGMACAISILGKSLADELALVDVLEDKLK
GEMMDLQHGSLFLQTPKIVADKDYSVTANSKIVVVTAGVRQQEGESRLNLVQRNVNVFKF
IIPQIVKYSPDCIIIVVSNPVDILTYVTWKLSGLPKHRVIGSGCNLDSARFRYLMAEKLG
IHPSSCHGWILGEHGDSSVAVWSGVNVAGVSLQELNPEMGTDNDSENWKEVHKMVVESAY
EVIKLKGYTNWAIGLSVADLIESMLKNLSRIHPVSTMVKGMYGIENEVFLSLPCILNARG
LTSVINQKLKDDEVAQLKKSADTLWDIQKDLKDL
>hsa:3988
MKMRFLGLVVCLVLWTLHSEGSGGKLTAVDPETNMNVSEIISYWGFPSEEYLVETEDGYI
LCLNRIPHGRKNHSDKGPKPVVFLQHGLLADSSNWVTNLANSSLGFILADAGFDVWMGNS
RGNTWSRKHKTLSVSQDEFWAFSYDEMAKYDLPASINFILNKTGQEQVYYVGHSQGTTIG
FIAFSQIPELAKRIKMFFALGPVASVAFCTSPMAKLGRLPDHLIKDLFGDKEFLPQSAFL
KWLGTHVCTHVILKELCGNLCFLLCGFNERNLNMSRVDVYTTHSPAGTSVQNMLHWSQAV
KFQKFQAFDWGSSAKNYFHYNQSYPPTYNVKDMLVPTAVWSGGHDWLADVYDVNILLTQI
TNLVFHESIPEWEHLDFIWGLDAPWRLYNKIINLMRKYQ
>hsa:3990
MDTSPLCFSILLVLCIFIQSSALGQSLKPEPFGRRAQAVETNKTLHEMKTRFLLFGETNQ
GCQIRINHPDTLQECGFNSSLPLVMIIHGWSVDGVLENWIWQMVAALKSQPAQPVNVGLV
DWITLAHDHYTIAVRNTRLVGKEVAALLRWLEESVQLSRSHVHLIGYSLGAHVSGFAGSS
IGGTHKIGRITGLDAAGPLFEGSAPSNRLSPDDANFVDAIHTFTREHMGLSVGIKQPIGH
YDFYPNGGSFQPGCHFLELYRHIAQHGFNAITQTIKCSHERSVHLFIDSLLHAGTQSMAY
PCGDMNSFSQGLCLSCKKGRCNTLGYHVRQEPRSKSKRLFLVTRAQSPFKVYHYQFKIQF
INQTETPIQTTFTMSLLGTKEKMQKIPITLGKGIASNKTYSFLITLDVDIGELIMIKFKW
ENSAVWANVWDTVQTIIPWSTGPRHSGLVLKTIRVKAGETQQRMTFCSENTDDLLLRPTQ
EKIFVKCEIKSKTSKRKIR
>hsa:3991
MEPGSKSVSRSDWQPEPHQRPITPLEPGPEKTPIAQPESKTLQGSNTQQKPASNQRPLTQ
QETPAQHDAESQKEPRAQQKSASQEEFLAPQKPAPQQSPYIQRVLLTQQEAASQQGPGLG
KESITQQEPALRQRHVAQPGPGPGEPPPAQQEAESTPAAQAKPGAKREPSAPTESTSQET
PEQSDKQTTPVQGAKSKQGSLTELGFLTKLQELSIQRSALEWKALSEWVTDSESESDVGS
SSDTDSPATMGGMVAQGVKLGFKGKSGYKVMSGYSGTSPHEKTSARNHRHYQDTASRLIH
NMDLRTMTQSLVTLAEDNIAFFSSQGPGETAQRLSGVFAGVREQALGLEPALGRLLGVAH
LFDLDPETPANGYRSLVHTARCCLAHLLHKSRYVASNRRSIFFRTSHNLAELEAYLAALT
QLRALVYYAQRLLVTNRPGVLFFEGDEGLTADFLREYVTLHKGCFYGRCLGFQFTPAIRP
FLQTISIGLVSFGEHYKRNETGLSVAASSLFTSGRFAIDPELRGAEFERITQNLDVHFWK
AFWNITEMEVLSSLANMASATVRVSRLLSLPPEAFEMPLTADPTLTVTISPPLAHTGPGP
VLVRLISYDLREGQDSEELSSLIKSNGQRSLELWPRPQQAPRSRSLIVHFHGGGFVAQTS
RSHEPYLKSWAQELGAPIISIDYSLAPEAPFPRALEECFFAYCWAIKHCALLGSTGERIC
LAGDSAGGNLCFTVALRAAAYGVRVPDGIMAAYPATMLQPAASPSRLLSLMDPLLPLSVL

SKCVSAYAGAKTEDHSNSDQKALGMMGLVRRDTALLLRDFRLGASSWLNSFLELSGRKSQ
KMSEPIAEPMRRSVSEAALAQPQGPLGTDSLKNLTLRDLSLRGNSETSSDTPEMSLSAET
LSPSTPSDVNFLLPPEDAGEEAEAKNELSPMDRGLGVRAAFPEGFHPRRSSQGATQMPLY
SSPIVKNPFMSPLLAPDSMLKSLPPVHIVACALDPMLDDSVMLARRLRNLGQPVTLRVVE
DLPHGFLTLAALCRETRQAAELCVERIRLVLTPPAGAGPSGETGAAGVDGGCGGRH
>hsa:4017
MERPLCSHLCSCLAMLALLSPLSLAQYDSWPHYPEYFQQPAPEYHQPQAPANVAKIQLRL
AGQKRKHSEGRVEVYYDGQWGTVCDDDFSIHAAHVVCRELGYVEAKSWTASSSYGKGEGP
IWLDNLHCTGNEATLAACTSNGWGVTDCKHTEDVGVVCSDKRIPGFKFDNSLINQIENLN
IQVEDIRIRAILSTYRKRTPVMEGYVEVKEGKTWKQICDKHWTAKNSRVVCGMFGFPGER
TYNTKVYKMFASRRKQRYWPFSMDCTGTEAHISSCKLGPQVSLDPMKNVTCENGLPAVVS
CVPGQVFSPDGPSRFRKAYKPEQPLVRLRGGAYIGEGRVEVLKNGEWGTVCDDKWDLVSA
SVVCRELGFGSAKEAVTGSRLGQGIGPIHLNEIQCTGNEKSIIDCKFNAESQGCNHEEDA
GVRCNTPAMGLQKKLRLNGGRNPYEGRVEVLVERNGSLVWGMVCGQNWGIVEAMVVCRQL
GLGFASNAFQETWYWHGDVNSNKVVMSGVKCSGTELSLAHCRHDGEDVACPQGGVQYGAG
VACSETAPDLVLNAEMVQQTTYLEDRPMFMLQCAMEENCLSASAAQTDPTTGYRRLLRFS
SQIHNNGQSDFRPKNGRHAWIWHDCHRHYHSMEVFTHYDLLNLNGTKVAEGHKASFCLED
TECEGDIQKNYECANFGDQGITMGCWDMYRHDIDCQWVDITDVPPGDYLFQVVINPNFEV
AESDYSNNIMKCRSRYDGHRIWMYNCHIGGSFSEETEKKFEHFSGLLNNQLSPQ
>hsa:4023
MESKALLVLTLAVWLQSLTASRGGVAAADQRRDFIDIESKFALRTPEDTAEDTCHLIPGV
AESVATCHFNHSSKTFMVIHGWTVTGMYESWVPKLVAALYKREPDSNVIVVDWLSRAQEH
YPVSAGYTKLVGQDVARFINWMEEEFNYPLDNVHLLGYSLGAHAAGIAGSLTNKKVNRIT
GLDPAGPNFEYAEAPSRLSPDDADFVDVLHTFTRGSPGRSIGIQKPVGHVDIYPNGGTFQ
PGCNIGEAIRVIAERGLGDVDQLVKCSHERSIHLFIDSLLNEENPSKAYRCSSKEAFEKG
LCLSCRKNRCNNLGYEINKVRAKRSSKMYLKTRSQMPYKVFHYQVKIHFSGTESETHTNQ
AFEISLYGTVAESENIPFTLPEVSTNKTYSFLIYTEVDIGELLMLKLKWKSDSYFSWSDW
WSSPGFAIQKIRVKAGETQKKVIFCSREKVSHLQKGKAPAVFVKCHDKSLNKKSG
>hsa:4025
MRVLLHLPALLASLILLQAAASTTRDPSLDLTSLSLEVGCGAPAPVVRCDPCSPYRTITG
DCNNRRKPALGAANRALARWLPAEYEDGLSLPFGWTPGKTRNGFPLPLAREVSNKIVGYL
NEEGVLDQNRSLLFMQWGQIVDHDLDFAPDTELGSSEYSKAQCDEYCIQGDNCFPIMFPP
NDPKAGTQGKCMPFFRAGFVCPTPPYKSLAREQINALTSFLDASFVYSSEPSLASRLRNL
SSPLGLMAVNQEVSDHGLPYLPYDSKKPSPCEFINTTARVPCFLAGDSRASEHILLATSH
TLFLREHNRLARELKRLNPQWDGEKLYQEARKILGAFVQIITFRDYLPILLGDHMQKWIP
PYQGYSESVDPRISNVFTFAFRFGHLEVPSSMFRLDENYQPWGPEPELPLHTLFFNTWRM
VKDGGIDPLVRGLLAKKSKLMKQNKMMTGELRNKLFQPTHRIHGFDLAAINTQRCRDHGQ
PGYNSWRAFCDLSQPQTLEELNTVLKSKMLAKKLLGLYGTPDNIDIWIGAIAEPLVERGR
VGPLLACLLGKQFQQIRDGDRFWWENPGVFTNEQKDSLQKMSFSRLVCDNTRITKVPRDP
FWANSYPYDFVDCSAIDKLDLSPWASVKN
>hsa:4048
MPEIVDTCSLASPASVCRTKHLHLRCSVDFTRRTLTGTAALTVQSQEDNLRSLVLDTKDL
TIEKVVINGQEVKYALGERQSYKGSPMEISLPIALSKNQEIVIEISFETSPKSSALQWLT
PEQTSGKEHPYLFSQCQAIHCRAILPCQDTPSVKLTYTAEVSVPKELVALMSAIRDGETP

DPEDPSRKIYKFIQKVPIPCYLIALVVGALESRQIGPRTLVWSEKEQVEKSAYEFSETES
MLKIAEDLGGPYVWGQYDLLVLPPSFPYGGMENPCLTFVTPTLLAGDKSLSNVIAHEISH
SWTGNLVTNKTWDHFWLNEGHTVYLERHICGRLFGEKFRHFNALGGWGELQNSVKTFGET
HPFTKLVVDLTDIDPDVAYSSVPYEKGFALLFYLEQLLGGPEIFLGFLKAYVEKFSYKSI
TTDDWKDFLYSYFKDKVDVLNQVDWNAWLYSPGLPPIKPNYDMTLTNACIALSQRWITAK
EDDLNSFNATDLKDLSSHQLNEFLAQTLQRAPLPLGHIKRMQEVYNFNAINNSEIRFRWL
RLCIQSKWEDAIPLALKMATEQGRMKFTRPLFKDLAAFDKSHDQAVRTYQEHKASMHPVT
AMLVGKDLKVD
>hsa:4051
MPQLSLSSLGLWPMAASPWLLLLLVGASWLLARILAWTYTFYDNCCRLRCFPQPPKRNWF
LGHLGLIHSSEEGLLYTQSLACTFGDMCCWWVGPWHAIVRIFHPTYIKPVLFAPAAIVPK
DKVFYSFLKPWLGDGLLLSAGEKWSRHRRMLTPAFHFNILKPYMKIFNESVNIMHAKWQL
LASEGSARLDMFEHISLMTLDSLQKCVFSFDSHCQEKPSEYIAAILELSALVTKRHQQIL
LYIDFLYYLTPDGQRFRRACRLVHDFTDAVIQERRRTLPSQGVDDFLQAKAKSKTLDFID
VLLLSKDEDGKKLSDEDIRAEADTFMFEGHDTTASGLSWVLYHLAKHPEYQERCRQEVQE
LLKDREPKEIEWDDLAQLPFLTMCIKESLRLHPPVPAVSRCCTQDIVLPDGRVIPKGIIC
LISVFGTHHNPAVWPDPEVYDPFRFDPKNIKERSPLAFIPFSAGPRNCIGQAFAMAEMKV
VLGLTLLRFRVLPDHTEPRRKPELVLRAEGGLWLRVEPLS
>hsa:4058
MGCWGQLLVWFGAAGAILCSSPGSQETFLRSSPLPLASPSPRDPKVSAPPSILEPASPLN
SPGTEGSWLFSTCGASGRHGPTQTQCDGAYAGTSVVVTVGAAGQLRGVQLWRVPGPGQYL
ISAYGAAGGKGAKNHLSRAHGVFVSAIFSLGLGESLYILVGQQGEDACPGGSPESQLVCL
GESRAVEEHAAMDGSEGVPGSRRWAGGGGGGGGATYVFRVRAGELEPLLVAAGGGGRAYL
RPRDRGRTQASPEKLENRSEAPGSGGRGGAAGGDASETDNLWADGEDGVSFIHPSSELFL
QPLAVTENHGEVEIRRHLNCSHCPLRDCQWQAELQLAECLCPEGMELAVDNVTCMDLHKP
PGPLVLMVAVVATSTLSLLMVCGVLILGGAWPGPVLASATRCHRGFPSQCYSAQTLPELC
SPQDELDFLMEALIISKFRHQNIVRCVGLSLRATPRLILLELMSGGDMKSFLRHSRPHLG
QPSPLVMRDLLQLAQDIAQGCHYLEENHFIHRDIAARNCLLSCAGPSRVAKIGDFGMARD
IYRASYYRRGDRALLPVKWMPPEAFLEGIFTSKTDSWSFGVLLWEIFSLGYMPYPGRTNQ
EVLDFVVGGGRMDPPRGCPGPVYRIMTQCWQHEPELRPSFASILERLQYCTQDPDVLNSL
LPMELGPTPEEEGTSGLGNRSLECLRPPQPQELSPEKLKSWGGSPLGPWLSSGLKPLKSR
GLQPQNLWNPTYRS
>hsa:4067
MGCIKSKGKDSLSDDGVDLKTQPVPESQLLPGQRFQTKDPEEQGDIVVALYPYDGIHPDD
LSFKKGEKMKVLEEHGEWWKAKSLLTKKEGFIPSNYVAKLNTLETEEWFFKDITRKDAER
QLLAPGNSAGAFLIRESETLKGSFSLSVRDFDPVHGDVIKHYKIRSLDNGGYYISPRITF
PCISDMIKHYQKQADGLCRRLEKACISPKPQKPWDKDAWEIPRESIKLVKRLGAGQFGEV
WMGYYNNSTKVAVKTLKPGTMSVQAFLEEANLMKTLQHDKLVRLYAVVTREEPIYIITEY
MAKGSLLDFLKSDEGGKVLLPKLIDFSAQIAEGMAYIERKNYIHRDLRAANVLVSESLMC
KIADFGLARVIEDNEYTAREGAKFPIKWTAPEAINFGCFTIKSDVWSFGILLYEIVTYGK
IPYPGRTNADVMTALSQGYRMPRVENCPDELYDIMKMCWKEKAEERPTFDYLQSVLDDFY
TATEGQYQQQP
>hsa:4128
MENQEKASIAGHMFDVVVIGGGISGLSAAKLLTEYGVSVLVLEARDRVGGRTYTIRNEHV

DYVDVGGAYVGPTQNRILRLSKELGIETYKVNVSERLVQYVKGKTYPFRGAFPPVWNPIA
YLDYNNLWRTIDNMGKEIPTDAPWEAQHADKWDKMTMKELIDKICWTKTARRFAYLFVNI
NVTSEPHEVSALWFLWYVKQCGGTTRIFSVTNGGQERKFVGGSGQVSERIMDLLGDQVKL
NHPVTHVDQSSDNIIIETLNHEHYECKYVINAIPPTLTAKIHFRPELPAERNQLIQRLPM
GAVIKCMMYYKEAFWKKKDYCGCMIIEDEDAPISITLDDTKPDGSLPAIMGFILARKADR
LAKLHKEIRKKKICELYAKVLGSQEALHPVHYEEKNWCEEQYSGGCYTAYFPPGIMTQYG
RVIRQPVGRIFFAGTETATKWSGYMEGAVEAGERAAREVLNGLGKVTEKDIWVQEPESKD
VPAVEITHTFWERNLPSVSGLLKIIGFSTSVTALGFVLYKYKLLPRS
>hsa:4129
MSNKCDVVVVGGGISGMAAAKLLHDSGLNVVVLEARDRVGGRTYTLRNQKVKYVDLGGSY
VGPTQNRILRLAKELGLETYKVNEVERLIHHVKGKSYPFRGPFPPVWNPITYLDHNNFWR
TMDDMGREIPSDAPWKAPLAEEWDNMTMKELLDKLCWTESAKQLATLFVNLCVTAETHEV
SALWFLWYVKQCGGTTRIISTTNGGQERKFVGGSGQVSERIMDLLGDRVKLERPVIYIDQ
TRENVLVETLNHEMYEAKYVISAIPPTLGMKIHFNPPLPMMRNQMITRVPLGSVIKCIVY
YKEPFWRKKDYCGTMIIDGEEAPVAYTLDDTKPEGNYAAIMGFILAHKARKLARLTKEER
LKKLCELYAKVLGSLEALEPVHYEEKNWCEEQYSGGCYTTYFPPGILTQYGRVLRQPVDR
IYFAGTETATHWSGYMEGAVEAGERAAREILHAMGKIPEDEIWQSEPESVDVPAQPITTT
FLERHLPSVPGLLRLIGLTTIFSATALGFLAHKRGLLVRV
>hsa:4143
MNGPVDGLCDHSLSEGVFMFTSESVGEGHPDKICDQISDAVLDAHLKQDPNAKVACETVC
KTGMVLLCGEITSMAMVDYQRVVRDTIKHIGYDDSAKGFDFKTCNVLVALEQQSPDIAQC
VHLDRNEEDVGAGDQGLMFGYATDETEECMPLTIILAHKLNARMADLRRSGLLPWLRPDS
KTQVTVQYMQDNGAVIPVRIHTIVISVQHNEDITLEEMRRALKEQVIRAVVPAKYLDEDT
VYHLQPSGRFVIGGPQGDAGVTGRKIIVDTYGGWGAHGGGAFSGKDYTKVDRSAAYAARW
VAKSLVKAGLCRRVLVQVSYAIGVAEPLSISIFTYGTSQKTERELLDVVHKNFDLRPGVI
VRDLDLKKPIYQKTACYGHFGRSEFPWEVPRKLVF
>hsa:4145
MQGHFPAERREGRPRRGTRGQQQLLVSPRFLRAWHPPPVSARMPTRRWAPGTQCITKCEH
TRPKPGELAFRKGDVVTILEACENKSWYRVKHHTSGQEGLLAAGALREREALSADPKLSL
MPWFHGKISGQEAVQQLQPPEDGLFLVRESARHPGDYVLCVSFGRDVIHYRVLHRDGHLT
IDEAVFFCNLMDMVEHYSKDKGAICTKLVRPKRKHGTKSAEEELARAGWLLNLQHLTLGA
QIGEGEFGAVLQGEYLGQKVAVKNIKCDVTAQAFLDETAVMTKMQHENLVRLLGVILHQG
LYIVMEHVSKGNLVNFLRTRGRALVNTAQLLQFSLHVAEGMEYLESKKLVHRDLAARNIL
VSEDLVAKVSDFGLAKAERKGLDSSRLPVKWTAPEALKHGKFTSKSDVWSFGVLLWEVFS
YGRAPYPKMSLKEVSEAVEKGYRMEPPEGCPGPVHVLMSSCWEAEPARRPPFRKLAEKLA
RELRSAGAPASVSGQDADGSTSPRSQEP
>hsa:4190
MRRCSYFPKDVTVFDKDDKSEPIRVLVTGAAGQIAYSLLYSIGNGSVFGKDQPIILVLLD
ITPMMGVLDGVLMELQDCALPLLKDVIATDKEDVAFKDLDVAILVGSMPRREGMERKDLL
KANVKIFKSQGAALDKYAKKSVKVIVVGNPANTNCLTASKSAPSIPKENFSCLTRLDHNR
AKAQIALKLGVTANDVKNVIIWGNHSSTQYPDVNHAKVKLQGKEVGVYEALKDDSWLKGE
FVTTVQQRGAAVIKARKLSSAMSAAKAICDHVRDIWFGTPEGEFVSMGVISDGNSYGVPD
DLLYSFPVVIKNKTWKFVEGLPINDFSREKMDLTAKELTEEKESAFEFLSSA
>hsa:4191

MLSALARPASAALRRSFSTSAQNNAKVAVLGASGGIGQPLSLLLKNSPLVSRLTLYDIAH
TPGVAADLSHIETKAAVKGYLGPEQLPDCLKGCDVVVIPAGVPRKPGMTRDDLFNTNATI
VATLTAACAQHCPEAMICVIANPVNSTIPITAEVFKKHGVYNPNKIFGVTTLDIVRANTF
VAELKGLDPARVNVPVIGGHAGKTIIPLISQCTPKVDFPQDQLTALTGRIQEAGTEVVKA
KAGAGSATLSMAYAGARFVFSLVDAMNGKEGVVECSFVKSQETECTYFSTPLLLGKKGIE
KNLGIGKVSSFEEKMISDAIPELKASIKKGEDFVKTLK

>hsa:4200

MLSRLRVVSTTCTLACRHLHIKEKGKPLMLNPRTNKGMAFTLQERQMLGLQGLLPPKIET
QDIQALRFHRNLKKMTSPLEKYIYIMGIQERNEKLFYRILQDDIESLMPIVYTPTVGLAC
SQYGHIFRRPKGLFISISDRGHVRSIVDNWPENHVKAVVVTDGERILGLGDLGVYGMGIP
VGKLCLYTACAGIRPDRCLPVCIDVGTDNIALLKDPFYMGLYQKRDRTQQYDDLIDEFMK
AITDRYGRNTLIQFEDFGNHNAFRFLRKYREKYCTFNDDIQGTAAVALAGLLAAQKVISK
PISEHKILFLGAGEAALGIANLIVMSMVENGLSEQEAQKKIWMFDKYGLLVKGRKAKIDS
YQEPFTHSAPESIPDTFEDAVNILKPSTIIGVAGAGRLFTPDVIRAMASINERPVIFALS
NPTAQAECTAEEAYTLTEGRCLFASGSPFGPVKLTDGRVFTPGQGNNVYIFPGYRIPIC

>hsa:4233

MKAPAVLAPGILVLLFTLVQRSNGECKEALAKSEMNVNMKYQLPNFTAETPIQNVILHEH
HIFLGATNYIYVLNEEDLQKVAEYKTGPVLEHPDCFPCQDCSSKANLSGGVWKDNINMAL
VVDTYYDDQLISCGSVNRGTCQRHVFPHNHTADIQSEVHCIFSPQIEEPSQCPDCVVSAL
GAKVLSSVKDRFINFFVGNTINSSYFPDHPLHSISVRRLKETKDGFMFLTDQSYIDVLPE
FRDSYPIKYVHAFESNNFIYFLTVQRETLDAQTFHTRIIRFCSINSGLHSYMEMPLECIL
TEKRKKRSTKKEVFNILQAAYVSKPGAQLARQIGASLNDDILFGVFAQSKPDSAEPMDRS
AMCAFPIKYVNDFFNKIVNKNNVRCLQHFYGPNHEHCFNRTLLRNSSGCEARRDEYRTEF
TTALQRVDLFMGQFSEVLLTSISTFIKGDLTIANLGTSEGRFMQVVVSRSGPSTPHVNFL
LDSHPVSPEVIVEHTLNQNGYTLVITGKKITKIPLNGLGCRHFQSCSQCLSAPPFVQCGW
CHDKCVRSEECLSGTWTQQICLPAIYKVFPNSAPLEGGTRLTICGWDFGFRRNNKFDLKK
TRVLLGNESCTLTLSESTMNTLKCTVGPAMNKHFNMSIIISNGHGTTQYSTFSYVDPVIT
SISPKYGPMAGGTLLTLTGNYLNSGNSRHISIGGKTCTLKSVSNSILECYTPAQTISTEF
AVKLKIDLANRETSIFSYREDPIVYEIHPTKSFISGGSTITGVGKNLNSVSVPRMVINVH
EAGRNFTVACQHRSNSEIICCTTPSLQQLNLQLPLKTKAFFMLDGILSKYFDLIYVHNPV
FKPFEKPVMISMGNENVLEIKGNDIDPEAVKGEVLKVGNKSCENIHLHSEAVLCTVPNDL
LKLNSELNIEWKQAISSTVLGKVIVQPDQNFTGLIAGVVSISTALLLLLGFFLWLKKRKQ
IKDLGSELVRYDARVHTPHLDRLVSARSVSPTTEMVSNESVDYRATFPEDQFPNSSQNGS
CRQVQYPLTDMSPILTSGDSDISSPLLQNTVHIDLSALNPELVQAVQHVVIGPSSLIVHF
NEVIGRGHFGCVYHGTLLDNDGKKIHCAVKSLNRITDIGEVSQFLTEGIIMKDFSHPNVL
SLLGICLRSEGSPLVVLPYMKHGDLRNFIRNETHNPTVKDLIGFGLQVAKGMKYLASKKF
VHRDLAARNCMLDEKFTVKVADFGLARDMYDKEYYSVHNKTGAKLPVKWMALESLQTQKF
TTKSDVWSFGVLLWELMTRGAPPYPDVNTFDITVYLLQGRRLLQPEYCPDPLYEVMLKCW
HPKAEMRPSFSELVSRISAIFSTFIGEHYVHVNATYVNVKCVAPYPSLLSSEDNADDEVD
TRPASFWETS

>hsa:4282

MPMFIVNTNVPRASVPDGFLSELTQQLAQATGKPPQYIAVHVVPDQLMAFGGSSEPCALC
SLHSIGKIGGAQNRSYSKLLCGLLAERLRISPDRVYINYYDMNAANVGWNNSTFA

>hsa:43

MRPPQCLLHTPSLASPLLLLLLLWLLGGGVGAEGREDAELLVTVRGGRLRGIRLKTPGGPV
SAFLGIPFAEPPMGPRRFLPPEPKQPWSGVVDATTFQSVCYQYVDTLYPGFEGTEMWNPN
RELSEDCLYLNVWTPYPRPTSPTPVLVWIYGGGFYSGASSLDVYDGRFLVQAERTVLVSM
NYRVGAFGFLALPGSREAPGNVGLLDQRLALQWVQENVAAFGGDPTSVTLFGESAGAASV
GMHLLSPPSRGLFHRAVLQSGAPNGPWATVGMGEARRRATQLAHLVGCPPGGTGGNDTEL
VACLRTRPAQVLVNHEWHVLPQESVFRFSFVPVVDGDFLSDTPEALINAGDFHGLQVLVG
VVKDEGSYFLVYGAPGFSKDNESLISRAEFLAGVRVGVPQVSDLAAEAVVLHYTDWLHPE
DPARLREALSDVVGDHNVVCPVAQLAGRLAAQGARVYAYVFEHRASTLSWPLWMGVPHGY
EIEFIFGIPLDPSRNYTAEEKIFAQRLMRYWANFARTGDPNEPRDPKAPQWPPYTAGAQQ
YVSLDLRPLEVRRGLRAQACAFWNRFLPKLLSATDTLDEAERQWKAEFHRWSSYMVHWKN
QFDHYSKQDRCSDL
>hsa:4311
MGKSESQMDITDINTPKPKKKQRWTPLEISLSVLVLLLTIIAVTMIALYATYDDGICKSS
DCIKSAARLIQNMDATTEPCTDFFKYACGGWLKRNVIPETSSRYGNFDILRDELEVVLKD
VLQEPKTEDIVAVQKAKALYRSCINESAIDSRGGEPLLKLLPDIYGWPVATENWEQKYGA
SWTAEKAIAQLNSKYGKKVLINLFVGTDDKNSVNHVIHIDQPRLGLPSRDYYECTGIYKE
ACTAYVDFMISVARLIRQEERLPIDENQLALEMNKVMELEKEIANATAKPEDRNDPMLLY
NKMTLAQIQNNFSLEINGKPFSWLNFTNEIMSTVNISITNEEDVVVYAPEYLTKLKPILT
KYSARDLQNLMSWRFIMDLVSSLSRTYKESRNAFRKALYGTTSETATWRRCANYVNGNME
NAVGRLYVEAAFAGESKHVVEDLIAQIREVFIQTLDDLTWMDAETKKRAEEKALAIKERI
GYPDDIVSNDNKLNNEYLELNYKEDEYFENIIQNLKFSQSKQLKKLREKVDKDEWISGAA
VVNAFYSSGRNQIVFPAGILQPPFFSAQQSNSLNYGGIGMVIGHEITHGFDDNGRNFNKD
GDLVDWWTQQSASNFKEQSQCMVYQYGNFSWDLAGGQHLNGINTLGENIADNGGLGQAYR
AYQNYIKKNGEEKLLPGLDLNHKQLFFLNFAQVWCGTYRPEYAVNSIKTDVHSPGNFRII
GTLQNSAEFSEAFHCRKNSYMNPEKKCRVW
>hsa:4329
MAALLAAAAVRARILQVSSKVKSSPTWYSASSFSSSVPTVKLFIGGKFVESKSDKWIDIH
NPATNEVIGRVPQATKAEMDAAIASCKRAFPAWADTSVLSRQQVLLRYQQLIKENLKEIA
KLITLEQGKTLADAEGDVFRGLQVVEHACSVTSLMMGETMPSITKDMDLYSYRLPLGVCA
GIAPFNFPAMIPLWMFPMAMVCGNTFLMKPSERVPGATMLLAKLLQDSGAPDGTLNIIHG
QHEAVNFICDHPDIKAISFVGSNKAGEYIFERGSRHGKRVQANMGAKNHGVVMPDANKEN
TLNQLVGAAFGAAGQRCMALSTAVLVGEAKKWLPELVEHAKNLRVNAGDQPGADLGPLIT
PQAKERVCNLIDSGTKEGASILLDGRKIKVKGYENGNFVGPTIISNVKPNMTCYKEEIFG
PVLVVLETETLDEAIQIVNNNPYGNGTAIFTTNGATARKYAHLVDVGQVGVNVPIPVPLP
MFSFTGSRSSFRGDTNFYGKQGIQFYTQLKTITSQWKEEDATLSSPAVVMPTMGR
>hsa:4353
MGVPFFSSLRCMVDLGPCWAGGLTAEMKLLLALAGLLAILATPQPSEGAAPAVLGEVDTS
LVLSSMEEAKQLVDKAYKERRESIKQRLRSGSASPMELLSYFKQPVAATRTAVRAADYLH
VALDLLERKLRSLWRRPFNVTDVLTPAQLNVLSKSSGCAYQDVGVTCPEQDKYRTITGMC
NNRRSPTLGASNRAFVRWLPAEYEDGFSLPYGWTPGVKRNGFPVALARAVSNEIVRFPTD
QLTPDQERSLMFMQWGQLLDHDLDFTPEPAARASFVTGVNCETSCVQQPPCFPLKIPPND
PRIKNQADCIPFFRSCPACPGSNITIRNQINALTSFVDASMVYGSEEPLARNLRNMSNQL
GLLAVNQRFQDNGRALLPFDNLHDDPCLLTNRSARIPCFLAGDTRSSEMPELTSMHTLLL
REHNRLATELKSLNPRWDGERLYQEARKIVGAMVQIITYRDYLPLVLGPTAMRKYLPTYR

SYNDSVDPRIANVFTNAFRYGHTLIQPFMFRLDNRYQPMEPNPRVPLSRVFFASWRVVLE
GGIDPILRGLMATPAKLNRQNQIAVDEIRERLFEQVMRIGLDLPALNMQRSRDHGLPGYN
AWRRFCGLPQPETVGQLGTVLRNLKLARKLMEQYGTPNNIDIWMGGVSEPLKRKGRVGPL
LACIIGTQFRKLRDGDRFWWENEGVFSMQQRQALAQISLPRIICDNTGITTVSKNNIFMS
NSYPRDFVNCSTLPALNLASWREAS
>hsa:444
MAEDKETKHGGHKNGRKGGLSGTSFFTWFMVIALLGVWTSVAVVWFDLVDYEEVLGKLGI
YDADGDGDFDVDDAKVLLGLKERSTSEPAVPPEEAEPHTEPEEQVPVEAEPQNIEDEAKE
QIQSLLHEMVHAEHVEGEDLQQEDGPTGEPQQEDDEFLMATDVDDRFETLEPEVSHEETE
HSYHVEETVSQDCNQDMEEMMSEQENPDSSEPVVEDERLHHDTDDVTYQVYEEQAVYEPL
ENEGIEITEVTAPPEDNPVEDSQVIVEEVSIFPVEEQQEVPPETNRKTDDPEQKAKVKKK
KPKLLNKFDKTIKAELDAAEKLRKRGKIEEAVNAFKELVRKYPQSPRARYGKAQCEDDLA
EKRRSNEVLRGAIETYQEVASLPDVPADLLKLSLKRRSDRQQFLGHMRGSLLTLQRLVQL
FPNDTSLKNDLGVGYLLIGDNDNAKKVYEEVLSVTPNDGFAKVHYGFILKAQNKIAESIP
YLKEGIESGDPGTDDGRFYFHLGDAMQRVGNKEAYKWYELGHKRGHFASVWQRSLYNVNG
LKAQPWWTPKETGYTELVKSLERNWKLIRDEGLAVMDKAKGLFLPEDENLREKGDWSQFT
LWQQGRRNENACKGAPKTCTLLEKFPETTGCRRGQIKYSIMHPGTHVWPHTGPTNCRLRM
HLGLVIPKEGCKIRCANETKTWEEGKVLIFDDSFEHEVWQDASSFRLIFIVDVWHPELTP
QQRRSLPAI
>hsa:4482
MLSATRRACQLLLLHSLFPVPRMGNSASNIVSPQEALPGRKEQTPVAAKHHVNGNRTVEP
FPEGTQMAVFEKTGHAEVVRVVYQPEHMSFEELLKVFWENHDPTQGMRQGNDHGTQYRSA
IYPTSAKQMEAALSSKENYQKVLSEHGFGPITTDIREGQTFYYAEDYHQQYLSKNPNGYC
GLGGTGVSCPVGIKK
>hsa:4486
MELLPPLPQSFLLLLLLPAKPAAGEDWQCPRTPYAASRDFDVKYVVPSFSAGGLVQAMVT
YEGDRNESAVFVAIRNRLHVLGPDLKSVQSLATGPAGDPGCQTCAACGPGPHGPPGDTDT
KVLVLDPALPALVSCGSSLQGRCFLHDLEPQGTAVHLAAPACLFSAHHNRPDDCPDCVAS
PLGTRVTVVEQGQASYFYVASSLDAAVAASFSPRSVSIRRLKADASGFAPGFVALSVLPK
HLVSYSIEYVHSFHTGAFVYFLTVQPASVTDDPSALHTRLARLSATEPELGDYRELVLDC
RFAPKRRRRGAPEGGQPYPVLRVAHSAPVGAQLATELSIAEGQEVLFGVFVTGKDGGPGV
GPNSVVCAFPIDLLDTLIDEGVERCCESPVHPGLRRGLDFFQSPSFCPNPPGLEALSPNT
SCRHFPLLVSSSFSRVDLFNGLLGPVQVTALYVTRLDNVTVAHMGTMDGRILQVELVRSL
NYLLYVSNFSLGDSGQPVQRDVSRLGDHLLFASGDQVFQVPIQGPGCRHFLTCGRCLRAW
HFMGCGWCGNMCGQQKECPGSWQQDHCPPKLTEFHPHSGPLRGSTRLTLCGSNFYLHPSG
LVPEGTHQVTVGQSPCRPLPKDSSKLRPVPRKDFVEEFECELEPLGTQAVGPTNVSLTVT
NMPPGKHFRVDGTSVLRGFSFMEPVLIAVQPLFGPRAGGTCLTLEGQSLSVGTSRAVLVN
GTECLLARVSEGQLLCATPPGATVASVPLSLQVGGAQVPGSWTFQYREDPVVLSISPNCG
YINSHITICGQHLTSAWHLVLSFHDGLRAVESRCERQLPEQQLCRLPEYVVRDPQGWVAG
NLSARGDGAAGFTLPGFRFLPPPHPPSANLVPLKPEEHAIKFEVCVDGECHILGRVVRPG
PDGVPQSTLLGILLPLLLLVAALATALVFSYWWRRKQLVLPPNLNDLASLDQTAGATPLP
ILYSGSDYRSGLALPAIDGLDSTTCVHGASFSDSEDESCVPLLRKESIQLRDLDSALLAE
VKDVLIPHERVVTHSDRVIGKGHFGVVYHGEYIDQAQNRIQCAIKSLSRITEMQQVEAFL
REGLLMRGLNHPNVLALIGIMLPPEGLPHVLLPYMCHGDLLQFIRSPQRNPTVKDLISFG

LQVARGMEYLAEQKFVHRDLAARNCMLDESFTVKVADFGLARDILDREYYSVQQHRHARL
PVKWMALESLQTYRFTTKSDVWSFGVLLWELLTRGAPPYRHIDPFDLTHFLAQGRRLPQP
EYCPDSLYQVMQQCWEADPAVRPTFRVLVGEVEQIVSALLGDHYVQLPATYMNLGPSTSH
EMNVRPEQPQFSPMPGNVRRPRPLSEPPRPT
>hsa:4548
MSPALQDLSQPEGLKKTLRDEINAILQKRIMVLDGGMGTMIQREKLNEEHFRGQEFKDHA
RPLKGNNDILSITQPDVIYQIHKEYLLAGADIIETNTFSSTSIAQADYGLEHLAYRMNMC
SAGVARKAAEEVTLQTGIKRFVAGALGPTNKTLSVSPSVERPDYRNITFDELVEAYQEQA
KGLLDGGVDILLIETIFDTANAKAALFALQNLFEEKYAPRPIFISGTIVDKSGRTLSGQT
GEGFVISVSHGEPLCIGLNCALGAAEMRPFIEIIGKCTTAYVLCYPNAGLPNTFGDYDET
PSMMAKHLKDFAMDGLVNIVGGCCGSTPDHIREIAEAVKNCKPRVPPATAFEGHMLLSGL
EPFRIGPYTNFVNIGERCNVAGSRKFAKLIMAGNYEEALCVAKVQVEMGAQVLDVNMDDG
MLDGPSAMTRFCNLIASEPDIAKVPLCIDSSNFAVIEAGLKCCQGKCIVNSISLKEGEDD
FLEKARKIKKYGAAMVVMAFDEEGQATETDTKIRVCTRAYHLLVKKLGFNPNDIIFDPNI
LTIGTGMEEHNLYAINFIHATKVIKETLPGARISGGLSNLSFSFRGMEAIREAMHGVFLY
HAIKSGMDMGIVNAGNLPVYDDIHKELLQLCEDLIWNKDPEATEKLLRYAQTQGTGGKKV
IQTDEWRNGPVEERLEYALVKGIEKHIIEDTEEARLNQKKYPRPLNIIEGPLMNGMKIVG
DLFGAGKMFLPQVIKSARVMKKAVGHLIPFMEKEREETRVLNGTVEEEDPYQGTIVLATV
KGDVHDIGKNIVGVVLGCNNFRVIDLGVMTPCDKILKAALDHKADIIGLSGLITPSLDEM
IFVAKEMERLAIRIPLLIGGATTSKTHTAVKIAPRYSAPVIHVLDASKSVVVCSQLLDEN
LKDEYFEEIMEEYEDIRQDHYESLKERRYLPLSQARKSGFQMDWLSEPHPVKPTFIGTQV
FEDYDLQKLVDYIDWKPFFDVWQLRGKYPNRGFPKIFNDKTVGGEARKVYDDAHNMLNTL
ISQKKLRARGVVGFWPAQSIQDDIHLYAEAAVPQAAEPIATFYGLRQQAEKDSASTEPYY
CLSDFIAPLHSGIRDYLGLFAVACFGVEELSKAYEDDGDDYSSIMVKALGDRLAEAFAEE
LHERVRRELWAYCGSEQLDVADLRRLRYKGIRPAPGYPSQPDHTEKLTMWRLADIEQSTG
IRLTESLAMAPASAVSGLYFSNLKSKYFAVGKISKDQVEDYALRKNISVAEVEKWLGPIL
GYDTD
>hsa:4552
MRRFLLLYATQQGQAKAIAEEICEQAVVHGFSADLHCISESDKYDLKTETAPLVVVVSTT
GTGDPPDTARKFVKEIQNQTLPVDFFAHLRYGLLGLGDSEYTYFCNGGKIIDKRLQELGA
RHFYDTGHADDCVGLELVVEPWIAGLWPALRKHFRSSRGQEEISGALPVASPASSRTDLV
KSELLHIESQVELLRFDDSGRKDSEVLKQNAVNSNQSNVVIEDFESSLTRSVPPLSQASL
NIPGLPPEYLQVHLQESLGQEESQVSVTSADPVFQVPISKAVQLTTNDAIKTTLLVELDI
SNTDFSYQPGDAFSVICPNSDSEVQSLLQRLQLEDKREHCVLLKIKADTKKKGATLPQHI
PAGCSLQFIFTWCLEIRAIPKKAFLRALVDYTSDSAEKRRLQELCSKQGAADYSRFVRDA
CACLLDLLLAFPSCQPPLSLLLEHLPKLQPRPYSCASSSLFHPGKLHFVFNIVEFLSTAT
TEVLRKGVCTGWLALLVASVLQPNIHASHEDSGKALAPKISISPRTTNSFHLPDDPSIPI
IMVGPGTGIAPFIGFLQHREKLQEQHPDGNFGAMWLFFGCRHKDRDYLFRKELRHFLKHG
ILTHLKVSFSRDAPVGEEEAPAKYVQDNIQLHGQQVARILLQENGHIYVCGDAKNMAKDV
HDALVQIISKEVGVEKLEAMKTLATLKEEKRYLQDIWS
>hsa:4593
MRELVNIPLVHILTLVAFSGTEKLPKAPVITTPLETVDALVEEVATFMCAVESYPQPEIS
WTRNKILIKLFDTRYSIRENGQLLTILSVEDSDDGIYCCTANNGVGGAVESCGALQVKMK
PKITRPPINVKIIEGLKAVLPCTTMGNPKPSVSWIKGDSPLRENSRIAVLESGSLRIHNV

QKEDAGQYRCVAKNSLGTAYSKVVKLEVEEESEPEQDTKVFARILRAPESHNVTFGSFVT
LHCTATGIPVPTITWIENGNAVSSGSIQESVKDRVIDSRLQLFITKPGLYTCIATNKHGE
KFSTAKAAATISIAEWREYCLAVKELFCAKEWLVMEEKTHRGLYRSEMHLLSVPECSKLP
SMHWDPTACARLPHLAFPPMTSSKPSVDIPNLPSSSSSSFSVSPTYSMTVIISIMSSFAI
FVLLTITTLYCCRRRKQWKNKKRESAAVTLTTLPSELLLDRLHPNPMYQRMPLLLNPKLL
SLEYPRNNIEYVRDIGEGAFGRVFQARAPGLLPYEPFTMVAVKMLKEEASADMQADFQRE
AALMAEFDNPNIVKLLGVCAVGKPMCLLFEYMAYGDLNEFLRSMSPHTVCSLSHSDLSMR
AQVSSPGPPPLSCAEQLCIARQVAAGMAYLSERKFVHRDLATRNCLVGENMVVKIADFGL
SRNIYSADYYKANENDAIPIRWMPPESIFYNRYTTESDVWAYGVVLWEIFSYGLQPYYGM
AHEEVIYYVRDGNILSCPENCPVELYNLMRLCWSKLPADRPSFTSIHRILERMCERAEGT
VSV
>hsa:4594
MLRAKNQLFLLSPHYLRQVKESSGSRLIQQRLLHQQQPLHPEWAALAKKQLKGKNPEDLI
WHTPEGISIKPLYSKRDTMDLPEELPGVKPFTRGPYPTMYTFRPWTIRQYAGFSTVEESN
KFYKDNIKAGQQGLSVAFDLATHRGYDSDNPRVRGDVGMAGVAIDTVEDTKILFDGIPLE
KMSVSMTMNGAVIPVLANFIVTGEEQGVPKEKLTGTIQNDILKEFMVRNTYIFPPEPSMK
IIADIFEYTAKHMPKFNSISISGYHMQEAGADAILELAYTLADGLEYSRTGLQAGLTIDE
FAPRLSFFWGIGMNFYMEIAKMRAGRRLWAHLIEKMFQPKNSKSLLLRAHCQTSGWSLTE
QDPYNNIVRTAIEAMAAVFGGTQSLHTNSFDEALGLPTVKSARIARNTQIIIQEESGIPK
VADPWGGSYMMECLTNDVYDAALKLINEIEEMGGMAKAVAEGIPKLRIEECAARRQARID
SGSEVIVGVNKYQLEKEDAVEVLAIDNTSVRNRQIEKLKKIKSSRDQALAERCLAALTEC
AASGDGNILALAVDASRARCTVGEITDALKKVFGEHKANDRMVSGAYRQEFGESKEITSA
IKRVHKFMEREGRRPRLLVAKMGQDGHDRGAKVIATGFADLGFDVDIGPLFQTPREVAQQ
AVDADVHAVGISTLAAGHKTLVPELIKELNSLGRPDILVMCGGVIPPQDYEFLFEVGVSN
VFGPGTRIPKAAVQVLDDIEKCLEKKQQSV
>hsa:4758
MTGERPSTALPDRRWGPRILGFWGGCRVWVFAAIFLLLSLAASWSKAENDFGLVQPLVTM
EQLLWVSGRQIGSVDTFRIPLITATPRGTLLAFAEARKMSSSDEGAKFIALRRSMDQGST
WSPTAFIVNDGDVPDGLNLGAVVSDVETGVVFLFYSLCAHKAGCQVASTMLVWSKDDGVS
WSTPRNLSLDIGTEVFAPGPGSGIQKQREPRKGRLIVCGHGTLERDGVFCLLSDDHGASW
RYGSGVSGIPYGQPKQENDFNPDECQPYELPDGSVVINARNQNNYHCHCRIVLRSYDACD
TLRPRDVTFDPELVDPVVAAGAVVTSSGIVFFSNPAHPEFRVNLTLRWSFSNGTSWRKET
VQLWPGPSGYSSLATLEGSMDGEEQAPQLYVLYEKGRNHYTESISVAKISVYGTL
>hsa:4759
MASLPVLQKESVFQSGAHAYRIPALLYLPGQQSLLAFAEQRASKKDEHAELIVLRRGDYD
APTHQVQWQAQEVVAQARLDGHRSMNPCPLYDAQTGTLFLFFIAIPGQVTEQQQLQTRAN
VTRLCQVTSTDHGRTWSSPRDLTDAAIGPAYREWSTFAVGPGHCLQLHDRARSLVVPAYA
YRKLHPIQRPIPSAFCFLSHDHGRTWARGHFVAQDTLECQVAEVETGEQRVVTLNARSHL
RARVQAQSTNDGLDFQESQLVKKLVEPPPQGCQGSVISFPSPRSGPGSPAQWLLYTHPTH
SWQRADLGAYLNPRPPAPEAWSEPVLLAKGSCAYSDLQSMGTGPDGSPLFGCLYEANDYE
EIVFLMFTLKQAFPAEYLPQ
>hsa:476
MGKGVGRDKYEPAAVSEQGDKKGKKGKKDRDMDELKKEVSMDDHKLSLDELHRKYGTDLS
RGLTSARAAEILARDGPNALTPPPTTPEWIKFCRQLFGGFSMLLWIGAILCFLAYSIQAA

TEEEPQNDNLYLGVVLSAVVIITGCFSYYQEAKSSKIMESFKNMVPQQALVIRNGEKMSI
NAEEVVVGDLVEVKGGDRIPADLRIISANGCKVDNSSLTGESEPQTRSPDFTNENPLETR
NIAFFSTNCVEGTARGIVVYTGDRTVMGRIATLASGLEGGQTPIAAEIEHFIHIITGVAV
FLGVSFFILSLILEYTWLEAVIFLIGIIVANVPEGLLATVTVCLTLTAKRMARKNCLVKN
LEAVETLGSTSTICSDKTGTLTQNRMTVAHMWFDNQIHEADTTENQSGVSFDKTSATWLA
LSRIAGLCNRAVFQANQENLPILKRAVAGDASESALLKCIELCCGSVKEMRERYAKIVEI
PFNSTNKYQLSIHKNPNTSEPQHLLVMKGAPERILDRCSSILLHGKEQPLDEELKDAFQN
AYLELGGLGERVLGFCHLFLPDEQFPEGFQFDTDDVNFPIDNLCFVGLISMIDPPRAAVP
DAVGKCRSAGIKVIMVTGDHPITAKAIAKGVGIISEGNETVEDIAARLNIPVSQVNPRDA
KACVVHGSDLKDMTSEQLDDILKYHTEIVFARTSPQQKLIIVEGCQRQGAIVAVTGDGVN
DSPALKKADIGVAMGIAGSDVSKQAADMILLDDNFASIVTGVEEGRLIFDNLKKSIAYTL
TSNIPEITPFLIFIIANIPLPLGTVTILCIDLGTDMVPAISLAYEQAESDIMKRQPRNPK
TDKLVNERLISMAYGQIGMIQALGGFFTYFVILAENGFLPIHLLGLRVDWDDRWINDVED
SYGQQWTYEQRKIVEFTCHTAFFVSIVVVQWADLVICKTRRNSVFQQGMKNKILIFGLFE
ETALAAFLSYCPGMGVALRMYPLKPTWWFCAFPYSLLIFVYDEVRKLIIRRRPGGWVEKE
TYY

>hsa:4835

MAGKKVLIVYAHQEPKSFNGSLKNVAVDELSRQGCTVTVSDLYAMNLEPRATDKDITGTL
SNPEVFNYGVETHEAYKQRSLASDITDEQKKVREADLVIFQFPLYWFSVPAILKGWMDRV
LCQGFAFDIPGFYDSGLLQGKLALLSVTTGGTAEMYTKTGVNGDSRYFLWPLQHGTLHFC
GFKVLAPQISFAPEIASEEERKGMVAAWSQRLQTIWKEEPIPCTAHWHFGQ

>hsa:4837

MESGFTSKDTYLSHFNPRDYLEKYYKFGSRHSAESQILKHLLKNLFKIFCLDGVKGDLLI
DIGSGPTIYQLLSACESFKEIVVTDYSDQNLQELEKWLKKEPEAFDWSPVVTYVCDLEGN
RVKGPEKEEKLRQAVKQVLKCDVTQSQPLGAVPLPPADCVLSTLCLDAACPDLPTYCRAL
RNLGSLLKPGGFLVIMDALKSSYYMIGEQKFSSLPLGREAVEAAVKEAGYTIEWFEVISQ
SYSSTMANNEGLFSLVARKLSRPL

>hsa:4860

MENGYTYEDYKNTAEWLLSHTKHRPQVAIICGSGLGGLTDKLTQAQIFDYGEIPNFPRST
VPGHAGRLVFGFLNGRACVMMQGRFHMYEGYPLWKVTFPVRVFHLLGVDTLVVTNAAGGL
NPKFEVGDIMLIRDHINLPGFSGQNPLRGPNDERFGDRFPAMSDAYDRTMRQRALSTWKQ
MGEQRELQEGTYVMVAGPSFETVAECRVLQKLGADAVGMSTVPEVIVARHCGLRVFGFSL
ITNKVIMDYESLEKANHEEVLAAGKQAAQKLEQFVSILMASIPLPDKAS

>hsa:4881

MPGPRRPAGSRLRLLLLLLLPPLLLLLRGSHAGNLTVAVVLPLANTSYPWSWARVGPAVE
LALAQVKARPDLLPGWTVRTVLGSSENALGVCSDTAAPLAAVDLKWEHNPAVFLGPGCVY
AAAPVGRFTAHWRVPLLTAGAPALGFGVKDEYALTTRAGPSYAKLGDFVAALHRRLGWER
QALMLYAYRPGDEEHCFFLVEGLFMRVRDRLNITVDHLEFAEDDLSHYTRLLRTMPRKGR
VIYICSSPDAFRTLMLLALEAGLCGEDYVFFHLDIFGQSLQGGQGPAPRRPWERGDGQDV
SARQAFQAAKIITYKDPDNPEYLEFLKQLKHLAYEQFNFTMEDGLVNTIPASFHDGLLLY
IQAVTETLAHGGTVTDGENITQRMWNRSFQGVTGYLKIDSSGDRETDFSLWDMDPENGAF
RVVLNYNGTSQELVAVSGRKLNWPLGYPPPDIPKCGFDNEDPACNQDHLSTLEVLALVGS
LSLLGILIVSFFIYRKMQLEKELASELWRVRWEDVEPSSLERHLRSAGSRLTLSGRGSNY
GSLLTTEGQFQVFAKTAYYKGNLVAVKRVNRKRIELTRKVLFELKHMRDVQNEHLTRFVG

ACTDPPNICILTEYCPRGSLQDILENESITLDWMFRYSLTNDIVKGMLFLHNGAICSHGN
LKSSNCVVDGRFVLKITDYGLESFRDLDPEQGHTVYAKKLWTAPELLRMASPPVRGSQAG
DVYSFGIILQEIALRSGVFHVEGLDLSPKEIIERVTRGEQPPFRPSLALQSHLEELGLLM
QRCWAEDPQERPPFQQIRLTLRKFNRENSSNILDNLLSRMEQYANNLEELVEERTQAYLE
EKRKAEALLYQILPHSVAEQLKRGETVQAEAFDSVTIYFSDIVGFTALSAESTPMQVVTL
LNDLYTCFDAVIDNFDVYKVETIGDAYMVVSGLPVRNGRLHACEVARMALALLDAVRSFR
IRHRPQEQLRLRIGIHTGPVCAGVVGLKMPRYCLFGDTVNTASRMESNGEALKIHLSSET
KAVLEEFGGFELELRGDVEMKGKGKVRTYWLLGERGSSTRG
>hsa:49
MVEMLPTAILLVLAVSVVAKDNATCDGPCGLRFRQNPQGGVRIVGGKAAQHGAWPWMVSL
QIFTYNSHRYHTCGGSLLNSRWVLTAAHCFVGKNNVHDWRLVFGAKEITYGNNKPVKAPL
QERYVEKIIIHEKYNSATEGNDIALVEITPPISCGRFIGPGCLPHFKAGLPRGSQSCWVA
GWGYIEEKAPRPSSILMEARVDLIDLDLCNSTQWYNGRVQPTNVCAGYPVGKIDTCQGDS
GGPLMCKDSKESAYVVVGITSWGVGCARAKRPGIYTATWPYLNWIASKIGSNALRMIQSA
TPPPPTTRPPPIRPPFSHPISAHLPWYFQPPPRPLPPRPPAAQPRPPPSPPPPPPPPASP
LPPPPPPPPPTPSSTTKLPQGLSFAKRLQQLIEVLKGKTYSDGKNHYDMETTELPELTST
S
>hsa:4907
MCPRAARAPATLLLALGAVLWPAAGAWELTILHTNDVHSRLEQTSEDSSKCVNASRCMGG
VARLFTKVQQIRRAEPNVLLLDAGDQYQGTIWFTVYKGAEVAHFMNALRYDAMALGNHEF
DNGVEGLIEPLLKEAKFPILSANIKAKGPLASQISGLYLPYKVLPVGDEVVGIVGYTSKE
TPFLSNPGTNLVFEDEITALQPEVDKLKTLNVNKIIALGHSGFEMDKLIAQKVRGVDVVV
GGHSNTFLYTGNPPSKEVPAGKYPFIVTSDDGRKVPVVQAYAFGKYLGYLKIEFDERGNV
ISSHGNPILLNSSIPEDPSIKADINKWRIKLDNYSTQELGKTIVYLDGSSQSCRFRECNM
GNLICDAMINNNLRHTDEMFWNHVSMCILNGGGIRSPIDERNNGIHVVYDLSRKPGDRVV
KLDVLCTKCRVPSYDPLKMDEVYKVILPNFLANGGDGFQMIKDELLRHDSGDQDINVVST
YISKMKVIYPAVEGRIKFSTGSHCHGSFSLIFLSLWAVIFVLYQ
>hsa:4914
MKEAALICLAPSVPPILTVKSWDTMQLRAARSRCTNLLAASYIENQQHLQHLELRDLRGL
GELRNLTIVKSGLRFVAPDAFHFTPRLSRLNLSFNALESLSWKTVQGLSLQELVLSGNPL
HCSCALRWLQRWEEEGLGGVPEQKLQCHGQGPLAHMPNASCGVPTLKVQVPNASVDVGDD
VLLRCQVEGRGLEQAGWILTELEQSATVMKSGGLPSLGLTLANVTSDLNRKNVTCWAEND
VGRAEVSVQVNVSFPASVQLHTAVEMHHWCIPFSVDGQPAPSLRWLFNGSVLNETSFIFT
EFLEPAANETVRHGCLRLNQPTHVNNGNYTLLAANPFGQASASIMAAFMDNPFEFNPEDP
IPDTNSTSGDPVEKKDETPFGVSVAVGLAVFACLFLSTLLLVLNKCGRRNKFGINRPAVL
APEDGLAMSLHFMTLGGSSLSPTEGKGSGLQGHIIENPQYFSDACVHHIKRRDIVLKWEL
GEGAFGKVFLAECHNLLPEQDKMLVAVKALKEASESARQDFQREAELLTMLQHQHIVRFF
GVCTEGRPLLMVFEYMRHGDLNRFLRSHGPDAKLLAGGEDVAPGPLGLGQLLAVASQVAA
GMVYLAGLHFVHRDLATRNCLVGQGLVVKIGDFGMSRDIYSTDYYRVGGRTMLPIRWMPP
ESILYRKFTTESDVWSFGVVLWEIFTYGKQPWYQLSNTEAIDCITQGRELERPRACPPEV
YAIMRGCWQREPQQRHSIKDVHARLQALAQAPPVYLDVLG
>hsa:4915
MSSWIRWHGPAMARLWGFCWLVVGFWRAAFACPTSCKCSASRIWCSDPSPGIVAFPRLEP
NSVDPENITEIFIANQKRLEIINEDDVEAYVGLRNLTIVDSGLKFVAHKAFLKNSNLQHI

NFTRNKLTSLSRKHFRHLDLSELILVGNPFTCSCDIMWIKTLQEAKSSPDTQDLYCLNES
SKNIPLANLQIPNCGLPSANLAAPNLTVEEGKSITLSCSVAGDPVPNMYWDVGNLVSKHM
NETSHTQGSLRITNISSDDSGKQISCVAENLVGEDQDSVNLTVHFAPTITFLESPTSDHH
WCIPFTVKGNPKPALQWFYNGAILNESKYICTKIHVTNHTEYHGCLQLDNPTHMNNGDYT
LIAKNEYGKDEKQISAHFMGWPGIDDGANPNYPDVIYEDYGTAANDIGDTTNRSNEIPST
DVTDKTGREHLSVYAVVVIASVVGFCLLVMLFLLKLARHSKFGMKGFVLFHKIPLDG
>hsa:4916
MDVSLCPAKCSFWRIFLLGSVWLDYVGSVLACPANCVCSKTEINCRRPDDGNLFPLLEGQ
DSGNSNGNASINITDISRNITSIHIENWRSLHTLNAVDMELYTGLQKLTIKNSGLRSIQP
RAFAKNPHLRYINLSSNRLTTLSWQLFQTLSLRELQLEQNFFNCSCDIRWMQLWQEQGEA
KLNSQNLYCINADGSQLPLFRMNISQCDLPEISVSHVNLTVREGDNAVITCNGSGSPLPD
VDWIVTGLQSINTHQTNLNWTNVHAINLTLVNVTSEDNGFTLTCIAENVVGMSNASVALT
VYYPPRVVSLEEPELRLEHCIEFVVRGNPPPTLHWLHNGQPLRESKIIHVEYYQEGEISE
GCLLFNKPTHYNNGNYTLIAKNPLGTANQTINGHFLKEPFPESTDNFILFDEVSPTPPIT
VTHKPEEDTFGVSIAVGLAAFACVLLVVLFVMINKYGRRSKFGMKGPVAVISGEEDSASP
LHHINHGITTPSSLDAGPDTVVIGMTRIPVIENPQYFRQGHNCHKPDTWVFSNIDNHGIL
NLKDNRDHLVPSTHYIYEEPEVQSGEVSYPRSHGFREIMLNPISLPGHSKPLNHGIYVED
VNVYFSKGRHGF
>hsa:4919
MHRPRRRGTRPPLLALLAALLLAARGAAAQETELSVSAELVPTSSWNISSELNKDSYLTL
DEPMNNITTSLGQTAELHCKVSGNPPPTIRWFKNDAPVVQEPRRLSFRSTIYGSRLRIRN
LDTTDTGYFQCVATNGKEVVSSTGVLFVKFGPPPTASPGYSDEYEEDGFCQPYRGIACAR
FIGNRTVYMESLHMQGEIENQITAAFTMIGTSSHLSDKCSQFAIPSLCHYAFPYCDETSS
VPKPRDLCRDECEILENVLCQTEYIFARSNPMILMRLKLPNCEDLPQPESPEAANCIRIG
IPMADPINKNHKCYNSTGVDYRGTVSVTKSGRQCQPWNSQYPHTHTFTALRFPELNGGHS
YCRNPGNQKEAPWCFTLDENFKSDLCDIPACGK
>hsa:4920
MARGSALPRRPLLCIPAVWAAAALLLSVSRTSGEVEVLDPNDPLGPLDGQDGPIPTLKGY
FLNFLEPVNNITIVQGQTAILHCKVAGNPPPNVRWLKNDAPVVQEPRRIIIRKTEYGSRL
RIQDLDTTDTGYYQCVATNGMKTITATGVLFVRLGPTHSPNHNFQDDYHEDGFCQPYRGI
ACARFIGNRTIYVDSLQMQGEIENRITAAFTMIGTSTHLSDQCSQFAIPSFCHFVFPLCD
ARSRTPKPRELCRDECEVLESDLCRQEYTIARSNPLILMRLQLPKCEALPMPESPDAANC
MRIGIPAERLGRYHQCYNGSGMDYRGTASTTKSGHQCQPWALQHPHSHHLSSTDFPELGG
GHAYCRNPGGQMEGPWCFTQNKNVRMELCDVPSCSPRDSSKMGILYILVPSIAIPLVIAC
LFFLVCMCRNKQKASASTPQRRQLMASPSQDMEMPLINQHKQAKLKEISLSAVRFMEELG
EDRFGKVYKGHLFGPAPGEQTQAVAIKTLKDKAEGPLREEFRHEAMLRARLQHPNVVCLL
GVVTKDQPLSMIFSYCSHGDLHEFLVMRSPHSDVGSTDDDRTVKSALEPPDFVHLVAQIA
AGMEYLSSHHVVHKDLATRNVLVYDKLNVKISDLGLFREVYAADYYKLLGNSLLPIRWMA
PEAIMYGKFSIDSDIWSYGVVLWEVFSYGLQPYCGYSNQDVVEMIRNRQVLPCPDDCPAW
VYALMIECWNEFPSRRPRFKDIHSRLRAWGNLSNYNSSAQTSGASNTTQTSSLSTSPVSN
VSNARYVGPKQKAPPFPQPQFIPMKGQIRPMVPPPQLYVPVNGYQPVPAYGAYLPNFYPV
QIPMQMAPQQVPPQMVPKPSSHHSGSGSTSTGYVTTAPSNTSMADRAALLSEGADDTQNA
PEDGAQSTVQEAEEEEEGSVPETELLGDCDTLQVDEAQVQLEA
>hsa:4921

MILIPRMLLVLFLLLPILSSAKAQVNPAICRYPLGMSGGQIPDEDITASSQWSESTAAKY
GRLDSEEGDGAWCPEIPVEPDDLKEFLQIDLHTLHFITLVGTQGRHAGGHGIEFAPMYKI
NYSRDGTRWISWRNRHGKQVLDGNSNPYDIFLKDLEPPIVARFVRFIPVTDHSMNVCMRV
ELYGCVWLDGLVSYNAPAGQQFVLPGGSIIYLNDSVYDGAVGYSMTEGLGQLTDGVSGLD
DFTQTHEYHVWPGYDYVGWRNESATNGYIEIMFEFDRIRNFTTMKVHCNNMFAKGVKIFK
EVQCYFRSEASEWEPNAISFPLVLDDVNPSARFVTVPLHHRMASAIKCQYHFADTWMMFS
EITFQSDAAMYNNSEALPTSPMAPTTYDPMLKVDDSNTRILIGCLVAIIFILLAIIVIIL
WRQFWQKMLEKASRRMLDDEMTVSLSLPSDSSMFNNNRSSSPSEQGSNSTYDRIFPLRPD
YQEPSRLIRKLPEFAPGEEESGCSGVVKPVQPSGPEGVPHYAEADIVNLQGVTGGNTYSV
PAVTMDLLSGKDVAVEEFPRKLLTFKEKLGEGQFGEVHLCEVEGMEKFKDKDFALDVSAN
QPVLVAVKMLRADANKNARNDFLKEIKIMSRLKDPNIIHLLAVCITDDPLCMITEYMENG
DLNQFLSRHEPPNSSSSDVRTVSYTNLKFMATQIASGMKYLSSLNFVHRDLATRNCLVGK
NYTIKIADFGMSRNLYSGDYYRIQGRAVLPIRWMSWESILLGKFTTASDVWAFGVTLWET
FTFCQEQPYSQLSDEQVIENTGEFFRDQGRQTYLPQPAICPDSVYKLMLSCWRRDTKNRP
SFQEIHLLLLQQGDE

>hsa:4942

MFSKLAHLQRFAVLSRGVHSSVASATSVATKKTVQGPPTSDDIFEREYKYGAHNYHPLPV
ALERGKGIYLWDVEGRKYFDFLSSYSAVNQGHCHPKIVNALKSQVDKLTLTSRAFYNNVL
GEYEEYITKLFNYHKVLPMNTGVEAGETACKLARKWGYTVKGIQKYKAKIVFAAGNFWGR
TLSAISSSTDPTSYDGFGPFMPGFDIIPYNDLPALERALQDPNVAAFMVEPIQGEAGVVV
PDPGYLMGVRELCTRHQVLFIADEIQTGLARTGRWLAVDYENVRPDIVLLGKALSGGLYP
VSAVLCDDDIMLTIKPGEHGSTYGGNPLGCRVAIAALEVLEEENLAENADKLGIILRNEL
MKLPSDVVTAVRGKGLLNAIVIKETKDWDAWKVCLRLRDNGLLAKPTHGDIIRFAPPLVI
KEDELRESIEIINKTILSF

>hsa:495

MGKAENYELYSVELGPGPGGDMAAKMSKKKKAGGGGGKRKEKLENMKKEMEINDHQLSVA
ELEQKYQTSATKGLSASLAAELLLRDGPNALRPPRGTPEYVKFARQLAGGLQCLMWVAAA
ICLIAFAIQASEGDLTTDDNLYLAIALIAVVVVTGCFGYYQEFKSTNIIASFKNLVPQQA
TVIRDGDKFQINADQLVVGDLVEMKGGDRVPADIRILAAQGCKVDNSSLTGESEPQTRSP
ECTHESPLETRNIAFFSTMCLEGTVQGLVVNTGDRTIIGRIASLASGVENEKTPIAIEIE
HFVDIIAGLAILFGATFFIVAMCIGYTFLRAMVFFMAIVVAYVPEGLLATVTVCLSLTAK
RLASKNCVVKNLEAVETLGSTSVICSDKTGTLTQNRMTVSHLWFDNHIHTADTTEDQSGQ
TFDQSSETWRALCRVLTLCNRAAFKSGQDAVPVPKRIVIGDASETALLKFSELTLGNAMG
YRDRFPKVCEIPFNSTNKFQLSIHTLEDPRDPRHLLVMKGAPERVLERCSSILIKGQELP
LDEQWREAFQTAYLSLGGLGERVLGFCQLYLNEKDYPPGYAFDVEAMNFPSSGLCFAGLV
SMIDPPRATVPDAVLKCRTAGIRVIMVTGDHPITAKAIAASVGIISEGSETVEDIAARLR
VPVDQVNRKDARACVINGMQLKDMDPSELVEALRTHPEMVFARTSPQQKLVIVESCQRLG
AIVAVTGDGVNDSPALKKADIGVAMGIAGSDAAKNAADMILLDDNFASIVTGVEQGRLIF
DNLKKSIAYTLTKNIPELTPYLIYITVSVPLPLGCITILFIELCTDIFPSVSLAYEKAES
DIMHLRPRNPKRDRLVNEPLAAYSYFQIGAIQSFAGFTDYFTAMAQEGWFPLLCVGLRAQ
WEDHHLQDLQDSYGQEWTFGQRLYQQYTCYTVFFISIEVCQIADVLIRKTRRLSAFQQGF
FRNKILVIAIVFQVCIGCFLCYCPGMPNIFNFMPIRFQWWLVPLPYGILIFVYDEIRKLG
VRCCPGSWWDQELYY

>hsa:4953

MNNFGNEEFDCHFLDEGFTAKDILDQKINEVSSSDDKDAFYVADLGDILKKHLRWLKALP
RVTPFYAVKCNDSKAIVKTLAATGTGFDCASKTEIQLVQSLGVPPERIIYANPCKQVSQI
KYAANNGVQMMTFDSEVELMKVARAHPKAKLVLRIATDDSKAVCRLSVKFGATLRTSRLL
LERAKELNIDVVGVSFHVGSGCTDPETFVQAISDARCVFDMGAEVGFSMYLLDIGGGFPG
SEDVKLKFEEITGVINPALDKYFPSDSGVRIIAEPGRYYVASAFTLAVNIIAKKIVLKEQ
TGSDDEDESSEQTFMYYVNDGVYGSFNCILYDHAHVKPLLQKRPKPDEKYYSSSIWGPTC
DGLDRIVERCDLPEMHVGDWMLFENMGAYTVAAASTFNGFQRPTIYYVMSGPAWQLMQQF
QNPDFPPEVEEQDASTLPVSCAWESGMKRHRAACASASINV
>hsa:50
MAPYSLLVTRLQKALGVRQYHVASVLCQRAKVAMSHFEPNEYIHYDLLEKNINIVRKRLN
RPLTLSEKIVYGHLDDPASQEIERGKSYLRLRPDRVAMQDATAQMAMLQFISSGLSKVAV
PSTIHCDHLIEAQVGGEKDLRRAKDINQEVYNFLATAGAKYGVGFWKPGSGIIHQIILEN
YAYPGVLLIGTDSHTPNGGGLGGICIGVGGADAVDVMAGIPWELKCPKVIGVKLTGSLSG
WSSPKDVILKVAGILTVKGGTGAIVEYHGPGVDSISCTGMATICNMGAEIGATTSVFPYN
HRMKKYLSKTGREDIANLADEFKDHLVPDPGCHYDQLIEINLSELKPHINGPFTPDLAHP
VAEVGKVAEKEGWPLDIRVGLIGSCTNSSYEDMGRSAAVAKQALAHGLKCKSQFTITPGS
EQIRATIERDGYAQILRDLGGIVLANACGPCIGQWDRKDIKKGEKNTIVTSYNRNFTGRN
DANPETHAFVTSPEIVTALAIAGTLKFNPETDYLTGTDGKKFRLEAPDADELPKGEFDPG
QDTYQHPPKDSSGQHVDVSPTSQRLQLLEPFDKWDGKDLEDLQILIKVKGKCTTDHISAA
GPWLKFRGHLDNISNNLLIGAINIENGKANSVRNAVTQEFGPVPDTARYYKKHGIRWVVI
GDENYGEGSSREHAALEPRHLGGRAIITKSFARIHETNLKKQGLLPLTFADPADYNKIHP
VDKLTIQGLKDFTPGKPLKCIIKHPNGTQETILLNHTFNETQIEWFRAGSALNRMKELQQ
>hsa:501
MWRLPRALCVHAAKTSKLSGPWSRPAAFMSTLLINQPQYAWLKELGLREENEGVYNGSWG
GRGEVITTYCPANNEPIARVRQASVADYEETVKKAREAWKIWADIPAPKRGEIVRQIGDA
LREKIQVLGSLVSLEMGKILVEGVGEVQEYVDICDYAVGLSRMIGGPILPSERSGHALIE
QWNPVGLVGIITAFNFPVAVYGWNNAIAMICGNVCLWKGAPTTSLISVAVTKIIAKVLED
NKLPGAICSLTCGGADIGTAMAKDERVNLLSFTGSTQVGKQVGLMVQERFGRSLLELGGN
NAIIAFEDADLSLVVPSALFAAVGTAGQRCTTARRLFIHESIHDEVVNRLKKAYAQIRVG
NPWDPNVLYGPLHTKQAVSMFLGAVEEAKKEGGTVVYGGKVMDRPGNYVEPTIVTGLGHD
ASIAHTETFAPILYVFKFKNEEEVFAWNNEVKQGLSSSIFTKDLGRIFRWLGPKGSDCGI
VNVNIPTSGAEIGGAFGGEKHTGGGRESGSDAWKQYMRRSTCTINYSKDLPLAQGIKFQ
>hsa:5033
MIWYILIIGILLPQSLAHPGFFTSIGQMTDLIHTEKDLVTSLKDYIKAEEDKLEQIKKWA
EKLDRLTSTATKDPEGFVGHPVNAFKLMKRLNTEWSELENLVLKDMSDGFISNLTIQRQY
FPNDEDQVGAAKALLRLQDTYNLDTDTISKGNLPGVKHKSFLTAEDCFELGKVAYTEADY
YHTELWMEQALRQLDEGEISTIDKVSVLDYLSYAVYQQGDLDKALLLTKKLLELDPEHQR
ANGNLKYFEYIMAKEKDVNKSASDDQSDQKTTPKKKGVAVDYLPERQKYEMLCRGEGIKM
TPRRQKKLFCRYHDGNRNPKFILAPAKQEDEWDKPRIIRFHDIISDAEIEIVKDLAKPRL
SRATVHDPETGKLTTAQYRVSKSAWLSGYENPVVSRINMRIQDLTGLDVSTAEELQVANY
GVGGQYEPHFDFARKDEPDAFKELGTGNRIATWLFYMSDVSAGGATVFPEVGASVWPKKG
TAVFWYNLFASGEGDYSTRHAACPVLVGNKWVSNKWLHERGQEFRRPCTLSELE
>hsa:5045
MELRPWLLWVVAATGTLVLLAADAQGQKVFTNTWAVRIPGGPAVANSVARKHGFLNLGQI

FGDYYHFWHRGVTKRSLSPHRPRHSRLQREPQVQWLEQQVAKRRTKRDVYQEPTDPKFPQ
QWYLSGVTQRDLNVKAAWAQGYTGHGIVVSILDDGIEKNHPDLAGNYDPGASFDVNDQDP
DPQPRYTQMNDNRHGTRCAGEVAAVANNGVCGVGVAYNARIGGVRMLDGEVTDAVEARSL
GLNPNHIHIYSASWGPEDDGKTVDGPARLAEEAFFRGVSQGRGGLGSIFVWASGNGGREH
DSCNCDGYTNSIYTLSISSATQFGNVPWYSEACSSTLATTYSSGNQNEKQIVTTDLRQKC
TESHTGTSASAPLAAGIIALTLEANKNLTWRDMQHLVVQTSKPAHLNANDWATNGVGRKV
SHSYGYGLLDAGAMVALAQNWTTVAPQRKCIIDILTEPKDIGKRLEVRKTVTACLGEPNH
ITRLEHAQARLTLSYNRRGDLAIHLVSPMGTRSTLLAARPHDYSADGFNDWAFMTTHSWD
EDPSGEWVLEIENTSEANNYGTLTKFTLVLYGTAPEGLPVPPESSGCKTLTSSQACVVCE
EGFSLHQKSCVQHCPPGFAPQVLDTHYSTENDVETIRASVCAPCHASCATCQGPALTDCL
SCPSHASLDPVEQTCSRQSQSSRESPPQQQPPRLPPEVEAGQRLRAGLLPSHLPEVVAGL
SCAFIVLVFVTVFLVLQLRSGFSFRGVKVYTMDRGLISYKGLPPEAWQEECPSDSEEDEG
RGERTAFIKDQSAL
>hsa:50484
MLLLRLPPHRSHASPLDCKLQDRCRKCYSPRSGQACPPALAAAWLRRCERRGGRPRGGRR
KELTLGLRPARCSAPGPAKDDAWRPQAGRSSSDTNESEIKSNEEPLLRKSSRRFVIFPIQ
YPDIWKMYKQAQASFWTAEEVDLSKDLPHWNKLKADEKYFISHILAFFAASDGIVNENLV
ERFSQEVQVPEARCFYGFQILIENVHSEMYSLLIDTYIRDPKKREFLFNAIETMPYVKKK
ADWALRWIADRKSTFGERVVAFAAVEGVFFSGSFAAIFWLKKRGLMPGLTFSNELISRDE
GLHCDFACLMFQYLVNKPSEERVREIIVDAVKIEQEFLTEALPVGLIGMNCILMKQYIEF
VADRLLVELGFSKVFQAENPFDFMENISLEGKTNFFEKRVSEYQRFAVMAETTDNVFTLD
ADF
>hsa:50487
MGVQAGLFGMLGFLGVALGGSPALRWYRTSCHLTKAVPGNPLGYLSFLAKDAQGLALIHA
RWDAHRRLQSCSWEDEPELTAAYGALCAHETAWGSFIHTPGPELQRALATLQSQWEACRA
LEESPAGARKKRAAGQSGVPGGGHQREKRGWTMPGTLWCGVGDSAGNSSELGVFQGPDLC
CREHDRCPQNISPLQYNYGIRNYRFHTISHCDCDTRFQQCLQNQHDSISDIVGVAFFNVL
EIPCFVLEEQEACVAWYWWGGCRMYGTVPLARLQPRTFYNASWSSRATSPTPSSRSPAPP
KPRQKQHLRKGPPHQKGSKRPSKANTTALQDPMVSPRLDVAPTGLQGPQGGLKPQGARWV
CRSFRRHLDQCEHQIGPREIEFQLLNSAQEPLFHCNCTRRLARFLRLHSPPEVTNMLWEL
LGTTCFKLAPPLDCVEGKNCSRDPRAIRVSARHLRRLQQRRHQLQDKGTDERQPWPSEPL
RGPMSFYNQCLQLTQAARRPDRQQKSWSQ
>hsa:5049
MSQGDSNPAAIPHAAEDIQGDDRWMSQHNRFVLDCKDKEPDVLFVGDSMVQLMQQYEIWR
ELFSPLHALNFGIGGDTTRHVLWRLKNGELENIKPKVIVVWVGTNNHENTAEEVAGGIEA
IVQLINTRQPQAKIIVLGKAAASKYSISEIVRLEQGSVNWSIGTYPDDTPATTRPAILQL
FTGKMSRITMKEKSRWTEEILH
>hsa:5050
MSGEENPASKPTPVQDVQGDGRWMSLHHRFVADSKDKEPEVVFIGDSLVQLMHQCEIWRE
LFSPLHALNFGIGGDGTQHVLWRLENGELEHIRPKIVVVWVGTNNHGHTAEQVTGGIKAI
VQLVNERQPQARVVVLGLLPRGQHPNPLREKNRQVNELVRAALAGHPRAHFLDADPGFVH
SDGTISHHDMYDYLHLSRLGYTPVCRALHSLLLRLLAQDQGQGAPLLEPAP
>hsa:5051
MGVNQSVGFPPVTGPHLVGCGDVMEGQNLQGSFFRLFYPCQKAEETMEQPLWIPRYEYCT

GLAEYLQFNKRCGGLLFNLAVGSCRLPVSWNGPFKTKDSGYPLIIFSHGLGAFRTLYSAF
CMELASRGFVVAVPEHRDRSAATTYFCKQAPEENQPTNESLQEEWIPFRRVEEGEKEFHV
RNPQVHQRVSECLRVLKILQEVTAGQTVFNILPGGLDLMTLKGNIDMSRVAVMGHSFGGA
TAILALAKETQFRCAVALDAWMFPLERDFYPKARGPVFFINTEKFQTMESVNLMKKICAQ
HEQSRIITVLGSVHRSQTDFAFVTGNLIGKFFSTETRGSLDPYEGQEVMVRAMLAFLQKH
LDLKEDYNQWNNLIEGIGPSLTPGAPHHLSSL
>hsa:5052
MSSGNAKIGHPAPNFKATAVMPDGQFKDISLSDYKGKYVVFFFYPLDFTFVCPTEIIAFS
DRAEEFKKLNCQVIGASVDSHFCHLAWVNTPKKQGGLGPMNIPLVSDPKRTIAQDYGVLK
ADEGISFRGLFIIDDKGILRQITVNDLPVGRSVDETLRLVQAFQFTDKHGEVCPAGWKPG
SDTIKPDVQKSKEYFSKQK
>hsa:5053
MSTAVLENPGLGRKLSDFGQETSYIEDNCNQNGAISLIFSLKEEVGALAKVLRLFEENDV
NLTHIESRPSRLKKDEYEFFTHLDKRSLPALTNIIKILRHDIGATVHELSRDKKKDTVPW
FPRTIQELDRFANQILSYGAELDADHPGFKDPVYRARRKQFADIAYNYRHGQPIPRVEYM
EEEKKTWGTVFKTLKSLYKTHACYEYNHIFPLLEKYCGFHEDNIPQLEDVSQFLQTCTGF
RLRPVAGLLSSRDFLGGLAFRVFHCTQYIRHGSKPMYTPEPDICHELLGHVPLFSDRSFA
QFSQEIGLASLGAPDEYIEKLATIYWFTVEFGLCKQGDSIKAYGAGLLSSFGELQYCLSE
KPKLLPLELEKTAIQNYTVTEFQPLYYVAESFNDAKEKVRNFAATIPRPFSVRYDPYTQR
IEVLDNTQQLKILADSINSEIGILCSALQKIK
>hsa:5091
MLKFRTVHGGLRLLGIRRTSTAPAASPNVRRLEYKPIKKVMVANRGEIAIRVFRACTELG
IRTVAIYSEQDTGQMHRQKADEAYLIGRGLAPVQAYLHIPDIIKVAKENNVDAVHPGYGF
LSERADFAQACQDAGVRFIGPSPEVVRKMGDKVEARAIAIAAGVPVVPGTDAPITSLHEA
HEFSNTYGFPIIFKAAYGGGGRGMRVVHSYEELEENYTRAYSEALAAFGNGALFVEKFIE
KPRHIEVQILGDQYGNILHLYERDCSIQRRHQKVVEIAPAAHLDPQLRTRLTSDSVKLAK
QVGYENAGTVEFLVDRHGKHYFIEVNSRLQVEHTVTEEITDVDLVHAQIHVAEGRSLPDL
GLRQENIRINGCAIQCRVTTEDPARSFQPDTGRIEVFRSGEGMGIRLDNASAFQGAVISP
HYDSLLVKVIAHGKDHPTAATKMSRALAEFRVRGVKTNIAFLQNVLNNQQFLAGTVDTQF
IDENPELFQLRPAQNRAQKLLHYLGHVMVNGPTTPIPVKASPSPTDPVVPAVPIGPPPAG
FRDILLREGPEGFARAVRNHPGLLLMDTTFRDAHQSLLATRVRTHDLKKIAPYVAHNFSK
LFSMENWGGATFDVAMRFLYECPWRRLQELRELIPNIPFQMLLRGANAVGYTNYPDNVVF
KFCEVAKENGMDVFRVFDSLNYLPNMLLGMEAAGSAGGVVEAAISYTGDVADPSRTKYSL
QYYMGLAEELVRAGTHILCIKDMAGLLKPTACTMLVSSLRDRFPDLPLHIHTHDTSGAGV
AAMLACAQAGADVVDVAADSMSGMTSQPSMGALVACTRGTPLDTEVPMERVFDYSEYWEG
ARGLYAAFDCTATMKSGNSDVYENEIPGGQYTNLHFQAHSMGLGSKFKEVKKAYVEANQM
LGDLIKVTPSSKIVGDLAQFMVQNGLSRAEAEAQAEELSFPRSVVEFLQGYIGVPHGGFP
EPFRSKVLKDLPRVEGRPGASLPPLDLQALEKELVDRHGEEVTPEDVLSAAMYPDVFAHF
KDFTATFGPLDSLNTRLFLQGPKIAEEFEVELERGKTLHIKALAVSDLNRAGQRQVFFEL
NGQLRSILVKDTQAMKEMHFHPKALKDVKGQIGAPMPGKVIDIKVVAGAKVAKGQPLCVL
SAMKMETVVTSPMEGTVRKVHVTKDMTLEGDDLILEIE
>hsa:50940
MSPKCSADAENSFKESMEKSSYSDWLINNSIAELVASTGLPVNISDAYQDPRFDAEADQI
SGFHIRSVLCVPIWNSNHQIIGVAQVLNRLDGKPFDDADQRLFEAFVIFCGLGINNTIMY

DQVKKSWAKQSVALDVLSYHATCSKAEVDKFKAANIPLVSELAIDDIHFDDFSLDVDAMI
TAALRMFMELGMVQKFKIDYETLCRWLLTVRKNYRMVLYHNWRHAFNVCQLMFAMLTTAG
FQDILTEVEILAVIVGCLCHDLDHRGTNNAFQAKSGSALAQLYGTSATLEHHHFNHAVMI
LQSEGHNIFANLSSKEYSDLMQLLKQSILATDLTLYFERRTEFFELVSKGEYDWNIKNHR
DIFRSMLMTACDLGAVTKPWEISRQVAELVTSEFFEQGDRERLELKLTPSAIFDRNRKDE
LPRLQLEWIDSICMPLYQALVKVNVKLKPMLDSVATNRSKWEELHQKRLLASTASSSPAS
VMVAKEDRN
>hsa:5095
MAGFWVGTAPLVAAGRRGRWPPQQLMLSAALRTLKHVLYYSRQCLMVSRNLGSVGYDPNE
KTFDKILVANRGEIACRVIRTCKKMGIKTVAIHSDVDASSVHVKMADEAVCVGPAPTSKS
YLNMDAIMEAIKKTRAQAVHPGYGFLSENKEFARCLAAEDVVFIGPDTHAIQAMGDKIES
KLLAKKAEVNTIPGFDGVVKDAEEAVRIAREIGYPVMIKASAGGGGKGMRIAWDDEETRD
GFRLSSQEAASSFGDDRLLIEKFIDNPRHIEIQVLGDKHGNALWLNERECSIQRRNQKVV
EEAPSIFLDAETRRAMGEQAVALARAVKYSSAGTVEFLVDSKKNFYFLEMNTRLQVEHPV
TECITGLDLVQEMIRVAKGYPLRHKQADIRINGWAVECRVYAEDPYKSFGLPSIGRLSQY
QEPLHLPGVRVDSGIQPGSDISIYYDPMISKLITYGSDRTEALKRMADALDNYVIRGVTH
NIALLREVIINSRFVKGDISTKFLSDVYPDGFKGHMLTKSEKNQLLAIASSLFVAFQLRA
QHFQENSRMPVIKPDIANWELSVKLHDKVHTVVASNNGSVFSVEVDGSKLNVTSTWNLAS
PLLSVSVDGTQRTVQCLSREAGGNMSIQFLGTVYKVNILTRLAAELNKFMLEKVTEDTSS
VLRSPMPGVVVAVSVKPGDAVAEGQEICVIEAMKMQNSMTAGKTGTVKSVHCQAGDTVGE
GDLLVELE
>hsa:5096
MAAALRVAAVGARLSVLASGLRAAVRSLCSQATSVNERIENKRRTALLGGGQRRIDAQHK
RGKLTARERISLLLDPGSFVESDMFVEHRCADFGMAADKNKFPGDSVVTGRGRINGRLVY
VFSQDFTVFGGSLSGAHAQKICKIMDQAITVGAPVIGLNDSGGARIQEGVESLAGYADIF
LRNVTASGVIPQISLIMGPCAGGAVYSPALTDFTFMVKDTSYLFITGPDVVKSVTNEDVT
QEELGGAKTHTTMSGVAHRAFENDVDALCNLRDFFNYLPLSSQDPAPVRECHDPSDRLVP
ELDTIVPLESTKAYNMVDIIHSVVDEREFFEIMPNYAKNIIVGFARMNGRTVGIVGNQPK
VASGCLDINSSVKGARFVRFCDAFNIPLITFVDVPGFLPGTAQEYGGIIRHGAKLLYAFA
EATVPKVTVITRKAYGGAYDVMSSKHLCGDTNYAWPTAEIAVMGAKGAVEIIFKGHENVE
AAQAEYIEKFANPFPAAVRGFVDDIIQPSSTRARICCDLDVLASKKVQRPWRKHANIPL
>hsa:51
MILNDPDFQHEDLNFLTRSQRYEVAVRKSAIMVKKMREFGIADPDEIMWFKNFVHRGRPE
PLDLHLGMFLPTLLHQATAEQQERFFMPAWNLEIIGTYAQTEMGHGTHLRGLETTATYDP
ETQEFILNSPTVTSIKWWPGGLGKTSNHAIVLAQLITKGKCYGLHAFIVPIREIGTHKPL
PGITVGDIGPKFGYDEIDNGYLKMDNHRIPRENMLMKYAQVKPDGTYVKPLSNKLTYGTM
VFVRSFLVGEAARALSKACTIAIRYSAVRHQSEIKPGEPEPQILDFQTQQYKLFPLLATA
YAFQFVGAYMKETYHRINEGIGQGDLSELPELHALTAGLKAFTSWTANTGIEACRMACGG
HGYSHCSGLPNIYVNFTPSCTFEGENTVMMLQTARFLMKSYDQVHSGKLVCGMVSYLNDL
PSQRIQPQQVAVWPTMVDINSPESLTEAYKLRAARLVEIAAKNLQKEVIHRKSKEVAWNL
TSVDLVRASEAHCHYVVVKLFSEKLLKIQDKAIQAVLRSLCLLYSLYGISQNAGDFLQGS
IMTEPQITQVNQRVKELLTLIRSDAVALVDAFDFQDVTLGSVLGRYDGNVYENLFEWAKN
SPLNKAEVHESYKHLKSLQSKL
>hsa:51004

MAARLVSRCGAVRAAPHSGPLVSWRRWSGASTDTVYDVVVSGGGLVGAAMACALGYDIHF
HDKKILLLEAGPKKVLEKLSETYSNRVSSISPGSATLLSSFGAWDHICNMRYRAFRRMQV
WDACSEALIMFDKDNLDDMGYIVENDVIMHALTKQLEAVSDRVTVLYRSKAIRYTWPCPF
PMADSSPWVHITLGDGSTFQTKLLIGADGHNSGVRQAVGIQNVSWNYDQSAVVATLHLSE
ATENNVAWQRFLPSGPIALLPLSDTLSSLVWSTSHEHAAELVSMDEEKFVDAVNSAFWSD
ADHTDFIDTAGAMLQYAVSLLKPTKVSARQLPPSVARVDAKSRVLFPLGLGHAAEYVRPR
VALIGDAAHRVHPLAGQGVNMGFGDISSLAHHLSTAAFNGKDLGSVSHLTGYETERQRHN
TALLAATDLLKRLYSTSASPLVLLRTWGLQATNAVSPLKEQIMAFASK

>hsa:51095

MLRCLYHWHRPVLNRRWSRLCLLKQYLFTMKLQSPEFQSLFTEGLKSLTELFVKENHELR
IAGGAVRDLLNGVKPQDIDFATTATPTQMKEMFQSAGIRMINNRGEKHGTITARLHEENF
EITTLRIDVTTDGRHAEVEFTTDWQKDAERRDLTINSMFLGFDGTLFDYFNGYEDLKNKK
VRFVGHAKQRIQEDYLRILRYFRFYGRIVDKPGDHDPETLEAIAENAKGLAGISGERIWV
ELKKILVGNHVNHLIHLIYDLDVAPYIGLPANASLEEFDKVSKNVDGFSPKPVTLLASLF
KVQDDVTKLDLRLKIAKEEKNLGLFIVKNRKDLIKATDSSDPLKPYQDFIIDSREPDATT
RVCELLKYQGEHCLLKEMQQWSIPPFPVSGHDIRKVGISSGKEIGALLQQLREQWKKSGY
QMEKDELLSYIKKT

>hsa:51166

MNYARFITAASAARNPSPIRTMTDILSRGPKSMISLAGGLPNPNMFPFKTAVITVENGKT
IQFGEEMMKRALQYSPSAGIPELLSWLKQLQIKLHNPPTIHYPPSQGQMDLCVTSGSQQG
LCKVFEMIINPGDNVLLDEPAYSGTLQSLHPLGCNIINVASDESGIVPDSLRDILSRWKP
EDAKNPQKNTPKFLYTVPNGNNPTGNSLTSERKKEIYELARKYDFLIIEDDPYYFLQFNK
FRVPTFLSMDVDGRVIRADSFSKIISSGLRIGFLTGPKPLIERVILHIQVSTLHPSTFNQ
LMISQLLHEWGEEGFMAHVDRVIDFYSNQKDAILAAADKWLTGLAEWHVPAAGMFLWIKV
KGINDVKELIEEKAVKMGVLMLPGNAFYVDSSAPSPYLRASFSSASPEQMDVAFQVLAQL
IKESL

>hsa:51172

MATSTGRWLLLRLALFGFLWEASGGLDSGASRDDDLLLPYPRARARLPRDCTRVRAGNRE
HESWPPPPATPGAGGLAVRTFVSHFRDRAVAGHLTRAVEPLRTFSVLEPGGPGGCAARRR
ATVEETARAADCRVAQNGGFFRMNSGECLGNVVSDERRVSSSGGLQNAQFGIRRDGTLVT
GYLSEEEVLDTENPFVQLLSGVVWLIRNGSIYINESQATECDETQETGSFSKFVNVISAR
TAIGHDRKGQLVLFHADGQTEQRGINLWEMAEFLLKQDVVNAINLDGGGSATFVLNGTLA
SYPSDHCQDNMWRCPRQVSTVVCVHEPRCQPPDCHGHGTCVDGHCQCTGHFWRGPGCDEL
DCGPSNCSQHGLCTETGCRCDAGWTGSNCSEECPLGWHGPGCQRPCKCEHHCPCDPKTGN
CSVSRVKQCLQPPEATLRAGELSFFTRTAWLALTLALAFLLLISTAANLSLLLSRAERNR
RLHGDYAYHPLQEMNGEPLAAEKEQPGGAHNPFKD

>hsa:51181

MELFLAGRRVLVTGAGKGRGTVQALHATGARVVAVSRTQADLDSLVRECPGIEPVCVDLG
DWEATERALGSVGPVDLLVNNAAVALLQPFLEVTKEAFDRSFEVNLRAVIQVSQIVARGL
IARGVPGAIVNVSSQCSQRAVTNHSVYCSTKGALDMLTKVMALELGPHKIRVNAVNPTVV
MTSMGQATWSDPHKAKTMLNRIPLGKFAEVEHVVNAILFLLSDRSGMTTGSTLPVEGGFW
AC

>hsa:51205

MITGVFSMRLWTPVGVLTSLAYCLHQRRVALAELQEADGQCPVDRSLLKLKMVQVVFRHG

ARSPLKPLPLEEQVEWNPQLLEVPPQTQFDYTVTNLAGGPKPYSPYDSQYHETTLKGGMF
AGQLTKVGMQQMFALGERLRKNYVEDIPFLSPTFNPQEVFIRSTNIFRNLESTRCLLAGL
FQCQKEGPIIIHTDEADSEVLYPNYQSCWSLRQRTRGRRQTASLQPGISEDLKKVKDRMG
IDSSDKVDFFILLDNVAAEQAHNLPSCPMLKRFARMIEQRAVDTSLYILPKEDRESLQMA
VGPFLHILESNLLKAVDSATAPDKIRKLYLYAAHDVTFIPLLMTLGIFDHKWPPFAVDLT
MELYQHLESKEWFVQLYYHGKEQVPRGCPDGLCPLDMFLNAMSVYTLSPEKYHALCSQTQ
VMEVGNEE
>hsa:51207
MAETSLPELGGEDKATPCPSILELEELLRAGKSSCSRVDEVWPNLFIGDAATANNRFELW
KLGITHVLNAAHKGLYCQGGPDFYGSSVSYLGVPAHDLPDFDISAYFSSAADFIHRALNT
PGAKVLVHCVVGVSRSATLVLAYLMLHQRLSLRQAVITVRQHRWVFPNRGFLHQLCRLDQ
QLRGAGQS
>hsa:5122
MERRAWSLQCTAFVLFCAWCALNSAKAKRQFVNEWAAEIPGGPEAASAIAEELGYDLLGQ
IGSLENHYLFKHKNHPRRSRRSAFHITKRLSDDDRVIWAEQQYEKERSKRSALRDSALNL
FNDPMWNQQWYLQDTRMTAALPKLDLHVIPVWQKGITGKGVVITVLDDGLEWNHTDIYAN
YDPEASYDFNDNDHDPFPRYDPTNENKHGTRCAGEIAMQANNHKCGVGVAYNSKVGGIRM
LDGIVTDAIEASSIGFNPGHVDIYSASWGPNDDGKTVEGPGRLAQKAFEYGVKQGRQGKG
SIFVWASGNGGRQGDNCDCDGYTDSIYTISISSASQQGLSPWYAEKCSSTLATSYSSGDY
TDQRITSADLHNDCTETHTGTSASAPLAAGIFALALEANPNLTWRDMQHLVVWTSEYDPL
ANNPGWKKNGAGLMVNSRFGFGLLNAKALVDLADPRTWRSVPEKKECVVKDNDFEPRALK
ANGEVIIEIPTRACEGQENAIKSLEHVQFEATIEYSRRGDLHVTLTSAAGTSTVLLAERE
RDTSPNGFKNWDFMSVHTWGENPIGTWTLRITDMSGRIQNEGRIVNWKLILHGTSSQPEH
MKQPRVYTSYNTVQNDRRGVEKMVDPGEEQPTQENPKENTLVSKSPSSSSVGGRRDELEE
GAPSQAMLRLLQSAFSKNSPPKQSPKKSPSAKLNIPYENFYEALEKLNKPSQLKDSEDSL
YNDYVDVFYNTKPYKHRDDRLLQALVDILNEEN
>hsa:51251
MTNQESAVHVKMMPEFQKSSVRIKNPTRVEEIICGLIKGGAAKLQIITDFDMTLSRFSYK
GKRCPTCHNIIDNCKLVTDECRKKLLQLKEKYYAIEVDPVLTVEEKYPYMVEWYTKSHGL
LVQQALPKAKLKEIVAESDVMLKEGYENFFDKLQQHSIPVFIFSAGIGDVLEEVIRQAGV
YHPNVKVVSNFMDFDETGVLKGFKGELIHVFNKHDGALRNTEYFNQLKDNSNIILLGDSQ
GDLRMADGVANVEHILKIGYLNDRVDELLEKYMDSYDIVLVQDESLEVANSILQKIL
>hsa:5126
MVFASAERPVFTNHFLVELHKGGEDKARQVAAEHGFGVRKLPFAEGLYHFYHNGLAKAKR
RRSLHHKQQLERDPRVKMALQQEGFDRKKRGYRDINEIDINMNDPLFTKQWYLINTGQAD
GTPGLDLNVAEAWELGYTGKGVTIGIMDDGIDYLHPDLASNYNAEASYDFSSNDPYPYPR
YTDDWFNSHGTRCAGEVSAAANNNICGVGVAYNSKVAGIRMLDQPFMTDIIEASSISHMP
QLIDIYSASWGPTDNGKTVDGPRELTLQAMADGVNKGRGGKGSIYVWASGDGGSYDDCNC
DGYASSMWTISINSAINDGRTALYDESCSSTLASTFSNGRKRNPEAGVATTDLYGNCTLR
HSGTSAAAPEAAGVFALALEANLGLTWRDMQHLTVLTSKRNQLHDEVHQWRRNGVGLEFN
HLFGYGVLDAGAMVKMAKDWKTVPERFHCVGGSVQDPEKIPSTGKLVLTLTTDACEGKEN
FVRYLEHVQAVITVNATRRGDLNINMTSPMGTKSILLSRRPRDDDSKVGFDKWPFMTTHT
WGEDARGTWTLELGFVGSAPQKGVLKEWTLMLHGTQSAPYIDQVVRDYQSKLAMSKKEEL
EEELDEAVERSLKSILNKN

>hsa:51292
MPHIDNDVKLDFKDVLLRPKRSTLKSRSEVDLTRSFSFRNSKQTYSGVPIIAANMDTVGT
FEMAKVLCKFSLFTAVHKHYSLVQWQEFAGQNPDCLEHLAASSGTGSSDFEQLEQILEAI
PQVKYICLDVANGYSEHFVEFVKDVRKRFPQHTIMAGNVVTGEMVEELILSGADIIKVGI
GPGSVCTTRKKTGVGYPQLSAVMECADAAHGLKGHIISDGGCSCPGDVAKAFGAGADFVM
LGGMLAGHSESGGELIERDGKKYKLFYGMSSEMAMKKYAGGVAEYRASEGKTVEVPFKGD
VEHTIRDILGGIRSTCTYVGAAKLKELSRRTTFIRVTQQVNPIFSEAC
>hsa:513
MLPAALLRRPGLGRLVRHARAYAEAAAAPAAASGPNQMSFTFASPTQVFFNGANVRQVDV
PTLTGAFGILAAHVPTLQVLRPGLVVVHAEDGTTSKYFVSSGSIAVNADSSVQLLAEEAV
TLDMLDLGAAKANLEKAQAELVGTADEATRAEIQIRIEANEALVKALE
>hsa:51302
MELISPTVIIILGCLALFLLLQRKNLRRPPCIKGWIPWIGVGFEFGKAPLEFIEKARIKY
GPIFTVFAMGNRMTFVTEEEGINVFLKSKKVDFELAVQNIVYRTASIPKNVFLALHEKLY
IMLKGKMGTVNLHQFTGQLTEELHEQLENLGTHGTMDLNNLVRHLLYPVTVNMLFNKSLF
STNKKKIKEFHQYFQVYDEDFEYGSQLPECLLRNWSKSKKWFLELFEKNIPDIKACKSAK
DNSMTLLQATLDIVETETSKENSPNYGLLLLWASLSNAVPVAFWTLAYVLSHPDIHKAIM
EGISSVFGKAGKDKIKVSEDDLENLLLIKWCVLETIRLKAPGVITRKVVKPVEILNYIIP
SGDLLMLSPFWLHRNPKYFPEPELFKPERWKKANLEKHSFLDCFMAFGSGKFQCPARWFA
LLEVQMCIILILYKYDCSLLDPLPKQSYLHLVGVPQPEGQCRIEYKQRI
>hsa:5136
MGSSATEIEELENTTFKYLTGEQTEKMWQRLKGILRCLVKQLERGDVNVVDLKKNIEYAA
SVLEAVYIDETRRLLDTEDELSDIQTDSVPSEVRDWLASTFTRKMGMTKKKPEEKPKFRS
IVHAVQAGIFVERMYRKTYHMVGLAYPAAVIVTLKDVDKWSFDVFALNEASGEHSLKFMI
YELFTRYDLINRFKIPVSCLITFAEALEVGYSKYKNPYHNLIHAADVTQTVHYIMLHTGI
MHWLTELEILAMVFAAAIHDYEHTGTTNNFHIQTRSDVAILYNDRSVLENHHVSAAYRLM
QEEEMNILINLSKDDWRDLRNLVIEMVLSTDMSGHFQQIKNIRNSLQQPEGIDRAKTMSL
ILHAADISHPAKSWKLHYRWTMALMEEFFLQGDKEAELGLPFSPLCDRKSTMVAQSQIGF
IDFIVEPTFSLLTDSTEKIVIPLIEEASKAETSSYVASSSTTIVGLHIADALRRSNTKGS
MSDGSYSPDYSLAAVDLKSFKNNLVDIIQQNKERWKELAAQEARTSSQKCEFIHQ
>hsa:51365
MPPGPWESCFWVGGLILWLSVGSSGDAPPTPQPKCADFQSANLFEGTDLKVQFLLFVPSN
PSCGQLVEGSSDLQNSGFNATLGTKLIIHGFRVLGTKPSWIDTFIRTLLRATNANVIAVD
WIYGSTGVYFSAVKNVLGVSESSIHIIGVSLGAHVGGMVGQLFGGQLGQITGLDPAGPEY
TRASVEERLDAGDALFVEAIHTDTDNLGIRIPVGHVDYFVNGGQDQPGCPTFFYAGYSYL
ICDHMRAVHLYISALENSCPLMAFPCASYKAFLAGRCLDCFNPFLLSCPRIGLVEQGGVK
IEPLPKEVKVYLLTTSSAPYCMHHSLVEFHLKELRNKDTNIEVTFLSSNITSSSKITIPK
QQRYGKGIIAHATPQCQINQVKFKFQSSNRVWKKDRTTIIGKFCTALLPVNDREKMVCLP
EPVNLQASVTVSCDLKIACV
>hsa:5137
MESPTKEIEEFESNSLKYLQPEQIEKIWLRLRGLRKYKKTSQRLRSLVKQLERGEASVVD
LKKNLEYAATVLESVYIDETRRLLDTEDELSDIQSDAVPSEVRDWLASTFTRQMGMMLRR
SDEKPRFKSIVHAVQAGIFVERMYRRTSNMVGLSYPPAVIEALKDVDKWSFDVFSLNEAS
GDHALKFIFYELLTRYDLISRFKIPISALVSFVEALEVGYSKHKNPYHNLMHAADVTQTV

HYLLYKTGVANWLTELEIFAIIFSAAIHDYEHTGTTNNFHIQTRSDPAILYNDRSVLENH
HLSAAYRLLQDDEEMNILINLSKDDWREFRTLVIEMVMATDMSCHFQQIKAMKTALQQPE
AIEKPKALSLMLHTADISHPAKAWDLHHRWTMSLLEEFFRQGDREAELGLPFSPLCDRKS
TMVAQSQVGFIDFIVEPTFTVLTDMTEKIVSPLIDETSQTGGTGQRRSSLNSISSSDAKR
SGVKTSGSEGSAPINNSVISVDYKSFKATWTEVVHINRERWRAKVPKEEKAKKEAEEKAR
LAAEEQQKEMEAKSQAEEGASGKAEKKTSGETKNQVNGTRANKSDNPRGKNSKAEKSSGE
QQQNGDFKDGKNKTDKKDHSNIGNDSKKTDDSQE
>hsa:5138
MVLVLHHILIAVVQFLRRGQQVFLKPDEPPPPPQPCADSLQDALLSLGSVIDISGLQRAV
KEALSAVLPRVETVYTYLLDGESQLVCEDPPHELPQEGKVREAIISQKRLGCNGLGFSDL
PGKPLARLVAPLAPDTQVLVMPLADKEAGAVAAVILVHCGQLSDNEEWSLQAVEKHTLVA
LRRVQVLQQRGPREAPRAVQNPPEGTAEDQKGGAAYTDRDRKILQLCGELYDLDASSLQL
KVLQYLQQETRASRCCLLLVSEDNLQLSCKVIGDKVLGEEVSFPLTGCLGQVVEDKKSIQ
LKDLTSEDVQQLQSMLGCELQAMLCVPVISRATDQVVALACAFNKLEGDLFTDEDEHVIQ
HCFHYTSTVLTSTLAFQKEQKLKCECQALLQVAKNLFTHLDDVSVLLQEIITEARNLSNA
EICSVFLLDQNELVAKVFDGGVVDDESYEIRIPADQGIAGHVATTGQILNIPDAYAHPLF
YRGVDDSTGFRTRNILCFPIKNENQEVIGVAELVNKINGPWFSKFDEDLATAFSIYCGIS
IAHSLLYKKVNEAQYRSHLANEMMMYHMKVSDDEYTKLLHDGIQPVAAIDSNFASFTYTP
RSLPEDDTSMAILSMLQDMNFINNYKIDCPTLARFCLMVKKGYRDPPYHNWMHAFSVSHF
CYLLYKNLELTNYLEDIEIFALFISCMCHDLDHRGTNNSFQVASKSVLAALYSSEGSVME
RHHFAQAIAILNTHGCNIFDHFSRKDYQRMLDLMRDIILATDLAHHLRIFKDLQKMAEVG
YDRNNKQHHRLLLCLLMTSCDLSDQTKGWKTTRKIAELIYKEFFSQGDLEKAMGNRPMEM
MDREKAYIPELQISFMEHIAMPIYKLLQDLFPKAAELYERVASNREHWTKVSHKFTIRGL
PSNNSLDFLDEEYEVPDLDGTRAPINGCCSLDAE
>hsa:5140
MRRDERDAKAMRSLQPPDGAGSPPESLRNGYVKSCVSPLRQDPPRGFFFHLCRFCNVELR
PPPASPQQPRRCSPFCRARLSLGALAAFVLALLLGAEPESWAAGAAWLRTLLSVCSHSLS
PLFSIACAFFFLTCFLTRTKRGPGPGRSCGSWWLLALPACCYLGDFLVWQWWSWPWGDGD
AGSAAPHTPPEAAAGRLLLVLSCVGLLLTLAHPLRLRHCVLVLLLASFVWWVSFTSLGSL
PSALRPLLSGLVGGAGCLLALGLDHFFQIREAPLHPRLSSAAEEKVPVIRPRRRSSCVSL
GETAASYYGSCKIFRRPSLPCISREQMILWDWDLKQWYKPHYQNSGGGNGVDLSVLNEAR
NMVSDLLTDPSLPPQVISSLRSISSLMGAFSGSCRPKINPLTPFPGFYPCSEIEDPAEKG
DRKLNKGLNRNSLPTPQLRRSSGTSGLLPVEQSSRWDRNNGKRPHQEFGISSQGCYLNGP
FNSNLLTIPKQRSSSVSLTHHVGLRRAGVLSSLSPVNSSNHGPVSTGSLTNRSPIEFPDT
ADFLNKPSVILQRSLGNAPNTPDFYQQLRNSDSNLCNSCGHQMLKYVSTSESDGTDCCSG
KSGEEENIFSKESFKLMETQQEEETEKKDSRKLFQEGDKWLTEEAQSEQQTNIEQEVSLD
LILVEEYDSLIEKMSNWNFPIFELVEKMGEKSGRILSQVMYTLFQDTGLLEIFKIPTQQF
MNYFRALENGYRDIPYHNRIHATDVLHAVWYLTTRPVPGLQQIHNGCGTGNETDSDGRIN
HGRIAYISSKSCSNPDESYGCLSSNIPALELMALYVAAAMHDYDHPGRTNAFLVATNAPQ
AVLYNDRSVLENHHAASAWNLYLSRPEYNFLLHLDHVEFKRFRFLVIEAILATDLKKHFD
FLAEFNAKANDVNSNGIEWSNENDRLLVCQVCIKLADINGPAKVRDLHLKWTEGIVNEFY
EQGDEEANLGLPISPFMDRSSPQLAKLQESFITHIVGPLCNSYDAAGLLPGQWLEAEEDN
DTESGDDEDGEELDTEDEEMENNLNPKPPRRKSRRRIFCQLMHHLTENHKIWKEIVEEEE
KCKADGNKLQVENSSLPQADEIQVIEEADEEE

>hsa:5141
MEPPTVPSERSLSLSLPGPREGQATLKPPPQHLWRQPRTPIRIQQRGYSDSAERAERERQ
PHRPIERADAMDTSDRPGLRTTRMSWPSSFHGTGTGSGGAGGGSSRRFEAENGPTPSPGR
SPLDSQASPGLVLHAGAATSQRRESFLYRSDSDYDMSPKTMSRNSSVTSEAHAEDLIVTP
FAQVLASLRSVRSNFSLLTNVPVPSNKRSPLGGPTPVCKATLSEETCQQLARETLEELDW
CLEQLETMQTYRSVSEMASHKFKRMLNRELTHLSEMSRSGNQVSEYISTTFLDKQNEVEI
PSPTMKEREKQQAPRPRPSQPPPPPVPHLQPMSQITGLKKLMHSNSLNNSNIPRFGVKTD
QEELLAQELENLNKWGLNIFCVSDYAGGRSLTCIMYMIFQERDLLKKFRIPVDTMVTYML
TLEDHYHADVAYHNSLHAADVLQSTHVLLATPALDAVFTDLEILAALFAAAIHDVDHPGV
SNQFLINTNSELALMYNDESVLENHHLAVGFKLLQEDNCDIFQNLSKRQRQSLRKMVIDM
VLATDMSKHMTLLADLKTMVETKKVTSSGVLLLDNYSDRIQVLRNMVHCADLSNPTKPLE
LYRQWTDRIMAEFFQQGDRERERGMEISPMCDKHTASVEKSQVGFIDYIVHPLWETWADL
VHPDAQEILDTLEDNRDWYYSAIRQSPSPPPEEESRGPGHPPLPDKFQFELTLEEEEEEE
ISMAQIPCTAQEALTAQGLSGVEEALDATIAWEASPAQESLEVMAQEASLEAELEAVYLT
QQAQSTGSAPVAPDEFSSREEFVVAVSHSSPSALALQSPLLPAWRTLSVSEHAPGLPGLP
STAAEVEAQREHQAAKRACSACAGTFGEDTSALPAPGGGGSGGDPT
>hsa:5142
MKEHGGTFSSTGISGGSGDSAMDSLQPLQPNYMPVCLFAEESYQKLAMETLEELDWCLDQ
LETIQTYRSVSEMASNKFKRMLNRELTHLSEMSRSGNQVSEYISNTFLDKQNDVEIPSPT
QKDREKKKKQQLMTQISGVKKLMHSSSLNNTSISRFGVNTENEDHLAKELEDLNKWGLNI
FNVAGYSHNRPLTCIMYAIFQERDLLKTFRISSDTFITYMMTLEDHYHSDVAYHNSLHAA
DVAQSTHVLLSTPALDAVFTDLEILAAIFAAAIHDVDHPGVSNQFLINTNSELALMYNDE
SVLENHHLAVGFKLLQEEHCDIFMNLTKKQRQTLRKMVIDMVLATDMSKHMSLLADLKTM
VETKKVTSSGVLLLDNYTDRIQVLRNMVHCADLSNPTKSLELYRQWTDRIMEEFFQQGDK
ERERGMEISPMCDKHTASVEKSQVGFIDYIVHPLWETWADLVQPDAQDILDTLEDNRNWY
QSMIPQSPSPPLDEQNRDCQGLMEKFQFELTLDEEDSEGPEKEGEGHSYFSSTKTLCVID
PENRDSLGETDIDIATEDKSPVDT
>hsa:5143
MENLGVGEGAEACSRLSRSRGRHSMTRAPKHLWRQPRRPIRIQQRFYSDPDKSAGCRERD
LSPRPELRKSRLSWPVSSCRRFDLENGLSCGRRALDPQSSPGLGRIMQAPVPHSQRRESF
LYRSDSDYELSPKAMSRNSSVASDLHGEDMIVTPFAQVLASLRTVRSNVAALARQQCLGA
AKQGPVGNPSSSNQLPPAEDTGQKLALETLDELDWCLDQLETLQTRHSVGEMASNKFKRI
LNRELTHLSETSRSGNQVSEYISRTFLDQQTEVELPKVTAEEAPQPMSRISGLHGLCHSA
SLSSATVPRFGVQTDQEEQLAKELEDTNKWGLDVFKVAELSGNRPLTAIIFSIFQERDLL
KTFQIPADTLATYLLMLEGHYHANVAYHNSLHAADVAQSTHVLLATPALEAVFTDLEILA
ALFASAIHDVDHPGVSNQFLINTNSELALMYNDASVLENHHLAVGFKLLQAENCDIFQNL
SAKQRLSLRRMVIDMVLATDMSKHMNLLADLKTMVETKKVTSLGVLLLDNYSDRIQVLQN
LVHCADLSNPTKPLPLYRQWTDRIMAEFFQQGDRERESGLDISPMCDKHTASVEKSQVGF
IDYIAHPLWETWADLVHPDAQDLLDTLEDNREWYQSKIPRSPSDLTNPERDGPDRFQFEL
TLEEAEEEDEEEEEEGEETALAKEALELPDTELLSPEAGPDPGDLPLDNQRT
>hsa:5144
MEAEGSSAPARAGSGEGSDSAGGATLKAPKHLWRHEQHHQYPLRQPQFRLLHPHHHLPPP
PPPSPQPQPQCPLQPPPPPPLPPPPPPPGAARGRYASSGATGRVRHRGYSDTERYLYCRA
MDRTSYAVETGHRPGLKKSRMSWPSSFQGLRRFDVDNGTSAGRSPLDPMTSPGSGLILQA

NFVHSQRRESFLYRSDSDYDLSPKSMSRNSSIASDIHGDDLIVTPFAQVLASLRTVRNNF
AALTNLQDRAPSKRSPMCNQPSINKATITEEAYQKLASETLEELDWCLDQLETLQTRHSV
SEMASNKFKRMLNRELTHLSEMSRSGNQVSEFISNTFLDKQHEVEIPSPTQKEKEKKKRP
MSQISGVKKLMHSSSLTNSSIPRFGVKTEQEDVLAKELEDVNKWGLHVFRIAELSGNRPL
TVIMHTIFQERDLLKTFKIPVDTLITYLMTLEDHYHADVAYHNNIHAADVVQSTHVLLST
PALEAVFTDLEILAAIFASAIHDVDHPGVSNQFLINTNSELALMYNDSSVLENHHLAVGF
KLLQEENCDIFQNLTKKQRQSLRKMVIDIVLATDMSKHMNLLADLKTMVETKKVTSSGVL
LLDNYSDRIQVLQNMVHCADLSNPTKPLQLYRQWTDRIMEEFFRQGDRERERGMEISPMC
DKHNASVEKSQVGFIDYIVHPLWETWADLVHPDAQDILDTLEDNREWYQSTIPQSPSPAP
DDPEEGRQGQTEKFQFELTLEEDGESDTEKDSGSQVEEDTSCSDSKTLCTQDSESTEIPL
DEQVEEEAVGEEEESQPEACVIDDRSPDT
>hsa:5145
MGEVTAEEVEKFLDSNIGFAKQYYNLHYRAKLISDLLGAKEAAVDFSNYHSPSSMEESEI
IFDLLRDFQENLQTEKCIFNVMKKLCFLLQADRMSLFMYRTRNGIAELATRLFNVHKDAV
LEDCLVMPDQEIVFPLDMGIVGHVAHSKKIANVPNTEEDEHFCDFVDILTEYKTKNILAS
PIMNGKDVVAIIMAVNKVDGSHFTKRDEEILLKYLNFANLIMKVYHLSYLHNCETRRGQI
LLWSGSKVFEELTDIERQFHKALYTVRAFLNCDRYSVGLLDMTKQKEFFDVWPVLMGEVP
PYSGPRTPDGREINFYKVIDYILHGKEDIKVIPNPPPDHWALVSGLPAYVAQNGLICNIM
NAPAEDFFAFQKEPLDESGWMIKNVLSMPIVNKKEEIVGVATFYNRKDGKPFDEMDETLM
ESLTQFLGWSVLNPDTYESMNKLENRKDIFQDIVKYHVKCDNEEIQKILKTREVYGKEPW
ECEEEELAEILQAELPDADKYEINKFHFSDLPLTELELVKCGIQMYYELKVVDKFHIPQE
ALVRFMYSLSKGYRKITYHNWRHGFNVGQTMFSLLVTGKLKRYFTDLEALAMVTAAFCHD
IDHRGTNNLYQMKSQNPLAKLHGSSILERHHLEFGKTLLRDESLNIFQNLNRRQHEHAIH
MMDIAIIATDLALYFKKRTMFQKIVDQSKTYESEQEWTQYMMLEQTRKEIVMAMMMTACD
LSAITKPWEVQSQVALLVAAEFWEQGDLERTVLQQNPIPMMDRNKADELPKLQVGFIDFV
CTFVYKEFSRFHEEITPMLDGITNNRKEWKALADEYDAKMKVQEEKKQKQQSAKSAAAGN
QPGGNPSPGGATTSKSCCIQ
>hsa:5146
MGEINQVAVEKYLEENPQFAKEYFDRKLRVEVLGEIFKNSQVPVQSSMSFSELTQVEESA
LCLELLWTVQEEGGTPEQGVHRALQRLAHLLQADRCSMFLCRSRNGIPEVASRLLDVTPT
SKFEDNLVGPDKEVVFPLDIGIVGWAAHTKKTHNVPDVKKNSHFSDFMDKQTGYVTKNLL
ATPIVVGKEVLAVIMAVNKVNASEFSKQDEEVFSKYLNFVSIILRLHHTSYMYNIESRRS
QILMWSANKVFEELTDVERQFHKALYTVRSYLNCERYSIGLLDMTKEKEFYDEWPIKLGE
VEPYKGPKTPDGREVNFYKIIDYILHGKEEIKVIPTPPADHWTLISGLPTYVAENGFICN
MMNAPADEYFTFQKGPVDETGWVIKNVLSLPIVNKKEDIVGVATFYNRKDGKPFDEHDEY
ITETLTQFLGWSLLNTDTYDKMNKLENRKDIAQEMLMNQTKATPEEIKSILKFQEKLNVD
VIDDCEEKQLVAILKEDLPDPRSAELYEFRFSDFPLTEHGLIKCGIRLFFEINVVEKFKV
PVEVLTRWMYTVRKGYRAVTYHNWRHGFNVGQTMFTLLMTGRLKKYYTDLEAFAMLAAAF
CHDIDHRGTNNLYQMKSTSPLARLHGSSILERHHLEYSKTLLQDESLNIFQNLNKRQFET
VIHLFEVAIIATDLALYFKKRTMFQKIVDACEQMQTEEEAIKYVTVDPTKKEIIMAMMMT
ACDLSAITKPWEVQSQVALMVANEFWEQGDLERTVLQQQPIPMMDRNKRDELPKLQVGFI
DFVCTFVYKEFSRFHKEITPMLSGLQNNRVEWKSLADEYDAKMKVIEEEAKKQEGGAEKA
AEDSGGGDDKKSKTCLML
>hsa:5147

MSAKDERAREILRGFKLNWMNLRDAETGKILWQGTEDLSVPGVEHEARVPKKILKCKAVS
RELNFSSTEQMEKFRLEQKVYFKGQCLEEWFFEFGFVIPNSTNTWQSLIEAAPESQMMPA
SVLTGNVIIETKFFDDDLLVSTSRVRLFYV
>hsa:5148
MNLEPPKAEFRSATRVAGGPVTPRKGPPKFKQRQTRQFKSKPPKKGVQGFGDDIPGMEGL
GTDITVICPWEAFNHLELHELAQYGII
>hsa:5149
MSDNTTLPAPASNQGPTTPRKGPPKFKQRQTRQFKSKPPKKGVKGFGDDIPGMEGLGTDI
TVICPWEAFSHLELHELAQFGII
>hsa:5150
MEVCYQLPVLPLDRPVPQHVLSRRGAISFSSSSALFGCPNPRQLSQRRGAISYDSSDQTA
LYIRMLGDVRVRSRAGFESERRGSHPYIDFRIFHSQSEIEVSVSARNIRRLLSFQRYLRS
SRFFRGTAVSNSLNILDDDYNGQAKCMLEKVGNWNFDIFLFDRLTNGNSLVSLTFHLFSL
HGLIEYFHLDMMKLRRFLVMIQEDYHSQNPYHNAVHAADVTQAMHCYLKEPKLANSVTPW
DILLSLIAAATHDLDHPGVNQPFLIKTNHYLATLYKNTSVLENHHWRSAVGLLRESGLFS
HLPLESRQQMETQIGALILATDISRQNEYLSLFRSHLDRGDLCLEDTRHRHLVLQMALKC
ADICNPCRTWELSKQWSEKVTEEFFHQGDIEKKYHLGVSPLCDRHTESIANIQIGFMTYL
VEPLFTEWARFSNTRLSQTMLGHVGLNKASWKGLQREQSSSEDTDAAFELNSQLLPQENR
LS
>hsa:5151
MRFHQDQLQVLLVFTKEDNQCNGFCRACEKAGFKCTVTKEAQAVLACFLDKHHDIIIIDH
RNPRQLDAEALCRSIRSSKLSENTVIVGVVRRVDREELSVMPFISAGFTRRYVENPNIMA
CYNELLQLEFGEVRSQLKLRACNSVFTALENSEDAIEITSEDRFIQYANPAFETTMGYQS
GELIGKELGEVPINEKKADLLDTINSCIRIGKEWQGIYYAKKKNGDNIQQNVKIIPVIGQ
GGKIRHYVSIIRVCNGNNKAEKISECVQSDTHTDNQTGKHKDRRKGSLDVKAVASRATEV
SSQRRHSSMARIHSMTIEAPITKVINIINAAQESSPMPVTEALDRVLEILRTTELYSPQF
GAKDDDPHANDLVGGLMSDGLRRLSGNEYVLSTKNTQMVSSNIITPISLDDVPPRIARAM
ENEEYWDFDIFELEAATHNRPLIYLGLKMFARFGICEFLHCSESTLRSWLQIIEANYHSS
NPYHNSTHSADVLHATAYFLSKERIKETLDPIDEVAALIAATIHDVDHPGRTNSFLCNAG
SELAILYNDTAVLESHHAALAFQLTTGDDKCNIFKNMERNDYRTLRQGIIDMVLATEMTK
HFEHVNKFVNSINKPLATLEENGETDKNQEVINTMLRTPENRTLIKRMLIKCADVSNPCR
PLQYCIEWAARISEEYFSQTDEEKQQGLPVVMPVFDRNTCSIPKSQISFIDYFITDMFDA
WDAFVDLPDLMQHLDNNFKYWKGLDEMKLRNLRPPPE
>hsa:5152
MGSGSSSYRPKAIYLDIDGRIQKVIFSKYCNSSDIMDLFCIATGLPRNTTISLLTTDDAM
VSIDPTMPANSERTPYKVRPVAIKQLSEREELIQSVLAQVAEQFSRAFKINELKAEVANH
LAVLEKRVELEGLKVVEIEKCKSDIKKMREELAARSSRTNCPCKYSFLDNHKKLTPRRDV
PTYPKYLLSPETIEALRKPTFDVWLWEPNEMLSCLEHMYHDLGLVRDFSINPVTLRRWLF
CVHDNYRNNPFHNFRHCFCVAQMMYSMVWLCSLQEKFSQTDILILMTAAICHDLDHPGYN
NTYQINARTELAVRYNDISPLENHHCAVAFQILAEPECNIFSNIPPDGFKQIRQGMITLI
LATDMARHAEIMDSFKEKMENFDYSNEEHMTLLKMILIKCCDISNEVRPMEVAEPWVDCL
LEEYFMQSDREKSEGLPVAPFMDRDKVTKATAQIGFIKFVLIPMFETVTKLFPMVEEIML
QPLWESRDRYEELKRIDDAMKELQKKTDSLTSGATEKSRERSRDVKNSEGDCA
>hsa:5156

MGTSHPAFLVLGCLLTGLSLILCQLSLPSILPNENEKVVQLNSSFSLRCFGESEVSWQYP
MSEEESSDVEIRNEENNSGLFVTVLEVSSASAAHTGLYTCYYNHTQTEENELEGRHIYIY
VPDPDVAFVPLGMTDYLVIVEDDDSAIIPCRTTDPETPVTLHNSEGVVPASYDSRQGFNG
TFTVGPYICEATVKGKKFQTIPFNVYALKATSELDLEMEALKTVYKSGETIVVTCAVFNN
EVVDLQWTYPGEVKGKGITMLEEIKVPSIKLVYTLTVPEATVKDSGDYECAARQATREVK
EMKKVTISVHEKGFIEIKPTFSQLEAVNLHEVKHFVVEVRAYPPPRISWLKNNLTLIENL
TEITTDVEKIQEIRYRSKLKLIRAKEEDSGHYTIVAQNEDAVKSYTFELLTQVPSSILDL
VDDHHGSTGGQTVRCTAEGTPLPDIEWMICKDIKKCNNETSWTILANNVSNIITEIHSRD
RSTVEGRVTFAKVEETIAVRCLAKNLLGAENRELKLVAPTLRSELTVAAAVLVLLVIVII
SLIVLVVIWKQKPRYEIRWRVIESISPDGHEYIYVDPMQLPYDSRWEFPRDGLVLGRVLG
SGAFGKVVEGTAYGLSRSQPVMKVAVKMLKPTARSSEKQALMSELKIMTHLGPHLNIVNL
LGACTKSGPIYIITEYCFYGDLVNYLHKNRDSFLSHHPEKPKKELDIFGLNPADESTRSY
VILSFENNGDYMDMKQADTTQYVPMLERKEVSKYSDIQRSLYDRPASYKKKSMLDSEVKN
LLSDDNSEGLTLLDLLSFTYQVARGMEFLASKNCVHRDLAARNVLLAQGKIVKICDFGLA
RDIMHDSNYVSKGSTFLPVKWMAPESIFDNLYTTLSDVWSYGILLWEIFSLGGTPYPGMM
VDSTFYNKIKSGYRMAKPDHATSEVYEIMVKCWNSEPEKRPSFYHLSEIVENLLPGQYKK
SYEKIHLDFLKSDHPAVARMRVDSDNAYIGVTYKNEEDKLKDWEGGLDEQRLSADSGYII
PLPDIDPVPEEEDLGKRNRHSSQTSEESAIETGSSSSTFIKREDETIEDIDMMDDIGIDS
SDLVEDSFL
>hsa:5158
MSLSEEQARSFLDQNPDFARQYFGKKLSPENVAAACEDGCPPDCDSLRDLCQVEESTALL
ELVQDMQESINMERVVFKVLRRLCTLLQADRCSLFMYRQRNGVAELATRLFSVQPDSVLE
DCLVPPDSEIVFPLDIGVVGHVAQTKKMVNVEDVAECPHFSSFADELTDYKTKNMLATPI
MNGKDVVAVIMAVNKLNGPFFTSEDEDVFLKYLNFATLYLKIYHLSYLHNCETRRGQVLL
WSANKVFEELTDIERQFHKAFYTVRAYLNCERYSVGLLDMTKEKEFFDVWSVLMGESQPY
SGPRTPDGREIVFYKVIDYILHGKEEIKVIPTPSADHWALASGLPSYVAESGFICNIMNA
SADEMFKFQEGALDDSGWLIKNVLSMPIVNKKEEIVGVATFYNRKDGKPFDEQDEVLMES
LTQFLGWSVMNTDTYDKMNKLENRKDIAQDMVLYHVKCDRDEIQLILPTRARLGKEPADC
DEDELGEILKEELPGPTTFDIYEFHFSDLECTELDLVKCGIQMYYELGVVRKFQIPQEVL
VRFLFSISKGYRRITYHNWRHGFNVAQTMFTLLMTGKLKSYYTDLEAFAMVTAGLCHDID
HRGTNNLYQMKSQNPLAKLHGSSILERHHLEFGKFLLSEETLNIYQNLNRRQHEHVIHLM
DIAIIATDLALYFKKRAMFQKIVDESKNYQDKKSWVEYLSLETTRKEIVMAMMMTACDLS
AITKPWEVQSKVALLVAAEFWEQGDLERTVLDQQPIPMMDRNKAAELPKLQVGFIDFVCT
FVYKEFSRFHEEILPMFDRLQNNRKEWKALADEYEAKVKALEEKEEEERVAAKKVGTEIC
NGGPAPKSSTCCIL
>hsa:5159
MRLPGAMPALALKGELLLLSLLLLLEPQISQGLVVTPPGPELVLNVSSTFVLTCSGSAPV
VWERMSQEPPQEMAKAQDGTFSSVLTLTNLTGLDTGEYFCTHNDSRGLETDERKRLYIFV
PDPTVGFLPNDAEELFIFLTEITEITIPCRVTDPQLVVTLHEKKGDVALPVPYDHQRGFS
GIFEDRSYICKTTIGDREVDSDAYYVYRLQVSSINVSVNAVQTVVRQGENITLMCIVIGN
EVVNFEWTYPRKESGRLVEPVTDFLLDMPYHIRSILHIPSAELEDSGTYTCNVTESVNDH
QDEKAINITVVESGYVRLLGEVGTLQFAELHRSRTLQVVFEAYPPPTVLWFKDNRTLGDS
SAGEIALSTRNVSETRYVSELTLVRVKVAEAGHYTMRAFHEDAEVQLSFQLQINVPVRVL
ELSESHPDSGEQTVRCRGRGMPQPNIIWSACRDLKRCPRELPPTLLGNSSEEESQLETNV

TYWEEEQEFEVVSTLRLQHVDRPLSVRCTLRNAVGQDTQEVIVVPHSLPFKVVVISAILA
LVVLTIISLIILIMLWQKKPRYEIRWKVIESVSSDGHEYIYVDPMQLPYDSTWELPRDQL
VLGRTLGSGAFGQVVEATAHGLSHSQATMKVAVKMLKSTARSSEKQALMSELKIMSHLGP
HLNVVNLLGACTKGGPIYIITEYCRYGDLVDYLHRNKHTFLQHHSDKRRPPSAELYSNAL
PVGLPLPSHVSLTGESDGGYMDMSKDESVDYVPMLDMKGDVKYADIESSNYMAPYDNYVP
SAPERTCRATLINESPVLSYMDLVGFSYQVANGMEFLASKNCVHRDLAARNVLICEGKLV
KICDFGLARDIMRDSNYISKGSTFLPLKWMAPESIFNSLYTTLSDVWSFGILLWEIFTLG
GTPYPELPMNEQFYNAIKRGYRMAQPAHASDEIYEIMQKCWEEKFEIRPPFSQLVLLLER
LLGEGYKKKYQQVDEEFLRSDHPAILRSQARLPGFHGLRSPLDTSSVLYTAVQPNEGDND
YIIPLPDPKPEVADEGPLEGSPSLASSTLNEVNTSSTISCDSPLEPQDEPEPEPQLELQV
EPEPELEQLPDSGCPAPRAEAEDSFL
>hsa:51645
MAAIPPDSWQPPNVYLETSMGIIVLELYWKHAPKTCKNFAELARRGYYNGTKFHRIIKDF
MIQGGDPTGTGRGGASIYGKQFEDELHPDLKFTGAGILAMANAGPDTNGSQFFVTLAPTQ
WLDGKHTIFGRVCQGIGMVNRVGMVETNSQDRPVDDVKIIKAYPSG
>hsa:5167
MERDGCAGGGSRGGEGGRAPREGPAGNGRDRGRSHAAEAPGDPQAAASLLAPMDVGEEPL
EKAARARTAKDPNTYKVLSLVLSVCVLTTILGCIFGLKPSCAKEVKSCKGRCFERTFGNC
RCDAACVELGNCCLDYQETCIEPEHIWTCNKFRCGEKRLTRSLCACSDDCKDKGDCCINY
SSVCQGEKSWVEEPCESINEPQCPAGFETPPTLLFSLDGFRAEYLHTWGGLLPVISKLKK
CGTYTKNMRPVYPTKTFPNHYSIVTGLYPESHGIIDNKMYDPKMNASFSLKSKEKFNPEW
YKGEPIWVTAKYQGLKSGTFFWPGSDVEINGIFPDIYKMYNGSVPFEERILAVLQWLQLP
KDERPHFYTLYLEEPDSSGHSYGPVSSEVIKALQRVDGMVGMLMDGLKELNLHRCLNLIL
ISDHGMEQGSCKKYIYLNKYLGDVKNIKVIYGPAARLRPSDVPDKYYSFNYEGIARNLSC
REPNQHFKPYLKHFLPKRLHFAKSDRIEPLTFYLDPQWQLALNPSERKYCGSGFHGSDNV
FSNMQALFVGYGPGFKHGIEADTFENIEVYNLMCDLLNLTPAPNNGTHGSLNHLLKNPVY
TPKHPKEVHPLVQCPFTRNPRDNLGCSCNPSILPIEDFQTQFNLTVAEEKIIKHETLPYG
RPRVLQKENTICLLSQHQFMSGYSQDILMPLWTSYTVDRNDSFSTEDFSNCLYQDFRIPL
SPVHKCSFYKNNTKVSYGFLSPPQLNKNSSGIYSEALLTTNIVPMYQSFQVIWRYFHDTL
LRKYAEERNGVNVVSGPVFDFDYDGRCDSLENLRQKRRVIRNQEILIPTHFFIVLTSCKD
TSQTPLHCENLDTLAFILPHRTDNSESCVHGKHDSSWVEELLMLHRARITDVEHITGLSF
YQQRKEPVSDILKLKTHLPTFSQED
>hsa:5168
MARRSSFQSCQIISLFTFAVGVNICLGFTAHRIKRAEGWEEGPPTVLSDSPWTNISGSCK
GRCFELQEAGPPDCRCDNLCKSYTSCCHDFDELCLKTARGWECTKDRCGEVRNEENACHC
SEDCLARGDCCTNYQVVCKGESHWVDDDCEEIKAAECPAGFVRPPLIIFSVDGFRASYMK
KGSKVMPNIEKLRSCGTHSPYMRPVYPTKTFPNLYTLATGLYPESHGIVGNSMYDPVFDA
TFHLRGREKFNHRWWGGQPLWITATKQGVKAGTFFWSVVIPHERRILTILQWLTLPDHER
PSVYAFYSEQPDFSGHKYGPFGPEMTNPLREIDKIVGQLMDGLKQLKLHRCVNVIFVGDH
GMEDVTCDRTEFLSNYLTNVDDITLVPGTLGRIRSKFSNNAKYDPKAIIANLTCKKPDQH
FKPYLKQHLPKRLHYANNRRIEDIHLLVERRWHVARKPLDVYKKPSGKCFFQGDHGFDNK
VNSMQTVFVGYGSTFKYKTKVPPFENIELYNVMCDLLGLKPAPNNGTHGSLNHLLRTNTF
RPTMPEEVTRPNYPGIMYLQSDFDLGCTCDDKVEPKNKLDELNKRLHTKGSTEERHLLYG
RPAVLYRTRYDILYHTDFESGYSEIFLMPLWTSYTVSKQAEVSSVPDHLTSCVRPDVRVS

PSFSQNCLAYKNDKQMSYGFLFPPYLSSSPEAKYDAFLVTNMVPMYPAFKRVWNYFQRVL
VKKYASERNGVNVISGPIFDYDYDGLHDTEDKIKQYVEGSSIPVPTHYYSIITSCLDFTQ
PADKCDGPLSVSSFILPHRPDNEESCNSSEDESKWVEELMKMHTARVRDIEHLTSLDFFR
KTSRSYPEILTLKTYLHTYESEI
>hsa:5169
MESTLTLATEQPVKKNTLKKYKIACIVLLALLVIMSLGLGLGLGLRKLEKQGSCRKKCFD
ASFRGLENCRCDVACKDRGDCCWDFEDTCVESTRIWMCNKFRCGETRLEASLCSCSDDCL
QRKDCCADYKSVCQGETSWLEENCDTAQQSQCPEGFDLPPVILFSMDGFRAEYLYTWDTL
MPNINKLKTCGIHSKYMRAMYPTKTFPNHYTIVTGLYPESHGIIDNNMYDVNLNKNFSLS
SKEQNNPAWWHGQPMWLTAMYQGLKAATYFWPGSEVAINGSFPSIYMPYNGSVPFEERIS
TLLKWLDLPKAERPRFYTMYFEEPDSSGHAGGPVSARVIKALQVVDHAFGMLMEGLKQRN
LHNCVNIILLADHGMDQTYCNKMEYMTDYFPRINFFYMYEGPAPRIRAHNIPHDFFSFNS
EEIVRNLSCRKPDQHFKPYLTPDLPKRLHYAKNVRIDKVHLFVDQQWLAVRSKSNTNCGG
GNHGYNNEFRSMEAIFLAHGPSFKEKTEVEPFENIEVYNLMCDLLRIQPAPNNGTHGSLN
HLLKVPFYEPSHAEEVSKFSVCGFANPLPTESLDCFCPHLQNSTQLEQVNQMLNLTQEEI
TATVKVNLPFGRPRVLQKNVDHCLLYHREYVSGFGKAMRMPMWSSYTVPQLGDTSPLPPT
VPDCLRADVRVPPSESQKCSFYLADKNITHGFLYPPASNRTSDSQYDALITSNLVPMYEE
FRKMWDYFHSVLLIKHATERNGVNVVSGPIFDYNYDGHFDAPDEITKHLANTDVPIPTHY
FVVLTSCKNKSHTPENCPGWLDVLPFIIPHRPTNVESCPEGKPEALWVEERFTAHIARVR
DVELLTGLDFYQDKVQPVSEILQLKTYLPTFETTI
>hsa:51727
MLSRCRSGLLHVLGLSFLLQTRRPILLCSPRLMKPLVVFVLGGPGAGKGTQCARIVEEMD
QTMAANAQKNKFLIDGFPRNQDNLQGWNKTMDGKADVSFVLFFDCNNEICIERCLERGKS
SGRSDDNRESLEKRIQTYLQSTKPIIDLYEEMGKVKKIDASKSVDEVFDEVVQIFDKEG
>hsa:52
MAEQATKSVLFVCLGNICRSPIAEAVFRKLVTDQNISENWRVDSAATSGYEIGNPPDYRG
QSCMKRHGIPMSHVARQVPSLDLKLCVLCFSGSLTAVLFLTGTWAGPQTQEL
>hsa:5294
MELENYKQPVVLREDNCRRRRRMKPRSAAASLSSMELIPIEFVLPTSQRKCKSPETALLH
VAGHGNVEQMKAQVWLRALETSVAADFYHRLGPHHFLLLYQKKGQWYEIYDKYQVVQTLD
CLRYWKATHRSPGQIHLVQRHPPSEESQAFQRQLTALIGYDVTDVSNVHDDELEFTRRGL
VTPRMAEVASRDPKLYAMHPWVTSKPLPEYLWKKIANNCIFIVIHRSTTSQTIKVSPDDT
PGAILQSFFTKMAKKKSLMDIPESQSEQDFVLRVCGRDEYLVGETPIKNFQWVRHCLKNG
EEIHVVLDTPPDPALDEVRKEEWPLVDDCTGVTGYHEQLTIHGKDHESVFTVSLWDCDRK
FRVKIRGIDIPVLPRNTDLTVFVEANIQHGQQVLCQRRTSPKPFTEEVLWNVWLEFSIKI
KDLPKGALLNLQIYCGKAPALSSKASAESPSSESKGKVQLLYYVNLLLIDHRFLLRRGEY
VLHMWQISGKGEDQGSFNADKLTSATNPDKENSMSISILLDNYCHPIALPKHQPTPDPEG
DRVRAEMPNQLRKQLEAIIATDPLNPLTAEDKELLWHFRYESLKHPKAYPKLFSSVKWGQ
QEIVAKTYQLLARREVWDQSALDVGLTMQLLDCNFSDENVRAIAVQKLESLEDDDVLHYL
LQLVQAVKFEPYHDSALARFLLKRGLRNKRIGHFLFWFLRSEIAQSRHYQQRFAVILEAY
LRGCGTAMLHDFTQQVQVIEMLQKVTLDIKSLSAEKYDVSSQVISQLKQKLENLQNSQLP
ESFRVPYDPGLKAGALAIEKCKVMASKKKPLWLEFKCADPTALSNETIGIIFKHGDDLRQ
DMLILQILRIMESIWETESLDLCLLPYGCISTGDKIGMIEIVKDATTIAKIQQSTVGNTG
AFKDEVLNHWLKEKSPTEEKFQAAVERFVYSCAGYCVATFVLGIGDRHNDNIMITETGNL

FHIDFGHILGNYKSFLGINKERVPFVLTPDFLFVMGTSGKKTSPHFQKFQDICVKAYLAL
RHHTNLLIIILFSMMLMTGMPQLTSKEDIEYIRDALTVGKNEEDAKKYFLDQIEVCRDKGW
TVQFNWFLHLVLGIKQGEKHSA
>hsa:53
MAGKRSGWSRAALLQLLLGVNLVVMPPTRARSLRFVTLLYRHGDRSPVKTYPKDPYQEEE
WPQGFGQLTKEGMLQHWELGQALRQRYHGFLNTSYHRQEVYVRSTDFDRTLMSAEANLAG
LFPPNGMQRFNPNISWQPIPVHTVPITEDRVRVASPSLGW
>hsa:5319
MKLLVLAVLLTVAAADSGISPRAVWQFRKMIKCVIPGSDPFLEYNNYGCYCGLGGSGTPV
DELDKCCQTHDNCYDQAKKLDSCKFLLDNPYTHTYSYSCSGSAITCSSKNKECEAFICNC
DRNAAICFSKAPYNKAHKNLDTKKYCQS
>hsa:5320
MKTLLLLAVIMIFGLLQAHGNLVNFHRMIKLTTGKEAALSYGFYGCHCGVGGRGSPKDAT
DRCCVTHDCCYKRLEKRGCGTKFLSYKFSNSGSRITCAKQDSCRSQLCECDKAAATCFAR
NKTTYNKKYQYYSNKHCRGSTPRC
>hsa:5321
MSFIDPYQHIIVEHQYSHKFTVVVLRATKVTKGAFGDMLDTPDPYVELFISTTPDSRKRT
RHFNNDINPVWNETFEFILDPNQENVLEITLMDANYVMDETLGTATFTVSSMKVGEKKEV
PFIFNQVTEMVLEMSLEVCSCPDLRFSMALCDQEKTFRQQRKEHIRESMKKLLGPKNSEG
LHSARDVPVVAILGSGGGFRAMVGFSGVMKALYESGILDCATYVAGLSGSTWYMSTLYSH
PDFPEKGPEEINEELMKNVSHNPLLLLTPQKVKRYVESLWKKKSSGQPVTFTDIFGMLIG
ETLIHNRMNTTLSSLKEKVNTAQCPLPLFTCLHVKPDVSELMFADWVEFSPYEIGMAKYG
TFMAPDLFGSKFFMGTVVKKYEENPLHFLMGVWGSAFSILFNRVLGVSGSQSRGSTMEEE
LENITTKHIVSNDSSDSDDESHEPKGTENEDAGSDYQSDNQASWIHRMIMALVSDSALFN
TREGRAGKVHNFMLGLNLNTSYPLSPLSDFATQDSFDDDELDAAVADPDEFERIYEPLDV
KSKKIHVVDSGLTFNLPYPLILRPQRGVDLIISFDFSARPSDSSPPFKELLLAEKWAKMN
KLPFPKIDPYVFDREGLKECYVFKPKNPDMEKDCPTIIHFVLANINFRKYKAPGVPRETE
EEKEIADFDIFDDPESPFSTFNFQYPNQAFKRLHDLMHFNTLNNIDVIKEAMVESIEYRR
QNPSRCSVSLSNVEARRFFNKEFLSKPKA
>hsa:5322
MKGLLPLAWFLACSVPAVQGGLLDLKSMIEKVTGKNALTNYGFYGCYCGWGGRGTPKDGT
DWCCWAHDHCYGRLEEKGCNIRTQSYKYRFAWGVVTCEPGPFCHVNLCACDRKLVYCLKR
NLRSYNPQYQYFPNILCS
>hsa:5327
MDAMKRGLCCVLLLCGAVFVSPSQEIHARFRRGARSYQVICRDEKTQMIYQQHQSWLRPV
LRSNRVEYCWCNSGRAQCHSVPVKSCSEPRCFNGGTCQQALYFSDFVCQCPEGFAGKCCE
IDTRATCYEDQGISYRGTWSTAESGAECTNWNSSALAQKPYSGRRPDAIRLGLGNHNYCR
NPDRDSKPWCYVFKAGKYSSEFCSTPACSEGNSDCYFGNGSAYRGTHSLTESGASCLPWN
SMILIGKVYTAQNPSAQALGLGKHNYCRNPDGDAKPWCHVLKNRRLTWEYCDVPSCSTCG
LRQYSQPQFRIKGGLFADIASHPWQAAIFAKHRRSPGERFLCGGILISSCWILSAAHCFQ
ERFPPHHLTVILGRTYRVVPGEEEQKFEVEKYIVHKEFDDDTYDNDIALLQLKSDSSRCA
QESSVVRTVCLPPADLQLPDWTECELSGYGKHEALSPFYSERLKEAHVRLYPSSRCTSQH
LLNRTVTDNMLCAGDTRSGGPQANLHDACQGDSGGPLVCLNDGRMTLVGIISWGLGCGQK
DVPGVYTKVTNYLDWIRDNMRP

>hsa:5328
MVFHLRTRYEQANCDCLNGGTCVSNKYFSNIHWCNCPKKFGGQHCEIDKSKTCYEGNGHF
YRGKASTDTMGRPCLPWNSATVLQQTYHAHRSDALQLGLGKHNYCRNPDNRRRPWCYVQV
GLKPLVQECMVHDCADGKKPSSPPEELKFQCGQKTLRPRFKIIGGEFTTIENQPWFAAIY
RRHRGGSVTYVCGGSLISPCWVISATHCFIDYPKKEDYIVYLGRSRLNSNTQGEMKFEVE
NLILHKDYSADTLAHHNDIALLKIRSKEGRCAQPSRTIQTICLPSMYNDPQFGTSCEITG
FGKENSTDYLYPEQLKMTVVKLISHRECQQPHYYGSEVTTKMLCAADPQWKTDSCQGDSG
GPLVCSLQGRMTLTGIVSWGRGCALKDKPGVYTRVSHFLPWIRSHTKEENGLAL
>hsa:5330
MSLLNPVLLPPKVKAYLSQGERFIKWDDETTVASPVILRVDPKGYYLYWTYQSKEMEFLD
ITSIRDTRFGKFAKMPKSQKLRDVFNMDFPDNSFLLKTLTVVSGPDMVDLTFHNFVSYKE
NVGKAWAEDVLALVKHPLTANASRSTFLDKILVKLKMQLNSEGKIPVKNFFQMFPADRKR
VEAALSACHLPKGKNDAINPEDFPEPVYKSFLMSLCPRPEIDEIFTSYHAKAKPYMTKEH
LTKFINQKQRDSRLNSLLFPPARPDQVQGLIDKYEPSGINAQRGQLSPEGMVWFLCGPEN
SVLAQDKLLLHHDMTQPLNHYFINSSHNTYLTAGQFSGLSSAEMYRQVLLSGCRCVELDC
WKGKPPDEEPIITHGFTMTTDIFFKEAIEAIAESAFKTSPYPIILSFENHVDSPRQQAKM
AEYCRTIFGDMLLTEPLEKFPLKPGVPLPSPEDLRGKILIKNKKNQFSGPTSSSKDTGGE
AEGSSPPSAPAGEGTVWAGEEGTELEEEEVEEEEEEESGNLDEEEIKKMQSDEGTAGLEV
TAYEEMSSLVNYIQPTKFVSFEFSAQKNRSYVISSFTELKAYDLLSKASVQFVDYNKRQM
SRIYPKGTRMDSSNYMPQMFWNAGCQMVALNFQTMDLPMQQNMAVFEFNGQSGYLLKHEF
MRRPDKQFNPFSVDRIDVVVATTLSITVISGQFLSERSVRTYVEVELFGLPGDPKRRYRT
KLSPSTNSINPVWKEEPFVFEKILMPELASLRVAVMEEGNKFLGHRIIPINALNSGYHHL
CLHSESNMPLTMPALFIFLEMKDYIPGAWADLTVALANPIKFFSAHDTKSVKLKEAMGGL
PEKPFPLASPVASQVNGALAPTSNGSPAARAGAREEAMKEAAEPRTASLEELRELKGVVK
LQRRHEKELRELERRGARRWEELLQRGAAQLAELGPPGVGGVGACKLGPGKGSRKKRSLP
REESAGAAPGEGPEGVDGRVRELKDRLELELLRQGEEQYECVLKRKEQHVAEQISKMMEL
AREKQAAELKALKETSENDTKEMKKKLETKRLERIQGMTKVTTDKMAQERLKREINNSHI
QEVVQVIKQMTENLERHQEKLEEKQAACLEQIREMEKQFQKEALAEYEARMKGLEAEVKE
SVRACLRTCFPSEAKDKPERACECPPELCEQDPLIAKADAQESRL
>hsa:5331
MAGAQPGVHALQLEPPTVVETLRRGSKFIKWDEETSSRNLVTLRVDPNGFFLYWTGPNME
VDTLDISSIRDTRTGRYARLPKDPKIREVLGFGGPDARLEEKLMTVVSGPDPVNTVFLNF
MAVQDDTAKVWSEELFKLAMNILAQNASRNTFLRKAYTKLKLQVNQDGRIPVKNILKMFS
ADKKRVETALESCGLKFNRSESIRPDEFSLEIFERFLNKLCLRPDIDKILLEIGAKGKPY
LTLEQLMDFINQKQRDPRLNEVLYPPLRPSQARLLIEKYEPNQQFLERDQMSMEGFSRYL
GGEENGILPLEALDLSTDMTQPLSAYFINSSHNTYLTAGQLAGTSSVEMYRQALLWGCRC
VELDVWKGRPPEEEPFITHGFTMTTEVPLRDVLEAIAETAFKTSPYPVILSFENHVDSAK
QQAKMAEYCRSIFGDALLIEPLDKYPLAPGVPLPSPQDLMGRILVKNKKRHRPSAGGPDS
AGRKRPLEQSNSALSESSAATEPSSPQLGSPSSDSCPGLSNGEEVGLEKPSLEPQKSLGD
EGLNRGPYVLGPADREDEEEDEEEEEQTDPKKPTTDEGTASSEVNATEEMSTLVNYIEPV
KFKSFEAARKRNKCFEMSSFVETKAMEQLTKSPMEFVEYNKQQLSRIYPKGTRVDSSNYM
PQLFWNVGCQLVALNFQTLDVAMQLNAGVFEYNGRSGYLLKPEFMRRPDKSFDPFTEVIV
DGIVANALRVKVISGQFLSDRKVGIYVEVDMFGLPVDTRRKYRTRTSQGNSFNPVWDEEP
FDFPKVVLPTLASLRIAAFEEGGKFVGHRILPVSAIRSGYHYVCLRNEANQPLCLPALLI

YTEASDYIPDDHQDYAEALINPIKHVSLMDQRARQLAALIGESEAQAGQETCQDTQSQQL
GSQPSSNPTPSPLDASPRRPPGPTTSPASTSLSSPGQRDDLIASILSEVAPTPLDELRGH
KALVKLRSRQERDLRELRKKHQRKAVTLTRRLLDGLAQAQAEGRCRLRPGALGGAADVED
TKEGEDEAKRYQEFQNRQVQSLLELREAQVDAEAQRRLEHLRQALQRLREVVLDANTTQF
KRLKEMNEREKKELQKILDRKRHNSISEAKMRDKHKKEAELTEINRRHITESVNSIRRLE
EAQKQRHDRLVAGQQQVLQQLAEEEPKLLAQLAQECQEQRARLPQEIRRSLLGEMPEGLG
DGPLVACASNGHAPGSSGHLSGADSESQEENTQL

>hsa:5332

MAKPYEFNWQKEVPSFLQEGAVFDRYEEESFVFEPNCLFKVDEFGFFLTWRSEGKEGQVL
ECSLINSIRSGAIPKDPKILAALEAVGKSENDLEGRIVCVCSGTDLVNISFTYMVAENPE
VTKQWVEGLRSIIHNFRANNVSPMTCLKKHWMKLAFMTNTNGKIPVRSITRTFASGKTEK
VIFQALKELGLPSGKNDEIEPTAFSYEKFYELTQKICPRTDIEDLFKKINGDKTDYLTVD
QLVSFLNEHQRDPRLNEILFPFYDAKRAMQIIEMYEPDEDLKKKGLISSDGFCRYLMSDE
NAPVFLDRLELYQEMDHPLAHYFISSSHNTYLTGRQFGGKSSVEMYRQVLLAGCRCVELD
CWDGKGEDQEPIITHGKAMCTDILFKDVIQAIKETAFVTSEYPVILSFENHCSKYQQYKM
SKYCEDLFGDLLLKQALESHPLEPGRALPSPNDLKRKILIKNKRLKPEVEKKQLEALRSM
MEAGESASPANILEDDNEEEIESADQEEEAHPEFKFGNELSADDLGHKEAVANSVKKGLV
TVEDEQAWMASYKYVGATTNIHPYLSTMINYAQPVKFQGFHVAEERNIHYNMSSFNESVG
LGYLKTHAIEFVNYNKRQMSRIYPKGGRVDSSNYMPQIFWNAGCQMVSLNYQTPDLAMQL
NQGKFEYNGSCGYLLKPDFMRRPDRTFDPFSETPVDGVIAATCSVQVISGQFLSDKKIGT
YVEVDMYGLPTDTIRKEFRTRMVMNNGLNPVYNEESFVFRKVILPDLAVLRIAVYDDNNK
LIGQRILPLDGLQAGYRHISLRNEGNKPLSLPTIFCNIVLKTYVPDGFGDIVDALSDPKK
FLSITEKRADQMRAMGIETSDIADVPSDTSKNDKKGKANTAKANVTPQSSSELRPTTTAA
LASGVEAKKGIELIPQVRIEDLKQMKAYLKHLKKQQKELNSLKKKHAKEHSTMQKLHCTQ
VDKIVAQYDKEKSTHEKILEKAMKKKGGSNCLEMKKETEIKIQTLTSDHKSKVKEIVAQH
TKEWSEMINTHSAEEQEIRDLHLSQQCELLKKLLINAHEQQTQQLKLSHDRESKEMRAHQ
AKISMENSKAISQDKSIKNKAERERRVRELNSSNTKKFLEERKRLAMKQSKEMDQLKKVQ
LEHLEFLEKQNEQLLKSCHAVSQTQGEGDAADGEIGSRDGPQTSNSSMKLQNAN

>hsa:5333

MQCLGIRSRSRSRELYLQERSLKVAALNGRRLGLQDDEDLQALLKGSQLLKVKSSSWRRE
RFYKLQEDCKTIWQESRKVMRTPESQLFSIEDIQEVRMGHRTEGLEKFARDVPEDRCFSI
VFKDQRNTLDLIAPSPADAQHWVLGLHKIIHHSGSMDQRQKLQHWIHSCLRKADKNKDNK
MSFKELQNFLKELNIQVDDSYARKIFRECDHSQTDSLEDEEIEAFYKMLTQRVEIDRTFA
EAAGSGETLSVDQLVTFLQHQQREEAAGPALALSLIERYEPSETAKAQRQMTKDGFLMYL
LSADGSAFSLAHRRVYQDMGQPLSHYLVSSSHNTYLLEDQLAGPSSTEAYIRALCKGCRC
LELDCWDGPNQEPIIYHGYTFTSKILFCDVLRAIRDYAFKASPYPVILSLENHCTLEQQR
VMARHLHAILGPMLLNRPLDGVTNSLPSPEQLKGKILLKGKKLGGLLPPGGEGGPEATVV
SDEDEAAEMEDEAVRSRVQHKPKEDKLRLAQELSDMVIYCKSVHFGGFSSPGTPGQAFYE
MASFSENRALRLLQESGNGFVRHNVGHLSRIYPAGWRTDSSNYSPVEMWNGGCQIVALNF
QTPGPEMDVYQGRFQDNGACGYVLKPAFLRDPNGTFNPRALAQGPWWARKRLNIRVISGQ
QLPKVNKNKNSIVDPKVTVEIHGVSRDVASRQTAVITNNGFNPWWDTEFAFEVVVPDLAL
IRFLVEDYDASSKNDFIGQSTIPLNSLKQGYRHVHLMSKNGDQHPSATLFVKISLQD

>hsa:5335

MAGAASPCANGCGPGAPSDAEVLHLCRSLEVGTVMTLFYSKKSQRPERKTFQVKLETRQI

TWSRGADKIEGAIDIREIKEIRPGKTSRDFDRYQEDPAFRPDQSHCFVILYGMEFRLKTL
SLQATSEDEVNMWIKGLTWLMEDTLQAPTPLQIERWLRKQFYSVDRNREDRISAKDLKNM
LSQVNYRVPNMRFLRERLTDLEQRSGDITYGQFAQLYRSLMYSAQKTMDLPFLEASTLRA
GERPELCRVSLPEFQQFLLDYQGELWAVDRLQVQEFMLSFLRDPLREIEEPYFFLDEFVT
FLFSKENSVWNSQLDAVCPDTMNNPLSHYWISSSHNTYLTGDQFSSESSLEAYARCLRMG
CRCIELDCWDGPDGMPVIYHGHTLTTKIKFSDVLHTIKEHAFVASEYPVILSIEDHCSIA
QQRNMAQYFKKVLGDTLLTKPVEISADGLPSPNQLKRKILIKHKKLAEGSAYEEVPTSMM
YSENDISNSIKNGILYLEDPVNHEWYPHYFVLTSSKIYYSEETSSDQGNEDEEEPKEVSS
STELHSNEKWFHGKLGAGRDGRHIAERLLTEYCIETGAPDGSFLVRESETFVGDYTLSFW
RNGKVQHCRIHSRQDAGTPKFFLTDNLVFDSLYDLITHYQQVPLRCNEFEMRLSEPVPQT
NAHESKEWYHASLTRAQAEHMLMRVPRDGAFLVRKRNEPNSYAISFRAEGKIKHCRVQQE
GQTVMLGNSEFDSLVDLISYYEKHPLYRKMKLRYPINEEALEKIGTAEPDYGALYEGRNP
GFYVEANPMPTFKCAVKALFDYKAQREDELTFIKSAIIQNVEKQEGGWWRGDYGGKKQLW
FPSNYVEEMVNPVALEPEREHLDENSPLGDLLRGVLDVPACQIAIRPEGKNNRLFVFSIS
MASVAHWSLDVAADSQEELQDWVKKIREVAQTADARLTEGKIMERRKKIALELSELVVYC
RPVPFDEEKIGTERACYRDMSSFPETKAEKYVNKAKGKKFLQYNRLQLSRIYPKGQRLDS
SNYDPLPMWICGSQLVALNFQTPDKPMQMNQALFMTGRHCGYVLQPSTMRDEAFDPFDKS
SLRGLEPCAISIEVLGARHLPKNGRGIVCPFVEIEVAGAEYDSTKQKTEFVVDNGLNPVW
PAKPFHFQISNPEFAFLRFVVYEEDMFSDQNFLAQATFPVKGLKTGYRAVPLKNNYSEDL
ELASLLIKIDIFPAKQENGDLSPFSGTSLRERGSDASGQLFHGRAREGSFESRYQQPFED
FRISQEHLADHFDSRERRAPRRTRVNGDNRL

>hsa:5336

MSTTVNVDSLAEYEKSQIKRALELGTVMTVFSFRKSTPERRTVQVIMETRQVAWSKTADK
IEGFLDIMEIKEIRPGKNSKDFERAKAVRQKEDCCFTILYGTQFVLSTLSLAADSKEDAV
NWLSGLKILHQEAMNASTPTIIESWLRKQIYSVDQTRRNSISLRELKTILPLINFKVSSA
KFLKDKFVEIGAHKDELSFEQFHLFYKKLMFEQQKSILDEFKKDSSVFILGNTDRPDASA
VYLHDFQRFLIHEQQEHWAQDLNKVRERMTKFIDDTMRETAEPFLFVDEFLTYLFSRENS
IWDEKYDAVDMQDMNNPLSHYWISSSHNTYLTGDQLRSESSPEAYIRCLRMGCRCIELDC
WDGPDGKPVIYHGWTRTTKIKFDDVVQAIKDHAFVTSSFPVILSIEEHCSVEQQRHMAKA
FKEVFGDLLLTKPTEASADQLPSPSQLREKIIIKHKKLGPRGDVDVNMEDKKDEHKQQGE
LYMWDSIDQKWTRHYCAIADAKLSFSDDIEQTMEEEVPQDIPPTELHFGEKWFHKKVEKR
TSAEKLLQEYCMETGGKDGTFLVRESETFPNDYTLSFWRSGRVQHCRIRSTMEGGTLKYY
LTDNLTFSSIYALIQHYRETHLRCAEFELRLTDPVPNPNPHESKPWYYDSLSRGEAEDML
MRIPRDGAFLIRKREGSDSYAITFRARGKVKHCRINRDGRHFVLGTSAYFESLVELVSYY
EKHSLYRKMRLRYPVTPELLERYNMERDINSLYDVSRMYVDPSEINPSMPQRTVKALYDY
KAKRSDELSFCRGALIHNVSKEPGGWWKGDYGTRIQQYFPSNYVEDISTADFEELEKQII
EDNPLGSLCRGILDLNTYNVVKAPQGKNQKSFVFILEPKQQGDPPVEFATDRVEELFEWF
QSIREITWKIDTKENNMKYWEKNQSIAIELSDLVVYCKPTSKTKDNLENPDFREIRSFVE
TKADSIIRQKPVDLLKYNQKGLTRVYPKGQRVDSSNYDPFRLWLCGSQMVALNFQTADKY
MQMNHALFSLNGRTGYVLQPESMRTEKYDPMPPESQRKILMTLTVKVLGARHLPKLGRSI
ACPFVEVEICGAEYDNNKFKTTVVNDNGLSPIWAPTQEKVTFEIYDPNLAFLRFVVYEED
MFSDPNFLAHATYPIKAVKSGFRSVPLKNGYSEDIELASLLVFCEMRPVLESEEELYSSC
RQLRRRQEELNNQLFLYDTHQNLRNANRDALVKEFSVNENQLQLYQEKCNKRLREKRVSN
SKFYS

>hsa:5337
MSLKNEPRVNTSALQKIAADMSNIIENLDTRELHFEGEEVDYDVSPSDPKIQEVYIPFSA
IYNTQGFKEPNIQTYLSGCPIKAQVLEVERFTSTTRVPSINLYTIELTHGEFKWQVKRKF
KHFQEFHRELLKYKAFIRIPIPTRRHTFRRQNVREEPREMPSLPRSSENMIREEQFLGRR
KQLEDYLTKILKMPMYRNYHATTEFLDISQLSFIHDLGPKGIEGMIMKRSGGHRIPGLNC
CGQGRACYRWSKRWLIVKDSFLLYMKPDSGAIAFVLLVDKEFKIKVGKKETETKYGIRID
NLSRTLILKCNSYRHARWWGGAIEEFIQKHGTNFLKDHRFGSYAAIQENALAKWYVNAKG
YFEDVANAMEEANEEIFITDWWLSPEIFLKRPVVEGNRWRLDCILKRKAQQGVRIFIMLY
KEVELALGINSEYTKRTLMRLHPNIKVMRHPDHVSSTVYLWAHHEKLVIIDQSVAFVGGI
DLAYGRWDDNEHRLTDVGSVKRVTSGPSLGSLPPAAMESMESLRLKDKNEPVQNLPIQKS
IDDVDSKLKGIGKPRKFSKFSLYKQLHRHHLHDADSISSIDSTSNTGSIRSLQTGVGELH
GETRFWHGKDYCNFVFKDWVQLDKPFADFIDRYSTPRMPWHDIASAVHGKAARDVARHFI
QRWNFTKIMKSKYRSLSYPFLLPKSQTTAHELRYQVPGSVHANVQLLRSAADWSAGIKYH
EESIHAAYVHVIENSRHYIYIENQFFISCADDKVVFNKIGDAIAQRILKAHRENQKYRVY
VVIPLLPGFEGDISTGGGNALQAIMHFNYRTMCRGENSILGQLKAELGNQWINYISFCGL
RTHAELEGNLVTELIYVHSKLLIADDNTVIIGSANINDRSMLGKRDSEMAVIVQDTETVP
SVMDGKEYQAGRFARGLRLQCFRVVLGYLDDPSEDIQDPVSDKFFKEVWVSTAARNATIY
DKVFRCLPNDEVHNLIQLRDFINKPVLAKEDPIRAEEELKKIRGFLVQFPFYFLSEESLL
PSVGTKEAIVPMEVWT
>hsa:5338
MTATPESLFPTGDELDSSQLQMESDEVDTLKEGEDPADRMHPFLAIYELQSLKVHPLVFA
PGVPVTAQVVGTERYTSGSKVGTCTLYSVRLTHGDFSWTTKKKYRHFQELHRDLLRHKVL
MSLLPLARFAVAYSPARDAGNREMPSLPRAGPEGSTRHAASKQKYLENYLNRLLTMSFYR
NYHAMTEFLEVSQLSFIPDLGRKGLEGMIRKRSGGHRVPGLTCCGRDQVCYRWSKRWLVV
KDSFLLYMCLETGAISFVQLFDPGFEVQVGKRSTEARHGVRIDTSHRSLILKCSSYRQAR
WWAQEITELAQGPGRDFLQLHRHDSYAPPRPGTLARWFVNGAGYFAAVADAILRAQEEIF
ITDWWLSPEVYLKRPAHSDDWRLDIMLKRKAEEGVRVSILLFKEVELALGINSGYSKRAL
MLLHPNIKVMRHPDQVTLWAHHEKLLVVDQVVAFLGGLDLAYGRWDDLHYRLTDLGDSSE
SAASQPPTPRPDSPATPDLSHNQFFWLGKDYSNLITKDWVQLDRPFEDFIDRETTPRMPW
RDVGVVVHGLPARDLARHFIQRWNFTKTTKAKYKTPTYPYLLPKSTSTANQLPFTLPGGQ
CTTVQVLRSVDRWSAGTLENSILNAYLHTIRESQHFLYIENQFFISCSDGRTVLNKVGDE
IVDRILKAHKQGWCYRVYVLLPLLPGFEGDISTGGGNSIQAILHFTYRTLCRGEYSILHR
LKAAMGTAWRDYISICGLRTHGELGGHPVSELIYIHSKVLIADDRTVIIGSANINDRSLL
GKRDSELAVLIEDTETEPSLMNGAEYQAGSVILGANTRPDLDLRDPICDDFFQLWQDMAE
SNANIYEQIFRCLPSNATRSLRTLREYVAVEPLATVSPPLARSELTQVQGHLVHFPLKFL
EDESLLPPLGSKEGMIPLEVWT
>hsa:5340
MEHKEVVLLLLLFLKSGQGEPLDDYVNTQGASLFSVTKKQLGAGSIEECAAKCEEDEEFT
CRAFQYHSKEQQCVIMAENRKSSIIIRMRDVVLFEKKVYLSECKTGNGKNYRGTMSKTKN
GITCQKWSSTSPHRPRFSPATHPSEGLEENYCRNPDNDPQGPWCYTTDPEKRYDYCDILE
CEEECMHCSGENYDGKISKTMSGLECQAWDSQSPHAHGYIPSKFPNKNLKKNYCRNPDRE
LRPWCFTTDPNKRWELCDIPRCTTPPPSSGPTYQCLKGTGENYRGNVAVTVSGHTCQHWS
AQTPHTHNRTPENFPCKNLDENYCRNPDGKRAPWCHTTNSQVRWEYCKIPSCDSSPVSTE
QLAPTAPPELTPVVQDCYHGDGQSYRGTSSTTTTGKKCQSWSSMTPHRHQKTPENYPNAG

LTMNYCRNPDADKGPWCFTTDPSVRWEYCNLKKCSGTEASVVAPPPVVLLPDVETPSEED
CMFGNGKGYRGKRATTVTGTPCQDWAAQEPHRHSIFTPETNPRAGLEKNYCRNPDGDVGG
PWCYTTNPRKLYDYCDVPQCAAPSFDCGKPQVEPKKCPGRVVGGCVAHPHSWPWQVSLRT
RFGMHFCGGTLISPEWVLTAAHCLEKSPRPSSYKVILGAHQEVNLEPHVQEIEVSRLFLE
PTRKDIALLKLSSPAVITDKVIPACLPSPNYVVADRTECFITGWGETQGTFGAGLLKEAQ
LPVIENKVCNRYEFLNGRVQSTELCAGHLAGGTDSCQGDSGGPLVCFEKDKYILQGVTSW
GLGCARPNKPGVYVRVSRFVTWIEGVMRNN
>hsa:5351
MRPLLLLALLGWLLLAEAKGDAKPEDNLLVLTVATKETEGFRRFKRSAQFFNYKIQALGL
GEDWNVEKGTSAGGGQKVRLLKKALEKHADKEDLVILFADSYDVLFASGPRELLKKFRQA
RSQVVFSAEELIYPDRRLETKYPVVSDGKRFLGSGGFIGYAPNLSKLVAEWEGQDSDSDQ
LFYTKIFLDPEKREQINITLDHRCRIFQNLDGALDEVVLKFEMGHVRARNLAYDTLPVLI
HGNGPTKLQLNYLGNYIPRFWTFETGCTVCDEGLRSLKGIGDEALPTVLVGVFIEQPTPF
VSLFFQRLLRLHYPQKHMRLFIHNHEQHHKAQVEEFLAQHGSEYQSVKLVGPEVRMANAD
ARNMGADLCRQDRSCTYYFSVDADVALTEPNSLRLLIQQNKNVIAPLMTRHGRLWSNFWG
ALSADGYYARSEDYVDIVQGRRVGVWNVPYISNIYLIKGSALRGELQSSDLFHHSKLDPD
MAFCANIRQQDVFMFLTNRHTLGHLLSLDSYRTTHLHNDLWEVFSNPEDWKEKYIHQNYT
KALAGKLVETPCPDVYWFPIFTEVACDELVEEMEHFGQWSLGNNKDNRIQGGYENVPTID
IHMNQIGFEREWHKFLLEYIAPMTEKLYPGYYTRAQFDLAFVVRYKPDEQPSLMPHHDAS
TFTINIALNRVGVDYEGGGCRFLRYNCSIRAPRKGWTLMHPGRLTHYHEGLPTTRGTRYI
AVSFVDP
>hsa:53938
MSVTLHTDVGDIKIEVFCERTPKTCEMESRCVPQAGVQWRDLGSLQPPPPGFKQVFCLSL
PRTGRGGNSIWGKKFEDEYSEYLKHNVRGVVSMANNGPNTNGSQFFITYGKQPHLDMKYT
VFGKVIDGLETLDELEKLPVNEKTYRPLNDVHIKDITIHANPFAQ
>hsa:54
MDMWTALLILQALLLPSLADGATPALRFVAVGDWGGVPNAPFHTAREMANAKEIARTVQI
LGADFILSLGDNFYFTGVQDINDKRFQETFEDVFSDRSLRKVPWYVLAGNHDHLGNVSAQ
IAYSKISKRWNFPSPFYRLHFKIPQTNVSVAIFMLDTVTLCGNSDDFLSQQPERPRDVKL
ARTQLSWLKKQLAAAREDYVLVAGHYPVWSIAEHGPTHCLVKQLRPLLATYGVTAYLCGH
DHNLQYLQDENGVGYVLSGAGNFMDPSKRHQRKVPNGYLRFHYGTEDSLGGFAYVEISSK
EMTVTYIEASGKSLFKTRLPRRARP
>hsa:5406
MLPLWTLSLLLGAVAGKEVCYERLGCFSDDSPWSGITERPLHILPWSPKDVNTRFLLYTN
ENPNNFQEVAADSSSISGSNFKTNRKTRFIIHGFIDKGEENWLANVCKNLFKVESVNCIC
VDWKGGSRTGYTQASQNIRIVGAEVAYFVEFLQSAFGYSPSNVHVIGHSLGAHAAGEAGR
RTNGTIGRITGLDPAEPCFQGTPELVRLDPSDAKFVDVIHTDGAPIVPNLGFGMSQVVGH
LDFFPNGGVEMPGCKKNILSQIVDIDGIWEGTRDFAACNHLRSYKYYTDSIVNPDGFAGF
PCASYNVFTANKCFPCPSGGCPQMGHYADRYPGKTNDVGQKFYLDTGDASNFARWRYKVS
VTLSGKKVTGHILVSLFGNKGNSKQYEIFKGTLKPDSTHSNEFDSDVDVGDLQMVKFIWY
NNVINPTLPRVGASKIIVETNVGKQFNFCSPETVREEVLLTLTPC
>hsa:5407
MLIFWTITLFLLGAAKGKEVCYEDLGCFSDTEPWGGTAIRPLKILPWSPEKIGTRFLLYT
NENPNNFQILLLSDPSTIEASNFQMDRKTRFIIHGFIDKGDESWVTDMCKKLFEVEEVNC

ICVDWKKGSQATYTQAANNVRVVGAQVAQMLDILLTEYSYPPSKVHLIGHSLGAHVAGEA
GSKTPGLSRITGLDPVEASFESTPEEVRLDPSDADFVDVIHTDAAPLIPFLGFGTNQQMG
HLDFFPNGGESMPGCKKNALSQIVDLDGIWAGTRDFVACNHLRSYKYYLESILNPDGFAA
YPCTSYKSFESDKCFPCPDQGCPQMGHYADKFAGRTSEEQQKFFLNTGEASNFARWRYGV
SITLSGRTATGQIKVALFGNKGNTHQYSIFRGILKPGSTHSYEFDAKLDVGTIEKVKFLW
NNNVINPTLPKVGATKITVQKGEEKTVYNFCSEDTVREDTLLTLTPC

>hsa:5408

MMLPPWTLGLLLLATVRGKEVCYGQLGCFSDEKPWAGTLQRPVKLLPWSPEDIDTRFLLY
TNENPNNFQLITGTEPDTIEASNFQLDRKTRFIIHGFLDKAEDSWPSDMCKKMFEVEKVN
CICVDWRHGSRAMYTQAVQNIRVVGAETAFLIQALSTQLGYSLEDVHVIGHSLGAHTAAE
AGRRLGGRVGRITGLDPAGPCFQDEPEEVRLDPSDAVFVDVIHTDSSPIVPSLGFGMSQK
VGHLDFFPNGGKEMPGCKKNVLSTITDIDGIWEGIGGFVSCNHLRSFEYYSSSVLNPDGF
LGYPCASYDEFQESKCFPCPAEGCPKMGHYADQFKGKTSAVEQTFFLNTGESGNFTSWRY
KVSVTLSGKEKVNGYIRIALYGSNENSKQYEIFKGSLKPDASHTCAIDVDFNVGKIQKVK
FLWNKRGINLSEPKLGASQITVQSGEDGTEYNFCSSDTVEENVLQSLYPC

>hsa:5422

MAPVHGDDSLSDSGSFVSSRARREKKSKKGRQEALERLKKAKAGEKYKYEVEDFTGVYEE
VDEEQYSKLVQARQDDDWIVDDDGIGYVEDGREIFDDDLEDDALDADEKGKDGKARNKDK
RNVKKLAVTKPNNIKSMFIACAGKKTADKAVDLSKDGLLGDILQDLNTETPQITPPPVMI
LKKKRSIGASPNPFSVHTATAVPSGKIASPVSRKEPPLTPVPLKRAEFAGDDVQVESTEE
EQESGAMEFEDGDFDEPMEVEEVDLEPMAAKAWDKESEPAEEVKQEADSGKGTVSYLGSF
LPDVSCWDIDQEGDSSFSVQEVQVDSSHLPLVKGADEEQVFHFYWLDAYEDQYNQPGVVF
LFGKVWIESAETHVSCCVMVKNIERTLYFLPREMKIDLNTGKETGTPISMKDVYEEFDEK
IATKYKIMKFKSKPVEKNYAFEIPDVPEKSEYLEVKYSAEMPQLPQDLKGETFSHVFGTN
TSSLELFLMNRKIKGPCWLEVKSPQLLNQPVSWCKVEAMALKPDLVNVIKDVSPPPLVVM
AFSMKTMQNAKNHQNEIIAMAALVHHSFALDKAAPKPPFQSHFCVVSKPKDCIFPYAFKE
VIEKKNVKVEVAATERTLLGFFLAKVHKIDPDIIVGHNIYGFELEVLLQRINVCKAPHWS
KIGRLKRSNMPKLGGRSGFGERNATCGRMICDVEISAKELIRCKSYHLSELVQQILKTER
VVIPMENIQNMYSESSQLLYLLEHTWKDAKFILQIMCELNVLPLALQITNIAGNIMSRTL
MGGRSERNEFLLLHAFYENNYIVPDKQIFRKPQQKLGDEDEEIDGDTNKYKKGRKKAAYA
GGLVLDPKVGFYDKFILLLDFNSLYPSIIQEFNICFTTVQRVASEAQKVTEDGEQEQIPE
LPDPSLEMGILPREIRKLVERRKQVKQLMKQQDLNPDLILQYDIRQKALKLTANSMYGCL
GFSYSRFYAKPLAALVTYKGREILMHTKEMVQKMNLEVIYGDTDSIMINTNSTNLEEVFK
LGNKVKSEVNKLYKLLEIDIDGVFKSLLLLKKKKYAALVVEPTSDGNYVTKQELKGLDIV
RRDWCDLAKDTGNFVIGQILSDQSRDTIVENIQKRLIEIGENVLNGSVPVSQFEINKALT
KDPQDYPDKKSLPHVHVALWINSQGGRKVKAGDTVSYVICQDGSNLTASQRAYAPEQLQK
QDNLTIDTQYYLAQQIHPVVARICEPIDGIDAVLIATWLGLDPTQFRVHHYHKDEENDAL
LGGPAQLTDEEKYRDCERFKCPCPTCGTENIYDNVFDGSGTDMEPSLYRCSNIDCKASPL
TFTVQLSNKLIMDIRRFIKKYYDGWLICEEPTCRNRTRHLPLQFSRTGPLCPACMKATLQ
PEYSDKSLYTQLCFYRYIFDAECALEKLTTDHEKDKLKKQFFTPKVLQDYRKLKNTAEQF
LSRSGYSEVNLSKLFAGCAVKS

>hsa:5423

MSKRKAPQETLNGGITDMLTELANFEKNVSQAIHKYNAYRKAASVIAKYPHKIKSGAEAK
KLPGVGTKIAEKIDEFLATGKLRKLEKIRQDDTSSSINFLTRVSGIGPSAARKFVDEGIK

TLEDLRKNEDKLNHHQRIGLKYFGDFEKRIPREEMLQMQDIVLNEVKKVDSEYIATVCGS
FRRGAESSGDMDVLLTHPSFTSESTKQPKLLHQVVEQLQKVHFITDTLSKGETKFMGVCQ
LPSKNDEKEYPHRRIDIRLIPKDQYYCGVLYFTGSDIFNKNMRAHALEKGFTINEYTIRP
LGVTGVAGEPLPVDSEKDIFDYIQWKYREPKDRSE
>hsa:5444
MAKLIALTLLGMGLALFRNHQSSYQTRLNALREVQPVELPNCNLVKGIETGSEDLEILPN
GLAFISSGLKYPGIKSFNPNSPGKILLMDLNEEDPTVLELGITGSKFDVSSFNPHGISTF
TDEDNAMYLLVVNHPDAKSTVELFKFQEEEKSLLHLKTIRHKLLPNLNDIVAVGPEHFYG
TNDHYFLDPYLQSWEMYLGLAWSYVVYYSPSEVRVVAEGFDFANGINISPDGKYVYIAEL
LAHKIHVYEKHANWTLTPLKSLDFNTLVDNISVDPETGDLWVGCHPNGMKIFFYDSENPP
ASEVLRIQNILTEEPKVTQVYAENGTVLQGSTVASVYKGKLLIGTVFHKALYCEL
>hsa:5445
MGRLVAVGLLGIALALLGERLLALRNRLKASREVESVDLPHCHLIKGIEAGSEDIDILPN
GLAFFSVGLKFPGLHSFAPDKPGGILMMDLKEEKPRARELRISRGFDLASFNPHGISTFI
DNDDTVYLFVVNHPEFKNTVEIFKFEEAENSLLHLKTVKHELLPSVNDITAVGPAHFYAT
NDHYFSDPFLKYLETYLNLHWANVVYYSPNEVKVVAEGFDSANGINISPDDKYIYVADIL
AHEIHVLEKHTNMNLTQLKVLELDTLVDNLSIDPSSGDIWVGCHPNGQKLFVYDPNNPPS
SEVLRIQNILSEKPTVTTVYANNGSVLQGSSVASVYDGKLLIGTLYHRALYCEL
>hsa:5447
MINMGDSHVDTSSTVSEAVAEEVSLFSMTDMILFSLIVGLLTYWFLFRKKKEEVPEFTKI
QTLTSSVRESSFVEKMKKTGRNIIVFYGSQTGTAEEFANRLSKDAHRYGMRGMSADPEEY
DLADLSSLPEIDNALVVFCMATYGEGDPTDNAQDFYDWLQETDVDLSGVKFAVFGLGNKT
YEHFNAMGKYVDKRLEQLGAQRIFELGLGDDDGNLEEDFITWREQFWPAVCEHFGVEATG
EESSIRQYELVVHTDIDAAKVYMGEMGRLKSYENQKPPFDAKNPFLAAVTTNRKLNQGTE
RHLMHLELDISDSKIRYESGDHVAVYPANDSALVNQLGKILGADLDVVMSLNNLDEESNK
KHPFPCPTSYRTALTYYLDITNPPRTNVLYELAQYASEPSEQELLRKMASSSGEGKELYL
SWVVEARRHILAILQDCPSLRPPIDHLCELLPRLQARYYSIASSSKVHPNSVHICAVVVE
YETKAGRINKGVATNWLRAKEPAGENGGRALVPMFVRKSQFRLPFKATTPVIMVGPGTGV
APFIGFIQERAWLRQQGKEVGETLLYYGCRRSDEDYLYREELAQFHRDGALTQLNVAFSR
EQSHKVYVQHLLKQDREHLWKLIEGGAHIYVCGDARNMARDVQNTFYDIVAELGAMEHAQ
AVDYIKKLMTKGRYSLDVWS
>hsa:54490
MALKWTSVLLLIHLGCYFSSGSCGKVLVWTGEYSHWMNMKTILKELVQRGHEVTVLASSA
SILFDPNDAFTLKLEVYPTSLTKTEFENIIMQQVKRWSDIQKDSFWLYFSQEQEILWEFH
DIFRNFCKDVVSNKKVMKKLQESRFDIIFADAFFPCGELLAALLNIPFVYSLCFTPGYTI
ERHSGGLIFPPSYIPVVMSKLSDQMTFMERVKNMIYVLYFDFWFQMCDMKKWDQFYSEVL
GRPTTLFETMGKADIWLMRNSWSFQFPHPFLPNIDFVGGLHCKPAKPLPKEMEEFVQSSG
ENGVVVFSLGSVISNMTAERANVIATALAKIPQKI
>hsa:54575
MARAGWTSPVPLCVCLLLTCGFAEAGKLLVVPMDGSHWFTMQSVVEKLILRGHEVVVVMP
EVSWQLERSLNCTVKTYSTSYTLEDQNREFMVFAHAQWKAQAQSIFSLLMSSSSGFLDLF
FSHCRSLFNDRKLVEYLKESSFDAVFLDPFDTCGLIVAKYFSLPSVVFTRGIFCHHLEEG
AQCPAPLSYVPNDLLGFSDAMTFKERVWNHIVHLEDHLFCQYLFRNALEIASEILQTPVT
AYDLYSHTSIWLLRTDFVLDYPKPVMPNMIFIGGINCHQGKPLPMEFEAYINASGEHGIV

VFSLGSMVSEIPEKKAMAIADALGKIPQTVLWRYTGTRPSNLANNTILVKWLPQNDLLGH
PMTRAFITHAGSHGVYESICNGVPMVMMPLFGDQMDNAKRMETKGAGVTLNVLEMTSEDL
ENALKAVINDKSYKENIMRLSSLHKDRPVEPLDLAVFWVEFVMRHKGAPHLRPAAHDLTW
YQYHSLDVIGFLLAVVLTVAFITFKCCAYGYRKCLGKKGRVKKAHKSKTH
>hsa:54576
MARTGWTSPIPLCVSLLLTCGFAEAGKLLVVPMDGSHWFTMQSVVEKLILRGHEVVVVMP
EVSWQLGKSLNCTVKTYSTSYTLEDLDREFMDFADAQWKAQVRSLFSLFLSSSNGFFNLF
FSHCRSLFNDRKLVEYLKESSFDAVFLDPFDACGLIVAKYFSLPSVVFARGIACHYLEEG
AQCPAPLSYVPRILLGFSDAMTFKERVRNHIMHLEEHLFCQYFSKNALEIASEILQTPVT
AYDLYSHTSIWLLRTDFVLDYPKPVMPNMIFIGGINCHQGKPLPMEFEAYINASGEHGIV
VFSLGSMVSEIPEKKAMAIADALGKIPQTVLWRYTGTRPSNLANNTILVKWLPQNDLLGH
PMTRAFITHAGSHGVYESICNGVPMVMMPLFGDQMDNAKRMETKGAGVTLNVLEMTSEDL
ENALKAVINDKSYKENIMRLSSLHKDRPVEPLDLAVFWVEFVMRHKGAPHLRPAAHDLTW
YQYHSLDVIGFLLAVVLTVAFITFKCCAYGYRKCLGKKGRVKKAHKSKTH
>hsa:54577
MARAGWTGLLPLYVCLLLTCGFAKAGKLLVVPMDGSHWFTMQSVVEKLILRGHEVVVVMP
EVSWQLGRSLNCTVKTYSTSYTLEDQDREFMVFADARWTAPLRSAFSLLTSSSNGIFDLF
FSNCRSLFNDRKLVEYLKESCFDAVFLDPFDACGLIVAKYFSLPSVVFARGIFCHYLEEG
AQCPAPLSYVPRLLLGFSDAMTFKERVWNHIMHLEEHLFCPYFFKNVLEIASEILQTPVT
AYDLYSHTSIWLLRTDFVLEYPKPVMPNMIFIGGINCHQGKPVPMEFEAYINASGEHGIV
VFSLGSMVSEIPEKKAMAIADALGKIPQTVLWRYTGTRPSNLANNTILVKWLPQNDLLGH
PMTRAFITHAGSHGVYESICNGVPMVMMPLFGDQMDNAKRMETKGAGVTLNVLEMTSEDL
ENALKAVINDKSYKENIMRLSSLHKDRPVEPLDLAVFWVEFVMRHKGAPHLRPAAHDLTW
YQYHSLDVIGFLLAVVLTVAFITFKCCAYGYRKCLGKKGRVKKAHKSKTH
>hsa:54578
MACLLRSFQRISAGVFFLALWGMVVGDKLLVVPQDGSHWLSMKDIVEVLSDRGHEIVVVV
PEVNLLLKESKYYTRKIYPVPYDQEELKNRYQSFGNNHFAERSFLTAPQTEYRNNMIVIG
LYFINCQSLLQDRDTLNFFKESKFDALFTDPALPCGVILAEYLGLPSVYLFRGFPCSLEH
TFSRSPDPVSYIPRCYTKFSDHMTFSQRVANFLVNLLEPYLFYCLFSKYEELASAVLKRD
VDIITLYQKVSVWLLRYDFVLEYPRPVMPNMVFIGGINCKKRKDLSQEFEAYINASGEHG
IVVFSLGSMVSEIPEKKAMAIADALGKIPQTVLWRYTGTRPSNLANNTILVKWLPQNDLL
GHPMTRAFITHAGSHGVYESICNGVPMVMMPLFGDQMDNAKRMETKGAGVTLNVLEMTSE
DLENALKAVINDKSYKENIMRLSSLHKDRPVEPLDLAVFWVEFVMRHKGAPHLRPAAHDL
TWYQYHSLDVIGFLLAVVLTVAFITFKCCAYGYRKCLGKKGRVKKAHKSKTH
>hsa:54579
MATGLQVPLPQLATGLLLLLSVQPWAESGKVLVVPTDGSHWLSMREALRDLHARGHQVVV
LTLEVNMYIKEENFFTLTTYAISWTQDEFDRLLLGHTQSFFETEHLLMKFSRRMAIMNNM
SLIIHRSCVELLHNEALIRHLHATSFDVVLTDPFHLCAAVLAKYLSIPAVFFLRNIPCDL
DFKGTQCPNPSSYIPRLLTTNSDHMTFLQRVKNMLYPLALSYLCHAVSAPYASLASELFQ
REVSVVDLVSHASVWLFRGDFVMDYPRPIMPNMVFIGGINCANGKPLSQEFEAYINASGE
HGIVVFSLGSMVSEIPEKKAMAIADALGKIPQTVLWRYTGTRPSNLANNTILVKWLPQND
LLGHPMTRAFITHAGSHGVYESICNGVPMVMMPLFGDQMDNAKRMETKGAGVTLNVLEMT
SEDLENALKAVINDKSYKENIMRLSSLHKDRPVEPLDLAVFWVEFVMRHKGAPHLRPAAH
DLTWYQYHSLDVIGFLLAVVLTVAFITFKCCAYGYRKCLGKKGRVKKAHKSKTH

>hsa:54600
MACTGWTSPLPLCVCLLLTCGFAEAGKLLVVPMDGSHWFTMRSVVEKLILRGHEVVVVMP
EVSWQLGRSLNCTVKTYSTSYTLEDLDREFKAFAHAQWKAQVRSIYSLLMGSYNDIFDLF
FSNCRSLFKDKKLVEYLKESSFDAVFLDPFDNCGLIVAKYFSLPSVVFARGILCHYLEEG
AQCPAPLSYVPRILLGFSDAMTFKERVRNHIMHLEEHLLCHRFFKNALEIASEILQTPVT
EYDLYSHTSIWLLRTDFVLDYPKPVMPNMIFIGGINCHQGKPLPMEFEAYINASGEHGIV
VFSLGSMVSEIPEKKAMAIADALGKIPQTVLWRYTGTRPSNLANNTILVKWLPQNDLLGH
PMTRAFITHAGSHGVYESICNGVPMVMMPLFGDQMDNAKRMETKGAGVTLNVLEMTSEDL
ENALKAVINDKSYKENIMRLSSLHKDRPVEPLDLAVFWVEFVMRHKGAPHLRPAAHDLTW
YQYHSLDVIGFLLAVVLTVAFITFKCCAYGYRKCLGKKGRVKKAHKSKTH
>hsa:54657
MARGLQVPLPRLATGLLLLLSVQPWAESGKVLVVPTDGSPWLSMREALRELHARGHQAVV
LTPEVNMHIKEEKFFTLTAYAVPWTQKEFDRVTLGYTQGFFETEHLLKRYSRSMAIMNNV
SLALHRCCVELLHNEALIRHLNATSFDVVLTDPVNLCGAVLAKYLSIPAVFFWRYIPCDL
DFKGTQCPNPSSYIPKLLTTNSDHMTFLQRVKNMLYPLALSYICHTFSAPYASLASELFQ
REVSVVDLVSYASVWLFRGDFVMDYPRPIMPNMVFIGGINCANGKPLSQEFEAYINASGE
HGIVVFSLGSMVSEIPEKKAMAIADALGKIPQTVLWRYTGTRPSNLANNTILVKWLPQND
LLGHPMTRAFITHAGSHGVYESICNGVPMVMMPLFGDQMDNAKRMETKGAGVTLNVLEMT
SEDLENALKAVINDKSYKENIMRLSSLHKDRPVEPLDLAVFWVEFVMRHKGAPHLRPAAH
DLTWYQYHSLDVIGFLLAVVLTVAFITFKCCAYGYRKCLGKKGRVKKAHKSKTH
>hsa:54658
MAVESQGGRPLVLGLLLCVLGPVVSHAGKILLIPVDGSHWLSMLGAIQQLQQRGHEIVVL
APDASLYIRDGAFYTLKTYPVPFQREDVKESFVSLGHNVFENDSFLQRVIKTYKKIKKDS
AMLLSGCSHLLHNKELMASLAESSFDVMLTDPFLPCSPIVAQYLSLPTVFFLHALPCSLE
FEATQCPNPFSYVPRPLSSHSDHMTFLQRVKNMLIAFSQNFLCDVVYSPYATLASEFLQR
EVTVQDLLSSASVWLFRSDFVKDYPRPIMPNMVFVGGINCLHQNPLSQEFEAYINASGEH
GIVVFSLGSMVSEIPEKKAMAIADALGKIPQTVLWRYTGTRPSNLANNTILVKWLPQNDL
LGHPMTRAFITHAGSHGVYESICNGVPMVMMPLFGDQMDNAKRMETKGAGVTLNVLEMTS
EDLENALKAVINDKSYKENIMRLSSLHKDRPVEPLDLAVFWVEFVMRHKGAPHLRPAAHD
LTWYQYHSLDVIGFLLAVVLTVAFITFKCCAYGYRKCLGKKGRVKKAHKSKTH
>hsa:54659
MATGLQVPLPWLATGLLLLLSVQPWAESGKVLVVPIDGSHWLSMREVLRELHARGHQAVV
LTPEVNMHIKEENFFTLTTYAISWTQDEFDRHVLGHTQLYFETEHFLKKFFRSMAMLNNM
SLVYHRSCVELLHNEALIRHLNATSFDVVLTDPVNLCAAVLAKYLSIPTVFFLRNIPCDL
DFKGTQCPNPSSYIPRLLTTNSDHMTFMQRVKNMLYPLALSYICHAFSAPYASLASELFQ
REVSVVDILSHASVWLFRGDFVMDYPRPIMPNMVFIGGINCANRKPLSQEFEAYINASGE
HGIVVFSLGSMVSEIPEKKAMAIADALGKIPQTVLWRYTGTRPSNLANNTILVKWLPQND
LLGHPMTRAFITHAGSHGVYESICNGVPMVMMPLFGDQMDNAKRMETKGAGVTLNVLEMT
SEDLENALKAVINDKSYKENIMRLSSLHKDRPVEPLDLAVFWVEFVMRHKGAPHLRPAAH
DLTWYQYHSLDVIGFLLAVVLTVAFITFKCCAYGYRKCLGKKGRVKKAHKSKTH
>hsa:54677
MENQLAKSTEERTFQYQDSLPSLPVPSLEESLKKYLESVTRTCYQIRGLDPDAKRGFLDL
TREGIQVKPFANQEEYKKTEEIVQKFQSGIGEKLHQKLLERAKGKRNWLEEWWLNVAYLD
VRIPSQLNVNFAGPAAHFEHYWPPKEGTQLERGSITLWHNLNYWQLLRKEKVPVHKVGNT

PLDMNQFRMLFSTCKVPGITRDSIMNYFRTESEGRSPNHIVVLCRGRAFVFDVIHEGCLV
TPPELLRQLTYIHKKCHSEPDGPGIAALTSEERTRWAKAREYLIGLDPENLALLEKIQSS
LLVYSMEDSSPHVTPEDYSEIIAAILIGDPTVRWGDKSYNLISFSNGVFGCNCDHAPFDA
MIMVNISYYVDEKIFQNEGRWKGSEKVRDIPLPEELIFIVDEKVLNDINQAKAQYLREAS
DLQIAAYAFTSFGKKLTKNKMLHPDTFIQLALQLAYYRLHGHPGCCYETAMTRHFYHGRT
ETMRSCTVEAVRWCQSMQDPSVNLRERQQKMLQAFAKHNKMMKDCSAGKGFDRHLLGLLL
IAKEEGLPVPELFTDPLFSKSGGGGNFVLSTSLVGYLRVQGVVVPMVHNGYGFFYHIRDD
RFVVACSAWKSCPETDAEKLVQLTFCAFHDMIQLMNSTHL
>hsa:5470
MGSGTSTQHHFAFQNAERAFKAAALIQRWYRRYVARLEMRRRCTWSIFQSIEYAGQQDQV
KLHDFFSYLMDHFIPSSHNDRDFLTRIFTEDRFAQDSEMKKCSDYESIEVPDSYTGPRLS
FPLLPDHATALVEAFRLKQQLHARYVLNLLYETKKHLVQLPNINRVSTCYSEEITVCGDL
HGQLDDLIFIFYKNGLPSPERSYVFNGDFVDRGKDSVEILMILFAFMLVYPKEFHLNRGN
HEDHMVNLRYGFTKEVMNKYKVHGKEILRTLQDVFCWLPLATLIDEKVLILHGGVSDITD
LELLDKIERSKIVSTMRCKTRQKSEKQMEEKRRANQKSSAQGPIPWFLPESRSLPSSPLR
LGSYKAQKTSRSSSIPCSGSLDGRELSRQVRSSVELELERCRQQAGLLVTGEKEEPSRSA
SEADSEAGELRKPTQEEWRQVVDILWSDPMAQEGCKANTIRGGGCYFGPDVTQQLLQKYN
MQFLIRSHECKPEGYEFCHNRKVLTIFSASNYYEVGSNRGAYVKLGPALTPHIVQYQANK
VTHTLTMRQRISRVEESALRALREKLFAHSSDLLSEFKKHDADKVGLITLSDWAAAVESV
LHLGLPWRMLRPQLVNSSADNMLEYKSWLKNLAKEQLSRENIQSSLLETLYRNRSNLETI
FRIIDSDHSGFISLDEFRQTWKLFSSHMNIDITDDCICDLARSIDFNKDGHIDINEFLEA
FRLVEKSCPEGDASECPQATNAKDSGCSSPGAH
>hsa:5475
MGCSSSSTKTRRSDTSLRAALIIQNWYRGYKARLKARQHYALTIFQSIEYADEQGQMQLS
TFFSFMLENYTHIHKEELELRNQSLESEQDMRDRWDYVDSIDVPDSYNGPRLQFPLTCTD
IDLLLEAFKEQQILHAHYVLEVLFETKKVLKQMPNFTHIQTSPSKEVTICGDLHGKLDDL
FLIFYKNGLPSERNPYVFNGDFVDRGKNSIEILMILCVSFLVYPNDLHLNRGNHEDFMMN
LRYGFTKEILHKYKLHGKRILQILEEFYAWLPIGTIVDNEILVIHGGISETTDLNLLHRV
ERNKMKSVLIPPTETNRDHDTDSKHNKVGVTFNAHGRIKTNGSPTEHLTEHEWEQIIDIL
WSDPRGKNGCFPNTCRGGGCYFGPDVTSKILNKYQLKMLIRSHECKPEGYEICHDGKVVT
IFSASNYYEEGSNRGAYIKLCSGTTPRFFQYQVTKATCFQPLRQRVDTMENSAIKILRER
VISRKSDLTRAFQLQDHRKSGKLSVSQWAFCMENILGLNLPWRSLSSNLVNIDQNGNVEY
MSSFQNIRIEKPVQEAHSTLVETLYRYRSDLEIIFNAIDTDHSGLISVEEFRAMWKLFSS
HYNVHIDDSQVNKLANIMDLNKDGSIDFNEFLKAFYVVHRYEDLMKPDVTNLG
>hsa:5476
MTSSPRAPPGEQGRGGAEMIRAAPPPLFLLLLLLLLLVSWASRGEAAPDQDEIQRLPGLA
KQPSFRQYSGYLKGSGSKHLHYWFVESQKDPENSPVVLWLNGGPGCSSLDGLLTEHGPFL
VQPDGVTLEYNPYSWNLIANVLYLESPAGVGFSYSDDKFYATNDTEVAQSNFEALQDFFR
LFPEYKNNKLFLTGESYAGIYIPTLAVLVMQDPSMNLQGLAVGNGLSSYEQNDNSLVYFA
YYHGLLGNRLWSSLQTHCCSQNKCNFYDNKDLECVTNLQEVARIVGNSGLNIYNLYAPCA
GGVPSHFRYEKDTVVVQDLGNIFTRLPLKRMWHQALLRSGDKVRMDPPCTNTTAASTYLN
NPYVRKALNIPEQLPQWDMCNFLVNLQYRRLYRSMNSQYLKLLSSQKYQILLYNGDVDMA
CNFMGDEWFVDSLNQKMEVQRRPWLVKYGDSGEQIAGFVKEFSHIAFLTIKGAGHMVPTD
KPLAAFTMFSRFLNKQPY

>hsa:5478
MVNPTVFFDIAVDGEPLGRVSFELFADKVPKTAENFRALSTGEKGFGYKGSCFHRIIPGF
MCQGGDFTRHNGTGGKSIYGEKFEDENFILKHTGPGILSMANAGPNTNGSQFFICTAKTE
WLDGKHVVFGKVKEGMNIVEAMERFGSRNGKTSKKITIADCGQLE
>hsa:5479
MLRLSERNMKVLLAAALIAGSVFFLLLPGPSAADEKKKGPKVTVKVYFDLRIGDEDVGRV
IFGLFGKTVPKTVDNFVALATGEKGFGYKNSKFHRVIKDFMIQGGDFTRGDGTGGKSIYG
ERFPDENFKLKHYGPGWVSMANAGKDTNGSQFFITTVKTAWLDGKHVVFGKVLEGMEVVR
KVESTKTDSRDKPLKDVIIADCGKIEVEKPFAIAKE
>hsa:5481
MSHPSPQAKPSNPSNPRVFFDVDIGGERVGRIVLELFADIVPKTAENFRALCTGEKGIGH
TTGKPLHFKGCPFHRIIKKFMIQGGDFSNQNGTGGESIYGEKFEDENFHYKHDREGLLSM
ANAGRNTNGSQFFITTVPTPHLDGKHVVFGQVIKGIGVARILENVEVKGEKPAKLCVIAE
CGELKEGDDGGIFPKDGSGDSHPDFPEDADIDLKDVDKILLITEDLKNIGNTFFKSQNWE
MAIKKYAEVLRYVDSSKAVIETADRAKLQPIALSCVLNIGACKLKMSNWQGAIDSCLEAL
ELDPSNTKALYRRAQGWQGLKEYDQALADLKKAQGIAPEDKAIQAELLKVKQKIKAQKDK
EKAVYAKMFA
>hsa:54878
MAAAMETEQLGVEIFETADCEENIESQDRPKLEPFYVERYSWSQLKKLLADTRKYHGYMM
AKAPHDFMFVKRNDPDGPHSDRIYYLAMSGENRENTLFYSEIPKTINRAAVLMLSWKPLL
DLFQATLDYGMYSREEELLRERKRIGTVGIASYDYHQGSGTFLFQAGSGIYHVKDGGPQG
FTQQPLRPNLVETSCPNIRMDPKLCPADPDWIAFIHSNDIWISNIVTREERRLTYVHNEL
ANMEEDARSAGVATFVLQEEFDRYSGYWWCPKAETTPSGGKILRILYEENDESEVEIIHV
TSPMLETRRADSFRYPKTGTANPKVTFKMSEIMIDAEGRIIDVIDKELIQPFEILFEGVE
YIARAGWTPEGKYAWSILLDRSQTRLQIVLISPELFIPVEDDVMERQRLIESVPDSVTPL
IIYEETTDIWINIHDIFHVFPQSHEEEIEFIFASECKTGFRHLYKITSILKESKYKRSSG
GLPAPSDFKCPIKEEIAITSGEWEVLGRHGSNIQVDEVRRLVYFEGTKDSPLEHHLYVVS
YVNPGEVTRLTDRGYSHSCCISQHCDFFISKYSNQKNPHCVSLYKLSSPEDDPTCKTKEF
WATILDSAGPLPDYTPPEIFSFESTTGFTLYGMLYKPHDLQPGKKYPTVLFIYGGPQVAI
AGAPVTLWIFYDTGYTERYMGHPDQNEQGYYLGSVAMQAEKFPSEPNRLLLLHGFLDENV
HFAHTSILLSFLVRAGKPYDLQIYPQERHSIRVPESGEHYELHLLHYLQENLGSRIAALK
VI
>hsa:5494
MGAFLDKPKMEKHNAQGQGNGLRYGLSSMQGWRVEMEDAHTAVIGLPSGLESWSFFAVYD
GHAGSQVAKYCCEHLLDHITNNQDFKGSAGAPSVENVKNGIRTGFLEIDEHMRVMSEKKH
GADRSGSTAVGVLISPQHTYFINCGDSRGLLCRNRKVHFFTQDHKPSNPLEKERIQNAGG
SVMIQRVNGSLAVSRALGDFDYKCVHGKGPTEQLVSPEPEVHDIERSEEDDQFIILACDG
IWDVMGNEELCDFVRSRLEVTDDLEKVCNEVVDTCLYKGSRDNMSVILICFPNAPKVSPE
AVKKEAELDKYLECRVEEIIKKQGEGVPDLVHVMRTLASENIPSLPPGGELASKRNVIEA
VYNRLNPYKNDDTDSTSTDDMW
>hsa:5495
MGAFLDKPKTEKHNAHGAGNGLRYGLSSMQGWRVEMEDAHTAVVGIPHGLEDWSFFAVYD
GHAGSRVANYCSTHLLEHITTNEDFRAAGKSGSALELSVENVKNGIRTGFLKIDEYMRNF
SDLRNGMDRSGSTAVGVMISPKHIYFINCGDSRAVLYRNGQVCFSTQDHKPCNPREKERI

QNAGGSVMIQRVNGSLAVSRALGDYDYKCVDGKGPTEQLVSPEPEVYEILRAEEDEFIIL
ACDGIWDVMSNEELCEYVKSRLEVSDDLENVCNWVVDTCLHKGSRDNMSIVLVCFSNAPK
VSDEAVKKDSELDKHLESRVEGKTNAF
>hsa:5496
MGAYLSQPNTVKCSGDGVGAPRLPLPYGFSAMQGWRVSMEDAHNCIPELDSETAMFSVYD
GHGGEEVALYCAKYLPDIIKDQKAYKEGKLQKALEDAFLAIDAKLTTEEVIKELAQIAGR
PTEDEDEKEKVADEDDVDNEEAALLHEEATMTIEELLTRYGQNCHKGPPHSKSGGGTGEE
PGSQGLNGEAGPEDSTRETPSQENGPTAKAYTGFSSNSERGTEAGQVGEPGIPTGEAGPS
CSSASDKLPRVAKSKFFEDSEDESDEAEEEEEDSEECSEEEDGYSSEEAENEEDEDDTEE
AEEDDEEEEEEMMVPGMEGKEEPGSDSGTTAVVALIRGKQLIVANAGDSRCVVSEAGKAL
DMSYDHKPEDEVELARIKNAGGKVTMDGRVNGGLNLSRAIGDHFYKRNKNLPPEEQMISA
LPDIKVLTLTDDHEFMVIACDGIWNVMSSQEVVDFIQSKISQRDENGELRLLSSIVEELL
DQCLAPDTSGDGTGCDNMTCIIICFKPRNTAELQPESGKRKLEEVLSTEGAEENGNSDKK
KKAKRD
>hsa:5499
MSDSEKLNLDSIIGRLLEGSRVLTPHCAPVQGSRPGKNVQLTENEIRGLCLKSREIFLSQ
PILLELEAPLKICGDIHGQYYDLLRLFEYGGFPPESNYLFLGDYVDRGKQSLETICLLLA
YKIKYPENFFLLRGNHECASINRIYGFYDECKRRYNIKLWKTFTDCFNCLPIAAIVDEKI
FCCHGGLSPDLQSMEQIRRIMRPTDVPDQGLLCDLLWSDPDKDVQGWGENDRGVSFTFGA
EVVAKFLHKHDLDLICRAHQVVEDGYEFFAKRQLVTLFSAPNYCGEFDNAGAMMSVDETL
MCSFQILKPADKNKGKYGQFSGLNPGGRPITPPRNSAKAKK
>hsa:55
MRAAPLLLARAASLSLGFLFLLFFWLDRSVLAKELKFVTLVFRHGDRSPIDTFPTDPIKE
SSWPQGFGQLTQLGMEQHYELGEYIRKRYRKFLNESYKHEQVYIRSTDVDRTLMSAMTNL
AALFPPEGVSIWNPILLWQPIPVHTVPLSEDQLLYLPFRNCPRFQELESETLKSEEFQKR
LHPYKDFIATLGKLSGLHGQDLFGIWSKVYDPLYCESVHNFTLPSWATEDTMTKLRELSE
LSLLSLYGIHKQKEKSRLQGGVLVNEILNHMKRATQIPSYKKLIMYSAHDTTVSGLQMAL
DVYNGLLPPYASCHLTELYFEKGEYFVEMYYRNETQHEPYPLMLPGCSPSCPLERFAELV
GPVIPQDWSTECMTTNSHQGTEDSTD
>hsa:5500
MADGELNVDSLITRLLEVRGCRPGKIVQMTEAEVRGLCIKSREIFLSQPILLELEAPLKI
CGDIHGQYTDLLRLFEYGGFPPEANYLFLGDYVDRGKQSLETICLLLAYKIKYPENFFLL
RGNHECASINRIYGFYDECKRRFNIKLWKTFTDCFNCLPIAAIVDEKIFCCHGGLSPDLQ
SMEQIRRIMRPTDVPDTGLLCDLLWSDPDKDVQGWGENDRGVSFTFGADVVSKFLNRHDL
DLICRAHQVVEDGYEFFAKRQLVTLFSAPNYCGEFDNAGGMMSVDETLMCSFQILKPSEK
KAKYQYGGLNSGRPVTPPRTANPPKKR
>hsa:5501
MADLDKLNIDSIIQRLLEVRGSKPGKNVQLQENEIRGLCLKSREIFLSQPILLELEAPLK
ICGDIHGQYYDLLRLFEYGGFPPESNYLFLGDYVDRGKQSLETICLLLAYKIKYPENFFL
LRGNHECASINRIYGFYDECKRRYNIKLWKTFTDCFNCLPIAAIVDEKIFCCHGGLSPDL
QSMEQIRRIMRPTDVPDQGLLCDLLWSDPDKDVLGWGENDRGVSFTFGAEVVAKFLHKHD
LDLICRAHQVVEDGYEFFAKRQLVTLFSAPNYCGEFDNAGAMMSVDETLMCSFQILKPAE
KKKPNATRPVTPPRVASGLNPSIQKASNYRNNTVLYE
>hsa:5515

MDEKVFTKELDQWIEQLNECKQLSESQVKSLCEKAKEILTKESNVQEVRCPVTVCGDVHG
QFHDLMELFRIGGKSPDTNYLFMGDYVDRGYYSVETVTLLVALKVRYRERITILRGNHES
RQITQVYGFYDECLRKYGNANVWKYFTDLFDYLPLTALVDGQIFCLHGGLSPSIDTLDHI
RALDRLQEVPHEGPMCDLLWSDPDDRGGWGISPRGAGYTFGQDISETFNHANGLTLVSRA
HQLVMEGYNWCHDRNVVTIFSAPNYCYRCGNQAAIMELDDTLKYSFLQFDPAPRRGEPHV
TRRTPDYFL
>hsa:5516
MDDKAFTKELDQWVEQLNECKQLNENQVRTLCEKAKEILTKESNVQEVRCPVTVCGDVHG
QFHDLMELFRIGGKSPDTNYLFMGDYVDRGYYSVETVTLLVALKVRYPERITILRGNHES
RQITQVYGFYDECLRKYGNANVWKYFTDLFDYLPLTALVDGQIFCLHGGLSPSIDTLDHI
RALDRLQEVPHEGPMCDLLWSDPDDRGGWGISPRGAGYTFGQDISETFNHANGLTLVSRA
HQLVMEGYNWCHDRNVVTIFSAPNYCYRCGNQAAIMELDDTLKYSFLQFDPAPRRGEPHV
TRRTPDYFL
>hsa:5523
MDIEEQKADIYEMGKIAKVCGCPLYWKAPMFRAAGGEKTGFVTAQSFIAMWRKLLNNHHD
DASKFICLLAKPNCSSLEQEDFIPLLQDVVDTHPGLTFLKDAPEFHSRYITTVIQRIFYT
VNRSWSGKITSTEIRKSNFLQTLALLEEEEDINQITDYFSYEHFYVIYCKFWELDTDHDL
YISQADLSRYNDQASSSRIIERIFSGAVTRGKTIQKEGRMSYADFVWFLISEEDKRNPTS
IEYWFRCMDVDGDGVLSMYELEYFYEEQCERMEAMGIEPLPFHDLLCQMLDLVKPAVDGK
ITLRDLKRCRMAHIFYDTFFNLEKYLDHEQRDPFAVQKDVENDGPEPSDWDRFAAEEYET
LVAEESAQAQFQEGFEDYETDEPASPSEFGNKSNKILSASLPEKCGKLQSVDEE
>hsa:5530
MSEPKAIDPKLSTTDRVVKAVPFPPSHRLTAKEVFDNDGKPRVDILKAHLMKEGRLEESV
ALRIITEGASILRQEKNLLDIDAPVTVCGDIHGQFFDLMKLFEVGGSPANTRYLFLGDYV
DRGYFSIECVLYLWALKILYPKTLFLLRGNHECRHLTEYFTFKQECKIKYSERVYDACMD
AFDCLPLAALMNQQFLCVHGGLSPEINTLDDIRKLDRFKEPPAYGPMCDILWSDPLEDFG
NEKTQEHFTHNTVRGCSYFYSYPAVCEFLQHNNLLSILRAHEAQDAGYRMYRKSQTTGFP
SLITIFSAPNYLDVYNNKAAVLKYENNVMNIRQFNCSPHPYWLPNFMDVFTWSLPFVGEK
VTEMLVNVLNICSDDELGSEEDGFDGATAAARKEVIRNKIRAIGKMARVFSVLREESESV
LTLKGLTPTGMLPSGVLSGGKQTLQSATVEAIEADEAIKGFSPQHKITSFEEAKGLDRIN
ERMPPRRDAMPSDANLNSINKALTSETNGTDSNGSNSSNIQ
>hsa:5531
MAEISDLDRQIEQLRRCELIKESEVKALCAKAREILVEESNVQRVDSPVTVCGDIHGQFY
DLKELFRVGGDVPETNYLFMGDFVDRGFYSVETFLLLLALKVRYPDRITLIRGNHESRQI
TQVYGFYDECLRKYGSVTVWRYCTEIFDYLSLSAIIDGKIFCVHGGLSPSIQTLDQIRTI
DRKQEVPHDGPMCDLLWSDPEDTTGWGVSPRGAGYLFGSDVVAQFNAANDIDMICRAHQL
VMEGYKWHFNETVLTVWSAPNYCYRCGNVAAILELDEHLQKDFIIFEAAPQETRGIPSKK
PVADYFL
>hsa:55312
MRHLPYFCRGQVVRGFGRGSKQLGIPTANFPEQVVDNLPADISTGIYYGWASVGSGDVHK
MVVSIGWNPYYKNTKKSMETHIMHTFKEDFYGEILNVAIVGYLRPEKNFDSLESLISAIQ
GDIEEAKKRLELPEHLKIKEDNFFQVSKSKIMNGH
>hsa:5532
MAAPEPARAAPPPPPPPPPPPGADRVVKAVPFPPTHRLTSEEVFDLDGIPRVDVLKNHLV

KEGRVDEEIALRIINEGAAILRREKTMIEVEAPITVCGDIHGQFFDLMKLFEVGGSPANT
RYLFLGDYVDRGYFSIECVLYLWVLKILYPSTLFLLRGNHECRHLTEYFTFKQECKIKYS
ERVYEACMEAFDSLPLAALLNQQFLCVHGGLSPEIHTLDDIRRLDRFKEPPAFGPMCDLL
WSDPSEDFGNEKSQEHFSHNTVRGCSYFYNYPAVCEFLQNNNLLSIIRAHEAQDAGYRMY
RKSQTTGFPSLITIFSAPNYLDVYNNKAAVLKYENNVMNIRQFNCSPHPYWLPNFMDVFT
WSLPFVGEKVTEMLVNVLSICSDDELMTEGEDQFDVGSAAARKEIIRNKIRAIGKMARVF
SVLREESESVLTLKGLTPTGMLPSGVLAGGRQTLQSATVEAIEAEKAIRGFSPPHRICSF
EEAKGLDRINERMPPRKDAVQQDGFNSLNTAHATENHGTGNHTAQ
>hsa:5533
MSGRRFHLSTTDRVIKAVPFPPTQRLTFKEVFENGKPKVDVLKNHLVKEGRLEEEVALKI
INDGAAILRQEKTMIEVDAPITVCGDIHGQFFDLMKLFEVGGSPSNTRYLFLGDYVDRGY
FSIECVLYLWSLKINHPKTLFLLRGNHECRHLTDYFTFKQECRIKYSEQVYDACMETFDC
LPLAALLNQQFLCVHGGMSPEITSLDDIRKLDRFTEPPAFGPVCDLLWSDPSEDYGNEKT
LEHYTHNTVRGCSYFYSYPAVCEFLQNNNLLSIIRAHEAQDAGYRMYRKSQATGFPSLIT
IFSAPNYLDVYNNKAAVLKYENNVMNIRQFNCSPHPYWLPNFMDVFTWSLPFVGEKVTEM
LVNVLNICSDDELISDDEAEDHYIPSYQKGSTTVRKEIIRNKIRAIGKMARVFSILRQES
ESVLTLKGLTPTGTLPLGVLSGGKQTIETATVEAVEAREAIRGFSLQHKIRSFEEARGLD
RINERMPPRKDSIHAGGPMKSVTSAHSHAAHRSDQGKKAHS
>hsa:55359
MGMTRMLLECSLSDKLCVIQEKQYEVIIVPTLLVTIFLILLGVILWLFIREQRTQQQRSG
PQGIAPVPPPRDLSWEAGHGGNVALPLKETSVENFLGATTPALAKLQVPREQLSEVLEQI
CSGSCGPIFRANMNTGDPSKPKSVILKALKEPAGLHEVQDFLGRIQFHQYLGKHKNLVQL
EGCCTEKLPLYMVLEDVAQGDLLSFLWTCRRDVMTMDGLLYDLTEKQVYHIGKQVLLALE
FLQEKHLFHGDVAARNILMQSDLTAKLCGLGLAYEVYTRGAISSTQTIPLKWLAPERLLL
RPASIRADVWSFGILLYEMVTLGAPPYPEVPPTSILEHLQRRKIMKRPSSCTHTMYSIMK
SCWRWREADRPSPRELRLRLEAAIKTADDEAVLQVPELVVPELYAAVAGIRVESLFYNYS
ML
>hsa:5536
MAMAEGERTECAEPPRDEPPADGALKRAEELKTQANDYFKAKDYENAIKFYSQAIELNPS
NAIYYGNRSLAYLRTECYGYALGDATRAIELDKKYIKGYYRRAASNMALGKFRAALRDYE
TVVKVKPHDKDAKMKYQECNKIVKQKAFERAIAGDEHKRSVVDSLDIESMTIEDEYSGPK
LEDGKVTISFMKELMQWYKDQKKLHRKCAYQTEKITVCGDTHGQFYDLLNIFELNGLPSE
TNPYIFNGDFVDRGSFSVEVILTLFGFKLLYPDHFHLLRGNHETDNMNQIYGFEGEVKAK
YTAQMYELFSEVFEWLPLAQCINGKVLIMHGGLFSEDGVTLDDIRKIERNRQPPDSGPMC
DLLWSDPQPQNGRSISKRGVSCQFGPDVTKAFLEENNLDYIIRSHEVKAEGYEVAHGGRC
VTVFSAPNYCDQMGNKASYIHLQGSDLRPQFHQFTAVPHPNVKPMAYANTLLQLGMM
>hsa:5537
MAPLDLDKYVEIARLCKYLPENDLKVSPICGLAPSGCGAPAGRPFLSPGPPPVFHFLRFL
KERLCDYVCDLLLEESNVQPVSTPVTVCGDIHGQFYDLCELFRTGGQVPDTNYIFMGDFV
DRGYYSLETFTYLLALKAKWPDRITLLRGNHESRQITQVYGFYDECQTKYGNANAWRYCT
KVFDMLTVAALIDEQILCVHGGLSPDIKTLDQIRTIERNQEIPHKGAFCDLVWSDPEDVD
TWAISPRGAGWLFGAKVTNEFVHINNLKLICRAHQLVHEGYKFMFDEKLVTVWSAPNYCY
RCGNIASIMVFKDVNTREPKLFRAVPDSERVIPPRTTTPYFL
>hsa:5538

MASPGCLWLLAVALLPWTCASRALQHLDPPAPLPLVIWHGMGDSCCNPLSMGAIKKMVEK
KIPGIYVLSLEIGKTLMEDVENSFFLNVNSQVTTVCQALAKDPKLQQGYNAMGFSQGGQF
LRAVAQRCPSPPMINLISVGGQHQGVFGLPRCPGESSHICDFIRKTLNAGAYSKVVQERL
VQAEYWHDPIKEDVYRNHSIFLADINQERGINESYKKNLMALKKFVMVKFLNDSIVDPVD
SEWFGFYRSGQAKETIPLQETSLYTQDRLGLKEMDNAGQLVFLATEGDHLQLSEEWFYAH
IIPFLG
>hsa:5547
MGRRALLLLLLSFLAPWATIALRPALRALGSLHLPTNPTSLPAVAKNYSVLYFQQKVDHF
GFNTVKTFNQRYLVADKYWKKNGGSILFYTGNEGDIIWFCNNTGFMWDVAEELKAMLVFA
EHRYYGESLPFGDNSFKDSRHLNFLTSEQALADFAELIKHLKRTIPGAENQPVIAIGGSY
GGMLAAWFRMKYPHMVVGALAASAPIWQFEDLVPCGVFMKIVTTDFRKSGPHCSESIHRS
WDAINRLSNTGSGLQWLTGALHLCSPLTSQDIQHLKDWISETWVNLAMVDYPYASNFLQP
LPAWPIKVVCQYLKNPNVSDSLLLQNIFQALNVYYNYSGQVKCLNISETATSSLGTLGWS
YQACTEVVMPFCTNGVDDMFEPHSWNLKELSDDCFQQWGVRPRPSWITTMYGGKNISSHT
NIVFSNGELDPWSGGGVTKDITDTLVAVTISEGAHHLDLRTKNALDPMSVLLARSLEVRH
MKNWIRDFYDSAGKQH
>hsa:5550
MLSLQYPDVYRDETAVQDYHGHKICDPYAWLEDPDSEQTKAFVEAQNKITVPFLEQCPIR
GLYKERMTELYDYPKYSCHFKKGKRYFYFYNTGLQNQRVLYVQDSLEGEARVFLDPNILS
DDGTVALRGYAFSEDGEYFAYGLSASGSDWVTIKFMKVDGAKELPDVLERVKFSCMAWTH
DGKGMFYNSYPQQDGKSDGTETSTNLHQKLYYHVLGTDQSEDILCAEFPDEPKWMGGAEL
SDDGRYVLLSIREGCDPVNRLWYCDLQQESSGIAGILKWVKLIDNFEGEYDYVTNEGTVF
TFKTNRQSPNYRVINIDFRDPEESKWKVLVPEHEKDVLEWIACVRSNFLVLCYLHDVKNI
LQLHDLTTGALLKTFPLDVGSIVGYSGQKKDTEIFYQFTSFLSPGIIYHCDLTKEELEPR
VFREVTVKGIDASDYQTVQIFYPSKDGTKIPMFIVHKKGIKLDGSHPAFLYGYGGFNISI
TPNYSVSRLIFVRHMGGILAVANIRGGGEYGETWHKGGILANKQNCFDDFQCAAEYLIKE
GYTSPKRLTINGGSNGGLLVAACANQRPDLFGCVIAQVGVMDMLKFHKYTIGHAWTTDYG
CSDSKQHFEWLVKYSPLHNVKLPEADDIQYPSMLLLTADHDDRVVPLHSLKFIATLQYIV
GRSRKQSNPLLIHVDTKAGHGAGKPTAKVIEEVSDMFAFIARCLNVDWIP
>hsa:55512
MVLYTTPFPNSCLSALHCVSWALIFPCYWLVDRLAASFIPTTYEKRQRADDPCCLQLLCT
ALFTPIYLALLVASLPFAFLGFLFWSPLQSARRPYIYSRLEDKGLAGGAALLSEWKGTGP
GKSFCFATANVCLLPDSLARVNNLFNTQARAKEIGQRIRNGAARPQIKIYIDSPTNTSIS
AASFSSLVSPQGGDGVARAVPGSIKRTASVEYKGDGGRHPGDEAANGPASGDPVDSSSPE
DACIVRIGGEEGGRPPEADDPVPGGQARNGAGGGPRGQTPNHNQQDGDSGSLGSPSASRE
SLVKGRAGPDTSASGEPGANSKLLYKASVVKKAAARRRRHPDEAFDHEVSAFFPANLDFL
CLQEVFDKRAATKLKEQLHGYFEYILYDVGVYGCQGCCSFKCLNSGLLFASRYPIMDVAY
HCYPNKCNDDALASKGALFLKVQVGSTPQDQRIVGYIACTHLHAPQEDSAIRCGQLDLLQ
DWLADFRKSTSSSSAANPEELVAFDVVCGDFNFDNCSSDDKLEQQHSLFTHYRDPCRLGP
GEEKPWAIGTLLDTNGLYDEDVCTPDNLQKVLESEEGRREYLAFPTSKSSGQKGRKELLK
GNGRRIDYMLHAEEGLCPDWKAEVEEFSFITQLSGLTDHLPVAMRLMVSSGEEEA
>hsa:5562
MRRLSSWRKMATAEKQKHDGRVKIGHYILGDTLGVGTFGKVKVGKHELTGHKVAVKILNR
QKIRSLDVVGKIRREIQNLKLFRHPHIIKLYQVISTPSDIFMVMEYVSGGELFDYICKNG

RLDEKESRRLFQQILSGVDYCHRHMVVHRDLKPENVLLDAHMNAKIADFGLSNMMSDGEF
LRTSCGSPNYAAPEVISGRLYAGPEVDIWSSGVILYALLCGTLPFDDDHVPTLFKKICDG
IFYTPQYLNPSVISLLKHMLQVDPMKRATIKDIREHEWFKQDLPKYLFPEDPSYSSTMID
DEALKEVCEKFECSEEEVLSCLYNRNHQDPLAVAYHLIIDNRRIMNEAKDFYLATSPPDS
FLDDHHLTRPHPERVPFLVAETPRARHTLDELNPQKSKHQGVRKAKWHLGIRSQSRPNDI
MAEVCRAIKQLDYEWKVVNPYYLRVRRKNPVTSTYSKMSLQLYQVDSRTYLLDFRSIDDE
ITEAKSGTATPQRSGSVSNYRSCQRSDSDAEAQGKSSEVSLTSSVTSLDSSPVDLTPRPG
SHTIEFFEMCANLIKILAQ
>hsa:55775
MSQEGDYGRWTISSSDESEEEKPKPDKPSTSSLLCARQGAANEPRYTCSEAQKAAHKRKI
SPVKFSNTDSVLPPKRQKSGSQEDLGWCLSSSDDELQPEMPQKQAEKVVIKKEKDISAPN
DGTAQRTENHGAPACHRLKEEEDEYETSGEGQDIWDMLDKGNPFQFYLTRVSGVKPKYNS
GALHIKDILSPLFGTLVSSAQFNYCFDVDWLVKQYPPEFRKKPILLVHGDKREAKAHLHA
QAKPYENISLCQAKLDIAFGTHHTKMMLLLYEEGLRVVIHTSNLIHADWHQKTQGIWLSP
LYPRIADGTHKSGESPTHFKADLISYLMAYNAPSLKEWIDVIHKHDLSETNVYLIGSTPG
RFQGSQKDNWGHFRLKKLLKDHASSMPNAESWPVVGQFSSVGSLGADESKWLCSEFKESM
LTLGKESKTPGKSSVPLYLIYPSVENVRTSLEGYPAGGSLPYSIQTAEKQNWLHSYFHKW
SAETSGRSNAMPHIKTYMRPSPDFSKIAWFLVTSANLSKAAWGALEKNGTQLMIRSYELG
VLFLPSAFGLDSFKVKQKFFAGSQEPMATFPVPYDLPPELYGSKDRPWIWNIPYVKAPDT
HGNMWVPS
>hsa:558
MAWRCPRMGRVPLAWCLALCGWACMAPRGTQAEESPFVGNPGNITGARGLTGTLRCQLQV
QGEPPEVHWLRDGQILELADSTQTQVPLGEDEQDDWIVVSQLRITSLQLSDTGQYQCLVF
LGHQTFVSQPGYVGLEGLPYFLEEPEDRTVAANTPFNLSCQAQGPPEPVDLLWLQDAVPL
ATAPGHGPQRSLHVPGLNKTSSFSCEAHNAKGVTTSRTATITVLPQQPRNLHLVSRQPTE
LEVAWTPGLSGIYPLTHCTLQAVLSDDGMGIQAGEPDPPEEPLTSQASVPPHQLRLGSLH
PHTPYHIRVACTSSQGPSSWTHWLPVETPEGVPLGPPENISATRNGSQAFVHWQEPRAPL
QGTLLGYRLAYQGQDTPEVLMDIGLRQEVTLELQGDGSVSNLTVCVAAYTAAGDGPWSLP
VPLEAWRPVKEPSTPAFSWPWWYVLLGAVVAAACVLILALFLVHRRKKETRYGEVFEPTV
ERGELVVRYRVRKSYSRRTTEATLNSLGISEELKEKLRDVMVDRHKVALGKTLGEGEFGA
VMEGQLNQDDSILKVAVKTMKIAICTRSELEDFLSEAVCMKEFDHPNVMRLIGVCFQGSE
RESFPAPVVILPFMKHGDLHSFLLYSRLGDQPVYLPTQMLVKFMADIASGMEYLSTKRFI
HRDLAARNCMLNENMSVCVADFGLSKKIYNGDYYRQGRIAKMPVKWIAIESLADRVYTSK
SDVWSFGVTMWEIATRGQTPYPGVENSEIYDYLRQGNRLKQPADCLDGLYALMSRCWELN
PQDRPSFTELREDLENTLKALPPAQEPDEILYVNMDEGGGYPEPPGAAGGADPPTQPDPK
DSCSCLTAAEVHPAGRYVLCPSTTPSPAQPADRGSPAAPGQEDGA
>hsa:55811
MLVFGDETHSHFLVIGQAVDDVRLAQNMAQMNDVILSPNCWQLCDRSMIEIESVPDQRAV
KVNFLKPPPNFNFDEFFTKCTTFMHYYPSGEHKNLLRLACTLKPDPELEMSLQKYVMESI
LKQIDNKQLQGYLSELRPVTIVFVNLMFEDQDKAEEIGPAIQDAYMHITSVLKIFQGQIN
KVFMFDKGCSFLCVFGFPGEKVPDELTHALECAMDIFDFCSQVHKIQTVSIGVASGIVFC
GIVGHTVRHEYTVIGQKVNLAARMMMYYPGIVTCDSVTYNGSNLPAYFFKELPKKVMKGV
ADSGPLYQYWGRTEKVMFGMACLICNRKEDYPLLGRNKEINYFMYTMKKFLISNSSQVLM
YEGLPGYGKSQILMKIEYLAQGKNHRIIAISLNKISFHQTFYTIQMFMANVLGLDTCKHY

KERQTNLRNKVMTLLDEKFYCLLNDIFHVQFPISREISRMSTLKKQKQLEILFMKILKLI
VKEERIIFIIDEAQFVDSTSWRFMEKLIRTLPIFIIMSLCPFVNIPCAAARAVIKNRNTT
YIVIGAVQPNDISNKICLDLNVSCISKELDSYLGEGSCGIPFYCEELLKNLEHHEVLVFQ
QTESEEKTNRTWNNLFKYSIKLTEKLNMVTLHSDKESEEVCHLTSGVRLKNLSPPTSLKE
ISLIQLDSMRLSHQMLVRCAAIIGLTFTTELLFEILPCWNMKMMIKTLATLVESNIFYCF
RNGKELQKALKQNDPSFEVHYRSLSLKPSEGMDHGEEEQLRELENEVIECHRIRFCNPMM
QKTAYELWLKDQRKAMHLKCARFLEEDAHRCDHCRGRDFIPYHHFTVNIRLNALDMDAIK
KMAMSHGFKTEEKLILSNSEIPETSAFFPENRSPEEIREKILNFFDHVLTKMKTSDEDII
PLESCQCEEILEIVILPLAHHFLALGENDKALYYFLEIASAYLIFCDNYMAYMYLNEGQK
LLKTLKKDKSWSQTFESATFYSLKGEVCFNMGQIVLAKKMLRKALKLLNRIFPYNLISLF
LHIHVEKNRHFHYVNRQAQESPPPGKKRLAQLYRQTVCLSLLWRIYSYSYLFHCKYYAHL
AVMMQMNTALETQNCFQIIKAYLDYSLYHHLAGYKGVWFKYEVMAMEHIFNLPLKGEGIE
IVAYVAETLVFNKLIMGHLDLAIELGSRALQMWALLQNPNRHYQSLCRLSRCLLLNSRYP
QLIQVLGRLWELSVTQEHIFSKAFFYFVCLDILLYSGFVYRTFEECLEFIHQYENNRILK
FHSGLLLGLYSSVAIWYARLQEWDNFYKFSNRAKNLLPRRTMTLTYYDGISRYMEGQVLH
LQKQIKEQSENAQASGEELLKNLENLVAQNTTGPVFCPRLYHLMAYVCILMGDGQKCGLF
LNTALRLSETQGNILEKCWLNMNKESWYSTSELKEDQWLQTILSLPSWEKIVAGRVNIQD
LQKNKFLMRANTVDNHF
>hsa:55902
MGLPEERVRSGSGSRGQEEAGAGGRARSWSPPPEVSRSAHVPSLQRYRELHRRSVEEPRE
FWGDIAKEFYWKTPCPGPFLRYNFDVTKGKIFIEWMKGATTNICYNVLDRNVHEKKLGDK
VAFYWEGNEPGETTQITYHQLLVQVCQFSNVLRKQGIQKGDRVAIYMPMIPELVVAMLAC
ARIGALHSIVFAGFSSESLCERILDSSCSLLITTDAFYRGEKLVNLKELADEALQKCQEK
GFPVRCCIVVKHLGRAELGMGDSTSQSPPIKRSCPDVQGKLKEKSKRVQPQISWNQGIDL
WWHELMQEAGDECEPEWCDAEDPLFILYTSGSTGKPKGVVHTVGGYMLYVATTFKYVFDF
HAEDVFWCTADIGWITGHSYVVTYGPLANGATSVLFEGIPTYPDVNRLWSIVDKYKVTKFY
TAPTAIRLLMKFGDEPVTKHSRASLQVLGTVGEPINPEAWLWYHRVVGAQRCPIVDTFWQ
TETGGHMLTPLPGATPMKPGSATFPFFGVAPAILNESGEELEGEAEGYLVFKQPWPGIMR
TVYGNHERFETTYFKKFPGYYVTGDGCQRDQDGYYWITGRIDDMLNVSGHLLSTAEVESA
LVEHEAVAEAAVVGHPHPVKGECLYCFVTLCDGHTFSPKLTEELKKQIREKIGPIATPDY
IQNAPGLPKTRSGKIMRRVLRKIAQNDHDLGDMSTVADPSVISHLFSHRCLTIQ
>hsa:5594
MAAAAAAGAGPEMVRGQVFDVGPRYTNLSYIGEGAYGMVCSAYDNVNKVRVAIKKISPFE
HQTYCQRTLREIKILLRFRHENIIGINDIIRAPTIEQMKDVYIVQDLMETDLYKLLKTQH
LSNDHICYFLYQILRGLKYIHSANVLHRDLKPSNLLLNTTCDLKICDFGLARVADPDHDH
TGFLTEYVATRWYRAPEIMLNSKGYTKSIDIWSVGCILAEMLSNRPIFPGKHYLDQLNHI
LGILGSPSQEDLNCIINLKARNYLLSLPHKNKVPWNRLFPNADSKALDLLDKMLTFNPHK
RIEVEQALAHPYLEQYYDPSDEPIAEAPFKFDMELDDLPKEKLKELIFEETARFQPGYRS
>hsa:5595
MAAAAAQGGGGGEPRRTEGVGPGVPGEVEMVKGQPFDVGPRYTQLQYIGEGAYGMVSSAY
DHVRKTRVAIKKISPFEHQTYCQRTLREIQILLRFRHENVIGIRDILRASTLEAMRDVYI
VQDLMETDLYKLLKSQQLSNDHICYFLYQILRGLKYIHSANVLHRDLKPSNLLINTTCDL
KICDFGLARIADPEHDHTGFLTEYVATRWYRAPEIMLNSKGYTKSIDIWSVGCILAEMLS
NRPIFPGKHYLDQLNHILGILGSPSQEDLNCIINMKARNYLQSLPSKTKVAWAKLFPKSD

SKALDLLDRMLTFNPNKRITVEEALAHPYLEQYYDPTDEVGQSPAAVGLGAGEQGGT
>hsa:5596
MAEKGDCIASVYGYDLGGRFVDFQPLGFGVNGLVLSAVDSRACRKVAVKKIALSDARSMK
HALREIKIIRRLDHDNIVKVYEVLGPKGTDLQGELFKFSVAYIVQEYMETDLARLLEQGT
LAEEHAKLFMYQLLRGLKYIHSANVLHRDLKPANIFISTEDLVLKIGDFGLARIVDQHYS
HKGYLSEGLVTKWYRSPRLLLSPNNYTKAIDMWAAGCILAEMLTGRMLFAGAHELEQMQL
ILETIPVIREEDKDELLRVMPSFVSSTWEVKRPLRKLLPEVNSEAIDFLEKILTFNPMDR
LTAEMGLQHPYMSPYSCPEDEPTSQHPFRIEDEIDDIVLMAANQSQLSNWDTCSSRYPVS
LSSDLEWRPDRCQDASEVQRDPRAGSAPLAEDVQVDPRKDSHSSSERFLEQSHSSMERAF
EADYGRSCDYKVGSPSYLDKLLWRDNKPHHYSEPKLILDLSHWKQAAGAPPTATGLADTG
AREDEPASLFLEIAQWVKSTQGGPEHASPPADDPERRLSASPPGRPAPVDGGASPQFDLD
VFISRALKLCTKPEDLPDNKLGDLNGACIPEHPGDLVQTEAFSKERW
>hsa:5597
MAEKFESLMNIHGFDLGSRYMDLKPLGCGGNGLVFSAVDNDCDKRVAIKKIVLTDPQSVK
HALREIKIIRRLDHDNIVKVFEILGPSGSQLTDDVGSLTELNSVYIVQEYMETDLANVLE
QGPLLEEHARLFMYQLLRGLKYIHSANVLHRDLKPANLFINTEDLVLKIGDFGLARIMDP
HYSHKGHLSEGLVTKWYRSPRLLLSPNNYTKAIDMWAAGCIFAEMLTGKTLFAGAHELEQ
MQLILESIPVVHEEDRQELLSVIPVYIRNDMTEPHKPLTQLLPGISREALDFLEQILTFS
PMDRLTAEEALSHPYMSIYSFPMDEPISSHPFHIEDEVDDILLMDETHSHIYNWERYHDC
QFSEHDWPVHNNFDIDEVQLDPRALSDVTDEEEVQVDPRKYLDGDREKYLEDPAFDTNYS
TEPCWQYSDHHENKYCDLECSHTCNYKTRSSSYLDNLVWRESEVNHYYEPKLIIDLSNWK
EQSKEKSDKKGKSKCERNGLVKAQIALEEASQQLAGKEREKNQGFDFDSFIAGTIQLSSQ
HEPTDVVDKLNDLNSSVSQLELKSLISKSVSQEKQEKGMANLAQLEALYQSSWDSQFVSG
GEDCFFINQFCEVRKDEQVEKENTYTSYLDKFFSRKEDTEMLETEPVEDGKLGERGHEEG
FLNNSGEFLFNKQLESIGIPQFHSPVGSPLKSIQATLTPSAMKSSPQIPHQTYSSILKHL
N
>hsa:5598
MAEPLKEEDGEDGSAEPPGPVKAEPAHTAASVAAKNLALLKARSFDVTFDVGDEYEIIET
IGNGAYGVVSSARRRLTGQQVAIKKIPNAFDVVTNAKRTLRELKILKHFKHDNIIAIKDI
LRPTVPYGEFKSVYVVLDLMESDLHQIIHSSQPLTLEHVRYFLYQLLRGLKYMHSAQVIH
RDLKPSNLLVNENCELKIGDFGMARGLCTSPAEHQYFMTEYVATRWYRAPELMLSLHEYT
QAIDLWSVGCIFGEMLARRQLFPGKNYVHQLQLIMMVLGTPSPAVIQAVGAERVRAYIQS
LPPRQPVPWETVYPGADRQALSLLGRMLRFEPSARISAAAALRHPFLAKYHDPDDEPDCA
PPFDFAFDREALTRERIKEAIVAEIEDFHARREGIRQQIRFQPSLQPVASEPGCPDVEMP
SPWAPSGDCAMESPPPAPPPCPGPAPDTIDLTLQPPPPVSEPAPPKKDGAISDNTKAALK
AALLKSLRSRLRDGPSAPLEAPEPRKPVTAQERQREREEKRRRRQERAKEREKRRQERER
KERGAGASGGPSTDPLAGLVLSDNDRSLLERWTRMARPAAPALTSVPAPAPAPTPTPTPV
QPTSPPPGPVAQPTGPQPQSAGSTSGPVPQPACPPPGPAPHPTGPPGPIPVPAPPQIATS
TSLLAAQSLVPPPGLPGSSTPGVLPYFPPGLPPPDAGGAPQSSMSESPDVNLVTQQLSKS
QVEDPLPPVFSGTPKGSGAGYGVGFDLEEFLNQSFDMGVADGPQDGQADSASLSASLLAD
WLEGHGMNPADIESLQREIQMDSPMLLADLPDLQDP
>hsa:5599
MSRSKRDNNFYSVEIGDSTFTVLKRYQNLKPIGSGAQGIVCAAYDAILERNVAIKKLSRP
FQNQTHAKRAYRELVLMKCVNHKNIIGLLNVFTPQKSLEEFQDVYIVMELMDANLCQVIQ

MELDHERMSYLLYQMLCGIKHLHSAGIIHRDLKPSNIVVKSDCTLKILDFGLARTAGTSF
MMTPYVVTRYYRAPEVILGMGYKENVDLWSVGCIMGEMVCHKILFPGRDYIDQWNKVIEQ
LGTPCPEFMKKLQPTVRTYVENRPKYAGYSFEKLFPDVLFPADSEHNKLKASQARDLLSK
MLVIDASKRISVDEALQHPYINVWYDPSEAEAPPPKIPDKQLDEREHTIEEWKELIYKEV
MDLEERTKNGVIRGQPSPLAQVQQ
>hsa:5600
MSGPRAGFYRQELNKTVWEVPQRLQGLRPVGSGAYGSVCSAYDARLRQKVAVKKLSRPFQ
SLIHARRTYRELRLLKHLKHENVIGLLDVFTPATSIEDFSEVYLVTTLMGADLNNIVKCQ
ALSDEHVQFLVYQLLRGLKYIHSAGIIHRDLKPSNVAVNEDCELRILDFGLARQADEEMT
GYVATRWYRAPEIMLNWMHYNQTVDIWSVGCIMAELLQGKALFPGSDYIDQLKRIMEVVG
TPSPEVLAKISSEHARTYIQSLPPMPQKDLSSIFRGANPLAIDLLGRMLVLDSDQRVSAA
EALAHAYFSQYHDPEDEPEAEPYDESVEAKERTLEEWKELTYQEVLSFKPPEPPKPPGSL
EIEQ
>hsa:5601
MSDSKCDSQFYSVQVADSTFTVLKRYQQLKPIGSGAQGIVCAAFDTVLGINVAVKKLSRP
FQNQTHAKRAYRELVLLKCVNHKNIISLLNVFTPQKTLEEFQDVYLVMELMDANLCQVIH
MELDHERMSYLLYQMLCGIKHLHSAGIIHRDLKPSNIVVKSDCTLKILDFGLARTACTNF
MMTPYVVTRYYRAPEVILGMGYKENVDIWSVGCIMGELVKGCVIFQGTDRILPRDLGPAM
LS
>hsa:5602
MSLHFLYYCSEPTLDVKIAFCQGFDKQVDVSYIAKHYNMSKSKVDNQFYSVEVGDSTFTV
LKRYQNLKPIGSGAQGIVCAAYDAVLDRNVAIKKLSRPFQNQTHAKRAYRELVLMKCVNH
KNIISLLNVFTPQKTLEEFQDVYLVMELMDANLCQVIQMELDHERMSYLLYQMLCGIKHL
HSAGIIHRDLKPSNIVVKSDCTLKILDFGLARTAGTSFMMTPYVVTRYYRAPEVILGMGY
KENVDIWSVGCIMGEMVRHKILFPGRDYIDQWNKVIEQLGTPCPEFMKKLQPTVRNYVEN
RPKYAGLTFPKLFPDSLFPADSEHNKLKASQARDLLSKMLVIDPAKRISVDDALQHPYIN
VWYDPAEVEAPPPQIYDKQLDEREHTIEEWKELIYKEVMNSEEKTKNGVVKGQPSPSAQV
QQ
>hsa:5603
MSLIRKKGFYKQDVNKTAWELPKTYVSPTHVGSGAYGSVCSAIDKRSGEKVAIKKLSRPF
QSEIFAKRAYRELLLLKHMQHENVIGLLDVFTPASSLRNFYDFYLVMPFMQTDLQKIMGM
EFSEEKIQYLVYQMLKGLKYIHSAGVVHRDLKPGNLAVNEDCELKILDFGLARHADAEMT
GYVVTRWYRAPEVILSWMHYNQTVDIWSVGCIMAEMLTGKTLFKGKDYLDQLTQILKVTG
VPGTEFVQKLNDKAAKSYIQSLPQTPRKDFTQLFPRASPQAADLLEKMLELDVDKRLTAA
QALTHPFFEPFRDPEEETEAQQPFDDSLEHEKLTVDEWKQHIYKEIVNFSPIARKDSRRR
SGMKL
>hsa:5604
MPKKKPTPIQLNPAPDGSAVNGTSSAETNLEALQKKLEELELDEQQRKRLEAFLTQKQKV
GELKDDDFEKISELGAGNGGVVFKVSHKPSGLVMARKLIHLEIKPAIRNQIIRELQVLHE
CNSPYIVGFYGAFYSDGEISICMEHMDGGSLDQVLKKAGRIPEQILGKVSIAVIKGLTYL
REKHKIMHRDVKPSNILVNSRGEIKLCDFGVSGQLIDSMANSFVGTRSYMSPERLQGTHY
SVQSDIWSMGLSLVEMAVGRYPIPPPDAKELELMFGCQVEGDAAETPPRPRTPGRPLSSY
GMDSRPPMAIFELLDYIVNEPPPKLPSGVFSLEFQDFVNKCLIKNPAERADLKQLMVHAF
IKRSDAEEVDFAGWLCSTIGLNQPSTPTHAAGV

>hsa:5605
MLARRKPVLPALTINPTIAEGPSPTSEGASEANLVDLQKKLEELELDEQQKKRLEAFLTQ
KAKVGELKDDDFERISELGAGNGGVVTKVQHRPSGLIMARKLIHLEIKPAIRNQIIRELQ
VLHECNSPYIVGFYGAFYSDGEISICMEHMDGGSLDQVLKEAKRIPEEILGKVSIAVLRG
LAYLREKHQIMHRDVKPSNILVNSRGEIKLCDFGVSGQLIDSMANSFVGTRSYMAPERLQ
GTHYSVQSDIWSMGLSLVELAVGRYPIPPPDAKELEAIFGRPVVDGEEGEPHSISPRPRP
PGRPVSGHGMDSRPAMAIFELLDYIVNEPPPKLPNGVFTPDFQEFVNKCLIKNPAERADL
KMLTNHTFIKRSEVEEVDFAGWLCKTLRLNQPGTPTRTAV
>hsa:5606
MSKPPAPNPTPPRNLDSRTFITIGDRNFEVEADDLVTISELGRGAYGVVEKVRHAQSGTI
MAVKRIRATVNSQEQKRLLMDLDINMRTVDCFYTVTFYGALFREGDVWICMELMDTSLDK
FYRKVLDKNMTIPEDILGEIAVSIVRALEHLHSKLSVIHRDVKPSNVLINKEGHVKMCDF
GISGYLVDSVAKTMDAGCKPYMAPERINPELNQKGYNVKSDVWSLGITMIEMAILRFPYE
SWGTPFQQLKQVVEEPSPQLPADRFSPEFVDFTAQCLRKNPAERMSYLELMEHPFFTLHK
TKKTDIAAFVKEILGEDS
>hsa:5607
MMEGHFPQSDVIGQVLPEATTTAFEYEDEDGDRITVRSDEEMKAMLSYYYSTVMEQQVNG
QLIEPLQIFPRACKPPGERNIHGLKVNTRAGPSQHSSPAVSDSLPSNSLKKSSAELKKIL
ANGQMNEQDIRYRDTLGHGNGGTVYKAYHVPSGKILAVKVILLDITLELQKQIMSELEIL
YKCDSSYIIGFYGAFFVENRISICTEFMDGGSLDVYRKMPEHVLGRIAVAVVKGLTYLWS
LKILHRDVKPSNMLVNTRGQVKLCDFGVSTQLVNSIAKTYVGTNAYMAPERISGEQYGIH
SDVWSLGISFMELALGRFPYPQIQKNQGSLMPLQLLQCIVDEDSPVLPVGEFSEPFVHFI
TQCMRKQPKERPAPEELMGHPFIVQFNDGNAAVVSMWVCRALEERRSQQGPP
>hsa:5608
MSQSKGKKRNPGLKIPKEAFEQPQTSSTPPRDLDSKACISIGNQNFEVKADDLEPIMELG
RGAYGVVEKMRHVPSGQIMAVKRIRATVNSQEQKRLLMDLDISMRTVDCPFTVTFYGALF
REGDVWICMELMDTSLDKFYKQVIDKGQTIPEDILGKIAVSIVKALEHLHSKLSVIHRDV
KPSNVLINALGQVKMCDFGISGYLVDSVAKTIDAGCKPYMAPERINPELNQKGYSVKSDI
WSLGITMIELAILRFPYDSWGTPFQQLKQVVEEPSPQLPADKFSAEFVDFTSQCLKKNSK
ERPTYPELMQHPFFTLHESKGTDVASFVKLILGD
>hsa:5624
MWQLTSLLLFVATWGISGTPAPLDSVFSSSERAHQVLRIRKRANSFLEELRHSSLERECI
EEICDFEEAKEIFQNVDDTLAFWSKHVDGDQCLVLPLEHPCASLCCGHGTCIDGIGSFSC
DCRSGWEGRFCQREVSFLNCSLDNGGCTHYCLEEVGWRRCSCAPGYKLGDDLLQCHPAVK
FPCGRPWKRMEKKRSHLKRDTEDQEDQVDPRLIDGKMTRRGDSPWQVVLLDSKKKLACGA
VLIHPSWVLTAAHCMDESKKLLVRLGEYDLRRWEKWELDLDIKEVFVHPNYSKSTTDNDI
ALLHLAQPATLSQTIVPICLPDSGLAERELNQAGQETLVTGWGYHSSREKEAKRNRTFVL
NFIKIPVVPHNECSEVMSNMVSENMLCAGILGDRQDACEGDSGGPMVASFHGTWFLVGLV
SWGEGCGLLHNYGVYTKVSRYLDWIHGHIRDKEAPQKSWAP
>hsa:5625
MKMTFYGHFVAGEDQESIQPLLRHYRAFGVSAILDYGVEEDLSPEEAEHKEMESCTSAAE
RDGSGTNKRDKQYQAHRAFGDRRNGVISARTYFYANEAKCDSHMETFLRCIEASGRVSDD
GFIAIKLTALGRPQFLLQFSEVLAKWRCFFHQMAVEQGQAGLAAMDTKLEVAVLQESVAK
LGIASRAEIEDWFTAETLGVSGTMDLLDWSSLIDSRTKLSKHLVVPNAQTGQLEPLLSRF

TEEEELQMTRMLQRMDVLAKKATEMGVRLMVDAEQTYFQPAISRLTLEMQRKFNVEKPLI
FNTYQCYLKDAYDNVTLDVELARREGWCFGAKLVRGAYLAQERARAAEIGYEDPINPTYE
ATNAMYHRCLDYVLEELKHNAKAKVMVASHNEDTVRFALRRMEELGLHPADHQVYFGQLL
GMCDQISFPLGQAGYPVYKYVPYGPVMEVLPYLSRRALENSSLMKGTHRERQLLWLELLR
RLRTGNLFHRPA
>hsa:5645
MNLLLILTFVAAAVAAPFDDDDKIVGGYICEENSVPYQVSLNSGYHFCGGSLISEQWVVS
AGHCYKSRIQVRLGEHNIEVLEGNEQFINAAKIIRHPKYNSRTLDNDILLIKLSSPAVIN
SRVSAISLPTAPPAAGTESLISGWGNTLSSGADYPDELQCLDAPVLSQAECEASYPGKIT
NNMFCVGFLEGGKDSCQGDSGGPVVSNGELQGIVSWGYGCAQKNRPGVYTKVYNYVDWIK
DTIAANS
>hsa:5646
MHMRETSGFTLKKGRSAPLVFHPPDALIAVPFDDDDKIVGGYTCEENSLPYQVSLNSGSH
FCGGSLISEQWVVSAAHCYKTRIQVRLGEHNIKVLEGNEQFINAAKIIRHPKYNRDTLDN
DIMLIKLSSPAVINARVSTISLPTAPPAAGTECLISGWGNTLSFGADYPDELKCLDAPVL
TQAECKASYPGKITNSMFCVGFLEGGKDSCQRDSGGPVVCNGQLQGVVSWGHGCAWKNRP
GVYTKVYNYVDWIKDTIAANS
>hsa:5650
MNEYTVHLGSDTLGDRRAQRIKASKSFRHPGYSTQTHVNDLMLVKLNSQARLSSMVKKVR
LPSRCEPPGTTCTVSGWGTTTSPDVTFPSDLMCVDVKLISPQDCTKVYKDLLENSMLCAG
IPDSKKNACNGDSGGPLVCRGTLQGLVSWGTFPCGQPNDPGVYTQVCKFTKWINDTMKKH
R
>hsa:5651
MGSKRGISSRHHSLSSYEIMFAALFAILVVLCAGLIAVSCLTIKESQRGAALGQSHEARA
TFKITSGVTYNPNLQDKLSVDFKVLAFDLQQMIDEIFLSSNLKNEYKNSRVLQFENGSII
VVFDLFFAQWVSDENVKEELIQGLEANKSSQLVTFHIDLNSVDILDKLTTTSHLATPGNV
SIECLPGSSPCTDALTCIKADLFCDGEVNCPDGSDEDNKMCATVCDGRFLLTGSSGSFQA
THYPKPSETSVVCQWIIRVNQGLSIKLSFDDFNTYYTDILDIYEGVGSSKILRASIWETN
PGTIRIFSNQVTATFLIESDESDYVGFNATYTAFNSSELNNYEKINCNFEDGFCFWVQDL
NDDNEWERIQGSTFSPFTGPNFDHTFGNASGFYISTPTGPGGRQERVGLLSLPLDPTLEP
ACLSFWYHMYGENVHKLSINISNDQNMEKTVFQKEGNYGDNWNYGQVTLNETVKFKVAFN
AFKNKILSDIALDDISLTYGICNGSLYPEPTLVPTPPPELPTDCGGPFELWEPNTTFSST
NFPNSYPNLAFCVWILNAQKGKNIQLHFQEFDLENINDVVEIRDGEEADSLLLAVYTGPG
PVKDVFSTTNRMTVLLITNDVLARGGFKANFTTGYHLGIPEPCKADHFQCKNGECVPLVN
LCDGHLHCEDGSDEADCVRFFNGTTNNNGLVRFRIQSIWHTACAENWTTQISNDVCQLLG
LGSGNSSKPIFSTDGGPFVKLNTAPDGHLILTPSQQCLQDSLIRLQCNHKSCGKKLAAQD
ITPKIVGGSNAKEGAWPWVVGLYYGGRLLCGASLVSSDWLVSAAHCVYGRNLEPSKWTAI
LGLHMKSNLTSPQTVPRLIDEIVINPHYNRRRKDNDIAMMHLEFKVNYTDYIQPICLPEE
NQVFPPGRNCSIAGWGTVVYQGTTANILQEADVPLLSNERCQQQMPEYNITENMICAGYE
EGGIDSCQGDSGGPLMCQENNRWFLAGVTSFGYKCALPNRPGVYARVSRFTEWIQSFLH
>hsa:5657
MAHRPPSPALASVLLALLLSGAARAAEIVGGHEAQPHSRPYMASLQMRGNPGSHFCGGTL
IHPSFVLTAAHCLRDIPQRLVNVVLGAHNVRTQEPTQQHFSVAQVFLNNYDAENKLNDVL
LIQLSSPANLSASVATVQLPQQDQPVPHGTQCLAMGWGRVGAHDPPAQVLQELNVTVVTF

FCRPHNICTFVPRRKAGICFGDSGGPLICDGIIQGIDSFVIWGCATRLFPDFFTRVALYV
DWIRSTLRRVEAKGRP
>hsa:56922
MAAASAVSVLLVAAERNRWHRLPSLLLPPRTWVWRQRTMKYTTATGRNITKVLIANRGEI
ACRVMRTAKKLGVQTVAVYSEADRNSMHVDMADEAYSIGPAPSQQSYLSMEKIIQVAKTS
AAQAIHPGCGFLSENMEFAELCKQEGIIFIGPPPSAIRDMGIKSTSKSIMAAAGVPVVEG
YHGEDQSDQCLKEHARRIGYPVMIKAVRGGGGKGMRIVRSEQEFQEQLESARREAKKSFN
DDAMLIEKFVDTPRHVEVQVFGDHHGNAVYLFERDCSVQRRHQKIIEEAPAPGIKSEVRK
KLGEAAVRAAKAVNYVGAGTVEFIMDSKHNFCFMEMNTRLQVEHPVTEMITGTDLVEWQL
RIAAGEKIPLSQEEITLQGHAFEARIYAEDPSNNFMPVAGPLVHLSTPRADPSTRIETGV
RQGDEVSVHYDPMIAKLVVWAADRQAALTKLRYSLRQYNIVGLHTNIDFLLNLSGHPEFE
AGNVHTDFIPQHHKQLLLSRKAAAKESLCQAALGLILKEKAMTDTFTLQAHDQFSPFSSS
SGRRLNISYTRNMTLKDGKNNVAIAVTYNHDGSYSMQIEDKTFQVLGNLYSEGDCTYLKC
SVNGVASKAKLIILENTIYLFSKEGSIEIDIPVPKYLSSVSSQETQGGPLAPMTGTIEKV
FVKAGDKVKAGDSLMVMIAMKMEHTIKSPKDGTVKKVFYREGAQANRHTPLVEFEEEESD
KRESE
>hsa:57016
MATFVELSTKAKMPIVGLGTWKSPLGKVKEAVKVAIDAGYRHIDCAYVYQNEHEVGEAIQ
EKIQEKAVKREDLFIVSKLWPTFFERPLVRKAFEKTLKDLKLSYLDVYLIHWPQGFKSGD
DLFPKDDKGNAIGGKATFLDAWEAMEELVDEGLVKALGVSNFSHFQIEKLLNKPGLKYKP
VTNQVECHPYLTQEKLIQYCHSKGITVTAYSPLGSPDRPWAKPEDPSLLEDPKIKEIAAK
HKKTAAQVLIRFHIQRNVIVIPKSVTPARIVENIQVFDFKLSDEEMATILSFNRNWRACN
VLQSSHLEDYPFNAEY
>hsa:57176
MCIPPPNVTGSLHIGHALTVAIQDALVRWHRMRGDQVLWVPGSDHAGIATQAVVEKQLWK
ERGVRRHELSREAFLREVWQWKEAKGGEICEQLRALGASLDWDRECFTMDVGSSVAVTEA
FVRLYKAGLLYRNHQLVNWSCALRSAISDIEVENRPLPGHTQLRLPGCPTPVSFGLLFSV
AFPVDGEPDAEVVVGTTRPETLPGDVAVAVHPDDSRYTHLHGRQLRHPLMGQPLPLITDY
AVQPHVGTGAVKVTPAHSPADAEMGARHGLSPLNVIAEDGTMTSLCGDWLQGLHRFVARE
KIMSVLSEWGLFRGLQNHPMVLPICSRSGDVIEYLLKNQWFVRCQEMGARAAKAVESGAL
ELSPSFHQKNWQHWFSHIGDWCVSRQLWWGHQIPAYLVVEDHAQGEEDCWVVGRSEAEAR
EVAAELTGRPGAELTLERDPDVLDTWFSSALFPFSALGWPQETPDLARFYPLSLLETGSD
LLLFWVGRMVMLGTQLTGQLPFSKVLLHPMVRDRQGRKMSKSLGNVLDPRDIISGVEMQV
LQEKLRSGNLDPAELAIVAAAQKKDFPHGIPECGTDALRFTLCSHGVQAGDLHLSVSEVQ
SCRHFCNKIWNALRFILNALGEKFVPQPAEELSPSSPMDAWILSRLALAAQECERGFLTR
ELSLVTHALHHFWLHNLCDVYLEAVKPVLWHSPRPLGPPQVLFSCADLGLRLLAPLMPFL
AEELWQRLPPRPGCPPAPSISVAPYPSACSLEHWRQPELERRFSRVQEVVQVLRALRATY
QLTKARPRVLLQSSEPGDQGLFEAFLEPLGTLGYCGAVGLLPPGAAAPSGWAQAPLSDTA
QVYMELQGLVDPQIQLPLLAARRYKLQKQLDSLTARTPSEGEAGTQRQQKLSSLQLELSK
LDKAASHLRQLMDEPPAPGSPEL
>hsa:5740
MAWAALLGLLAALLLLLLLSRRRTRRPGEPPLDLGSIPWLGYALDFGKDAASFLTRMKEK
HGDIFTILVGGRYVTVLLDPHSYDAVVWEPRTRLDFHAYAIFLMERIFDVQLPHYSPSDE
KARMKLTLLHRELQALTEAMYTNLHAVLLGDATEAGSGWHEMGLLDFSYSFLLRAGYLTL

YGIEALPRTHESQAQDRVHSADVFHTFRQLDRLLPKLARGSLSVGDKDHMCSVKSRLWKL
LSPARLARRAHRSKWLESYLLHLEEMGVSEEMQARALVLQLWATQGNMGPAAFWLLLFLL
KNPEALAAVRGELESILWQAEQPVSQTTTLPQKVLDSTPVLDSVLSESLRLTAAPFITRE
VVVDLAMPMADGREFNLRRGDRLLLFPFLSPQRDPEIYTDPEVFKYNRFLNPDGSEKKDF
YKDGKRLKNYNMPWGAGHNHCLGRSYAVNSIKQFVFLVLVHLDLELINADVEIPEFDLSR
YGFGLMQPEHDVPVRYRIRP

>hsa:5742

MSRSLLLWFLLFLLLLPPLPVLLADPGAPTPVNPCCYYPCQHQGICVRFGLDRYQCDCTR
TGYSGPNCTIPGLWTWLRNSLRPSPSFTHFLLTHGRWFWEFVNATFIREMLMRLVLTVRS
NLIPSPPTYNSAHDYISWESFSNVSYYTRILPSVPKDCPTPMGTKGKKQLPDAQLLARRF
LLRRKFIPDPQGTNLMFAFFAQHFTHQFFKTSGKMGPGFTKALGHGVDLGHIYGDNLERQ
YQLRLFKDGKLKYQVLDGEMYPPSVEEAPVLMHYPRGIPPQSQMAVGQEVFGLLPGLMLY
ATLWLREHNRVCDLLKAEHPTWGDEQLFQTTRLILIGETIKIVIEEYVQQLSGYFLQLKF
DPELLFGVQFQYRNRIAMEFNHLYHWHPLMPDSFKVGSQEYSYEQFLFNTSMLVDYGVEA
LVDAFSRQIAGRIGGGRNMDHHILHVAVDVIRESREMRLQPFNEYRKRFGMKPYTSFQEL
VGEKEMAAELEELYGDIDALEFYPGLLLEKCHPNSIFGESMIEIGAPFSLKGLLGNPICS
PEYWKPSTFGGEVGFNIVKTATLKKLVCLNTKTCPYVSFRVPDASQDDGPAVERPSTEL

>hsa:5743

MLARALLLCAVLALSHTANPCCSHPCQNRGVCMSVGFDQYKCDCTRTGFYGENCSTPEFL
TRIKLFLKPTPNTVHYILTHFKGFWNVVNNIPFLRNAIMSYVLTSRSHLIDSPPTYNADY
GYKSWEAFSNLSYYTRALPPVPDDCPTPLGVKGKKQLPDSNEIVEKLLLRRKFIPDPQGS
NMMFAFFAQHFTHQFFKTDHKRGPAFTNGLGHGVDLNHIYGETLARQRKLRLFKDGKMKY
QIIDGEMYPPTVKDTQAEMIYPPQVPEHLRFAVGQEVFGLVPGLMMYATIWLREHNRVCD
VLKQEHPEWGDEQLFQTSRLILIGETIKIVIEDYVQHLSGYHFKLKFDPELLFNKQFQYQ
NRIAAEFNTLYHWHPLLPDTFQIHDQKYNYQQFIYNNSILLEHGITQFVESFTRQIAGRV
AGGRNVPPAVQKVSQASIDQSRQMKYQSFNEYRKRFMLKPYESFEELTGEKEMSAELEAL
YGDIDAVELYPALLVEKPRPDAIFGETMVEVGAPFSLKGLMGNVICSPAYWKPSTFGGEV
GFQIINTASIQSLICNNVKGCPFTSFSVPDPELIKTVTINASSSRSGLDDINPTVLLKER
STEL

>hsa:5747

MAAAYLDPNLNHTPNSSTKTHLGTGMERSPGAMERVLKVFHYFESNSEPTTWASIIRHGD
ATDVRGIIQKIVDSHKVKHVACYGFRLSHLRSEEVHWLHVDMGVSSVREKYELAHPPEEW
KYELRIRYLPKGFLNQFTEDKPTLNFFYQQVKSDYMLEIADQVDQEIALKLGCLEIRRSY
WEMRGNALEKKSNYEVLEKDVGLKRFFPKSLLDSVKAKTLRKLIQQTFRQFANLNREESI
LKFFEILSPVYRFDKECFKCALGSSWIISVELAIGPEEGISYLTDKGCNPTHLADFTQVQ
TIQYSNSEDKDRKGMLQLKIAGAPEPLTVTAPSLTIAENMADLIDGYCRLVNGTSQSFII
RPQKEGERALPSIPKLANSEKQGMRTHAVSVSETDDYAEIIDEEDTYTMPSTRDYEIQRE
RIELGRCIGEGQFGDVHQGIYMSPENPALAVAIKTCKNCTSDSVREKFLQEALTMRQFDH
PHIVKLIGVITENPVWIIMELCTLGELRSFLQVRKYSLDLASLILYAYQLSTALAYLESK
RFVHRDIAARNVLVSSNDCVKLGDFGLSRYMEDSTYYKASKGKLPIKWMAPESINFRRFT
SASDVWMFGVCMWEILMHGVKPFQGVKNNDVIGRIENGERLPMPPNCPPTLYSLMTKCWA
YDPSRRPRFTELKAQLSTILEEEKAQQEERMRMESRRQATVSWDSGGSDEAPPKPSRPGY
PSPRSSEGFYPSPQHMVQTNHYQVSGYPGSHGITAMAGSIYPGQASLLDQTDSWNHRPQE
IAMWQPNVEDSTVLDLRGIGQVLPTHLMEERLIRQQQEMEEDQRWLEKEERFLKPDVRLS

RGSIDREDGSLQGPIGNQHIYQPVGKPGKEEKNWAERNPAAPPKKPPRPGAPGHLGSLAS
LSSPADSYNEGVKPWRLQPQEISPPPTANLDRSNDKVYENVTGLVKAVIEMSSKIQPAPP
EEYVPMVKEVGLALRTLLATVDETIPLLPASTHREIEMAQKLLNSDLGELINKMKLAQQY
VMTSLQQEYKKQMLTAAHALAVDAKNLLDVIDQARLKMLGQTRPH

>hsa:5754

MGSFLSGEKRPSAPTVGSAMEKKEFPTPPGRVGPGTQTAIVFIKQPSSQDALQGRRALLR
CEVEAPGPVHVYWLLDGAPVQDTERRFAQGSSLSFAAVDRLQDSGTFQCVARDDVTGEEA
RSANASFNIKWIEAGPVVLKHPASEAEIQPQTQVTLRCHIDGHPRPTYQWFRDGTPLSDG
QSNHTVSSKERNLTLRPAGPEHSGLYSCCAHSAFGQACSSQNFTLSIADESFARVVLAPQ
DVVVARYEEAMFHCQFSAQPPPSLQWLFEDETPITNRSRPPHLRRATVFANGSLLLTQVR
PRNAGIYRCIGQGQRGPPIILEATLHLAEIEDMPLFEPRVFTAGSEERVTCLPPKGLPEP
SVWWEHAGVRLPTHGRVYQKGHELVLANIAESDAGVYTCHAANLAGQRRQDVNITVATVP
SWLKKPQDSQLEEGKPGYLDCLTQATPKPTVVWYRNQMLISEDSRFEVFKNGTLRINSVE
VYDGTWYRCMSSTPAGSIEAQARVQVLEKLKFTPPPQPQQCMEFDKEATVPCSATGREKP
TIKWERADGSSLPEWVTDNAGTLHFARVTRDDAGNYTCIASNGPQGQIRAHVQLTVAVFI
TFKVEPERTTVYQGHTALLQCEAQGDPKPLIQWKGKDRILDPTKLGPRMHIFQNGSLVIH
DVAPEDSGRYTCIAGNSCNIKHTEAPLYVVDKPVPEESEGPGSPPPYKMIQTIGLSVGAA
VAYIIAVLGLMFYCKKRCKAKRLQKQPEGEEPEMECLNGGPLQNGQPSAEIQEEVALTSL
GSGPAATNKRHSTSDKMHFPRSSLQPITTLGKSEFGEVFLAKAQGLEEGVAETLVLVKSL
QSKDEQQQLDFRRELEMFGKLNHANVVRLLGLCREAEPHYMVLEYVDLGDLKQFLRISKS
KDEKLKSQPLSTKQKVALCTQVALGMEHLSNNRFVHKDLAARNCLVSAQRQVKVSALGLS
KDVYNSEYYHFRQAWVPLRWMSPEAILEGDFSTKSDVWAFGVLMWEVFTHGEMPHGGQAD
DEVLADLQAGKARLPQPEGCPSKLYRLMQRCWALSPKDRPSFSEIASALGDSTVDSKP

>hsa:57665

MAVATAAAVLAALGGALWLAARRFVGPRVQRLRRGGDPGLMHGKTVLITGANSGLGRATA
AELLRLGARVIMGCRDRARAEEAAGQLRRELRQAAECGPEPGVSGVGELIVRELDLASLR
SVRAFCQEMLQEEPRLDVLINNAGIFQCPYMKTEDGFEMQFGVNHLGHFLLTNLLLGLLK
SSAPSRIVVVSSKLYKYGDINFDDLNSEQSYNKSFCYSRSKLANILFTRELARRLEGTNV
TVNVLHPGIVRTNLGRHIHIPLLVKPLFNLVSWAFFKTPVEGAQTSIYLASSPEVEGVSG
RYFGDCKEEELLPKAMDESVARKLWDISEVMVGLLK

>hsa:58190

MVAAPWATQEQEEGRGIQPGDRGDQKSAASQKPRSRGILHSLFCCVCRDDGEALPAHSGA
PLLVEENGAIPKTPVQYLLPEAKAQDSDKICVVIDLDETLVHSSFKPVNNADFIIPVEID
GVVHQVYVLKRPHVDEFLQRMGELFECVLFTASLAKYADPVADLLDKWGAFRARLFRESC
VFHRGNYVKDLSRLGRDLRRVLILDNSPASYVFHPDNAVPVASWFDNMSDTELHDLLPFF
EQLSRVDDVYSVLRQPRPGS

>hsa:5831

MSVGFIGAGQLAFALAKGFTAAGVLAAHKIMASSPDMDLATVSALRKMGVKLTPHNKETV
QHSDVLFLAVKPHIIPFILDEIGADIEDRHIVVSCAAGVTISSIEKKLSAFRPAPRVIRC
MTNTPVVVREGATVYATGTHAQVEDGRLMEQLLSSVGFCTEVEEDLIDAVTGLSGSGPAY
AFTALDALADGGVKMGLPRRLAVRLGAQALLGAAKMLLHSEQHPGQLKDNVSSPGGATIH
ALHVLESGGFRSLLINAVEASCIRTRELQSMADQEQVSPAAIKKTILDKVKLDSPAGTAL
SPSGHTKLLPRSLAPAGKD

>hsa:5834

MAKPLTDSEKRKQISVRGLAGLGDVAEVRKSFNRHLHFTLVKDRNVATPRDYFFALAHTV
RDHLVGRWIRTQQHYYERDPKRIYYLSLEFYMGRTLQNTMVNLGLQNACDEAIYQLGLDL
EELEEIEEDAGLGNGGLGRLAACFLDSMATLGLAAYGYGIRYEFGIFNQKIVNGWQVEEA
DDWLRYGNPWEKARPEYMLPVHFYGRVEHTPDGVKWLDTQVVLAMPYDTPVPGYKNNTVN
TMRLWSAKAPNDFKLQDFNVGDYIEAVLDRNLAENISRVLYPNDNFFEGKELRLKQEYFV
VAATLQDIIRRFKSSKFGCRDPVRTCFETFPDKVAIQLNDTHPALSIPELMRILVDVEKV
DWDKAWEITKKTCAYTNHTVLPEALERWPVSMFEKLLPRHLEIIYAINQRHLDHVAALFP
GDVDRLRRMSVIEEGDCKRINMAHLCVIGSHAVNGVARIHSEIVKQSVFKDFYELEPEKF
QNKTNGITPRRWLLLCNPGLADTIVEKIGEEFLTDLSQLKKLLPLVSDEVFIRDVAKVKQ
ENKLKFSAFLEKEYKVKINPSSMFDVHVKRIHEYKRQLLNCLHVVTLYNRIKRDPAKAFV
PRTVMIGGKAAPGYHMAKLIIKLVTSIGDVVNHDPVVGDRLKVIFLENYRVSLAEKVIPA
ADLSQQISTAGTEASGTGNMKFMLNGALTIGTMDGANVEMAEEAGAENLFIFGLRVEDVE
ALDRKGYNAREYYDHLPELKQAVDQISSGFFSPKEPDCFKDIVNMLMHHDRFKVFADYEA
YMQCQAQVDQLYRNPKEWTKKVIRNIACSGKFSSDRTITEYAREIWGVEPSDLQIPPPNI
PRD

>hsa:5836

MAKPLTDQEKRRQISIRGIVGVENVAELKKSFNRHLHFTLVKDRNVATTRDYYFALAHTV
RDHLVGRWIRTQQHYYDKCPKLGLDIEELEEIEEDAGLGNGGLGRLAACFLDSMATLGLA
AYGYGIRYEYGIFNQKIRDGWQVEEADDWLRYGNPWEKSRPEFMLPVHFYGKVEHTNTGT
KWIDTQVVLALPYDTPVPGYMNNTVNTMRLWSARAPNDFNLRDFNVGDYIQAVLDRNLAE
NISRVLYPNDNFFEGKELRLKQEYFVVAATLQDIIRRFKASKFGSTRGAGTVFDAFPDQV
AIQLNDTHPALAIPELMRIFVDIEKLPWSKAWELTQKTFAYTNHTVLPEALERWPVDLVE
KLLPRHLEIIYEINQKHLDRIVALFPKDVDRLRRMSLIEEEGSKRINMAHLCIVGSHAVN
GVAKIHSDIVKTKVFKDFSELEPDKFQNKTNGITPRRWLLLCNPGLAELIAEKIGEDYVK
DLSQLTKLHSFLGDDVFLRELAKVKQENKLKFSQFLETEYKVKINPSSMFDVQVKRIHEY
KRQLLNCLHVITMYNRIKKDPKKLFVPRTVIIGGKAAPGYHMAKMIIKLITSVADVVNND
PMVGSKLKVIFLENYRVSLAEKVIPATDLSEQISTAGTEASGTGNMKFMLNGALTIGTMD
GANVEMAEEAGEENLFIFGMRIDDVAALDKKGYEAKEYYEALPELKLVIDQIDNGFFSPK
QPDLFKDIINMLFYHDRFKVFADYEAYVKCQDKVSQLYMNPKAWNTMVLKNIAASGKFSS
DRTIKEYAQNIWNVEPSDLKISLSNESNKVNGN

>hsa:5837

MSRPLSDQEKRKQISVRGLAGVENVTELKKNFNRHLHFTLVKDRNVATPRDYYFALAHTV
RDHLVGRWIRTQQHYYEKDPKKISGGWQMEEADDWLRYGNPWEKARPEFTLPVHFYGHVE
HTSQGAKWVDTQVVLAMPYDTPVPGYRNNVVNTMRLWSAKAPNDFNLKDFNVGGYIQAVL
DRNLAENISRVLYPNDNFFEGKELRLKQEYFVVAATLQDIIRRFKSSKFGCRDPVRTNFD
AFPDKVAIQLNDTHPSLAIPELMRILVDLERMDWDKAWDVTVRTCAYTNHTVLPEALERW
PVHLLETLLPRHLQIIYEINQRFLNRVAAAFPGDVDRLRRMSLVEEGAVKRINMAHLCIA
GSHAVNGVARIHSEILKKTIFKDFYELEPHKFQNKTNGITPRRWLVLCNPGLAEVIAERI
GEDFISDLDQLRKLLSFVDDEAFIRDVAKVKQENKLKFAAYLEREYKVHINPNSLFDIQV
KRIHEYKRQLLNCLHVITLYNRIKREPNKFFVPRTVMIGGKAAPGYHMAKMIIRLVTAIG
DVVNHDPAVGDRLRVIFLENYRVSLAEKVIPAADLSEQISTAGTEASGTGNMKFMLNGAL
TIGTMDGANVEMAEEAGEENFFIFGMRVEDVDKLDQRGYNAQEYYDRIPELRQVIEQLSS
GFFSPKQPDLFKDIVNMLMHHDRFKVFADYEDYIKCQEKVSALYKNPREWTRMVIRNIAT
SGKFSSDRTIAQYAREIWGVEPSRQRLPAPDEAI

>hsa:586

MKDCSNGCSAECTGEGGSKEVVGTFKAKDLIVTPATILKEKPDPNNLVFGTVFTDHMLTV
EWSSEFGWEKPHIKPLQNLSLHPGSSALHYAVEVFDKEELLECIQQLVKLDQEWVPYSTS
ASLYIRPTFIGTEPSLGVKKPTKALLFVLLSPVGPYFSSGTFNPVSLWANPKYVRAWKGG
TGDCKMGGNYGSSLFAQCEAVDNGCQQVLWLYGEDHQITEVGTMNLFLYWINEDGEEELA
TPPLDGIILPGVTRRCILDLAHQWGEFKVSERYLTMDDLTTALEGNRVREMFGSGTACVV
CPVSDILYKGETIHIPTMENGPKLASRILSKLTDIQYGREESDWTIVLS

>hsa:5860

MAAAAAAGEARRVLVYGGRGALGSRCVQAFRARNWWVASVDVVENEEASASIIVKMTDSF
TEQADQVTAEVGKLLGEEKVDAILCVAGGWAGGNAKSKSLFKNCDLMWKQSIWTSTISSH
LATKHLKEGGLLTLAGAKAALDGTPGMIGYGMAKGAVHQLCQSLAGKNSGMPPGAAAIAV
LPVTLDTPMNRKSMPEADFSSWTPLEFLVETFHDWITGKNRPSSGSLIQVVTTEGRTELT
PAYF

>hsa:587

MAAAALGQLFEGMKAFKGKDQQVRLFRPWLNMDRMLRSAMRLCLPSFDKLELLECIRRLI
EVDKDWVPDAAGTSLYVRPVLIGNEPSLGVSQPTRALLFVILCPVGAYFPGGSVTPVSLL
ADPAFIRAWVGGVGNYKLGGNYGPTVLVQQEALKRGCEQVLWLYGPDHQLTEVGTMNIFV
YWTHEDGVLELVTPPLNGVILPGVVRQSLLDMAQTWGEFRVVERTITMKQLLRALEEGRV
REVFGSGTACQVCPVHRILYKDRNLHIPTMENGPELILRFQKELKEIQYGIRAHEWMFPV

>hsa:590

MHSKVTIICIRFLFWFLLLCMLIGKSHTEDDIIIATKNGKVRGMNLTVFGGTVTAFLGIP
YAQPPLGRLRFKKPQSLTKWSDIWNATKYANSCCQNIDQSFPGFHGSEMWNPNTDLSEDC
LYLNVWIPAPKPKNATVLIWIYGGGFQTGTSSLHVYDGKFLARVERVIVVSMNYRVGALG
FLALPGNPEAPGNMGLFDQQLALQWVQKNIAAFGGNPKSVTLFGESAGAASVSLHLLSPG
SHSLFTRAILQSGSFNAPWAVTSLYEARNRTLNLAKLTGCSRENETEIIKCLRNKDPQEI
LLNEAFVVPYGTPLSVNFGPTVDGDFLTDMPDILLELGQFKKTQILVGVNKDEGTAFLVY
GAPGFSKDNNSIITRKEFQEGLKIFFPGVSEFGKESILFHYTDWVDDQRPENYREALGDV
VGDYNFICPALEFTKKFSEWGNNAFFYYFEHRSSKLPWPEWMGVMHGYEIEFVFGLPLER
RDNYTKAEEILSRSIVKRWANFAKYGNPNETQNNSTSWPVFKSTEQKYLTLNTESTRIMT
KLRAQQCRFWTSFFPKVLEMTGNIDEAEWEWKAGFHRWNNYMMDWKNQFNDYTSKKESCV
GL

>hsa:5972

MDGWRRMPRWGLLLLLWGSCTFGLPTDTTTFKRIFLKRMPSIRESLKERGVDMARLGPEW
SQPMKRLTLGNTTSSVILTNYMDTQYYGEIGIGTPPQTFKVVFDTGSSNVWVPSSKCSRL
YTACVYHKLFDASDSSSYKHNGTELTLRYSTGTVSGFLSQDIITVGGITVTQMFGEVTEM
PALPFMLAEFDGVVGMGFIEQAIGRVTPIFDNIISQGVLKEDVFSFYYNRDSENSQSLGG
QIVLGGSDPQHYEGNFHYINLIKTGVWQIQMKGVSVGSSTLLCEDGCLALVDTGASYISG
STSSIEKLMEALGAKKRLFDYVVKCNEGPTLPDISFHLGGKEYTLTSADYVFQESYSSKK
LCTLAIHAMDIPPPTGPTWALGATFIRKFYTEFDRRNNRIGFALAR

>hsa:5979

MAKATSGAAGLRLLLLLLLPLLGKVALGLYFSRDAYWEKLYVDQAAGTPLLYVHALRDAP
EEVPSFRLGQHLYGTYRTRLHENNWICIQEDTGLLYLNRSLDHSSWEKLSVRNRGFPLLT
VYLKVFLSPTSLREGECQWPGCARVYFSFFNTSFPACSSLKPRELCFPETRPSFRIRENR
PPGTFHQFRLLPVQFLCPNISVAYRLLEGEGLPFRCAPDSLEVSTRWALDREQREKYELV

AVCTVHAGAREEVVMVPFPVTVYDEDDSAPTFPAGVDTASAVVEFKRKEDTVVATLRVFD
ADVVPASGELVRRYTSTLLPGDTWAQQTFRVEHWPNETSVQANGSFVRATVHDYRLVLNR
NLSISENRTMQLAVLVNDSDFQGPGAGVLLLHFNVSVLPVSLHLPSTYSLSVSRRARRFA
QIGKVCVENCQAFSGINVQYKLHSSGANCSTLGVVTSAEDTSGILFVNDTKALRRPKCAE
LHYMVVATDQQTSRQAQAQLLVTVEGSYVAEEAGCPLSCAVSKRRLECEECGGLGSPTGR
CEWRQGDGKGITRNFSTCSPSTKTCPDGHCDVVETQDINICPQDCLRGSIVGGHEPGEPR
GIKAGYGTCNCFPEEEKCFCEPEDIQDPLCDELCRTVIAAAVLFSFIVSVLLSAFCIHCY
HKFAHKPPISSAEMTFRRPAQAFPVSYSSSGARRPSLDSMENQVSVDAFKILEDPKWEFP
RKNLVLGKTLGEGEFGKVVKATAFHLKGRAGYTTVAVKMLKENASPSELRDLLSEFNVLK
QVNHPHVIKLYGACSQDGPLLLIVEYAKYGSLRGFLRESRKVGPGYLGSGGSRNSSSLDH
PDERALTMGDLISFAWQISQGMQYLAEMKLVHRDLAARNILVAEGRKMKISDFGLSRDVY
EEDSYVKRSQGRIPVKWMAIESLFDHIYTTQSDVWSFGVLLWEIVTLGGNPYPGIPPERL
FNLLKTGHRMERPDNCSEEMYRLMLQCWKQEPDKRPVFADISKDLEKMMVKRRDYLDLAA
STPSDSLIYDDGLSEEETPLVDCNNAPLPRALPSTWIENKLYGRISHAFTRF
>hsa:6098
MKNIYCLIPKLVNFATLGCLWISVVQCTVLNSCLKSCVTNLGQQLDLGTPHNLSEPCIQG
CHFWNSVDQKNCALKCRESCEVGCSSAEGAYEEEVLENADLPTAPFASSIGSHNMTLRWK
SANFSGVKYIIQWKYAQLLGSWTYTKTVSRPSYVVKPLHPFTEYIFRVVWIFTAQLQLYS
PPSPSYRTHPHGVPETAPLIRNIESSSPDTVEVSWDPPQFPGGPILGYNLRLISKNQKLD
AGTQRTSFQFYSTLPNTIYRFSIAAVNEVGEGPEAESSITTSSSAVQQEEQWLFLSRKTS
LRKRSLKHLVDEAHCLRLDAIYHNITGISVDVHQQIVYFSEGTLIWAKKAANMSDVSDLR
IFYRGSGLISSISIDWLYQRMYFIMDELVCVCDLENCSNIEEITPPSISAPQKIVADSYN
GYVFYLLRDGIYRADLPVPSGRCAEAVRIVESCTLKDFAIKPQAKRIIYFNDTAQVFMST
FLDGSASHLILPRIPFADVKSFACENNDFLVTDGKVIFQQDALSFNEFIVGCDLSHIEEF
GFGNLVIFGSSSQLHPLPGRPQELSVLFGSHQALVQWKPPALAIGANVILISDIIELFEL
GPSAWQNWTYEVKVSTQDPPEVTHIFLNISGTMLNVPELQSAMKYKVSVRASSPKRPGPW
SEPSVGTTLVPASEPPFIMAVKEDGLWSKPLNSFGPGEFLSSDIGNVSDMDWYNNSLYYS
DTKGDVFVWLLNGTDISENYHLPSIAGAGALAFEWLGHFLYWAGKTYVIQRQSVLTGHTD
IVTHVKLLVNDMVVDSVGGYLYWTTLYSVESTRLNGESSLVLQTQPWFSGKKVIALTLDL
SDGLLYWLVQDSQCIHLYTAVLRGQSTGDTTITEFAAWSTSEISQNALMYYSGRLFWING
FRIITTQEIGQKTSVSVLEPARFNQFTIIQTSLKPLPGNFSFTPKVIPDSVQESSFRIEG
NASSFQILWNGPPAVDWGVVFYSVEFSAHSKFLASEQHSLPVFTVEGLEPYALFNLSVTP
YTYWGKGPKTSLSLRAPETVPSAPENPRIFILPSGKCCNKNEVVVEFRWNKPKHENGVLT
KFEIFYNISNQSITNKTCEDWIAVNVTPSVMSFQLEGMSPRCFIAFQVRAFTSKGPGPYA
DVVKSTTSEINPFPHLITLLGNKIVFLDMDQNQVVWTFSAERVISAVCYTADNEMGYYAE
GDSLFLLHLHNRSSSELFQDSLVFDITVITIDWISRHLYFALKESQNGMQVFDVDLEHKV
KYPREVKIHNRNSTIISFSVYPLLSRLYWTEVSNFGYQMFYYSIISHTLHRILQPTATNQ
QNKRNQCSCNVTEFELSGAMAIDTSNLEKPLIYFAKAQEIWAMDLEGCQCWRVITVPAML
AGKTLVSLTVDGDLIYWIITAKDSTQIYQAKKGNGAIVSQVKALRSRHILAYSSVMQPFP
DKAFLSLASDTVEPTILNATNTSLTIRLPLAKTNLTWYGITSPTPTYLVYYAEVNDRKNS
SDLKYRILEFQDSIALIEDLQPFSTYMIQIAVKNYYSDPLEHLPPGKEIWGKTKNGVPEA
VQLINTTVRSDTSLIISWRESHKPNGPKESVRYQLAISHLALIPETPLRQSEFPNGRLTL
LVTRLSGGNIYVLKVLACHSEEMWCTESHPVTVEMFNTPEKPYSLVPENTSLQFNWKAPL
NVNLIRFWVELQKWKYNEFYHVKTSCSQGPAYVCNITNLQPYTSYNVRVVVVYKTGENST

SLPESFKTKAGVPNKPGIPKLLEGSKNSIQWEKAEDNGCRITYYILEIRKSTSNNLQNQN
LRWKMTFNGSCSSVCTWKSKNLKGIFQFRVVAANNLGFGEYSGISENIILVGDDFWIPET
SFILTIIVGIFLVVTIPLTFVWHRRLKNQKSAKEGVTVLINEDKELAELRGLAAGVGLAN
ACYAIHTLPTQEEIENLPAFPREKLTLRLLLGSGAFGEVYEGTAVDILGVGSGEIKVAVK
TLKKGSTDQEKIEFLKEAHLMSKFNHPNILKQLGVCLLNEPQYIILELMEGGDLLTYLRK
ARMATFYGPLLTLVDLVDLCVDISKGCVYLERMHFIHRDLAARNCLVSVKDYTSPRIVKI
GDFGLARDIYKNDYYRKRGEGLLPVRWMAPESLMDGIFTTQSDVWSFGILIWEILTLGHQ
PYPAHSNLDVLNYVQTGGRLEPPRNCPDDLWNLMTQCWAQEPDQRPTFHRIQDQLQLFRN
FFLNSIYKSRDEANNSGVINESFEGEDGDVICLNSDDIMPVALMETKNREGLNYMVLATE
CGQGEEKSEGPLGSQESESCGLRKEEKEPHADKDFCQEKQVAYCPSGKPEGLNYACLTHS
GYGDGSD
>hsa:613
MVDPVGFAEAWKAQFPDSEPPRMELRSVGDIEQELERCKASIRRLEQEVNQERFRMIYLQ
TLLAKEKKSYDRQRWGFRRAAQAPDGASEPRASASRPQPAPADGADPPPAEEPEARPDGE
GSPGKARPGTARRPGAAASGERDDRGPPASVAALRSNFERIRKGHGQPGADAEKPFYVNV
EFHHERGLVKVNDKEVSDRISSLGSQAMQMERKKSQHGAGSSVGDASRPPYRGRSSESSC
GVDGDYEDAELNPRFLKDNLIDANGGSRPPWPPLEYQPYQSIYVGGMMEGEGKGPLLRSQ
STSEQEKRLTWPRRSYSPRSFEDCGGGYTPDCSSNENLTSSEEDFSSGQSSRVSPSPTTY
RMFRDKSRSPSQNSQQSFDSSSPPTPQCHKRHRHCPVVVSEATIVGVRKTGQIWPNDGEG
AFHGDADGSFGTPPGYGCAADRAEEQRRHQDGLPYIDDSPSSSPHLSSKGRGSRDALVSG
ALESTKASELDLEKGLEMRKWVLSGILASEETYLSHLEALLLPMKPLKAAATTSQPVLTS
QQIETIFFKVPELYEIHKEFYDGLFPRVQQWSHQQRVGDLFQKLASQLGVYRAFVDNYGV
AMEMAEKCCQANAQFAEISENLRARSNKDAKDPTTKNSLETLLYKPVDRVTRSTLVLHDL
LKHTPASHPDHPLLQDALRISQNFLSSINEEITPRRQSMTVKKGEHRQLLKDSFMVELVE
GARKLRHVFLFTDLLLCTKLKKQSGGKTQQYDCKWYIPLTDLSFQMVDELEAVPNIPLVP
DEELDALKIKISQIKNDIQREKRANKGSKATERLKKKLSEQESLLLLMSPSMAFRVHSRN
GKSYTFLISSDYERAEWRENIREQQKKCFRSFSLTSVELQMLTNSCVKLQTVHSIPLTIN
KEDDESPGLYGFLNVIVHSATGFKQSSNLYCTLEVDSFGYFVNKAKTRVYRDTAEPNWNE
EFEIELEGSQTLRILCYEKCYNKTKIPKEDGESTDRLMGKGQVQLDPQALQDRDWQRTVI
AMNGIEVKLSVKFNSREFSLKRMPSRKQTGVFGVKIAVVTKRERSKVPYIVRQCVEEIER
RGMEEVGIYRVSGVATDIQALKAAFDVNNKDVSVMMSEMDVNAIAGTLKLYFRELPEPLF
TDEFYPNFAEGIALSDPVAKESCMLNLLLSLPEANLLTFLFLLDHLKRVAEKEAVNKMSL
HNLATVFGPTLLRPSEKESKLPANPSQPITMTDSWSLEVMSQVQVLLYFLQLEAIPAPDS
KRQSILFSTEV
>hsa:6240
MHVIKRDGRQERVMFDKITSRIQKLCYGLNMDFVDPAQITMKVIQGLYSGVTTVELDTLA
AETAATLTTKHPDYAILAARIAVSNLHKETKKVFSDVMEDLYNYINPHNGKHSPMVAKST
LDIVLANKDRLNSAIIYDRDFSYNYFGFKTLERSYLLKINGKVAERPQHMLMRVSVGIHK
EDIDAAIETYNLLSERWFTHASPTLFNAGTNRPQLSSCFLLSMKDDSIEGIYDTLKQCAL
ISKSAGGIGVAVSCIRATGSYIAGTNGNSNGLVPMLRVYNNTARYVDQGGNKRPGAFAIY
LEPWHLDIFEFLDLKKNTGKEEQRARDLFFALWIPDLFMKRVETNQDWSLMCPNECPGLD
EVWGEEFEKLYASYEKQGRVRKVVKAQQLWYAIIESQTETGTPYMLYKDSCNRKSNQQNL
GTIKCSNLCTEIVEYTSKDEVAVCNLASLALNMYVTSEHTYDFKKLAEVTKVVVRNLNKI
IDINYYPVPEACLSNKRHRPIGIGVQGLADAFILMRYPFESAEAQLLNKQIFETIYYGAL

EASCDLAKEQGPYETYEGSPVSKGILQYDMWNVTPTDLWDWKVLKEKIAKYGIRNSLLIA
PMPTASTAQILGNNESIEPYTSNIYTRRVLSGEFQIVNPHLLKDLTERGLWHEEMKNQII
ACNGSIQSIPEIPDDLKQLYKTVWEISQKTVLKMAAERGAFIDQSQSLNIHIAEPNYGKL
TSMHFYGWKQGLKTGMYYLRTRPAANPIQFTLNKEKLKDKEKVSKEEEEKERNTAAMVCS
LENRDECLMCGS

>hsa:6241

MLSLRVPLAPITDPQQLQLSPLKGLSLVDKENTPPALSGTRVLASKTARRIFQEPTEPKT
KAAAPGVEDEPLLRENPRRFVIFPIEYHDIWQMYKKAEASFWTAEEVDLSKDIQHWESLK
PEERYFISHVLAFFAASDGIVNENLVERFSQEVQITEARCFYGFQIAMENIHSEMYSLLI
DTYIKDPKEREFLFNAIETMPCVKKKADWALRWIGDKEATYGERVVAFAAVEGIFFSGSF
ASIFWLKKRGLMPGLTFSNELISRDEGLHCDFACLMFKHLVHKPSEERVREIIINAVRIE
QEFLTEALPVKLIGMNCTLMKQYIEFVADRLMLELGFSKVFRVENPFDFMENISLEGKTN
FFEKRVGEYQRMGVMSSPTENSFTLDADF

>hsa:6259

MRGAARLGRPGRSCLPGARGLRAPPPPPLLLLLALLPLLPAPGAAAAPAPRPPELQSASA
GPSVSLYLSEDEVRRLIGLDAELYYVRNDLISHYALSFSLLVPSETNFLHFTWHAKSKVE
YKLGFQVDNVLAMDMPQVNISVQGEVPRTLSVFRVELSCTGKVDSEVMILMQLNLTVNSS
KNFTVLNFKRRKMCYKKLEEVKTSALDKNTSRTIYDPVHAAPTTSTRVFYISVGVCCAVI
FLVAIILAVLHLHSMKRIELDDSISASSSSQGLSQPSTQTTQYLRADTPNNATPITSSLG
YPTLRIEKNDLRSVTLLEAKGKVKDIAISRERITLKDVLQEGTFGRIFHGILIDEKDPNK
EKQAFVKTVKDQASEIQVTMMLTESCKLRGLHHRNLLPITHVCIEEGEKPMVILPYMNWG
NLKLFLRQCKLVEANNPQAISQQDLVHMAIQIACGMSYLARREVIHKDLAARNCVIDDTL
QVKITDNALSRDLFPMDYHCLGDNENRPVRWMALESLVNNEFSSASDVWAFGVTLWELMT
LGQTPYVDIDPFEMAAYLKDGYRIAQPINCPDELFAVMACCWALDPEERPKFQQLVQCLT
EFHAALGAYV

>hsa:6300

MSSPPPARSGFYRQEVTKTAWEVRAVYRDLQPVGSGAYGAVCSAVDGRTGAKVAIKKLYR
PFQSELFAKRAYRELRLLKHMRHENVIGLLDVFTPDETLDDFTDFYLVMPFMGTDLGKLM
KHEKLGEDRIQFLVYQMLKGLRYIHAAGIIHRDLKPGNLAVNEDCELKILDFGLARQADS
EMTGYVVTRWYRAPEVILNWMRYTQTVDIWSVGCIMAEMITGKTLFKGSDHLDQLKEIMK
VTGTPPAEFVQRLQSDEAKNYMKGLPELEKKDFASILTNASPLAVNLLEKMLVLDAEQRV
TAGEALAHPYFESLHDTEDEPQVQKYDDSFDDVDRTLDEWKRVTYKEVLSFKPPRQLGAR
VSKETPL

>hsa:63036

MIRTLLLSTLVAGALSCGDPTYPPYVTRVVGGEEARPNSWPWQVSLQYSSNGKWYHTCGG
SLIANSWVLTAAHCISSSRTYRVGLGRHNLYVAESGSLAVSVSKIVVHKDWNSNQISKGN
DIALLKLANPVSLTDKIQLACLPPAGTILPNNYPCYVTGWGRLQTNGAVPDVLQQGRLLV
VDYATCSSSAWWGSSVKTSMICAGGDGVISSCNGDSGGPLNCQASDGRWQVHGIVSFGSR
LGCNYYHKPSVFTRVSNYIDWINSVIANN

>hsa:635

MPPVGGKKAKKGILERLNAGEIVIGDGGFVFALEKRGYVKAGPWTPEAAVEHPEAVRQLH
REFLRAGSNVMQTFTFYASEDKLENRGNYVLEKISGQEVNEAACDIARQVADEGDALVAG
GVSQTPSYLSCKSETEVKKVFLQQLEVFMKKNVDFLIAEYFEHVEEAVWAVETLIASGKP
VAATMCIGPEGDLHGVPPGECAVRLVKAGASIIGVNCHFDPTISLKTVKLMKEGLEAARL

KAHLMSQPLAYHTPDCNKQGFIDLPEFPFGLEPRVATRWDIQKYAREAYNLGVRYIGGCC
GFEPYHIRAIAEELAPERGFLPPASEKHGSWGSGLDMHTKPWVRARARKEYWENLRIASG
RPYNPSMSKPDGWGVTKGTAELMQQKEATTEQQLKELFEKQKFKSQ

>hsa:63904

MTASASSFSSSQGVQQPSIYSFSQITRSLFLSNGVAANDKLLLSSNRITAIVNASVEVVN
VFFEGIQYIKVPVTDARDSRLYDFFDPIADLIHTIDMRQGRTLLHCMAGVSRSASLCLAY
LMKYHSMSLLDAHTWTKSRRPIIRPNNGFWEQLINYEFKLFNNNTVRMINSPVGNIPDIY
EKDLRMMISM

>hsa:64087

MWAVLRLALRPCARASPAGPRAYHGDSVASLGTQPDLGSALYQENYKQMKALVNQLHERV
EHIKLGGGEKARALHISRGKLLPRERIDNLIDPGSPFLELSQFAGYQLYDNEEVPGGGII
TGIGRVSGVECMIIANDATVKGGAYYPVTVKKQLRAQEIAMQNRLPCIYLVDSGGAYLPR
QADVFPDRDHFGRTFYNQAIMSSKNIAQIAVVMGSCTAGGAYVPAMADENIIVRKQGTIF
LAGPPLVKAATGEEVSAEDLGGADLHCRKSGVSDHWALDDHHALHLTRKVVRNLNYQKKL
DVTIEPSEEPLFPADELYGIVGANLKRSFDVREVIARIVDGSRFTEFKAFYGDTLVTGFA
RIFGYPVGIVGNNGVLFSESAKKGTHFVQLCCQRNIPLLFLQNITGFMVGREYEAEGIAK
DGAKMVAAVACAQVPKITLIIGGSYGAGNYGMCGRAYSPRFLYIWPNARISVMGGEQAAN
VLATITKDQRAREGKQFSSADEAALKEPIIKKFEEEGNPYYSSARVWDDGIIDPADTRLV
LGLSFSAALNAPIEKTDFGIFRM

>hsa:6416

MAAPSPSGGGGSGGGSGSGTPGPVGSPAPGHPAVSSMQGKRKALKLNFANPPFKSTARFT
LNPNPTGVQNPHIERLRTHSIESSGKLKISPEQHWDFTAEDLKDLGEIGRGAYGSVNKMV
HKPSGQIMAVKRIRSTVDEKEQKQLLMDLDVVMRSSDCPYIVQFYGALFREGDCWICMEL
MSTSFDKFYKYVYSVLDDVIPEEILGKITLATVKALNHLKENLKIIHRDIKPSNILLDRS
GNIKLCDFGISGQLVDSIAKTRDAGCRPYMAPERIDPSASRQGYDVRSDVWSLGITLYEL
ATGRFPYPKWNSVFDQLTQVVKGDPPQLSNSEEREFSPSFINFVNLCLTKDESKRPKYKE
LLKHPFILMYEERAVEVACYVCKILDQMPATPSSPMYVD

>hsa:64499

MLNLLLLALPVLASRAYAAPAPGQALQRVGIVGGQEAPRSKWPWQVSLRVRDRYWMHFCG
GSLIHPQWVLTAAHCVGPDVKDLAALRVQLREQHLYYQDQLLPVSRIIVHPQFYTAQIGA
DIALLELEEPVNVSSHVHTVTLPPASETFPPGMPCWVTGWGDVDNDERLPPPFPLKQVKV
PIMENHICDAKYHLGAYTGDDVRIVRDDMLCAGNTRRDSCQGDSGGPLVCKVNGTWLQAG
VVSWGEGCAQPNRPGIYTRVTYYLDWIHHYVPKKP

>hsa:645

MAVKKIAIFGATGQTGLTTLAQAVQAGYEVTVLVRDSSRLPSEGPRPAHVVVGDVLQAAD
VDKTVAGQDAVIVLLGTRNDLSPTTVMSEGARNIVAAMKAHGVDKVVACTSAFLLWDPTK
VPPRLQAVTDDHIRMHKVLRESGLKYVAVMPPHIGDQPLTGAYTVTLDGRGPSRVISKHD
LGHFMLRCLTTDEYDGHSTYPSHQYQ

>hsa:64600

MADGAKANPKGFKKKVLDRCFSGWRGPRFGASCPSRTSRSSLGMKKFFTVAILAGSVLST
AHGSLLNLKAMVEAVTGRSAILSFVGYGCYCGLGGRGQPKDEVDWCCHAHDCCYQELFDQ
GCHPYVDHYDHTIENNTEIVCSDLNKTECDKQTCMCDKNMVLCLMNQTYREEYRGFLNVY
CQGPTPNCSIYEPPPEEVTCSHQSPAPPAPP

>hsa:64802

MENSEKTEVVLLACGSFNPITNMHLRLFELAKDYMNGTGRYTVVKGIISPVGDAYKKKGL
IPAYHRVIMAELATKNSKWVEVDTWESLQKEWKETLKVLRHHQEKLEASDCDHQQNSPTL
ERPGRKRKWTETQDSSQKKSLEPKTKAVPKVKLLCGADLLESFAVPNLWKSEDITQIVAN
YGLICVTRAGNDAQKFIYESDVLWKHRSNIHVVNEWIANDISSTKIRRALRRGQSIRYLV
PDLVQEYIEKHNLYSSESEDRNAGVILAPLQRNTAEAKT
>hsa:64816
MDLIPNFAMETWVLVATSLVLLYIYGTHSHKLFKKLGIPGPTPLPFLGTILFYLRGLWNF
DRECNEKYGEMWGLYEGQQPMLVIMDPDMIKTVLVKECYSVFTNQMPLGPMGFLKSALSF
AEDEEWKRIRTLLSPAFTSVKFKEMVPIISQCGDMLVRSLRQEAENSKSINLKDFFGAYT
MDVITGTLFGVNLDSLNNPQDPFLKNMKKLLKLDFLDPFLLLISLFPFLTPVFEALNIGL
FPKDVTHFLKNSIERMKESRLKDKQKHRVDFFQQMIDSQNSKETKSHKALSDLELVAQSI
IIIFAAYDTTSTTLPFIMYELATHPDVQQKLQEEIDAVLPNKAPVTYDALVQMEYLDMVV
NETLRLFPVVSRVTRVCKKDIEINGVFIPKGLAVMVPIYALHHDPKYWTEPEKFCPESRF
SKKNKDSIDLYRYIPFGAGPRNCIGMRFALTNIKLAVIRALQNFSFKPCKETQIPLKLDN
LPILQPEKPIVLKVHLRDGITSGP
>hsa:64850
MCELYSKRDTLGLRKKHIGPSCKVFFASDPIKIVRAQRQYMFDENGEQYLDCINNVAHGV
VKAALKQMELLNTNSRFLHDNIVEYAKRLSATLPEKLSVCYFTNSGSEANDLALRLARQF
RGHQDVITLDHAYHGHLSSLIEISPYKFQKGKDVKKEFVHVAPTPDTYRGKYREDHADSA
SAYADEVKKIIEDAHNSGRKIAAFIAESMQSCGGQIIPPAGYFQKVAEYVHGAGGVFIAD
EVQVGFGRVGKHFWSFQMYGEDFVPDIVTMGKPMGNGHPVACVVTTKEIAEAFSSSGMEY
FNTYGGNPVSCAVGLAVLDIIENEDLQGNAKRVGNYLTELLKKQKAKHTLIGDIRGIGLF
IGIDLVKDHLKRTPATAEAQHIIYKMKEKRVLLSADGPHRNVLKIKPPMCFTEEDAKFMV
DQLDRILTVLEEAMGTKTESVTSENTPCKTKMLKEAHIELLRDSTTDSKENPSRKRNGMC
TDTHSLLSKRLKT
>hsa:64902
MTLIWRHLLRPLCLVTSAPRILEMHPFLSLGTSRTSVTKLSLHTKPRMPPCDFMPERYQS
LGYNRVLEIHKEHLSPVVTAYFQKPLLLHQGHMEWLFDAEGSRYLDFFSGIVTVSVGHCH
PKVNAVAQKQLGRLWHTSTVFFHPPMHEYAEKLAALLPEPLKVIFLVNSGSEANELAMLM
ARAHSNNIDIISFRGAYHGCSPYTLGLTNVGTYKMELPGGTGCQPTMCPDVFRGPWGGSH
CRDSPVQTIRKCSCAPDCCQAKDQYIEQFKDTLSTSVAKSIAGFFAEPIQGVNGVVQYPK
GFLKEAFELVRARGGVCIADEVQTGFGRLGSHFWGFQTHDVLPDIVTMAKGIGNGFPMAA
VITTPEIAKSLAKCLQHFNTFGGNPMACAIGSAVLEVIKEENLQENSQEVGTYMLLKFAK
LRDEFEIVGDVRGKGLMIGIEMVQDKISCRPLPREEVNQIHEDCKHMGLLVGRGSIFSQT
FRIAPSMCITKPEVDFAVEVFRSALTQHMERRAK
>hsa:657
MPQLYIYIRLLGAYLFIISRVQGQNLDSMLHGTGMKSDSDQKKSENGVTLAPEDTLPFLK
CYCSGHCPDDAINNTCITNGHCFAIIEEDDQGETTLASGCMKYEGSDFQCKDSPKAQLRR
TIECCRTNLCNQYLQPTLPPVVIGPFFDGSIRWLVLLISMAVCIIAMIIFSSCFCYKHYC
KSISSRRRYNRDLEQDEAFIPVGESLKDLIDQSQSSGSGSGLPLLVQRTIAKQIQMVRQV
GKGRYGEVWMGKWRGEKVAVKVFFTTEEASWFRETEIYQTVLMRHENILGFIAADIKGTG
SWTQLYLITDYHENGSLYDFLKCATLDTRALLKLAYSAACGLCHLHTEIYGTQGKPAIAH
RDLKSKNILIKKNGSCCIADLGLAVKFNSDTNEVDVPLNTRVGTKRYMAPEVLDESLNKN
HFQPYIMADIYSFGLIIWEMARRCITGGIVEEYQLPYYNMVPSDPSYEDMREVVCVKRLR

PIVSNRWNSDECLRAVLKLMSECWAHNPASRLTALRIKKTLAKMVESQDVKI
>hsa:658
MLLRSAGKLNVGTKKEDGESTAPTPRPKVLRCKCHHHCPEDSVNNICSTDGYCFTMIEED
DSGLPVVTSGCLGLEGSDFQCRDTPIPHQRRSIECCTERNECNKDLHPTLPPLKNRDFVD
GPIHHRALLISVTVCSLLLVLIILFCYFRYKRQETRPRYSIGLEQDETYIPPGESLRDLI
EQSQSSGSGSGLPLLVQRTIAKQIQMVKQIGKGRYGEVWMGKWRGEKVAVKVFFTTEEAS
WFRETEIYQTVLMRHENILGFIAADIKGTGSWTQLYLITDYHENGSLYDYLKSTTLDAKS
MLKLAYSSVSGLCHLHTEIFSTQGKPAIAHRDLKSKNILVKKNGTCCIADLGLAVKFISD
TNEVDIPPNTRVGTKRYMPPEVLDESLNRNHFQSYIMADMYSFGLILWEVARRCVSGGIV
EEYQLPYHDLVPSDPSYEDMREIVCIKKLRPSFPNRWSSDECLRQMGKLMTECWAHNPAS
RLTALRVKKTLAKMSESQDIKL
>hsa:660
MDTKSILEELLLKRSQQKKKMSPNNYKERLFVLTKTNLSYYEYDKMKRGSRKGSIEIKKI
RCVEKVNLEEQTPVERQYPFQIVYKDGLLYVYASNEESRSQWLKALQKEIRGNPHLLVKY
HSGFFVDGKFLCCQQSCKAAPGCTLWEAYANLHTAVNEEKHRVPTFPDRVLKIPRAVPVL
KMDAPSSSTTLAQYDNESKKNYGSQPPSSSTSLAQYDSNSKKIYGSQPNFNMQYIPREDF
PDWWQVRKLKSSSSSEDVASSNQKERNVNHTTSKISWEFPESSSSEEEENLDDYDWFAGN
ISRSQSEQLLRQKGKEGAFMVRNSSQVGMYTVSLFSKAVNDKKGTVKHYHVHTNAENKLY
LAENYCFDSIPKLIHYHQHNSAGMITRLRHPVSTKANKVPDSVSLGNGIWELKREEITLL
KELGSGQFGVVQLGKWKGQYDVAVKMIKEGSMSEDEFFQEAQTMMKLSHPKLVKFYGVCS
KEYPIYIVTEYISNGCLLNYLRSHGKGLEPSQLLEMCYDVCEGMAFLESHQFIHRDLAAR
NCLVDRDLCVKVSDFGMTRYVLDDQYVSSVGTKFPVKWSAPEVFHYFKYSSKSDVWAFGI
LMWEVFSLGKQPYDLYDNSQVVLKVSQGHRLYRPHLASDTIYQIMYSCWHELPEKRPTFQ
QLLSSIEPLREKDKH
>hsa:6609
MPRYGASLRQSCPRSGREQGQDGTAGAPGLLWMGLVLALALALALALALSDSRVLWAPAE
AHPLSPQGHPARLHRIVPRLRDVFGWGNLTCPICKGLFTAINLGLKKEPNVARVGSVAIK
LCNLLKIAPPAVCQSIVHLFEDDMVEVWRRSVLSPSEACGLLLGSTCGHWDIFSSWNISL
PTVPKPPPKPPSPPAPGAPVSRILFLTDLHWDHDYLEGTDPDCADPLCCRRGSGLPPASR
PGAGYWGEYSKCDLPLRTLESLLSGLGPAGPFDMVYWTGDIPAHDVWHQTRQDQLRALTT
VTALVRKFLGPVPVYPAVGNHESTPVNSFPPPFIEGNHSSRWLYEAMAKAWEPWLPAEAL
RTLRIGGFYALSPYPGLRLISLNMNFCSRENFWLLINSTDPAGQLQWLVGELQAAEDRGD
KVHIIGHIPPGHCLKSWSWNYYRIVARYENTLAAQFFGHTHVDEFEVFYDEETLSRPLAV
AFLAPSATTYIGLNPGYRVYQIDGNYSGSSHVVLDHETYILNLTQANIPGAIPHWQLLYR
ARETYGLPNTLPTAWHNLVYRMRGDMQLFQTFWFLYHKGHPPSEPCGTPCRLATLCAQLS
ARADSPALCRHLMPDGSLPEAQSLWPRPLFC
>hsa:6610
MKPNFSLRLRIFNLNCWGIPYLSKHRADRMRRLGDFLNQESFDLALLEEVWSEQDFQYLR
QKLSPTYPAAHHFRSGIIGSGLCVFSKHPIQELTQHIYTLNGYPYMIHHGDWFSGKAVGL
LVLHLSGMVLNAYVTHLHAEYNRQKDIYLAHRVAQAWELAQFIHHTSKKADVVLLCGDLN
MHPEDLGCCLLKEWTGLHDAYLETRDFKGSEEGNTMVPKNCYVSQQELKPFPFGVRIDYV
LYKAVSGFYISCKSFETTTGFDPHRGTPLSDHEALMATLFVRHSPPQQNPSSTHGPAERS
PLMCVLKEAWTELGLGMAQARWWATFASYVIGLGLLLLALLCVLAAGGGAGEAAILLWTP
SVGLVLWAGAFYLFHVQEVNGLYRAQAELQHVLGRAREAQDLGPEPQPALLLGQQEGDRT

KEQ
>hsa:6646
MELKPFFMKEVGSHFDDFVTNLIEKSASLDNGGCALTTFSVLEGEKNNHRAKDLRAPPEQ
GKIFIARRSLLDELLEVDHIRTIYHMFIALLILFILSTLVVDYIDEGRLVLEFSLLSYAF
GKFPTVVWTWWIMFLSTFSVPYFLFQHWATGYSKSSHPLIRSLFHGFLFMIFQIGVLGFG
PTYVVLAYTLPPASRFIIIFEQIRFVMKAHSFVRENVPRVLNSAKEKSSTVPIPTVNQYL
YFLFAPTLIYRDSYPRNPTVRWGYVAMKFAQVFGCFFYVYYIFERLCAPLFRNIKQEPFS
ARVLVLCVFNSILPGVLILFLTFFAFLHCWLNAFAEMLRFGDRMFYKDWWNSTSYSNYYR
TWNVVVHDWLYYYAYKDFLWFFSKRFKSAAMLAVFAVSAVVHEYALAVCLSFFYPVLFVL
FMFFGMAFNFIVNDSRKKPIWNVLMWTSLFLGNGVLLCFYSQEWYARQHCPLKNPTFLDY
VRPRSWTCRYVF
>hsa:6652
MAAAAKPNNLSLVVHGPGDLRLENYPIPEPGPNEVLLRMHSVGICGSDVHYWEYGRIGNF
IVKKPMVLGHEASGTVEKVGSSVKHLKPGDRVAIEPGAPRENDEFCKMGRYNLSPSIFFC
ATPPDDGNLCRFYKHNAAFCYKLPDNVTFEEGALIEPLSVGIHACRRGGVTLGHKVLVCG
AGPIGMVTLLVAKAMGAAQVVVTDLSATRLSKAKEIGADLVLQISKESPQEIARKVEGQL
GCKPEVTIECTGAEASIQAGIYATRSGGNLVLVGLGSEMTTVPLLHAAIREVDIKGVFRY
CNTWPVAISMLASKSVNVKPLVTHRFPLEKALEAFETFKKGLGLKIMLKCDPSDQNP
>hsa:670
MVAVLGGRGVLRLRLLLSALKPGIHVPRAGPAAAFGTSVTSAKVAVNGVQLHYQQTGEGD
HAVLLLPGMLGSGETDFGPQLKNLNKKLFTVVAWDPRGYGHSRPPDRDFPADFFERDAKD
AVDLMKALKFKKVSLLGWSDGGITALIAAAKYPSYIHKMVIWGANAYVTDEDSMIYEGIR
DVSKWSERTRKPLEALYGYDYFARTCEKWVDGIRQFKHLPDGNICRHLLPRVQCPALIVH
GEKDPLVPRFHADFIHKHVKGSRLHLMPEGKHNLHLRFADEFNKLAEDFLQ
>hsa:6713
MWTFLGIATFTYFYKKFGDFITLANREVLLCVLVFLSLGLVLSYRCRHRNGGLLGRQQSG
SQFALFSDILSGLPFIGFFWAKSPPESENKEQLEARRRRKGTNISETSLIGTAACTSTSS
QNDPEVIIVGAGVLGSALAAVLSRDGRKVTVIERDLKEPDRIVGEFLQPGGYHVLKDLGL
GDTVEGLDAQVVNGYMIHDQESKSEVQIPYPLSENNQVQSGRAFHHGRFIMSLRKAAMAE
PNAKFIEGVVLQLLEEDDVVMGVQYKDKETGDIKELHAPLTVVADGLFSKFRKSLVSNKV
SVSSHFVGFLMKNAPQFKANHAELILANPSPVLIYQISSSETRVLVDIRGEMPRNLREYM
VEKIYPQIPDHLKEPFLEATDNSHLRSMPASFLPPSSVKKRGVLLLGDAYNMRHPLTGGG
MTVAFKDIKLWRKLLKGIPDLYDDAAIFEAKKSFYWARKTSHSFVVNILAQALYELFSAT
DDSLHQLRKACFLYFKLGGECVAGPVGLLSVLSPNPLVLIGHFFAVAIYAVYFCFKSEPW
ITKPRALLSSGAVLYKACSVIFPLIYSEMKYMVH
>hsa:6714
MGSNKSKPKDASQRRRSLEPAENVHGAGGGAFPASQTPSKPASADGHRGPSAAFAPAAAE
PKLFGGFNSSDTVTSPQRAGPLAGGVTTFVALYDYESRTETDLSFKKGERLQIVNNTEGD
WWLAHSLSTGQTGYIPSNYVAPSDSIQAEEWYFGKITRRESERLLLNAENPRGTFLVRES
ETTKGAYCLSVSDFDNAKGLNVKHYKIRKLDSGGFYITSRTQFNSLQQLVAYYSKHADGL
CHRLTTVCPTSKPQTQGLAKDAWEIPRESLRLEVKLGQGCFGEVWMGTWNGTTRVAIKTL
KPGTMSPEAFLQEAQVMKKLRHEKLVQLYAVVSEEPIYIVTEYMSKGSLLDFLKGETGKY
LRLPQLVDMAAQIASGMAYVERMNYVHRDLRAANILVGENLVCKVADFGLARLIEDNEYT
ARQGAKFPIKWTAPEAALYGRFTIKSDVWSFGILLTELTTKGRVPYPGMVNREVLDQVER

GYRMPCPPECPESLHDLMCQCWRKEPEERPTFEYLQAFLEDYFTSTEPQYQPGENL
>hsa:6716
MQVQCQQSPVLAGSATLVALGALALYVAKPSGYGKHTESLKPAATRLPARAAWFLQELPS
FAVPAGILARQPLSLFGPPGTVLLGLFCLHYFHRTFVYSLLNRGRPYPAILILRGTAFCT
GNGVLQGYYLIYCAEYPDGWYTDIRFSLGVFLFILGMGINIHSDYILRQLRKPGEISYRI
PQGGLFTYVSGANFLGEIIEWIGYALATWSLPALAFAFFSLCFLGLRAFHHHRFYLKMFE
DYPKSRKALIPFIF
>hsa:6725
MEPFLRRRLAFLSFFWDKIWPAGGEPDHGTPGSLDPNTDPVPTLPAEPCSPFPQLFLALY
DFTARCGGELSVRRGDRLCALEEGGGYIFARRLSGQPSAGLVPITHVAKASPETLSDQPW
YFSGVSRTQAQQLLLSPPNEPGAFLIRPSESSLGGYSLSVRAQAKVCHYRVSMAADGSLY
LQKGRLFPGLEELLTYYKANWKLIQNPLLQPCMPQKAPRQDVWERPHSEFALGRKLGEGY
FGEVWEGLWLGSLPVAIKVIKSANMKLTDLAKEIQTLKGLRHERLIRLHAVCSGGEPVYI
VTELMRKGNLQAFLGTPEGRALRLPPLLGFACQVAEGMSYLEEQRVVHRDLAARNVLVDD
GLACKVADFGLARLLKDDIYSPSSSSKIPVKWTAPEAANYRVFSQKSDVWSFGVLLHEVF
TYGQCPYEGMTNHETLQQIMRGYRLPRPAACPAEVYVLMLECWRSSPEERPSFATLREKL
HAIHRCHP
>hsa:6768
MGSDRARKGGGGPKDFGAGLKYNSRHEKVNGLEEGVEFLPVNNVKKVEKHGPGRWVVLAA
VLIGLLLVLLGIGFLVWHLQYRDVRVQKVFNGYMRITNENFVDAYENSNSTEFVSLASKV
KDALKLLYSGVPFLGPYHKESAVTAFSEGSVIAYYWSEFSIPQHLVEEAERVMAEERVVM
LPPRARSLKSFVVTSVVAFPTDSKTVQRTQDNSCSFGLHARGVELMRFTTPGFPDSPYPA
HARCQWALRGDADSVLSLTFRSFDLASCDERGSDLVTVYNTLSPMEPHALVQLCGTYPPS
YNLTFHSSQNVLLITLITNTERRHPGFEATFFQLPRMSSCGGRLRKAQGTFNSPYYPGHY
PPNIDCTWNIEVPNNQHVKVRFKFFYLLEPGVPAGTCPKDYVEINGEKYCGERSQFVVTS
NSNKITVRFHSDQSYTDTGFLAEYLSYDSSDPCPGQFTCRTGRCIRKELRCDGWADCTDH
SDELNCSCDAGHQFTCKNKFCKPLFWVCDSVNDCGDNSDEQGCSCPAQTFRCSNGKCLSK
SQQCNGKDDCGDGSDEASCPKVNVVTCTKHTYRCLNGLCLSKGNPECDGKEDCSDGSDEK
DCDCGLRSFTRQARVVGGTDADEGEWPWQVSLHALGQGHICGASLISPNWLVSAAHCYID
DRGFRYSDPTQWTAFLGLHDQSQRSAPGVQERRLKRIISHPFFNDFTFDYDIALLELEKP
AEYSSMVRPICLPDASHVFPAGKAIWVTGWGHTQYGGTGALILQKGEIRVINQTTCENLL
PQQITPRMMCVGFLSGGVDSCQGDSGGPLSSVEADGRIFQAGVVSWGDGCAQRNKPGVYT
RLPLFRDWIKENTGV
>hsa:6799
MELIQDISRPPLEYVKGVPLIKYFAEALGPLQSFQARPDDLLISTYPKSGTTWVSQILDM
IYQGGDLEKCHRAPIFMRVPFLEFKVPGIPSGMETLKNTPAPRLLKTHLPLALLPQTLLD
QKVKVVYVARNAKDVAVSYYHFYHMAKVYPHPGTWESFLEKFMAGEVSYGSWYQHVQEWW
ELSRTHPVLYLFYEDMKENPKREIQKILEFVGRSLPEETVDLMVEHTSFKEMKKNPMTNY
TTVRREFMDHSISPFMRKGMAGDWKTTFTVAQNERFDADYAEKMAGCSLSFRSEL
>hsa:683
MAAQGCAASRLLQLLLQLLLLLLLLAAGGARARWRGEGTSAHLRDIFLGRCAEYRALLSP
EQRNKNCTAIWEAFKVALDKDPCSVLPSDYDLFINLSRHSIPRDKSLFWENSHLLVNSFA
DNTRRFMPLSDVLYGRVADFLSWCRQKNDSGLDYQSCPTSEDCENNPVDSFWKRASIQYS
KDSSGVIHVMLNGSEPTGAYPIKGFFADYEIPNLQKEKITRIEIWVMHEIGGPNVESCGE

GSMKVLEKRLKDMGFQYSCINDYRPVKLLQCVDHSTHPDCALKSAAAATQRKAPSLYTEQ
RAGLIIPLFLVLASRTQL
>hsa:686
MAHAHIQGGRRAKSRFVVCIMSGARSKLALFLCGCYVVALGAHTGEESVADHHEAEYYVA
AVYEHPSILSLNPLALISRQEALELMNQNLDIYEQQVMTAAQKDVQIIVFPEDGIHGFNF
TRTSIYPFLDFMPSPQVVRWNPCLEPHRFNDTEVLQRLSCMAIRGDMFLVANLGTKEPCH
SSDPRCPKDGRYQFNTNVVFSNNGTLVDRYRKHNLYFEAAFDVPLKVDLITFDTPFAGRF
GIFTCFDILFFDPAIRVLRDYKVKHVVYPTAWMNQLPLLAAIEIQKAFAVAFGINVLAAN
VHHPVLGMTGSGIHTPLESFWYHDMENPKSHLIIAQVAKNPVGLIGAENATGETDPSHSK
FLKILSGDPYCEKDAQEVHCDEATKWNVNAPPTFHSEMMYDNFTLVPVWGKEGYLHVCSN
GLCCYLLYERPTLSKELYALGVFDGLHTVHGTYYIQVCALVRCGGLGFDTCGQEITEATG
IFEFHLWGNFSTSYIFPLFLTSGMTLEVPDQLGWENDHYFLRKSRLSSGLVTAALYGRLY
ERD
>hsa:6897
MFEEKASSPSGKMGGEEKPIGAGEEKQKEGGKKKNKEGSGDGGRAELNPWPEYIYTRLEM
YNILKAEHDSILAEKAEKDSKPIKVTLPDGKQVDAESWKTTPYQIACGISQGLADNTVIA
KVNNVVWDLDRPLEEDCTLELLKFEDEEAQAVYWHSSAHIMGEAMERVYGGCLCYGPPIE
NGFYYDMYLEEGGVSSNDFSSLEALCKKIIKEKQAFERLEVKKETLLAMFKYNKFKCRIL
NEKVNTPTTTVYRCGPLIDLCRGPHVRHTGKIKALKIHKNSSTYWEGKADMETLQRIYGI
SFPDPKMLKEWEKFQEEAKNRDHRKIGRDQELYFFHELSPGSCFFLPKGAYIYNALIEFI
RSEYRKRGFQEVVTPNIFNSRLWMTSGHWQHYSENMFSFEVEKELFALKPMNCPGHCLMF
DHRPRSWRELPLRLADFGVLHRNELSGALTGLTRVRRFQQDDAHIFCAMEQIEDEIKGCL
DFLRTVYSVFGFSFKLNLSTRPEKFLGDIEVWDQAEKQLENSLNEFGEKWELNSGDGAFY
GPKIDIQIKDAIGRYHQCATIQLDFQLPIRFNLTYVSHDGDDKKRPVIVHRAILGSVERM
IAILTENYGGKWPFWLSPRQVMVVPVGPTCDEYAQKVRQQFHDAKFMADIDLDPGCTLNK
KIRNAQLAQYNFILVVGEKEKISGTVNIRTRDNKVHGERTISETIERLQQLKEFRSKQAE
EEF
>hsa:6898
MDPYMIQMSSKGNLPSILDVHVNVGGRSSVPGKMKGRKARWSVRPSDMAKKTFNPIRAIV
DNMKVKPNPNKTMISLSIGDPTVFGNLPTDPEVTQAMKDALDSGKYNGYAPSIGFLSSRE
EIASYYHCPEAPLEAKDVILTSGCSQAIDLCLAVLANPGQNILVPRPGFSLYKTLAESMG
IEVKLYNLLPEKSWEIDLKQLEYLIDEKTACLIVNNPSNPCGSVFSKRHLQKILAVAARQ
CVPILADEIYGDMVFSDCKYEPLATLSTDVPILSCGGLAKRWLVPGWRLGWILIHDRRDI
FGNEIRDGLVKLSQRILGPCTIVQGALKSILCRTPGEFYHNTLSFLKSNADLCYGALAAI
PGLRPVRPSGAMYLMVGIEMEHFPEFENDVEFTERLVAEQSVHCLPATCFEYPNFIRVVI
TVPEVMMLEACSRIQEFCEQHYHCAEGSQEECDK
>hsa:695
MAAVILESIFLKRSQQKKKTSPLNFKKRLFLLTVHKLSYYEYDFERGRRGSKKGSIDVEK
ITCVETVVPEKNPPPERQIPRRGEESSEMEQISIIERFPYPFQVVYDEGPLYVFSPTEEL
RKRWIHQLKNVIRYNSDLVQKYHPCFWIDGQYLCCSQTAKNAMGCQILENRNGSLKPGSS
HRKTKKPLPPTPEEDQILKKPLPPEPAAAPVSTSELKKVVALYDYMPMNANDLQLRKGDE
YFILEESNLPWWRARDKNGQEGYIPSNYVTEAEDSIEMYEWYSKHMTRSQAEQLLKQEGK
EGGFIVRDSSKAGKYTVSVFAKSTGDPQGVIRHYVVCSTPQSQYYLAEKHLFSTIPELIN
YHQHNSAGLISRLKYPVSQQNKNAPSTAGLGYGSWEIDPKDLTFLKELGTGQFGVVKYGK

WRGQYDVAIKMIKEGSMSEDEFIEEAKVMMNLSHEKLVQLYGVCTKQRPIFIITEYMANG
CLLNYLREMRHRFQTQQLLEMCKDVCEAMEYLESKQFLHRDLAARNCLVNDQGVVKVSDF
GLSRYVLDDEYTSSVGSKFPVRWSPPEVLMYSKFSSKSDIWAFGVLMWEIYSLGKMPYER
FTNSETAEHIAQGLRLYRPHLASEKVYTIMYSCWHEKADERPTFKILLSNILDVMDEES
>hsa:7006
MNFNTILEEILIKRSQQKKKTSPLNYKERLFVLTKSMLTYYEGRAEKKYRKGFIDVSKIK
CVEIVKNDDGVIPCQNKYPFQVVHDANTLYIFAPSPQSRDLWVKKLKEEIKNNNNIMIKY
HPKFWTDGSYQCCRQTEKLAPGCEKYNLFESSIRKALPPAPETKKRRPPPPIPLEEEDNS
EEIVVAMYDFQAAEGHDLRLERGQEYLILEKNDVHWWRARDKYGNEGYIPSNYVTGKKSN
NLDQYEWYCRNMNRSKAEQLLRSEDKEGGFMVRDSSQPGLYTVSLYTKFGGEGSSGFRHY
HIKETTTSPKKYYLAEKHAFGSIPEIIEYHKHNAAGLVTRLRYPVSVKGKNAPTTAGFSY
EKWEINPSELTFMRELGSGLFGVVRLGKWRAQYKVAIKAIREGAMCEEDFIEEAKVMMKL
THPKLVQLYGVCTQQKPIYIVTEFMERGCLLNFLRQRQGHFSRDVLLSMCQDVCEGMEYL
ERNSFIHRDLAARNCLVSEAGVVKVSDFGMARYVLDDQYTSSSGAKFPVKWCPPEVFNYS
RFSSKSDVWSFGVLMWEVFTEGRMPFEKYTNYEVVTMVTRGHRLYQPKLASNYVYEVMLR
CWQEKPEGRPSFEDLLRTIDELVECEETFGR
>hsa:7010
MDSLASLVLCGVSLLLSGTVEGAMDLILINSLPLVSDAETSLTCIASGWRPHEPITIGRD
FEALMNQHQDPLEVTQDVTREWAKKVVWKREKASKINGAYFCEGRVRGEAIRIRTMKMRQ
QASFLPATLTMTVDKGDNVNISFKKVLIKEEDAVIYKNGSFIHSVPRHEVPDILEVHLPH
AQPQDAGVYSARYIGGNLFTSAFTRLIVRRCEAQKWGPECNHLCTACMNNGVCHEDTGEC
ICPPGFMGRTCEKACELHTFGRTCKERCSGQEGCKSYVFCLPDPYGCSCATGWKGLQCNE
ACHPGFYGPDCKLRCSCNNGEMCDRFQGCLCSPGWQGLQCEREGIPRMTPKIVDLPDHIE
VNSGKFNPICKASGWPLPTNEEMTLVKPDGTVLHPKDFNHTDHFSVAIFTIHRILPPDSG
VWVCSVNTVAGMVEKPFNISVKVLPKPLNAPNVIDTGHNFAVINISSEPYFGDGPIKSKK
LLYKPVNHYEAWQHIQVTNEIVTLNYLEPRTEYELCVQLVRRGEGGEGHPGPVRRFTTAS
IGLPPPRGLNLLPKSQTTLNLTWQPIFPSSEDDFYVEVERRSVQKSDQQNIKVPGNLTSV
LLNNLHPREQYVVRARVNTKAQGEWSEDLTAWTLSDILPPQPENIKISNITHSSAVISWT
ILDGYSISSITIRYKVQGKNEDQHVDVKIKNATITQYQLKGLEPETAYQVDIFAENNIGS
SNPAFSHELVTLPESQAPADLGGGKMLLIAILGSAGMTCLTVLLAFLIILQLKRANVQRR
MAQAFQNVREEPAVQFNSGTLALNRKVKNNPDPTIYPVLDWNDIKFQDVIGEGNFGQVLK
ARIKKDGLRMDAAIKRMKEYASKDDHRDFAGELEVLCKLGHHPNIINLLGACEHRGYLYL
AIEYAPHGNLLDFLRKSRVLETDPAFAIANSTASTLSSQQLLHFAADVARGMDYLSQKQF
IHRDLAARNILVGENYVAKIADFGLSRGQEVYVKKTMGRLPVRWMAIESLNYSVYTTNSD
VWSYGVLLWEIVSLGGTPYCGMTCAELYEKLPQGYRLEKPLNCDDEVYDLMRQCWREKPY
ERPSFAQILVSLNRMLEERKTYVNTTLYEKFTYAGIDCSAEEAA
>hsa:7015
MPRAPRCRAVRSLLRSHYREVLPLATFVRRLGPQGWRLVQRGDPAAFRALVAQCLVCVPW
DARPPPAAPSFRQVSCLKELVARVLQRLCERGAKNVLAFGFALLDGARGGPPEAFTTSVR
SYLPNTVTDALRGSGAWGLLLRRVGDDVLVHLLARCALFVLVAPSCAYQVCGPPLYQLGA
ATQARPPPHASGPRRRLGCERAWNHSVREAGVPLGLPAPGARRRGGSASRSLPLPKRPRR
GAAPEPERTPVGQGSWAHPGRTRGPSDRGFCVVSPARPAEEATSLEGALSGTRHSHPSVG
RQHHAGPPSTSRPPRPWDTPCPPVYAETKHFLYSSGDKEQLRPSFLLSSLRPSLTGARRL
VETIFLGSRPWMPGTPRRLPRLPQRYWQMRPLFLELLGNHAQCPYGVLLKTHCPLRAAVT

PAAGVCAREKPQGSVAAPEEEDTDPRRLVQLLRQHSSPWQVYGFVRACLRRLVPPGLWGS
RHNERRFLRNTKKFISLGKHAKLSLQELTWKMSVRDCAWLRRSPGVGCVPAAEHRLREEI
LAKFLHWLMSVYVVELLRSFFYVTETTFQKNRLFFYRKSVWSKLQSIGIRQHLKRVQLRE
LSEAEVRQHREARPALLTSRLRFIPKPDGLRPIVNMDYVVGARTFRREKRAERLTSRVKA
LFSVLNYERARRPGLLGASVLGLDDIHRAWRTFVLRVRAQDPPPELYFVKVDVTGAYDTI
PQDRLTEVIASIIKPQNTYCVRRYAVVQKAAHGHVRKAFKSHVSTLTDLQPYMRQFVAHL
QETSPLRDAVVIEQSSSLNEASSGLFDVFLRFMCHHAVRIRGKSYVQCQGIPQGSILSTL
LCSLCYGDMENKLFAGIRRDGLLLRLVDDFLLVTPHLTHAKTFLSYARTSIRASLTFNRG
FKAGRNMRRKLFGVLRLKCHSLFLDLQVNSLQTVCTNIYKILLLQAYRFHACVLQLPFHQ
QVWKNPTFFLRVISDTASLCYSILKAKNAGMSLGAKGAAGPLPSEAVQWLCHQAFLLKLT
RHRVTYVPLLGSLRTAQTQLSRKLPGTTLTALEAAANPALPSDFKTILD
>hsa:7046
MEAAVAAPRPRLLLLVLAAAAAAAAALLPGATALQCFCHLCTKDNFTCVTDGLCFVSVTE
TTDKVIHNSMCIAEIDLIPRDRPFVCAPSSKTGSVTTTYCCNQDHCNKIELPTTGLPLLV
QRTIARTIVLQESIGKGRFGEVWRGKWRGEEVAVKIFSSREERSWFREAEIYQTVMLRHE
NILGFIAADNKDNGTWTQLWLVSDYHEHGSLFDYLNRYTVTVEGMIKLALSTASGLAHLH
MEIVGTQGKPAIAHRDLKSKNILVKKNGTCCIADLGLAVRHDSATDTIDIAPNHRVGTKR
YMAPEVLDDSINMKHFESFKRADIYAMGLVFWEIARRCSIGGIHEDYQLPYYDLVPSDPS
VEEMRKVVCEQKLRPNIPNRWQSCEALRVMAKIMRECWYANGAARLTALRIKKTLSQLSQ
QEGIKM
>hsa:7054
MPTPDATTPQAKGFRRAVSELDAKQAEAIMSPRFIGRRQSLIEDARKEREAAVAAAAAAV
PSEPGDPLEAVAFEEKEGKAVLNLLFSPRATKPSALSRAVKVFETFEAKIHHLETRPAQR
PRAGGPHLEYFVRLEVRRGDLAALLSGVRQVSEDVRSPAGPKVPWFPRKVSELDKCHHLV
TKFDPDLDLDHPGFSDQVYRQRRKLIAEIAFQYRHGDPIPRVEYTAEEIATWKEVYTTLK
GLYATHACGEHLEAFALLERFSGYREDNIPQLEDVSRFLKERTGFQLRPVAGLLSARDFL
ASLAFRVFQCTQYIRHASSPMHSPEPDCCHELLGHVPMLADRTFAQFSQDIGLASLGASD
EEIEKLSTLYWFTVEFGLCKQNGEVKAYGAGLLSSYGELLHCLSEEPEIRAFDPEAAAVQ
PYQDQTYQSVYFVSESFSDAKDKLRSYASRIQRPFSVKFDPYTLAIDVLDSPQAVRRSLE
GVQDELDTLAHALSAIG
>hsa:7075
MWAGAGRGSDAWGPPLLLEKDDRIVRTPPGPPLRLARNGSHQVTLRGFSKPSDLVGVFSC
VGGAGARRTRVIYVHNSPGAHLLPDKVTHTVNKGDTAVLSARVHKEKQTDVIWKSNGSYF
YTLDWHEAQDGRFLLQLPNVQPPSSGIYSATYLEASPLGSAFFRLIVRGCGAGRWGPGCT
KECPGCLHGGVCHDHDGECVCPPGFTGTRCEQACREGRFGQSCQEQCPGISGCRGLTFCL
PDPYGCSCGSGWRGSQCQEACAPGHFGADCRLQCQCQNGGTCDRFSGCVCPSGWHGVHCE
KSDRIPQILNMASELEFNLETMPRINCAAAGNPFPVRGSIELRKPDGTVLLSTKAIVEPE
KTTAEFEVPRLVLADSGFWECRVSTSGGQDSRRFKVNVKVPPVPLAAPRLLTKQSRQLVV
SPLVSFSGDGPISTVRLHYRPQDSTMDWSTIVVDPSENVTLMNLRPKTGYSVRVQLSRPG
EGGEGAWGPPTLMTTDCPEPLLQPWLEGWHVEGTDRLRVSWSLPLVPGPLVGDGFLLRLW
DGTRGQERRENVSSPQARTALLTGLTPGTHYQLDVQLYHCTLLGPASPPAHVLLPPSGPP
APRHLHAQALSDSEIQLTWKHPEALPGPISKYVVEVQVAGGAGDPLWIDVDRPEETSTII
RGLNASTRYLFRMRASIQGLGDWSNTVEESTLGNGLQAEGPVQESRAAEEGLDQQLILAV
VGSVSATCLTILAALLTLVCIRRSCLHRRRTFTYQSGSGEETILQFSSGTLTLTRRPKLQ

PEPLSYPVLEWEDITFEDLIGEGNFGQVIRAMIKKDGLKMNAAIKMLKEYASENDHRDFA
GELEVLCKLGHHPNIINLLGACKNRGYLYIAIEYAPYGNLLDFLRKSRVLETDPAFAREH
GTASTLSSRQLLRFASDAANGMQYLSEKQFIHRDLAARNVLVGENLASKIADFGLSRGEE
VYVKKTMGRLPVRWMAIESLNYSVYTTKSDVWSFGVLLWEIVSLGGTPYCGMTCAELYEK
LPQGYRMEQPRNCDDEVYELMRQCWRDRPYERPPFAQIALQLGRMLEARKAYVNMSLFEN
FTYAGIDATAEEA
>hsa:7083
MSCINLPTVLPGSPSKTRGQIQVILGPMFSGKSTELMRRVRRFQIAQYKCLVIKYAKDTR
YSSSFCTHDRNTMEALPACLLRDVAQEALGVAVIGIDEGQFFPDIVEFCEAMANAGKTVI
VAALDGTFQRKPFGAILNLVPLAESVVKLTAVCMECFREAAYTKRLGTEKEVEVIGGADK
YHSVCRLCYFKKASGQPAGPDNKENCPVPGKPGEAVAARKLFAPQQILQCSPAN
>hsa:7084
MGAFCQRPSSDKEQEKEKKSVICVEGNIASGKTTCLEFFSNATDVEVLTEPVSKWRNVRG
HNPLGLMYHDASRWGLTLQTYVQLTMLDRHTRPQVSSVRLMERSIHSARYIFVENLYRSG
KMPEVDYVVLSEWFDWILRNMDVSVDLIVYLRTNPETCYQRLKKRCREEEKVIPLEYLEA
IHHLHEEWLIKGSLFPMAAPVLVIEADHHMERMLELFEQNRDRILTPENRKHCP
>hsa:7150
MSGDHLHNDSQIEADFRLNDSHKHKDKHKDREHRHKEHKKEKDREKSKHSNSEHKDSEKK
HKEKEKTKHKDGSSEKHKDKHKDRDKEKRKEEKVRASGDAKIKKEKENGFSSPPQIKDEP
EDDGYFVPPKEDIKPLKRPRDEDDADYKPKKIKTEDTKKEKKRKLEEEEDGKLKKPKNKD
KDKKVPEPDNKKKKPKKEEEQKWKWWEEERYPEGIKWKFLEHKGPVFAPPYEPLPENVKF
YYDGKVMKLSPKAEEVATFFAKMLDHEYTTKEIFRKNFFKDWRKEMTNEEKNIITNLSKC
DFTQMSQYFKAQTEARKQMSKEEKLKIKEENEKLLKEYGFCIMDNHKERIANFKIEPPGL
FRGRGNHPKMGMLKRRIMPEDIIINCSKDAKVPSPPPGHKWKEVRHDNKVTWLVSWTENI
QGSIKYIMLNPSSRIKGEKDWQKYETARRLKKCVDKIRNQYREDWKSKEMKVRQRAVALY
FIDKLALRAGNEKEEGETADTVGCCSLRVEHINLHPELDGQEYVVEFDFLGKDSIRYYNK
VPVEKRVFKNLQLFMENKQPEDDLFDRLNTGILNKHLQDLMEGLTAKVFRTYNASITLQQ
QLKELTAPDENIPAKILSYNRANRAVAILCNHQRAPPKTFEKSMMNLQTKIDAKKEQLAD
ARRDLKSAKADAKVMKDAKTKKVVESKKKAVQRLEEQLMKLEVQATDREENKQIALGTSK
LNYLDPRITVAWCKKWGVPIEKIYNKTQREKFAWAIDMADEDYEF
>hsa:7153
MEVSPLQPVNENMQVNKIKKNEDAKKRLSVERIYQKKTQLEHILLRPDTYIGSVELVTQQ
MWVYDEDVGINYREVTFVPGLYKIFDEILVNAADNKQRDPKMSCIRVTIDPENNLISIWN
NGKGIPVVEHKVEKMYVPALIFGQLLTSSNYDDDEKKVTGGRNGYGAKLCNIFSTKFTVE
TASREYKKMFKQTWMDNMGRAGEMELKPFNGEDYTCITFQPDLSKFKMQSLDKDIVALMV
RRAYDIAGSTKDVKVFLNGNKLPVKGFRSYVDMYLKDKLDETGNSLKVIHEQVNHRWEVC
LTMSEKGFQQISFVNSIATSKGGRHVDYVADQIVTKLVDVVKKKNKGGVAVKAHQVKNHM
WIFVNALIENPTFDSQTKENMTLQPKSFGSTCQLSEKFIKAAIGCGIVESILNWVKFKAQ
VQLNKKCSAVKHNRIKGIPKLDDANDAGGRNSTECTLILTEGDSAKTLAVSGLGVVGRDK
YGVFPLRGKILNVREASHKQIMENAEINNIIKIVGLQYKKNYEDEDSLKTLRYGKIMIMT
DQDQDGSHIKGLLINFIHHNWPSLLRHRFLEEFITPIVKVSKNKQEMAFYSLPEFEEWKS
STPNHKKWKVKYYKGLGTSTSKEAKEYFADMKRHRIQFKYSGPEDDAAISLAFSKKQIDD
RKEWLTNFMEDRRQRKLLGLPEDYLYGQTTTYLTYNDFINKELILFSNSDNERSIPSMVD
GLKPGQRKVLFTCFKRNDKREVKVAQLAGSVAEMSSYHHGEMSLMMTIINLAQNFVGSNN

LNLLQPIGQFGTRLHGGKDSASPRYIFTMLSSLARLLFPPKDDHTLKFLYDDNQRVEPEW
YIPIIPMVLINGAEGIGTGWSCKIPNFDVREIVNNIRRLMDGEEPLPMLPSYKNFKGTIE
ELAPNQYVISGEVAILNSTTIEISELPVRTWTQTYKEQVLEPMLNGTEKTPPLITDYREY
HTDTTVKFVVKMTEEKLAEAERVGLHKVFKLQTSLTCNSMVLFDHVGCLKKYDTVLDILR
DFFELRLKYYGLRKEWLLGMLGAESAKLNNQARFILEKIDGKIIIENKPKKELIKVLIQR
GYDSDPVKAWKEAQQKVPDEEENEESDNEKETEKSDSVTDSGPTFNYLLDMPLWYLTKEK
KDELCRLRNEKEQELDTLKRKSPSDLWKEDLATFIEELEAVEAKEKQDEQVGLPGKGGKA
KGKKTQMAEVLPSPRGQRVIPRITIEMKAEAEKKNKKKIKNENTEGSPQEDGVELEGLKQ
RLEKKQKREPGTKTKKQTTLAFKPIKKGKKRNPWSDSESDRSSDESNFDVPPRETEPRRA
ATKTKFTMDLDSDEDFSDFDEKTDDEDFVPSDASPPKTKTSPKLSNKELKPQKSVVSDLE
ADDVKGSVPLSSSPPATHFPDETEITNPVPKKNVTVKKTAAKSQSSTSTTGAKKRAAPKG
TKRDPALNSGVSQKPDPAKTKNRRKRKPSTSDDSDSNFEKIVSKAVTSKKSKGESDDFHM
DFDSAVAPRAKSVRAKKPIKYLEESDEDDLF

>hsa:7155

MAKSGGCGAGAGVGGGNGALTWVNNAAKKEESETANKNDSSKKLSVERVYQKKTQLEHIL
LRPDTYIGSVEPLTQFMWVYDEDVGMNCREVTFVPGLYKIFDEILVNAADNKQRDKNMTC
IKVSIDPESNIISIWNNGKGIPVVEHKVEKVYVPALIFGQLLTSSNYDDDEKKVTGGRNG
YGAKLCNIFSTKFTVETACKEYKHSFKQTWMNNMMKTSEAKIKHFDGEDYTCITFQPDLS
KFKMEKLDKDIVALMTRRAYDLAGSCRGVKVMFNGKKLPVNGFRSYVDLYVKDKLDETGV
ALKVIHELANERWDVCLTLSEKGFQQISFVNSIATTKGGRHVDYVVDQVVGKLIEVVKKK
NKAGVSVKPFQVKNHIWVFINCLIENPTFDSQTKENMTLQPKSFGSKCQLSEKFFKAASN
CGIVESILNWVKFKAQTQLNKKCSSVKYSKIKGIPKLDDANDAGGKHSLECTLILTEGDS
AKSLAVSGLGVIGRDRYGVFPLRGKILNVREASHKQIMENAEINNIIKIVGLQYKKSYDD
AESLKTLRYGKIMIMTDQDQDGSHIKGLLINFIHHNWPSLLKHGFLEEFITPIVKASKNK
QELSFYSIPEFDEWKKHIENQKAWKIKYYKGLGTSTAKEAKEYFADMERHRILFRYAGPE
DDAAITLAFSKKKIDDRKEWLTNFMEDRRQRRLHGLPEQFLYGTATKHLTYNDFINKELI
LFSNSDNERSIPSLVDGFKPGQRKVLFTCFKRNDKREVKVAQLAGSVAEMSAYHHGEQAL
MMTIVNLAQNFVGSNNINLLQPIGQFGTRLHGGKDAASPRYIFTMLSTLARLLFPAVDDN
LLKFLYDDNQRVEPEWYIPIIPMVLINGAEGIGTGWACKLPNYDAREIVNNVRRMLDGLD
PHPMLPNYKNFKGTIQELGQNQYAVSGEIFVVDRNTVEITELPVRTWTQVYKEQVLEPML
NGTDKTPALISDYKEYHTDTTVKFVVKMTEEKLAQAEAAGLHKVFKLQTTLTCNSMVLFD
HMGCLKKYETVQDILKEFFDLRLSYYGLRKEWLVGMLGAESTKLNNQARFILEKIQGKIT
IENRSKKDLIQMLVQRGYESDPVKAWKEAQEKAAEEDETQNQHDDSSSDSGTPSGPDFNY
ILNMSLWSLTKEKVEELIKQRDAKGREVNDLKRKSPSDLWKEDLAAFVEELDKVESQERE
DVLAGMSGKAIKGKVGKPKVKKLQLEETMPSPYGRRIIPEITAMKADASKKLLKKKKGDL
DTAAVKVEFDEEFSGAPVEGAGEEALTPSVPINKGPKPKREKKEPGTRVRKTPTSSGKPS
AKKVKKRNPWSDDESKSESDLEETEPVVIPRDSLLRRAAAERPKYTFDFSEEEDDDADDD
DDDNNDLEELKVKASPITNDGEDEFVPSDGLDKDEYTFSPGKSKATPEKSLHDKKSQDFG
NLFSFPSYSQKSEDDSAKFDSNEEDSASVFSPSFGLKQTDKVPSKTVAAKKGKPSSDTVP
KPKRAPKQKKVVEAVNSDSDSEFGIPKKTTTPKGKGRGAKKRKASGSENEGDYNPGRKTS
KTTSKKPKKTSFDQDSDVDIFPSDFPTEPPSLPRTGRARKEVKYFAESDEEEDDVDFAMF
N

>hsa:7156

MIFPVARYALRWLRRPEDRAFSRAAMEMALRGVRKVLCVAEKNDAAKGIADLLSNGRMRR

REGLSKFNKIYEFDYHLYGQNVTMVMTSVSGHLLAHDFQMQFRKWQSCNPLVLFEAEIEK
YCPENFVDIKKTLERETRQCQALVIWTDCDREGENIGFEIIHVCKAVKPNLQVLRARFSE
ITPHAVRTACENLTEPDQRVSDAVDVRQELDLRIGAAFTRFQTLRLQRIFPEVLAEQLIS
YGSCQFPTLGFVVERFKAIQAFVPEIFHRIKVTHDHKDGIVEFNWKRHRLFNHTACLVLY
QLCVEDPMATVVEVRSKPKSKWRPQALDTVELEKLASRKLRINAKETMRIAEKLYTQGYI
SYPRTETNIFPRDLNLTVLVEQQTPDPRWGAFAQSILERGGPTPRNGNKSDQAHPPIHPT
KYTNNLQGDEQRLYEFIVRHFLACCSQDAQGQETTVEIDIAQERFVAHGLMILARNYLDV
YPYDHWSDKILPVYEQGSHFQPSTVEMVDGETSPPKLLTEADLIALMEKHGIGTDATHAE
HIETIKARMYVGLTPDKRFLPGHLGMGLVEGYDSMGYEMSKPDLRAELEADLKLICDGKK
DKFVVLRQQVQKYKQVFIEAVAKAKKLDEALAQYFGNGTELAQQEDIYPAMPEPIRKCPQ
CNKDMVLKTKKNGGFYLSCMGFPECRSAVWLPDSVLEASRDSSVCPVCQPHPVYRLKLKF
KRGSLPPTMPLEFVCCIGGCDDTLREILDLRFSGGPPRASQPSGRLQANQSLNRMDNSQH
PQPADSRQTGSSKALAQTLPPPTAAGESNSVTCNCGQEAVLLTVRKEGPNRGRQFFKCNG
GSCNFFLWADSPNPGAGGPPALAYRPLGASLGCPPGPGIHLGGFGNPGDGSGSGTSCLCS
QPSVTRTVQKDGPNKGRQFHTCAKPREQQCGFFQWVDENTAPGTSGAPSWTGDRGRTLES
EARSKRPRASSSDMGSTAKKPRKCSLCHQPGHTRPFCPQNR
>hsa:7172
MDGTRTSLDIEEYSDTEVQKNQVLTLEEWQDKWVNGKTAFHQEQGHQLLKKHLDTFLKGK
SGLRVFFPLCGKAVEMKWFADRGHSVVGVEISELGIQEFFTEQNLSYSEEPITEIPGTKV
FKSSSGNISLYCCSIFDLPRTNIGKFDMIWDRGALVAINPGDRKCYADTMFSLLGKKFQY
LLCVLSYDPTKHPGPPFYVPHAEIERLFGKICNIRCLEKVDAFEERHKSWGIDCLFEKLY
LLTEK
>hsa:7173
MRALAVLSVTLVMACTEAFFPFISRGKELLWGKPEESRVSSVLEESKRLVDTAMYATMQR
NLKKRGILSPAQLLSFSKLPEPTSGVIARAAEIMETSIQAMKRKVNLKTQQSQHPTDALS
EDLLSIIANMSGCLPYMLPPKCPNTCLANKYRPITGACNNRDHPRWGASNTALARWLPPV
YEDGFSQPRGWNPGFLYNGFPLPPVREVTRHVIQVSNEVVTDDDRYSDLLMAWGQYIDHD
IAFTPQSTSKAAFGGGADCQMTCENQNPCFPIQLPEEARPAAGTACLPFYRSSAACGTGD
QGALFGNLSTANPRQQMNGLTSFLDASTVYGSSPALERQLRNWTSAEGLLRVHARLRDSG
RAYLPFVPPRAPAACAPEPGIPGETRGPCFLAGDGRASEVPSLTALHTLWLREHNRLAAA
LKALNAHWSADAVYQEARKVVGALHQIITLRDYIPRILGPEAFQQYVGPYEGYDSTANPT
VSNVFSTAAFRFGHATIHPLVRRLDASFQEHPDLPGLWLHQAFFSPWTLLRGGGLDPLIR
GLLARPAKLQVQDQLMNEELTERLFVLSNSSTLDLASINLQRGRDHGLPGYNEWREFCGL
PRLETPADLSTAIASRSVADKILDLYKHPDNIDVWLGGLAENFLPRARTGPLFACLIGKQ
MKALRDGDWFWWENSHVFTDAQRRELEKHSLSRVICDNTGLTRVPMDAFQVGKFPEDFES
CDSITGMNLEAWRETFPQDDKCGFPESVENGDFVHCEESGRRVLVYSCRHGYELQGREQL
TCTQEGWDFQPPLCKDVNECADGAHPPCHASARCRNTKGGFQCLCADPYELGDDGRTCVD
SGRLPRVTWISMSLAALLIGGFAGLTSTVICRWTRTGTKSTLPISETGGGTPELRCGKHQ
AVGTSPQRAAAQDSEQESAGMEGRDTHRLPRAL
>hsa:7174
MATAATEEPFPFHGLLPKKETGAASFLCRYPEYDGRGVLIAVLDTGVDPGAPGMQVTTDG
KPKIVDIIDTTGSGDVNTATEVEPKDGEIVGLSGRVLKIPASWTNPSGKYHIGIKNGYDF
YPKALKERIQKERKEKIWDPVHRVALAEACRKQEEFDVANNGSSQANKLIKEELQSQVEL
LNSFEKKYSDPGPVYDCLVWHDGEVWRACIDSNEDGDLSKSTVLRNYKEAQEYGSFGTAE

MLNYSVNIYDDGNLLSIVTSGGAHGTHVASIAAGHFPEEPERNGVAPGAQILSIKIGDTR
LSTMETGTGLIRAMIEVINHKCDLVNYSYGEATHWPNSGRICEVINEAVWKHNIIYVSSA
GNNGPCLSTVGCPGGTTSSVIGVGAYVSPDMMVAEYSLREKLPANQYTWSSRGPSADGAL
GVSISAPGGAIASVPNWTLRGTQLMNGTSMSSPNACGGIALILSGLKANNIDYTVHSVRR
ALENTAVKADNIEVFAQGHGIIQVDKAYDYLVQNTSFANKLGFTVTVGNNRGIYLRDPVQ
VAAPSDHGVGIEPVFPENTENSEKISLQLHLALTSNSSWVQCPSHLELMNQCRHINIRVD
PRGLREGLHYTEVCGYDIASPNAGPLFRVPITAVIAAKVNESSHYDLAFTDVHFKPGQIR
RHFIEVPEGATWAEVTVCSCSSEVSAKFVLHAVQLVKQRAYRSHEFYKFCSLPEKGTLTE
AFPVLGGKAIEFCIARWWASLSDVNIDYTISFHGIVCTAPQLNIHASEGINRFDVQSSLK
YEDLAPCITLKNWVQTLRPVSAKTKPLGSRDVLPNNRQLYEMVLTYNFHQPKSGEVTPSC
PLLCELLYESEFDSQLWIIFDQNKRQMGSGDAYPHQYSLKLEKGDYTIRLQIRHEQISDL
ERLKDLPFIVSHRLSNTLSLDIHENHSFALLGKKKSSNLTLPPKYNQPFFVTSLPDDKIP
KGAGPGCYLAGSLTLSKTELGKKADVIPVHYYLIPPPTKTKNGSKDKEKDSEKEKDLKEE
FTEALRDLKIQWMTKLDSSDIYNELKETYPNYLPLYVARLHQLDAEKERMKRLNEIVDAA
NAVISHIDQTALAVYIAMKTDPRPDAATIKNDMDKQKSTLVDALCRKGCALADHLLHTQA
QDGAISTDAEGKEEEGESPLDSLAETFWETTKWTDLFDNKVLTFAYKHALVNKMYGRGLK
FATKLVEEKPTKENWKNCIQLMKLLGWTHCASFTENWLPIMYPPDYCVF
>hsa:7294
MILSSYNTIQSVFCCCCCCSVQKRQMRTQISLSTDEELPEKYTQRRRPWLSQLSNKKQSN
TGRVQPSKRKPLPPLPPSEVAEEKIQVKALYDFLPREPCNLALRRAEEYLILEKYNPHWW
KARDRLGNEGLIPSNYVTENKITNLEIYEWYHRNITRNQAEHLLRQESKEGAFIVRDSRH
LGSYTISVFMGARRSTEAAIKHYQIKKNDSGQWYVAERHAFQSIPELIWYHQHNAAGLMT
RLRYPVGLMGSCLPATAGFSYEKWEIDPSELAFIKEIGSGQFGVVHLGEWRSHIQVAIKA
INEGSMSEEDFIEEAKVMMKLSHSKLVQLYGVCIQRKPLYIVTEFMENGCLLNYLRENKG
KLRKEMLLSVCQDICEGMEYLERNGYIHRDLAARNCLVSSTCIVKISDFGMTRYVLDDEY
VSSFGAKFPIKWSPPEVFLFNKYSSKSDVWSFGVLMWEVFTEGKMPFENKSNLQVVEAIS
EGFRLYRPHLAPMSIYEVMYSCWHEKPEGRPTFAELLRAVTEIAETW
>hsa:7297
MPLRHWGMARGSKPVGDGAQPMAAMGGLKVLLHWAGPGGGEPWVTFSESSLTAEEVCIHI
AHKVGITPPCFNLFALFDAQAQVWLPPNHILEIPRDASLMLYFRIRFYFRNWHGMNPREP
AVYRCGPPGTEASSDQTAQGMQLLDPASFEYLFEQGKHEFVNDVASLWELSTEEEIHHFK
NESLGMAFLHLCHLALRHGIPLEEVAKKTSFKDCIPRSFRRHIRQHSALTRLRLRNVFRR
FLRDFQPGRLSQQMVMVKYLATLERLAPRFGTERVPVCHLRLLAQAEGEPCYIRDSGVAP
TDPGPESAAGPPTHEVLVTGTGGIQWWPVEEEVNKEEGSSGSSGRNPQASLFGKKAKAHK
AVGQPADRPREPLWAYFCDFRDITHVVLKEHCVSIHRQDNKCLELSLPSRAAALSFVSLV
DGYFRLTADSSHYLCHEVAPPRLVMSIRDGIHGPLLEPFVQAKLRPEDGLYLIHWSTSHP
YRLILTVAQRSQAPDGMQSLRLRKFPIEQQDGAFVLEGWGRSFPSVRELGAALQGCLLRA
GDDCFSLRRCCLPQPGETSNLIIMRGARASPRTLNLSQLSFHRVDQKEITQLSHLGQGTR
TNVYEGRLRVEGSGDPEEGKMDDEDPLVPGRDRGQELRVVLKVLDPSHHDIALAFYETAS
LMSQVSHTHLAFVHGVCVRGPENIMVTEYVEHGPLDVWLRRERGHVPMAWKMVVAQQLAS
ALSYLENKNLVHGNVCGRNILLARLGLAEGTSPFIKLSDPGVGLGALSREERVERIPWLA
PECLPGGANSLSTAMDKWGFGATLLEICFDGEAPLQSRSPSEKEHFYQRQHRLPEPSCPQ
LATLTSQCLTYEPTQRPSFRTILRDLTRLQPHNLADVLTVNPDSPASDPTVFHKRYLKKI
RDLGEGHFGKVSLYCYDPTNDGTGEMVAVKALKADCGPQHRSGWKQEIDILRTLYHEHII

KYKGCCEDQGEKSLQLVMEYVPLGSLRDYLPRHSIGLAQLLLFAQQICEGMAYLHAQHYI
HRDLAARNVLLDNDRLVKIGDFGLAKAVPEGHEYYRVREDGDSPVFWYAPECLKEYKFYY
ASDVWSFGVTLYELLTHCDSSQSPPTKFLELIGIAQGQMTVLRLTELLERGERLPRPDKC
PCEVYHLMKNCWETEASFRPTFENLIPILKTVHEKYQGQAPSVFSVC
>hsa:7298
MPVAGSELPRRPLPPAAQERDAEPRPPHGELQYLGQIQHILRCGVRKDDRTGTGTLSVFG
MQARYSLRDEFPLLTTKRVFWKGVLEELLWFIKGSTNAKELSSKGVKIWDANGSRDFLDS
LGFSTREEGDLGPVYGFQWRHFGAEYRDMESDYSGQGVDQLQRVIDTIKTNPDDRRIIMC
AWNPRDLPLMALPPCHALCQFYVVNSELSCQLYQRSGDMGLGVPFNIASYALLTYMIAHI
TGLKPGDFIHTLGDAHIYLNHIEPLKIQLQREPRPFPKLRILRKVEKIDDFKAEDFQIEG
YNPHPTIKMEMAV
>hsa:7299
MLLAVLYCLLWSFQTSAGHFPRACVSSKNLMEKECCPPWSGDRSPCGQLSGRGSCQNILL
SNAPLGPQFPFTGVDDRESWPSVFYNRTCQCSGNFMGFNCGNCKFGFWGPNCTERRLLVR
RNIFDLSAPEKDKFFAYLTLAKHTISSDYVIPIGTYGQMKNGSTPMFNDINIYDLFVWMH
YYVSMDALLGGSEIWRDIDFAHEAPAFLPWHRLFLLRWEQEIQKLTGDENFTIPYWDWRD
AEKCDICTDEYMGGQHPTNPNLLSPASFFSSWQIVCSRLEEYNSHQSLCNGTPEGPLRRN
PGNHDKSRTPRLPSSADVEFCLSLTQYESGSMDKAANFSFRNTLEGFASPLTGIADASQS
SMHNALHIYMNGTMSQVQGSANDPIFLLHHAFVDSIFEQWLRRHRPLQEVYPEANAPIGH
NRESYMVPFIPLYRNGDFFISSKDLGYDYSYLQDSDPDSFQDYIKSYLEQASRIWSWLLG
AAMVGAVLTALLAGLVSLLCRHKRKQLPEEKQPLLMEKEDYHSLYQSHL
>hsa:7301
MALRRSMGRPGLPPLPLPPPPRLGLLLAALASLLLPESAAAGLKLMGAPVKLTVSQGQPV
KLNCSVEGMEEPDIQWVKDGAVVQNLDQLYIPVSEQHWIGFLSLKSVERSDAGRYWCQVE
DGGETEISQPVWLTVEGVPFFTVEPKDLAVPPNAPFQLSCEAVGPPEPVTIVWWRGTTKI
GGPAPSPSVLNVTGVTQSTMFSCEAHNLKGLASSRTATVHLQALPAAPFNITVTKLSSSN
ASVAWMPGADGRALLQSCTVQVTQAPGGWEVLAVVVPVPPFTCLLRDLVPATNYSLRVRC
ANALGPSPYADWVPFQTKGLAPASAPQNLHAIRTDSGLILEWEEVIPEAPLEGPLGPYKL
SWVQDNGTQDELTVEGTRANLTGWDPQKDLIVRVCVSNAVGCGPWSQPLVVSSHDRAGQQ
GPPHSRTSWVPVVLGVLTALVTAAALALILLRKRRKETRFGQAFDSVMARGEPAVHFRAA
RSFNRERPERIEATLDSLGISDELKEKLEDVLIPEQQFTLGRMLGKGEFGSVREAQLKQE
DGSFVKVAVKMLKADIIASSDIEEFLREAACMKEFDHPHVAKLVGVSLRSRAKGRLPIPM
VILPFMKHGDLHAFLLASRIGENPFNLPLQTLIRFMVDIACGMEYLSSRNFIHRDLAARN
CMLAEDMTVCVADFGLSRKIYSGDYYRQGCASKLPVKWLALESLADNLYTVQSDVWAFGV
TMWEIMTRGQTPYAGIENAEIYNYLIGGNRLKQPPECMEDVYDLMYQCWSADPKQRPSFT
CLRMELENILGQLSVLSASQDPLYINIERAEEPTAGGSLELPGRDQPYSGAGDGSGMGAV
GGTPSDCRYILTPGGLAEQPGQAEHQPESPLNETQRLLLLQQGLLPHSSC
>hsa:7363
MSMKWTSALLLIQLSCYFSSGSCGKVLVWPTEFSHWMNIKTILDELVQRGHEVTVLASSA
SISFDPNSPSTLKFEVYPVSLTKTEFEDIIKQLVKRWAELPKDTFWSYFSQVQEIMWTFN
DILRKFCKDIVSNKKLMKKLQESRFDVVLADAVFPFGELLAELLKIPFVYSLRFSPGYAI
EKHSGGLLFPPSYVPVVMSELSDQMTFIERVKNMIYVLYFEFWFQIFDMKKWDQFYSEVL
GRPTTLSETMAKADIWLIRNYWDFQFPHPLLPNVEFVGGLHCKPAKPLPKEMEEFVQSSG
ENGVVVFSLGSMVSNTSEERANVIASALAKIPQKVLWRFDGNKPDTLGLNTRLYKWIPQN

DLLGHPKTRAFITHGGANGIYEAIYHGIPMVGVPLFADQPDNIAHMKAKGAAVSLDFHTM
SSTDLLNALKTVINDPLYKENAMKLSRIHHDQPVKPLDRAVFWIEFVMRHKGAKHLRVAA
HDLTWFQYHSLDVTGFLLACVATVIFIITKCLFCVWKFVRTGKKGKRD
>hsa:7364
MSVKWTSVILLIQLSFCFSSGNCGKVLVWAAEYSHWMNIKTILDELIQRGHEVTVLASSA
SILFDPNNSSALKIEIYPTSLTKTELENFIMQQIKRWSDLPKDTFWLYFSQVQEIMSIFG
DITRKFCKDVVSNKKFMKKVQESRFDVIFADAIFPCSELLAELFNIPFVYSLSFSPGYTF
EKHSGGFIFPPSYVPVVMSELTDQMTFMERVKNMIYVLYFDFWFEIFDMKKWDQFYSEVL
GRPTTLSETMGKADVWLIRNSWNFQFPYPLLPNVDFVGGLHCKPAKPLPKEMEDFVQSSG
ENGVVVFSLGSMVSNMTEERANVIASALAQIPQKVLWRFDGNKPDTLGLNTRLYKWIPQN
DLLGHPKTRAFITHGGANGIYEAIYHGIPMVGIPLFADQPDNIAHMKARGAAVRVDFNTM
SSTDLLNALKRVINDPSYKENVMKLSRIQHDQPVKPLDRAVFWIEFVMRHKGAKHLRVAA
HDLTWFQYHSLDVIGFLLVCVATVIFIVTKCCLFCFWKFARKAKKGKND
>hsa:7365
MALKWTTVLLIQLSFYFSSGSCGKVLVWAAEYSLWMNMKTILKELVQRGHEVTVLASSAS
ILFDPNDSSTLKLEVYPTSLTKTEFENIIMQLVKRLSEIQKDTFWLPFSQEQEILWAIND
IIRNFCKDVVSNKKLMKKLQESRFDIVFADAYLPCGELLAELFNIPFVYSHSFSPGYSFE
RHSGGFIFPPSYVPVVMSKLSDQMTFMERVKNMLYVLYFDFWFQIFNMKKWDQFYSEVLG
RPTTLSETMRKADIWLMRNSWNFKFPHPFLPNVDFVGGLHCKPAKPLPKEMEEFVQSSGE
NGVVVFSLGSMVSNMTEERANVIATALAKIPQKVLWRFDGNKPDALGLNTRLYKWIPQND
LLGHPKTRAFITHGGANGIYEAIYHGIPMVGIPLFFDQPDNIAHMKAKGAAVRVDFNTMS
STDLLNALKTVINDPSYKENIMKLSRIQHDQPVKPLDRAVFWIEFVMRHKGAKHLRVAAH
NLTWFQYHSLDVIGFLLACVATVLFIITKCCLFCFWKFARKGKKGKRD
>hsa:7366
MSLKWTSVFLLIQLSCYFSSGSCGKVLVWPTEYSHWINMKTILEELVQRGHEVTVLTSSA
STLVNASKSSAIKLEVYPTSLTKNYLEDSLLKILDRWIYGVSKNTFWSYFSQLQELCWEY
YDYSNKLCKDAVLNKKLMMKLQESKFDVILADALNPCGELLAELFNIPFLYSLRFSVGYT
FEKNGGGFLFPPSYVPVVMSELSDQMIFMERIKNMIHMLYFDFWFQIYDLKKWDQFYSEV
LGRPTTLFETMGKAEMWLIRTYWDFEFPRPFLPNVDFVGGLHCKPAKPLPKEMEEFVQSS
GENGIVVFSLGSMISNMSEESANMIASALAQIPQKVLWRFDGKKPNTLGSNTRLYKWLPQ
NDLLGHPKTKAFITHGGTNGIYEAIYHGIPMVGIPLFADQHDNIAHMKAKGAALSVDIRT
MSSRDLLNALKSVINDPVYKENVMKLSRIHHDQPMKPLDRAVFWIEFVMRHKGAKHLRVA
AHNLTWIQYHSLDVIAFLLACVATVIFIITKFCLFCFRKLAKKGKKKKRD
>hsa:7367
MSLKWMSVFLLMQLSCYFSSGSCGKVLVWPTEYSHWINMKTILEELVQRGHEVIVLTSSA
SILVNASKSSAIKLEVYPTSLTKNDLEDFFMKMFDRWTYSISKNTFWSYFSQLQELCWEY
SDYNIKLCEDAVLNKKLMRKLQESKFDVLLADAVNPCGELLAELLNIPFLYSLRFSVGYT
VEKNGGGFLFPPSYVPVVMSELSDQMIFMERIKNMIYMLYFDFWFQAYDLKKWDQFYSEV
LGRPTTLFETMGKAEMWLIRTYWDFEFPRPFLPNVDFVGGLHCKPAKPLPKEMEEFVQSS
GENGIVVFSLGSMISNMSEESANMIASALAQIPQKVLWRFDGKKPNTLGSNTRLYKWLPQ
NDLLGHPKTKAFITHGGTNGIYEAIYHGIPMVGIPLFADQHDNIAHMKAKGAALSVDIRT
MSSRDLLNALKSVINDPIYKENIMKLSRIHHDQPVKPLDRAVFWIEFVMRHKGAKHLRVA
AHNLTWIQYHSLDVIAFLLACVATMIFMITKCCLFCFRKLAKTGKKKKRD
>hsa:7371

MAGDSEQTLQNHQQPNGGEPFLIGVSGGTASGKSSVCAKIVQLLGQNEVDYRQKQVVILS
QDSFYRVLTSEQKAKALKGQFNFDHPDAFDNELILKTLKEITEGKTVQIPVYDFVSHSRK
EETVTVYPADVVLFEGILAFYSQEVRDLFQMKLFVDTDADTRLSRRVLRDISERGRDLEQ
ILSQYITFVKPAFEEFCLPTKKYADVIIPRGADNLVAINLIVQHIQDILNGGPSKRQTNG
CLNGYTPSRKRQASESSSRPH
>hsa:7372
MAVARAALGPLVTGLYDVQAFKFGDFVLKSGLSSPIYIDLRGIVSRPRLLSQVADILFQT
AQNAGISFDTVCGVPYTALPLATVICSTNQIPMLIRRKETKDYGTKRLVEGTINPGETCL
IIEDVVTSGSSVLETVEVLQKEGLKVTDAIVLLDREQGGKDKLQAHGIRLHSVCTLSKML
EILEQQKKVDAETVGRVKRFIQENVFVAANHNGSPLSIKEAPKELSFGARAELPRIHPVA
SKLLRLMQKKETNLCLSADVSLARELLQLADALGPSICMLKTHVDILNDFTLDVMKELIT
LAKCHEFLIFEDRKFADIGNTVKKQYEGGIFKIASWADLVNAHVVPGSGVVKGLQEVGLP
LHRGCLLIAEMSSTGSLATGDYTRAAVRMAEEHSEFVVGFISGSRVSMKPEFLHLTPGVQ
LEAGGDNLGQQYNSPQEVIGKRGSDIIIVGRGIISAADRLEAAEMYRKAAWEAYLSRLGV
>hsa:7378
MAATGANAEKAESHNDCPVRLLNPNIAKMKEDILYHFNLTTSRHNFPALFGDVKFVCVGG
SPSRMKAFIRCVGAELGLDCPGRDYPNICAGTDRYAMYKVGPVLSVSHGMGIPSISIMLH
ELIKLLYYARCSNVTIIRIGTSGGIGLEPGTVVITEQAVDTCFKAEFEQIVLGKRVIRKT
DLNKKLVQELLLCSAELSEFTTVVGNTMCTLDFYEGQGRLDGALCSYTEKDKQAYLEAAY
AAGVRNIEMESSVFAAMCSACGLQAAVVCVTLLNRLEGDQISSPRNVLSEYQQRPQRLVS
YFIKKKLSKA
>hsa:7453
MPNSEPASLLELFNSIATQGELVRSLKAGNASKDEIDSAVKMLVSLKMSYKAAAGEDYKA
DCPPGNPAPTSNHGPDATEAEEDFVDPWTVQTSSAKGIDYDKLIVRFGSSKIDKELINRI
ERATGQRPHHFLRRGIFFSHRDMNQVLDAYENKKPFYLYTGRGPSSEAMHVGHLIPFIFT
KWLQDVFNVPLVIQMTDDEKYLWKDLTLDQAYSYAVENAKDIIACGFDINKTFIFSDLDY
MGMSSGFYKNVVKIQKHVTFNQVKGIFGFTDSDCIGKISFPAIQAAPSFSNSFPQIFRDR
TDIQCLIPCAIDQDPYFRMTRDVAPRIGYPKPALLHSTFFPALQGAQTKMSASDPNSSIF
LTDTAKQIKTKVNKHAFSGGRDTIEEHRQFGGNCDVDVSFMYLTFFLEDDDKLEQIRKDY
TSGAMLTGELKKALIEVLQPLIAEHQARRKEVTDEIVKEFMTPRKLSFDFQ
>hsa:7498
MTADKLVFFVNGRKVVEKNADPETTLLAYLRRKLGLSGTKLGCGEGGCGACTVMLSKYDR
LQNKIVHFSANACLAPICSLHHVAVTTVEGIGSTKTRLHPVQERIAKSHGSQCGFCTPGI
VMSMYTLLRNQPEPTMEEIENAFQGNLCRCTGYRPILQGFRTFARDGGCCGGDGNNPNCC
MNQKKDHSVSLSPSLFKPEEFTPLDPTQEPIFPPELLRLKDTPRKQLRFEGERVTWIQAS
TLKELLDLKAQHPDAKLVVGNTEIGIEMKFKNMLFPMIVCPAWIPELNSVEHGPDGISFG
AACPLSIVEKTLVDAVAKLPAQKTEVFRGVLEQLRWFAGKQVKSVASVGGNIITASPISD
LNPVFMASGAKLTLVSRGTRRTVQMDHTFFPGYRKTLLSPEEILLSIEIPYSREGEYFSA
FKQASRREDDIAKVTSGMRVLFKPGTTEVQELALCYGGMANRTISALKTTQRQLSKLWKE
ELLQDVCAGLAEELHLPPDAPGGMVDFRCTLTLSFFFKFYLTVLQKLGQENLEDKCGKLD
PTFASATLLFQKDPPADVQLFQEVPKGQSEEDMVGRPLPHLAADMQASGEAVYCDDIPRY
ENELSLRLVTSTRAHAKIKSIDTSEAKKVPGFVCFISADDVPGSNITGICNDETVFAKDK
VTCVGHIIGAVVADTPEHTQRAAQGVKITYEELPAIITIEDAIKNNSFYGPELKIEKGDL
KKGFSEADNVVSGEIYIGGQEHFYLETHCTIAVPKGEAGEMELFVSTQNTMKTQSFVAKM

LGVPANRIVVRVKRMGGGFGGKETRSTVVSTAVALAAYKTGRPVRCMLDRDEDMLITGGR
HPFLARYKVGFMKTGTVVALEVDHFSNVGNTQDLSQSIMERALFHMDNCYKIPNIRGTGR
LCKTNLPSNTAFRGFGGPQGMLIAECWMSEVAVTCGMPAEEVRRKNLYKEGDLTHFNQKL
EGFTLPRCWEECLASSQYHARKSEVDKFNKENCWKKRGLCIIPTKFGISFTVPFLNQAGA
LLHVYTDGSVLLTHGGTEMGQGLHTKMVQVASRALKIPTSKIYISETSTNTVPNTSPTAA
SVSADLNGQAVYAACQTILKRLEPYKKKNPSGSWEDWVTAAYMDTVSLSATGFYRTPNLG
YSFETNSGNPFHYFSYGVACSEVEIDCLTGDHKNLRTDIVMDVGSSLNPAIDIGQVEGAF
VQGLGLFTLEELHYSPEGSLHTRGPSTYKIPAFGSIPIEFRVSLLRDCPNKKAIYASKAV
GEPPLFLAASIFFAIKDAIRAARAQHTGNNVKELFRLDSPATPEKIRNACVDKFTTLCVT
GVPENCKPWSVRV
>hsa:7525
MGCIKSKENKSPAIKYRPENTPEPVSTSVSHYGAEPTTVSPCPSSSAKGTAVNFSSLSMT
PFGGSSGVTPFGGASSSFSVVPSSYPAGLTGGVTIFVALYDYEARTTEDLSFKKGERFQI
INNTEGDWWEARSIATGKNGYIPSNYVAPADSIQAEEWYFGKMGRKDAERLLLNPGNQRG
IFLVRESETTKGAYSLSIRDWDEIRGDNVKHYKIRKLDNGGYYITTRAQFDTLQKLVKHY
TEHADGLCHKLTTVCPTVKPQTQGLAKDAWEIPRESLRLEVKLGQGCFGEVWMGTWNGTT
KVAIKTLKPGTMMPEAFLQEAQIMKKLRHDKLVPLYAVVSEEPIYIVTEFMSKGSLLDFL
KEGDGKYLKLPQLVDMAAQIADGMAYIERMNYIHRDLRAANILVGENLVCKIADFGLARL
IEDNEYTARQGAKFPIKWTAPEAALYGRFTIKSDVWSFGILQTELVTKGRVPYPGMVNRE
VLEQVERGYRMPCPQGCPESLHELMNLCWKKDPDERPTFEYIQSFLEDYFTATEPQYQPG
ENL
>hsa:7535
MPDPAAHLPFFYGSISRAEAEEHLKLAGMADGLFLLRQCLRSLGGYVLSLVHDVRFHHFP
IERQLNGTYAIAGGKAHCGPAELCEFYSRDPDGLPCNLRKPCNRPSGLEPQPGVFDCLRD
AMVRDYVRQTWKLEGEALEQAIISQAPQVEKLIATTAHERMPWYHSSLTREEAERKLYSG
AQTDGKFLLRPRKEQGTYALSLIYGKTVYHYLISQDKAGKYCIPEGTKFDTLWQLVEYLK
LKADGLIYCLKEACPNSSASNASGAAAPTLPAHPSTLTHPQRRIDTLNSDGYTPEPARIT
SPDKPRPMPMDTSVYESPYSDPEELKDKKLFLKRDNLLIADIELGCGNFGSVRQGVYRMR
KKQIDVAIKVLKQGTEKADTEEMMREAQIMHQLDNPYIVRLIGVCQAEALMLVMEMAGGG
PLHKFLVGKREEIPVSNVAELLHQVSMGMKYLEEKNFVHRDLAARNVLLVNRHYAKISDF
GLSKALGADDSYYTARSAGKWPLKWYAPECINFRKFSSRSDVWSYGVTMWEALSYGQKPY
KKMKGPEVMAFIEQGKRMECPPECPPELYALMSDCWIYKWEDRPDFLTVEQRMRACYYSL
ASKVEGPPGSTQKAEAACA
>hsa:759
MASPDWGYDDKNGPEQWSKLYPIANGNNQSPVDIKTSETKHDTSLKPISVSYNPATAKEI
INVGHSFHVNFEDNDNRSVLKGGPFSDSYRLFQFHFHWGSTNEHGSEHTVDGVKYSAELH
VAHWNSAKYSSLAEAASKADGLAVIGVLMKVGEANPKLQKVLDALQAIKTKGKRAPFTNF
DPSTLLPSSLDFWTYPGSLTHPPLYESVTWIICKESISVSSEQLAQFRSLLSNVEGDNAV
PMQHNNRPTQPLKGRTVRASF
>hsa:760
MSHHWGYGKHNGPEHWHKDFPIAKGERQSPVDIDTHTAKYDPSLKPLSVSYDQATSLRIL
NNGHAFNVEFDDSQDKAVLKGGPLDGTYRLIQFHFHWGSLDGQGSEHTVDKKKYAAELHL
VHWNTKYGDFGKAVQQPDGLAVLGIFLKVGSAKPGLQKVVDVLDSIKTKGKSADFTNFDP
RGLLPESLDYWTYPGSLTTPPLLECVTWIVLKEPISVSSEQVLKFRKLNFNGEGEPEELM

VDNWRPAQPLKNRQIKASFK
>hsa:761
MAKEWGYASHNGPDHWHELFPNAKGENQSPVELHTKDIRHDPSLQPWSVSYDGGSAKTIL
NNGKTCRVVFDDTYDRSMLRGGPLPGPYRLRQFHLHWGSSDDHGSEHTVDGVKYAAELHL
VHWNPKYNTFKEALKQRDGIAVIGIFLKIGHENGEFQIFLDALDKIKTKGKEAPFTKFDP
SCLFPACRDYWTYQGSFTTPPCEECIVWLLLKEPMTVSSDQMAKLRSLLSSAENEPPVPL
VSNWRPPQPINNRVVRASFK
>hsa:762
MRMLLALLALSAARPSASAESHWCYEVQAESSNYPCLVPVKWGGNCQKDRQSPINIVTTK
AKVDKKLGRFFFSGYDKKQTWTVQNNGHSVMMLLENKASISGGGLPAPYQAKQLHLHWSD
LPYKGSEHSLDGEHFAMEMHIVHEKEKGTSRNVKEAQDPEDEIAVLAFLVEAGTQVNEGF
QPLVEALSNIPKPEMSTTMAESSLLDLLPKEEKLRHYFRYLGSLTTPTCDEKVVWTVFRE
PIQLHREQILAFSQKLYYDKEQTVSMKDNVRPLQQLGQRTVIKSGAPGRPLPWALPALLG
PMLACLLAGFLR
>hsa:763
MLGRNTWKTSAFSFLVEQMWAPLWSRSMRPGRWCSQRSCAWQTSNNTLHPLWTVPVSVPG
GTRQSPINIQWRDSVYDPQLKPLRVSYEAASCLYIWNTGYLFQVEFDDATEASGISGGPL
ENHYRLKQFHFHWGAVNEGGSEHTVDGHAYPAELHLVHWNSVKYQNYKEAVVGENGLAVI
GVFLKLGAHHQTLQRLVDILPEIKHKDARAAMRPFDPSTLLPTCWDYWTYAGSLTTPPLT
ESVTWIIQKEPVEVAPSQLSAFRTLLFSALGEEEKMMVNNYRPLQPLMNRKVWASFQATN
EGTRS
>hsa:765
MRALVLLLSLFLLGGQAQHVSDWTYSEGALDEAHWPQHYPACGGQRQSPINLQRTKVRYN
PSLKGLNMTGYETQAGEFPMVNNGHTVQISLPSTMRMTVADGTVYIAQQMHFHWGGASSE
ISGSEHTVDGIRHVIEIHIVHYNSKYKSYDIAQDAPDGLAVLAAFVEVKNYPENTYYSNF
ISHLANIKYPGQRTTLTGLDVQDMLPRNLQHYYTYHGSLTTPPCTENVHWFVLADFVKLS
RTQVWKLENSLLDHRNKTIHNDYRRTQPLNHRVVESNFPNQEYTLGSEFQFYLHKIEEIL
DYLRRALN
>hsa:766
MSLSITNNGHSVQVDFNDSDDRTVVTGGPLEGPYRLKQFHFHWGKKHDVGSEHTVDGKSF
PSELHLVHWNAKKYSTFGEAASAPDGLAVVGVFLETGDEHPSMNRLTDALYMVRFKGTKA
QFSCFNPKCLLPASRHYWTYPGSLTTPPLSESVTWIVLREPICISERQMGKFRSLLFTSE
DDERIHMVNNFRPPQPLKGRVVKASFRA
>hsa:767
MADLSFIEDTVAFPEKEEDEEEEEEGVEWGYEEGVEWGLVFPDANGEYQSPINLNSREAR
YDPSLLDVRLSPNYVVCRDCEVTNDGHTIQVILKSKSVLSGGPLPQGHEFELYEVRFHWG
RENQRGSEHTVNFKAFPMELHLIHWNSTLFGSIDEAVGKPHGIAIIALFVQIGKEHVGLK
AVTEILQDIQYKGKSKTIPCFNPNTLLPDPLLRDYWVYEGSLTIPPCSEGVTWILFRYPL
TISQLQIEEFRRLRTHVKGAELVEGCDGILGDNFRPTQPLSDRVIRAAFQ
>hsa:768
MAPLCPSPWLPLLIPAPAPGLTVQLLLSLLLLVPVHPQRLPRMQEDSPLGGGSSGEDDPL
GEEDLPSEEDSPREEDPPGEEDLPGEEDLPGEEDLPEVKPKSEEEGSLKLEDLPTVEAPG
DPQEPQNNAHRDKEGDDQSHWRYGGDPPWPRVSPACAGRFQSPVDIRPQLAAFCPALRPL
ELLGFQLPPLPELRLRNNGHSVQLTLPPGLEMALGPGREYRALQLHLHWGAAGRPGSEHT

VEGHRFPAEIHVVHLSTAFARVDEALGRPGGLAVLAAFLEEGPEENSAYEQLLSRLEEIA
EEGSETQVPGLDISALLPSDFSRYFQYEGSLTTPPCAQGVIWTVFNQTVMLSAKQLHTLS
DTLWGPGDSRLQLNFRATQPLNGRVIEASFPAGVDSSPRAAEPVQLNSCLAAGDILALVF
GLLFAVTSVAFLVQMRRQHRRGTKGGVSYRPAEVAETGA
>hsa:771
MPRRSLHAAAVLLLVILKEQPSSPAPVNGSKWTYFGPDGENSWSKKYPSCGGLLQSPIDL
HSDILQYDASLTPLEFQGYNLSANKQFLLTNNGHSVKLNLPSDMHIQGLQSRYSATQLHL
HWGNPNDPHGSEHTVSGQHFAAELHIVHYNSDLYPDASTASNKSEGLAVLAVLIEMGSFN
PSYDKIFSHLQHVKYKGQEAFVPGFNIEELLPERTAEYYRYRGSLTTPPCNPTVLWTVFR
NPVQISQEQLLALETALYCTHMDDPSPREMINNFRQVQKFDERLVYTSFSQVQVCTAAGL
SLGIILSLALAGILGICIVVVVSIWLFRRKSIKKGDNKGVIYKPATKMETEAHA
>hsa:780
MGPEALSSLLLLLLVASGDADMKGHFDPAKCRYALGMQDRTIPDSDISASSSWSDSTAAR
HSRLESSDGDGAWCPAGSVFPKEEEYLQVDLQRLHLVALVGTQGRHAGGLGKEFSRSYRL
RYSRDGRRWMGWKDRWGQEVISGNEDPEGVVLKDLGPPMVARLVRFYPRADRVMSVCLRV
ELYGCLWRDGLLSYTAPVGQTMYLSEAVYLNDSTYDGHTVGGLQYGGLGQLADGVVGLDD
FRKSQELRVWPGYDYVGWSNHSFSSGYVEMEFEFDRLRAFQAMQVHCNNMHTLGARLPGG
VECRFRRGPAMAWEGEPMRHNLGGNLGDPRARAVSVPLGGRVARFLQCRFLFAGPWLLFS
EISFISDVVNNSSPALGGTFPPAPWWPPGPPPTNFSSLELEPRGQQPVAKAEGSPTAILI
GCLVAIILLLLLIIALMLWRLHWRRLLSKAERRVLEEELTVHLSVPGDTILINNRPGPRE
PPPYQEPRPRGNPPHSAPCVPNGSGAPV
>hsa:79001
MGSTWGSPGWVRLALCLTGLVLSLYALHVKAARARDRDYRALCDVGTAISCSRVFSSRWG
RGFGLVEHVLGQDSILNQSNSIFGCIFYTLQLLLGCLRTRWASVLMLLSSLVSLAGSVYL
AWILFFVLYDFCIVCITTYAINVSLMWLSFRKVQEPQGKAKRH
>hsa:7957
MRFRFGVVVPPAVAGARPELLVVGSRPELGRWEPRGAVRLRPAGTAAGDGALALQEPGLW
LGEVELAAEEAAQDGAEPGRVDTFWYKFLKREPGGELSWEGNGPHHDRCCTYNENNLVDG
VYCLPIGHWIEATGHTNEMKHTTDFYFNIAGHQAMHYSRILPNIWLGSCPRQVEHVTIKL
KHELGITAVMNFQTEWDIVQNSSGCNRYPEPMTPDTMIKLYREEGLAYIWMPTPDMSTEG
RVQMLPQAVCLLHALLEKGHIVYVHCNAGVGRSTAAVCGWLQYVMGWNLRKVQYFLMAKR
PAVYIDEEAASQDTFPL
>hsa:79799
MRSDKSALVFLLLQLFCVGCGFCGKVLVWPCDMSHWLNVKVILEELIVRGHEVTVLTHSK
PSLIDYRKPSALKFEVVHMPQDRTEENEIFVDLALNVLPGLSTWQSVIKLNDFFVEIRGT
LKMMCESFIYNQTLMKKLQETNYDVMLIDPVIPCGDLMAELLAVPFVLTLRISVGGNMER
SCGKLPAPLSYVPVPMTGLTDRMTFLERVKNSMLSVLFHFWIQDYDYHFWEEFYSKALGR
PTTLCETVGKAEIWLIRTYWDFEFPQPYQPNFEFVGGLHCKPAKALPKEMENFVQSSGED
GIVVFSLGSLFQNVTEEKANIIASALAQIPQKVLWRYKGKKPSTLGANTRLYDWIPQNDL
LGHPKTKAFITHGGMNGIYEAIYHGVPMVGVPIFGDQLDNIAHMKAKGAAVEINFKTMTS
EDLLRALRTVITDSSYKENAMRLSRIHHDQPVKPLDRAVFWIEFVMRHKGAKHLRSAAHD
LTWFQHYSIDVIGFLLACVATAIFLFTKCFLFSCQKFNKTRKIEKRE
>hsa:80339
MYDAERGWSLSFAGCGFLGFYHVGATRCLSEHAPHLLRDARMLFGASAGALHCVGVLSGI

PLEQTLQVLSDLVRKARSRNIGIFHPSFNLSKFLRQGLCKCLPANVHQLISGKIGISLTR
VSDGENVLVSDFRSKDEVVDALVCSCFIPFYSGLIPPSFRGVRYVDGGVSDNVPFIDAKT
TITVSPFYGEYDICPKVKSTNFLHVDITKLSLRLCTGNLYLLSRAFVPPDLKVLGEICLR
GYLDAFRFLEEKGICNRPQPGLKSSSEGMDPEVAMPSWANMSLDSSPESAALAVRLEGDE
LLDHLRLSILPWDESILDTLSPRLATALSEEMKDKGGYMSKICNLLPIRIMSYVMLPCTL
PVESAIAIVQRLVTWLPDMPDDVLWLQWVTSQVFTRVLMCLLPASRSQMPVSSQQASPCT
PEQDWPCWTPCSPKGCPAETKAEATPRSILRSSLNFFLGNKVPAGAEGLSTFPSFSLEKS
L
>hsa:80824
MAHEMIGTQIVTERLVALLESGTEKVLLIDSRPFVEYNTSHILEAININCSKLMKRRLQQ
DKVLITELIQHSAKHKVDIDCSQKVVVYDQSSQDVASLSSDCFLTVLLGKLEKSFNSVHL
LAGGFAEFSRCFPGLCEGKSTLVPTCISQPCLPVANIGPTRILPNLYLGCQRDVLNKELM
QQNGIGYVLNASNTCPKPDFIPESHFLRVPVNDSFCEKILPWLDKSVDFIEKAKASNGCV
LVHCLAGISRSATIAIAYIMKRMDMSLDEAYRFVKEKRPTISPNFNFLGQLLDYEKKIKN
QTGASGPKSKLKLLHLEKPNEPVPAVSEGGQKSETPLSPPCADSATSEAAGQRPVHPASV
PSVPSVQPSLLEDSPLVQALSGLHLSADRLEDSNKLKRSFSLDIKSVSYSASMAASLHGF
SSSEDALEYYKPSTTLDGTNKLCQFSPVQELSEQTPETSPDKEEASIPKKLQTARPSDSQ
SKRLHSVRTSSSGTAQRSLLSPLHRSGSVEDNYHTSFLFGLSTSQQHLTKSAGLGLKGWH
SDILAPQTSTPSLTSSWYFATESSHFYSASAIYGGSASYSAYSCSQLPTCGDQVYSVRRR
QKPSDRADSRRSWHEESPFEKQFKRRSCQMEFGESIMSENRSREELGKVGSQSSFSGSME
IIEVS
>hsa:81579
MALLSRPALTLLLLLMAAVVRCQEQAQTTDWRATLKTIRNGVHKIDTYLNAALDLLGGED
GLCQYKCSDGSKPFPRYGYKPSPPNGCGSPLFGVHLNIGIPSLTKCCNQHDRCYETCGKS
KNDCDEEFQYCLSKICRDVQKTLGLTQHVQACETTVELLFDSVIHLGCKPYLDSQRAACR
CHYEEKTDL
>hsa:8192
MWPGILVGGARVASCRYPALGPRLAAHFPAQRPPQRTLQNGLALQRCLHATATRALPLIP
IVVEQTGRGERAYDIYSRLLRERIVCVMGPIDDSVASLVIAQLLFLQSESNKKPIHMYIN
SPGGVVTAGLAIYDTMQYILNPICTWCVGQAASMGSLLLAAGTPGMRHSLPNSRIMIHQP
SGGARGQATDIAIQAEEIMKLKKQLYNIYAKHTKQSLQVIESAMERDRYMSPMEAQEFGI
LDKVLVHPPQDGEDEPTLVQKEPVEAAPAAEPVPAST
>hsa:8288
MHLLPALAGVLATLVLAQPCEGTDPASPGAVETSVLRDCIAEAKLLVDAAYNWTQKSIKQ
RLRSGSASPMDLLSYFKQPVAATRTVVRAADYMHVALGLLEEKLQPQRSGPFNVTDVLTE
PQLRLLSQASGCALRDQAERCSDKYRTITGRCNNKRRPLLGASNQALARWLPAEYEDGLS
LPFGWTPSRRRNGFLLPLVRAVSNQIVRFPNERLTSDRGRALMFMQWGQFIDHDLDFSPE
SPARVAFTAGVDCERTCAQLPPCFPIKIPPNDPRIKNQRDCIPFFRSAPSCPQNKNRVRN
QINALTSFVDASMVYGSEVSLSLRLRNRTNYLGLLAINQRFQDNGRALLPFDNLHDDPCL
LTNRSARIPCFLAGDTRSTETPKLAAMHTLFMREHNRLATELRRLNPRWNGDKLYNEARK
IMGAMVQIITYRDFLPLVLGKARARRTLGHYRGYCSNVDPRVANVFTLAFRFGHTMLQPF
MFRLDSQYRASAPNSHVPLSSAFFASWRIVYEGGIDPILRGLMATPAKLNRQDAMLVDEL
RDRLFRQVRRIGLDLAALNMQRSRDHGLPGYNAWRRFCGLSQPRNLAQLSRVLKNQDLAR
KFLNLYGTPDNIDIWIGAIAEPLLPGARVGPLLACLFENQFRRARDGDRFWWQKRGVFTK

RQRKALSRISLSRIICDNTGITTVSRDIFRANIYPRGFVNCSRIPRLNLSAWRGT
>hsa:834
MADKVLKEKRKLFIRSMGEGTINGLLDELLQTRVLNKEEMEKVKRENATVMDKTRALIDS
VIPKGAQACQICITYICEEDSYLAGTLGLSAAPQAVQDNPAMPTSSGSEGNVKLCSLEEA
QRIWKQKSAEIYPIMDKSSRTRLALIICNEEFDSIPRRTGAEVDITGMTMLLQNLGYSVD
VKKNLTASDMTTELEAFAHRPEHKTSDSTFLVFMSHGIREGICGKKHSEQVPDILQLNAI
FNMLNTKNCPSLKDKPKVIIIQACRGDSPGVVWFKDSVGVSGNLSLPTTEEFEDDAIKKA
HIEKDFIAFCSSTPDNVSWRHPTMGSVFIGRLIEHMQEYACSCDVEEIFRKVRFSFEQPD
GRAQMPTTERVTLTRCFYLFPGH
>hsa:8398
MQFFGRLVNTFSGVTNLFSNPFRVKEVAVADYTSSDRVREEGQLILFQNTPNRTWDCVLV
NPRNSQSGFRLFQLELEADALVNFHQYSSQLLPFYESSPQVLHTEVLQHLTDLIRNHPSW
SVAHLAVELGIRECFHHSRIISCANCAENEEGCTPLHLACRKGDGEILVELVQYCHTQMD
VTDYKGETVFHYAVQGDNSQVLQLLGRNAVAGLNQVNNQGLTPLHLACQLGKQEMVRVLL
LCNARCNIMGPNGYPIHSAMKFSQKGCAEMIISMDSSQIHSKDPRYGASPLHWAKNAEMA
RMLLKRGCNVNSTSSAGNTALHVAVMRNRFDCAIVLLTHGANADARGEHGNTPLHLAMSK
DNVEMIKALIVFGAEVDTPNDFGETPTFLASKIGRQLQDLMHISRARKPAFILGSMRDEK
RTHDHLLCLDGGGVKGLIIIQLLIAIEKASGVATKDLFDWVAGTSTGGILALAILHSKSM
AYMRGMYFRMKDEVFRGSRPYESGPLEEFLKREFGEHTKMTDVRKPKVMLTGTLSDRQPA
ELHLFRNYDAPETVREPRFNQNVNLRPPAQPSDQLVWRAARSSGAAPTYFRPNGRFLDGG
LLANNPTLDAMTEIHEYNQDLIRKGQANKVKKLSIVVSLGTGRSPQVPVTCVDVFRPSNP
WELAKTVFGAKELGKMVVDCCTDPDGRAVDRARAWCEMVGIQYFRLNPQLGTDIMLDEVS
DTVLVNALWETEVYIYEHREEFQKLIQLLLSP
>hsa:8399
MGPLPVCLPIMLLLLLPSLLLLLLLPGPGSGEASRILRVHRRGILELAGTVGCVGPRTPI
AYMKYGCFCGLGGHGQPRDAIDWCCHGHDCCYTRAEEAGCSPKTERYSWQCVNQSVLCGP
AENKCQELLCKCDQEIANCLAQTEYNLKYLFYPQFLCEPDSPKCD
>hsa:84152
MLFRLSEHSSPEEEASPHQRASGEGHHLKSKRPNPCAYTPPSLKAVQRIAESHLQSISNL
NENQASEEEDELGELRELGYPREEDEEEEEDDEEEEEEEDSQAEVLKVIRQSAGQKTTCG
QGLEGPWERPPPLDESERDGGSEDQVEDPALSEPGEEPQRPSPSEPGT
>hsa:84171
MAWSPPATLFLFLLLLGQPPPSRPQSLGTTKLRLVGPESKPEEGRLEVLHQGQWGTVCDD
NFAIQEATVACRQLGFEAALTWAHSAKYGQGEGPIWLDNVRCVGTESSLDQCGSNGWGVS
DCSHSEDVGVICHPRRHRGYLSETVSNALGPQGRRLEEVRLKPILASAKQHSPVTEGAVE
VKYEGHWRQVCDQGWTMNNSRVVCGMLGFPSEVPVDSHYYRKVWDLKMRDPKSRLKSLTN
KNSFWIHQVTCLGTEPHMANCQVQVAPARGKLRPACPGGMHAVVSCVAGPHFRPPKTKPQ
RKGSWAEEPRVRLRSGAQVGEGRVEVLMNRQWGTVCDHRWNLISASVVCRQLGFGSAREA
LFGARLGQGLGPIHLSEVRCRGYERTLSDCPALEGSQNGCQHENDAAVRCNVPNMGFQNQ
VRLAGGRIPEEGLLEVQVEVNGVPRWGSVCSENWGLTEAMVACRQLGLGFAIHAYKETWF
WSGTPRAQEVVMSGVRCSGTELALQQCQRHGPVHCSHGGGRFLAGVSCMDSAPDLVMNAQ
LVQETAYLEDRPLSQLYCAHEENCLSKSADHMDWPYGYRRLLRFSTQIYNLGRTDFRPKT
GRDSWVWHQCHRHYHSIEVFTHYDLLTLNGSKVAEGHKASFCLEDTNCPTGLQRRYACAN
FGEQGVTVGCWDTYRHDIDCQWVDITDVGPGNYIFQVIVNPHYEVAESDFSNNMLQCRCK

YDGHRVWLHNCHTGNSYPANAELSLEQEQRLRNNLI

>hsa:8435

MEPGGARLRLQRTEGLGGERERQPCGDGNTETHRAPDLVQWTRHMEAVKAQLLEQAQGQL
RELLDRAMREAIQSYPSQDKPLPPPPPGSLSRTQEPSLGKQKVFIIRKSLLDELMEVQHF
RTIYHMFIAGLCVFIISTLAIDFIDEGRLLLEFDLLIFSFGQLPLALVTWVPMFLSTLLA
PYQALRLWARGTWTQATGLGCALLAAHAVVLCALPVHVAVEHQLPPASRCVLVFEQVRFL
MKSYSFLREAVPGTLRARRGEGIQAPSFSSYLYFLFCPTLIYRETYPRTPYVRWNYVAKN
FAQALGCVLYACFILGRLCVPVFANMSREPFSTRALVLSILHATLPGIFMLLLIFFAFLH
CWLNAFAEMLRFGDRMFYRDWWNSTSFSNYYRTWNVVVHDWLYSYVYQDGLRLLGARARG
VAMLGVFLVSAVAHEYIFCFVLGFFYPVMLILFLVIGGMLNFMMHDQRTGPAWNVLMWTM
LFLGQGIQVSLYCQEWYARRHCPLPQATFWGLVTPRSWSCHT

>hsa:84532

MAARTLGRGVGRLLGSLRGLSGQPARPPCGVSAPRRAASGPSGSAPAVAAAAAQPGSYPA
LSAQAAREPAAFWGPLARDTLVWDTPYHTVWDCDFSTGKIGWFLGGQLNVSVNCLDQHVR
KSPESVALIWERDEPGTEVRITYRELLETTCRLANTLKRHGVHRGDRVAIYMPVSPLAVA
AMLACARIGAVHTVIFAGFSAESLAGRINDAKCKVVITFNQGLRGGRVVELKKIVDEAVK
HCPTVQHVLVAHRTDNKVHMGDLDVPLEQEMAKEDPVCAPESMGSEDMLFMLYTSGSTGM
PKGIVHTQAGYLLYAALTHKLVFDHQPGDIFGCVADIGWITGHSYVVYGPLCNGATSVLF
ESTPVYPNAGRYWETVERLKINQFYGAPTAVRLLLKYGDAWVKKYDRSSLRTLGSVGEPI
NCEAWEWLHRVVGDSRCTLVDTWWQTGGICIAPRPSEEGAEILPAMAMRPFFGIVPVLMD
EKGSVVEGSNVSGALCISQAWPGMARTIYGDHQRFVDAYFKAYPGYYFTGDGAYRTEGGY
YQITGRMDDVINISGHRLGTAEIEDAIADHPAVPESAVIGYPHDIKGEAAFAFIVVKDSA
GDSDVVVQELKSMVATKIAKYAVPDEILVVKRLPKTRSGKVMRRLLRKIITSEAQELGDT
TTLEDPSIIAEILSVYQKCKDKQAAAK

>hsa:84618

MEPGQPREPQEPREPGPGAETAAAPVWEEAKIFYDNLAPKKKPKSPKPQNAVTIAVSSRA
LFRMDEEQQIYTEQGVEEYVRYQLEHENEPFSPGPAFPFVKALEAVNRRLRELYPDSEDV
FDIVLMTNNHAQVGVRLINSINHYDLFIERFCMTGGNSPICYLKAYHTNLYLSADAEKVR
EAIDEGIAAATIFSPSRDVVVSQSQLRVAFDGDAVLFSDESERIVKAHGLDRFFEHEKAH
ENKPLAQGPLKGFLEALGRLQKKFYSKGLRLECPIRTYLVTARSAASSGARALKTLRSWG
LETDEALFLAGAPKGPLLEKIRPHIFFDDQMFHVAGAQEMGTVAAHVPYGVAQTPRRTAP
AKQAPSAQ

>hsa:84695

MRPVSVWQWSPWGLLLCLLCSSCLGSPSPSTGPEKKAGSQGLRFRLAGFPRKPYEGRVEI
QRAGEWGTICDDDFTLQAAHILCRELGFTEATGWTHSAKYGPGTGRIWLDNLSCSGTEQS
VTECASRGWGNSDCTHDEDAGVICKDQRLPGFSDSNVIEVEHHLQVEEVRIRPAVGWGRR
PLPVTEGLVEVRLPDGWSQVCDKGWSAHNSHVVCGMLGFPSEKRVNAAFYRLLAQRQQHS
FGLHGVACVGTEAHLSLCSLEFYRANDTARCPGGGPAVVSCVPGPVYAASSGQKKQQQSK
PQGEARVRLKGGAHPGEGRVEVLKASTWGTVCDRKWDLHAASVVCRELGFGSAREALSGA
RMGQGMGAIHLSEVRCSGQELSLWKCPHKNITAEDCSHSQDAGVRCNLPYTGAETRIRLS
GGRSQHEGRVEVQIGGPGPLRWGLICGDDWGTLEAMVACRQLGLGYANHGLQETWYWDSG
NITEVVMSGVRCTGTELSLDQCAHHGTHITCKRTGTRFTAGVICSETASDLLLHSALVQE
TAYIEDRPLHMLYCAAEENCLASSARSANWPYGHRRLLRFSSQIHNLGRADFRPKAGRHS
WVWHECHGHYHSMDIFTHYDILTPNGTKVAEGHKASFCLEDTECQEDVSKRYECANFGEQ

GITVGCWDLYRHDIDCQWIDITDVKPGNYILQVVINPNFEVAESDFTNNAMKCNCKYDGH
RIWVHNCHIGDAFSEEANRRFERYPGQTSNQII
>hsa:84706
MGQQPITFLRQVMALCTYPNLLDSPSFPEDAKKRARRILQACGGNSLGSYSASQGVNCIR
EDVAAYITRRDGGVPADPDNIYLTTGASDGISTILKILVSGGGKSRTGVMIPIPQYPLYS
AVISELDAIQVNYYLDEENCWALNVNELRRAVQEAKDHCDPKVLCIINPGNPTGQVQSRK
CIEDVIHFAWEEKLFLLADEVYQDNVYSPDCRFHSFKKVLYEMGPEYSSNVELASFHSTS
KGYMGECGYRGGYMEVINLHPEIKGQLVKLLSVRLCPPVSGQAAMDIVVNPPVAGEESFE
QFSREKESVLGNLAKKAKLTEDLFNQVPGIHCNPLQGAMYAFPRIFIPAKAVEAAQAHQM
APDMFYCMKLLEETGICVVPGSGFGQREGTYHFRMTILPPVEKLKTVLQKVKDFHINFLE
KYA
>hsa:84812
MASLLQDQLTTDQDLLLMQEGMPMRKVRSKSWKKLRYFRLQNDGMTVWHARQARGSAKPS
FSISDVETIRNGHDSELLRSLAEELPLEQGFTIVFHGRRSNLDLMANSVEEAQIWMRGLQ
LLVDLVTSMDHQERLDQWLSDWFQRGDKNQDGKMSFQEVQRLLHLMNVEMDQEYAFSLFQ
AADTSQSGTLEGEEFVQFYKALTKRAEVQELFESFSADGQKLTLLEFLDFLQEEQKERDC
TSELALELIDRYEPSDSGKLRHVLSMDGFLSYLCSKDGDIFNPACLPIYQDMTQPLNHYF
ICSSHNTYLVGDQLCGQSSVEGYIRALKRGCRCVEVDVWDGPSGEPVVYHGHTLTSRILF
KDVVATVAQYAFQTSDYPVILSLETHCSWEQQQTMARHLTEILGEQLLSTTLDGVLPTQL
PSPEELRRKILVKGKKLTLEEDLEYEEEEAEPELEESELALESQFETEPEPQEQNLQNKD
KKKKSKPILCPALSSLVIYLKSVSFRSFTHSKEHYHFYEISSFSETKAKRLIKEAGNEFV
QHNTWQLSRVYPSGLRTDSSNYNPQELWNAGCQMVAMNMQTAGLEMDICDGHFRQNGGCG
YVLKPDFLRDIQSSFHPEKPISPFKAQTLLIQVISGQQLPKVDKTKEGSIVDPLVKVQIF
GVRLDTARQETNYVENNGFNPYWGQTLCFRVLVPELAMLRFVVMDYDWKSRNDFIGQYTL
PWTCMQQGYRHIHLLSKDGISLRPASIFVYICIQEGLEGDES
>hsa:8513
MWLLLTMASLISVLGTTHGLFGKLHPGSPEVTMNISQMITYWGYPNEEYEVVTEDGYILE
VNRIPYGKKNSGNTDAGYDVWLGNSRGNTWARRNLYYSPDSVEFWAFSFDEMAKYDLPAT
IDFIVKKTGQKQLHYVGHSQGTTIGFIAFSTNPSLAKRIKTFYALAPVATVKYTKSLINK
LRFVPQSLFKFIFGDKIFYPHNFFDQFLATEVCSREMLNLLCSNALFIICGFDSKNFNTS
RLDVYLSHNPAGTSVQNMFHWTQAVKSGKFQAYDWGSPVQNRMHYDQSQPPYYNVTAMNV
PIAVWNGGKDLLADPQDVGLLLPKLPNLIYHKEIPFYNHLDFIWAMDAPQEVYNDIVSMI
SEDKK
>hsa:8529
MSQLSLSWLGLWPVAASPWLLLLLVGASWLLAHVLAWTYAFYDNCRRLRCFPQPPRRNWF
WGHQGMVNPTEEGMRVLTQLVATYPQGFKVWMGPISPLLSLCHPDIIRSVINASAAIAPK
DKFFYSFLEPWLGDGLLLSAGDKWSRHRRMLTPAFHFNILKPYMKIFNESVNIMHAKWQL
LASEGSACLDMFEHISLMTLDSLQKCVFSFDSHCQEKPSEYIAAILELSALVSKRHHEIL
LHIDFLYYLTPDGQRFRRACRLVHDFTDAVIQERRRTLPSQGVDDFLQAKAKSKTLDFID
VLLLSKDEDGKKLSDEDIRAEADTFMFEGHDTTASGLSWVLYHLAKHPEYQERCRQEVQE
LLKDREPKEIEWDDLAHLPFLTMCMKESLRLHPPVPVISRHVTQDIVLPDGRVIPKGIIC
LISVFGTHHNPAVWPDPEVYDPFRFDPENIKERSPLAFIPFSAGPRNCIGQTFAMAEMKV
VLALTLLRFRVLPDHTEPRRKPELVLRAEGGLWLRVEPLS
>hsa:85313

MAVLLETTLGDVVIDLYTEERPRACLNFLKLCKIKYYNYCLIHNVQRDFIIQTGDPTGTG
RGGESIFGQLYGDQASFFEAEKVPRIKHKKKGTVSMVNNGSDQHGSQFLITTGENLDYLD
GVHTVFGEVTEGMDIIKKINETFVDKDFVPYQDIRINHTVILDDPFDDPPDLLIPDRSPE
PTREQLDSGRIGADEEIDDFKGRSAEEVEEIKAEKEAKTQAILLEMVGDLPDADIKPPEN
VLFVCKLNPVTTDEDLEIIFSRFGPIRSCEVIRDWKTGESLCYAFIEFEKEEDCEKAFFK
MDNVLIDDRRIHVDFSQSVAKVKWKGKGGKYTKSDFKEYEKEQDKPPNLVLKDKVKPKQD
TKYDLILDEQAEDSKSSHSHTSKKHKKKTHHCSEEKEDEDYMPIKNTNQDIYREMGFGHY
EEEESCWEKQKSEKRDRTQNRSRSRSRERDGHYSNSHKSKYQTDLYERERSKKRDRSRSP
KKSKDKEKSKYR
>hsa:8555
MSREGAGAALVAEVIKDRLCFAILYSRPKSASNVHYFSIDNELEYENFYADFGPLNLAMV
YRYCCKINKKLKSITMLRKKIVHFTGSDQRKQANAAFLVGCYMVIYLGRTPEEAYRILIF
GETSYIPFRDAAYGSCNFYITLLDCFHAVKKAMQYGFLNFNSFNLDEYEHYEKAENGDLN
WIIPDRFIAFCGPHSRARLESGYHQHSPETYIQYFKNHNVTTIIRLNKRMYDAKRFTDAG
FDHHDLFFADGSTPTDAIVKEFLDICENAEGAIAVHCKAGLGRTGTLIACYIMKHYRMTA
AETIAWVRICRPGSVIGPQQQFLVMKQTNLWLEGDYFRQKLKGQENGQHRAAFSKLLSGV
DDISINGVENQDQQEPEPYSDDDEINGVTQGDRLRALKSRRQSKTNAIPLTVILQSSVQS
CKTSEPNISGSAGITKRTTRSASRKSSVKSLSISRTKTVLR
>hsa:8556
MAAESGELIGACEFMKDRLYFATLRNRPKSTVNTHYFSIDEELVYENFYADFGPLNLAMV
YRYCCKLNKKLKSYSLSRKKIVHYTCFDQRKRANAAFLIGAYAVIYLKKTPEEAYRALLS
GSNPPYLPFRDASFGNCTYNLTILDCLQGIRKGLQHGFFDFETFDVDEYEHYERVENGDF
NWIVPGKFLAFSGPHPKSKIENGYPLHAPEAYFPYFKKHNVTAVVRLNKKIYEAKRFTDA
GFEHYDLFFIDGSTPSDNIVRRFLNICENTEGAIAVHCKAGLGRTGTLIACYVMKHYRFT
HAEIIAWIRICRPGSIIGPQQHFLEEKQASLWVQGDIFRSKLKNRPSSEGSINKILSGLD
DMSIGGNLSKTQNMERFGEDNLEDDDVEMKNGITQGDKLRALKSQRQPRTSPSCAFRSDD
TKGHPRAVSQPFRLSSSLQGSAVTLKTSKMALSPSATAKRINRTSLSSGATVRSFSINSR
LASSLGNLNAATDDPENKKTSSSSKAGFTASPFTNLLNGSSQPTTRNYPELNNNQYNRSS
NSNGGNLNSPPGPHSAKTEEHTTILRPSYTGLSSSSARFLSRSIPSLQSEYVHY
>hsa:8622
MGCAPSIHVSQSGVIYCRDSDESSSPRQTTSVSQGPAAPLPGLFVQTDAADAIPPSRASG
PPSVARVRRARTELGSGSSAGSAAPAATTSRGRRRHCCSSAEAETQTCYTSVKQVSSAEV
RIGPMRLTQDPIQVLLIFAKEDSQSDGFWWACDRAGYRCNIARTPESALECFLDKHHEII
VIDHRQTQNFDAEAVCRSIRATNPSEHTVILAVVSRVSDDHEEASVLPLLHAGFNRRFME
NSSIIACYNELIQIEHGEVRSQFKLRACNSVFTALDHCHEAIEITSDDHVIQIHKIHRDS
GDNSQTEPHSFRYKNRRKESIDVKSISSRGSDAPSLQNRRYPSMARIHSMTIEAPITKVI
NIINAAQENSPVTVAEALDRVLEILRTTELYSPQLGTKDEDPHTSDLVGGLMTDGLRRLS
GNEYVFTKNVHQSHSHLAMPITINDVPPCISQLLDNEESWDFNIFELEAITHKRPLVYLG
LKVFSRFGVCEFLNCSETTLRAWFQVIEANYHSSNAYHNSTHAADVLHATAFFLGKERVK
GSLDQLDEVAALIAATVHDVDHPGRTNSFLCNAGSELAVLYNDTAVLESHHTALAFQLTV
KDTKCNIFKNIDRNHYRTLRQAIIDMVLATEMTKHFEHVNKFVNSINKPMAAEIEGSDCE
CNPAGKNFPENQILIKRMMIKCADVANPCRPLDLCIEWAGRISEEYFAQTDEEKRQGLPV
VMPVFDRNTCSIPKSQISFIDYFITDMFDAWDAFAHLPALMQHLADNYKHWKTLDDLKCK
SLRLPSDS

>hsa:8654
MERAGPSFGQQRQQQQPQQQKQQQRDQDSVEAWLDDHWDFTFSYFVRKATREMVNAWFAE
RVHTIPVCKEGIRGHTESCSCPLQQSPRADNSAPGTPTRKISASEFDRPLRPIVVKDSEG
TVSFLSDSEKKEQMPLTPPRFDHDEGDQCSRLLELVKDISSHLDVTALCHKIFLHIHGLI
SADRYSLFLVCEDSSNDKFLISRLFDVAEGSTLEEVSNNCIRLEWNKGIVGHVAALGEPL
NIKDAYEDPRFNAEVDQITGYKTQSILCMPIKNHREEVVGVAQAINKKSGNGGTFTEKDE
KDFAAYLAFCGIVLHNAQLYETSLLENKRNQVLLDLASLIFEEQQSLEVILKKIAATIIS
FMQVQKCTIFIVDEDCSDSFSSVFHMECEELEKSSDTLTREHDANKINYMYAQYVKNTME
PLNIPDVSKDKRFPWTTENTGNVNQQCIRSLLCTPIKNGKKNKVIGVCQLVNKMEENTGK
VKPFNRNDEQFLEAFVIFCGLGIQNTQMYEAVERAMAKQMVTLEVLSYHASAAEEETREL
QSLAAAVVPSAQTLKITDFSFSDFELSDLETALCTIRMFTDLNLVQNFQMKHEVLCRWIL
SVKKNYRKNVAYHNWRHAFNTAQCMFAALKAGKIQNKLTDLEILALLIAALSHDLDHRGV
NNSYIQRSEHPLAQLYCHSIMEHHHFDQCLMILNSPGNQILSGLSIEEYKTTLKIIKQAI
LATDLALYIKRRGEFFELIRKNQFNLEDPHQKELFLAMLMTACDLSAITKPWPIQQRIAE
LVATEFFDQGDRERKELNIEPTDLMNREKKNKIPSMQVGFIDAICLQLYEALTHVSEDCF
PLLDGCRKNRQKWQALAEQQEKMLINGESGQAKRN
>hsa:8836
MASPGCLLCVLGLLLCGAASLELSRPHGDTAKKPIIGILMQKCRNKVMKNYGRYYIAASY
VKYLESAGARVVPVRLDLTEKDYEILFKSINGILFPGGSVDLRRSDYAKVAKIFYNLSIQ
SFDDGDYFPVWGTCLGFEELSLLISGECLLTATDTVDVAMPLNFTGGQLHSRMFQNFPTE
LLLSLAVEPLTANFHKWSLSVKNFTMNEKLKKFFNVLTTNTDGKIEFISTMEGYKYPVYG
VQWHPEKAPYEWKNLDGISHAPNAVKTAFYLAEFFVNEARKNNHHFKSESEEEKALIYQF
SPIYTGNISSFQQCYIFD
>hsa:8854
MKNQCETVWLKSPIKLKLIFINNEWQNSESGRVFPVYNPATGEQVCEVQEADKADIDKAV
QAARLAFSLGSVWRRMDASERGRLLDKLADLVERDRAVLATMESLNGGKPFLQAFYVDLQ
GVIKTFRYYAGWADKIHGMTIPVDGDYFTFTRHEPIGVCGQIIPWNFPLLMFAWKIAPAL
CCGNTVVIKPAEQTPLSALYMGALIKEAGFPPGVINILPGYGPTAGAAIASHIGIDKIAF
TGSTEVGKLIQEAAGRSNLKRVTLELGGKSPNIIFADADLDYAVEQAHQGVFFNQGQCCT
AGSRIFVEESIYEEFVRRSVERAKRRVVGSPFDPTTEQGPQIDKKQYNKILELIQSGVAE
GAKLECGGKGLGRKGFFIEPTVFSNVTDDMRIAKEEIFGPVQEILRFKTMDEVIERANNS
DFGLVAAVFTNDINKALTVSSAMQAGTVWINCYNALNAQSPFGGFKMSGNGREMGEFGLR
EYSEVKTVTVKIPQKNS
>hsa:8940
MKTVLMVAEKPSLAQSIAKILSRGSLSSHKGLNGACSVHEYTGTFAGQPVRFKMTSVCGH
VMTLDFLGKYNKWDKVDPAELFSQAPTEKKEANPKLNMVKFLQVEGRGCDYIVLWLDCDK
EGENICFEVLDAVLPVMNKAHGGEKTVFRARFSSITDTDICNAMACLGEPDHNEALSVDA
RQELDLRIGCAFTRFQTKYFQGKYGDLDSSLISFGPCQTPTLGFCVERHDKIQSFKPETY
WVLQAKVNTDKDRSLLLDWDRVRVFDREIAQMFLNMTKLEKEAQVEATSRKEKAKQRPLA
LNTVEMLRVASSSLGMGPQHAMQTAERLYTQGYISYPRTETTHYPENFDLKGSLRQQANH
PYWADTVKRLLAEGINRPRKGHDAGDHPPITPMKSATEAELGGDAWRLYEYITRHFIATV
SHDCKYLQSTISFRIGPELFTCSGKTVLSPGFTEVMPWQSVPLEESLPTCQRGDAFPVGE
VKMLEKQTNPPDYLTEAELITLMEKHGIGTDASIPVHINNICQRNYVTVESGRRLKPTNL
GIVLVHGYYKIDAELVLPTIRSAVEKQLNLIAQGKADYRQVLGHTLDVFKRKFHYFVDSI

AGMDELMEVSFSPLAATGKPLSRCGKCHRFMKYIQAKPSRLHCSHCDETYTLPQNGTIKL
YKELRCPLDDFELVLWSSGSRGKSYPLCPYCYNHPPFRDMKKGMGCNECTHPSCQHSLSM
LGIGQCVECESGVLVLDPTSGPKWKVACNKCNVVAHCFENAHRVRVSADTCSVCEAALLD
VDFNKAKSPLPGDETQHMGCVFCDPVFQELVELKHAASCHPMHRGGPGRRQGRGRGRARR
PPGKPNPRRPKDKMSALAAYFV
>hsa:8972
MARKKLKKFTTLEIVLSVLLLVLFIISIVLIVLLAKESLKSTAPDPGTTGTPDPGTTGTP
DPGTTGTTHARTTGPPDPGTTGTTPVSAECPVVNELERINCIPDQPPTKATCDQRGCCWN
PQGAVSVPWCYYSKNHSYHVEGNLVNTNAGFTARLKNLPSSPVFGSNVDNVLLTAEYQTS
NRFHFKLTDQTNNRFEVPHEHVQSFSGNAAASLTYQVEISRQPFSIKVTRRSNNRVLFDS
SIGPLLFADQFLQLSTRLPSTNVYGLGEHVHQQYRHDMNWKTWPIFNRDTTPNGNGTNLY
GAQTFFLCLEDASGLSFGVFLMNSNAMEVVLQPAPAITYRTIGGILDFYVFLGNTPEQVV
QEYLELIGRPALPSYWALGFHLSRYEYGTLDNMREVVERNRAAQLPYDVQHADIDYMDER
RDFTYDSVDFKGFPEFVNELHNNGQKLVIIVDPAISNNSSSSKPYGPYDRGSDMKIWVNS
SDGVTPLIGEVWPGQTVFPDYTNPNCAVWWTKEFELFHNQVEFDGIWIDMNEVSNFVDGS
VSGCSTNNLNNPPFTPRILDGYLFCKTLCMDAVQHWGKQYDIHNLYGYSMAVATAEAAKT
VFPNKRSFILTRSTFAGSGKFAAHWLGDNTATWDDLRWSIPGVLEFNLFGIPMVGPDICG
FALDTPEELCRRWMQLGAFYPFSRNHNGQGYKDQDPASFGADSLLLNSSRHYLNIRYTLL
PYLYTLFFRAHSRGDTVARPLLHEFYEDNSTWDVHQQFLWGPGLLITPVLDEGAEKVMAY
VPDAVWYDYETGSQVRWRKQKVEMELPGDKIGLHLRGGYIFPTQQPNTTTLASRKNPLGL
IIALDENKEAKGELFWDNGETKDTVANKVYLLCEFSVTQNRLEVNISQSTYKDPNNLAFN
EIKILGTEEPSNVTVKHNGVPSQTSPTVTYDSNLKVAIITDIDLLLGEAYTVEWSIKIRD
EEKIDCYPDENGASAENCTARGCIWEASNSSGVPFCYFVNDLYSVSDVQYNSHGATADIS
LKSSVYANAFPSTPVNPLRLDVTYHKNEMLQFKIYDPNKNRYEVPVPLNIPSMPSSTPEG
QLYDVLIKKNPFGIEIRRKSTGTIIWDSQLLGFTFSDMFIRISTRLPSKYLYGFGETEHR
SYRRDLEWHTWGMFSRDQPPGYKKNSYGVHPYYMGLEEDGSAHGVLLLNSNAMDVTFQPL
PALTYRTTGGVLDFYVFLGPTPELVTQQYTELIGRPVMVPYWSLGFQLCRYGYQNDSEIA
SLYDEMVAAQIPYDVQYSDIDYMERQLDFTLSPKFAGFPALINRMKADGMRVILILDPAI
SGNETQPYPAFTRGVEDDVFIKYPNDGDIVWGKVWPDFPDVVVNGSLDWDSQVELYRAYV
AFPDFFRNSTAKWWKREIEELYNNPQNPERSLKFDGMWIDMNEPSSFVNGAVSPGCRDAS
LNHPPYMPHLESRDRGLSSKTLCMESQQILPDGSLVQHYNVHNLYGWSQTRPTYEAVQEV
TGQRGVVITRSTFPSSGRWAGHWLGDNTAAWDQLKKSIIGMMEFSLFGISYTGADICGFF
QDAEYEMCVRWMQLGAFYPFSRNHNTIGTRRQDPVSWDVAFVNISRTVLQTRYTLLPYLY
TLMHKAHTEGVTVVRPLLHEFVSDQVTWDIDSQFLLGPAFLVSPVLERNARNVTAYFPRA
RWYDYYTGVDINARGEWKTLPAPLDHINLHVRGGYILPWQEPALNTHLSRQKFMGFKIAL
DDEGTAGGWLFWDDGQSIDTYGKGLYYLASFSASQNTMQSHIIFNNYITGTNPLKLGYIE
IWGVGSVPVTSVSISVSGMVITPSFNNDPTTQVLSIDVTDRNISLHNFTSLTWISTL
>hsa:90
MVDGVMILPVLIMIALPSPSMEDEKPKVNPKLYMCVCEGLSCGNEDHCEGQQCFSSLSIN
DGFHVYQKGCFQVYEQGKMTCKTPPSPGQAVECCQGDWCNRNITAQLPTKGKSFPGTQNF
HLEVGLIILSVVFAVCLLACLLGVALRKFKRRNQERLNPRDVEYGTIEGLITTNVGDSTL
ADLLDHSCTSGSGSGLPFLVQRTVARQITLLECVGKGRYGEVWRGSWQGENVAVKIFSSR
DEKSWFRETELYNTVMLRHENILGFIASDMTSRHSSTQLWLITHYHEMGSLYDYLQLTTL
DTVSCLRIVLSIASGLAHLHIEIFGTQGKPAIAHRDLKSKNILVKKNGQCCIADLGLAVM

HSQSTNQLDVGNNPRVGTKRYMAPEVLDETIQVDCFDSYKRVDIWAFGLVLWEVARRMVS
NGIVEDYKPPFYDVVPNDPSFEDMRKVVCVDQQRPNIPNRWFSDPTLTSLAKLMKECWYQ
NPSARLTALRIKKTLTKIDNSLDKLKTDC
>hsa:9023
MSCHNCSDPQVLCSSGQLFLQPLWDHLRSWEALLQSPFFPVIFSITTYVGFCLPFVVLDI
LCSWVPALRRYKIHPDFSPSAQQLLPCLGQTLYQHVMFVFPVTLLHWARSPALLPHEAPE
LLLLLHHILFCLLLFDMEFFVWHLLHHKVPWLYRTFHKVHHQNSSSFALATQYMSVWELF
SLGFFDMMNVTLLGCHPLTTLTFHVVNIWLSVEDHSGYNFPWSTHRLVPFGWYGGVVHHD
LHHSHFNCNFAPYFTHWDKILGTLRTASVPAR
>hsa:9088
MLERWHQLQPRRVSFRGEASETLQSPGYDPSRPESFFQQSFQRLSRLGHGSYGEVFKVRS
KEDGRLYAVKRSMSPFRGPKDRARKLAEVGSHEKVGQHPCCVRLEQAWEEGGILYLQTEL
CGPSLQQHCEAWGASLPEAQVWGYLRDTLLALAHLHSQGLVHLDVKPANIFLGPRGRCKL
GDFGLLVELGTAGAGEVQEGDPRYMAPELLQGSYGTAADVFSLGLTILEVACNMELPHGG
EGWQQLRQGYLPPEFTAGLSSELRSVLVMMLEPDPKLRATAEALLALPVLRQPRAWGVLW
CMAAEALSRGWALWQALLALLCWLWHGLAHPASWLQPLGPPATPPGSPPCSLLLDSSLSS
NWDDDSLGPSLSPEAVLARTVGSTSTPRSRCTPRDALDLSDINSEPPRGSFPSFEPRNLL
SLFEDTLDPT
>hsa:91
MAESAGASSFFPLVVLLLAGSGGSGPRGVQALLCACTSCLQANYTCETDGACMVSIFNLD
GMEHHVRTCIPKVELVPAGKPFYCLSSEDLRNTHCCYTDYCNRIDLRVPSGHLKEPEHPS
MWGPVELVGIIAGPVFLLFLIIIIVFLVINYHQRVYHNRQRLDMEDPSCEMCLSKDKTLQ
DLVYDLSTSGSGSGLPLFVQRTVARTIVLQEIIGKGRFGEVWRGRWRGGDVAVKIFSSRE
ERSWFREAEIYQTVMLRHENILGFIAADNKDNGTWTQLWLVSDYHEHGSLFDYLNRYTVT
IEGMIKLALSAASGLAHLHMEIVGTQGKPGIAHRDLKSKNILVKKNGMCAIADLGLAVRH
DAVTDTIDIAPNQRVGTKRYMAPEVLDETINMKHFDSFKCADIYALGLVYWEIARRCNSG
GVHEEYQLPYYDLVPSDPSIEEMRKVVCDQKLRPNIPNWWQSYEALRVMGKMMRECWYAN
GAARLTALRIKKTLSQLSVQEDVKI
>hsa:91039
MRKVKKLRLDKENTGSWRSFSLNSEGAERMATTGTPTADRGDAAATDDPAARFQVQKHSW
DGLRSIIHGSRKYSGLIVNKAPHDFQFVQKTDESGPHSHRLYYLGMPYGSRENSLLYSEI
PKKVRKEALLLLSWKQMLDHFQATPHHGVYSREEELLRERKRLGVFGITSYDFHSESGLF
LFQASNSLFHCRDGGKNGFMVSPMKPLEIKTQCSGPRMDPKICPADPAFFSFINNSDLWV
ANIETGEERRLTFCHQGLSNVLDDPKSAGVATFVIQEEFDRFTGYWWCPTASWEGSEGLK
TLRILYEEVDESEVEVIHVPSPALEERKTDSYRYPRTGSKNPKIALKLAEFQTDSQGKIV
STQEKELVQPFSSLFPKVEYIARAGWTRDGKYAWAMFLDRPQQWLQLVLLPPALFIPSTE
NEEQRLASARAVPRNVQPYVVYEEVTNVWINVHDIFYPFPQSEGEDELCFLRANECKTGF
CHLYKVTAVLKSQGYDWSEPFSPGEDEFKCPIKEEIALTSGEWEVLARHGSKIWVNEETK
LVYFQGTKDTPLEHHLYVVSYEAAGEIVRLTTPGFSHSCSMSQNFDMFVSHYSSVSTPPC
VHVYKLSGPDDDPLHKQPRFWASMMEAASCPPDYVPPEIFHFHTRSDVRLYGMIYKPHAL
QPGKKHPTVLFVYGGPQVQLVNNSFKGIKYLRLNTLASLGYAVVVIDGRGSCQRGLRFEG
ALKNQMGQVEIEDQVEGLQFVAEKYGFIDLSRVAIHGWSYGGFLSLMGLIHKPQVFKVAI
AGAPVTVWMAYDTGYTERYMDVPENNQHGYEAGSVALHVEKLPNEPNRLLILHGFLDENV
HFFHTNFLVSQLIRAGKPYQLQIYPNERHSIRCPESGEHYEVTLLHFLQEYL

>hsa:9150
MKGLCAECGQDLTQLQSKNGKQQVPLSTATVSMVHSVPELMVSSEQAEQLGREDQQRLHR
NRKLVLMVDLDQTLIHTTEQHCQQMSNKGIFHFQLGRGEPMLHTRLRPHCKDFLEKIAKL
YELHVFTFGSRLYAHTIAGFLDPEKKLFSHRILSRDECIDPFSKTGNLRNLFPCGDSMVC
IIDDREDVWKFAPNLITVKKYVYFQGTGDMNAPPGSRESQTRKKVNHSRGTEVSEPSPPV
RDPEGVTQAPGVEPSNGLEKPARELNGSEAATPRDSPRPGKPDERDIWPPAQAPTSSQEL
AGAPEPQGSCAQGGRVAPGQRPAQGATGTDLDFDLSSDSESSSESEGTKSSSSASDGESE
GKRGRQKPKAAPEGAGALAQGSSLEPGRPAAPSLPGEAEPGAHAPDKEPELGGQEEGERD
GLCGLGNGCADRKEAETESQNSELSGVTAGESLDQSMEEEEEEDTDEDDHLIYLEEILVR
VHTDYYAKYDRYLNKEIEEAPDIRKIVPELKSKVLADVAIIFSGLHPTNFPIEKTREHYH
ATALGAKILTRLVLSPDAPDRATHLIAARAGTEKVLQAQECGHLHVVNPDWLWSCLERWD
KVEEQLFPLRDDHTKAQRENSPAAFPDREGVPPTALFHPMPVLPKAQPGPEVRIYDSNTG
KLIRTGARGPPAPSSSLPIRQEPSSFRAVPPPQPQMFGEELPDAQDGEQPGPSRRKRQPS
MSETMPLYTLCKEDLESMDKEVDDILGEGSDDSDSEKRRPEEQEEEPQPRKPGTRRERTL
GAPASSERSAAGGRGPRGHKRKLNEEDAASESSRESSNEDEGSSSEADEMAKALEAELND
LM
>hsa:93
MTAPWVALALLWGSLCAGSGRGEAETRECIYYNANWELERTNQSGLERCEGEQDKRLHCY
ASWRNSSGTIELVKKGCWLDDFNCYDRQECVATEENPQVYFCCCEGNFCNERFTHLPEAG
GPEVTYEPPPTAPTLLTVLAYSLLPIGGLSLIVLLAFWMYRHRKPPYGHVDIHEDPGPPP
PSPLVGLKPLQLLEIKARGRFGCVWKAQLMNDFVAVKIFPLQDKQSWQSEREIFSTPGMK
HENLLQFIAAEKRGSNLEVELWLITAFHDKGSLTDYLKGNIITWNELCHVAETMSRGLSY
LHEDVPWCRGEGHKPSIAHRDFKSKNVLLKSDLTAVLADFGLAVRFEPGKPPGDTHGQVG
TRRYMAPEVLEGAINFQRDAFLRIDMYAMGLVLWELVSRCKAADGPVDEYMLPFEEEIGQ
HPSLEELQEVVVHKKMRPTIKDHWLKHPGLAQLCVTIEECWDHDAEARLSAGCVEERVSL
IRRSVNGTTSDCLVSLVTSVTNVDLPPKESSI
>hsa:93650
MAGLGFWGHPAGPLLLLLLLVLPPRALPEGPLVFVALVFRHGDRAPLASYPMDPHKEVAS
TLWPRGLGQLTTEGVRQQLELGRFLRSRYEAFLSPEYRREEVYIRSTDFDRTLESAQANL
AGLFPEAAPGSPEARWRPIPVHTVPVAEDKLLRFPMRSCPRYHELLREATEAAEYQEALE
GWTGFLSRLENFTGLSLVGEPLRRAWKVLDTLMCQQAHGLPLPAWASPDVLRTLAQISAL
DIGAHVGPPRAAEKAQLTGGILLNAILANFSRVQRLGLPLKMVMYSAHDSTLLALQGALG
LYDGHTPPYAACLGFEFRKHLGNPAKDGGNVTVSLFYRNDSAHLPLPLSLPGCPAPCPLG
RFYQLTAPARPPAHGVSCHGPYEAAIPPAPVVPLLAGAVAVLVALSLGLGLLAWRPGCLR
ALGGPV
>hsa:9388
MSNSVPLLCFWSLCYCFAAGSPVPFGPEGRLEDKLHKPKATQTEVKPSVRFNLRTSKDPE
HEGCYLSVGHSQPLEDCSFNMTAKTFFIIHGWTMSGIFENWLHKLVSALHTREKDANVVV
VDWLPLAHQLYTDAVNNTRVVGHSIARMLDWLQEKDDFSLGNVHLIGYSLGAHVAGYAGN
FVKGTVGRITGLDPAGPMFEGADIHKRLSPDDADFVDVLHTYTRSFGLSIGIQMPVGHID
IYPNGGDFQPGCGLNDVLGSIAYGTITEVVKCEHERAVHLFVDSLVNQDKPSFAFQCTDS
NRFKKGICLSCRKNRCNSIGYNAKKMRNKRNSKMYLKTRAGMPFRVYHYQMKIHVFSYKN
MGEIEPTFYVTLYGTNADSQTLPLEIVERIEQNATNTFLVYTEEDLGDLLKIQLTWEGAS
QSWYNLWKEFRSYLSQPRNPGRELNIRRIRVKSGETQRKLTFCTEDPENTSISPGRELWF

RKCRDGWRMKNETSPTVELP

>hsa:94

MTLGSPRKGLLMLLMALVTQGDPVKPSRGPLVTCTCESPHCKGPTCRGAWCTVVLVREEG
RHPQEHRGCGNLHRELCRGRPTEFVNHYCCDSHLCNHNVSLVLEATQPPSEQPGTDGQLA
LILGPVLALLALVALGVLGLWHVRRRQEKQRGLHSELGESSLILKASEQGDSMLGDLLDS
DCTTGSGSGLPFLVQRTVARQVALVECVGKGRYGEVWRGLWHGESVAVKIFSSRDEQSWF
RETEIYNTVLLRHDNILGFIASDMTSRNSSTQLWLITHYHEHGSLYDFLQRQTLEPHLAL
RLAVSAACGLAHLHVEIFGTQGKPAIAHRDFKSRNVLVKSNLQCCIADLGLAVMHSQGSD
YLDIGNNPRVGTKRYMAPEVLDEQIRTDCFESYKWTDIWAFGLVLWEIARRTIVNGIVED
YRPPFYDVVPNDPSFEDMKKVVCVDQQTPTIPNRLAADPVLSGLAQMMRECWYPNPSARL
TALRIKKTLQKISNSPEKPKVIQ

>hsa:9420

MAGEVSAATGRFSLERLGLPGLALAAALLLLALCLLVRRTRRPGEPPLIKGWLPYLGVVL
NLRKDPLRFMKTLQKQHGDTFTVLLGGKYITFILDPFQYQLVIKNHKQLSFRVFSNKLLE
KAFSISQLQKNHDMNDELHLCYQFLQGKSLDILLESMMQNLKQVFEPQLLKTTSWDTAEL
YPFCSSIIFEITFTTIYGKVIVCDNNKFISELRDDFLKFDDKFAYLVSNIPIELLGNVKS
IREKIIKCFSSEKLAKMQGWSEVFQSRQDVLEKYYVHEDLEIGAHHLGFLWASVANTIPT
MFWAMYYLLRHPEAMAAVRDEIDRLLQSTGQKKGSGFPIHLTREQLDSLICLESSIFEAL
RLSSYSTTIRFVEEDLTLSSETGDYCVRKGDLVAIFPPVLHGDPEIFEAPEEFRYDRFIE
DGKKKTTFFKRGKKLKCYLMPFGTGTSKCPGRFFALMEIKQLLVILLTYFDLEIIDDKPI
GLNYSRLLFGIQYPDSDVLFRYKVKS

>hsa:9563

MWNMLIVAMCLALLGCLQAQELQGHVSIILLGATGDLAKKYLWQGLFQLYLDEAGRGHSF
SFHGAALTAPKQGQELMAKALESLSCPKDMAPSHCAEHKDQFLQLSQYRQLKTAEDYQAL
NKDIEAQLQHAGLREAGRIFYFSVPPFAYEDIARNINSSCRPGPGAWLRVVLEKPFGHDH
FSAQQLATELGTFFQEEEMYRVDHYLGKQAVAQILPFRDQNRKALDGLWNRHHVERVEII
MKETVDAEGRTSFYEEYGVIRDVLQNHLTEVLTLVAMELPHNVSSAEAVLRHKLQVFQAL
RGLQRGSAVVGQYQSYSEQVRRELQKPDSFHSLTPTFAAVLVHIDNLRWEGVPFILMSGK
ALDERVGYARILFKNQACCVQSEKHWAAAQSQCLPRQLVFHIGHGDLGSPAVLVSRNLFR
PSLPSSWKEMEGPPGLRLFGSPLSDYYAYSPVRERDAHSVLLSHIFHGRKNFFITTENLL
ASWNFWTPLLESLAHKAPRLYPGGAENGRLLDFEFSSGRLFFSQQQPEQLVPGPGPAPMP
SDFQVLRAKYRESPLVSAWSEELISKLANDIEATAVRAVRRFGQFHLALSGGSSPVALFQ
QLATAHYGFPWAHTHLWLVDERCVPLSDPESNFQGLQAHLLQHVRIPYYNIHPMPVHLQQ
RLCAEEDQGAQIYAREISALVANSSFDLVLLGMGADGHTASLFPQSPTGLDGEQLVVLTT
SPSQPHRRMSLSLPLINRAKKVAVLVMGRMKREITTLVSRVGHEPKKWPISGVLPHSGQL
VWYMDYDAFLG

>hsa:9601

MRPRKAFLLLLLLLGLVQLLAVAGAEGPDEDSSNRENAIEDEEEEEEEDDDEEEDDLEVKE
ENGVLVLNDANFDNFVADKDTVLLEFYAPWCGHCKQFAPEYEKIANILKDKDPPIPVAKI
DATSASVLASRFDVSGYPTIKILKKGQAVDYEGSRTQEEIVAKVREVSQPDWTPPPEVTL
VLTKENFDEVVNDADIILVEFYAPWCGHCKKLAPEYEKAAKELSKRSPPIPLAKVDATAE
TDLAKRFDVSGYPTLKIFRKGRPYDYNGPREKYGIVDYMIEQSGPPSKEILTLKQVQEFL
KDGDDVIIIGVFKGESDPAYQQYQDAANNLREDYKFHHTFSTEIAKFLKVSQGQLVVMQP
EKFQSKYEPRSHMMDVQGSTQDSAIKDFVLKYALPLVGHRKVSNDAKRYTRRPLVVVYYS

VDFSFDYRAATQFWRSKVLEVAKDFPEYTFAIADEEDYAGEVKDLGLSESGEDVNAAILD
ESGKKFAMEPEEFDSDTLREFVTAFKKGKLKPVIKSQPVPKNNKGPVKVVVGKTFDSIVM
DPKKDVLIEFYAPWCGHCKQLEPVYNSLAKKYKGQKGLVIAKMDATANDVPSDRYKVEGF
PTIYFAPSGDKKNPVKFEGGDRDLEHLSKFIEEHATKLSRTKEEL
>hsa:9641
MEYCSSGSLLSVLESPENAFGLPEDEFLVVLRCVVAGMNHLRENGIVHRDIKPGNIMRLV
GEEGQSIYKLTDFGAARELDDDEKFVSVYGTEEYLHPDMYERAVLRKPQQKAFGVTVDLW
SIGVTLYHAATGSLPFIPFGGPRRNKEIMYRITTEKPAGAIAGAQRRENGPLEWSYTLPI
TCQLSLGLQSQLVPILANILEVEQAKCWGFDQFFAETSDILQRVVVHVFSLSQAVLHHIY
IHAHNTIAIFQEAVHKQTSVAPRHQEYLFEGHLCVLEPSVSAQHIAHTTASSPLTLFSTA
IPKGLAFRDPALDVPKFVPKVDLQADYNTAKGVLGAGYQALRLARALLDGQELMFRGLHW
VMEVLQATCRRTLEVARTSLLYLSSSLGTERFSSVAGTPEIQELKAAAELRSRLRTLAEV
LSRCSQNITETQESLSSLNRELVKSRDQVHEDRSIQQIQCCLDKMNFIYKQFKKSRMRPG
LGYNEEQIHKLDKVNFSHLAKRLLQVFQEECVQKYQASLVTHGKRMRVVHETRNHLRLVG
CSVAACNTEAQGVQESLSKLLEELSHQLLQDRAKGAQASPPPIAPYPSPTRKDLLLHMQE
LCEGMKLLASDLLDNNRIIERLNRVPAPPDV
>hsa:9647
MSSGAPQKSSPMASGAEETPGFLDTLLQDFPALLNPEDPLPWKAPGTVLSQEEVEGELAE
LAMGFLGSRKAPPPLAAALAHEAVSQLLQTDLSEFRKLPREEEEEEEDDDEEEKAPVTLL
DAQSLAQSFFNRLWEVAGQWQKQVPLAARASQRQWLVSIHAIRNTRRKMEDRHVSLPSFN
QLFGLSDPVNRAYFAVFDGHGGVDAARYAAVHVHTNAARQPELPTDPEGALREAFRRTDQ
MFLRKAKRERLQSGTTGVCALIAGATLHVAWLGDSQVILVQQGQVVKLMEPHRPERQDEK
ARIEALGGFVSHMDCWRVNGTLAVSRAIGDVFQKPYVSGEADAASRALTGSEDYLLLACD
GFFDVVPHQEVVGLVQSHLTRQQGSGLRVAEELVAAARERGSHDNITVMVVFLRDPQELL
EGGNQGEGDPQAEGRRQDLPSSLPEPETQAPPRS
>hsa:9945
MCGIFAYMNYRVPRTRKEIFETLIKGLQRLEYRGYDSAGVAIDGNNHEVKERHIQLVKKR
GKVKALDEELYKQDSMDLKVEFETHFGIAHTRWATHGVPSAVNSHPQRSDKGNEFVVIHN
GIITNYKDLRKFLESKGYEFESETDTETIAKLIKYVFDNRETEDITFSTLVERVIQQLEG
AFALVFKSVHYPGEAVATRRGSPLLIGVRSKYKLSTEQIPILYRTCTLENVKNICKTRMK
RLDSSACLHAVGDKAVEFFFASDASAIIEHTNRVIFLEDDDIAAVADGKLSIHRVKRSAS
DDPSRAIQTLQMELQQIMKGNFSAFMQKEIFEQPESVFNTMRGRVNFETNTVLLGGLKDH
LKEIRRCRRLIVIGCGTSYHAAVATRQVLEELTELPVMVELASDFLDRNTPVFRDDVCFF
ISQSGETADTLLALRYCKDRGALTVGVTNTVGSSISRETDCGVHINAGPEIGVASTKAYT
SQFISLVMFGLMMSEDRISLQNRRQEIIRGLRSLPELIKEVLSLEEKIHDLALELYTQRS
LLVMGRGYNYATCLEGALKIKEITYMHSEGILAGELKHGPLALIDKQMPVIMVIMKDPCF
AKCQNALQQVTARQGRPIILCSKDDTESSKFAYKTIELPHTVDCLQGILSVIPLQLLSFH
LAVLRGYDVDFPRNLAKSVTVE
>hsa:9955
MAPPGPASALSTSAEPLSRSIFRKFLLMLCSLLTSLYVFYCLAERCQTLSGPVVGLSGGG
EEAGAPGGGVLAGGPRELAVWPAAAQRKRLLQLPQWRRRRPPAPRDDGEEAAWEEESPGL
SGGPGGSGAGSTVAEAPPGTLALLLDEGSKQLPQAIIIGVKKGGTRALLEFLRVHPDVRA
VGAEPHFFDRSYDKGLAWYRDLMPRTLDGQITMEKTPSYFVTREAPARISAMSKDTKLIV
VVRDPVTRAISDYTQTLSKRPDIPTFESLTFKNRTAGLIDTSWSAIQIGIYAKHLEHWLR

HFPIRQMLFVSGERLISDPAGELGRVQDFLGLKRIITDKHFYFNKTKGFPCLKKAEGSSR
PHCLGKTKGRTHPEIDREVVRRLREFYRPFNLKFYQMTGHDFGWDG
>hsa:94009
MAENAAPGLISELKLAVPWGHIAAKAWGSLQGPPVLCLHGWLDNASSFDRLIPLLPQDFY
YVAMDFGGHGLSSHYSPGVPYYLQTFVSEIRRVVAALKWNRFSILGHSFGEYSGQELTGP
GVGLGGKDRSCCPTPFPCTYEICKER
